# An eccentric rod-like linear connection of two heterocycles: synthesis of pyridine *trans*-tetrafluoro-λ^6^-sulfanyl triazoles†
†Electronic supplementary information (ESI) available. CCDC 1823578 and 1823581. For ESI and crystallographic data in CIF or other electronic format see DOI: 10.1039/c8sc01216d


**DOI:** 10.1039/c8sc01216d

**Published:** 2018-05-14

**Authors:** Prajwalita Das, Kiyoteru Niina, Tomoya Hiromura, Etsuko Tokunaga, Norimichi Saito, Norio Shibata

**Affiliations:** a Department of Nanopharmaceutical Sciences and Department of Life Science and Applied Chemistry , Nagoya Institute of Technology , Gokiso, Showa-ku , Nagoya 466-8555 , Japan . Email: nozshiba@nitech.ac.jp; b Pharmaceutical Division , Ube Industries, Ltd. , Seavans North Bldg, 1-2-1 Shibaura, Minato-ku , Tokyo 105-8449 , Japan; c Institute of Advanced Fluorine-Containing Materials , Zhejiang Normal University , 688 Yingbin Avenue , 321004 Jinhua , China

## Abstract

The *trans*-tetrafluoro-λ^6^-sulfane (SF_4_) group has been utilized as a unique three-dimensional building block for the linear connection of two independent N-heterocycles, pyridines and triazoles. The linearly connected heterocyclic compounds were synthesized by thermal Huisgen 1,3-dipolar cycloaddition between previously unknown pyridine SF_4_-alkynes and readily available azides, providing a series of rod-like SF_4_-connected N-heterocycles in good to excellent yields. X-ray crystallographic analysis of the target products revealed the *trans*-geometry of the SF_4_ group, which linearly connects two independent N-heterocycles. This research will open the field of chemistry of SF_4_-connected heterocyclic compounds.

## Abstract 2

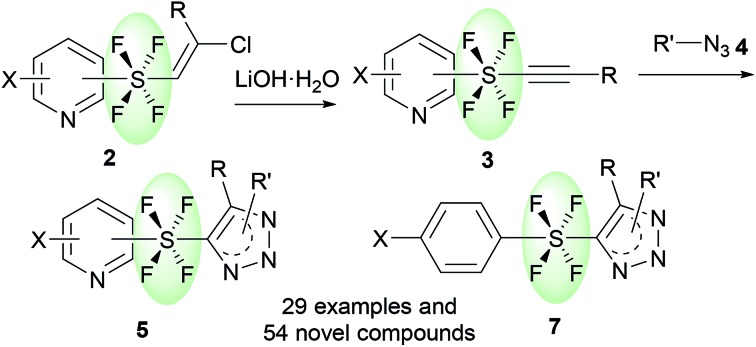
An eccentric *trans*-SF_4_ unit achieves a rod-like linear connection of two independent N-heterocycles, pyridines and triazoles.

## Introduction

**Fig. 1:**
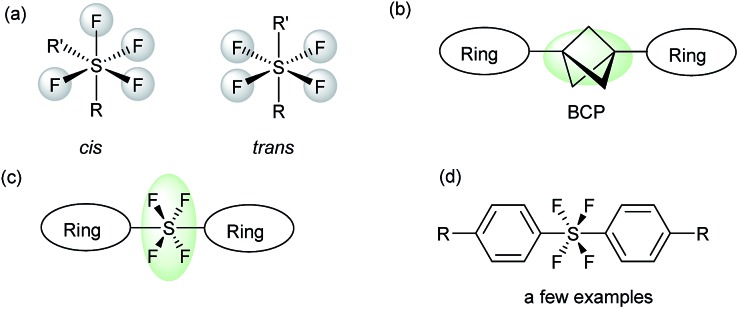
(a) SF_4_ compounds with *cis*- and *trans*-geometry. (b) Rod-like molecules with a BCP moiety. (c) Proposed rod-like molecules with a *trans*-SF_4_ moiety. (d) Bisaryltetrafluorosulfuranes.

Hypervalent sulfur fluorides belong to an interesting class of compounds, which are popular among both medicinal and material chemists alike.^1^ The pentafluoro-λ^6^-sulfane (pentafluorosulfanyl, SF_5_) group has turned out to be the front-runner of this class of compounds, with chemists realizing the varied potential of this moiety.^2^ Over the years, a steady increase can be seen in the number of publications related to SF_5_-containing compounds.^2^ On the other hand, the tetrafluoro-λ^6^-sulfane (tetrafluorosulfanyl, SF_4_) moiety, with equally potent functionality, has not been exploited enough and is highly underdeveloped.^3^ The SF_4_ moiety not only has unique physiochemical properties, which make it suitable as a unit of liquid crystals,^1^,^3^ but also has interesting geometric features that enable it to connect two independent functional groups *via* the central hypervalent sulfur atom in either the *cis* or *trans* configuration of R-SF_4_-R′ (Fig. 1a).^3^,^4^ Due to the octahedral geometry of the R-SF_4_-R′ moiety, the *trans*-SF_4_ configuration has the ability to function as a building block in the construction of linear structures *via* its axial bonds, which makes the *trans*-SF_4_ configuration highly significant.^3^,^4^ The rod-like connection by the *trans*-SF_4_ unit potentially suggests new approaches for designing novel pharmaceuticals, while also leading to sought-after fluorine-containing drug candidates. As an extension of our research on fluorine-containing heterocycles,^5^ we were interested in the development of a novel method to connect two heterocyclic rings *via* a rod-like linear linker. Linear molecules are currently receiving chemists' attention due to their material and biological applications.^6^,^7^ Bicyclo-[1.1.1]pentane (BCP) has gained the most attention in achieving linear connection^7^ (Fig. 1b). Due to the intrinsically linear framework and lipophilicity of BCP, it has been proposed as a bioisostere of a *p*-substituted benzene ring7*e* and as an alkenyl group,7*d* both of which are found in pharmaceuticals. Our present objective is to propose the *trans*-SF_4_ unit as a novel bio-isosteric unit next to BCP and to design rod-like molecules with two independent (hetero)aromatic rings (Fig. 1c).

While the initial idea of SF_4_-linked diaromatic compounds, Ar-SF_4_-Ar, appeared in 1973,3*a* followed by a handful of reports (*i.e.*, 4 papers and 1 patent),3a–e only circumstantial evidence was reported. Kirsch and co-workers in 1999 obviously isolated *trans*-SF_4_-linked aromatic building blocks based on direct fluorination of the corresponding bis(aryl)sulfide (Fig. 1d),3*d* but that method has a serious limitation, *i.e.*, the necessity of substrates substituted by *p*-nitro groups to deactivate the aromatic moiety, preventing the reaction with fluorine. Therefore, novel methodologies for *trans*-SF_4_-linked bis-aromatic compounds are highly desired. Besides, SF_4_-linked heteroaromatic building blocks have never been reported.

**Scheme 1:**
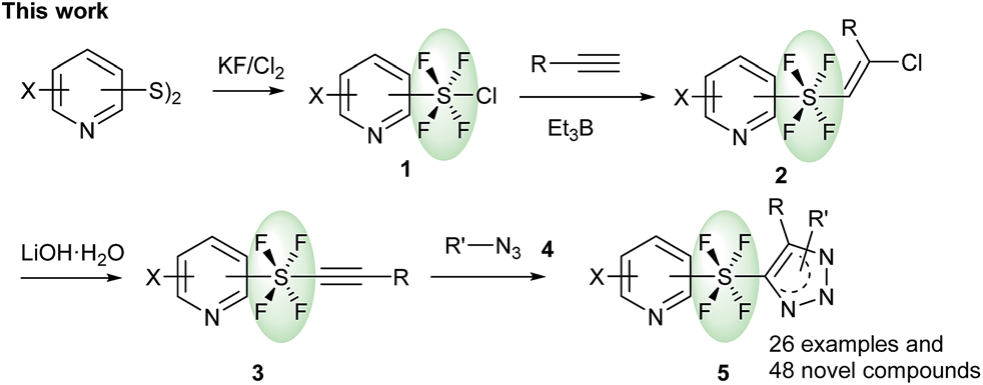
Synthesis of *trans*-SF_4_ linked pyridine and triazole compounds *via* a cycloaddition reaction.

Heterocycles, which are common skeletal components of natural products, often exhibit bioactive properties, and are thus extensively used as pharmaceuticals.^8^ Pyridines and triazoles are two of the most commonly occurring N-heterocycles in medicinal chemistry.^9^ The use of the *trans*-SF_4_ moiety as a rod-like linear linker for pyridine and triazole groups would provide a fascinating novel set of compounds, which should have a very interesting physiochemical profile due to their linear structure^6^,^7^ and the fluorinated N-heterocycles.^10^ Herein, we present the first method for the connection of two independent N-heterocyclic molecules *via* a rod-like linear *trans*-SF_4_ unit, where the axial bonds of the octahedral disubstituted SF_4_ moiety are responsible for the linear connection (Scheme 1). First, the pyridine tetrafluorosulfanyl chlorides **1** (Py-SF_4_Cl) were prepared from pyridine disulfides ((Py-S)_2_) with KF/Cl_2_ by oxidative chloro-tetrafluorination. It should however be noted that the generation of the tetrafluorosulfanyl chloride group in any organic molecule itself is challenging. The chloro-fluorination needs to be performed under a completely dry and inert atmosphere in FEP bottles, while the isolation of the product requires special equipment.2g,h,j Once the pyridine tetrafluorosulfanyl chlorides **1** were synthesized, based on our previous reports2j,3k their radical addition to alkynes was attempted to furnished pyridine *trans*-SF_4_-alkenes **2**. Then pyridine *trans*-SF_4_-alkenes **2** were converted to previously unknown pyridine SF_4_-alkynes **3** by treatment with LiOH·H_2_O. Finally, reaction of the pyridine SF_4_-alkynes **3** with azides **4** under thermal Huisgen 1,3-dipolar cycloaddition conditions provided the eccentric, three-dimensionally unique *trans*-SF_4_ linked pyridine and triazole derivatives **5** in high yields (Scheme 1).^11^ While a few examples of aryl-SF_4_-aryl were reported,^3^ this is the first example for the synthesis of heteroaryl-SF_4_-heteroaryl systems. An aryl-SF_4_-heteroaryl system was also accessed by the method. Since both heteroaryl and fluorinated moieties are sought after building blocks for drug candidates, our novel, eccentric heteroaromatic molecules should suggest new fields of drug design.

## Results and discussion

**Scheme 2:**
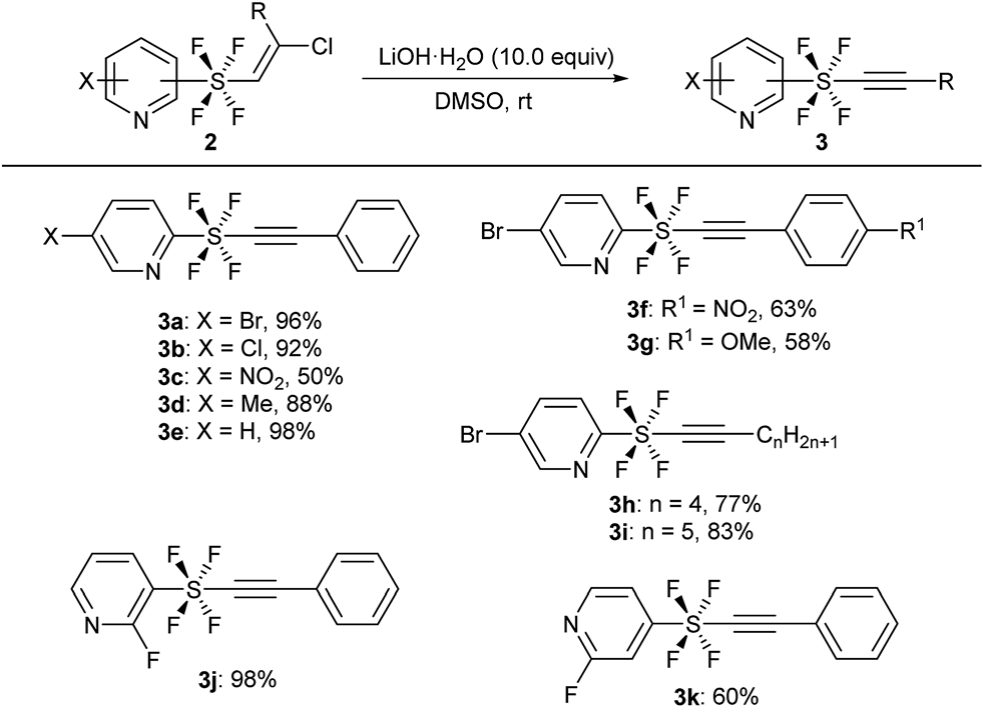
Synthesis of pyridine SF_4_-alkynes **3** from alkenes **2**. ^*a*^Reaction of **2** (1.0 equiv.) was performed in the presence of LiOH·H_2_O (10.0 equiv.) in DMSO at rt.

The key precursors, pyridine SF_4_-alkynes **3**, were prepared in good yields from the pyridine SF_4_-alkenes **2** 2j,3k by subjecting the latter to dehydrochlorination under basic conditions. Thus, treatment of pyridine SF_4_ chloroalkenes **2** with an excess amount of LiOH·H_2_O in DMSO at room temperature furnished the desired pyridine SF_4_ alkynes **3**. A wide variety of functional groups including halogens (Br, Cl, and F), an electron-withdrawing NO_2_ group, and an electron-donating methyl group in the pyridine ring were well tolerated under these strong basic conditions. All the positions of the SF_4_ unit on the pyridine ring, namely the *ortho*-, *meta*- and *para*-SF_4_-pyridines **2**, were accepted for this transformation. The desired pyridine SF_4_ alkynes **3** with an aryl or alkyl group at the terminal position were also obtained in good to excellent yields (Scheme 2).

With the precursor pyridine SF_4_-alkynes **3** in hand, we first examined the reaction conditions that would allow the azide/alkyne cycloaddition to take place (see the ESI for details†). Using alkyne **3a** and benzyl azide **4a**, initially ruthenium catalyst^12^ Cp*Ru(PPh_3_)_2_Cl_2_ was used in toluene at 80 °C and 110 °C to give target product **5a** in 24% and 32% yield respectively, as a mixture of 1,4- and 1,5-disubstituted isomers (isomer A and isomer B, entries 1 and 2). When the catalyst loading of Cp*Ru(PPh_3_)_2_Cl_2_ was increased, the yield decreased (entry 3) and a subsequent decrease of the catalyst increased the yield to 56% (entry 4). We then realized that our reaction does not require an Ru catalyst, but undergoes a thermal cycloaddition, where the catalyst initially led to the start of material decomposition. Running the reaction in the absence of a catalyst gave the product satisfactorily in 83% yield (entry 5). Being a thermal reaction, the regioisomers A and B were obtained in a 2 : 1 ratio.^13^ The formation of 1,4-disubstituted isomer A was slightly preferred because it avoided the steric repulsions between the bulky SF_4_ and the benzyl group (Table 1).

**Table 1:** Optimization of reaction conditions^*a*^

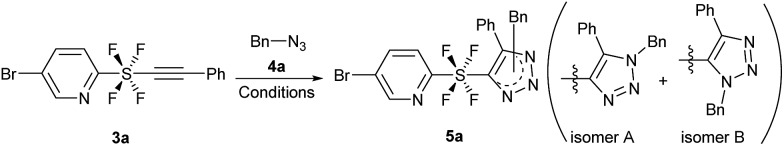		
Entry	Conditions	**5a**
1	**3a** (1.0 equiv.), **4a** (3.0 equiv.), Cp*Ru(PPh_3_)_2_Cl_2_ (10 mol%) in toluene at 80 °C	24%,^*b*^ 1.5 : 1^*c*^
2	**3a** (1.0 equiv.), **4a** (3.0 equiv.), Cp*Ru(PPh_3_)_2_Cl_2_ (10 mol%) in toluene at 110 °C	32%,^*b*^ 1.5 : 1^*c*^
3	**3a** (1.0 equiv.), **4a** (3.0 equiv.), Cp*Ru(PPh_3_)_2_Cl_2_ (20 mol%) in toluene at 110 °C	10%,^*b*^ 1.5 : 1^*c*^
4	**3a** (1.0 equiv.), **4a** (3.0 equiv.), Cp*Ru(PPh_3_)_2_Cl_2_ (5 mol%) in toluene at 110 °C	56%,^*b*^ 1.5 : 1^*c*^
5	**3a** (1.0 equiv.), **4a** (3.0 equiv.), in toluene at 110 °C	83%,^*b*^ 2 : 1^*c*^

^*a*^Reaction was performed at the 0.1 mmol scale at the given conditions for 24 h.

^*b*^Total yield of both regioisomers from ^19^F NMR.

^*c*^Ratio of two regioisomers A and B.

**Scheme 3:**
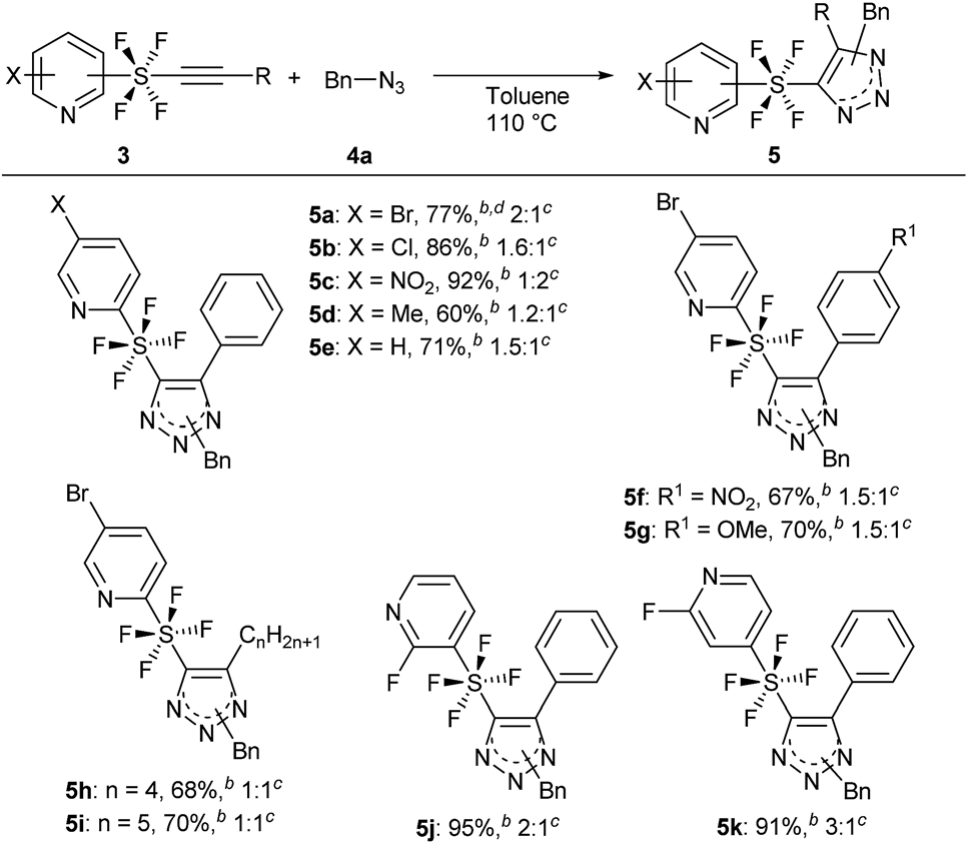
Evaluation of substrate scope by changing alkynes **3**. ^*a*^Reaction of **3** (0.5 mmol) was performed with **4a** (1.5 mmol) in toluene at 110 °C for 24 h unless otherwise mentioned. ^*b*^Isolated yield. ^*c*^Ratio of regioisomers A and B. ^*d*^Reaction was performed at a 1 g scale of **3**.

With the optimized reaction conditions in hand, we began the substrate screening by modifying the SF_4_-alkynes **3** (Scheme 3). Changing the halogen on the pyridine ring to Cl gave product **5b** in 86% yield with a ratio of 1.6 : 1 (isomers A : B). Having the electron-withdrawing NO_2_ group on pyridine gave **5c** in an excellent yield of 92%. It is of interest that in this case we observed a reversal in selectivity, with a preference for the 1,5-disubstituted product (isomers A : B = 1 : 2). 4-Me pyridine SF_4_-alkyne **3d** and unsubstituted pyridine SF_4_-alkyne **3e** both sustained the reaction to give the desired triazole products **5d** and **5e** in 60% and 71% yield, respectively. Next, we analysed the effect of the electron-withdrawing NO_2_ and electron-donating OMe on the phenyl ring of the alkyne moiety (**3f** and **3g**). There did not appear to be any drastic effect of the substituents as both alkynes gave products **5f** and **5g** in 67% and 70% yield, respectively with a 1.5 : 1 ratio (isomers A : B). Replacing the aromatic ring by an aliphatic straight chain (*n*-butyl or *n*-pentyl) also gave products **5h** and **5i** in 68% and 70% yield, respectively. We further expanded our reaction by employing *m*-SF_4_ pyridine alkyne **3j** and *p*-SF_4_ pyridine alkyne **3k** as substrates. Both alkynes gave products **5j** and **5k** in excellent yields of 95% and 91%, respectively. The selectivity of the regioisomers increased slightly to 3 : 1. This reaction could be reproduced at a 1 g scale of alkyne **3a** to **5a** without any loss of yield.

**Scheme 4:**
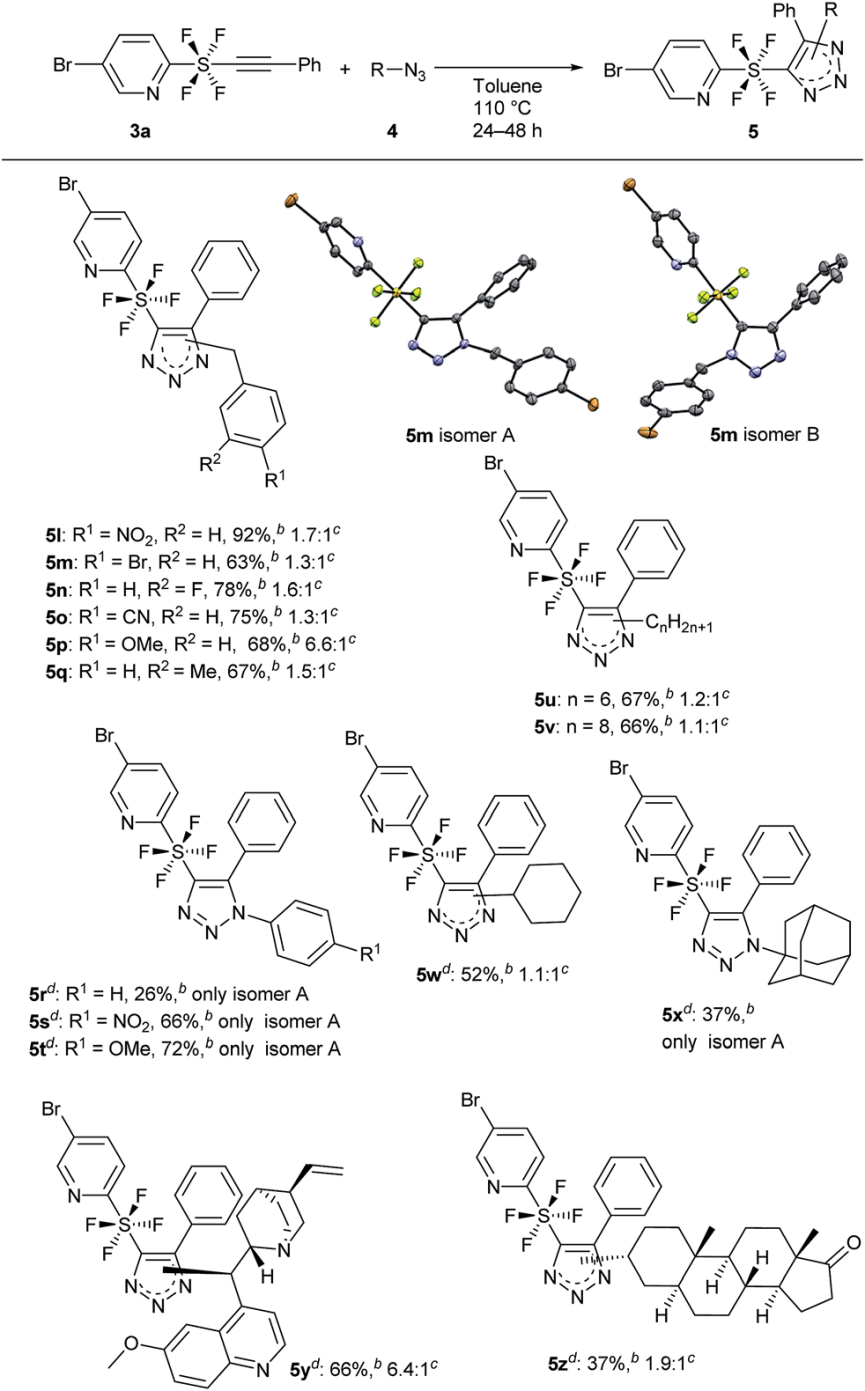
Evaluation of substrate scope by changing azide **3**. Molecular structures of **5m** with thermal ellipsoids set to 50% probability: **5m** isomer A (CCDC ; 1823578 †) and **5m** isomer B (CCDC ; 1823581 †). ^*a*^Reaction of **3a** (0.5 mmol) was performed with **4** (1.5 mmol) in toluene at 110 °C for 24 h unless otherwise mentioned. ^*b*^Isolated yield. ^*c*^Ratio of the two regioisomers A and B. ^*d*^Reaction was performed for 48 h.

The substrate scope of azides **4** was further investigated for the cycloaddition (Scheme 4). Changing the substituents on the benzene ring of the benzyl azides **4** gave good results. The presence of an electron-withdrawing group was sustained well to give products **5** having NO_2_ (**5l**), Br (**5m**), and F (**5n**) on the benzene ring in good to excellent yields. Even a CN group was borne to give the triazole product **5o** in 75% yield. An electron-donating group also gave products **5p** (OMe) and **5q** (Me) in 67–68% yields. It is noteworthy that, in the case of **5p**, selectivity for the regioisomers increased to 6.6 : 1. The single crystal X-ray structures of each of the regioisomers of **5m**, isomer A and isomer B, clearly revealed the specific regiochemistry of the octahedral sulfur centre, connecting the pyridine and the triazole parts linearly with its axial bonds and four fluorines occupying the equatorial plane. Switching from benzyl to phenyl azide revealed that the 1,4-disubstituted products **5r–t** (isomer A) formed exclusively. This was possibly due to the high steric hindrance of the SF_4_ moiety and phenyl ring in the 1,5-disubstituted product (isomer B). Aliphatic azides **4** having 6 and 8 carbons were used and the respective products **5u** and **5v** were obtained (67–66% yields) with almost no regioselectivity. Cyclohexyl azide gave product **5w** in moderate (52%) yield, while very bulky adamantyl azide gave exclusively the 1,4-disubstituted product **5x** (isomer A) regioselectively in 37% yield. The cycloaddition reaction also proceeded well with two azide derivatives, quinine and epiandrosterone, to give products **5y** and **5z** respectively, having a drug-like structure, in 37% to 67% yields. Isomer A of **5y** was in fact positively obtained selectively over isomer B (6.4 : 1).

**Scheme 5:**
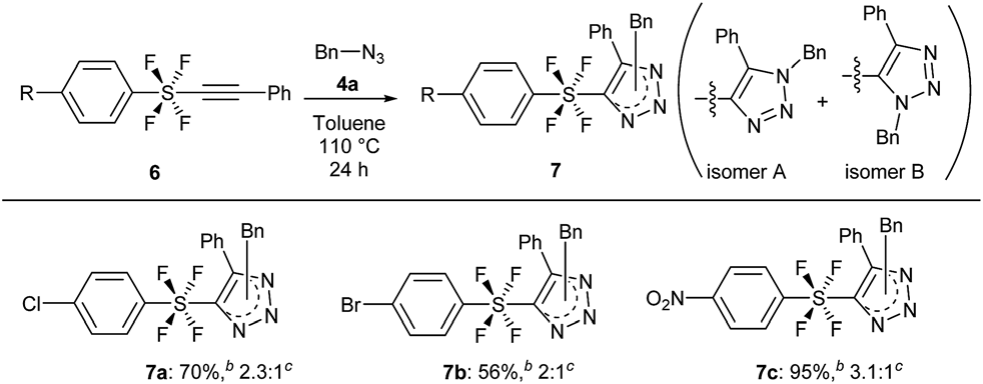
Cycloaddition with benzene SF_4_-alkynes **6** and azide **4a**. ^*a*^Reaction of **6** (0.5 mmol) was performed with **4a** (1.5 mmol) in toluene at 110 °C for 24 h. ^*b*^Isolated yield. ^*c*^Ratio of the two regioisomers A and B.

The cycloaddition reaction was also extended to benzene SF_4_-alkynes **6**, which were synthesized according to a reported procedure3*i* (see the ESI for details†). The reactions of these alkynes **6** were completely feasible with the simple benzyl azide **4a** (Scheme 5). While the Br and Cl-benzene SF_4_-alkyne **6a** and **6b** gave products **7a** and **7b** in good yields of 56% and 70% respectively (isomer A : B ratio 2 : 1 and 2.3 : 1 respectively), the NO_2_-benzene SF_4_-alkyne **6c** underwent the reaction to provide product **7c** in an excellent yield of 95% with a ratio of 3.1 : 1 (isomers A : B). Like compounds **5**, **7** are also the first examples of aryl-SF_4_-heteroaryl systems.

**Scheme 6:**
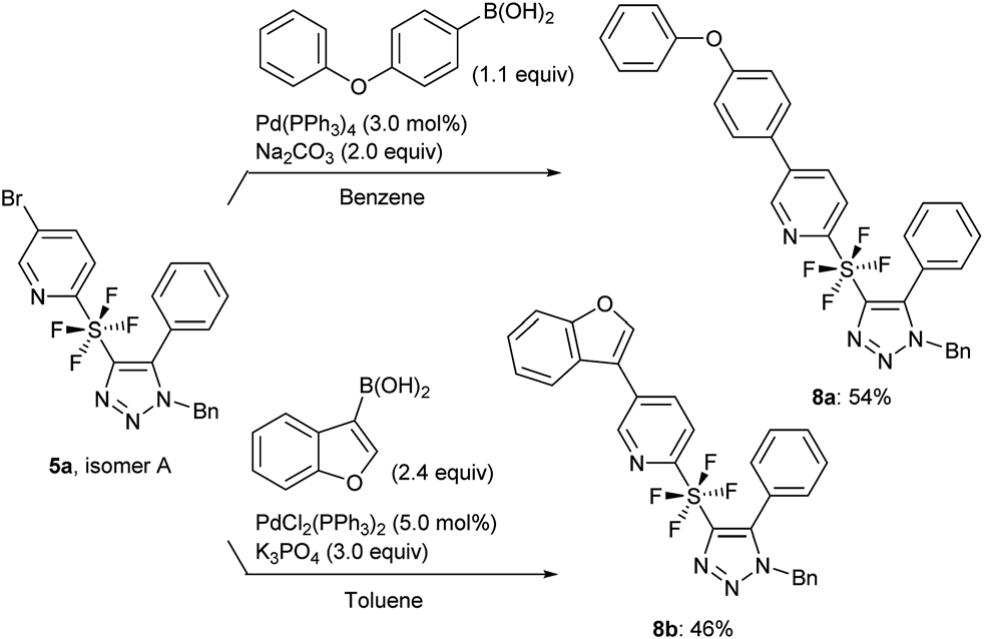
Coupling of **5a** with boronic acids.

Following the synthesis of the cycloaddition products, we used isomer A of **5a** for a further application. Suzuki coupling *via* the bromo substituent on pyridine was attempted.^14^ The use of phenoxyphenyl and benzofuran boronic acids, which are aromatic and heteroaromatic substrates, gave the coupled products **8a** and **8b** in 54% and 46% yield, respectively (Scheme 6).

## Conclusion

In conclusion, we designed and synthesized novel tetrafluoro-λ^6^-sulfanes **5** having a linear connection between pyridine and triazole rings *via* the *trans*-tetrafluoro-λ^6^-sulfane (SF_4_) moiety. The desired compounds **5** were obtained by thermal Huisgen 1,3-dipolar cycloaddition between pyridine SF_4_-alkynes **3** and azides **4**. Benzene-SF_4_-triazole product **7** was also synthesized using the same protocol. Further coupling of **5** with boronic acids was also possible. These compounds are eccentric fluorinated heterocycles with potential bioactive and surface properties. Further investigations on the application of **5** are underway.

## Conflicts of interest

There are no conflicts to declare.

## Supplementary Material

Supplementary information

Crystal structure data
