# Supplementary material for: An eccentric rod-like linear connection of two heterocycles: synthesis of pyridine trans-tetrafluoro-λ6-sulfanyl triazoles
Source: Chem Sci. 2018 May 14;9(22):4931–6. doi: 10.1039/c8sc01216d (PMC5994873; doi:10.1039/c8sc01216d)
Supplement: Supplementary file 1 [file SC-009-C8SC01216D-s001.pdf]

Electronic Supplementary Material (ESI) for Chemical Science.
This journal is © The Royal Society of Chemistry 2018

Electronic Supplementary Information

An eccentric rod-like linear connection of two heterocycles: Synthesis of pyridine *trans*-tetrafluoro-λ^6-sulfanyl triazoles

Prajwalita Das,[a] Kiyoteru Niina,[a] Tomoya Hiromura,[a] Etsuko Tokunaga,[a] Norimichi Saito[b] and Norio Shibata*[a,c]

[a] Department of Nanopharmaceutical Sciences and Department of Life Science and Applied Chemistry, Nagoya Institute of Technology, Gokiso, Showa-ku, Nagoya 466-8555, Japan.

[b] Pharmaceutical Division, Ube Industries, Ltd. Seavans North Bldg., 1-2-1 Shibaura, Minato-ku, Tokyo 105-8449, Japan.

[c] Institute of Advanced Fluorine-Containing Materials, Zhejiang Normal University, 688 Yingbin Avenue, 321004 Jinhua, China.

Contents

General Information.....S1

Optimization Table S1.....S2

Scheme S1 Metal catalysed reaction.....S3

Synthesis of Pyridine-SF_4-alkyne **3**.....S4

Synthesis of Benzene-SF_4-alkyne **6**.....S7

Synthesis of the Pyridine/Benzene-SF_4-triazole **5** and **7**.....S7

Reference.....S27

X-ray crystal structure.....S28

NMR spectra.....S29

General information

All reactions were performed in oven-dried glassware under positive pressure of nitrogen unless otherwise mentioned. Solvents were transferred *via* syringe and were introduced into the reaction vessels though a rubber septum. All of the reactions were monitored by thin-layer chromatography (TLC) carried out on 0.25 mm Merck silica gel (60-F254). The TLC plates were visualized with UV light. Products were purified by column chromatography carried out on columns packed with silica gel (60N spherical neutral size 63-210 μm). The ^{1}H NMR (300 MHz) and ^{19}F NMR (282 MHz) spectra were recorded for solution in $CDCl_3$ and $(CD_3)_2CO$ on a Varian Mercury 300. ^{13}C NMR (125 MHz) spectra for solution in $CDCl_3$ and $(CD_3)_2CO$ were recorded on a BRUKER 500 UltraShield^{TR}. Chemical shifts (δ) are expressed in ppm downfield from TMS (δ = 0.00) for ^{1}H and C_6F_6 [δ = −162.2 ($CDCl_3$) or −163.5 ($(CD_3)_2CO$)] as an internal standard ^{19}F NMR. For ^{13}C NMR, $CDCl_3$ (δ = 77.16) or $(CD_3)_2CO$ (δ = 29.84) is referred as residual standard. High resolution mass spectrometry was recorded on a SHIMADZU GCMS-QP5050A (EI-MS) and SHIMAZU LCMS-2020 (ESI-MS and APCI-MS). Infrared spectra were recorded on a JASCO FT/IR-4100 spectrometer. Melting points were recorded on a BUCHI M-565. Chemicals were purchased and used without further purification unless otherwise noted. Solvents benzene, toluene, dioxane, DMF and THF were dried and distilled before use.

Table S1. Optimization of reaction conditions.

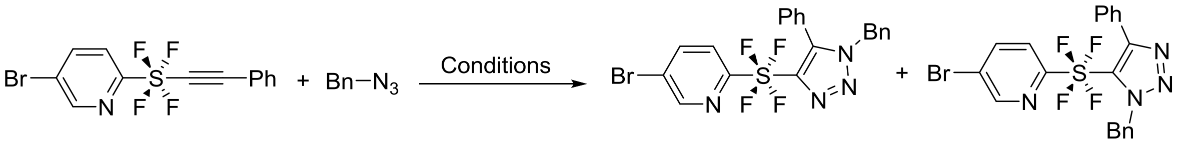

Entry	Conditions[a]	Result
1	**3a** (2.0 equiv), **4a** (1.0 equiv), $Cp^*Ru(PPh_3)_2Cl_2$ (10 mol%) in benzene at 80 °C	Traces
2	**3a** (1.0 equiv), **4a** (1.0 equiv), $Cp^*Ru(PPh_3)_2Cl_2$ (10 mol%) in benzene at 80 °C	9%,[b] 1:1[c]
3	**3a** (1.0 equiv), **4a** (3.0 equiv), $Cp^*Ru(PPh_3)_2Cl_2$ (10 mol%) in benzene at 80 °C	19%,[b] 1:1[c]
4	**3a** (1.0 equiv), **4a** (3.0 equiv), $Cp^*Ru(PPh_3)_2Cl_2$ (10 mol%) in toluene at 80 °C	24%,[b] 1.5:1[c]
5	**3a** (1.0 equiv), **4a** (3.0 equiv), $Cp^*Ru(PPh_3)_2Cl_2$ (10 mol%) in dioxane at 80 °C	23%,[b] 1.5:1[c]
6	**3a** (1.0 equiv), **4a** (3.0 equiv), $Cp^*Ru(PPh_3)_2Cl_2$ (10 mol%) in DMF at 80 °C	17%,[b] 1.4:1[c]
7	**3a** (1.0 equiv), **4a** (3.0 equiv), $Cp^*Ru(PPh_3)_2Cl_2$ (10 mol%) in THF at 80 °C	19%,[b] 1.7:1[c]
8	**3a** (1.0 equiv), **4a** (3.0 equiv), $Cp^*Ru(PPh_3)_2Cl_2$ (10 mol%) in toluene at 110 °C	32%,[b] 1.5:1[c]
9	**3a** (1.0 equiv), **4a** (3.0 equiv), $Cp^*Ru(PPh_3)_2Cl_2$ (20 mol%) in toluene at 110 °C	10%,[b] 1.5:1[c]
10	**3a** (1.0 equiv), **4a** (3.0 equiv), $Cp^*Ru(PPh_3)_2Cl_2$ (5 mol%) in toluene at 110 °C	56%,[b] 1.5:1[c]
11	**3a** (1.0 equiv), **4a** (3.0 equiv), in toluene at 110 °C	83%,[b] 2:1[c]

[a] Reactions were performed at 0.1 mmol scale at the given conditions for 24 h. [b] Total yield of **5a** from ^{19}F NMR. [c] Ratio of the two regioisomers A and B.

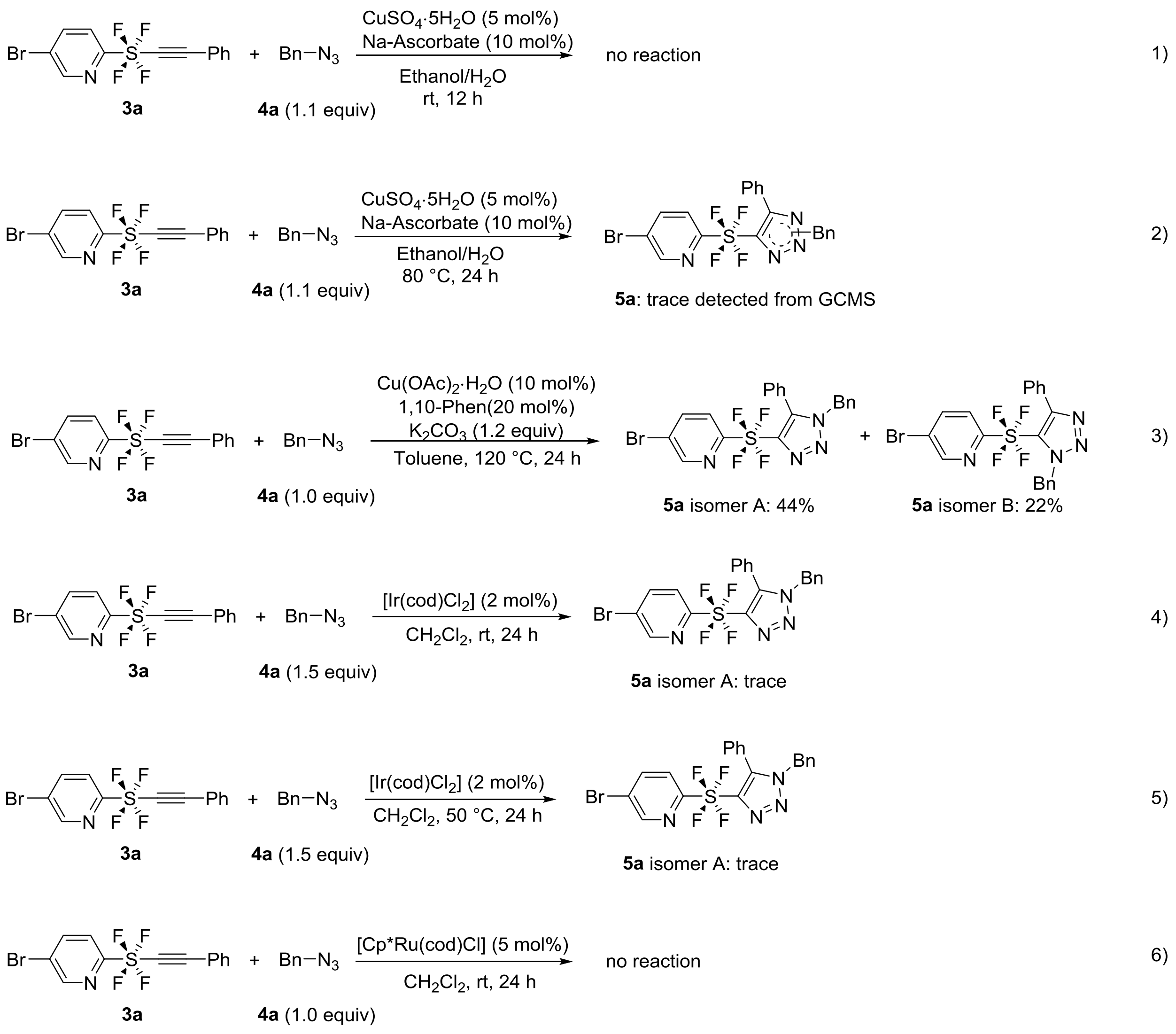

Scheme S1. Metal catalysed reactions.

The click reaction was also attempted under more metal catalysed conditions. Reaction 1 was performed under the typical Cu catalysed click reaction condition,[1] but it did not proceed even after 12 h. When the reaction temperature was elevated to 80 °C for 24 h (reaction 2), trace amount of the product could be detected from GCMS. In reaction 3,[2] the product was formed but, no increased selectivity was observed for the regioisomers (A:B = 2:1). Thus, we concluded that, there was no copper assistance in reactions 2 and 3, but they were solely driven by the thermal energy.

We also attempted the reaction under Ir catalysis[3] at room temperature and elevated 50 °C but, could see only traces of **5a** isomer A after 24 h (reaction 5 and 6). On the other hand, when we changed the Ru catalyst,[4] the reaction didn't proceed (reaction 7).

Concluding from these unfavourable results, we opted for the thermally induced click reaction.

Synthesis of the Pyridine-SF$_4$-alkyne 3, General Procedure 1:

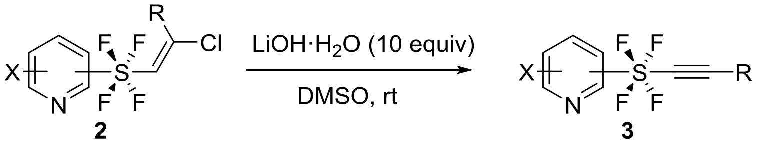

Prepared according to literature procedure.[1] Pyridine-SF$_4$-alkene **2** (1.0 equiv) was added to DMSO (0.3 M) in a round-bottom flask at room temperature, followed by Lithium hydroxide monohydrate (10.0 equiv) and allowed to stir at room temperature. The reaction progress was monitored by ^{19}F NMR, and after the ^{19}F NMR indicated the complete conversion to product, the reaction mixture was poured onto ice and extracted with Et_2O twice. The organic phase was dried with Na_2SO_4 and concentrated *in vacuo* to obtain the crude product. The crude product was purified by column chromatography on silica-gel eluting with *n*-Hexane/AcOEt mixture, to give the product **3a**–**k**.

5-Bromo-2-(tetrafluoro(phenylethynyl)- λ^6-sulfaneyl)pyridine (3a)

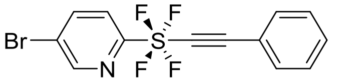

Prepared according to general procedure 1, by stirring **2a** (0.8 mmol) at rt for 24 h to obtain **3a** as white solid in 90% yield (262 mg). mp: 93–94 °C; HRMS (ESI$^+$): *m/z* calcd for $C_{13}H_8BrF_4NNaS$ [M+Na]$^+$: 387.9395 found: 387.9390. ^{1}H NMR (300 MHz, CDCl$_3$): δ = 7.49–7.36 (m, 3H), 7.60 (d, *J* = 6.6 Hz, 2H), 7.68 (d, *J* = 8.7 Hz, 1H), 8.00 (d, *J* = 8.4 Hz, 1H), 8.61 (d, *J* = 2.1 Hz, 1H), ^{19}F NMR (282 MHz, CDCl$_3$): δ = 77.61 (s, 4F), ^{13}C NMR (125 MHz, CDCl$_3$): δ = 74.23 (quint, *J* = 9.8 Hz), 93.88 (quint, *J* = 51.2 Hz, 1H), 118.70, 122.81 (quint, *J* = 3.8 Hz), 123.21, 128.69, 130.56, 132.69, 141.04, 148.58 (quint, *J* = 2.5 Hz), 167.85 (quint, *J* = 31.2 Hz). ATR-FTIR (KBr): *v* = 3115, 3049, 2916, 2222, 1446, 1368, 1092, 1007, 869, 700 cm^{-1}.

5-Chloro-2-(tetrafluoro(phenylethynyl)- λ^6-sulfaneyl)pyridine (3b)

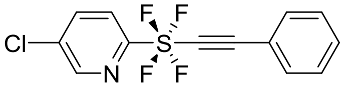

Prepared according to general procedure 1 by stirring **2b** (1.2 mmol) at rt for 24 h to obtain **3b** as white solid in 92% yield (354 mg). mp: 133–134 °C; HRMS (ESI$^+$): *m/z* calcd for $C_{13}H_8NF_4NaSCl$ [M+Na]$^+$: 343.9900 found: 343.9887. ^{1}H NMR (300 MHz, CDCl$_3$): δ = 7.36–7.48 (m, 3H), 7.59 (d, *J* = 6.9 Hz, 2H), 7.73 (d, *J* = 8.7 Hz, 1H), 7.84 (d, *J* = 8.7 Hz, 1H), 8.50 (d, *J* = 2.1 Hz, 1H), ^{19}F NMR (282 MHz, CDCl$_3$): δ = 77.70 (s, 4F), ^{13}C NMR (125 MHz, CDCl$_3$): δ = 74.19 (quint, *J* = 9.8 Hz), 93.90 (quint, *J* = 52.3 Hz), 118.67, 122.42 (quint, *J* = 4.3 Hz), 128.68, 130.55, 132.67, 134.49, 138.10, 146.31, 167.23 (quint, *J* = 30.6 Hz). ATR-FTIR (KBr): *v* = 3050, 2221, 1571, 1486, 1448, 1108, 779, 709 cm^{-1}.

5-Nitro-2-(tetrafluoro(phenylethynyl)- λ^6-sulfaneyl)pyridine (3c)

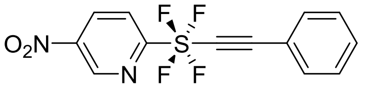

Prepared according to general procedure 1 by stirring **2c** (3.9 mmol) at rt for 24 h to obtain **3c** as brown solid in 50% yield (636 mg). mp: 142–143 °C; HRMS (EI$^+$): *m/z* calcd for $C_{13}H_8N_2O_2F_4S$ [M]$^+$: 332.0243 found: 332.0227. ^{1}H NMR (300 MHz, CDCl$_3$): δ = 7.38–7.50 (m, 3H), 7.61 (d, *J* = 6.9 Hz, 2H), 8.00 (d, *J* = 8.7 Hz, 1H), 8.67 (d, *J* = 8.7 Hz, 1H), 9.37 (d, *J* = 2.4 Hz, 1H), ^{19}F NMR (282 MHz, CDCl$_3$): δ = 77.45 (s, 4F), ^{13}C NMR (125 MHz, CDCl$_3$): δ = 75.16 (quint, *J* = 9.8 Hz), 93.18 (quint, *J* = 50.7 Hz), 118.34, 122.44 (quint, *J* = 3.8 Hz), 128.75, 130.80, 132.71, 133.92, 143.48, 144.98, 171.93 (quint, *J* = 31.3 Hz). ATR-FTIR (KBr): *v* = 3060, 2917, 2219, 1604, 1565, 1535, 1488, 1450, 1355, 809, 755, 690 cm^{-1}.

5-Methyl-2-(tetrafluoro(phenylethynyl)- λ⁶-sulfaneyl)pyridine (3d)

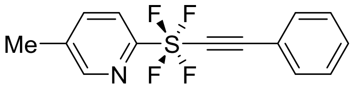

Prepared according to general procedure 1 by stirring **2d** (3.0 mmol) at rt for 24 h to obtain **3d** as light brown solid in 88% yield (799 mg). mp: 102–103 °C; HRMS (ESI$^+$): *m/z* calcd for $C_{14}H_{11}NF_4NaS$ $[M+Na]^+$: 324.0446 found: 324.0443. ^1H NMR (300 MHz, $CDCl_3$): δ = 2.40 (s, 3H), 7.34–7.46 (m, 3H), 7.58 (d, *J* = 6.6 Hz, 2H), 7.65 (s, 2H), 8.34 (s, 1H), ^{19}F NMR (282 MHz, $CDCl_3$): δ = 77.07 (s, 4F), ^{13}C NMR (125 MHz, $CDCl_3$): δ = 18.17, 73.55 (quint, *J* = 10.1 Hz), 94.39 (quint, *J* = 53.2 Hz), 118.89, 120.81 (quint, *J* = 5.0 Hz), 128.63, 130.39, 132.62, 136.63, 138.88, 147.57, 167.48 (quint, *J* = 28.8 Hz). ATR-FTIR (KBr): *v* = 3060, 2927, 2217, 1577, 1492, 1461, 1072, 777 cm^{-1}.

2-(tetrafluoro(phenylethynyl)- λ⁶-sulfaneyl)pyridine (3e)

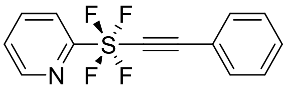

Prepared according to general procedure 1 by stirring **2e** (1.0 mmol) at rt for 24 h to obtain **3e** as light-yellow solid in 92% yield (264 mg). mp: 78–79 °C; HRMS (ESI$^+$): *m/z* calcd for $C_{13}H_9NF_4NaS$ $[M+Na]^+$: 310.0290 found: 310.0291. ^1H NMR (300 MHz, $CDCl_3$): δ = 7.35–7.47 (m, 4H), 7.57–7.60 (m, 2H), 7.76 (d, *J* = 8.1 Hz, 1H), 7.87 (t, *J* = 7.5 Hz, 1H), 8.55 (dd, *J* = 4.5 Hz, 1.5 Hz, 1H), ^{19}F NMR (282 MHz, $CDCl_3$): δ = 76.46 (s, 4F), ^{13}C NMR (125 MHz, $CDCl_3$): δ = 73.81 (quint, *J* = 10.0 Hz), 94.24 (quint, *J* = 52.5 Hz), 118.79, 121.40–121.47 (m), 126.36, 128.66, 130.47, 132.63, 138.68, 147.65, 169.51 (quint, *J* = 30.0 Hz). ATR-FTIR (KBr): *v* = 3060, 2221, 1579, 1490, 1457, 1099, 800, 759 cm^{-1}.

5-Bromo-2-(tetrafluoro((4-nitrophenyl)ethynyl)- λ⁶-sulfaneyl)pyridine (3f)

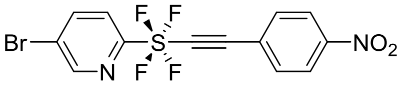

Prepared according to general procedure 1 by stirring **2f** (0.8 mmol) at rt for 24 h to obtain **3f** as white solid in 63% yield (206 mg). mp: 209–210 °C; HRMS (EI$^+$): *m/z* calcd for $C_{13}H_7N_2O_2F_4SBr$ $[M]^+$: 409.9348 found: 409.9355. ^1H NMR (300 MHz, $CDCl_3$): δ = 7.67 (d, *J* = 8.4 Hz, 1H), 7.78 (d, *J* = 8.7 Hz, 2H), 8.02 (d, *J* = 8.7 Hz, 1H), 8.27 (d, *J* = 8.7 Hz, 2H), 8.62 (d, *J* = 2.4 Hz, 1H), ^{19}F NMR (282 MHz, $CDCl_3$): δ = 77.08 (s, 4F), ^{13}C NMR (125 MHz, $CDCl_3$): δ = 71.48 (quint, *J* = 10.0 Hz), 97.19 (quint, *J* = 53.6 Hz), 122.73 (quint, *J* = 5.0 Hz), 123.54, 123.87, 125.50, 133.66, 141.18, 148.64, 148.75, 167.22 (quint, *J* = 29.3 Hz), ATR-FTIR (KBr): *v* = 3062, 2227, 1594, 1519, 1444, 1346, 1091, 765, 707 cm^{-1}.

5-Bromo-2-(tetrafluoro((4-methoxyphenyl)ethynyl)- λ⁶-sulfaneyl)pyridine (3g)

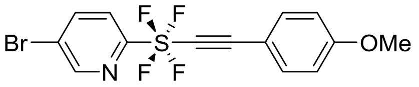

Prepared according to general procedure 1 by stirring **2g** (1 mmol) at rt for 24 h to obtain **3g** as white solid in 98% yield (387 mg). mp: 163–164 °C; HRMS (ESI$^+$): *m/z* calcd for $C_{14}H_{10}NOF_4NaSBr$ $[M+Na]^+$: 417.9500 found: 417.9501. ^1H NMR (300 MHz, $CDCl_3$): δ = 3.84 (s, 3H), 6.90 (dt, *J* = 9.0 Hz, 2.4 Hz, 2H), 7.53 (d, *J* = 8.7 Hz, 2H), 7.67 (d, *J* = 8.7 Hz, 1H), 7.99 (dd, *J* = 8.7 Hz, 0.9 Hz, 1H), 8.60 (d, *J* = 2.1 Hz 1H), ^{19}F NMR (282 MHz, $CDCl_3$): δ = 77.89 (s, 4F), ^{13}C NMR (125 MHz, $CDCl_3$): δ = 55.52, 74.81 (quint, *J* =9.8 Hz), 93.26 (quint, *J* = 51.3 Hz), 110.40, 114.35, 122.81 (quint, *J* = 5.0 Hz), 123.11, 134.37, 141.00, 148.53, 161.35, 168.05 (quint, *J* = 30.6 Hz). ATR-FTIR (KBr): *v* = 3046, 2219, 1606, 1511, 1446, 1243, 1093, 1031, 786, 678 cm^{-1}.

5-bromo-2-(((4-butylphenyl)ethynyl)tetrafluoro-λ^6-sulfaneyl)pyridine (3h)

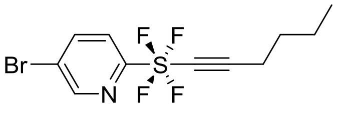

Prepared according to general procedure 1 by stirring **2h** (1.8 mmol) at rt for 24 h to obtain **3h** as white solid in 77% yield (480 mg). mp: 68–69 °C; HRMS (ESI$^+$): *m/z* calcd for $C_{11}H_{12}NF_4NaSBr$ [M+Na]$^+$: 367.9708 found: 367.9709. ^{1}H NMR (300 MHz, $CDCl_3$): δ = 0.95 (t, *J* = 7.2 Hz, 3H), 1.41–1.65 (m, 4H), 2.30–2.38 (m, 2H), 7.61 (d, *J* = 8.7 Hz, 1H), 7.96 (dt, *J* = 8.7 Hz, 0.9 Hz, 1H), 8.57 (d, *J* = 3.0 Hz, 1H), ^{19}F NMR (282 MHz, $CDCl_3$): δ = 77.61 (s, 4F), ^{13}C NMR (125 MHz, $CDCl_3$): δ = 13.64, 17.49, 22.02, 29.50, 76.45 (quint, *J* = 8.8 Hz), 85.97 (quint, *J* = 50.0 Hz), 122.76 (quint, *J* = 3.8 Hz), 123.00, 140.94, 148.46 (t, *J* = 2.5 Hz), 168.12 (quint, *J* = 31.3 Hz). ATR-FTIR (KBr): *v* = 3052, 2960, 2235, 1552, 1448, 1091, 794, 690 cm^{-1}.

5-bromo-2-(tetrafluoro((4-pentylphenyl)ethynyl)-λ^6-sulfaneyl)pyridine (3i)

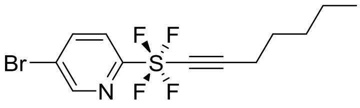

Prepared according to general procedure 1 by stirring **2i** (1.2 mmol) at rt for 24 h to obtain **3i** as white solid in 83% yield (359 mg). mp: 49–50 °C; HRMS (ESI$^+$): *m/z* calcd for $C_{12}H_{14}NF_4NaSBr$ [M+Na]$^+$: 381.9864 found: 381.9859. ^{1}H NMR (300 MHz, $CDCl_3$): δ = 0.92 (t, *J* = 7.2 Hz, 3H), 1.26–1.47 (m, 4H), 1.57–1.67 (m, 2H), 2.29–2.38 (m, 2H), 7.61 (d, *J* = 8.7 Hz, 1H), 7.96 (dt, *J* = 8.7 Hz, 1.2 Hz, 1H), 8.57 (d, *J* = 2.1 Hz, 1H), ^{19}F NMR (282 MHz, $CDCl_3$): δ = 77.65 (s, 4F), ^{13}C NMR (125 MHz, $CDCl_3$): δ = 14.02, 17.73, 22.20, 27.15, 31.02, 76.48 (quint, *J* = 9.8 Hz), 85.97 (quint, *J* = 51.0 Hz), 122.75 (quint, *J* = 4.4 Hz), 122.99, 140.94, 148.43 (t, *J* = 2.5 Hz), 168.11 (quint, *J* = 31.2 Hz). ATR-FTIR (KBr): *v* = 3052, 2954, 2235, 1567, 1452, 1091, 781, 701 cm^{-1}.

2-fluoro-3-(tetrafluoro(phenylethynyl)-λ^6-sulfaneyl)pyridine (3j)

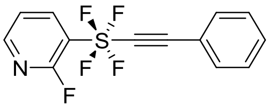

Prepared according to general procedure 1 by stirring **2j** (1.2 mmol) at rt for 8 h to obtain **3j** as light-yellow solid in 98% yield (334 mg). mp: 85–86 °C; HRMS (ESI$^+$): *m/z* calcd for $C_{13}H_9NFS$ [M+H]$^+$: 306.0376 found: 306.0382. ^{1}H NMR (300 MHz, $CDCl_3$): δ = 7.28–7.32 (m, 1H), 7.36–7.49 (m, 3H), 7.58 (d, *J* = 6.9 Hz, 2H), 8.23 (td, *J* = 8.1 Hz, 1.8 Hz, 1H), 8.33 (d, *J* = 4.5 Hz, 1H), ^{19}F NMR (282 MHz, $CDCl_3$): δ = –60.29– –59.95 (m, 1F), 91.65 (d, *J* = 22.6 Hz, 4F), ^{13}C NMR (125 MHz, $CDCl_3$): δ = 74.16 (quint, *J* = 8.8 Hz), 94.32 (quint, *J* = 51.3 Hz), 118.44, 121.63 (d, *J* = 5.0 Hz), 128.71, 130.66, 132.64, 139.72 (quint, *J* = 5.0 Hz), 140.32 (quint, *J* = 27.5 Hz), 149.92 (d, *J* = 15.0 Hz), 155.33 (d, *J* = 24.5 Hz) . ATR-FTIR (KBr): *v* = 3070, 2223, 1583, 1490, 1442, 1276, 1240, 1095, 788, 715 cm^{-1}.

2-fluoro-4-(tetrafluoro(phenylethynyl)-λ^6-sulfaneyl)pyridine (3k)

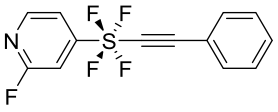

Prepared according to general procedure 1 by stirring **2k** (1.9 mmol) at rt for 24 h to obtain **3k** as light-yellow solid in 60% yield (350 mg). mp: 93–94 °C; HRMS (ESI$^+$): *m/z* calcd for $C_{13}H_9NF_5S$ [M+H]$^+$: 306.0376 found: 306.0373. ^{1}H NMR (300 MHz, $CDCl_3$): δ = 7.31 (t, *J* = 2.1 Hz, 1H), 7.37–7.50 (m, 3H), 7.54–7.60 (m, 3H), 8.36 (d, *J* = 5.4 Hz, 1H), ^{19}F NMR (282 MHz, $CDCl_3$): δ = –64.67 (s, 1F), 86.04 (s, 4F), ^{13}C NMR (125 MHz, $CDCl_3$): δ = 74.63 (quint, *J* = 10.0 Hz), 93.73 (quint, *J* = 51.3 Hz), 107.50–108.00 (m), 118.25–118.37 (m), 128.76, 130.80, 132.68, 148.49 (d, *J* = 15.0 Hz), 162.72, 164.64, 168.27–169.20 (m). ATR-FTIR (KBr): *v* = 3029, 2227, 1596, 1575, 1477, 1444, 1228, 1101, 788, 750 cm^{-1}.

Synthesis of benzene-tetrafluoro(phenylethynyl)-λ^6-sulfane (6)

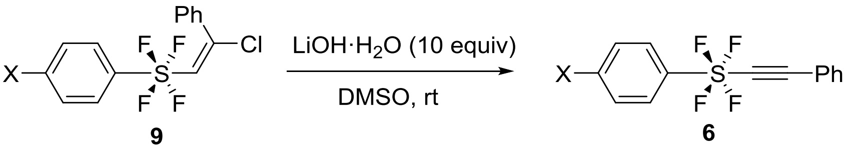

Alkyne **6b** and **6c** were prepared according to literature procedure.[5] Alkyne **6a** was synthesized using a modified literature procedure.[5]

(4-bromophenyl)tetrafluoro(phenylethynyl)-λ^6-sulfane (6a)

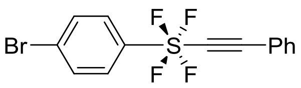

Benzene-SF_4-alkene **9a** (3.0 mmol) was added to DMSO (0.3 M) in a round-bottom flask at room temperature, followed by Lithium hydroxide monohydrate (10.0 equiv) and allowed to stir at room temperature. The reaction progress was monitored by ^{19}F NMR, and after the ^{19}F NMR indicated the complete conversion to product, the reaction mixture was poured onto ice and extracted with Et_2O twice. The organic phase was dried with Na_2SO_4 and concentrated *in vacuo* to obtain the crude product. The crude product was purified by washing with hexane to obtain **6a** as a yellow solid in 30% yield. mp: 69–72 °C; HRMS (ESI$^+$): *m/z* calcd for $C_{14}H_8F_4SBr$ [M–H]$^+$: 362.9466 found: 362.9491. ^{1}H NMR (300 MHz, $CDCl_3$): δ = 7.38–7.44 (m, 3H), 7.53–7.67 (m, 6H), ^{19}F NMR (282 MHz, $CDCl_3$): δ = 88.48 (s, 4F), ^{13}C NMR (125 MHz, $CDCl_3$): δ = 73.06 (quint, *J* = 10.0 Hz), 95.03 (quint, *J* = 53.6 Hz, 1H), 118.78, 125.05, 127.68 (quint, *J* = 5.0 Hz), 128.67, 130.44, 131.58, 132.61, 158.17 (quint, *J* = 23.6 Hz). ATR-FTIR (KBr): *v* = 3111, 2925, 2225, 1574, 1475, 1070, 752, 683 cm^{-1}.

(Synthesis of the Pyridine/Benzene-SF_4-triazole 5 or 7, General Procedure 2:

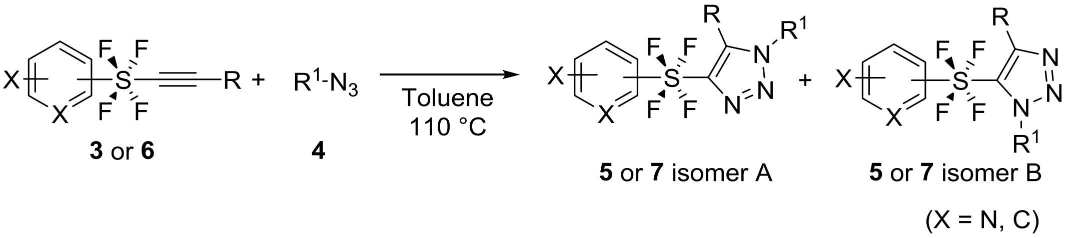

An oven dried test tube was charged with alkyne **3** or **6** (0.5 mmol), azide **4** (1.5 mmol) and toluene (2.5 mL) and allowed to stir at 110 °C for 24 h or unless mentioned otherwise. The reaction was allowed to cool to room temperature and the solvent was evaporated *in vacuo* to give the crude products. The ratio of the two regioisomers was calculated from the crude ^{19}F NMR. The products were isolated using column chromatography on silica-gel, eluting with *n*-Hexane/AcOEt mixture, to get pure regioisomers of **5**, A and B. The total isolated yield of the reaction was calculated by adding the weight of pure A, B and the inseparable mixture of A, B obtained after column chromatography.

2-((1-benzyl-5-phenyl-1H-1,2,3-triazol-4-yl)tetrafluoro-λ^6-sulfaneyl)-5-bromopyridine (5a-A)

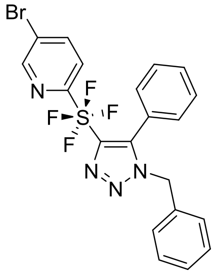

Prepared according to general procedure 2, by stirring at 110 °C for 24 h and separated from its regiosiomer by column chromatography (*n*-Hexane/AcOEt, 4/1) to isolate pure **5a**-A as white solid. mp: 138–139 °C; HRMS (ESI$^+$): *m/z* calcd for $C_{20}H_{15}N_4F_4NaSBr$ [M+Na]$^+$: 521.0035 found: 521.0029. ^{1}H NMR (300 MHz, $CDCl_3$): δ = 5.30 (s, 2H), 6.94–6.97 (m, 2H), 7.15 (d, *J* = 7.2 Hz, 2H), 7.20–7.25 (m, 3H), 7.38 (t, *J* = 6.9 Hz, 2H), 7.46 (t, *J* = 7.2 Hz, 1H), 7.64 (d, *J* = 8.7 Hz, 1H), 7.92 (d, *J* = 8.1 Hz, 1H), 8.50 (d, *J* = 2.1 Hz, 1H), ^{19}F NMR (282 MHz, $CDCl_3$): δ = 61.31 (s, 4F), ^{13}C NMR (125 MHz, $CDCl_3$): δ = 53.06, 122.81, 122.96 (quint, *J* = 3.8 Hz), 126.27, 127.96, 128.57, 128.60, 128.83, 130.13, 130.24, 133.69–137.03 (m), 134.19, 140.86, 148.28, 159.29 (quint, *J* = 32.5 Hz), 168.21 (quint, *J* = 30 Hz). ATR-FTIR (KBr): *v* = 3048, 1560, 1496, 1479, 1446, 1361, 1074, 794, 696 cm^{-1}.

2-((1-benzyl-4-phenyl-1H-1,2,3-triazol-5-yl)tetrafluoro-λ^6-sulfaneyl)-5-bromopyridine (5a-B)

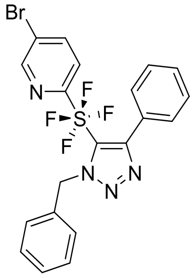

Prepared according to general procedure 2, by stirring at 110 °C for 24 h and separated from its regiosiomer by column chromatography (Hexane/AcOEt, 4/1) to isolate pure **5b**-B as white solid. mp: 155–156 °C; HRMS (ESI$^+$): *m/z* calcd for $C_{20}H_{15}N_4F_4NaSBr$ [M+Na]$^+$: 521.0035 found: 521.0040. ^{1}H NMR (300 MHz, $CDCl_3$): δ = 5.94 (s, 2H), 7.30–7.35 (m, 5H), 7.39–7.41 (m, 3H), 7.56–7.59 (m, 2H), 7.63 (d, *J* = 8.1 Hz, 1H), 7.80 (t, *J* = 7.8 Hz, 1H), 8.48 (d, *J* = 3.3 Hz, 1H), ^{19}F NMR (282 MHz, $CDCl_3$): δ = 67.66 (s, 4F), ^{13}C NMR (125 MHz, $CDCl_3$): δ = 55.77, 122.59–122.69 (m), 123.52, 127.75, 128.02, 128.39, 128.84, 128.94, 130.21, 131.14, 135.11, 141.13, 144.39, 148.08–148.90 (m), 148.61, 167.38–167.85 (m). ATR-FTIR (KBr): *v* = 3062, 1560, 1498, 1446, 1369, 1093, 786, 696 cm^{-1}.

Total isolated yield of **5a** is 77% (192 mg).

2-((1-benzyl-5-phenyl-1H-1,2,3-triazol-4-yl)tetrafluoro-λ^6-sulfaneyl)-5-chloropyridine (5b-A)

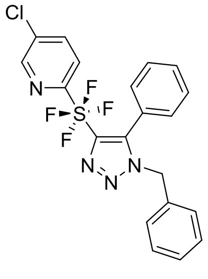

Prepared according to general procedure 2, by stirring at 110 °C for 24 h and separated from its regiosiomer by column chromatography (*n*-Hexane/AcOEt, 7/3) to isolate pure **5b**-A as white solid. mp: 141–142 °C; HRMS (ESI$^+$): *m/z* calcd for $C_{20}H_{15}N_4F_4NaSCl$ [M+Na]$^+$: 477.0540 found: 477.0566. ^{1}H NMR (300 MHz, $CDCl_3$): δ = 5.31 (s, 2H), 6.96 (dd, *J* = 1.5 Hz, 7.2 Hz, 2H), 7.15 (d, *J* = 7.2 Hz , 2H), 7.21–7.30 (m, 3H), 7.36–7.42 (m, 2H), 7.45–7.50 (m, 1H), 7.70–7.79 (m, 2H), 8.42 (d, *J* = 2.4 Hz, 1H), ^{19}F NMR (282 MHz, $CDCl_3$): δ = 61.39 (s, 4F), ^{13}C NMR (125 MHz, $CDCl_3$): δ = 53.10, 122.61 (quint, *J* = 3.8 Hz), 126.33, 128.00, 128.60, 128.64, 128.87, 130.16, 130.27, 134.01 (quint, *J* = 5.0 Hz), 134.13, 134.22, 137.95, 146.07, 159.35 (quint, *J* = 33.0 Hz), 167.66 (quint, *J* = 30.0 Hz), ATR-FTIR (KBr): *v* = 3066, 3031, 1562, 1482, 1454, 1126, 1108, 777, 700 cm^{-1}.

2-((1-benzyl-4-phenyl-1H-1,2,3-triazol-5-yl)tetrafluoro-λ^6-sulfaneyl)-5-chloropyridine (5b-B)

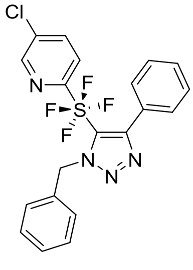

Prepared according to general procedure 2, by stirring at 110 °C for 24 h and separated from its regiosiomer by column chromatography (*n*-Hexane/AcOEt, 7/3) to isolate pure **5b**-B as white solid. mp: 140–141 °C; HRMS (ESI$^+$): *m/z* calcd for $C_{20}H_{15}N_4F_4NaSCl$ [M+Na]$^+$: 477.0540 found: 477.0536. ^{1}H NMR (300 MHz, $CDCl_3$): δ = 5.93 (s, 2H), 7.30–7.37 (m, 5H), 7.40–7.44 (m, 3H), 7.54–7.57 (m, 2H), 7.61 (d, *J* = 9.0 Hz , 1H), 7.79 (d, *J* = 8.7 Hz , 1H), 8.45 (d, *J* = 2.4 Hz, 1H), ^{19}F NMR (282 MHz,

$CDCl_3$): δ = 67.78 (s, 4F), ^{13}C NMR (125 MHz, $CDCl_3$): δ = 55.75, 122.25–122.31 (m), 127.72, 128.00, 128.37, 128.82, 128.92, 130.20, 131.15, 134.79, 135.11, 138.18, 144.36, 146.32, 148.64 (quint, J = 35.0 Hz), 166.98 (quint, J = 29.0 Hz), ATR-FTIR (KBr): v = 3054, 2967, 1567, 1477, 1450, 1124, 1074, 781 cm^{-1}.

Total isolated yield of **5b** is 86% (195 mg).

2-((1-benzyl-5-phenyl-1H-1,2,3-triazol-4-yl)tetrafluoro-λ^6-sulfaneyl)-5-nitropyridine (5c-A)

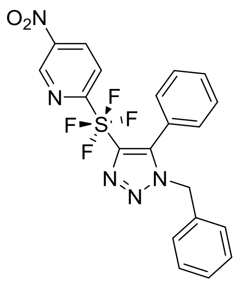

Prepared according to general procedure 2, by stirring at 110 °C for 24 h and separated from its regiosiomer by column chromatography (Hexane/AcOEt, 4/1) to isolate pure **5c**-A as light brown solid. mp: 136–137 °C; HRMS (ESI$^+$): m/z calcd for $C_{20}H_{15}N_5O_2F_4NaS$ $[M+Na]^+$: 488.0780 found: 488.0781. ^1H NMR (300 MHz, $CDCl_3$): δ = 5.32 (s, 2H), 6.97 (dd, J = 7.2 Hz, 1.5 Hz, 2H), 7.16 (d, J = 7.2 Hz, 2H), 7.22–7.31 (m, 3H), 7.38–7.52 (m, 3H), 7.97 (d, J = 9.0 Hz, 1H), 8.59 (dd, J = 9.0 Hz, 2.4 Hz, 1H), 9.27 (d, J = 2.7 Hz, 1H), ^{19}F NMR (282 MHz, $CDCl_3$): δ = 61.23 (s, 4F), ^{13}C NMR (125 MHz, $CDCl_3$): δ = 52.14, 122.89–122.96 (m), 123.01, 124.18, 125.91, 128.87, 128.94, 130.08, 130.58, 134.28 (quint, J = 3.8 Hz), 140.97, 141.00, 148.10, 148.43, 159.18–159.71 (m), 167.83–168.31 (m). ATR-FTIR (KBr): v = 3050, 1606, 1567, 1535, 1482, 1455, 1359, 1076, 779, 763 cm^{-1}.

2-((1-benzyl-4-phenyl-1H-1,2,3-triazol-5-yl)tetrafluoro-λ^6-sulfaneyl)-5-nitropyridine (5c-B)

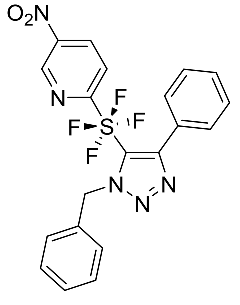

Prepared according to general procedure 2, by stirring at 110 °C for 24 h and separated from its regiosiomer by column chromatography (Hexane/AcOEt, 4/1) to isolate pure **5c**-B as light brown solid. mp: 149–150 °C; HRMS (ESI$^+$): m/z calcd for $C_{20}H_{15}N_5O_2F_4NaS$ $[M+Na]^+$: 488.0780 found: 488.0794. ^1H NMR (300 MHz, $CDCl_3$): δ = 5.93 (s, 2H), 7.33–7.39 (m, 5H), 7.40–7.45 (m, 3H), 7.54–7.57 (m, 2H), 7.87 (d, J = 9.0 Hz, 1H), 8.60 (dd, J = 8.7 Hz, 2.1 Hz, 1H), 9.30 (d, J = 2.4 Hz, 1H), ^{19}F NMR (282 MHz, $CDCl_3$): δ = 67.49 (s, 4F), ^{13}C NMR (125 MHz, $CDCl_3$): δ =54.91, 122.55, 123.75, 124.18, 128.13, 128.41, 129.18, 130.14, 130.69, 141.25, 142.18, 144.75, 147.97, 148.48–149.04 (m), 148.69, 167.16–167.61 (m). ATR-FTIR (KBr): v = 3035, 1602, 1569, 1533, 1494, 1473, 1448, 1357, 1074, 806, 763 cm^{-1}.

Total isolated yield of **5c** is 92% (214 mg).

2-((1-benzyl-5-phenyl-1H-1,2,3-triazol-4-yl)tetrafluoro-λ^6-sulfaneyl)-5-methylpyridine (5d-A)

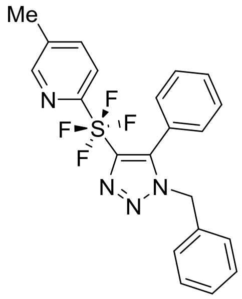

Prepared according to general procedure 2, by stirring at 110 °C for 24 h and separated from its regiosiomer by column chromatography (Hexane/AcOEt, 7/3) to isolate pure **1d**-A as white solid. mp: 130–131 °C; HRMS (ESI$^+$): m/z calcd for $C_{21}H_{18}N_4F_4NaS$ $[M+Na]^+$: 457.1086 found: 457.1109. ^1H NMR (300 MHz, $CDCl_3$): δ = 2.33 (s, 3H), 5.30 (s, 2H), 6.96 (dd, J = 7.8 Hz, 2.0 Hz, 2H), 7.15 (d, J = 7.2 Hz, 2H), 7.20–7.27 (m, 3H), 7.34–7.47 (m, 3H), 7.56–7.65 (m, 2H), 8.26 (s, 1H), ^{19}F NMR (282 MHz, $CDCl_3$): δ = 60.62 (s, 4F), ^{13}C NMR (125 MHz, $CDCl_3$): δ = 17.10, 52.05, 119.93–120.03 (m), 125.52, 126.98, 127.54, 127.57, 127.83, 129.03, 129.33, 132.86–132.92 (m), 133.31, 135.22, 137.72, 146.37, 158.78 (quint, J = 32.5 Hz), 166.95 (quint, J = 28.8 Hz). ATR-FTIR (KBr): v = 3056, 2964, 2933, 1577, 1461, 1376, 1074, 769, 732 cm^{-1}.

2-((1-benzyl-4-phenyl-1H-1,2,3-triazol-5-yl)tetrafluoro-λ⁶-sulfaneyl)-5-methylpyridine (5d-B)

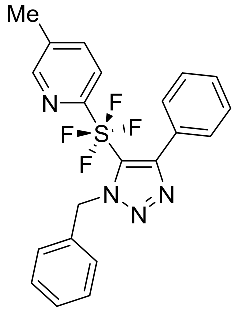

Prepared according to general procedure 2, by stirring at 110 °C for 24 h and separated from its regiosiomer by column chromatography (Hexane/AcOEt, 7/3) to isolate pure **5d**-B as white solid. mp: 118–119 °C; HRMS (ESI$^+$): *m/z* calcd for $C_{21}H_{18}N_4F_4NaS$ $[M+Na]^+$: 457.1086 found: 457.1082. ^1H NMR (300 MHz, $CDCl_3$): δ = 2.37 (s, 3H), 5.94 (s, 2H), 7.34–7.36 (m, 5H), 7.39–7.41 (m, 3H), 7.52–7.62 (m, 4H), 8.30 (s, 1H), ^{19}F NMR (282 MHz, $CDCl_3$): δ = 67.04 (s, 4F), ^{13}C NMR (125 MHz, $CDCl_3$): δ = 18.17, 55.69, 120.69–120.75 (m), 127.77, 127.95, 128.28, 128.78, 128.81, 130.25, 131.36, 135.27, 136.97, 138.93, 144.21, 147.60, 149.12 (quint, *J* = 36.3 Hz), 167.13–167.57 (m). ATR-FTIR (KBr): *v* = 3033, 2958, 2925, 1573, 1459, 1376, 1076, 781, 725 cm^{-1}.

Total isolated yield of **5d** is 60% (130 mg).

2-((1-benzyl-5-phenyl-1H-1,2,3-triazol-4-yl)tetrafluoro-λ⁶-sulfaneyl)pyridine (5e-A)

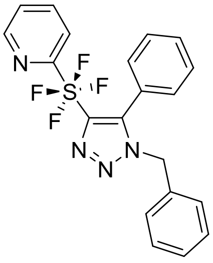

Prepared according to general procedure 2, by stirring at 110 °C for 24 h and separated from its regiosiomer by column chromatography (*n*-Hexane/AcOEt, 7/3) to isolate pure **5e**-A as light brown solid. mp: 141–142 °C; HRMS (ESI$^+$): *m/z* calcd for $C_{20}H_{16}N_4F_4NaS$ $[M+Na]^+$: 443.0929 found: 443.0929. ^1H NMR (300 MHz, $CDCl_3$): δ = 5.30 (s, 2H), 6.94–6.97 (m, 2H), 7.16 (d, *J* = 7.2 Hz, 2H), 7.20–7.29 (m, 3H), 7.32–7.47 (m, 4H), 7.72–7.81 (m, 2H), 8.45 (d, *J* = 4.2 Hz, 1H), ^{19}F NMR (282 MHz, $CDCl_3$): δ = 60.06 (s, 4F), ^{13}C NMR (125 MHz, $CDCl_3$): δ = 52.97, 121.43–121.54 (m), 125.59, 126.35, 127.90, 128.49, 128.50, 128.76, 130.01, 130.22, 133.89 (quint, *J* = 5.0 Hz), 134.20, 138.47, 147.35, 159.53 (quint, *J* = 33.8 Hz), 169.84 (quint, *J* = 28.8 Hz). ATR-FTIR (KBr): *v* = 3035, 1577, 1479, 1457, 1361, 1076, 773 cm^{-1}.

2-((1-benzyl-4-phenyl-1H-1,2,3-triazol-5-yl)tetrafluoro-λ⁶-sulfaneyl)pyridine (5e-B):

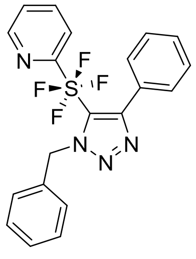

Prepared according to general procedure 2, by stirring at 110 °C for 24 h and separated from its regiosiomer by column chromatography (*n*-Hexane/AcOEt, 7/3) to isolate pure **5e**-B as light brown solid. mp: 131–132 °C; HRMS (ESI$^+$): *m/z* calcd for $C_{20}H_{16}N_4F_4NaS$ $[M+Na]^+$: 443.0929 found: 443.0921. ^1H NMR (300 MHz, $CDCl_3$): δ = 5.94 (s, 2H), 7.30–7.35 (m, 5H), 7.39–7.41 (m, 4H), 7.56–7.59 (m, 2H), 7.63 (d, *J* = 8.1 Hz, 1H), 7.80 (t, *J* = 7.8 Hz, 1H), 8.49 (d, *J* = 4.5 Hz, 1H), ^{19}F NMR (282 MHz, $CDCl_3$): δ = 66.39 (s, 4F), ^{13}C NMR (125 MHz, $CDCl_3$): δ = 55.66, 121.23–121.29 (m), 126.56, 127.70, 127.93, 128.26, 128.75, 128.81, 130.19, 131.26, 135.18, 138.76, 144.23, 147.62, 148.91 (quint, *J* = 35.0 Hz), 169.26 (quint, *J* = 27.5 Hz). ATR-FTIR (KBr): *v* = 3035, 1581, 1496, 1461, 1361, 1324, 1076, 759 cm^{-1}.

Total isolated yield of **5e** is 71% (149 mg).

2-((1-benzyl-5-(4-nitrophenyl)-1H-1,2,3-triazol-4-yl)tetrafluoro-λ^6-sulfaneyl)-5-bromopyridine (5f-A)

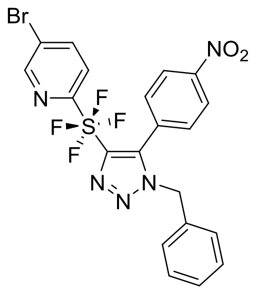

Prepared according to general procedure 2, by stirring at 110 °C for 24 h and separated from its regiosiomer by column chromatography (*n*-Hexane/AcOEt, 3/1) to isolate pure **5f**-A as white solid. mp: 176–179 °C; HRMS (ESI$^+$): *m/z* calcd for $C_{20}H_{14}N_5O_2F_4NaSBr$ [M+Na]$^+$: 565.9885 found: 565.9886. ^{1}H NMR (300 MHz, $CDCl_3$): δ = 5.36 (s, 2H), 6.93 (d, *J* = 6.6 Hz, 2H), 7.23–7.32 (m, 5H), 7.64 (d, *J* = 8.7 Hz, 1H), 7.95 (d, *J* = 8.7 Hz, 1H), 8.23 (td, *J* = 8.7 Hz, 2.4 Hz, 2H), 8.52 (d, *J* = 2.4 Hz, 1H), ^{19}F NMR (282 MHz, $CDCl_3$): δ = 61.72 (s, 4F), ^{13}C NMR (125 MHz, $CDCl_3$): δ = 53.68, 122.85 (quint, *J* = 5.0 Hz), 123.16, 123.66, 127.75, 129.08, 129.18, 131.63, 131.79 (quint, *J* = 3.8 Hz), 133.06, 133.68, 141.05, 148.48, 148.90, 159.79 (quint, *J* = 34.0 Hz), 167.89 (quint, *J* = 31.0 Hz), ATR-FTIR (KBr): *v* = 3056, 1602, 1527, 1448, 1344, 1093, 767, 692 cm^{-1}.

2-((1-benzyl-4-(4-nitrophenyl)-1H-1,2,3-triazol-5-yl)tetrafluoro-λ^6-sulfaneyl)-5-bromopyridine (5f-B)

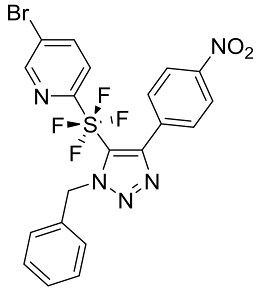

Prepared according to general procedure 2, by stirring at 110 °C for 24 h and separated from its regiosiomer by column chromatography (*n*-Hexane/AcOEt, 3/1) to isolate pure **5f**-B as white solid. mp: 179–180 °C; HRMS (ESI$^+$): *m/z* calcd for $C_{20}H_{14}N_5O_2F_4NaSBr$ [M+Na]$^+$: 565.9885 found: 565.9881. ^{1}H NMR (300 MHz, $CDCl_3$): δ = 5.94 (s, 2H), 7.36 (brs, 5H), 7.56 (d, *J* = 8.7 Hz, 1H), 7.77 (d, *J* = 8.7 Hz, 2H), 7.98 (d, *J* = 8.1 Hz, 1H), 8.28 (d, *J* = 8.7 Hz, 2H), 8.57, (d, *J* = 2.1 Hz, 1H), ^{19}F NMR (282 MHz, $CDCl_3$): δ = 67.96 (s, 4F), ^{13}C NMR (125 MHz, $CDCl_3$): δ = 56.03, 122.48, 123.28, 123.85, 127.85, 128.63, 128.94, 131.34, 134.63, 137.68, 141.31, 142.21, 148.23, 148.77, 148.98 (quint, *J* = 39.0 Hz), 167.28 (quint, *J* = 29.0 Hz), ATR-FTIR (KBr): *v* = 3056, 1602, 1513, 1450, 1349, 1099, 779, 692 cm^{-1}.

Total isolated yield of **5f** is 67% (182 mg).

2-((1-benzyl-5-(4-methoxyphenyl)-1H-1,2,3-triazol-4-yl)tetrafluoro-λ^6-sulfaneyl)-5-bromopyridine (5g-A)

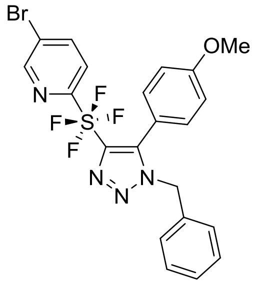

Prepared according to general procedure 2, by stirring at 110 °C for 24 h and separated from its regiosiomer by column chromatography (*n*-Hexane/AcOEt, 7/3) to isolate pure **5g**-A as white solid. mp: 147–148 °C; HRMS (ESI$^+$): *m/z* calcd for $C_{21}H_{17}N_4OF_4NaSBr$ [M+Na]$^+$: 551.0140 found: 551.0139. ^{1}H NMR (300 MHz, $CDCl_3$): δ = 3.83 (s, 3H), 5.82 (s, 2H), 6.90 (d, *J* = 9.0 Hz, 2H), 6.99–7.02 (m, 2H), 7.07 (d, *J* = 8.7 Hz, 2H), 7.24–7.28 (m, 3H), 7.65 (d, *J* = 8.7 Hz, 1H), 7.92 (d, *J* = 8.7 Hz, 1H), 8.52 (d, *J* = 2.1 Hz, 1H), ^{19}F NMR (282 MHz, $CDCl_3$): δ = 61.19 (s, 4F), ^{13}C NMR (125 MHz, $CDCl_3$): δ = 52.92, 55.42, 114.10, 117.97, 122.79, 122.99 (quint, *J* = 5.0 Hz), 127.95, 128.58, 128.85, 131.61, 133.96 (t, *J* = 3.8 Hz), 134.39, 140.86, 148.29, 159.45 (quint, *J* = 32.4 Hz), 160.86, 168.31 (quint, *J* = 31.2 Hz), ATR-FTIR (KBr): *v* = 3045, 2956, 1562, 1494, 1255, 1091, 1000, 769, 686 cm^{-1}.

2-((1-benzyl-4-(4-methoxyphenyl)-1H-1,2,3-triazol-5-yl)tetrafluoro-λ^6-sulfaneyl)-5-bromopyridine (5g-B)

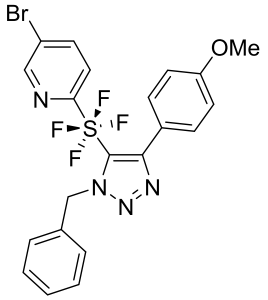

Prepared according to general procedure 2, by stirring at 110 °C for 24 h and separated from its regiosiomer by column chromatography (*n*-Hexane/AcOEt, 7/3) to isolate pure **5g**-B as white solid. mp: 149–150 °C; HRMS (ESI$^+$): *m/z* calcd for $C_{21}H_{17}N_4OF_4NaSBr$ [M+Na]$^+$: 551.0140 found: 551.0151. ^{1}H NMR (300 MHz, CDCl$_3$): δ = 3.83 (s, 3H), 5.91 (s, 2H), 6.94 (d, *J* = 9.0 Hz, 2H), 7.29–7.34 (m, 5H), 7.48–7.57 (m, 3H), 7.95 (d, *J* = 9.0 Hz, 1H), 8.55 (d, *J* = 2.1 Hz, 1H), ^{19}F NMR (282 MHz, CDCl$_3$): δ = 67.40 (s, 4F), ^{13}C NMR (125 MHz, CDCl$_3$): δ = 55.35, 55.75, 113.48, 122.65, 123.41, 123.47, 127.70, 128.33, 128.81, 131.43, 135.18, 141.12, 144.14, 148.46 (quint, *J* = 35.0 Hz), 148.58, 160.06, 167.68 (quint, *J* = 29.5 Hz), ATR-FTIR (KBr): *v* = 3064, 2940, 1552, 1486, 1448, 1249, 1095, 1031, 784, 682 cm^{-1}.

Total isolated yield of **5g** is 70% (185 mg).

2-((1-benzyl-5-butyl-1H-1,2,3-triazol-4-yl)tetrafluoro-λ^6-sulfaneyl)-5-bromopyridine (5h-A)

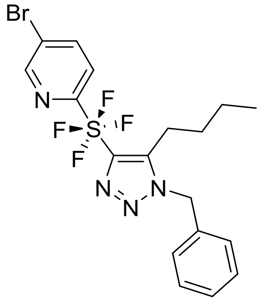

Prepared according to general procedure 2, by stirring at 110 °C for 24 h and separated from its regiosiomer by column chromatography (*n*-Hexane/AcOEt, 4/1) to isolate pure **5h**-A as white solid. mp: 127–128 °C; HRMS (ESI$^+$): *m/z* calcd for $C_{18}H_{19}N_4F_4NaSBr$ [M+Na]$^+$: 501.0348 found: 501.0349. ^{1}H NMR (300 MHz, CDCl$_3$): δ = 0.84 (t, *J* = 6.9 Hz, 3H), 1.26–1.35 (m, 4H), 2.76–2.81 (m, 2H), 5.54 (s, 2H), 7.22–7.26 (m, 2H), 7.23–7.36 (m, 3H), 7.78 (d, *J* = 8.7 Hz, 1H), 8.02 (d, *J* = 8.7 Hz, 1H), 8.62 (d, *J* = 2.1 Hz, 1H), ^{19}F NMR (282 MHz, CDCl$_3$): δ = 59.61 (s, 4F), ^{13}C NMR (125 MHz, CDCl$_3$): δ = 13.64, 22.89, 23.42, 30.39, 52.82, 122.91, 123.05 (quint, 5.0 Hz), 127.35, 128.75, 129.19, 134.23–134.29, 141.03, 148.34, 159.00 (quint, *J* = 32.8 Hz), 168.56 (quint, *J* = 31.5 Hz). ATR-FTIR (KBr): *v* = 3031, 2958, 1563, 1448, 1093, 775, 690 cm^{-1}.

2-((1-benzyl-4-butyl-1H-1,2,3-triazol-5-yl)tetrafluoro-λ^6-sulfaneyl)-5-bromopyridine (5h-B)

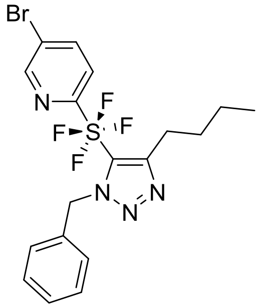

Prepared according to general procedure 2, by stirring at 110 °C for 24 h and separated from its regiosiomer by column chromatography (*n*-Hexane/AcOEt, 4/1) to isolate pure **5h**-B as white solid. mp: 67–68 °C; HRMS (ESI$^+$): *m/z* calcd for $C_{18}H_{19}N_4F_4NaSBr$ [M+Na]$^+$: 501.0348 found: 501.0360. ^{1}H NMR (300 MHz, CDCl$_3$): δ = 0.95 (t, *J* = 7.0 Hz, 3H), 1.37–1.50 (m, 2H), 1.74–1.84 (m, 2H), 2.92 (t, *J* = 7.2 Hz, 2H), 5.82 (s, 2H), 7.22–7.25 (m, 2H), 7.28–7.35 (m, 3H), 7.69 (d, *J* = 8.7 Hz, 1H), 8.03 (d, *J* = 8.7 Hz, 1H), 8.62 (d, *J* = 2.1 Hz, 1H), ^{19}F NMR (282 MHz, CDCl$_3$): δ = 64.98 (s, 4F), ^{13}C NMR (125 MHz, CDCl$_3$): δ = 14.01, 22.76, 26.35, 31.09, 55.16, 122.66 (t, *J* = 3.8 Hz), 123.51, 127.49, 128.15, 128.71, 135.39, 141.23, 144.96, 148.52 (quint, *J* = 34.5 Hz), 148.64, 167.97 (quint, *J* = 29.9 Hz), ATR-FTIR (KBr): *v* = 3064, 2960, 1552, 1452, 1091, 769, 694 cm^{-1}.

Total isolated yield of **5h** is 68% (163 mg).

2-((1-benzyl-5-pentyl-1H-1,2,3-triazol-4-yl)tetrafluoro-λ^6-sulfaneyl)-5-bromopyridine (5i-A)

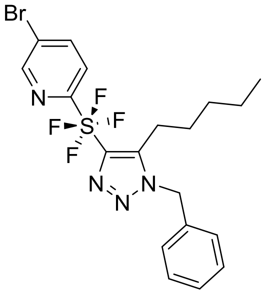

Prepared according to general procedure 2, by stirring at 110 °C for 24 h and separated from its regiosiomer by column chromatography (*n*-Hexane/AcOEt, 4/1) to isolate pure **5i**-A as white solid. mp: 109–110 °C; HRMS (ESI$^+$): *m/z* calcd for $C_{19}H_{21}N_4F_4NaSBr$ [M+Na]$^+$: 515.0504 found: 515.0516. ^{1}H NMR (300 MHz, $CDCl_3$): δ = 0.83 (t, *J* = 6.9 Hz, 3H), 1.19–1.31 (m, 6H), 2.78 (t, *J* = 7.5 Hz, 2H), 5.54 (s, 2H), 7.22–7.26 (m, 2H), 7.32–7.41 (m, 3H), 7.78 (d, *J* = 8.7 Hz, 1H), 8.02 (d, *J* = 8.7 Hz, 1H), 8.62 (d, *J* = 2.1 Hz, 1H), ^{19}F NMR (282 MHz, $CDCl_3$): δ = 59.57 (s, 4F), ^{13}C NMR (125 MHz, $CDCl_3$): δ = 13.97, 22.29, 23.73, 28.15, 31.95, 52.89, 122.95, 123.10 (quint, *J* = 5.0 Hz), 127.39, 128.83, 129.26, 134.27, 134.29–134.35 (m), 141.07, 148.42, 159.03 (quint, *J* = 32.4 Hz), 168.61 (quint, *J* = 31.6 Hz). ATR-FTIR (KBr): *v* = 3064, 2935, 1554, 1452, 1093, 765, 692 cm^{-1}.

2-((1-benzyl-4-pentyl-1H-1,2,3-triazol-5-yl)tetrafluoro-λ^6-sulfaneyl)-5-bromopyridine (5i-B)

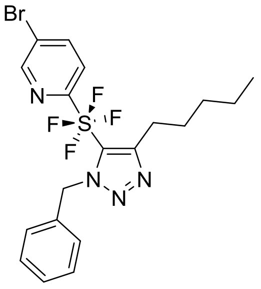

Prepared according to general procedure 2, by stirring at 110 °C for 24 h and separated from its regiosiomer by column chromatography (*n*-Hexane/AcOEt, 4/1) to isolate pure **5i**-B as white solid. mp: 72–73 °C; HRMS (ESI$^+$): *m/z* calcd for $C_{19}H_{21}N_4F_4NaSBr$ [M+Na]$^+$: 515.0504 found: 515.0515. ^{1}H NMR (300 MHz, $CDCl_3$): δ = 0.90 (t, *J* = 6.9 Hz, 3H), 1.30–1.44 (m, 4H), 1.76–1.84 (m, 2H), 2.91 (t, *J* = 7.8 Hz, 2H), 5.82 (s, 2H), 7.22–7.25 (m, 2H), 7.28–7.35 (m, 3H), 7.68 (d, *J* = 8.7 Hz, 1H), 8.03 (d, *J* = 9.0 Hz, 1H), 8.62 (d, *J* = 2.1 Hz, 1H), ^{19}F NMR (282 MHz, $CDCl_3$): δ = 65.00 (s, 4F), ^{13}C NMR (125 MHz, $CDCl_3$): δ = 14.14, 22.58, 26.60, 28.68, 31.84, 55.17, 122.66, 123.51, 127.49, 128.16, 128.71, 135.39, 141.23, 144.99, 148.50 (quint, *J* = 34.4 Hz), 148.64, 167.98 (quint, *J* = 30.2 Hz). ATR-FTIR (KBr): *v* = 3062, 2933, 1565, 1494, 1450, 1093, 779, 690 cm^{-1}.

Total isolated yield of **5i** is 70% (173 mg).

3-((1-benzyl-5-phenyl-1H-1,2,3-triazol-4-yl)tetrafluoro-λ^6-sulfaneyl)-2-fluoropyridine (5j-A)

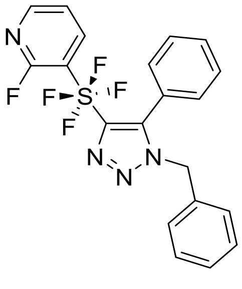

Prepared according to general procedure 2, by stirring at 110 °C for 24 h and separated from its regiosiomer by column chromatography (Hexane/AcOEt, 7/3) to isolate pure **5j**-A as white solid. mp: 152–153 °C; HRMS (ESI$^+$): *m/z* calcd for $C_{20}H_{15}N_4F_5NaS$ [M+Na]$^+$: 461.0835 found: 461.0837. ^{1}H NMR (300 MHz, $CDCl_3$): δ = 5.32 (s, 2H), 6.96 (dd, *J* = 7.8 Hz, 1.5 Hz, 2H), 7.15 (d, *J* = 7.2 Hz, 2H), 7.20–7.31 (m, 4H), 7.38–7.43 (m, 2H), 7.46–7.52 (m 1H), 8.17 (td, *J* = 8.1 Hz, 1.8 Hz, 1H), 8.25 (d, *J* = 4.8 Hz, 1H), ^{19}F NMR (282 MHz, $CDCl_3$): δ = –60.15 (quintd, *J* = 22.6 Hz, 8.5 Hz, 1F), 75.11 (d, *J* = 22.6 Hz, 4F), ^{13}C NMR (125 MHz, $CDCl_3$): δ = 53.11, 121.47 (d, *J* = 5.0 Hz), 126.12, 127.96, 128.59, 128.65, 128.87, 130.23 (d, *J* = 3.8 Hz), 133.92–133.99 (m), 134.14, 139.69–139.81 (m), 140.31–141.42 (m), 149.56 (d, *J* = 15.0 Hz), 154.30, 156.26, 159.64 (quint, *J* = 31.3 Hz). ATR-FTIR (KBr): *v* = 3031, 2929, 1587, 1573, 1496, 1438, 1280, 1232, 1076, 777, 694 cm^{-1}.

3-((1-benzyl-4-phenyl-1H-1,2,3-triazol-5-yl)tetrafluoro-λ^6-sulfaneyl)-2-fluoropyridine (5j-B)

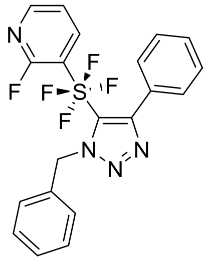

Prepared according to general procedure 2, by stirring at 110 °C for 24 h and separated from its regiosiomer by column chromatography (Hexane/AcOEt, 7/3) to isolate pure **5j**-B as white solid. mp: 157–158 °C; HRMS (ESI$^+$): *m/z* calcd for $C_{20}H_{15}N_4F_5NaS$ [M+Na]$^+$: 461.0835 found: 461.0835. ^{1}H NMR (300 MHz, $CDCl_3$): δ = 5.92 (s, 2H), 7.23–7.27 (m, 1H), 7.32–7.38 (m, 5H), 7.42–7.45 (m, 3H), 7.53–7.56 (m, 2H), 8.08 (td, *J* = 8.1 Hz, 1.5 Hz, 1H), 8.31 (d, *J* = 4.8 Hz, 1H), ^{19}F NMR (282 MHz, $CDCl_3$): δ = –60.08 (quintd, *J* = 22.6 Hz, 8.46 Hz, 1F), 81.46 (d, *J* = 22.6 Hz, 4F), ^{13}C NMR (125 MHz, $CDCl_3$): δ = 55.88, 121.70 (d, *J* = 5.0 Hz), 127.84, 128.06, 128.50, 128.87, 129.04, 130.17, 130.97, 134.92, 139.51, 140.05–140.68 (m), 144.29, 148.62–149.17 (m), 150.25 (d, *J* = 15.0 Hz), 155.09 (d, *J* = 245 Hz). ATR-FTIR (KBr): *v* = 3035, 2923, 1590, 1438, 1284, 1228, 1093, 813, 730 cm^{-1}.

Total isolated yield of **5j** is 95% (208 mg).

4-((1-benzyl-5-phenyl-1H-1,2,3-triazol-4-yl)tetrafluoro-λ^6-sulfaneyl)-2-fluoropyridine (5k-A)

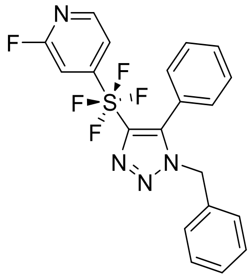

Prepared according to general procedure 2, by stirring at 110 °C for 24 h and separated from its regiosiomer by column chromatography (*n*-Hexane/AcOEt, 4/1) to isolate pure **5k**-A as white solid. mp: 131–132 °C; HRMS (ESI$^+$): *m/z* calcd for $C_{20}H_{15}N_4F_5NaS$ [M+Na]$^+$: 461.0835 found: 461.0829. ^{1}H NMR (300 MHz, $CDCl_3$): δ = 5.32 (s, 2H), 6.96 (dd, *J* = 1.2 Hz, 6.9 Hz, 2H), 7.14 (d, *J* = 7.5 Hz, 2H), 7.24–7.31 (m, 4H), 7.40–7.53 (m, 4H), 8.27 (d, *J* = 5.7 Hz, 1H), ^{19}F NMR (282 MHz, $CDCl_3$): δ = –65.11 (s, 1F), 69.64 (s, 4F), ^{13}C NMR (125 MHz, $CDCl_3$): δ = 53.19, 107.76 (quintd, *J* = 42.5 Hz, 5.0 Hz), 118.42 (sextet), 125.05, 128.01, 128.67, 128.72, 128.91, 130.19, 130.34, 133.94–134.01 (m), 134.06, 148.24 (d, *J* = 13.8 Hz), 159.29 (quint, *J* = 32.5 Hz), 162.60, 164.52, 169.19 (dquint, *J* = 27.5 Hz, 7.5 Hz). ATR-FTIR (KBr): *v* = 3064, 1592, 1475, 1448, 1234, 1105, 786, 701 cm^{-1}.

4-((1-benzyl-4-phenyl-1H-1,2,3-triazol-5-yl)tetrafluoro-λ^6-sulfaneyl)-2-fluoropyridine (5k-B)

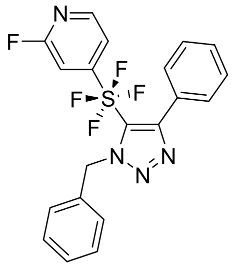

Prepared according to general procedure 2, by stirring at 110 °C for 24 h and separated from its regiosiomer by column chromatography (*n*-Hexane/AcOEt, 4/1) to isolate pure **5k**-B as white solid. mp: 131–132 °C; HRMS (ESI$^+$): *m/z* calcd for $C_{20}H_{15}N_4F_5NaS$ [M+Na]$^+$: 461.0835 found: 461.0822. ^{1}H NMR (300 MHz, $CDCl_3$): δ = 5.90 (s, 2H), 7.21–7.22 (m, 1H), 7.31–7.46 (m, 9H), 7.51–7.55 (m, 2H), 8.32 (d, *J* = 5.7 Hz, 1H), ^{19}F NMR (282 MHz, $CDCl_3$): δ = –64.12 (s, 1F), 76.07 (s, 4F), ^{13}C NMR (125 MHz, $CDCl_3$): δ = 55.88, 107.61 (dt, *J* = 41.3 Hz, 5.0 Hz), 118.00–118.11 (m), 127.58, 128.11, 128.54, 128.93, 129.14, 130.10, 130.82, 134.88, 144.48, 148.57 (quint, *J* = 33.6 Hz), 148.65 (d, *J* = 15.0 Hz), 162.66, 164.58, 168.47 (dquint, *J* = 26.3 Hz, 7.5 Hz). ATR-FTIR (KBr): *v* = 3035, 1589, 1575, 1473, 1230, 1101, 782, 696 cm^{-1}.

Total isolated yield of **5k** is 91% (199 mg).

5-bromo-2-(tetrafluoro(1-(4-nitrobenzyl)-5-phenyl-1H-1,2,3-triazol-4-yl)-λ^6-sulfaneyl)pyridine (5l-A)

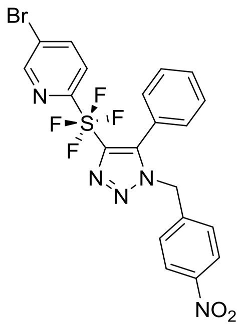

Prepared according to general procedure 2, by stirring at 110 °C for 24 h and separated from its regiosiomer by column chromatography (*n*-Hexane/AcOEt, 7/3) to isolate pure **5l**-A as yellow solid. mp: 153–154 °C; HRMS (ESI$^+$): *m/z* calcd for $C_{20}H_{14}N_5O_2F_4NaSBr$ [M+Na]$^+$: 565.9885 found: 565.9889. ^{1}H NMR (300 MHz, $CDCl_3$): δ = 5.42 (s, 2H), 7.15 (d, *J* = 8.7 Hz, 4H), 7.42 (t, *J* = 7.2 Hz, 2H), 7.51 (t, *J* = 7.5 Hz, 1H), 7.66 (d, *J* = 8.7 Hz, 1H), 7.95 (d, *J* = 8.7 Hz, 1H), 8.13 (d, *J* = 8.7 Hz, 2H), 8.53 (d, *J* = 2.4 Hz, 1H), ^{19}F NMR (282 MHz, $CDCl_3$): δ = 61.33 (s, 4F), ^{13}C NMR (125 MHz, $CDCl_3$): δ = 52.14, 122.89–123.00 (m), 124.18, 125.91, 128.87, 128.94, 130.08, 130.58, 134.28 (quint, *J* = 3.8 Hz), 140.97, 141.00, 148.10, 148.43, 159.18–159.71, 167.83–168.31. ATR-FTIR (KBr): *v* = 3060, 1604, 1565, 1521, 1481, 1452, 1415, 1348, 1095, 786, 696 cm^{-1}.

5-bromo-2-(tetrafluoro(1-(4-nitrobenzyl)-4-phenyl-1H-1,2,3-triazol-5-yl)-λ^6-sulfaneyl)pyridine (5l-B)

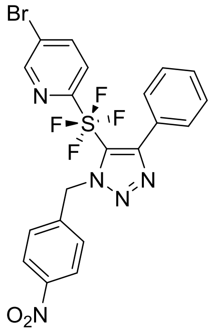

Prepared according to general procedure 2, by stirring at 110 °C for 24 h and separated from its regiosiomer by column chromatography (*n*-Hexane/AcOEt, 7/3) to isolate pure **5l**-B as white solid. mp: 186–187 °C; HRMS (ESI$^+$): *m/z* calcd for $C_{20}H_{14}N_5O_2F_4NaSBr$ [M+Na]$^+$: 565.9885 found: 565.9869. ^{1}H NMR (300 MHz, $CDCl_3$): δ = 6.03 (s, 2H), 7.41–7.58 (m, 8H), 7.96 (d, *J* = 8.4 Hz, 1H), 8.24 (dt, *J* = 8.7 Hz, 1.8 Hz, 2H), 8.55 (d, *J* = 2.4 Hz, 1H), ^{19}F NMR (282 MHz, $CDCl_3$): δ = 67.62 (s, 4F), ^{13}C NMR (125 MHz, $CDCl_3$): δ = 54.91, 122.55, 123.75, 1242.18, 128.13, 128.41, 129.18, 130.14, 130.69, 141.25, 142.18, 144.75, 147.75, 147.97, 148.69, 167.39 (quint, *J* = 28.8 Hz). ATR-FTIR (KBr): *v* = 3077, 1600, 1517, 1479, 1444, 1417, 1344, 1097, 765, 692 cm^{-1}.

Total isolated yield of **5l** is 92% (250 mg).

5-bromo-2-((1-(4-bromobenzyl)-5-phenyl-1H-1,2,3-triazol-4-yl)tetrafluoro-λ^6-sulfaneyl)pyridine (5m-A)

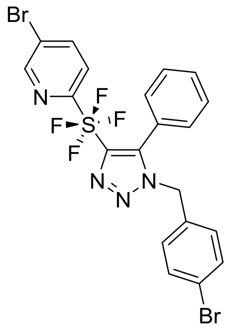

Prepared according to general procedure 2, by stirring at 110 °C for 24 h and separated from its regiosiomer by column chromatography (*n*-Hexane/AcOEt, 7/3) to isolate pure **5m**-A as white solid. mp: 132–133 °C; HRMS (ESI$^+$): *m/z* calcd for $C_{20}H_{14}N_4F_4NaSBr_2$ [M+Na]$^+$: 598.9140 found: 598.9132. ^{1}H NMR (300 MHz, $CDCl_3$): δ = 5.26 (s, 2H), 6.84 (d, *J* = 8.4 Hz, 2H), 7.16 (d, *J* = 7.2 Hz, 2H), 7.36–7.51 (m, 5H), 7.65 (d, *J* = 8.7 Hz, 1H), 7.93 (d, *J* = 8.7 Hz, 1H), 8.51 (d, *J* = 2.1 Hz, 1H), ^{19}F NMR (282 MHz, $CDCl_3$): δ = 61.29 (s, 4F), ^{13}C NMR (125 MHz, $CDCl_3$): δ = 52.38, 122.84, 122.86, 122.90–122.97 (m), 126.11, 128.70, 129.70, 130.17, 130.28, 132.01, 133.12, 133.92–134.02 (m), 140.89, 148.30, 159.30 (quint, *J* = 32.5 Hz), 168.13 (quint, *J* = 30 Hz). ATR-FTIR (KBr): *v* = 3056, 1596, 1558, 1486, 1359, 1095, 782, 698 cm^{-1}.

5-bromo-2-((1-(4-bromobenzyl)-4-phenyl-1H-1,2,3-triazol-5-yl)tetrafluoro-λ⁶-sulfaneyl)pyridine (5m-B)

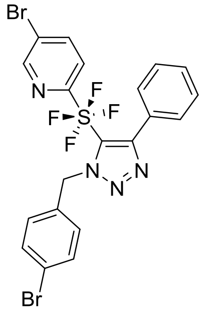

Prepared according to general procedure 2, by stirring at 110 °C for 24 h and separated from its regiosiomer by column chromatography (*n*-Hexane/AcOEt, 7/3) to isolate pure **5m**-B as white solid. mp: 150–151 °C; HRMS (ESI$^+$): *m/z* calcd for $C_{20}H_{14}N_4F_4NaSBr_2$ [M+Na]$^+$: 598.9140 found: 598.9147. ^1H NMR (300 MHz, $CDCl_3$): δ = 5.87 (s, 2H), 7.23 (d, *J* = 8.4 Hz, 2H), 7.41–7.44 (m, 3H), 7.47–7.52 (m, 2H), 7.53–7.55 (m, 3H), 7.95 (d, *J* = 8.7 Hz, 1H), 8.56 (d, *J* = 2.4 Hz, 1H), ^{19}F NMR (282 MHz, $CDCl_3$): δ = 67.62 (s, 4F), ^{13}C NMR (125 MHz, $CDCl_3$): δ = 55.15, 122.59, 123.61, 128.05, 129.03, 129.54, 130.18, 130.97, 132.03, 134.08, 141.18, 144.50, 148.28–148.84 (m), 148.65, 167.30–167.76 (m). ATR-FTIR (KBr): *v* = 3048, 1554, 1486, 1446, 1359, 1095, 771, 692 cm^{-1}.

Total isolated yield of **5m** is 63% (182 mg).

5-bromo-2-(tetrafluoro(1-(3-fluorobenzyl)-5-phenyl-1H-1,2,3-triazol-4-yl)-λ⁶-sulfaneyl)pyridine (5n-A)

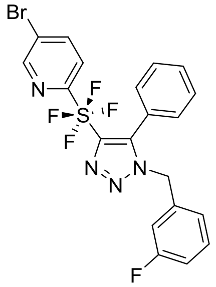

Prepared according to general procedure 2, by stirring at 110 °C for 24 h and separated from its regiosiomer by column chromatography (Hexane/AcOEt, 4/1) to isolate pure **5n**-A as white solid. mp: 134–135 °C; HRMS (ESI$^+$): *m/z* calcd for $C_{20}H_{14}N_4F_5NaSBr$ [M+Na]$^+$: 538.9940 found: 538.9933. ^1H NMR (300 MHz, $CDCl_3$): δ = 5.30 (s, 2H), 6.66 (d, *J* = 9.3 Hz, 1H), 6.76 (d, *J* = 7.8 Hz, 1H), 6.99 (td, *J* = 8.4 Hz, 2.4 Hz, 1H), 7.16 (d, *J* = 7.2 Hz, 2H), 7.19–7.25 (m, 1H), 7.38–7.52 (m, 3H), 7.66 (d, *J* = 8.7 Hz, 1H), 7.94 (d, *J* = 8.7 Hz, 1H), 8.53 (d, *J* = 2.4 Hz, 1H), ^{19}F NMR (282 MHz, $CDCl_3$): δ = –112.47– –112.38 (m, 1F), 61.29 (s, 4F), ^{13}C NMR (125 MHz, $CDCl_3$): δ = 52.48 (d, *J* = 1.3 Hz), 115.11 (d, *J* = 22.5 Hz), 115.76 (d, *J* = 21.3 Hz), 122.90, 122.95–123.06 (m), 123.63 (d, *J* = 2.5 Hz), 126.14, 128.73, 130.19, 130.35, 130.57 (d, *J* = 7.5 Hz), 134.05–134.15 (m), 136.46 (d, *J* = 7.5 Hz), 140.91, 148.37, 159.35 (quint, *J* = 32.5 Hz), 162.83 (d, *J* = 246.3 Hz), 168.19 (quint, *J* = 31.3 Hz). ATR-FTIR (KBr): *v* = 3077, 1616, 1590, 1556, 1486, 1450, 1361, 1253, 1093, 765, 696 cm^{-1}.

5-bromo-2-(tetrafluoro(1-(3-fluorobenzyl)-4-phenyl-1H-1,2,3-triazol-5-yl)-λ⁶-sulfaneyl)pyridine (5n-B)

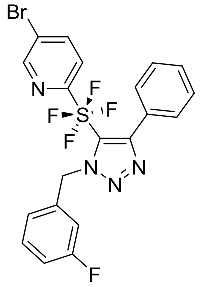

Prepared according to general procedure 2, by stirring at 110 °C for 24 h and separated from its regiosiomer by column chromatography (Hexane/AcOEt, 4/1) to isolate pure **5n**-B as white solid. mp: 148–149 °C; HRMS (ESI$^+$): *m/z* calcd for $C_{20}H_{14}N_4F_5NaSBr$ [M+Na]$^+$: 538.9940 found: 538.9955. ^1H NMR (300 MHz, $CDCl_3$): δ = 5.91 (s, 2H), 7.04 (d, *J* = 9.3 Hz, 2H), 7.11 (d, *J* = 7.8 Hz, 1H), 7.30–7.37 (m, 1H), 7.41–7.44 (m, 3H), 7.53–7.58 (m, 3H), 7.95 (d, *J* = 8.7 Hz, 1H), 8.56 (d, *J* = 2.1 Hz, 1H), ^{19}F NMR (282 MHz, $CDCl_3$): δ = –112.83– –112.75 (m, 1F), 67.61 (s, 4F), ^{13}C NMR (125 MHz, $CDCl_3$): δ = 55.14, 114.79 (d, *J* = 21.3 Hz), 115.43 (d, *J* = 21.3 Hz), 122.59–122.65 (m), 123.28 (d, *J* = 2.5 Hz), 123.60, 128.05, 129.03, 130.19, 130.46 (d, *J* = 8.75 Hz), 130.97, 137.49 (d, *J* = 7.5 Hz), 141.18, 144.50, 148.09–149.20 (m), 148.64, 162.99 (d, *J* = 245.0 Hz), 167.52 (quint, *J* = 28.8 Hz). ATR-FTIR (KBr): *v* = 3052, 1590, 1486, 1448, 1365, 1261, 1097, 798, 692 cm^{-1}.

Total isolated yield of **5n** is 78% (212 mg).

4-((4-((5-bromopyridin-2-yl)tetrafluoro-λ⁶-sulfaneyl)-5-phenyl-1H-1,2,3-triazol-1-yl)methyl)benzonitrile (5o-A**)**

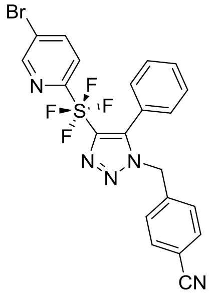

Prepared according to general procedure 2, by stirring at 110 °C for 24 h and separated from its regiosiomer by column chromatography (Hexane/AcOEt, 7/3) to isolate pure **5o**-A as white solid. mp: 160–161 °C; HRMS (ESI⁺): *m/z* calcd for $C_{21}H_{14}N_5F_4NaSBr$ $[M+Na]^+$: 545.9987 found: 545.9988. ^1H NMR (300 MHz, $CDCl_3$): δ = 5.37 (s, 2H), 7.08 (d, *J* = 8.4 Hz, 2H), 7.14 (d, *J* = 7.2 Hz, 2H), 7.39–7.44 (m, 2H), 7.47–7.52 (m, 1H), 7.55–7.58 (m, 2H), 7.65 (d, *J* = 8.7 Hz, 1H), 7.95 (d, *J* = 9.0 Hz, 1H), 8.53 (d, *J* = 2.1 Hz, 1H), ^{19}F NMR (282 MHz, $CDCl_3$): δ = 61.33 (s, 4F), ^{13}C NMR (125 MHz, $CDCl_3$): δ = 52.41, 112.77, 118.25, 122.87–122.97 (m), 125.92, 128.62, 128.86, 130.06, 130.49, 132.73, 134.16–134.26 (m), 139.17, 140.95, 148.38, 159.40 (quint, *J* = 33.8 Hz), 186.06 (quint, *J* = 31.25 Hz). ATR-FTIR (KBr): *v* = 3064, 2227, 1610, 1565, 1508, 1482, 1450, 1357, 1093, 794, 700 cm^{-1}.

4-((5-((5-bromopyridin-2-yl)tetrafluoro-λ⁶-sulfaneyl)-4-phenyl-1H-1,2,3-triazol-1-yl)methyl)benzonitrile (5o-B**)**

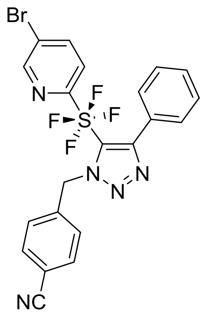

Prepared according to general procedure 2, by stirring at 110 °C for 24 h and separated from its regiosiomer by column chromatography (Hexane/AcOEt, 7/3) to isolate pure **5o**-B as white solid. mp: 154–155 °C; HRMS (ESI⁺): *m/z* calcd for $C_{21}H_{14}N_5F_4NaSBr$ $[M+Na]^+$: 545.9987 found: 545.9990. ^1H NMR (300 MHz, $CDCl_3$): δ = 5.98 (s, 2H), 7.39–7.45 (m, 5H), 7.51–7.57 (m, 3H), 7.66 (d, *J* = 8.4 Hz, 2H), 7.96 (d, *J* = 9.0 Hz, 1H), 8.55 (d, *J* = 2.4 Hz, 1H), ^{19}F NMR (282 MHz, $CDCl_3$): δ = 67.61 (s, 4F), ^{13}C NMR (125 MHz, $CDCl_3$): δ = 55.09, 112.40, 118.50, 122.53, 123.70, 128.09, 128.20, 129.13, 130.10, 130.70, 132.71, 140.28, 141.23, 144.64, 148.64, 148.72 (quint, *J* = 35.0 Hz), 167.37 (quint, *J* = 28.8 Hz). ATR-FTIR (KBr): *v* = 3056, 2229, 1610, 1567, 1506, 1477, 1450, 1359, 1093, 777, 694 cm^{-1}.

Total isolated yield of **5o** is 75% (197 mg).

5-bromo-2-(tetrafluoro(1-(4-methoxybenzyl)-5-phenyl-1H-1,2,3-triazol-4-yl)-λ⁶-sulfaneyl)pyridine (5p-A**)**

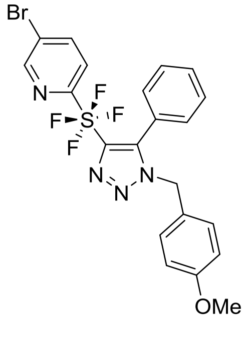

Prepared according to general procedure 2, by stirring at 110 °C for 24 h and separated from its regiosiomer by column chromatography (*n*-Hexane/AcOEt, 7/3) to isolate pure **5p**-A as white solid. mp: 156–157 °C; HRMS (ESI⁺): *m/z* calcd for $C_{21}H_{17}N_4OF_4NaSBr$ $[M+Na]^+$: 551.0140 found: 551.0162. ^1H NMR (300 MHz, $CDCl_3$): δ = 3.77 (s, 3H), 5.24 (s, 2H), 6.73–6.78 (m, 2H), 6.87–6.91 (m, 2H), 7.16 (d, *J* = 6.9 Hz, 2H), 7.38–7.51 (m, 3H), 7.65 (d, *J* = 8.7 Hz, 1H), 7.92 (dd, *J* = 8.7 Hz, 1.2 Hz, 1H), 8.52 (d, *J* = 2.4 Hz, 1H), ^{19}F NMR (282 MHz, $CDCl_3$): δ = 61.32 (s, 4F), ^{13}C NMR (125 MHz, $CDCl_3$): δ = 52.66, 55.39, 114.15, 122.82, 122.29 (quint, *J* = 3.8 Hz), 126.23, 126.43, 128.59, 129.59, 130.13, 130.33, 133.77 (quint, *J* = 5.0 Hz), 140.86, 148.31, 159.30 (quint, *J* = 32.5 Hz), 159.78, 168.26 (quint, *J* = 31.3 Hz). ATR-FTIR (KBr): *v* = 3041, 1616, 1589, 1560, 1513, 1446, 1357,1245, 1089, 1035, 808, 755 cm^{-1}.

5-bromo-2-(tetrafluoro(1-(4-methoxybenzyl)-4-phenyl-1H-1,2,3-triazol-5-yl)-λ^6-sulfaneyl)pyridine (5p-B)

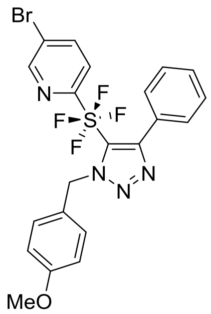

Prepared according to general procedure 2, by stirring at 110 °C for 24 h and separated from its regiosiomer by column chromatography (*n*-Hexane/AcOEt, 7/3) to isolate pure **5p**-B as white solid. mp: 155–156 °C; HRMS (ESI$^+$): *m/z* calcd for $C_{21}H_{17}N_4OF_4NaSBr$ $[M+Na]^+$: 551.0140 found: 551.0146. ^{1}H NMR (300 MHz, $CDCl_3$): δ = 3.80 (s, 3H), 5.85 (s, 2H), 6.86–6.91 (m, 2H), 7.34 (d, *J* = 8.7 Hz, 2H), 7.39–7.42 (m, 3H), 7.52–7.57 (m, 3H), 7.95 (d, *J* = 8.7 Hz, 1H), 8.56 (d, *J* = 2.1 Hz, 1H), ^{19}F NMR (282 MHz, $CDCl_3$): δ = 67.76 (s, 4F), ^{13}C NMR (125 MHz, $CDCl_3$): δ = 55.40, 114.15, 122.65, 123.49, 127.02, 127.98, 128.88, 129.59, 130.20, 131.20, 141.13, 144.28, 148.10–148.38 (m), 148.59, 159.69, 167.66 (quint, *J* = 28.8 Hz). ATR-FTIR (KBr): *v* = 3066, 1610, 1513, 1454, 1361, 1251, 1097, 1025, 800, 757 cm^{-1}.

Total isolated yield of **5p** is 68% (180 mg).

5-bromo-2-(tetrafluoro(1-(3-methylbenzyl)-5-phenyl-1H-1,2,3-triazol-4-yl)-λ^6-sulfaneyl)pyridine (5q-A)

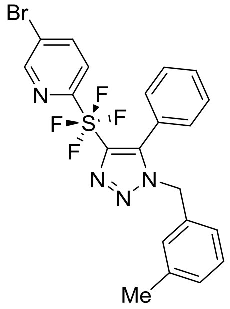

Prepared according to general procedure 2, by stirring at 110 °C for 24 h and separated from its regiosiomer by column chromatography (*n*-Hexane/AcOEt, 7/3) to isolate pure **5q**-A as white solid. mp: 108–109 °C; HRMS (ESI$^+$): *m/z* calcd for $C_{21}H_{17}N_4F_4NaSBr$ $[M+Na]^+$: 535.0191 found: 535.0189. ^{1}H NMR (300 MHz, $CDCl_3$): δ = 2.25 (s, 3H), 5.27 (s, 2H), 6.72–6.76 (m, 2H), 7.06–7.16 (m, 4H), 7.37–7.42 (m, 2H), 7.45–7.50 (m, 1H), 7.65 (d, *J* = 8.7 Hz, 1H), 7.92 (dd, *J* = 8.7 Hz, 0.9 Hz, 1H), 8.52 (d, *J* = 2.1 Hz, 1H), ^{19}F NMR (282 MHz, $CDCl_3$): δ = 61.32 (s, 4F), ^{13}C NMR (125 MHz, $CDCl_3$): δ = 21.38, 53.17, 122.83, 122.99 (quint, *J* = 5.0 Hz), 125.10, 126.37, 128.54, 128.71, 128.88, 129.38, 130.11, 130.33, 133.89–133.96 (m), 134.00, 138.61, 140.87, 148.32, 159.30 (quint, *J* = 32.5 Hz), 168.26 (quint, *J* = 30.0 Hz). ATR-FTIR (KBr): *v* = 3056, 1610, 1554, 1482, 1444, 1093, 782, 694 cm^{-1}.

5-bromo-2-(tetrafluoro(1-(3-methylbenzyl)-4-phenyl-1H-1,2,3-triazol-5-yl)-λ^6-sulfaneyl)pyridine (5q-B)

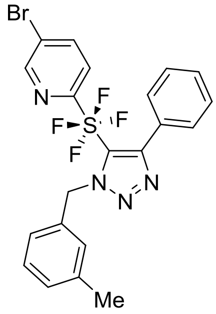

Prepared according to general procedure 2, by stirring at 110 °C for 24 h and separated from its regiosiomer by column chromatography (*n*-Hexane/AcOEt, 7/3) to isolate pure **5q**-B as white solid. mp: 104–105 °C; HRMS (ESI$^+$): *m/z* calcd for $C_{21}H_{17}N_4F_4NaSBr$ $[M+Na]^+$: 535.0191 found: 535.0203. ^{1}H NMR (300 MHz, $CDCl_3$): δ = 2.34 (s, 3H), 5.88 (s, 2H), 7.11–7.16 (m, 3H), 7.21–7.28 (m, 1H), 7.39–7.41 (m, 3H), 7.52–7.57 (m, 3H), 7.92 (d, *J* = 8.7 Hz, 1H), 8.54 (d, *J* = 2.1 Hz, 1H), ^{19}F NMR (282 MHz, $CDCl_3$): δ = 67.69 (s, 4F), ^{13}C NMR (125 MHz, $CDCl_3$): δ = 21.54, 55.71, 122.60–122.65 (m), 123.47, 124.80, 127.97, 128.37, 128.67, 128.89, 129.14, 130.19, 131.15, 134.97, 138.53, 141.11, 144.29, 148.03–148.86 (m), 148.54, 167.59 (quint, *J* = 30.0 Hz). ATR-FTIR (KBr): *v* = 3048, 1606, 1562, 1479, 1446, 1091, 786, 690 cm^{-1}.

Total isolated yield of **5q** is 67% (172 mg).

5-bromo-2-((1,5-diphenyl-1H-1,2,3-triazol-4-yl)tetrafluoro-λ^6-sulfaneyl)pyridine (5r-A)

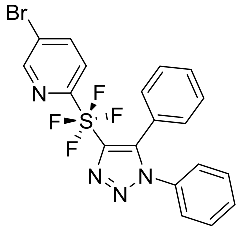

Prepared according to general procedure 2, by stirring at 110 °C for 48 h and isolated by column chromatography (*n*-Hexane/AcOEt, 7/3) to get only **5r**-A as brown solid in 26% yield (63.4 mg). mp: 177–178 °C; HRMS (ESI$^+$): *m/z* calcd for $C_{19}H_{13}N_4F_4NaSBr$ [M+Na]$^+$: 506.9878 found: 506.9891. ^{1}H NMR (300 MHz, $CDCl_3$): δ = 7.28–7.38 (m, 10H), 7.71 (d, *J* = 8.7 Hz, 1H), 7.97 (d, *J* = 8.7 Hz, 1H), 8.57 (d, *J* = 2.4 Hz, 1H), ^{19}F NMR (282 MHz, $CDCl_3$): δ = 61.70 (s, 4F), ^{13}C NMR (125 MHz, $CDCl_3$): δ = 122.96, 123.06 (quint, *J* = 5.0 Hz), 125.51, 126.57, 128.57, 129.34, 129.72, 129.97, 130.57, 134.28, 136.12, 140.96, 148.44, 159.18–159.71 (m), 168.03–168.51 (m). ATR-FTIR (KBr): *v* = 3056, 1592, 1565, 1494, 1477, 1446, 1357, 1089, 782, 698 cm^{-1}.

5-bromo-2-(tetrafluoro(1-(4-nitrophenyl)-5-phenyl-1H-1,2,3-triazol-4-yl)-λ^6-sulfaneyl)pyridine (5s-A)

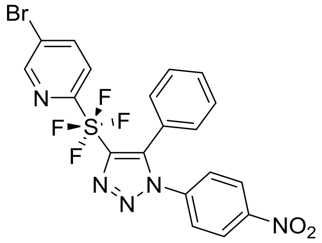

Prepared according to general procedure 2, by stirring at 110 °C for 48 h and isolated by column chromatography (*n*-Hexane/AcOEt, 4/1) to get only **5s**-A as light brown solid in 66% yield (174 mg). mp: 162–163 °C; HRMS (ESI$^+$): *m/z* calcd for $C_{19}H_{12}N_5O_2F_4NaSBr$ [M+Na]$^+$: 551.9729 found: 551.9714. ^{1}H NMR (300 MHz, $CDCl_3$): δ = 7.35 (d, *J* = 7.5 Hz, 2H), 7.39–7.52 (m, 5H), 7.70 (d, *J* = 8.7 Hz, 1H), 7.99 (d, *J* = 8.7 Hz, 1H), 8.22 (dt, *J* = 9.0 Hz, 2.7 Hz, 2H), 8.56 (d, *J* = 2.4 Hz, 1H), ^{19}F NMR (282 MHz, $CDCl_3$): δ = 61.89 (s, 4F), ^{13}C NMR (125 MHz, $CDCl_3$): δ = 122.94 (quint, *J* = 3.8 Hz), 123.11, 124.82, 125.77, 125.82, 129.06, 130.38, 130.60, 134.41 (quint, *J* = 5.0 Hz), 140.74, 141.03, 147.84, 148.46, 159.80 (quint, *J* = 33.8 Hz), 167.94 (quint, *J* = 30.0 Hz). ATR-FTIR (KBr): *v* = 3056, 1596, 1498, 1444, 1284, 1110, 782, 696 cm^{-1}.

5-bromo-2-(tetrafluoro(1-(4-methoxyphenyl)-5-phenyl-1*H*-1,2,3-triazol-4-yl)-λ^6-sulfaneyl)pyridine (5t-A)

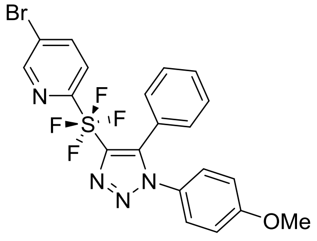

Prepared according to general procedure 2, by stirring at 110 °C for 48 h and isolated by column chromatography (*n*-Hexane/AcOEt, 4/1) to get only **5t**-A as yellow solid in 72% yield (185 mg). mp: 166–167 °C; HRMS (ESI$^+$): *m/z* calcd for $C_{20}H_{15}N_4OF_4NaSBr$ [M+Na]$^+$: 536.9984 found: 536.9991. ^{1}H NMR (300 MHz, $CDCl_3$): δ = 3.78 (s, 3H), 6.83 (td, *J* = 9.0 Hz, 2.1 Hz, 2H), 7.18 (td, *J* = 9.0 Hz, 2.1 Hz, 2H), 7.28–7.39 (m, 5H), 7.70 (d, *J* = 8.7 Hz, 1H), 7.96 (d, *J* = 8.7 Hz, 1H), 8.56 (d, *J* = 2.1 Hz, 1H), ^{19}F NMR (282 MHz, $CDCl_3$): δ = 61.66 (s, 4F), ^{13}C NMR (125 MHz, $CDCl_3$): δ = 55.62, 114.40, 122.90, 123.04 (quint, *J* = 5.0 Hz), 126.67, 126.87, 128.50, 126.68, 129.00, 129.84, 130.55, 134.27 (quint, *J* = 3.8 Hz), 140.93, 148.38, 159.28 (quint, *J* = 32.5 Hz), 160.30, 168.28 (quint, *J* = 31.3 Hz). ATR-FTIR (KBr): *v* = 3066, 1608, 1513, 1444, 1253, 1091, 773, 694 cm^{-1}.

5-bromo-2-(tetrafluoro(1-hexyl-5-phenyl-1*H*-1,2,3-triazol-4-yl)-λ⁶-sulfaneyl)pyridine (5u-A)

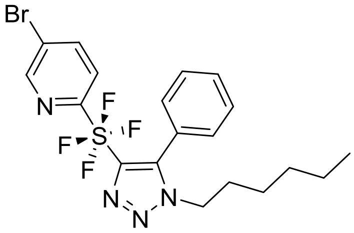

Prepared according to general procedure 2, by stirring at 110 °C for 24 h and separated from its regiosiomer by column chromatography (*n*-Hexane/AcOEt, 4/1) to isolate pure **5u**-A as white solid. mp: 97–98 °C; HRMS (ESI$^+$): *m/z* calcd for $C_{19}H_{21}N_4F_4NaSBr$ [M+Na]$^+$: 515.0504 found: 515.0505. ^1H NMR (300 MHz, $CDCl_3$): δ = 0.83 (t, *J* = 6.6 Hz, 3H), 1.15–1.26 (m, 6H), 1.71–1.80 (m, 2H), 4.09 (t, *J* = 7.5 Hz, 2H), 7.34–7.37 (m, 2H), 7.47–7.51 (m, 3H), 7.66 (d, *J* = 8.7 Hz, 1H), 7.94 (d, *J* = 8.7 Hz, 1H), 8.52 (d, *J* = 2.4 Hz, 1H), ^{19}F NMR (282 MHz, $CDCl_3$): δ = 61.30 (s, 4F), ^{13}C NMR (125 MHz, $CDCl_3$): δ = 14.01, 22.41, 26.10, 29.96, 31.04, 31.08, 49.23, 122.82, 123.01 (quint, *J* = 5.0 Hz), 126.72, 128.79, 130.15 (d, J = 2.5 Hz), 133.76 (quint, *J* = 5.0 Hz), 140.88, 148.33, 159.08 (quint, *J* = 32.5 Hz), 168.33 (quint, *J* = 30.0 Hz). ATR-FTIR (KBr): *v* = 3058, 2931, 1556, 1448, 1095, 771, 696 cm^{-1}.

5-bromo-2-(tetrafluoro(1-hexyl-4-phenyl-1*H*-1,2,3-triazol-5-yl)-λ⁶-sulfaneyl)pyridine (5u-B)

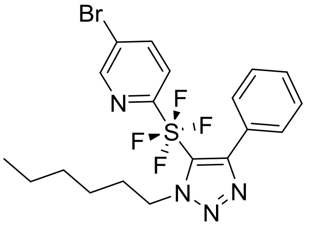

Prepared according to general procedure 2, by stirring at 110 °C for 24 h and separated from its regiosiomer by column chromatography (*n*-Hexane/AcOEt, 4/1) to isolate pure **5u**-A as yellow oil. HRMS (ESI$^+$): *m/z* calcd for $C_{19}H_{21}N_4F_4NaSBr$ [M+Na]$^+$: 515.0504 found: 515.0511. ^1H NMR (300 MHz, $CDCl_3$): δ = 0.83 (t, *J* = 6.6 Hz, 3H), 1.19–1.26 (m, 6H), 1.71–1.78 (m, 2H), 4.09 (t, *J* = 7.5 Hz, 2H), 7.34–7.37 (m, 2H), 7.46–7.51 (m, 3H), 7.67 (d, *J* = 8.7 Hz, 1H), 7.94 (d, *J* = 8.7 Hz, 1H), 8.52 (d, *J* = 2.1 Hz, 1H), ^{19}F NMR (282 MHz, $CDCl_3$): δ = 66.87 (s, 4F), ^{13}C NMR (125 MHz, $CDCl_3$): δ = 14.22, 22.75, 26.89, 29.22, 30.52, 31.87, 52.86, 122.65–122.70 (m), 123.48, 127.96, 128.83, 130.20, 131.34, 141.15, 148.09–149.16 (m), 148.58, 167.77 (quint, *J* = 30.0 Hz). ATR-FTIR (NaCl): *v* = 3055, 2927, 1558, 1448, 1093, 777, 690 cm^{-1}.

Total isolated yield of **5u** is 67% (165 mg).

5-bromo-2-(tetrafluoro(1-octyl-5-phenyl-1H-1,2,3-triazol-4-yl)-λ⁶-sulfaneyl)pyridine (5v-A)

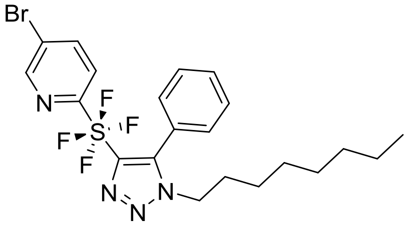

Prepared according to general procedure 2, by stirring at 110 °C for 24 h and separated from its regiosiomer by column chromatography (*n*-Hexane/AcOEt, 4/1) to isolate pure **5v**-A as white solid. HRMS (ESI$^+$): *m/z* calcd for $C_{21}H_{25}N_4F_4NaSBr$ [M+Na]$^+$: 543.0817 found: 543.0817. ^1H NMR (300 MHz, $CDCl_3$): δ = 0.86 (t, *J* = 6.3 Hz, 3H), 1.19–1.26 (m, 10H), 1.76 (t, *J* = 6.0 Hz, 2H), 4.09 (t, *J* = 7.2 Hz, 2H), 7.34–7.37 (m, 2H), 7.46–7.51 (m, 3H), 7.67 (d, *J* = 8.7 Hz, 1H), 7.94 (d, *J* = 8.4 Hz, 1H), 8.53 (d, *J* = 2.4 Hz, 1H); ^{19}F NMR (282 MHz, $CDCl_3$): δ = 61.31 (s, 4F), ^{13}C NMR (125 MHz, $CDCl_3$): δ = 14.18, 22.68, 26.39, 28.84, 29.00, 29.97, 31.75, 49.22, 122.80, 123.01 (quint, *J* = 3.8 Hz), 126.72, 128.78, 130.13, 130.14, 133.75 (quint, *J* = 3.8 Hz), 140.87, 148.31, 159.07 (quint, *J* = 32.5 Hz), 168.33 (quint, *J* = 32.3 Hz). ATR-FTIR (KBr): *v* = 3060, 2933, 1563, 1481, 1452, 1361, 1072, 784, 629 cm^{-1}.

5-bromo-2-(tetrafluoro(1-octyl-4-phenyl-1H-1,2,3-triazol-5-yl)-λ[6]-sulfaneyl)pyridine (5v-B)

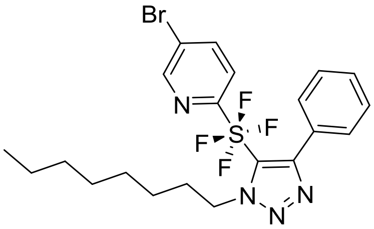

Prepared according to general procedure 2, by stirring at 110 °C for 24 h and separated from its regiosiomer by column chromatography (*n*-Hexane/AcOEt, 4/1) to isolate pure **5v**-B as yellow oil. mp: 90–91 °C; HRMS (ESI$^+$): *m/z* calcd for $C_{21}H_{25}N_4F_4NaSBr$ [M+Na]$^+$: 543.0817 found: 543.0809. ^{1}H NMR (300 MHz, $CDCl_3$): δ = 0.88 (t, *J* = 6.6 Hz, 3H), 1.19–1.49 (m, 10H), 2.04–2.17 (m, 2H), 4.68 (t, *J* = 7.8 Hz, 2H), 7.40–7.43 (m, 3H), 7.52–7.55 (m, 2H), 7.58 (d, *J* = 8.7 Hz, 1H), 7.97 (d, *J* = 8.7 Hz, 1H), 8.58 (d, *J* = 2.4 Hz, 1H), ^{19}F NMR (282 MHz, $CDCl_3$): δ = 66.88 (s, 4F), ^{13}C NMR (125 MHz, $CDCl_3$): δ = 14.22, 22.75, 26.89, 29.22, 30.52, 31.87, 52.86, 122.65–122.70 (m), 123.48, 127.27, 127.96, 128.83, 130.21, 131.34, 141.15, 144.02, 148.09–149.13 (m), 148.58, 167.77 (quint, *J* = 30.0 Hz). ATR-FTIR (NaCl): *ν* = 3062, 2945, 1558, 1448, 1359, 1093, 777, 692 cm^{-1}.

Total isolated yield of **5v** is 66% (172 mg).

5-bromo-2-((1-cyclohexyl-5-phenyl-1H-1,2,3-triazol-4-yl)tetrafluoro-λ[6]-sulfaneyl)pyridine (5w-A)

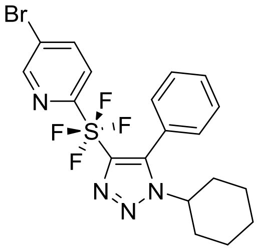

Prepared according to general procedure 2, by stirring at 110 °C for 48 h and separated from its regiosiomer by column chromatography (*n*-Hexane/AcOEt, 4/1) to isolate pure **5w**-A as white solid. mp: 90–91 °C; HRMS (ESI$^+$): *m/z* calcd for $C_{19}H_{19}N_4F_4NaSBr$ [M+Na]$^+$: 513.0348 found: 513.0354. ^{1}H NMR (300 MHz, $CDCl_3$): δ = 1.11–1.34 (m, 3H), 1.63–1.72 (m, 1H), 1.83–2.16 (m, 6H), 3.75–3.86 (m, 1H), 7.32–7.35 (m, 2H), 7.48–7.51 (m, 3H), 7.66 (d, *J* = 8.7 Hz, 1H), 7.93 (d, *J* = 8.4 Hz, 1H), 8.53 (d, *J* = 2.1 Hz, 1H), ^{19}F NMR (282 MHz, $CDCl_3$): δ = 61.47 (s, 4F), ^{13}C NMR (125 MHz, $CDCl_3$): δ = 24.92, 25.44, 33.23, 59.16, 122.78, 123.05 (quint, *J* = 3.8 Hz), 126.96, 128.80, 130.11, 130.18, 133.11 (quint, *J* = 5.0 Hz), 140.85, 148.33, 158.90 (quint, *J* = 31.3 Hz), 168.44 (quint, *J* = 31.3 Hz). ATR-FTIR (KBr): *ν* = 3072, 2940, 1554, 1446, 1355, 1093, 777, 700 cm^{-1}.

5-bromo-2-((1-cyclohexyl-4-phenyl-1H-1,2,3-triazol-5-yl)tetrafluoro-λ[6]-sulfaneyl)pyridine (5w-B)

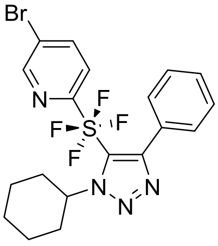

Prepared according to general procedure 2, by stirring at 110 °C for 48 h and separated from its regiosiomer by column chromatography (*n*-Hexane/AcOEt, 4/1) to isolate pure **5w**-B as white solid. mp: 116–117 °C; HRMS (ESI$^+$): *m/z* calcd for $C_{19}H_{19}N_4F_4NaSBr$ [M+Na]$^+$: 513.0348 found: 513.0346. ^{1}H NMR (300 MHz, $CDCl_3$): δ = 1.34–1.47 (m, 3H), 1.72–1.78 (m, 1H), 1.98 (d, *J* = 12.0 Hz, 2H), 2.13–2.23 (m, 4H), 4.85–4.95 (m, 1H), 7.39–7.42 (m, 3H), 7.50–7.53 (m, 2H), 7.59 (d, *J* = 8.7 Hz, 1H), 7.97 (d, *J* = 8.4 Hz, 1H), 8.57 (d, *J* = 2.4 Hz, 1H), ^{19}F NMR (282 MHz, $CDCl_3$): δ = 66.97 (s, 4F), ^{13}C NMR (125 MHz, $CDCl_3$): δ = 25.21, 25.96, 33.82, 62.82, 122.71, 123.45, 127.89, 128.76, 130.33, 131.46, 141.13, 143.32, 148.05 (quint, *J* = 32.5 Hz), 148.55, 167.91 (quint, *J* = 30.0 Hz). ATR-FTIR (KBr): *ν* = 3062, 2942, 1565, 1475, 1450, 1371, 1095, 777, 700 cm^{-1}.

Total isolated yield of **5w** is 52% (128 mg).

2-((1-((3s,5s,7s)-adamantan-1-yl)-5-phenyl-1H-1,2,3-triazol-4-yl)tetrafluoro-λ⁶-sulfaneyl)-5-bromopyridine (5x-A)

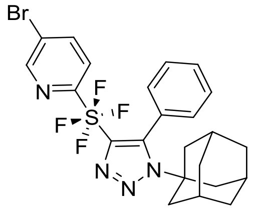

Prepared according to general procedure 2, by stirring at 110 °C for 48 h and isolated by column chromatography (*n*-Hexane/AcOEt, 4/1) to get pure **5x**-A as white solid in 37% yield (100 mg). mp: 170–171 °C; HRMS (ESI$^+$): *m/z* calcd for $C_{23}H_{23}N_4NaSBrF_4$ $[M+Na]^+$: 565.0661 found: 565.0669. ^{1}H NMR (300 MHz, $CDCl_3$): δ = 1.53–1.70 (m, 6H), 2.09 (brs, 3H), 2.16 (s, 6H), 7.34–7.49 (m, 5H), 7.61 (d, *J* = 8.7 Hz, 1H), 7.91 (d, *J* = 8.4 Hz, 1H), 8.50 (d, *J* = 2.1 Hz, 1H), ^{19}F NMR (282 MHz, $CDCl_3$): δ = 61.27 (s, 4F), ^{13}C NMR (125 MHz, $CDCl_3$): δ = 29.73, 35.68, 42.61, 66.52, 122.65 123.00 (quint, *J* = 3.8 Hz), 127.82, 128.47, 129.79, 131.50, 133.13–133.18 (m), 140.76, 148.76, 160.68 (quint, *J* = 30.0 Hz), 168.45 (quint, *J* = 31.3 Hz). ATR-FTIR (KBr): *v* = 3054, 2937, 1552, 1477, 1444, 1093, 779, 698 cm^{-1}.

2-((4-((5-bromopyridin-2-yl)tetrafluoro-λ⁶-sulfaneyl)-5-phenyl-1H-1,2,3-triazol-1-yl)(6-methoxyquinolin-4-yl)methyl)-5-vinylquinuclidine (5y-A)

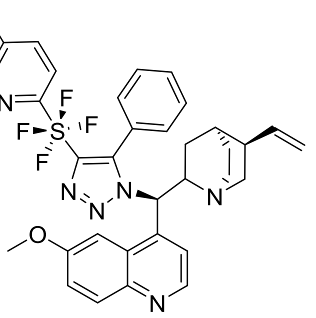

Prepared according to general procedure 2, by stirring at 110 °C for 48 h and separated from its regiosiomer by column chromatography (*n*-Hexane/AcOEt, 2/3) to isolate pure **5y**-A as white solid. mp: 193–194 °C; HRMS (ESI$^+$): *m/z* calcd for $C_{33}H_{32}N_6OF_4SBr$ $[M+Na]^+$: 715.1478 found: 715.1479. ^{1}H NMR (300 MHz, $(CD_3)_2CO$): δ = 0.76 (t, *J* = 9.9 Hz, 1H), 1.52–1.61 (m, 4H), 2.29 (brs, 1H), 2.73–2.83 (m, 3H), 3.09–3.17 (m, 2H), 3.77 (s, 3H), 4.25 (brs, 1H), 4.97–5.08 (m, 2H), 5.92–6.03 (m, 1H), 6.94 (brs, 2H), 7.40 (dd, *J* = 9.3 Hz, 2.4 Hz, 3H), 7.60–7.62 (m, 2H), 7.71 (d, *J* = 8.7 Hz, 2H), 7.99 (d, *J* = 9.3 Hz, 1H), 8.21 (d, *J* = 8.7 Hz, 1H), 8.56 (d, *J* = 2.4 Hz, 1H), 8.73 (brs, 1H), ^{19}F NMR (282 MHz, $CDCl_3$): δ = 61.35 (s, 4F), ^{13}C NMR (125 MHz, $(CD_3)_2CO$): δ = 26.19, 28.44, 28.60, 40.52, 42.00, 55.79, 56.27, 100.90, 114.70, 121.98, 122.75, 123.43, 123.75 (quint, *J* = 5.0 Hz), 127.36, 128.09, 128.68, 129.47, 131.10, 131.51, 131.67, 132.97, 135.30, 139.82, 142.25, 142.97, 145.66, 148.95, 159.08, 159.66 (quint, *J* = 35.0 Hz), 169.16 (quint, *J* = 31.3 Hz). ATR-FTIR (KBr): *v* = 3062, 2946, 1508, 1475, 1446, 1241, 1091, 1027, 777, 696 cm^{-1}.

2-((5-((5-bromopyridin-2-yl)tetrafluoro-λ⁶-sulfaneyl)-4-phenyl-1H-1,2,3-triazol-1-yl)(6-methoxyquinolin-4-yl)methyl)-5-vinylquinuclidine (5y-B)

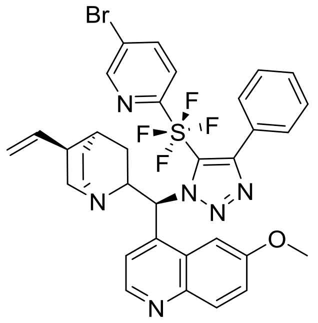

Prepared according to general procedure 2, by stirring at 110 °C for 48 h and separated from its regiosiomer by column chromatography (*n*-Hexane/AcOEt, 2/3) to isolate pure **5y**-B as white solid. mp: 172–173 °C; HRMS (ESI$^+$): *m/z* calcd for $C_{33}H_{32}N_6OSBrF_4$ $[M+Na]^+$: 715.1478 found: 715.1451. ^{1}H NMR (300 MHz, $CDCl_3$): δ = 0.94–1.02 (m, 1H), 1.25–1.38 (m, 1H), 1.60–1.72 (m, 4H), 2.27 (brs, 1H), 2.83–2.96 (m, 2H), 3.12–3.20 (m, 1H), 3.26–3.37 (m, 1H), 4.02 (s, 3H), 4.44–4.54 (m, 1H), 4.98–5.06 (m, 2H), 5.79–5.91 (m, 1H), 7.04 (d, *J* = 9.9 Hz, 1H), 7.38–7.41 (m, 5H), 7.53–7.61 (m, 2H), 7.88–7.94 (m, 2H), 8.05 (d, *J* = 9.3 Hz, 1H), 8.51 (d, *J* = 2.1 Hz, 1H), 8.80 (d, *J* = 4.5 Hz, 1H), ^{19}F NMR (282 MHz, $CDCl_3$): δ = 67.96 (s, 4F), ^{13}C NMR (125 MHz, $CDCl_3$): δ = 25.32, 37.75, 28.39, 39.72, 41.38, 55.22, 55.55, 60.58, 61.05, 101.13, 114.80, 120.63, 122.12, 122.56, 123.48, 127.81, 127.87,

128.82, 130.41, 131.28, 132.16, 141.11, 141.21, 141.76, 143.95, 144.83, 148.18, 148.42, 149.20–149.73 (m), 158.11, 167.42–167.89 (m). ATR-FTIR (KBr): *v* = 3060, 2938, 1508, 1475, 1446, 1241, 1093, 1031, 775, 700 cm^{-1}.

Total isolated yield of **5y** is 66% (236 mg).

3-(4-((5-bromopyridin-2-yl)tetrafluoro-λ^6-sulfaneyl)-5-phenyl-1H-1,2,3-triazol-1-yl)-10,13-dimethylhexadecahydro-17H-cyclopenta[a]phenanthren-17-one (5z-A)

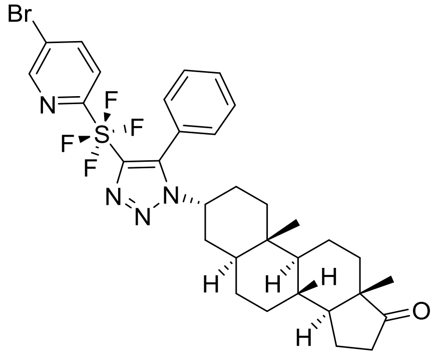

Prepared according to general procedure 2, by stirring at 110 °C for 48 h and separated from its regiosiomer by column chromatography (*n*-Hexane/AcOEt, 4/1) to isolate pure **5z**-A as white solid. mp: 108–109 °C; HRMS (ESI$^+$): *m/z* calcd for $C_{32}H_{37}N_4ONaSBrF_4$ $[M+Na]^+$: 703.1705 found: 703.1700. ^1H NMR (300 MHz, $CDCl_3$): δ = 0.81 (s, 3H), 0.86 (s, 3H), 0.95–1.20 (m, 3H), 1.25–1.36 (m, 4H), 1.42–1.59 (m, 4H), 1.65–1.82 (m, 4H), 1.87–2.17 (m, 6H), 2.39–2.48 (m, 1H), 4.31 (s, 1H), 7.30–7.33 (m, 2H), 7.46 (m, 3H), 7.66 (d, *J* = 8.7 Hz, 1H), 7.93 (d, *J* = 8.7 Hz, 1H), 8.54 (d, *J* = 2.4 Hz, 1H), ^{19}F NMR (282 MHz, $CDCl_3$): δ = 61.40 (s, 4F), ^{13}C NMR (125 MHz, $CDCl_3$): δ = 0.04, 9.62, 11.93, 18.11, 19.87, 24.35, 26.05, 28.54, 29.58, 31.22 (d, *J* = 6.3 Hz), 33.09, 33.73, 33.98, 37.17, 45.90, 49.46, 52.00, 52.35, 114.53, 120.77, 121.03 (quint, *J* = 3.8 Hz), 125.27, 126.78, 128.00, 128.20, 131.38 (quint, *J* = 3.8 Hz), 138.83, 146.30, 157.03 (quint, *J* = 31.3 Hz), 166.39 (quint, *J* = 31.3 Hz). ATR-FTIR (KBr): *v* = 3064, 2937, 1735, 1548, 1473, 1446, 1099, 779, 698 cm^{-1}.

3-(5-((5-bromopyridin-2-yl)tetrafluoro-λ^6-sulfaneyl)-4-phenyl-1H-1,2,3-triazol-1-yl)-10,13-dimethylhexadecahydro-17H-cyclopenta[a]phenanthren-17-one (5z-B)

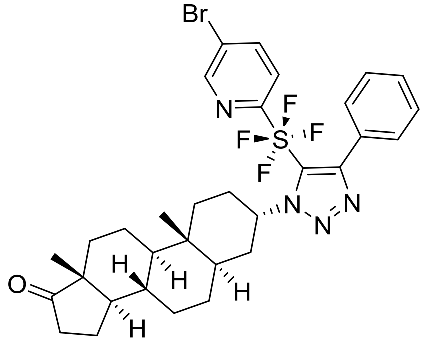

Prepared according to general procedure 2, by stirring at 110 °C for 48 h and separated from its regiosiomer by column chromatography (*n*-Hexane/AcOEt, 4/1) to isolate pure **5z**-B as white solid. mp: 127–128 °C; HRMS (ESI$^+$): *m/z* calcd for $C_{32}H_{37}N_4ONaSBrF_4$ $[M+Na]^+$: 703.1705 found: 703.1696. ^1H NMR (300 MHz, $CDCl_3$): δ = 0.88 (s, 3H), 0.90 (s, 3H), 1.05–1.15 (m, 3H), 1.21–1.38 (m, 5H), 1.43–1.60 (m, 2H), 1.72–1.84 (m, 4H), 1.87–1.99 (m, 2H), 2.03–2.23 (m, 5H), 2.40–2.49 (m, 1H), 5.41 (s, 1H), 7.40–7.42 (m, 3H), 7.51–7.56 (m, 3H), 7.96 (d, *J* = 7.2 Hz, 1H), 8.57 (d, *J* = 2.1 Hz, 1H), ^{19}F NMR (282 MHz, $CDCl_3$): δ = 67.38 (s, 4F), ^{13}C NMR (125 MHz, $CDCl_3$): δ = 11.72, 13.99, 20.17, 21.91, 26.79, 28.10, 30.62, 31.65, 33.33, 33.44, 35.21, 35.65, 36.03, 39.05, 47.96, 51.54, 54.06, 56.67, 122.67, 123.43, 127.89, 128.74, 130.29, 131.66, 141.14, 143.40, 148.25–148.78 (m), 148.55, 167.71–168.18 (m). ATR-FTIR (KBr): *v* = 3058, 2935, 1737, 1560, 1473, 1448, 1093, 775, 684 cm^{-1}.

Total isolated yield of **5z** is 37% (126 mg).

1-benzyl-4-((4-bromophenyl)tetrafluoro-λ^6-sulfaneyl)-5-phenyl-1*H*-1,2,3-triazole (7a-A)

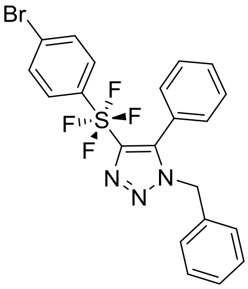

Prepared according to general procedure 2, by stirring at 110 °C for 24 h and separated from its regiosiomer by column chromatography (*n*-Hexane/AcOEt, 4/1) to isolate pure **7a**-A as white solid. mp: 143–145 °C; HRMS (ESI$^+$): *m/z* calcd for $C_{21}H_{16}N_3F_4NaSBr$ [M+Na]$^+$: 520.0082 found: 521.0073. ^{1}H NMR (300 MHz, $CDCl_3$): δ = 5.30 (s, 2H), 6.94–6.96 (m, 2H), 7.15 (d, *J* = 7.2 Hz, 2H), 7.20–7.25 (m, 3H), 7.37–7.50 (m, 5H), 7.58 (d, *J* = 9.0 Hz, 2H), ^{19}F NMR (282 MHz, $CDCl_3$): δ = 71.25 (s, 4F), ^{13}C NMR (125 MHz, $CDCl_3$): δ = 53.06, 124.61, 126.45, 127.72 (quint, *J* = 3.8 Hz), 127.96, 128.52, 128.59, 128.83, 130.09, 130.25, 131.32, 131.37, 136.60 (quint, *J* = 3.8 Hz), 134.21, 137.54, 158.63 (quint, *J* = 25 Hz), 160.46 (quint, *J* = 33.8 Hz). ATR-FTIR (KBr): *ν* = 3060, 1574, 1477, 1448, 1325, 1070, 762, 663 cm^{-1}.

1-benzyl-5-((4-bromophenyl)tetrafluoro-λ^6-sulfaneyl)-4-phenyl-1*H*-1,2,3-triazole (7a-B)

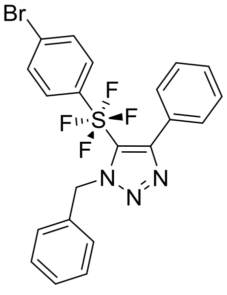

Prepared according to general procedure 2, by stirring at 110 °C for 24 h and separated from its regiosiomer by column chromatography (*n*-Hexane/AcOEt, 4/1) to isolate pure **7a**-B as white solid. mp: 132–133 °C; HRMS (ESI$^+$): *m/z* calcd for $C_{21}H_{16}N_3F_4NaSBr$ [M+Na]$^+$: 520.0082 found: 521.0095. ^{1}H NMR (300 MHz, $CDCl_3$): δ = 5.90 (s, 2H), 7.32–7.43 (m, 8H), 7.47–7.57 (m, 6H), ^{19}F NMR (282 MHz, $CDCl_3$): δ = 77.90 (s, 4F), ^{13}C NMR (125 MHz, $CDCl_3$): δ = 55.78, 125.41, 127.52, (t, *J* = 5 Hz), 127.68, 128.01, 128.38, 128.84, 128.90, 130.19, 131.28, 131,66, 135.21, 144.03, 149.78 (quint, *J* = 37.5 Hz), 158.02 (quint, *J* = 23.8 Hz). ATR-FTIR (KBr): *ν* = 3064, 1573, 1475, 1454, 1329, 1068, 781, 756, 656 cm^{-1}.

Total isolated yield of **7b** is 56% (140 mg).

1-benzyl-5-phenyl-4-(tetrafluoro(phenyl)-λ^6-sulfaneyl)-1H-1,2,3-triazole (7b-A)

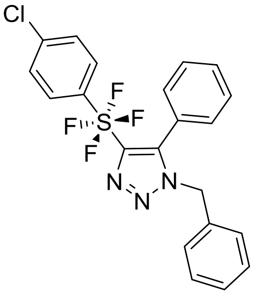

Prepared according to general procedure 2, by stirring at 110 °C for 24 h and separated from its regiosiomer by column chromatography (*n*-Hexane/AcOEt, 85/15) to isolate pure **7b**-A as white solid. mp: 137–138 °C; HRMS (ESI$^+$): *m/z* calcd for $C_{21}H_{16}N_3F_4NaSCl$ [M+Na]$^+$: 476.0587 found: 476.0589. ^{1}H NMR (300 MHz, $CDCl_3$): δ = 5.30 (s, 2H), 6.94–6.96 (m, 2H), 7.14 (d, *J* = 7.2 Hz, 2H), 7.21–7.31 (m, 5H), 7.37–7.51 (m, 3H), 7.66 (d, *J* = 9.0 Hz, 2H), ^{19}F NMR (282 MHz, $CDCl_3$): δ = 72.38 (s, 4F), ^{13}C NMR (125 MHz, $CDCl_3$): δ = 53.08, 126.49, 127.54 (quint, *J* = 5.0 Hz), 127.98, 128.32, 128.54, 128.61, 128.85, 130.10, 130.27, 133.62 (quint, *J* = 5.0 Hz), 134.22, 136.34, 158.06 (quint, *J* = 25.0 Hz), 160.51 (quint, *J* = 33.8 Hz). ATR-FTIR (KBr): *ν* = 3037, 1531, 1477, 1454, 1099, 795, 754 cm^{-1}.

1-benzyl-4-phenyl-5-(tetrafluoro(phenyl)-λ[6]-sulfaneyl)-1H-1,2,3-triazole (7b-B)

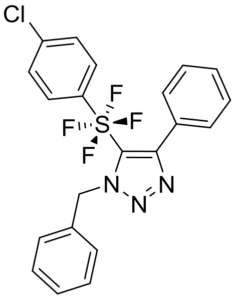

Prepared according to general procedure 2, by stirring at 110 °C for 24 h and separated from its regiosiomer by column chromatography (*n*-Hexane/AcOEt, 85/15) to isolate pure **7b**-B as white solid. mp: 129–132 °C; HRMS (ESI$^+$): *m/z* calcd for $C_{21}H_{16}N_3F_4NaSCl$ $[M+Na]^+$: 476.0587 found: 476.0590. ^{1}H NMR (300 MHz, $CDCl_3$): δ = 5.91 (s, 2H), 7.34–7.43 (m, 8H), 7.53–7.55 (m, 2H), 7.88 (d, *J* = 9.3 Hz, 2H), 8.23 (d, *J* = 9.0 Hz, 2H), ^{19}F NMR (282 MHz, $CDCl_3$): δ = 78.04 (s, 4F), ^{13}C NMR (125 MHz, $CDCl_3$): δ = 55.78, 127.35 (t, *J* = 5.0 Hz), 127.68, 128.01, 128.38, 128.64, 128.84, 128.91, 130.19, 131.28, 135.22, 137.14, 144.02, 149.83 (quint, *J* = 36.3 Hz), 157.43 (quint, *J* = 22.5 Hz). ATR-FTIR (KBr): *v* = 3035, 1540, 1477, 1455, 1097, 827, 767 cm^{-1}.

1-benzyl-5-phenyl-4-(tetrafluoro(4-nitrophenyl)-λ[6]-sulfaneyl)-1H-1,2,3-triazole (7c-A)

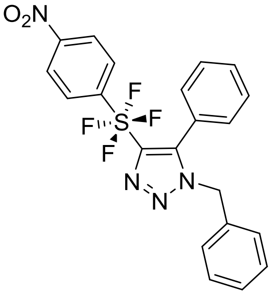

Prepared according to general procedure 2, by stirring at 110 °C for 24 h and separated from its regiosiomer by column chromatography (*n*-Hexane/AcOEt, 85/15) to isolate pure **7c**-A as white solid. mp: 140–142 °C; HRMS (ESI$^+$): *m/z* calcd for $C_{21}H_{16}N_4O_2F_4NaS$ $[M+Na]^+$: 487.0828 found: 487.0820. ^{1}H NMR (300 MHz, $CDCl_3$): δ = 5.32 (s, 2H), 6.97 (d, *J* = 6.0 Hz, 2H), 7.16 (d, *J* = 7.2 Hz, 2H), 7.26 (d, *J* = 6.6 Hz, 3H), 7.40–7.54, 7.91 (d, *J* = 9.0 Hz, 2H), 8.20 (d, *J* = 8.7 Hz, 2H), ^{19}F NMR (282 MHz, $CDCl_3$): δ = 71.01 (s, 4F), ^{13}C NMR (125 MHz, $CDCl_3$): δ = 53.17, 123.66, 126.23, 127.63 (quint, *J* = 5.0 Hz), 128.01, 128.64, 128.69, 128.90, 130.23, 130.27, 133.85 (quint, *J* = 3.8 Hz), 134.11, 148.31, 159.83 (quint, *J* = 32.5 Hz), 163.55 (quint, *J* = 26.3 Hz), ATR-FTIR (KBr): *v* = 3068, 1612, 1529, 1481, 1450, 1089, 757cm^{-1}.

1-benzyl-4-phenyl-5-(tetrafluoro(4-nitrophenyl)- λ[6]-sulfaneyl)-1H-1,2,3-triazole (7c-B)

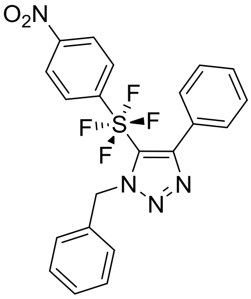

Prepared according to general procedure 2, by stirring at 110 °C for 24 h and separated from its regiosiomer by column chromatography (*n*-Hexane/AcOEt, 85/15) to isolate pure **7c**-B as white solid. mp: 121–122 °C; HRMS (ESI$^+$): *m/z* calcd for $C_{21}H_{16}N_4O_2F_4NaS$ $[M+Na]^+$: 487.0828 found: 487.0832. ^{1}H NMR (300 MHz, $CDCl_3$): δ = 5.91 (s, 2H), 7.34–7.43 (m, 8H), 7.53–7.55 (m, 2H), 7.88 (d, *J* = 9.3 Hz, 2H), 8.22 (d, *J* = 9.0 Hz, 2H), ^{19}F NMR (282 MHz, $CDCl_3$): δ = 77.52 (s, 4F), ^{13}C NMR (125 MHz, $CDCl_3$): δ = 58.83, 123.89, 127.45 (t, *J* = 5.0 Hz), 127.57, 128.05, 128.45, 128.87, 129.03, 130.11, 130.98, 134.99, 144.26, 148.63, 149.12 (quint, *J* = 35.0 Hz), 162.77 (quint, *J* = 23.8 Hz). ATR-FTIR (KBr): *v* = 3064, 1610, 1535, 1479, 1448, 1079, 767 cm^{-1}.

2-((1-benzyl-5-phenyl-1H-1,2,3-triazol-4-yl)tetrafluoro-λ⁶-sulfaneyl)-5-(4-phenoxyphenyl)pyridine (8a)

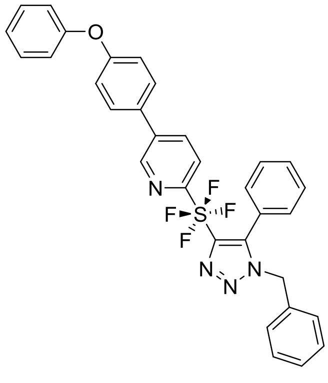

Prepared according to modified literature procedure.[6] $Pd(PPh_3)_4$ (3 mg, 3 mol%) was added to a solution of **5a** (50 mg, 0.1 mmol) in benzene (0.2 mL), followed by an 2M aqueous solution of Na_2CO_3 (0.1 mL), and the reaction was stirred at room temperature. A solution of (4-phenoxyphenyl)boronic acid (24 mg) in ethanol (0.1 mL) was added and the reaction mixture was heated to 80 °C and refluxed for 22 h. After the designated time, the reaction was allowed to cool to room temperature and water was added. It was extracted with AcOEt and the organic layer was dried over Na_2SO_4. The solvent was concentrated *in vacuo* to give crude product which was purified by silica gel column chromatography (*n*-Hexane/AcOEt, 7/3) to give the desired product **6a** as white solid in 54% yield (32 mg). mp: 149–150 °C; HRMS (ESI$^+$): *m/z* calcd for $C_{32}H_{24}N_4OF_4NaS$ [M+Na]$^+$: 611.1505 found: 611.1502. ^{1}H NMR (300 MHz, $CDCl_3$): δ 5.32 (s, 2H), 6.97 (dd, *J* = 6.9 Hz, 1.5 Hz, 2H), 7.18 (d, *J* = 6.9 Hz, 2H), 7.22–7.29 (m, 3H), 7.30–7.42 (m, 4H), 7.44–7.50 (m, 3H), 7.57 (d, *J* = 8.1 Hz, 1H), 7.73 (d, *J* = 7.2 Hz, 1H), 7.87 (d, *J* = 8.1 Hz, 2H), 8.05 (d, *J* = 8.7 Hz, 1H), 8.75 (d, *J* = 2.1 Hz, 1H), ^{19}F NMR (282 MHz, $CDCl_3$): δ = 60.82 (s, 4F), ^{13}C NMR (125 MHz, $CDCl_3$): δ = 53.11, 112.25, 117.87, 119.87, 121.86 (quint, *J* = 3.8 Hz), 123.82, 125.43, 125.55, 126.47, 128.02, 128.6 (d, *J* = 2.5 Hz), 128.87, 130.12, 130.34, 130.83, 133.95–134.01 (m), 134.29, 136.65, 142.66, 145.47, 155.90, 159.65 (quint, *J* = 32.5 Hz), 168.78 (quint, *J* = 30.0 Hz). ATR-FTIR (KBr): *ν* = 3064, 1558, 1490, 1461, 1245, 773, 701 cm^{-1}.

5-(benzofuran-3-yl)-2-((1-benzyl-5-phenyl-1H-1,2,3-triazol-4-yl)tetrafluoro-λ⁶-sulfaneyl)pyridine (8b)

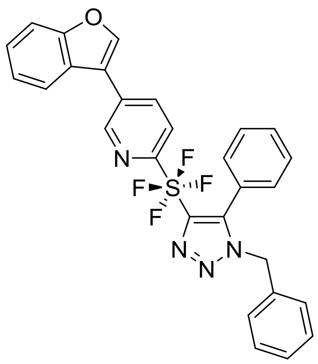

Prepared according to modified literature procedure.[7] In a flame dried schlenk tube in argon atmosphere, **5a** (100 mg, 0.2 mmol), $PdCl_2(PPh_3)_2$ (7 mg, 5 mol%), K_3PO_4 (127 mg, 0.6 mmol), benzofuran-3-ylboronic acid (78 mg, 0.48 mmol) and toluene (2.4 mL) was added. The reaction mixture was evacuated and backfilled with argon and allowed to stir at 100 °C for 48 h. After the designated time, the reaction was allowed to cool to room temperature and diluted with water. It was extracted with CH_2Cl_2 and the organic layer was dried over Na_2SO_4. The solvent was concentrated *in vacuo* to give crude product which was purified by silica gel column chromatography (*n*-Hexane/AcOEt, 7/3) to give the desired product **6b** as light yellow solid in 46% yield (50 mg). mp: 143–144 °C; HRMS (ESI$^+$): *m/z* calcd for $C_{28}H_{20}N_4OF_4NaS$ [M+Na]$^+$: 559.1192 found: 559.1184. ^{1}H NMR (300 MHz, $CDCl_3$): δ = 5.33 (s, 2H), 6.98 (dd, *J* = 6.0 Hz, 3.0 Hz, 2H), 7.19 (d, *J* = 9.0 Hz, 2H), 7.22–7.29 (m, 3H), 7.30–7.42 (m, 4H), 7.45–7.50 (m, 1H), 7.58 (d, *J* = 9.0 Hz, 1H), 7.74 (d, *J* = 9.0 Hz, 1H), 7.87 (d, *J* = 9.0 Hz, 2H), 8.05 (d, *J* = 9.0 Hz, 1H), 8.75 (d, *J* = 3.0 Hz, 1H), ^{19}F NMR (282 MHz, $CDCl_3$): δ = 60.72 (s, 4F), ^{13}C NMR (125 MHz, $CDCl_3$): δ = 53.11, 112.25, 117.87, 119.86, 121.86 (quint, *J* = 3.8 Hz), 123.82, 125.43, 125.55, 126.47, 128.02, 128.60, 128.63, 128.87, 130.12, 130.34, 130.83, 134.01 (quint, *J* = 3.8 Hz), 134.29, 136.65, 142.66, 145.47, 155.90, 159.65 (quint, *J* = 32.5 Hz), 168.78 (quint, *J* = 30.0 Hz). ATR-FTIR (KBr): *ν* = 3127, 3046, 1565, 1477, 1452, 1222, 1095, 777, 701 cm^{-1}.

Reference:

1. F. Himo, T. Lovell, R. Hilgraf, V. V. Rostovtsev, L. Noodleman, K. B. Sharpless, V. V. Fokin, *J. Am. Chem. Soc.*, 2005, **127**, 210.
2. F.-H. Cui, J. Chen, Z.-Y. Mo, S.-X. Su, Y.-Y. Chen, X.-L. Ma, H.-T. Tang, H.-S Wang, Y.-M. Pan and Y.-L. Xu, *Org. Lett.* 2018, **20**, 925.
3. S. Ding, G. Jia and J. Sun, *Angew. Chem. Int. Ed.*, 2014, **53**, 1877.
4. P. Destito, J. R. Couceiro, H. Faustino, F. Lopez and J. L. Mascarenas, *Angew. Chem. Int. Ed.,* 2017, **56**, 10766.
5. L. Zhong, P. R. Savoie, A. S. Filatov and J. T. Welch, *Angew. Chem., Int. Ed.,* 2014, **53**, 526
6. S. Reimann, S. Parpart, P. Ehlers, M. Sharif, A. Spannenbergb and P. Langer *Org. Biomol. Chem.,* 2015, **13**, 6832.
7. S. He, P. Li, X. Dai, H. Liu, Z. Lai, D. Xiao, C. C. McComas, C. Du, Y. Liu, J. Yin, Q. Dang, N. Zorn, X. Peng, R. P. Nargund, A. Palani *Tetrahedron Lett.,* 2017, **58**, 1373.

X-ray crystal structure:

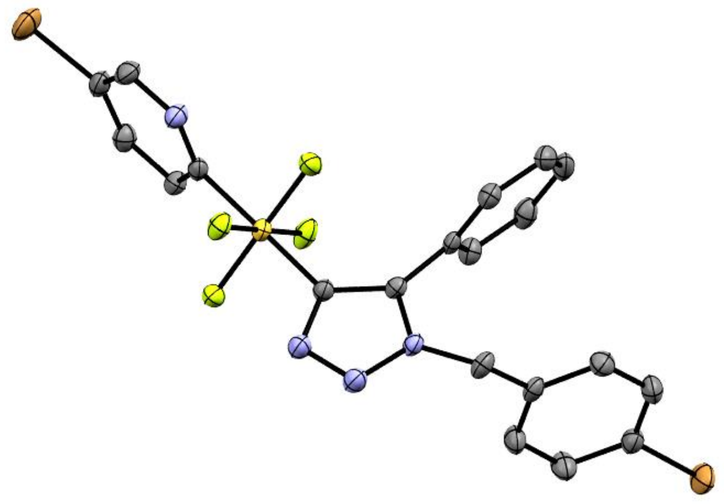

Fig. S1: Ortep diagram of **5m** isomer A drawn at 50% probability. The hydrogen atoms have been omitted for clarity.

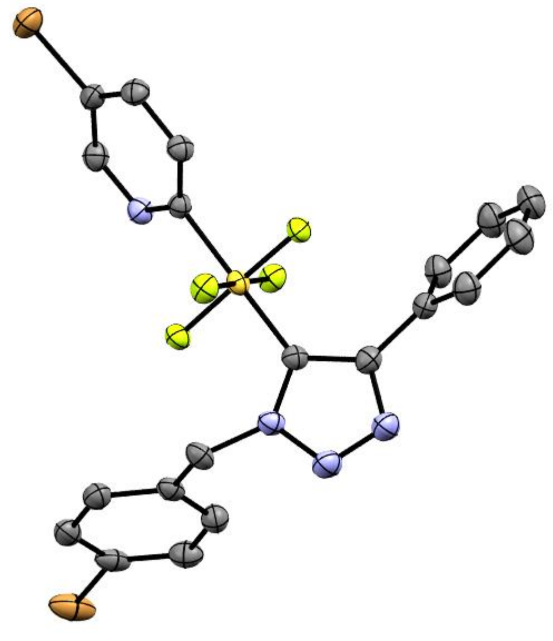

Fig. S2: Ortep diagram of **5m** isomer B drawn at 50% probability. The hydrogen atoms have been omitted for clarity.

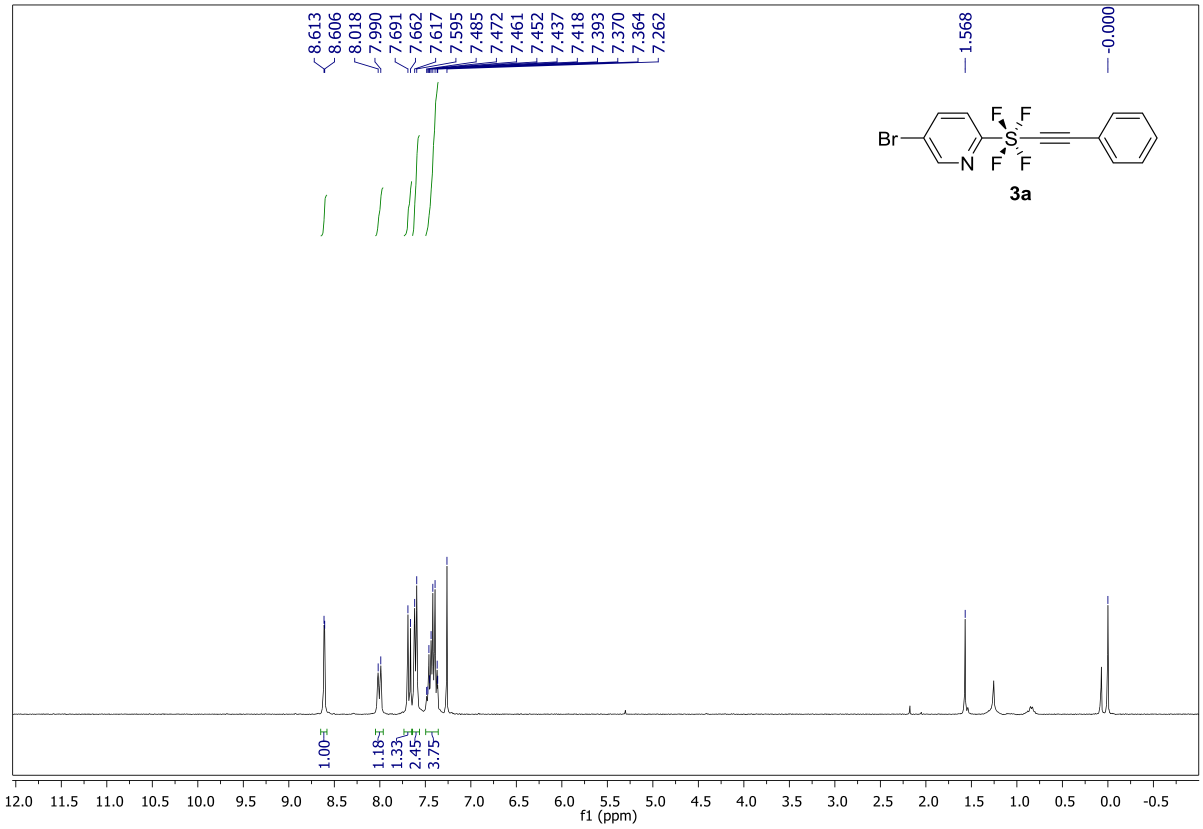
8.613
8.606
8.018
7.990
7.691
7.662
7.617
7.595
7.485
7.472
7.461
7.452
7.437
7.418
7.393
7.370
7.364
7.262
1.568
-0.000
Br
N
F
S
3a
1.00
1.18
1.33
2.45
3.75
12.0 11.5 11.0 10.5 10.0 9.5 9.0 8.5 8.0 7.5 7.0 6.5 6.0 5.5 5.0 4.5 4.0 3.5 3.0 2.5 2.0 1.5 1.0 0.5 0.0 -0.5
f1 (ppm)

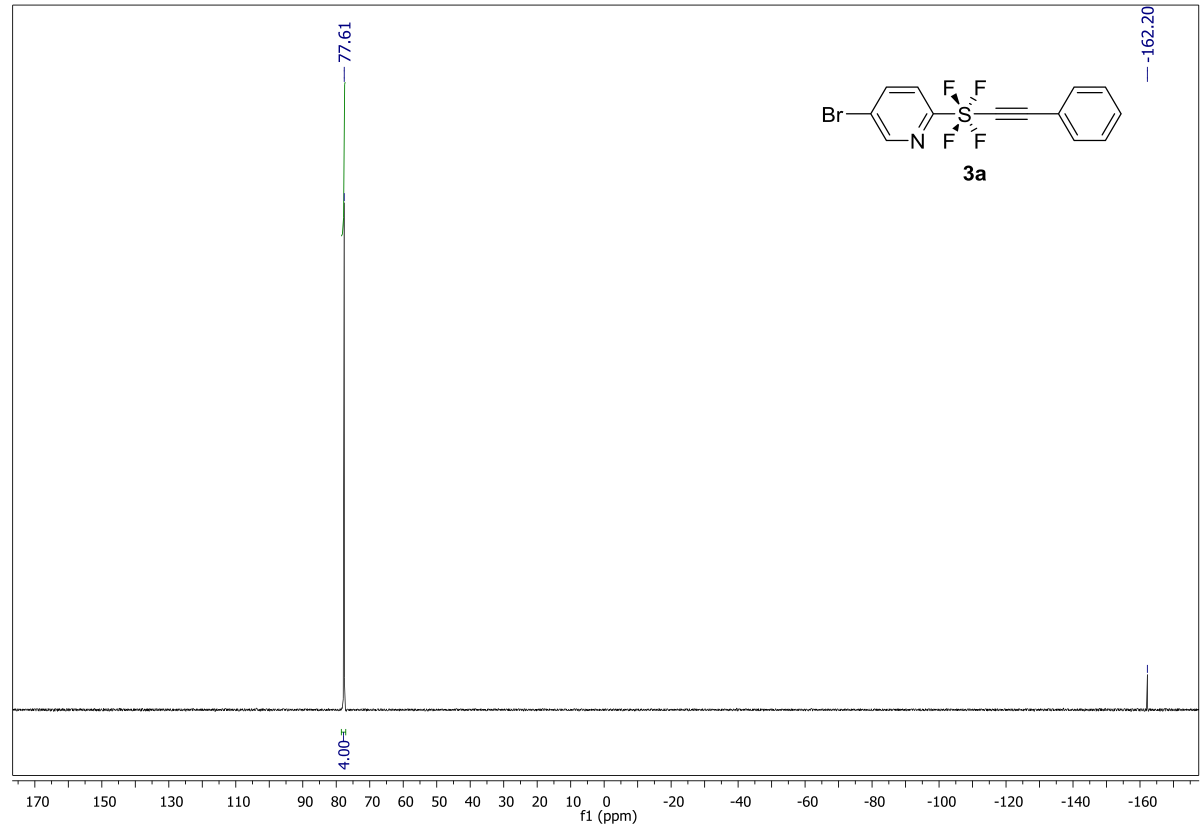
77.61
-162.20
Br
N
F
S
3a
4.00
170 150 130 110 90 80 70 60 50 40 30 20 10 0 -20 -40 -60 -80 -100 -120 -140 -160
f1 (ppm)

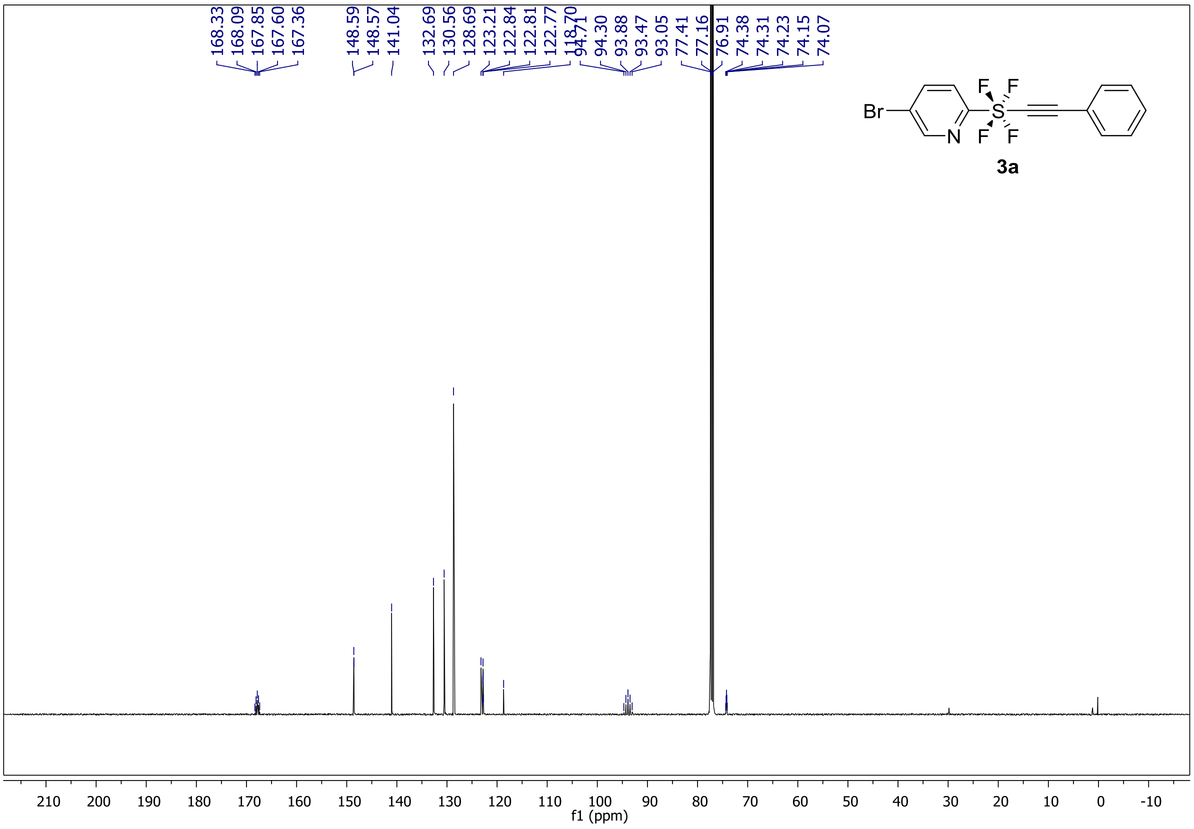
168.33
168.09
167.85
167.60
167.36
148.59
148.57
141.04
132.69
130.56
128.69
123.21
122.84
122.81
122.77
118.70
94.71
94.30
93.88
93.47
93.05
77.41
77.16
76.91
74.38
74.31
74.23
74.15
74.07
Br
N
F
S
3a
f1 (ppm)

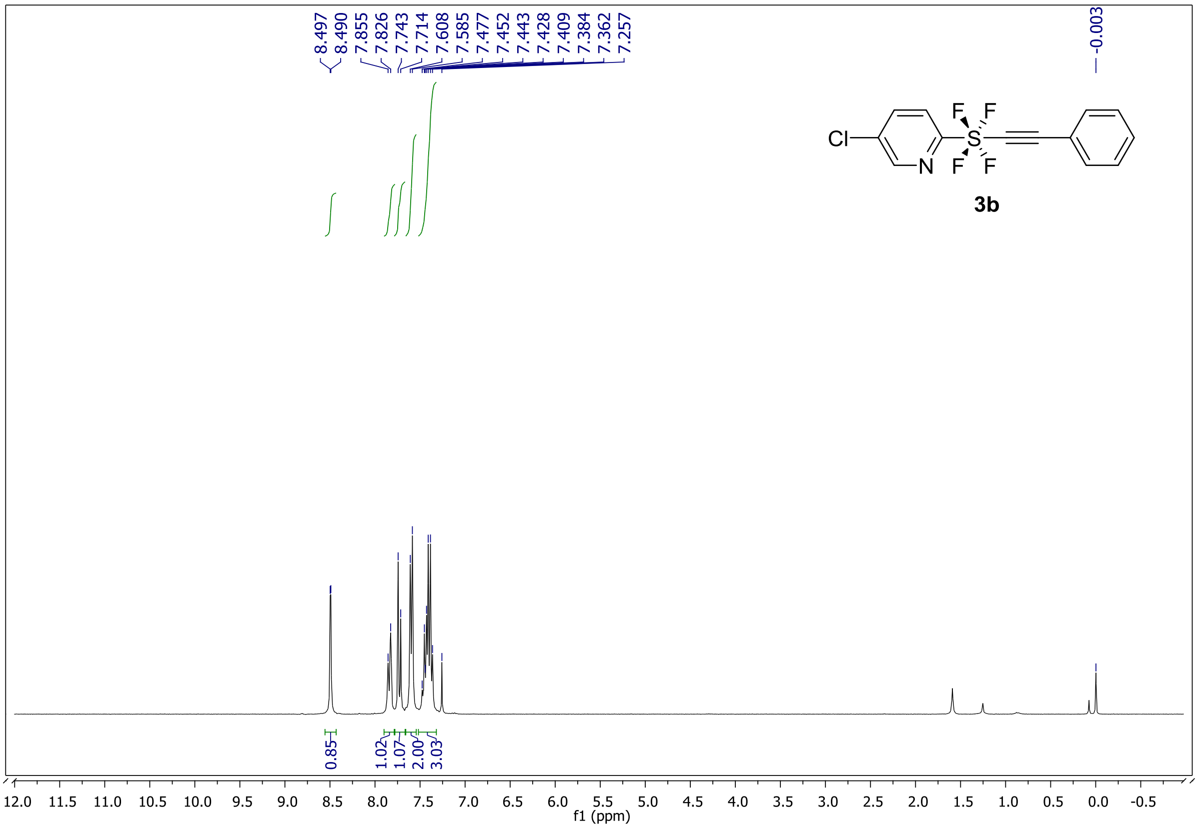
8.497
8.490
7.855
7.826
7.743
7.714
7.608
7.585
7.477
7.452
7.443
7.428
7.409
7.384
7.362
7.257
-0.003
Cl
N
F
S
3b
0.85
1.02
1.07
2.00
3.03
f1 (ppm)

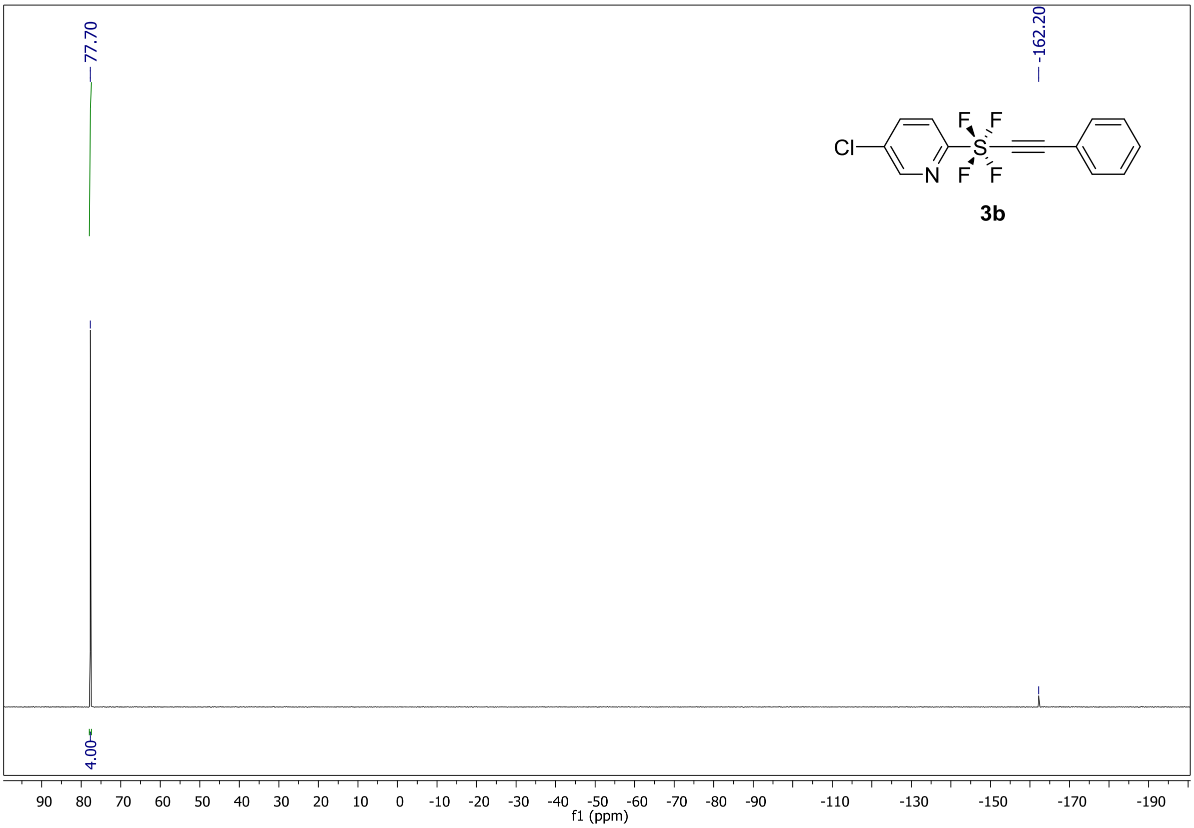
-77.70
-162.20
Cl
N
F
F
S
F
F
3b
4.00
90 80 70 60 50 40 30 20 10 0 -10 -20 -30 -40 -50 -60 -70 -80 -90 -110 -130 -150 -170 -190
f1 (ppm)

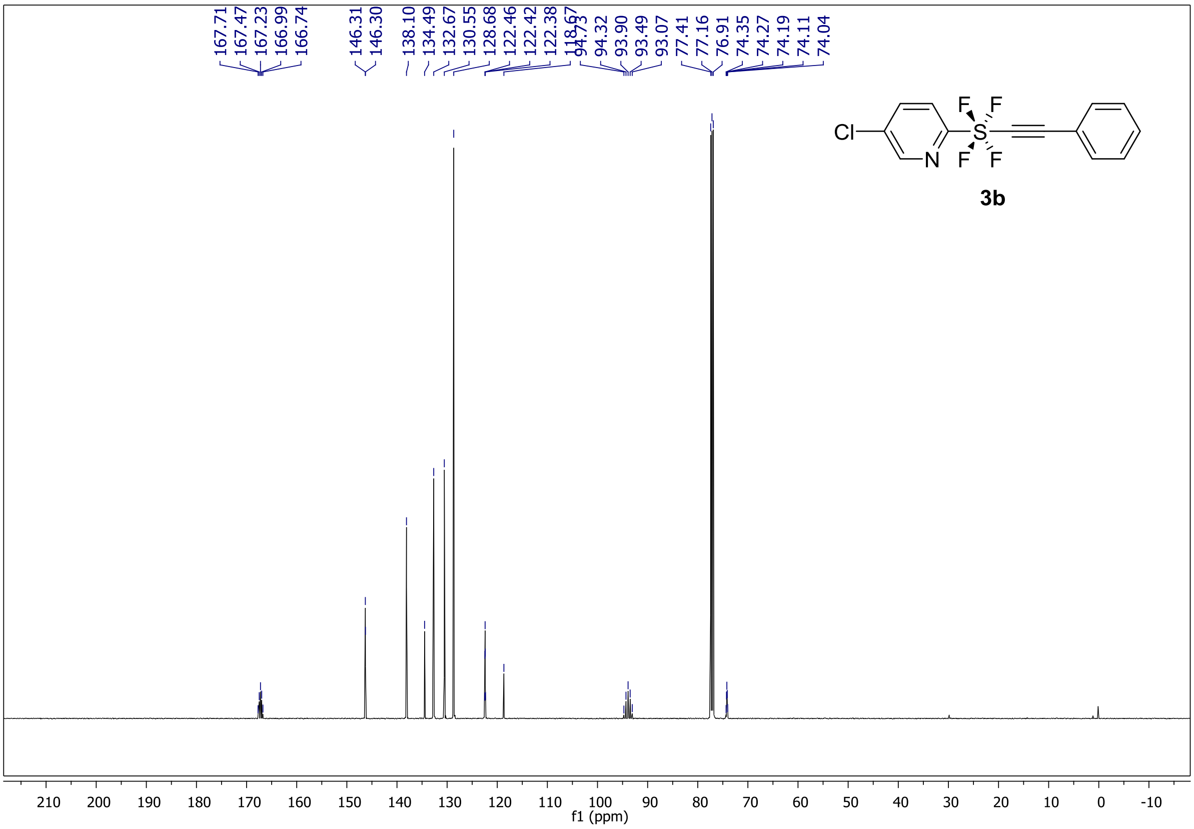
167.71
167.47
167.23
166.99
166.74
146.31
146.30
138.10
134.49
132.67
130.55
128.68
122.46
122.42
122.38
118.67
94.73
94.32
93.90
93.49
93.07
77.41
77.16
76.91
74.35
74.27
74.19
74.11
74.04
Cl
N
F
F
S
F
F
3b
210 200 190 180 170 160 150 140 130 120 110 100 90 80 70 60 50 40 30 20 10 0 -10
f1 (ppm)

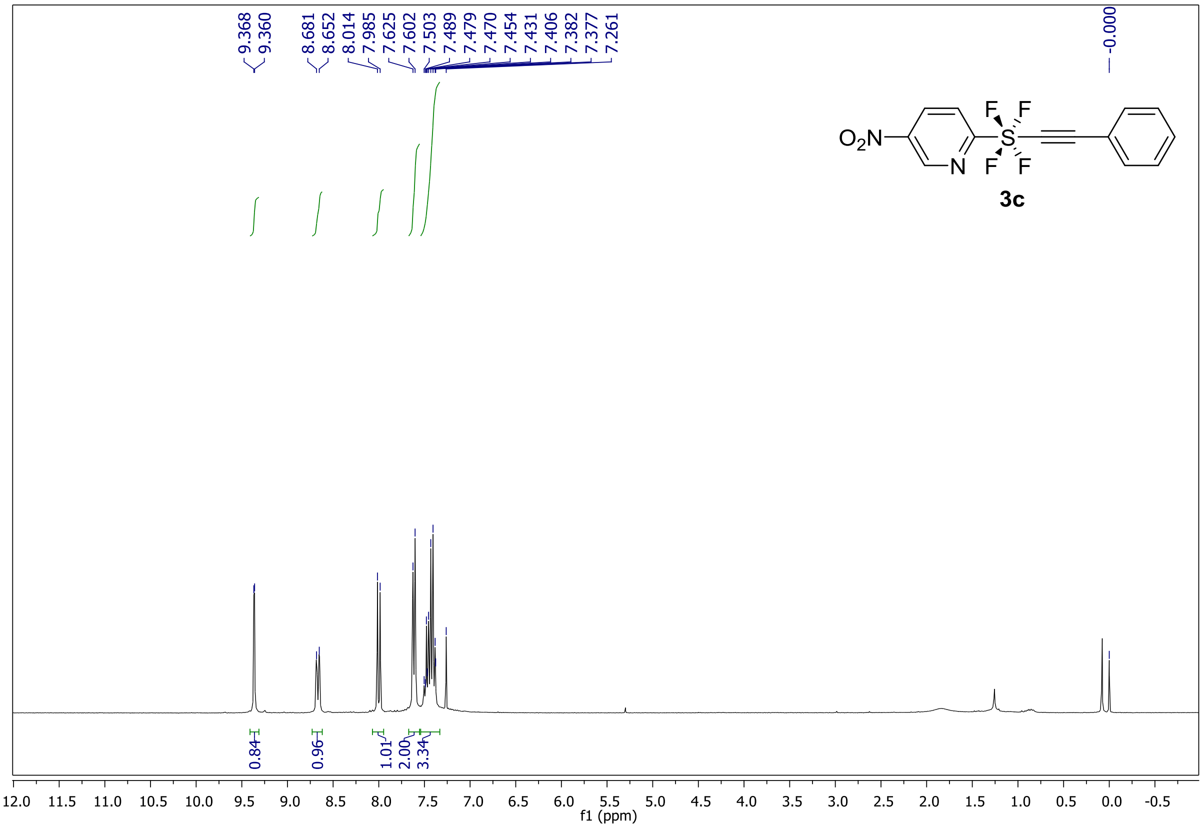

9.368
9.360
8.681
8.652
8.014
7.985
7.625
7.602
7.503
7.489
7.479
7.470
7.454
7.431
7.406
7.382
7.377
7.261
-0.000
O_2N
N
S
F
3c
0.84
0.96
1.01
2.00
3.34
f1 (ppm)

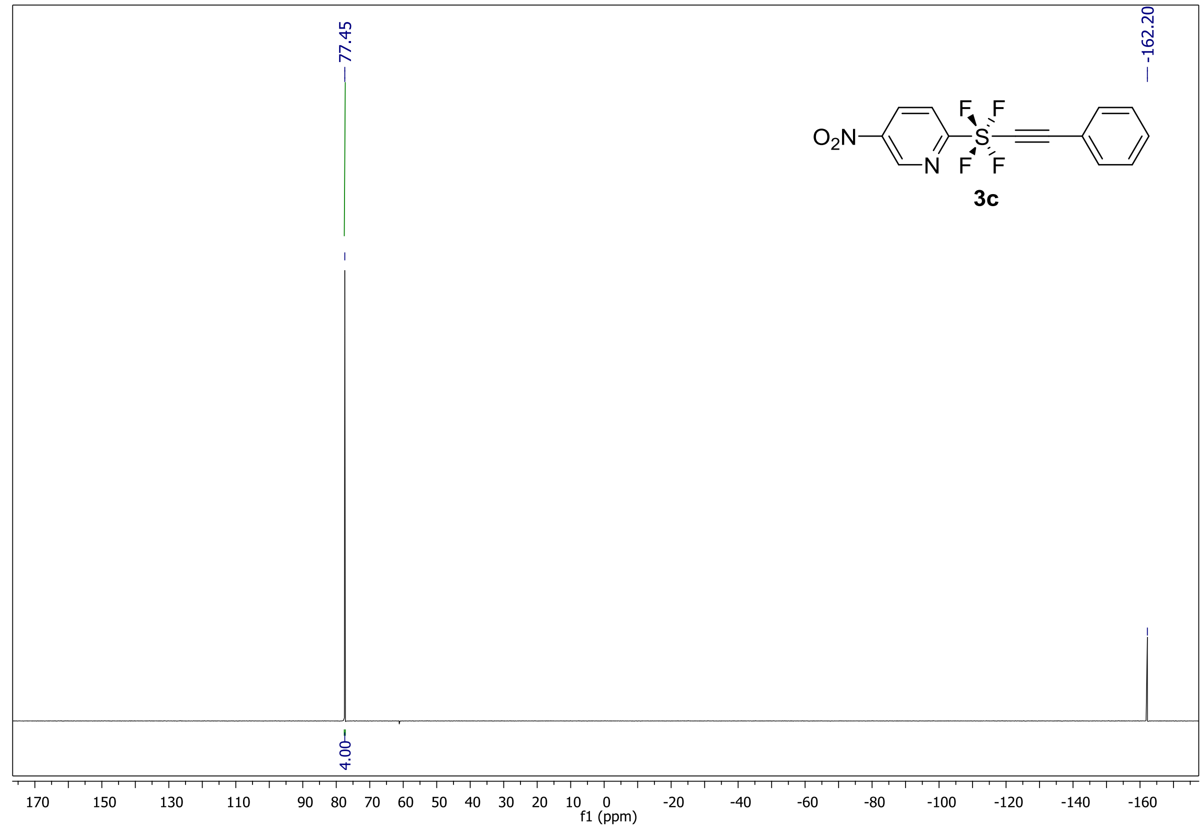

77.45
-162.20
O_2N
N
S
F
3c
4.00
f1 (ppm)

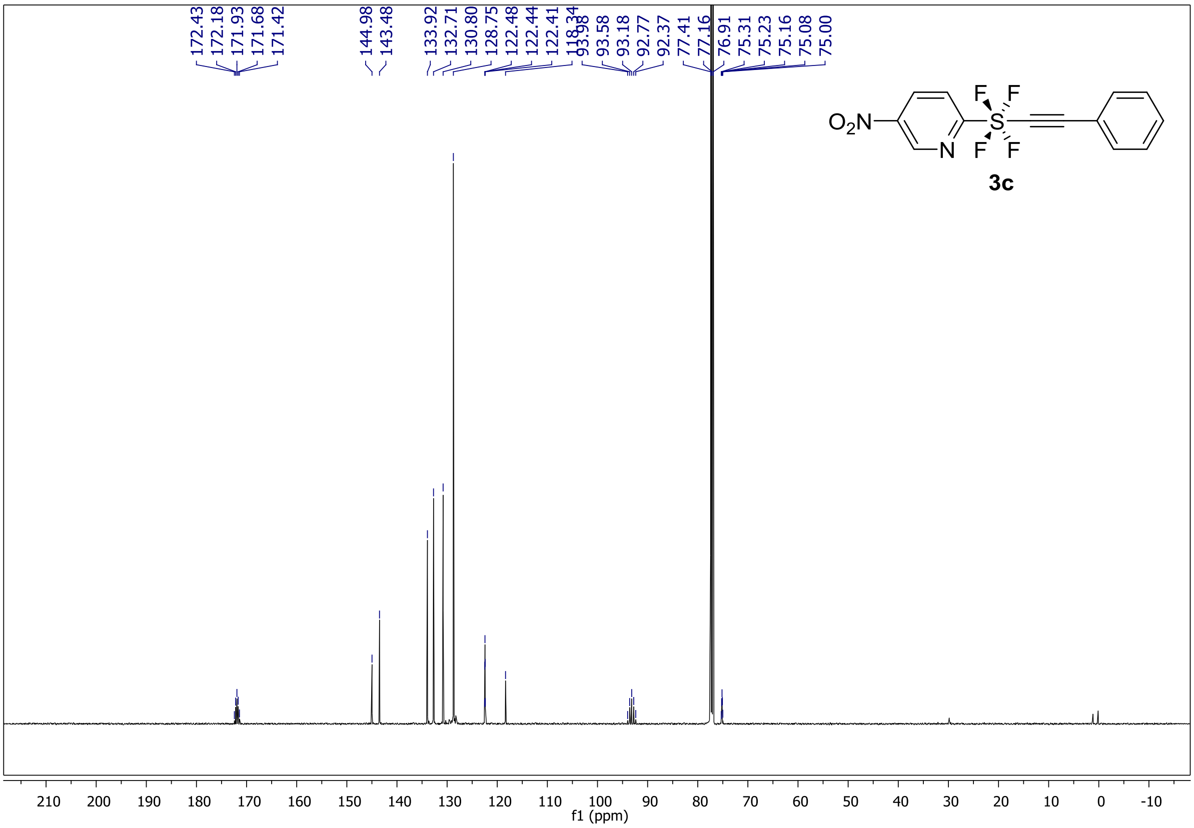
172.43
172.18
171.93
171.68
171.42
144.98
143.48
133.92
132.71
130.80
128.75
122.48
122.44
122.41
118.34
93.98
93.58
93.18
92.77
92.37
77.41
77.16
76.91
75.31
75.23
75.16
75.08
75.00
O2N
N
F
F
F
F
S
3c
f1 (ppm)

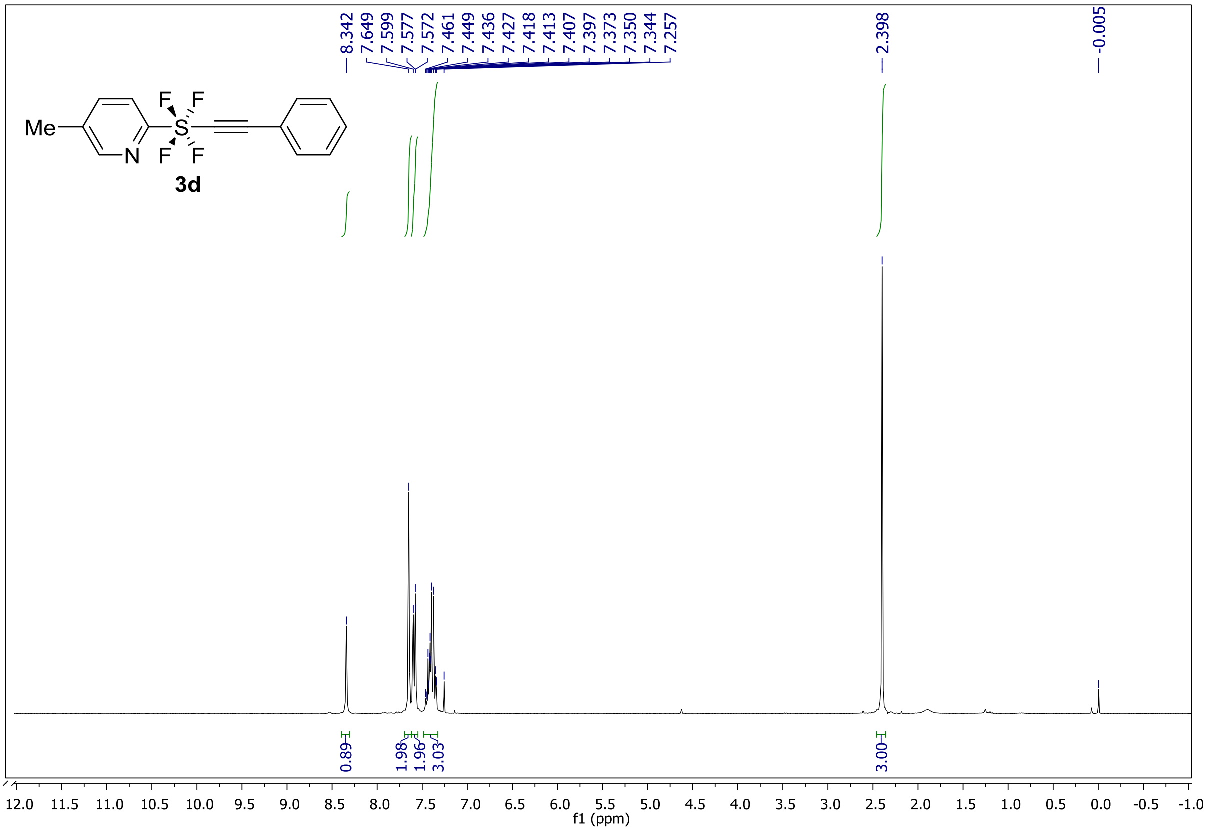
8.342
7.649
7.599
7.577
7.572
7.461
7.449
7.436
7.427
7.418
7.413
7.407
7.397
7.373
7.350
7.344
7.257
2.398
-0.005
Me
N
F
F
F
F
S
3d
0.89
1.98
1.96
3.03
3.00
f1 (ppm)

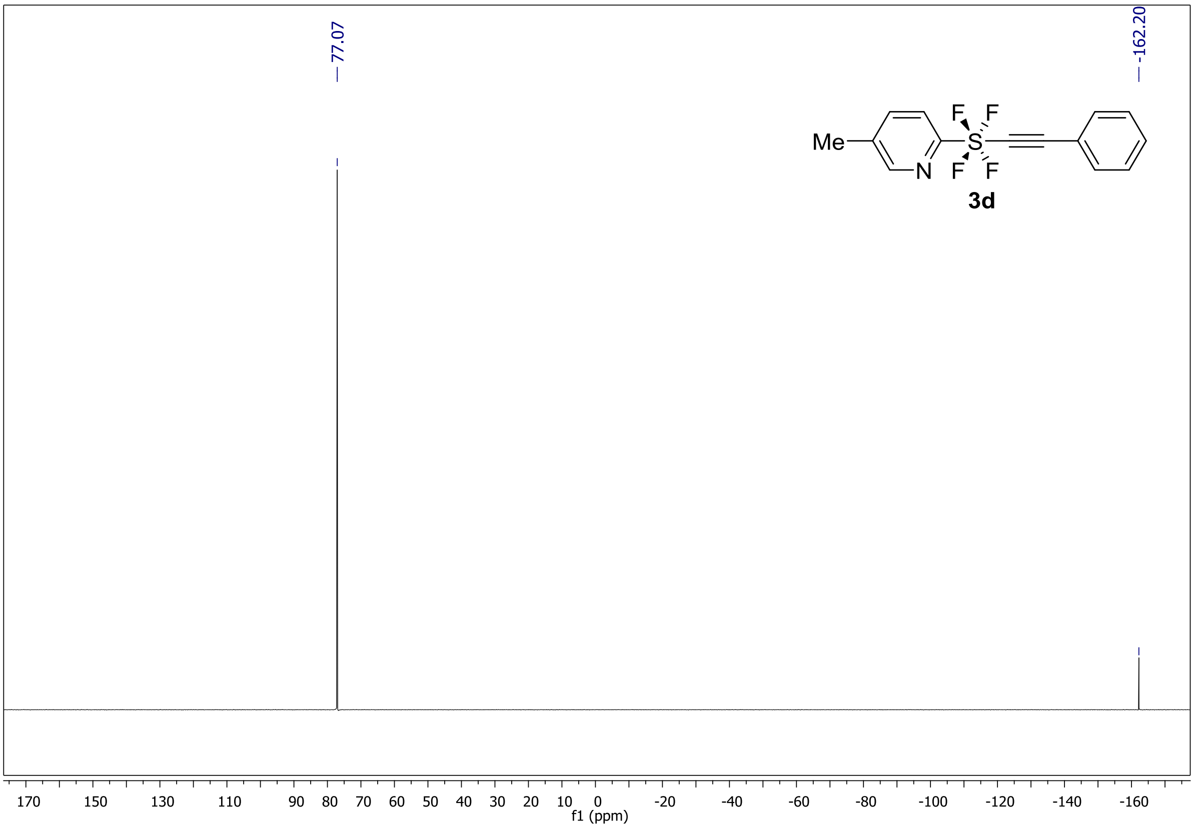
77.07
-162.20
Me
N
F
S
3d
f1 (ppm)

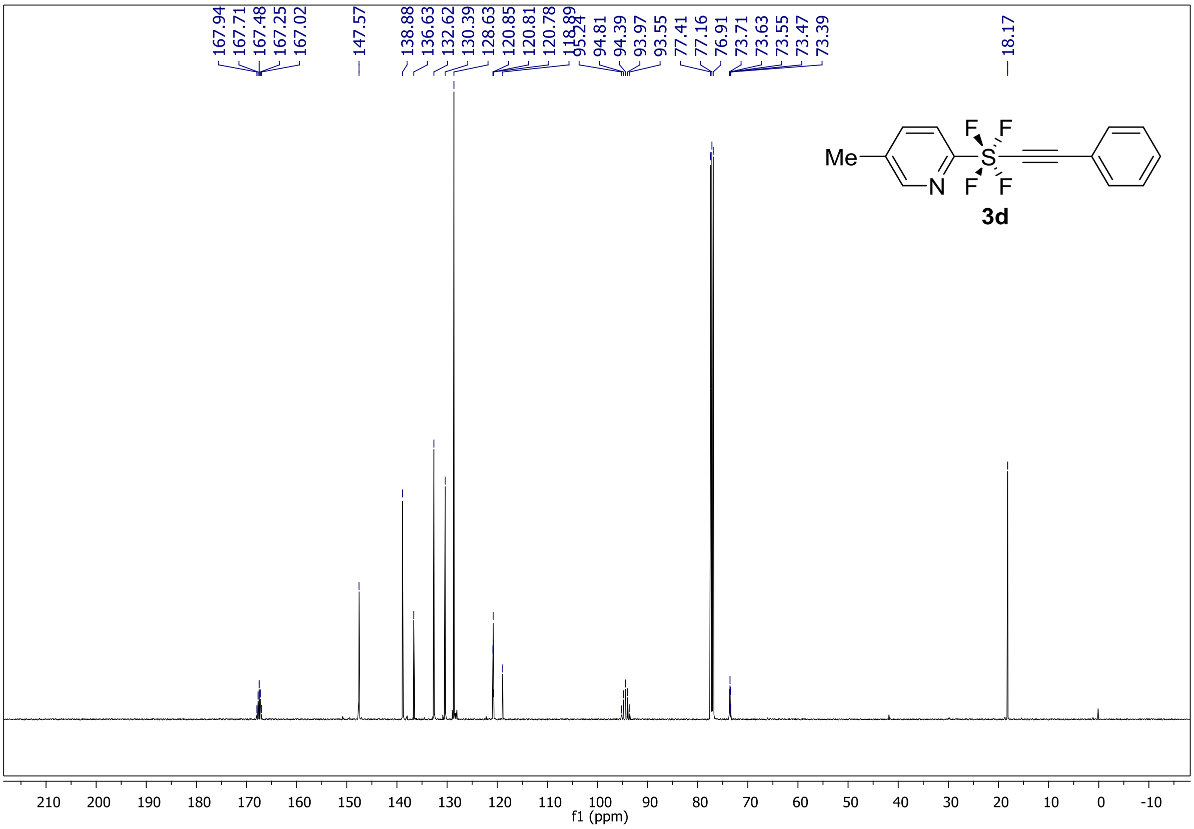
167.94
167.71
167.48
167.25
167.02
147.57
138.88
136.63
132.62
130.39
128.63
120.85
120.81
120.78
118.89
95.24
94.81
94.39
93.97
93.55
77.41
77.16
76.91
73.71
73.63
73.55
73.47
73.39
18.17
Me
N
F
S
3d
f1 (ppm)

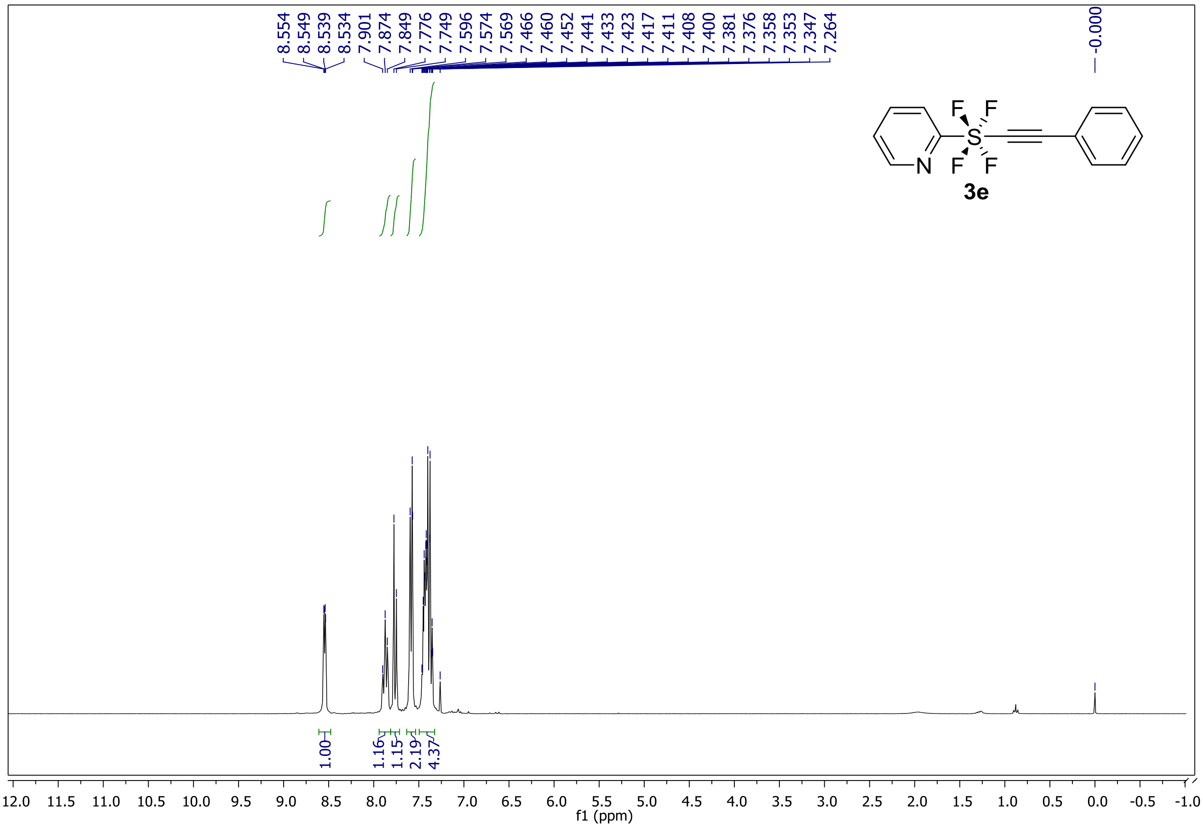
8.554
8.549
8.539
8.534
7.901
7.874
7.849
7.776
7.749
7.596
7.574
7.569
7.466
7.460
7.452
7.441
7.433
7.423
7.417
7.411
7.408
7.400
7.381
7.376
7.358
7.353
7.347
7.264
-0.000
F
F
S
F
F
N
3e
1.00
1.16
1.15
2.19
4.37
12.0 11.5 11.0 10.5 10.0 9.5 9.0 8.5 8.0 7.5 7.0 6.5 6.0 5.5 5.0 4.5 4.0 3.5 3.0 2.5 2.0 1.5 1.0 0.5 0.0 -0.5 -1.0
f1 (ppm)

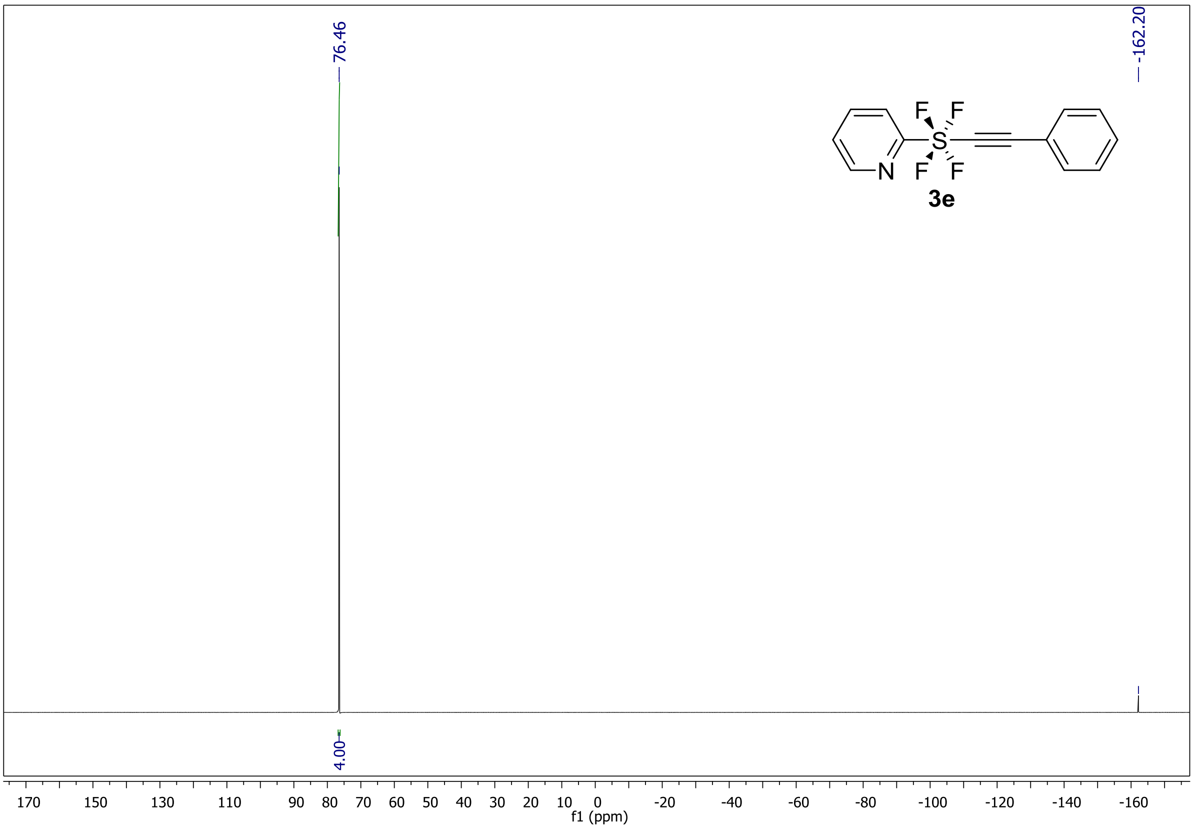
76.46
-162.20
F
F
S
F
F
N
3e
4.00
170 150 130 110 90 80 70 60 50 40 30 20 10 0 -20 -40 -60 -80 -100 -120 -140 -160
f1 (ppm)

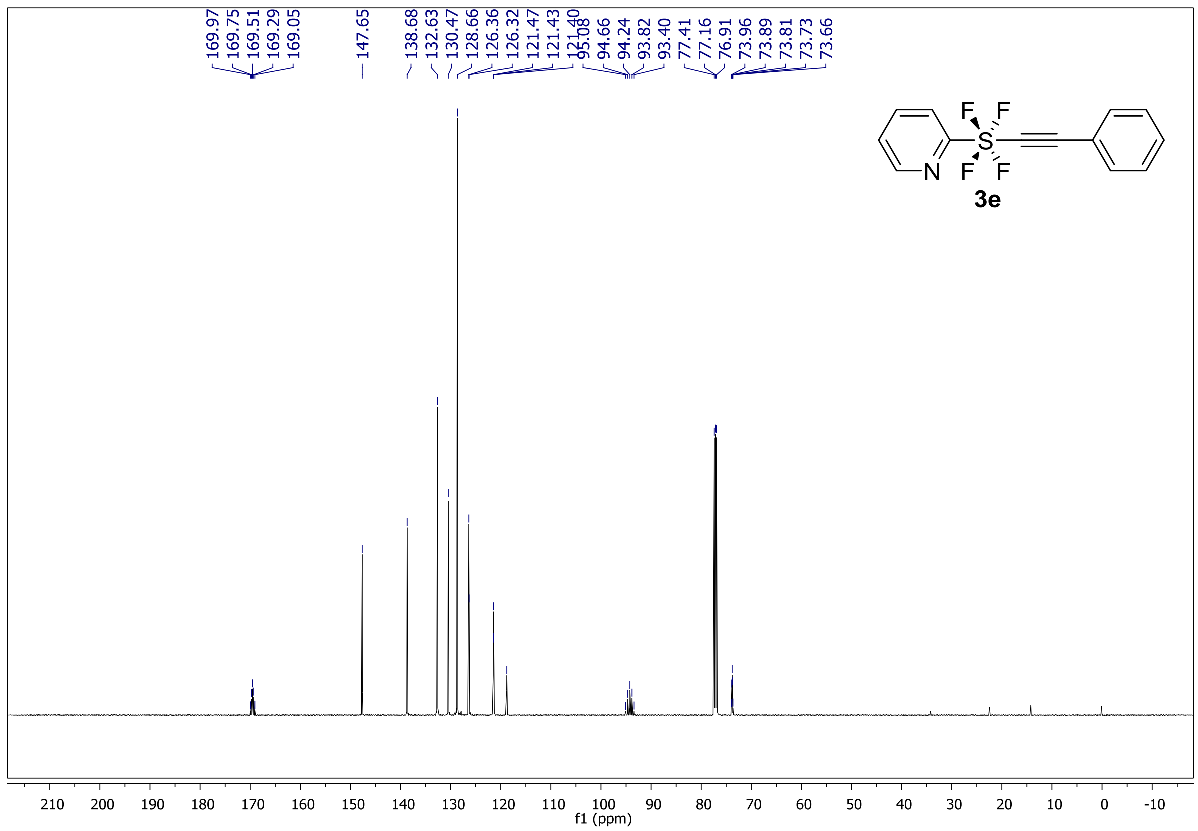
169.97
169.75
169.51
169.29
169.05
147.65
138.68
132.63
130.47
128.66
126.36
126.32
121.47
121.43
121.40
95.08
94.66
94.24
93.82
93.40
77.41
77.16
76.91
73.96
73.89
73.81
73.73
73.66
3e
210 200 190 180 170 160 150 140 130 120 110 100 90 80 70 60 50 40 30 20 10 0 -10
f1 (ppm)

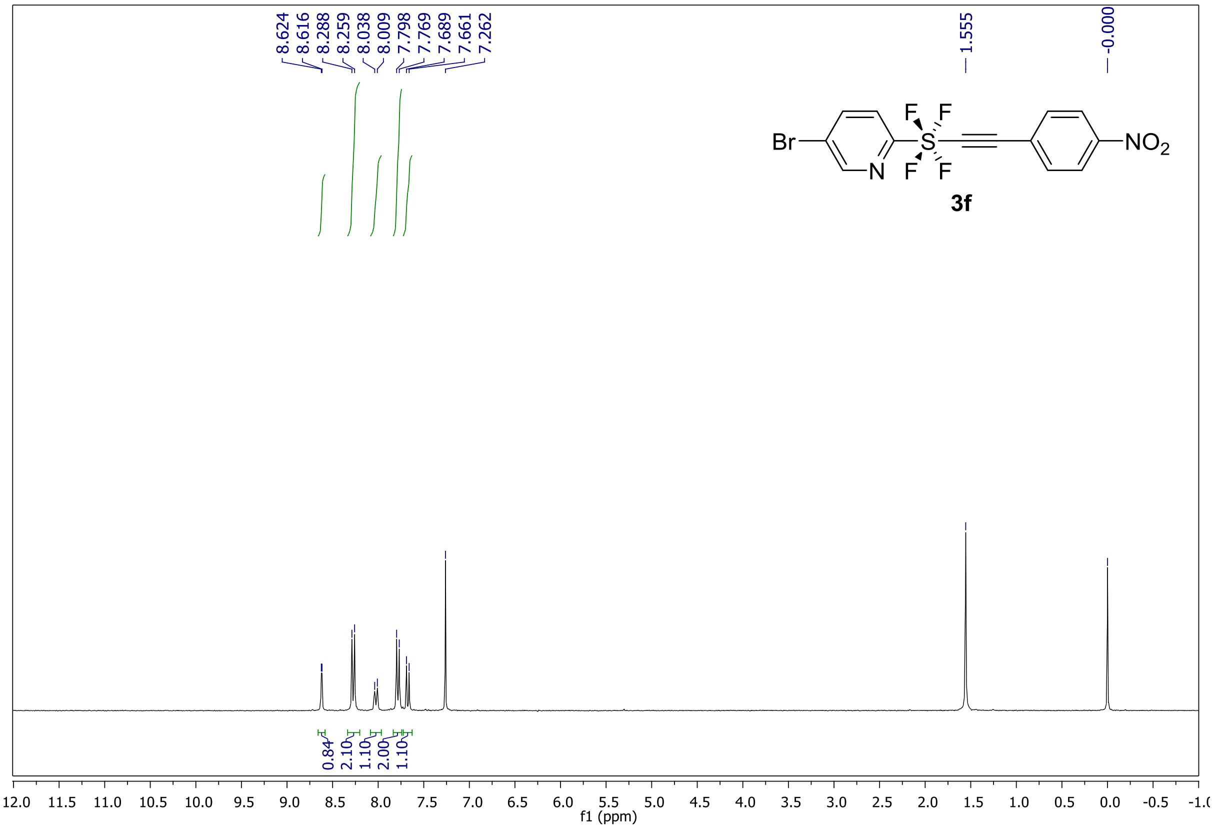
8.624
8.616
8.288
8.259
8.038
8.009
7.798
7.769
7.689
7.661
7.262
1.555
-0.000
3f
0.84
2.10
1.10
2.00
1.10
12.0 11.5 11.0 10.5 10.0 9.5 9.0 8.5 8.0 7.5 7.0 6.5 6.0 5.5 5.0 4.5 4.0 3.5 3.0 2.5 2.0 1.5 1.0 0.5 0.0 -0.5 -1.0
f1 (ppm)

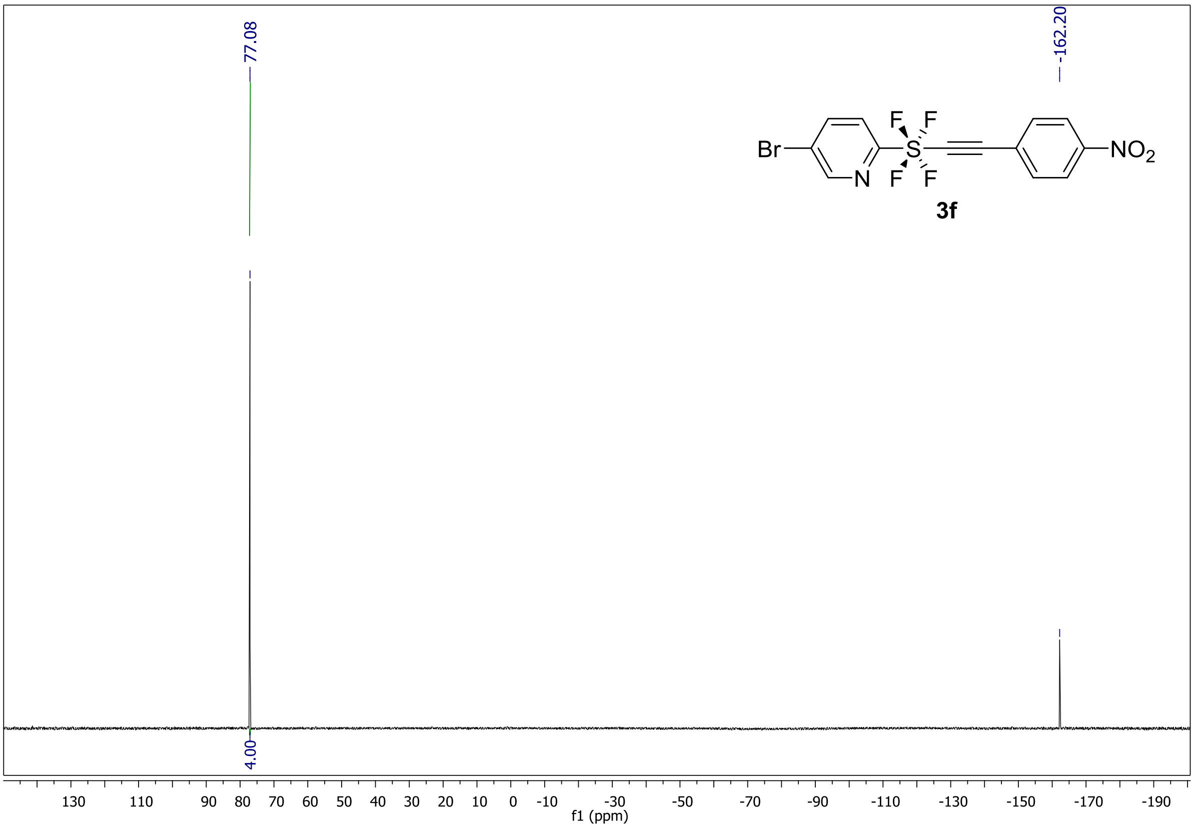
77.08
-162.20
Br
F
F
S
F
F
N
NO2
3f
4.00
130
110
90
80
70
60
50
40
30
20
10
0
-10
-30
-50
-70
-90
-110
-130
-150
-170
-190
f1 (ppm)

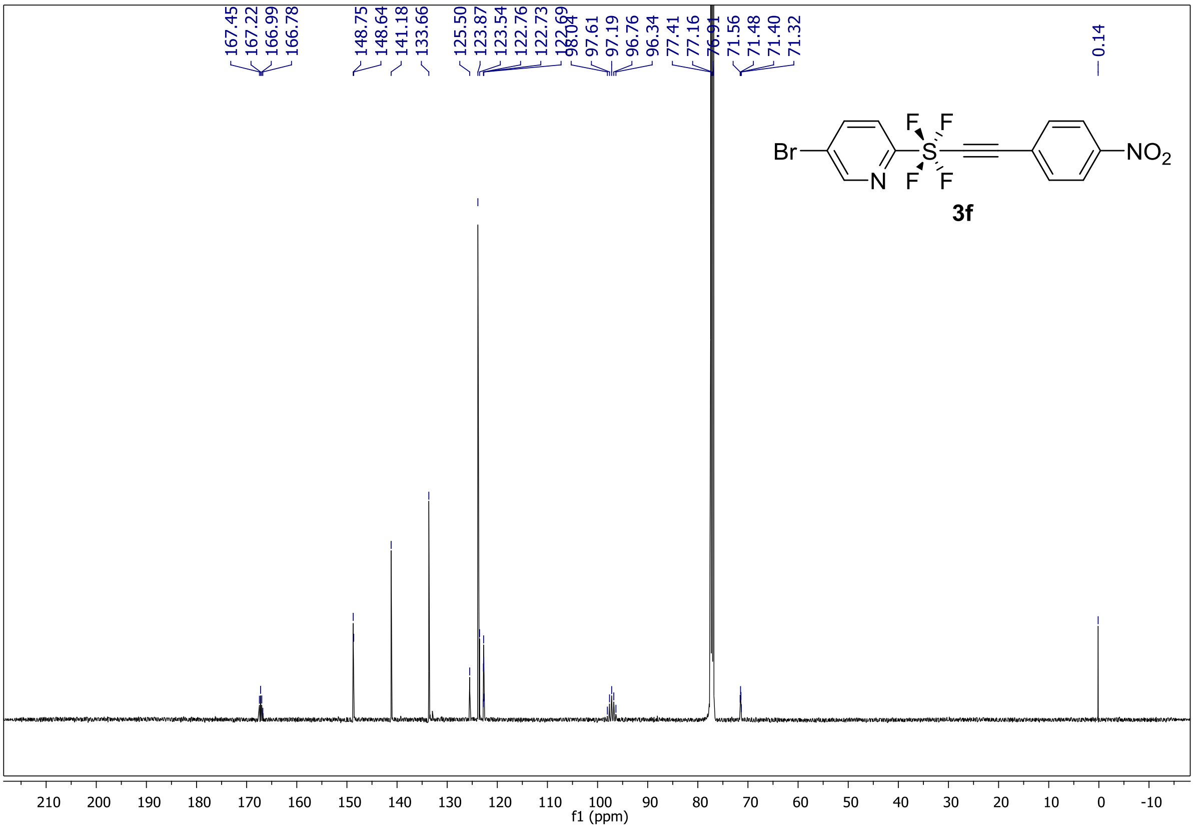
167.45
167.22
166.99
166.78
148.75
148.64
141.18
133.66
125.50
123.87
123.54
122.76
122.73
122.69
98.04
97.61
97.19
96.76
96.34
77.41
77.16
76.91
71.56
71.48
71.40
71.32
0.14
Br
F
F
S
F
F
N
NO2
3f
210
200
190
180
170
160
150
140
130
120
110
100
90
80
70
60
50
40
30
20
10
0
-10
f1 (ppm)

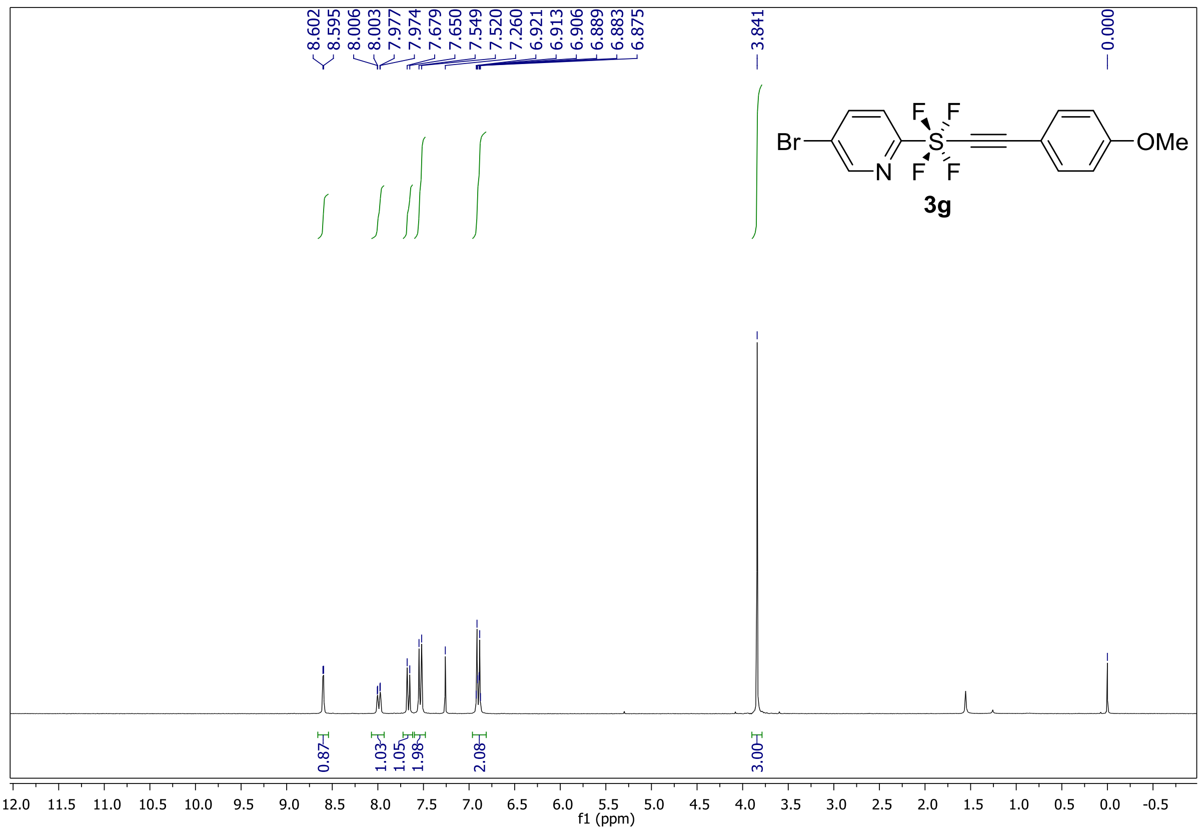
8.602
8.595
8.006
8.003
7.977
7.974
7.679
7.650
7.549
7.520
7.260
6.921
6.913
6.906
6.889
6.883
6.875
3.841
0.000
Br
N
F
F
F
F
S
OMe
3g
0.87
1.03
1.05
1.98
2.08
3.00
12.0 11.5 11.0 10.5 10.0 9.5 9.0 8.5 8.0 7.5 7.0 6.5 6.0 5.5 5.0 4.5 4.0 3.5 3.0 2.5 2.0 1.5 1.0 0.5 0.0 -0.5
f1 (ppm)

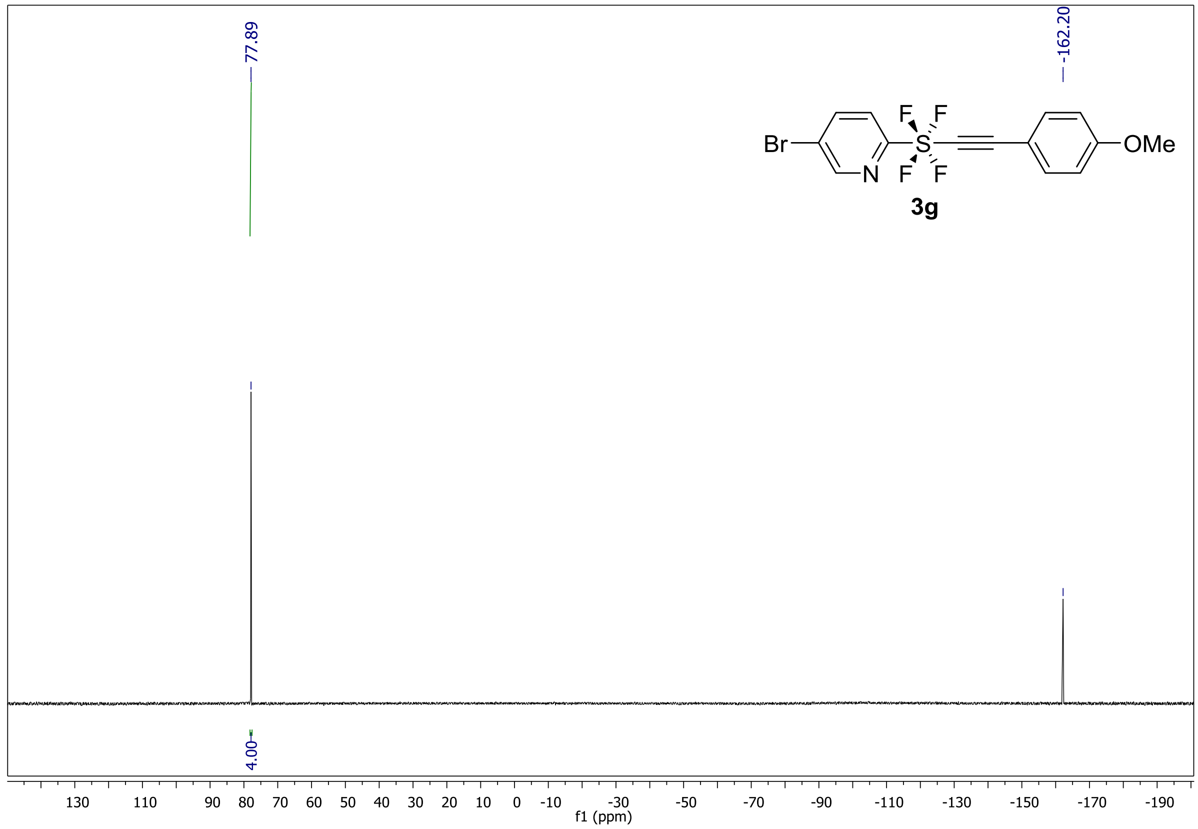
77.89
-162.20
Br
N
F
F
F
F
S
OMe
3g
4.00
130 110 90 80 70 60 50 40 30 20 10 0 -10 -30 -50 -70 -90 -110 -130 -150 -170 -190
f1 (ppm)

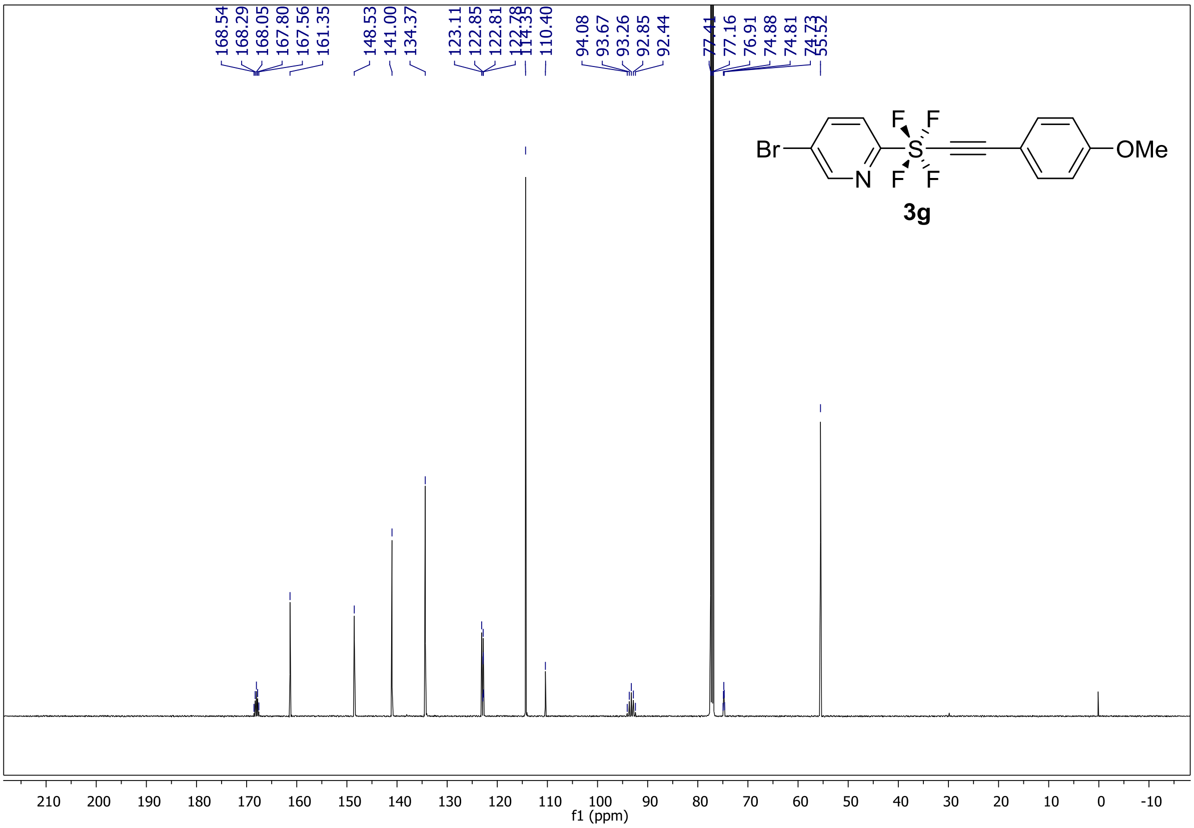

168.54
168.29
168.05
167.80
167.56
161.35
148.53
141.00
134.37
123.11
122.85
122.81
114.35
110.40
94.08
93.67
93.26
92.85
92.44
77.16
76.91
74.88
74.81
55.52
Br
N
F
S
OMe
3g
f1 (ppm)

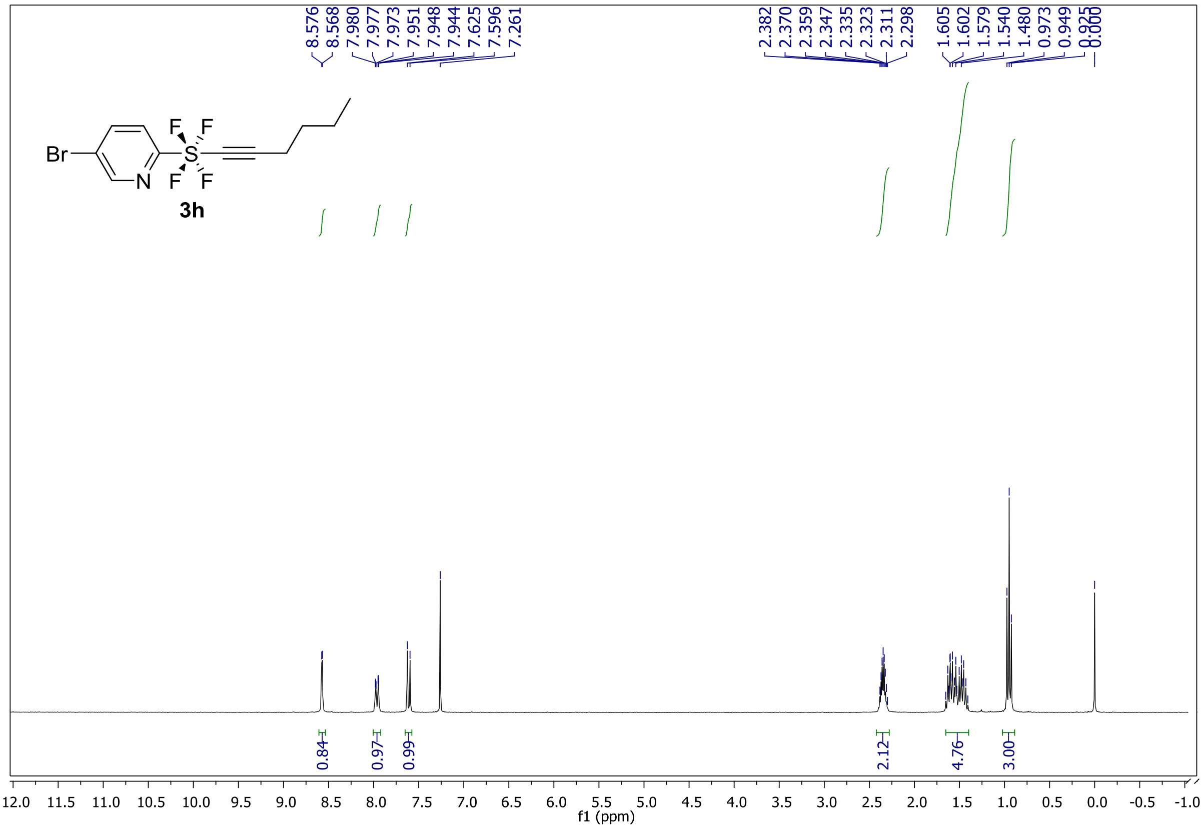

8.576
8.568
7.980
7.977
7.973
7.951
7.948
7.944
7.625
7.596
7.261
2.382
2.370
2.359
2.347
2.335
2.323
2.311
2.298
1.605
1.602
1.579
1.540
1.480
0.973
0.949
0.000
Br
N
F
S
3h
0.84
0.97
0.99
2.12
4.76
3.00
f1 (ppm)

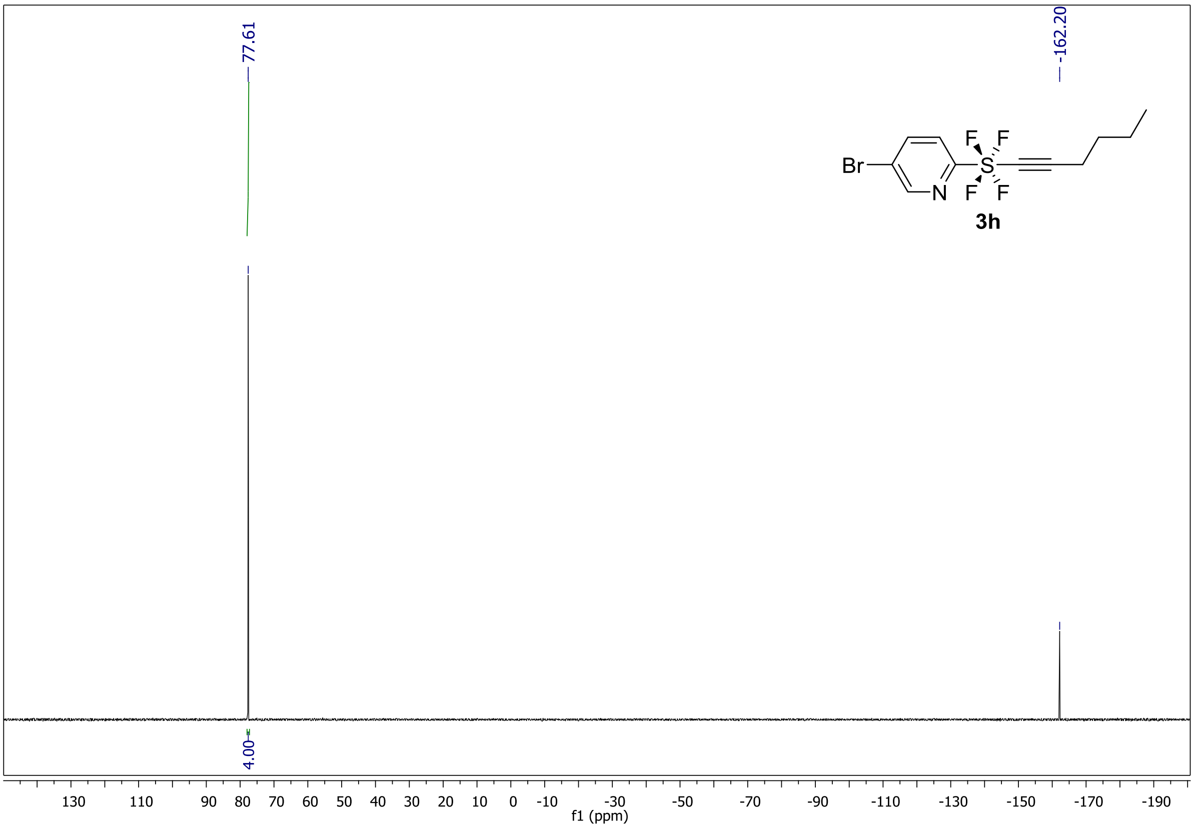
77.61
-162.20
Br
N
S
F
3h
4.00
130
110
90
80
70
60
50
40
30
20
10
0
-10
-30
-50
-70
-90
-110
-130
-150
-170
-190
f1 (ppm)

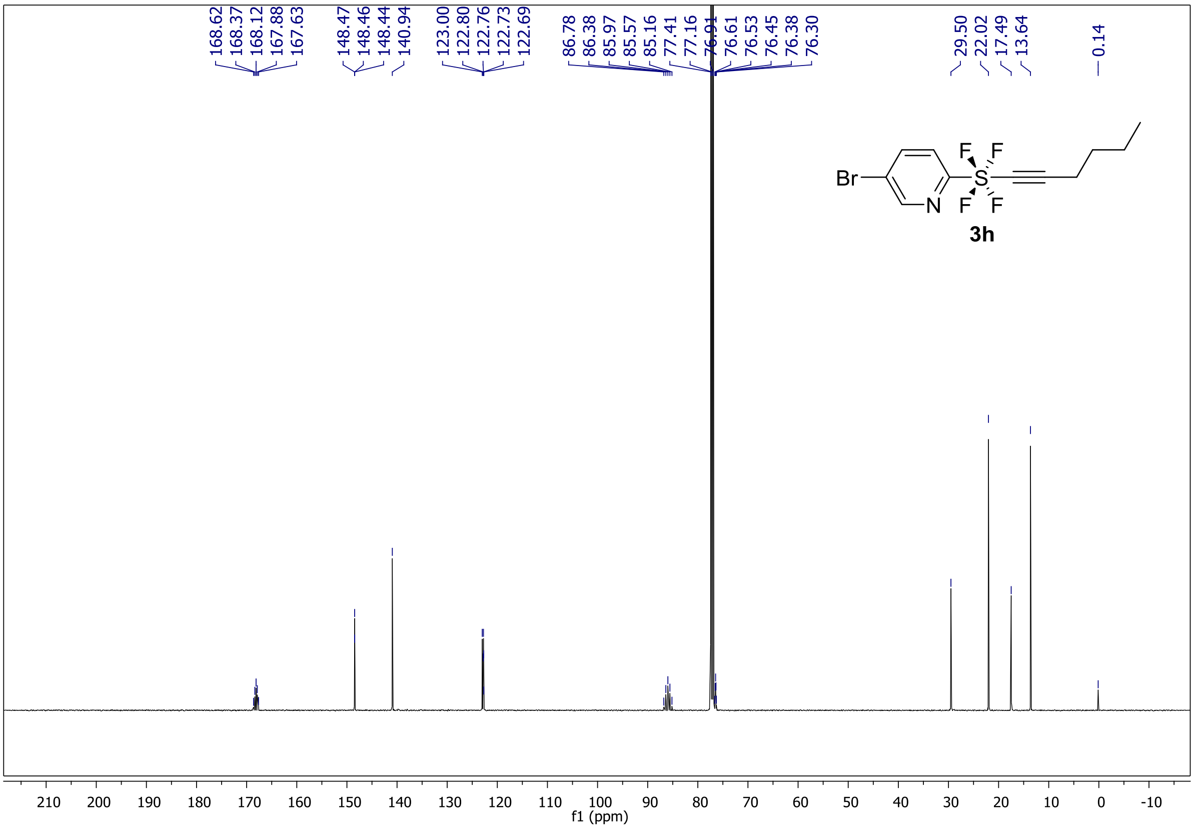
168.62
168.37
168.12
167.88
167.63
148.47
148.46
148.44
140.94
123.00
122.80
122.76
122.73
122.69
86.78
86.38
85.97
85.57
85.16
77.41
77.16
76.91
76.61
76.53
76.45
76.38
76.30
29.50
22.02
17.49
13.64
0.14
Br
N
S
F
3h
210
200
190
180
170
160
150
140
130
120
110
100
90
80
70
60
50
40
30
20
10
0
-10
f1 (ppm)

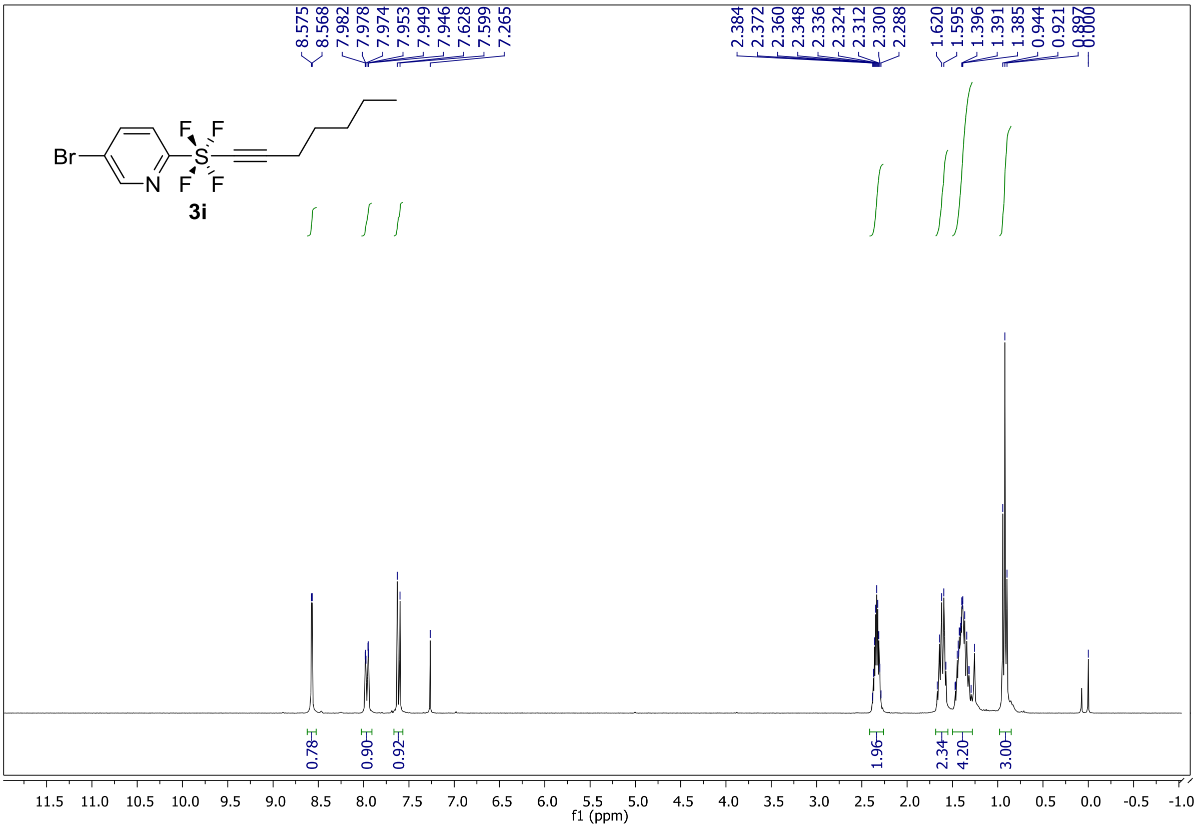
8.575
8.568
7.982
7.978
7.974
7.953
7.949
7.946
7.628
7.599
7.265
2.384
2.372
2.360
2.348
2.336
2.324
2.312
2.300
2.288
1.620
1.595
1.396
1.391
1.385
0.944
0.921
0.897
0.000
Br
N
S
F
F
F
F
3i
0.78
0.90
0.92
1.96
2.34
4.20
3.00
11.5 11.0 10.5 10.0 9.5 9.0 8.5 8.0 7.5 7.0 6.5 6.0 5.5 5.0 4.5 4.0 3.5 3.0 2.5 2.0 1.5 1.0 0.5 0.0 -0.5 -1.0
f1 (ppm)

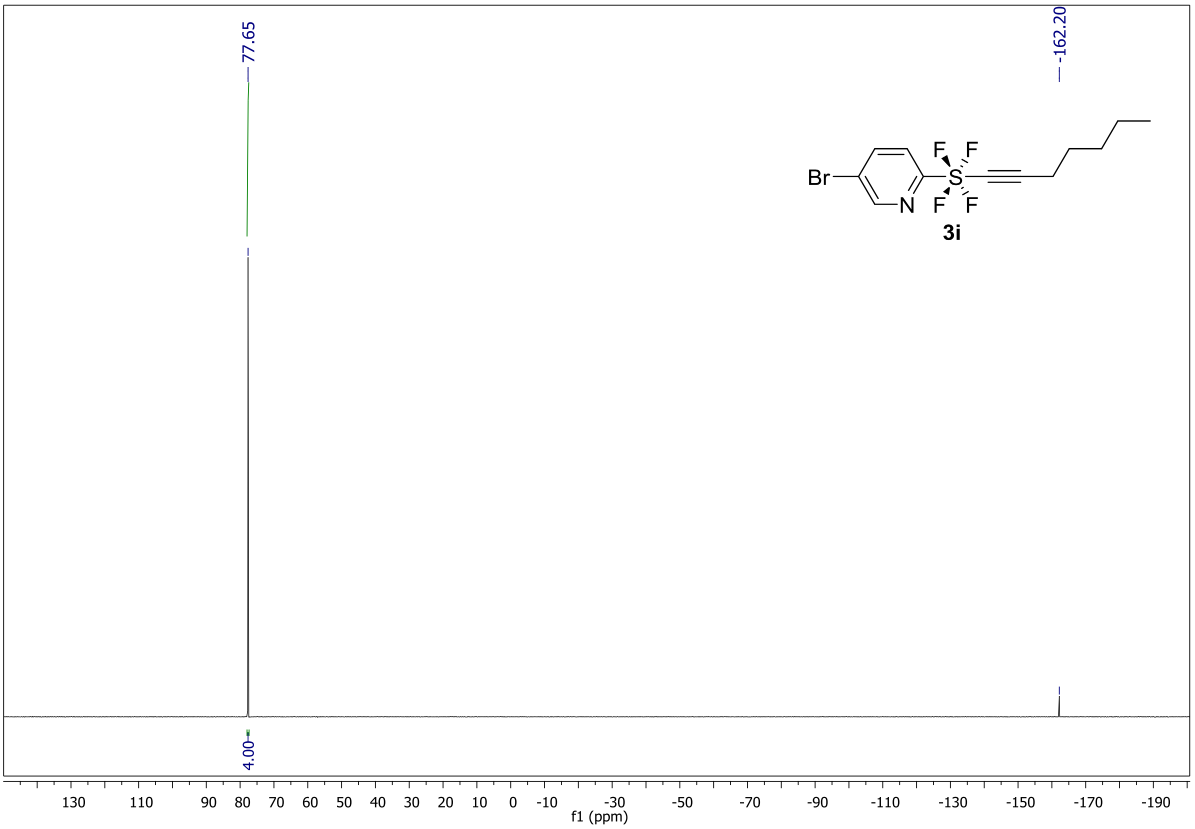
77.65
-162.20
Br
N
S
F
F
F
F
3i
4.00
130 110 90 80 70 60 50 40 30 20 10 0 -10 -30 -50 -70 -90 -110 -130 -150 -170 -190
f1 (ppm)

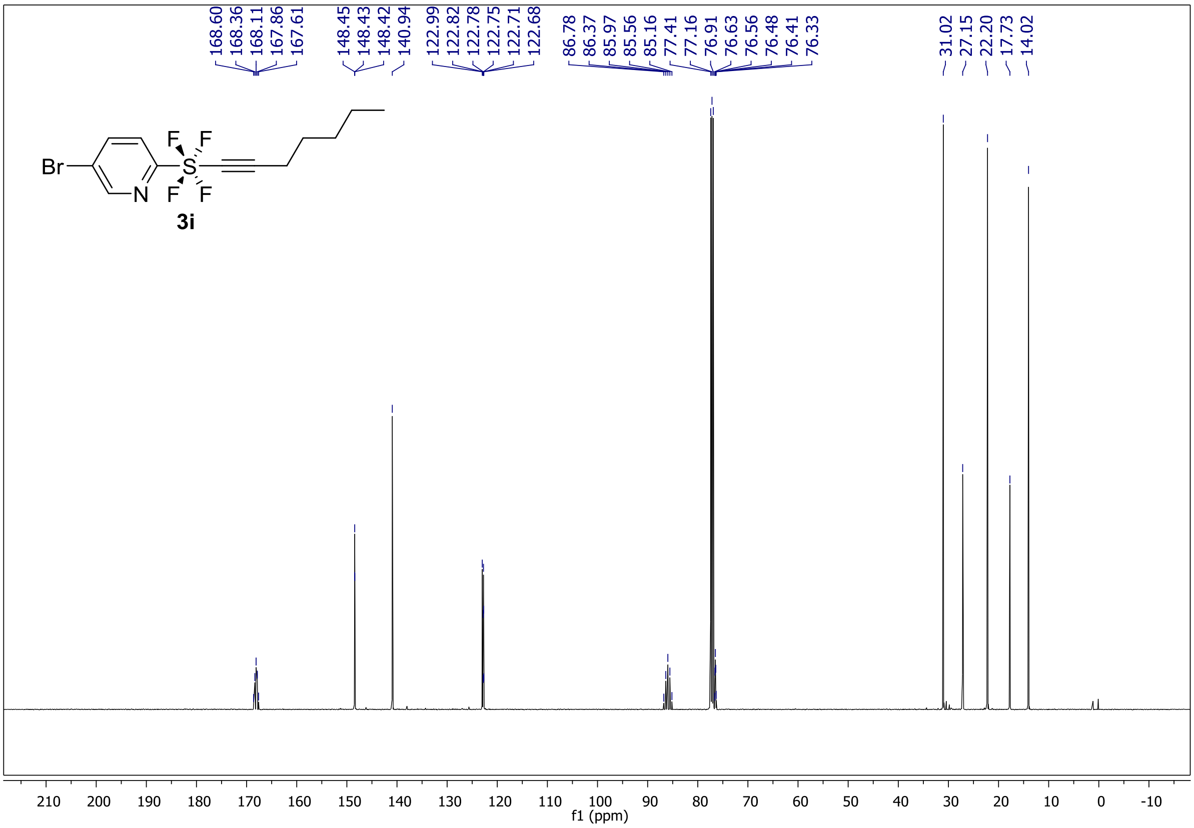
168.60
168.36
168.11
167.86
167.61
148.45
148.43
148.42
140.94
122.99
122.82
122.78
122.75
122.71
122.68
86.78
86.37
85.97
85.56
85.16
77.41
77.16
76.91
76.63
76.56
76.48
76.41
76.33
31.02
27.15
22.20
17.73
14.02
Br
F
F
S
F
F
N
3i
210 200 190 180 170 160 150 140 130 120 110 100 90 80 70 60 50 40 30 20 10 0 -10
f1 (ppm)

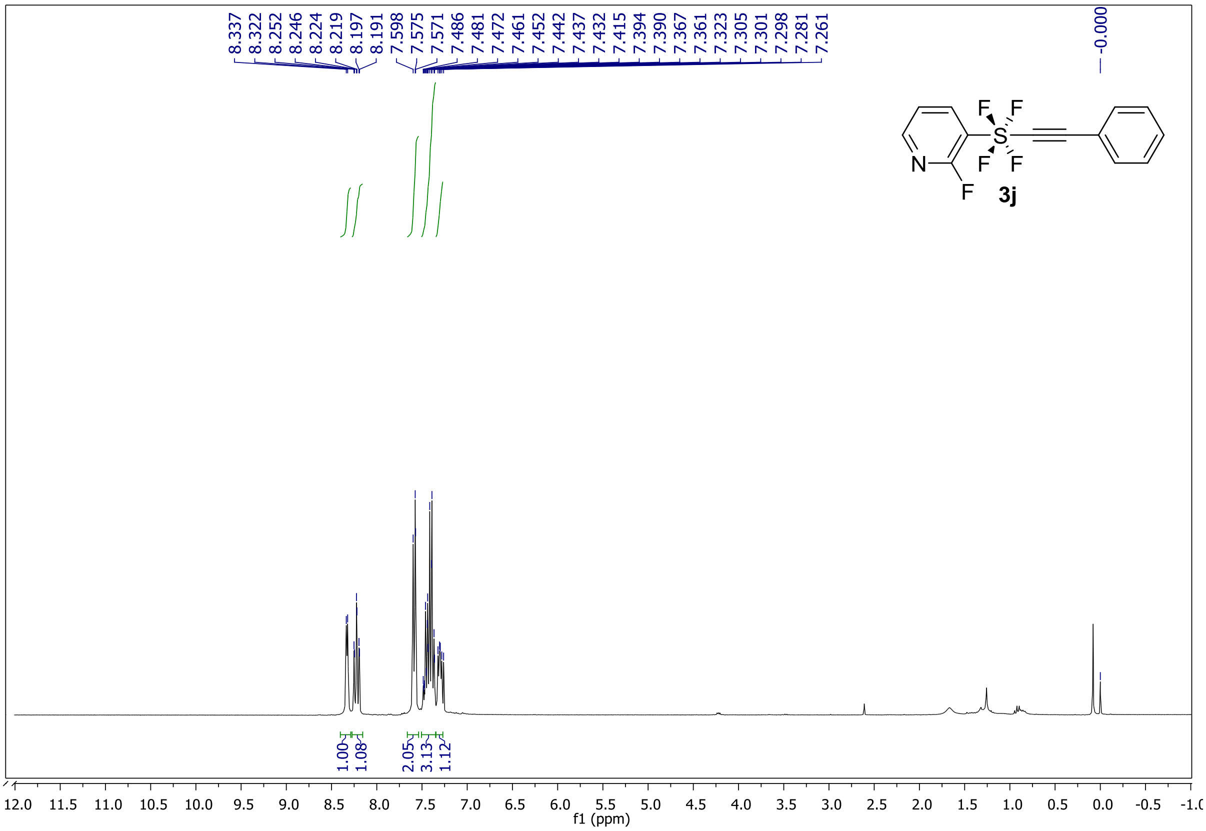
8.337
8.322
8.252
8.246
8.224
8.219
8.197
8.191
7.598
7.575
7.571
7.486
7.481
7.472
7.461
7.452
7.442
7.437
7.432
7.415
7.394
7.390
7.367
7.361
7.323
7.305
7.301
7.298
7.281
7.261
-0.000
F
F
S
F
F
N
F
3j
1.00
1.08
2.05
3.13
1.12
12.0 11.5 11.0 10.5 10.0 9.5 9.0 8.5 8.0 7.5 7.0 6.5 6.0 5.5 5.0 4.5 4.0 3.5 3.0 2.5 2.0 1.5 1.0 0.5 0.0 -0.5 -1.0
f1 (ppm)

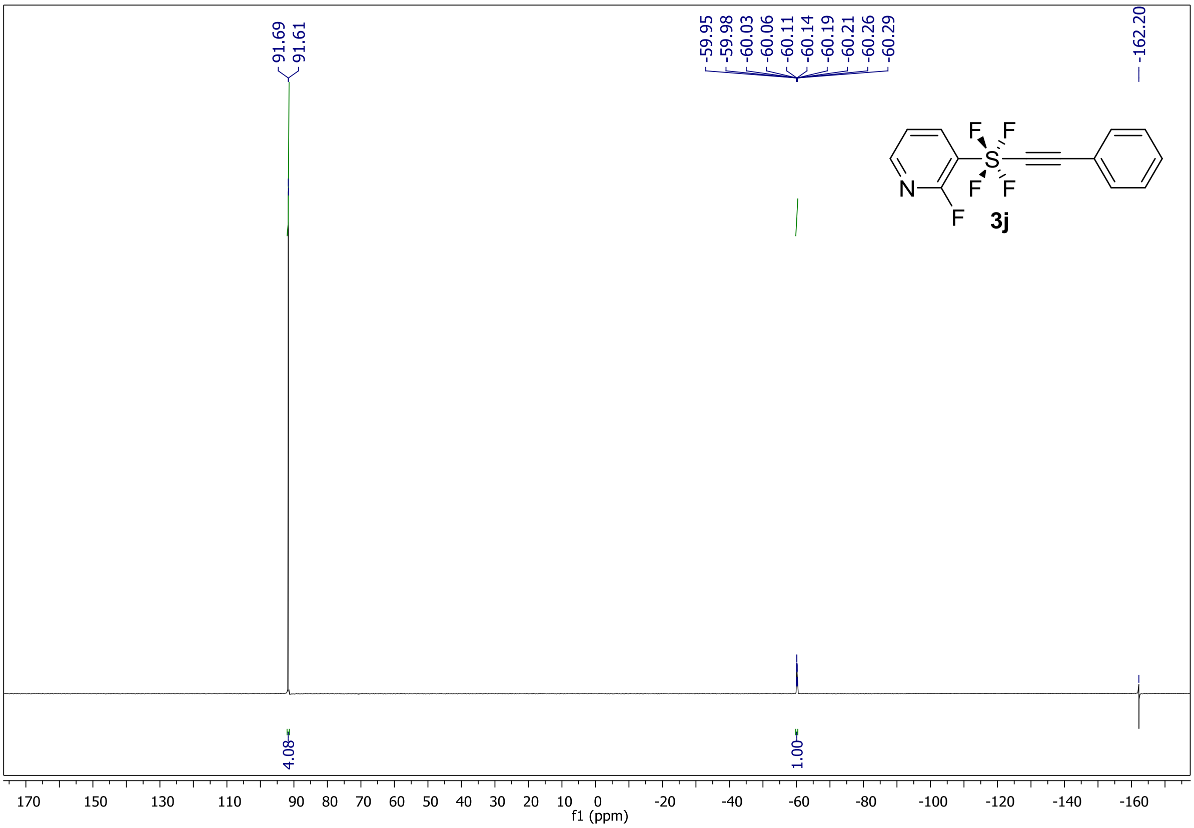
91.69
91.61
-59.95
-59.98
-60.03
-60.06
-60.11
-60.14
-60.19
-60.21
-60.26
-60.29
-162.20
F
F
S
F
F
N
F
3j
4.08
1.00
170 150 130 110 90 80 70 60 50 40 30 20 10 0 -20 -40 -60 -80 -100 -120 -140 -160
f1 (ppm)

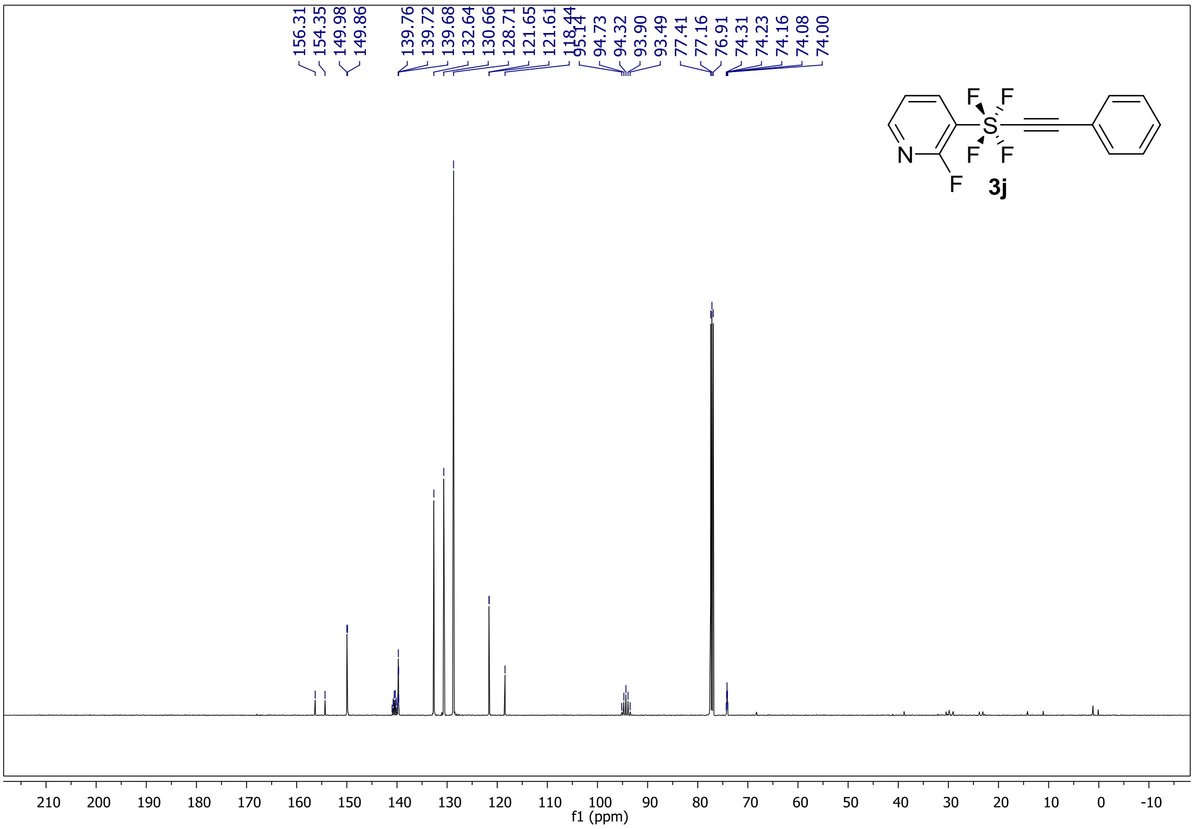
156.31
154.35
149.98
149.86
139.76
139.72
139.68
132.64
130.66
128.71
121.65
121.61
118.44
95.14
94.73
94.32
93.90
93.49
77.41
77.16
76.91
74.31
74.23
74.16
74.08
74.00
F
F
S
F
F
N
F
3j
210 200 190 180 170 160 150 140 130 120 110 100 90 80 70 60 50 40 30 20 10 0 -10
f1 (ppm)

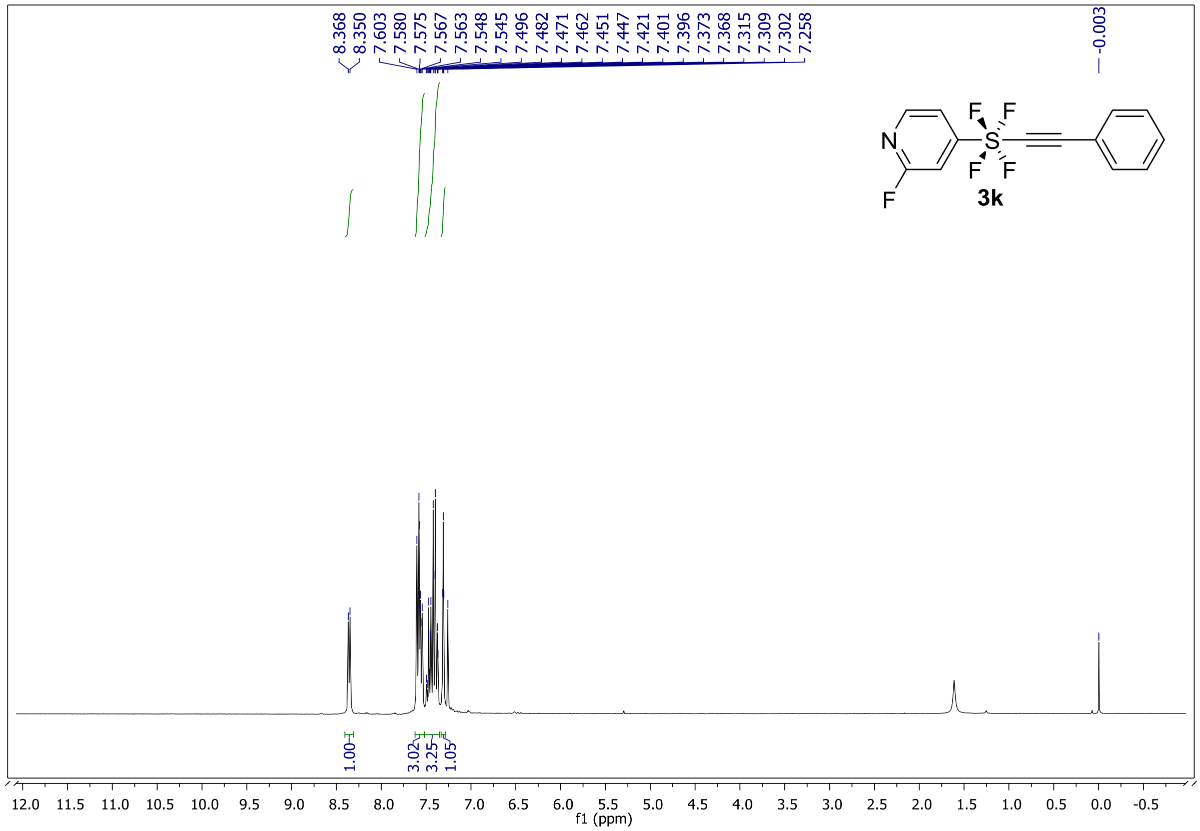
8.368
8.350
7.603
7.580
7.575
7.567
7.563
7.548
7.545
7.496
7.482
7.471
7.462
7.451
7.447
7.421
7.401
7.396
7.373
7.368
7.315
7.309
7.302
7.258
-0.003
3k
1.00
3.02
3.25
1.05
f1 (ppm)

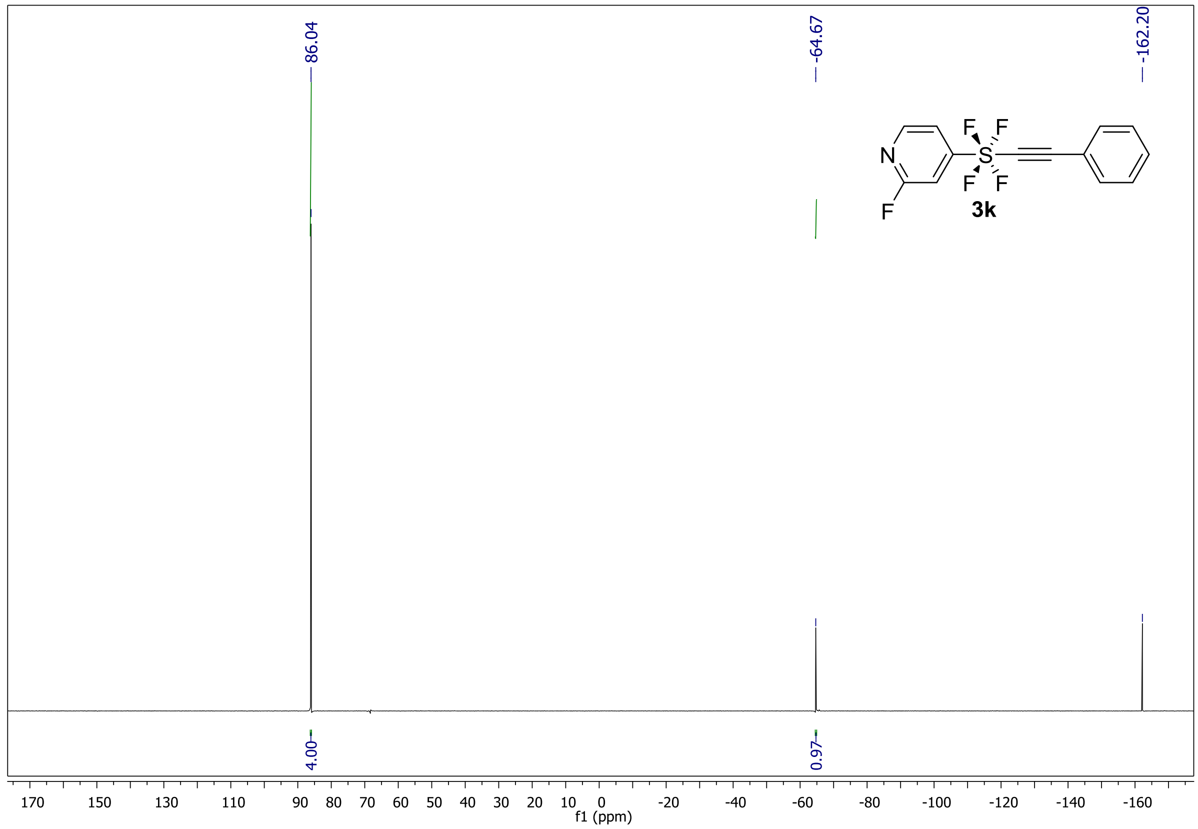
86.04
-64.67
-162.20
3k
4.00
0.97
f1 (ppm)

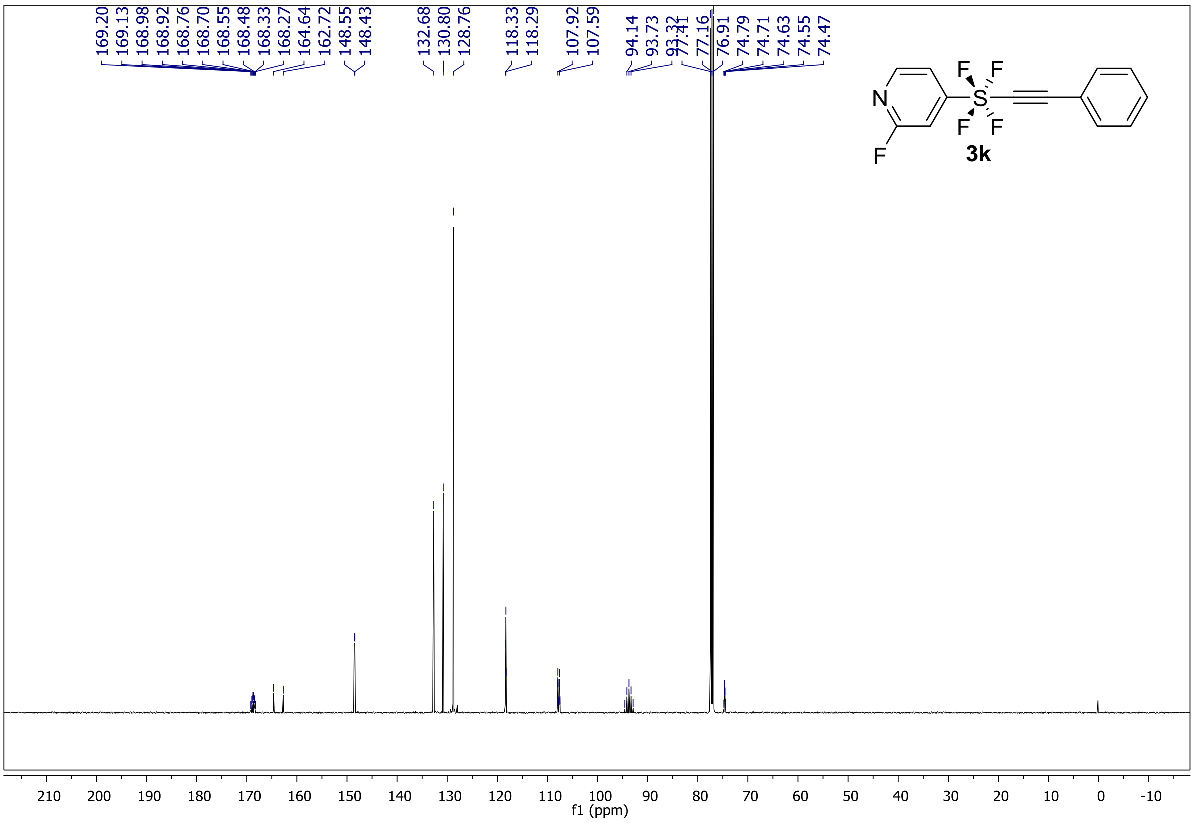
169.20
169.13
168.98
168.92
168.76
168.70
168.55
168.48
168.33
168.27
164.64
162.72
148.55
148.43
132.68
130.80
128.76
118.33
118.29
107.92
107.59
94.14
93.73
93.32
77.41
77.16
76.91
74.79
74.71
74.63
74.55
74.47
3k
210 200 190 180 170 160 150 140 130 120 110 100 90 80 70 60 50 40 30 20 10 0 -10
f1 (ppm)

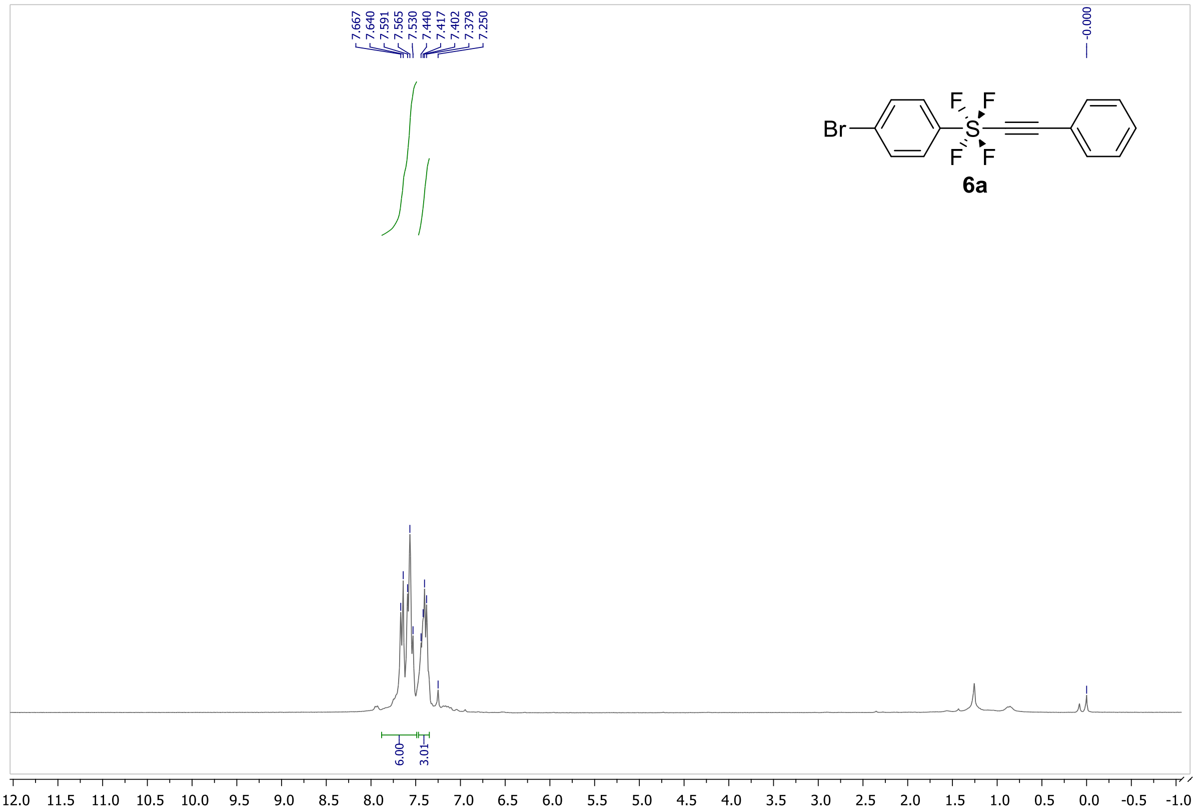
7.667
7.640
7.591
7.565
7.530
7.440
7.417
7.402
7.379
7.250
-0.000
Br
6a
6.00
3.01
12.0 11.5 11.0 10.5 10.0 9.5 9.0 8.5 8.0 7.5 7.0 6.5 6.0 5.5 5.0 4.5 4.0 3.5 3.0 2.5 2.0 1.5 1.0 0.5 0.0 -0.5 -1.0

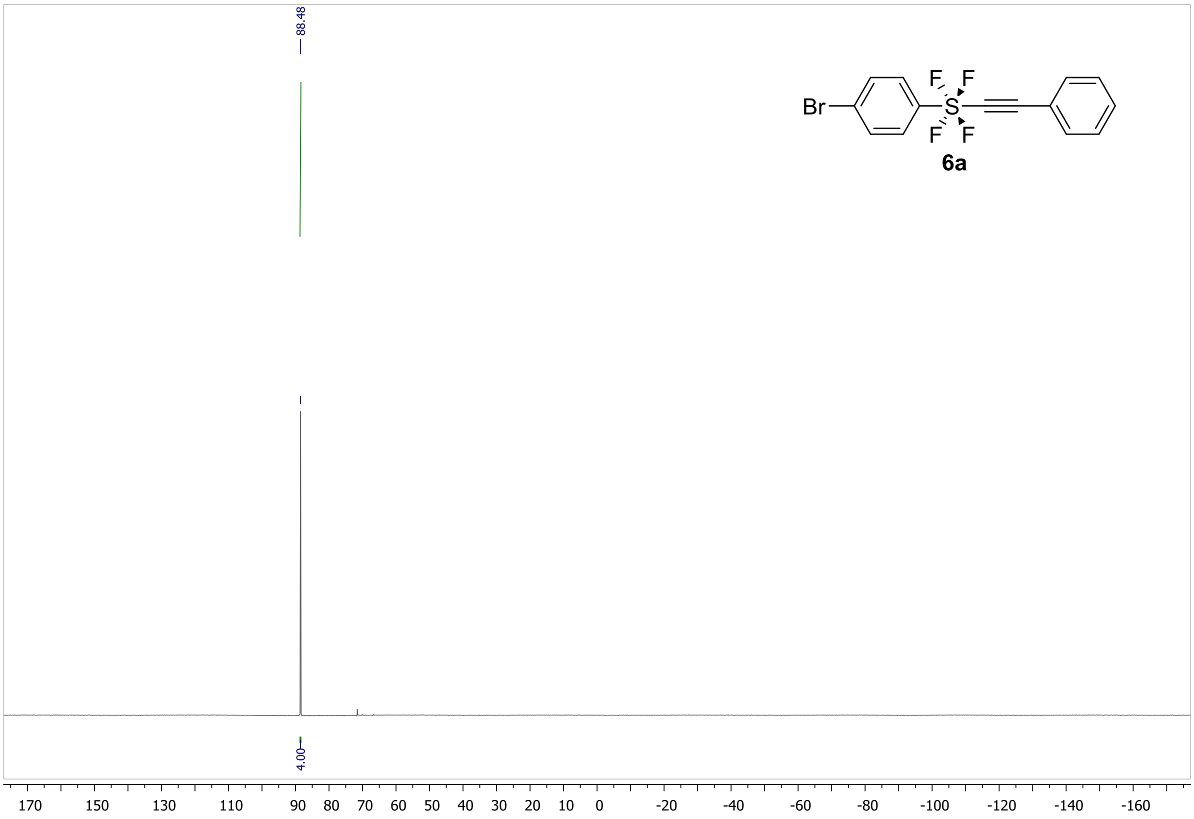
88.48
Br
F F
S
F F
6a
4.00
170 150 130 110 90 80 70 60 50 40 30 20 10 0 -20 -40 -60 -80 -100 -120 -140 -160

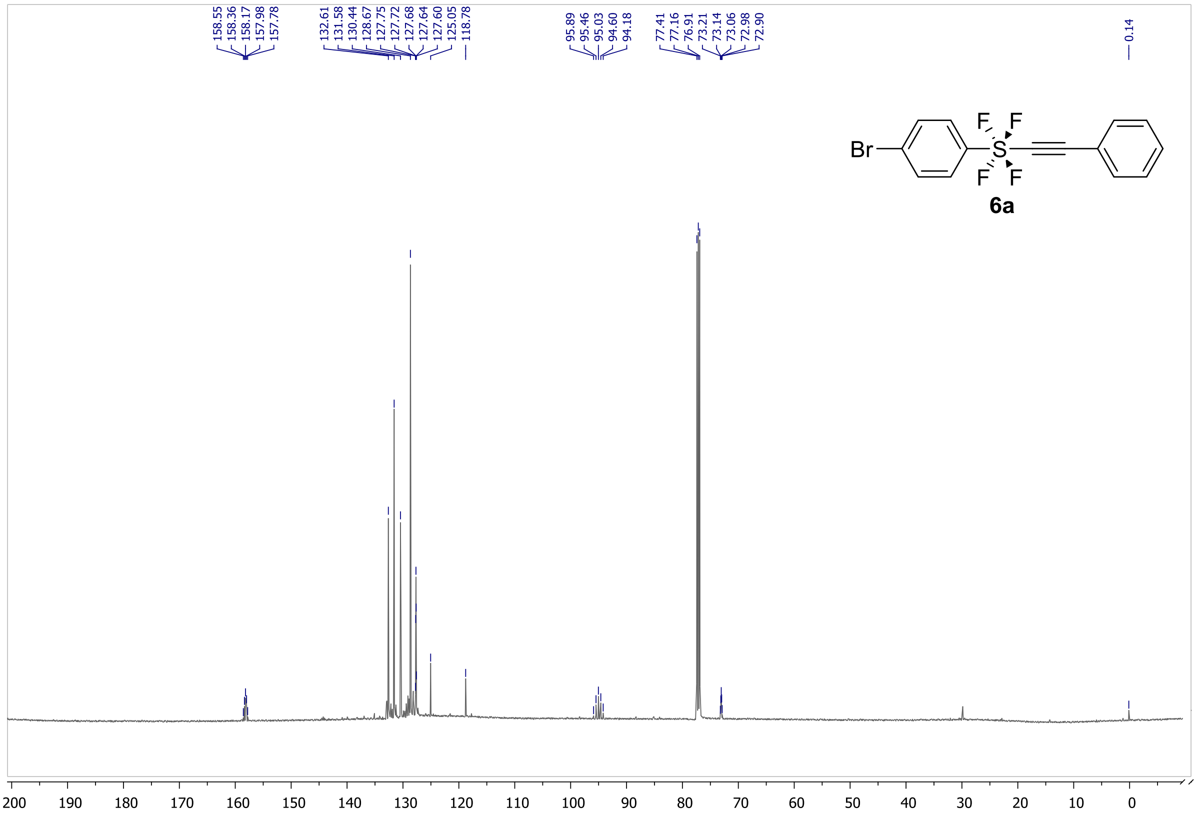
158.55 158.36 158.17 157.98 157.78
132.61 131.58 130.44 128.67 127.75 127.72 127.68 127.64 127.60 125.05 118.78
95.89 95.46 95.03 94.60 94.18
77.41 77.16 76.91 73.21 73.14 73.06 72.98 72.90
0.14
Br
F F
S
F F
6a
200 190 180 170 160 150 140 130 120 110 100 90 80 70 60 50 40 30 20 10 0

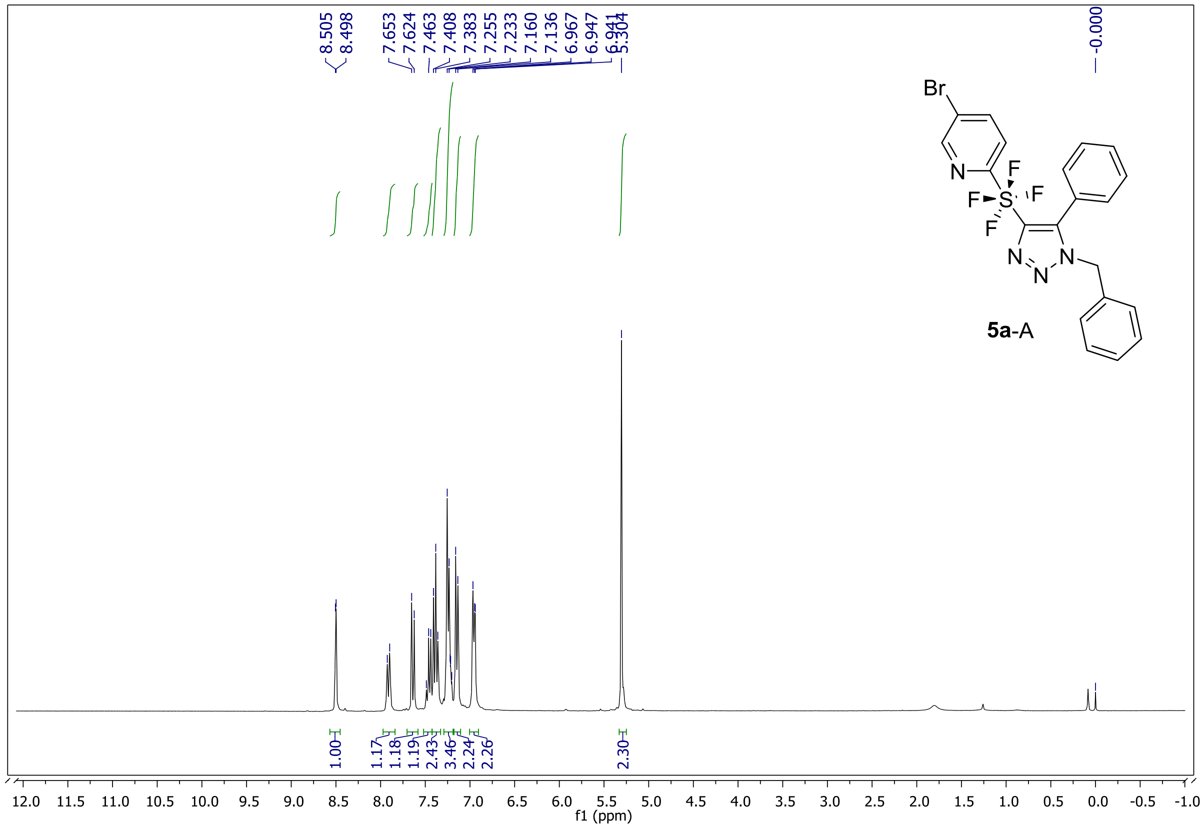
8.505
8.498
7.653
7.624
7.463
7.408
7.383
7.255
7.233
7.160
7.136
6.967
6.947
6.941
5.304
-0.000
Br
N
F
F
F
F
S
N
N
N
5a-A
1.00
1.17
1.18
1.19
2.43
3.46
2.24
2.26
2.30
12.0 11.5 11.0 10.5 10.0 9.5 9.0 8.5 8.0 7.5 7.0 6.5 6.0 5.5 5.0 4.5 4.0 3.5 3.0 2.5 2.0 1.5 1.0 0.5 0.0 -0.5 -1.0
f1 (ppm)

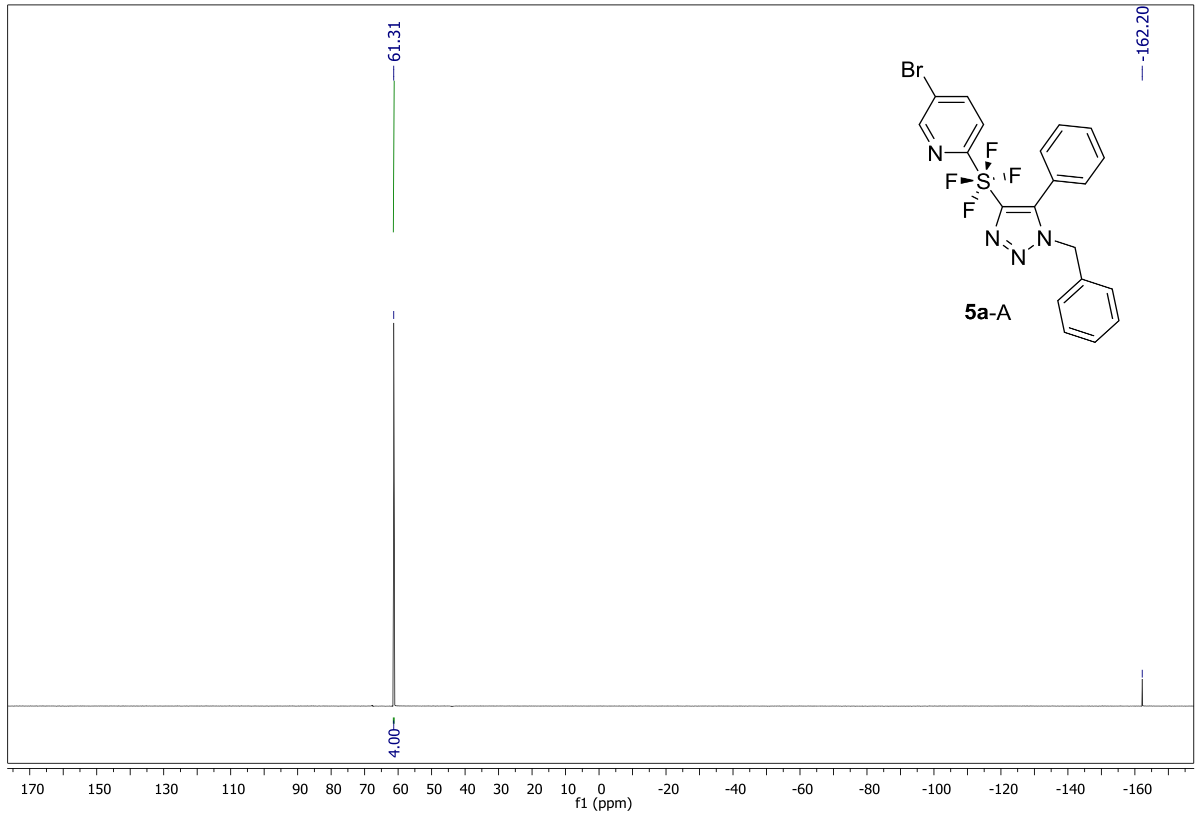
61.31
-162.20
Br
N
F
F
F
F
S
N
N
N
5a-A
4.00
170 150 130 110 90 80 70 60 50 40 30 20 10 0 -20 -40 -60 -80 -100 -120 -140 -160
f1 (ppm)

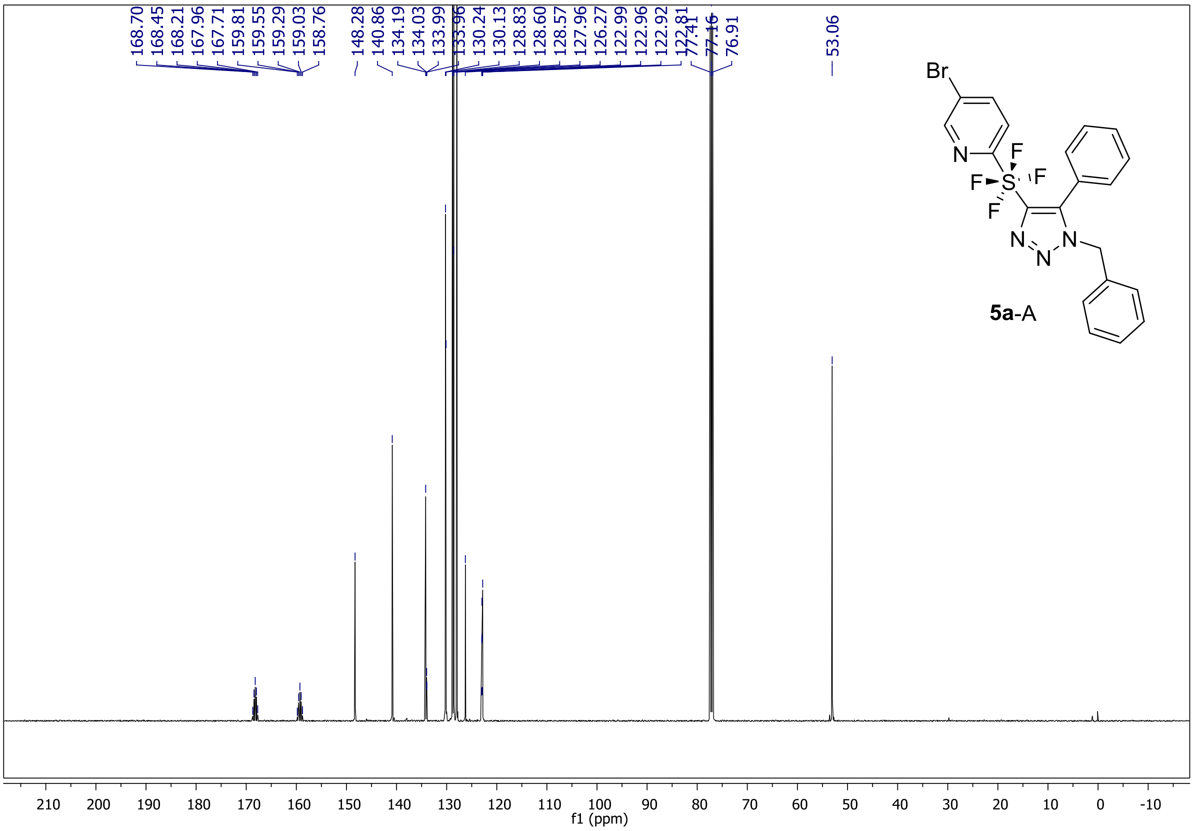
168.70
168.45
168.21
167.96
167.71
159.81
159.55
159.29
159.03
158.76
148.28
140.86
134.19
134.03
133.99
133.96
130.24
130.13
128.83
128.60
128.57
127.96
126.27
122.99
122.96
122.92
122.81
77.41
77.16
76.91
53.06
Br
N
F
F
S
F
F
N
N
N
5a-A
210 200 190 180 170 160 150 140 130 120 110 100 90 80 70 60 50 40 30 20 10 0 -10
f1 (ppm)

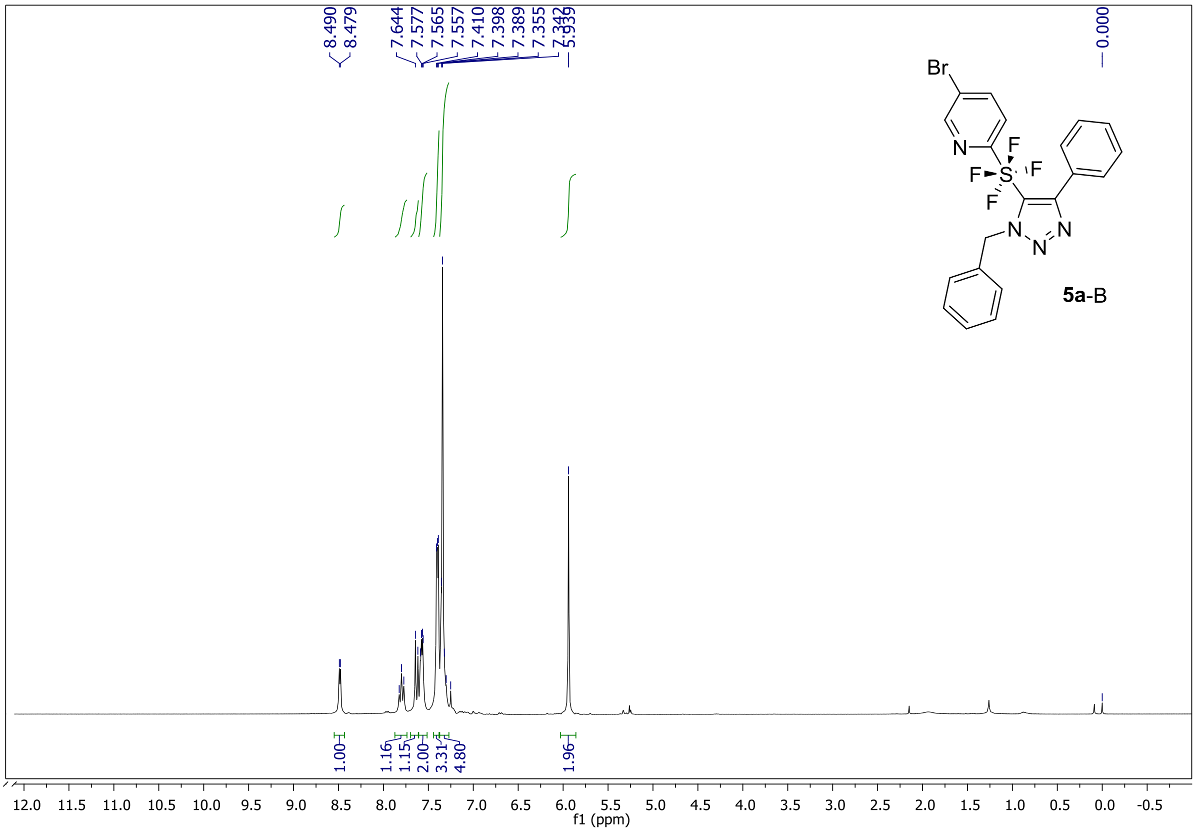
8.490
8.479
7.644
7.577
7.565
7.557
7.410
7.398
7.389
7.355
7.342
5.939
0.000
Br
N
F
F
S
F
F
N
N
N
5a-B
1.00
1.16
1.15
2.00
3.31
4.80
1.96
12.0 11.5 11.0 10.5 10.0 9.5 9.0 8.5 8.0 7.5 7.0 6.5 6.0 5.5 5.0 4.5 4.0 3.5 3.0 2.5 2.0 1.5 1.0 0.5 0.0 -0.5
f1 (ppm)

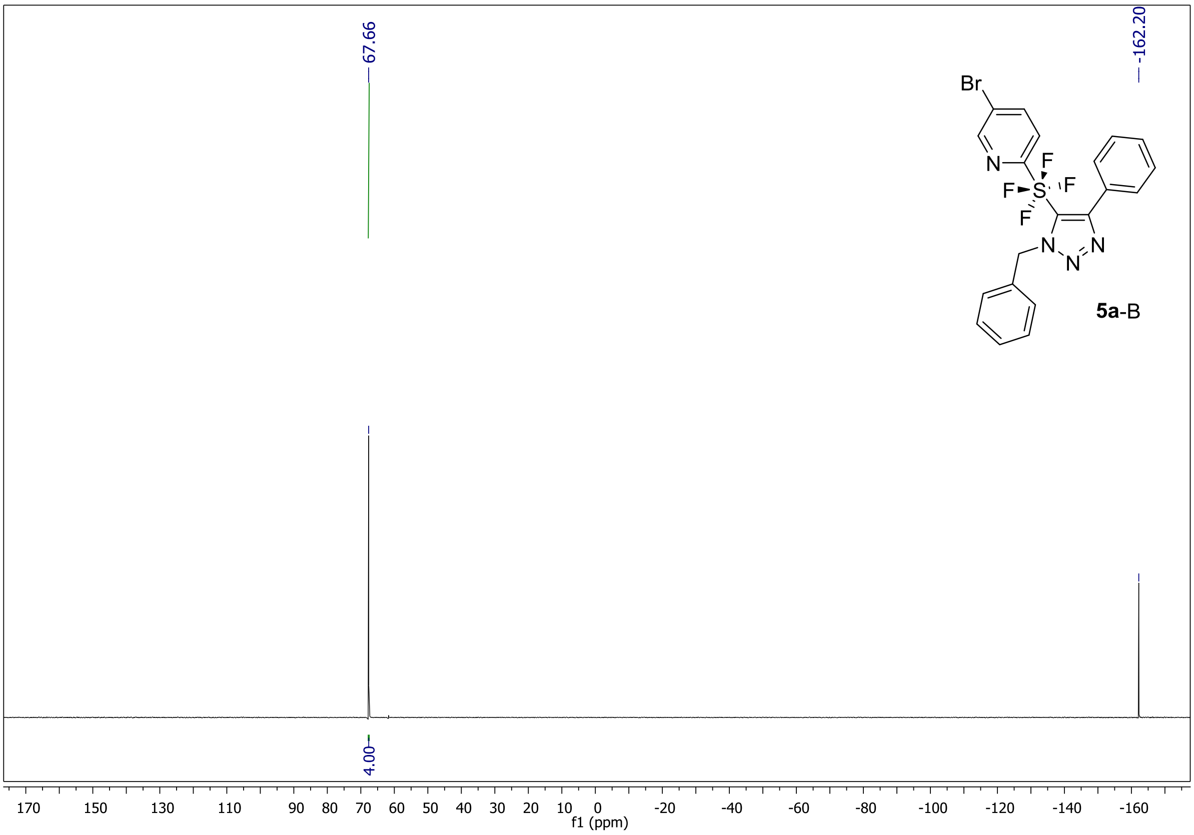
67.66
-162.20
4.00
170 150 130 110 90 80 70 60 50 40 30 20 10 0 -20 -40 -60 -80 -100 -120 -140 -160
f1 (ppm)
Br
N
F
F
F
F
S
N
N
N
5a-B

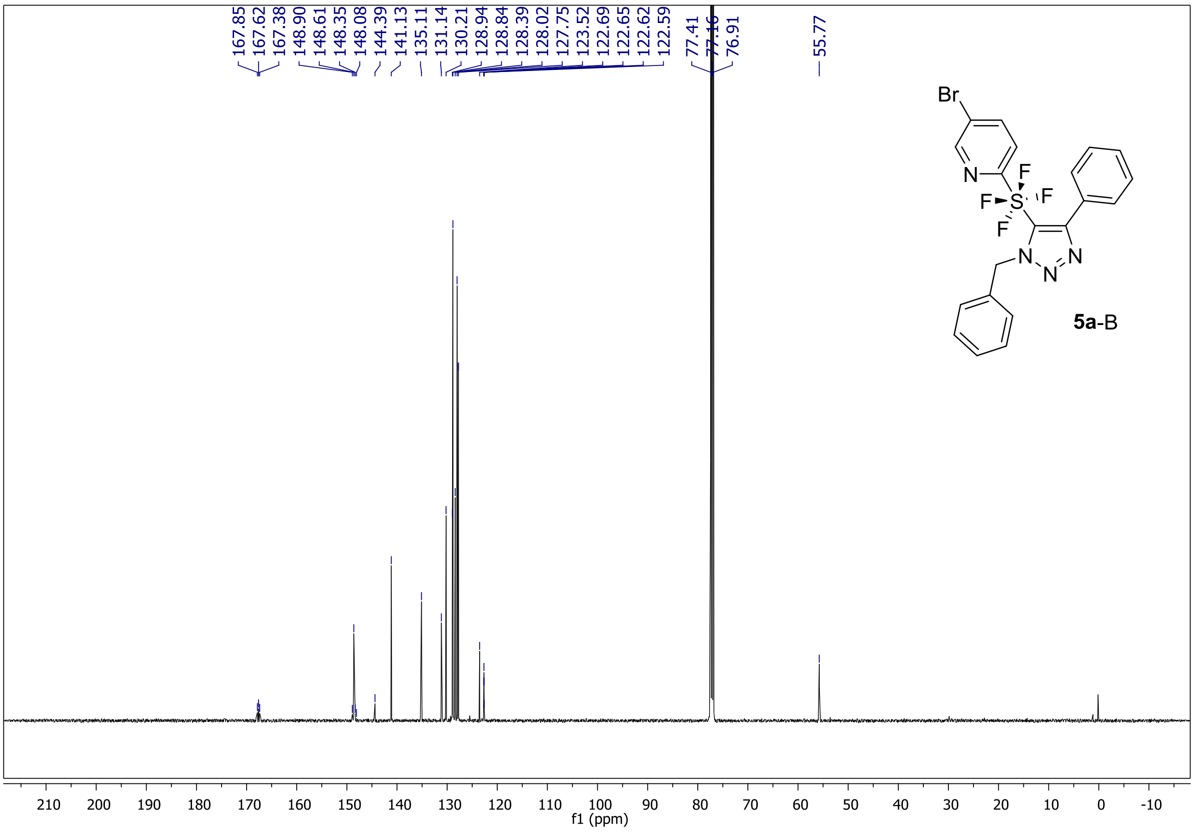
167.85
167.62
167.38
148.90
148.61
148.35
148.08
144.39
141.13
135.11
131.14
130.21
128.94
128.84
128.39
128.02
127.75
123.52
122.69
122.65
122.62
122.59
77.41
77.16
76.91
55.77
210 200 190 180 170 160 150 140 130 120 110 100 90 80 70 60 50 40 30 20 10 0 -10
f1 (ppm)
Br
N
F
F
F
F
S
N
N
N
5a-B

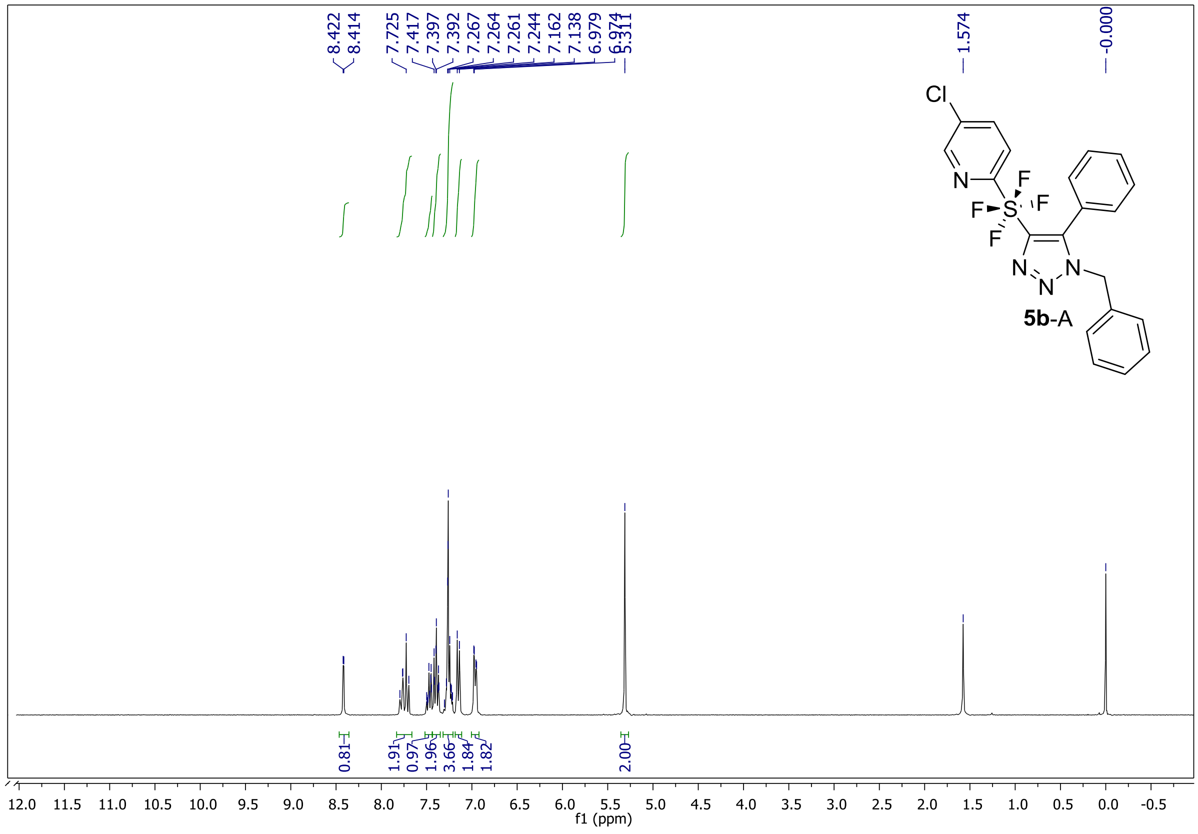
8.422
8.414
7.725
7.417
7.397
7.392
7.267
7.264
7.261
7.244
7.162
7.138
6.979
6.974
5.311
1.574
-0.000
Cl
N
F
F
F
F
S
N
N
N
5b-A
0.81
1.91
0.97
1.96
3.66
1.84
1.82
2.00
12.0 11.5 11.0 10.5 10.0 9.5 9.0 8.5 8.0 7.5 7.0 6.5 6.0 5.5 5.0 4.5 4.0 3.5 3.0 2.5 2.0 1.5 1.0 0.5 0.0 -0.5
f1 (ppm)

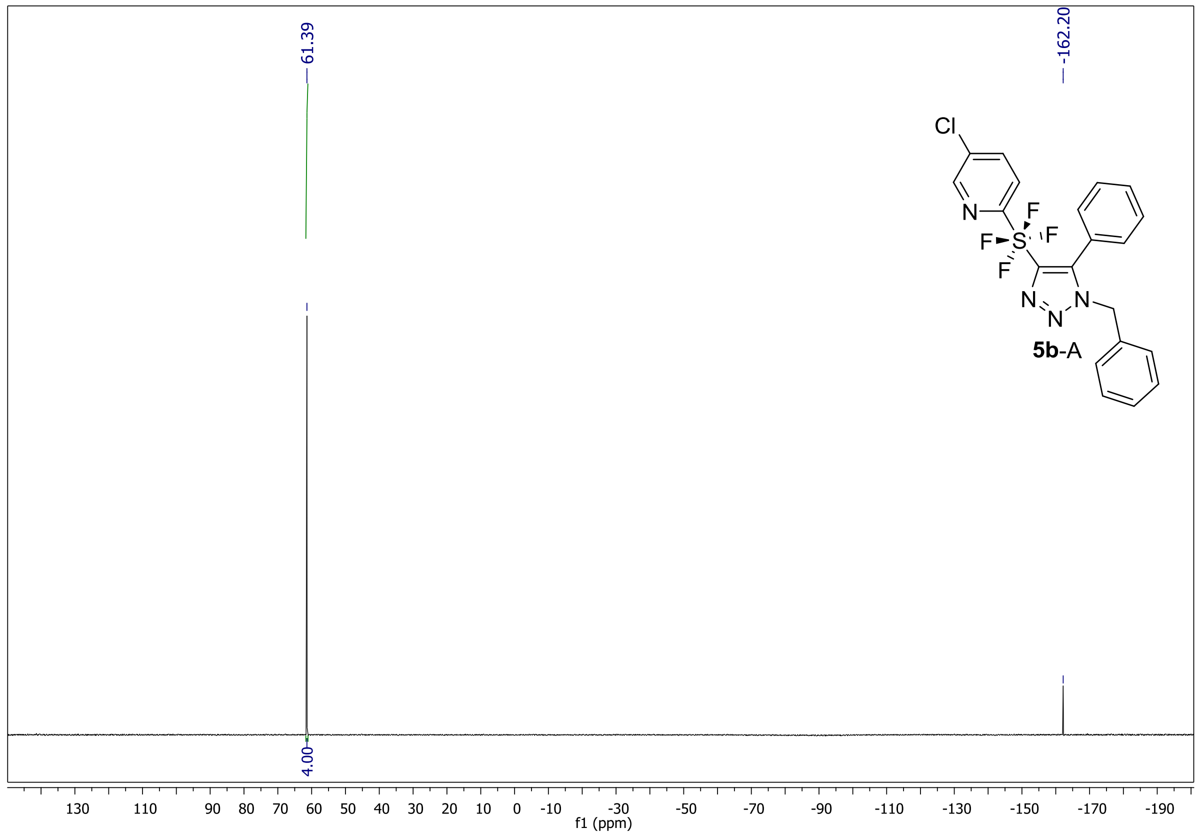
61.39
-162.20
Cl
N
F
F
F
F
S
N
N
N
5b-A
4.00
130 110 90 80 70 60 50 40 30 20 10 0 -10 -30 -50 -70 -90 -110 -130 -150 -170 -190
f1 (ppm)

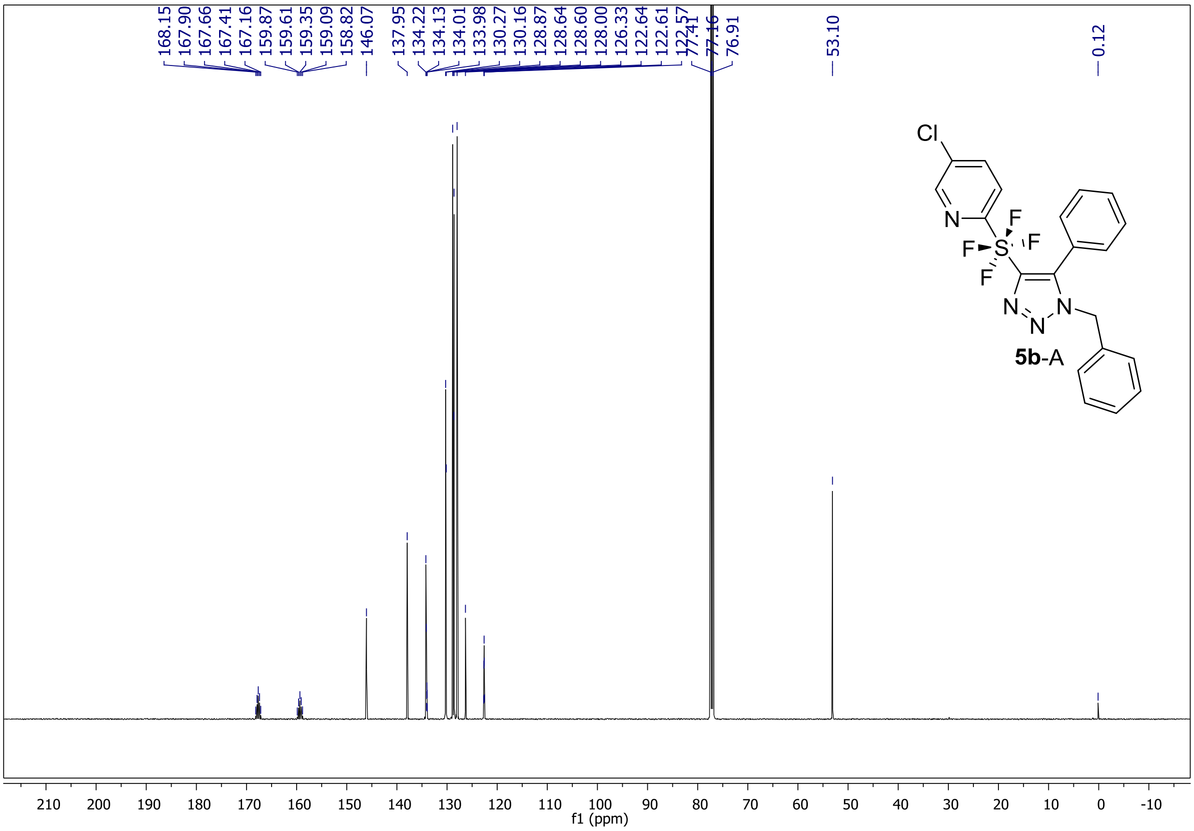
168.15
167.90
167.66
167.41
167.16
159.87
159.61
159.35
159.09
158.82
146.07
137.95
134.22
134.13
134.01
133.98
130.27
130.16
128.87
128.64
128.60
128.00
126.33
122.64
122.61
122.57
77.41
77.16
76.91
53.10
0.12
Cl
N
F
F
F
F
S
N
N
N
5b-A
210 200 190 180 170 160 150 140 130 120 110 100 90 80 70 60 50 40 30 20 10 0 -10
f1 (ppm)

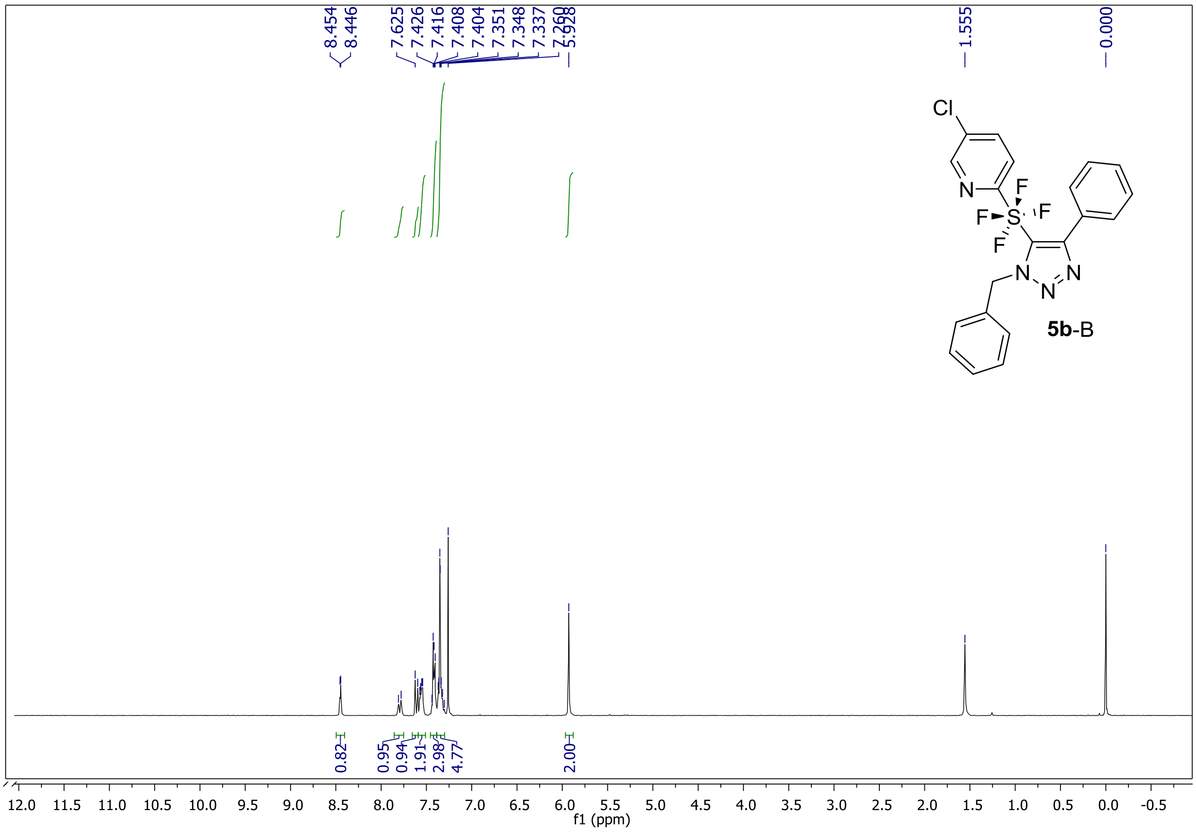
8.454
8.446
7.625
7.426
7.416
7.408
7.404
7.351
7.348
7.337
7.260
5.928
1.555
0.000
Cl
N
F
F
F
F
S
N
N
N
5b-B
0.82
0.95
0.94
1.91
2.98
4.77
2.00
12.0 11.5 11.0 10.5 10.0 9.5 9.0 8.5 8.0 7.5 7.0 6.5 6.0 5.5 5.0 4.5 4.0 3.5 3.0 2.5 2.0 1.5 1.0 0.5 0.0 -0.5
f1 (ppm)

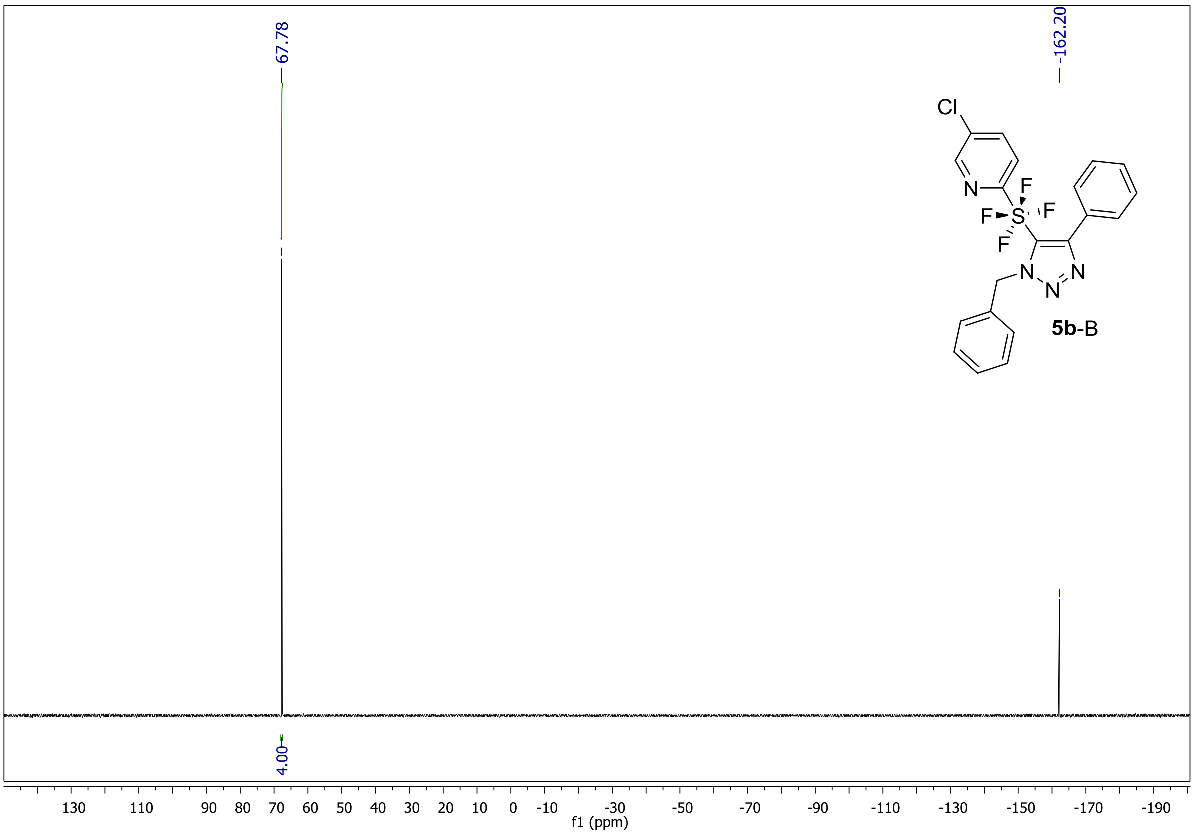
67.78
-162.20
4.00
5b-B
f1 (ppm)

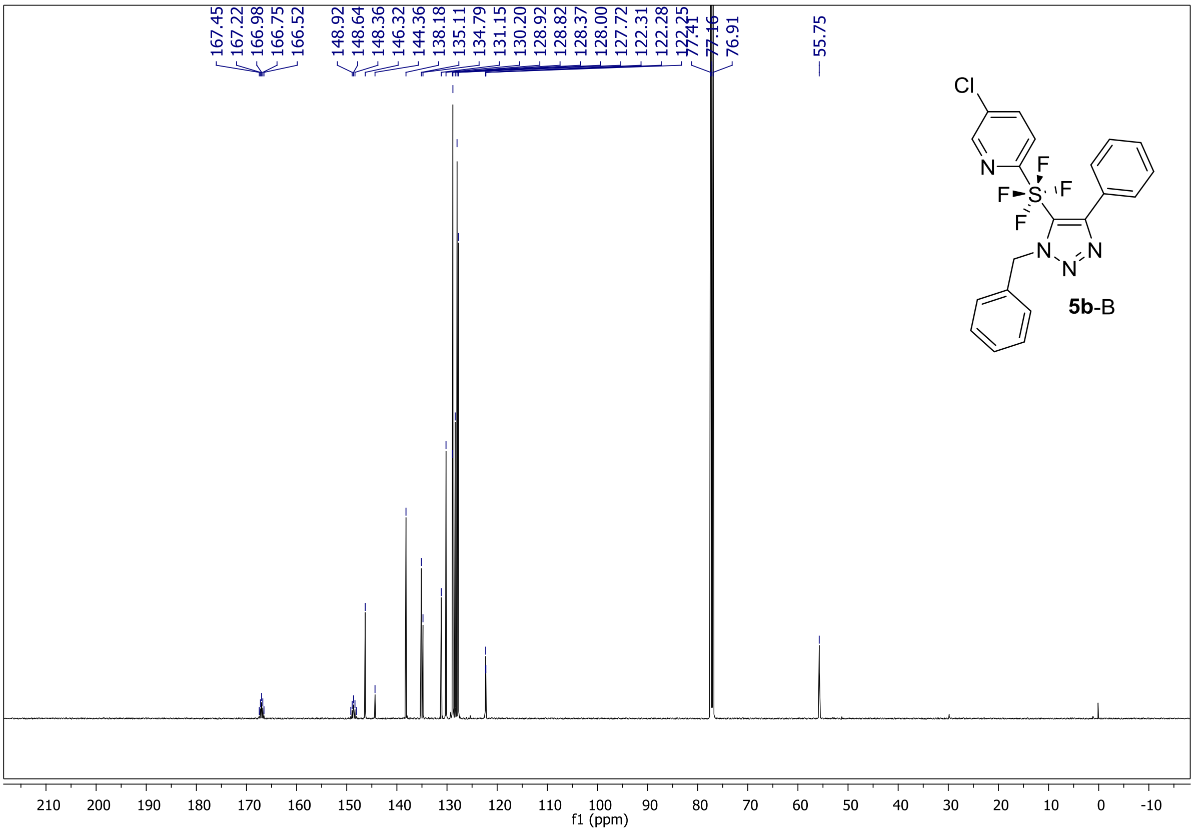
167.45
167.22
166.98
166.75
166.52
148.92
148.64
148.36
146.32
144.36
138.18
135.11
134.79
131.15
130.20
128.92
128.82
128.37
128.00
127.72
122.31
122.28
122.25
77.41
77.16
76.91
55.75
5b-B
f1 (ppm)

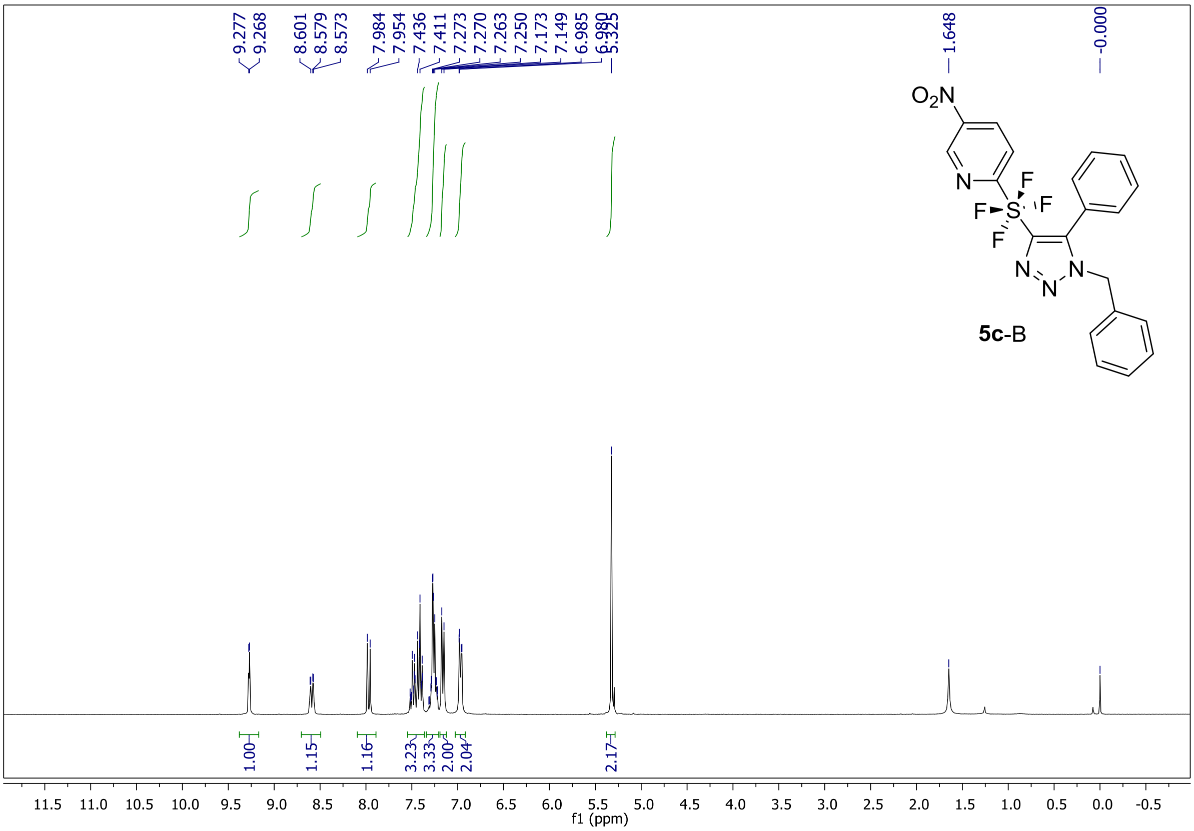
9.277
9.268
8.601
8.579
8.573
7.984
7.954
7.436
7.411
7.273
7.270
7.263
7.250
7.173
7.149
6.985
6.980
5.325
1.648
-0.000
O_2N
N
F
F
S
F
F
N
N
N
5c-B
1.00
1.15
1.16
3.23
3.33
2.00
2.04
2.17
11.5 11.0 10.5 10.0 9.5 9.0 8.5 8.0 7.5 7.0 6.5 6.0 5.5 5.0 4.5 4.0 3.5 3.0 2.5 2.0 1.5 1.0 0.5 0.0 -0.5
f1 (ppm)

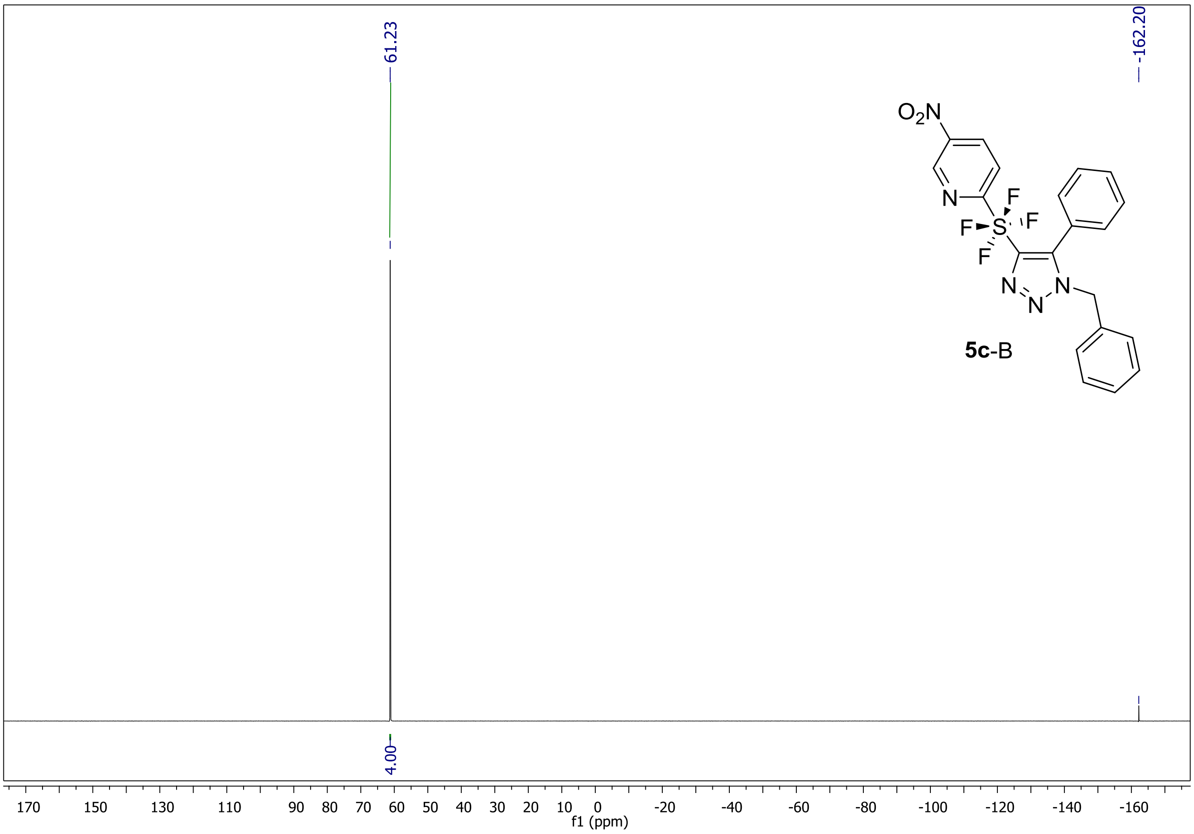
61.23
-162.20
O_2N
N
F
F
S
F
F
N
N
N
5c-B
4.00
170 150 130 110 90 80 70 60 50 40 30 20 10 0 -20 -40 -60 -80 -100 -120 -140 -160
f1 (ppm)

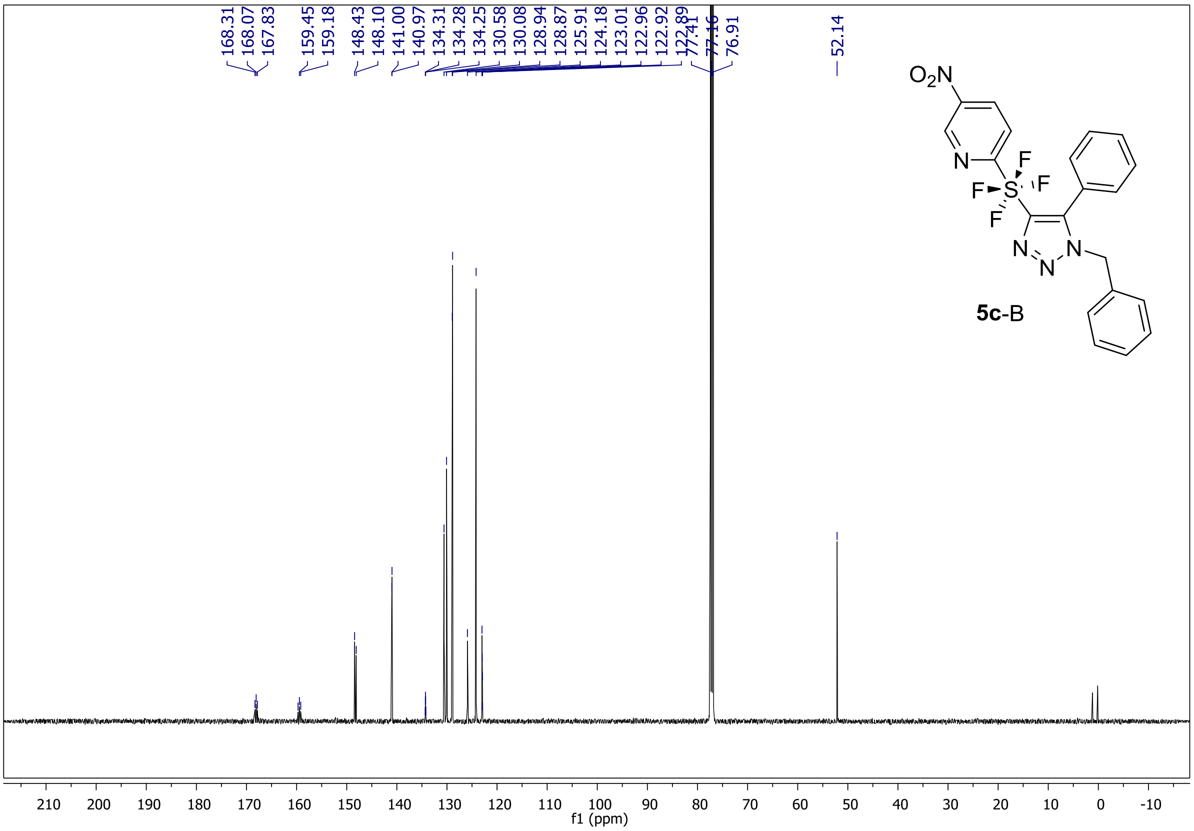
168.31
168.07
167.83
159.45
159.18
148.43
148.10
141.00
140.97
134.31
134.28
134.25
130.58
130.08
128.94
128.87
125.91
124.18
123.01
122.96
122.92
122.89
77.41
77.16
76.91
52.14
O2N
N
F
S
N
5c-B
f1 (ppm)

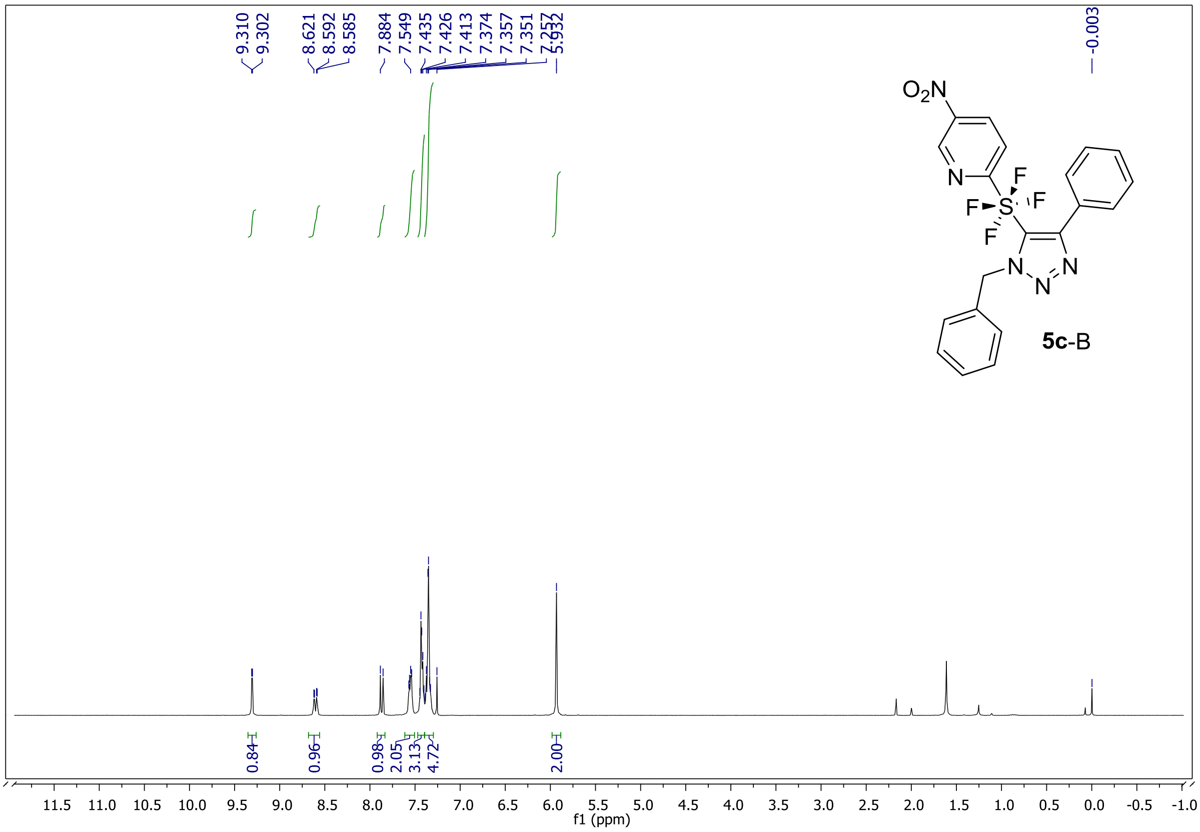
9.310
9.302
8.621
8.592
8.585
7.884
7.549
7.435
7.426
7.413
7.374
7.357
7.351
7.257
5.932
-0.003
O2N
N
F
S
N
5c-B
0.84
0.96
0.98
2.05
3.13
4.72
2.00
f1 (ppm)

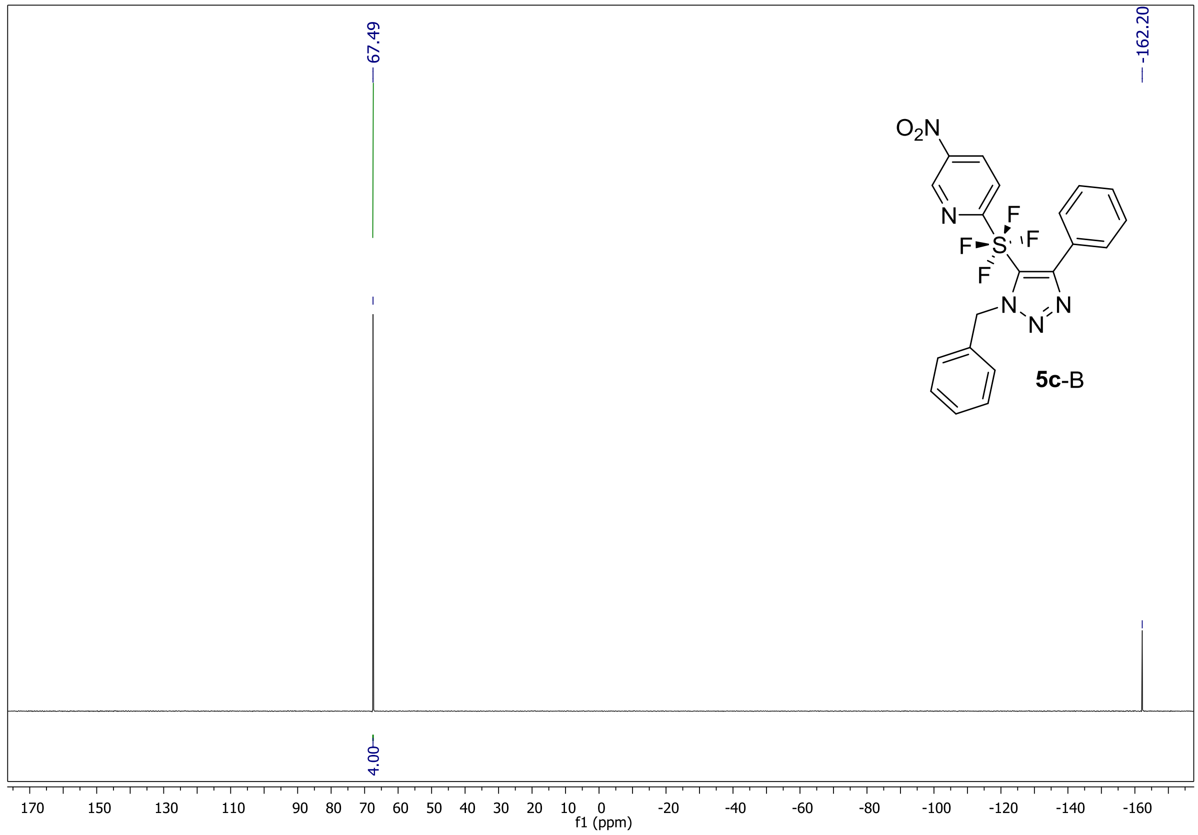

67.49
-162.20
O_2N
N
F
F
F
S
F
N
N
N
5c-B
4.00
170 150 130 110 90 80 70 60 50 40 30 20 10 0 -20 -40 -60 -80 -100 -120 -140 -160
f1 (ppm)

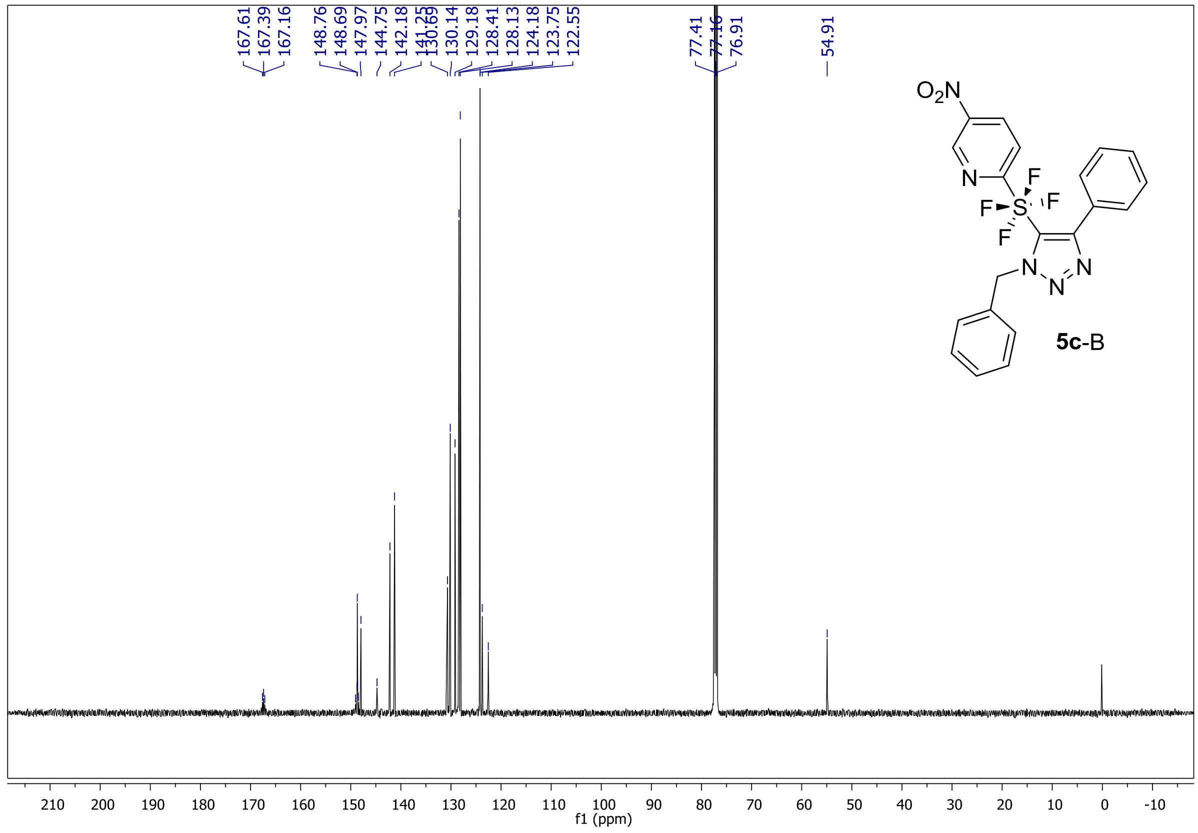

167.61
167.39
167.16
148.76
148.69
147.97
144.75
142.18
141.25
130.69
130.14
129.18
128.41
128.13
124.18
123.75
122.55
77.41
77.16
76.91
54.91
O_2N
N
F
F
F
S
F
N
N
N
5c-B
210 200 190 180 170 160 150 140 130 120 110 100 90 80 70 60 50 40 30 20 10 0 -10
f1 (ppm)

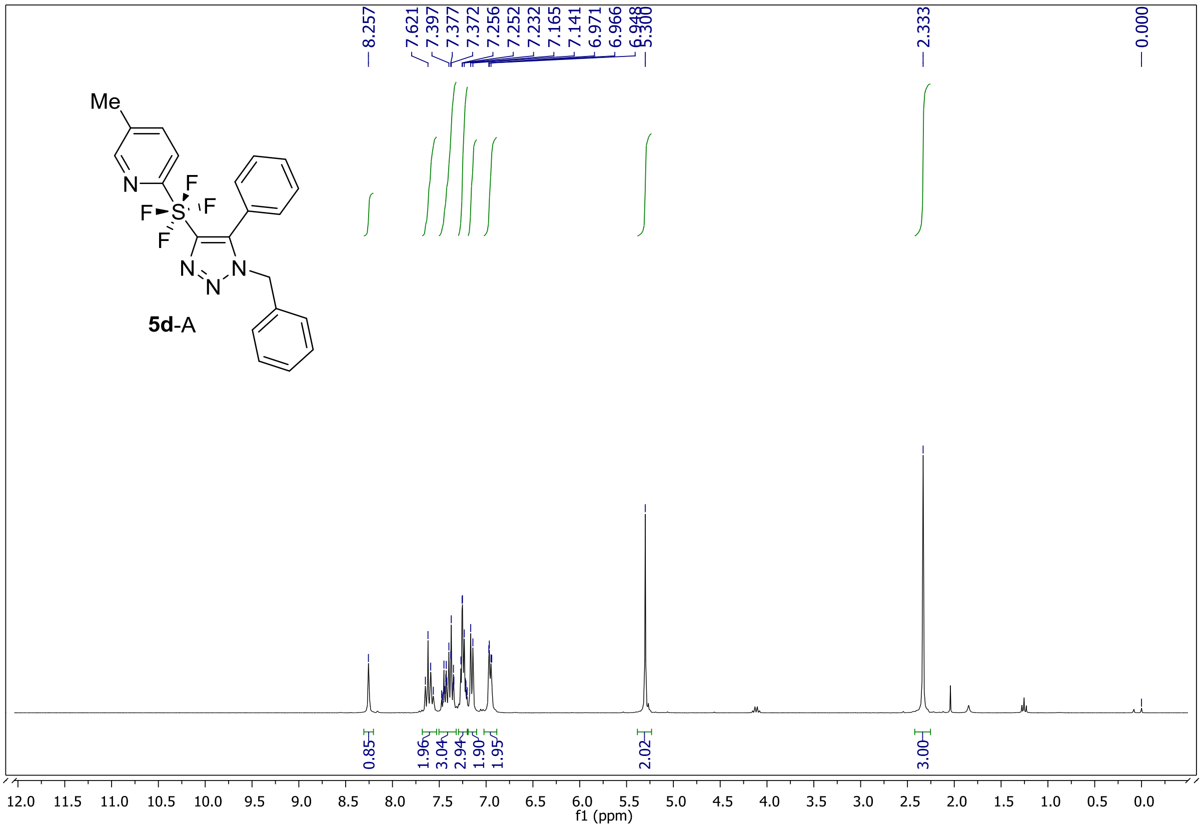
8.257
7.621
7.397
7.377
7.372
7.256
7.252
7.232
7.165
7.141
6.971
6.966
6.948
5.300
2.333
0.000
Me
N
F
F
F
S
F
N
N
N
5d-A
0.85
1.96
3.04
2.94
1.90
1.95
2.02
3.00
12.0 11.5 11.0 10.5 10.0 9.5 9.0 8.5 8.0 7.5 7.0 6.5 6.0 5.5 5.0 4.5 4.0 3.5 3.0 2.5 2.0 1.5 1.0 0.5 0.0
f1 (ppm)

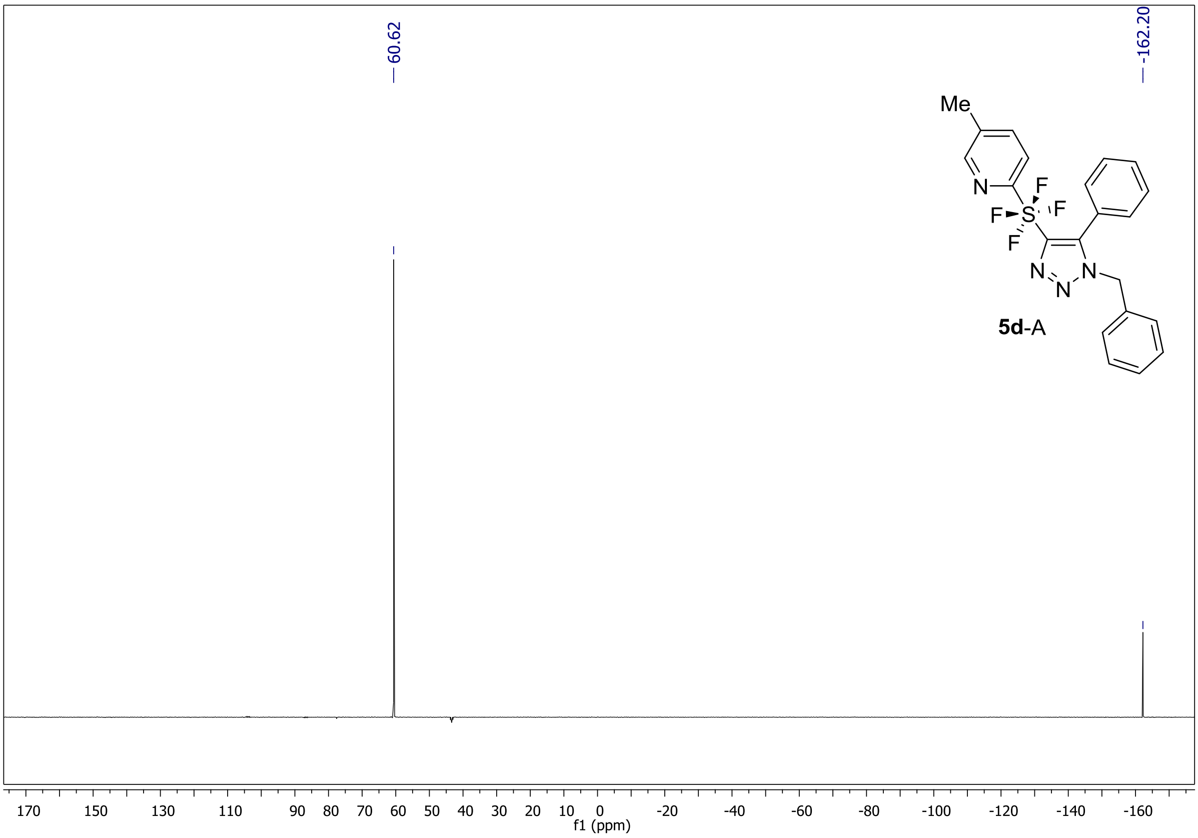
60.62
-162.20
Me
N
F
F
F
S
F
N
N
N
5d-A
170 150 130 110 90 80 70 60 50 40 30 20 10 0 -20 -40 -60 -80 -100 -120 -140 -160
f1 (ppm)

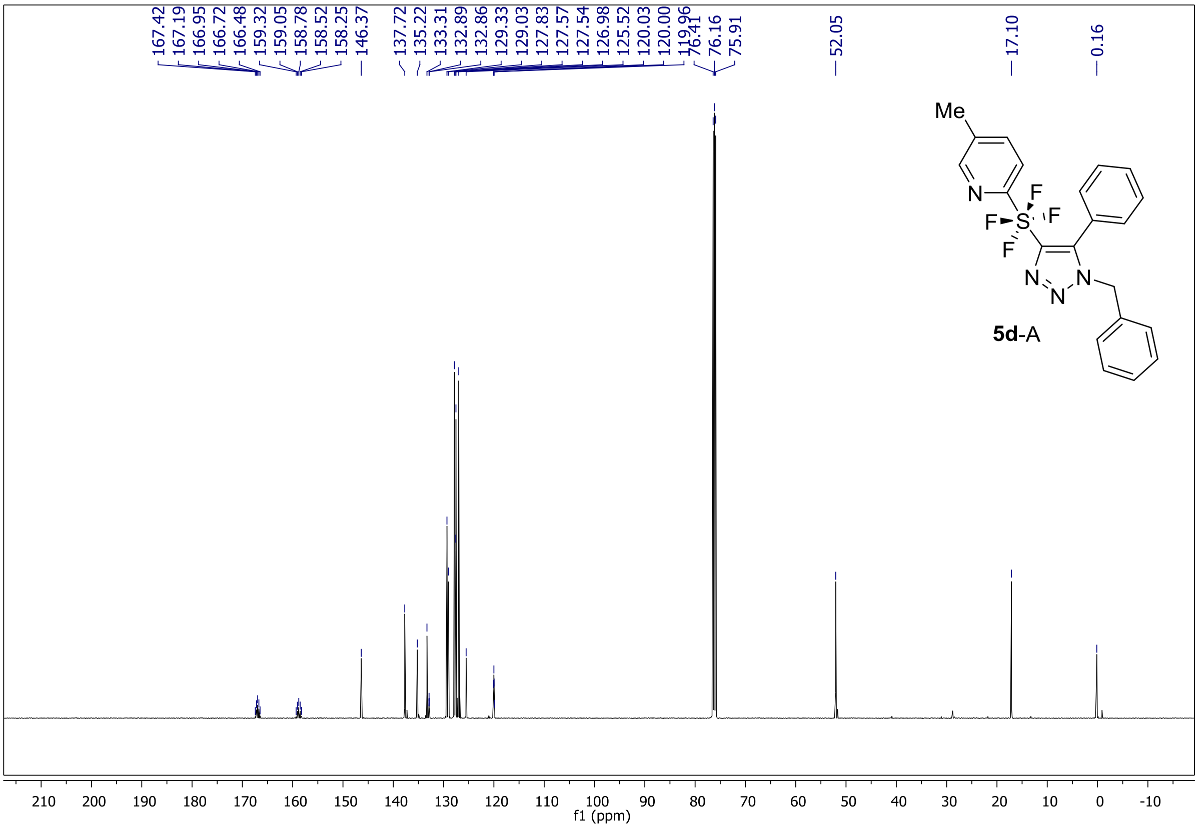
167.42
167.19
166.95
166.72
166.48
159.32
159.05
158.78
158.52
158.25
146.37
137.72
135.22
133.31
132.89
132.86
129.33
129.03
127.83
127.57
127.54
126.98
125.52
120.03
120.00
119.96
76.41
76.16
75.91
52.05
17.10
0.16
Me
N
F
F
S
F
F
N
N
N
5d-A
f1 (ppm)

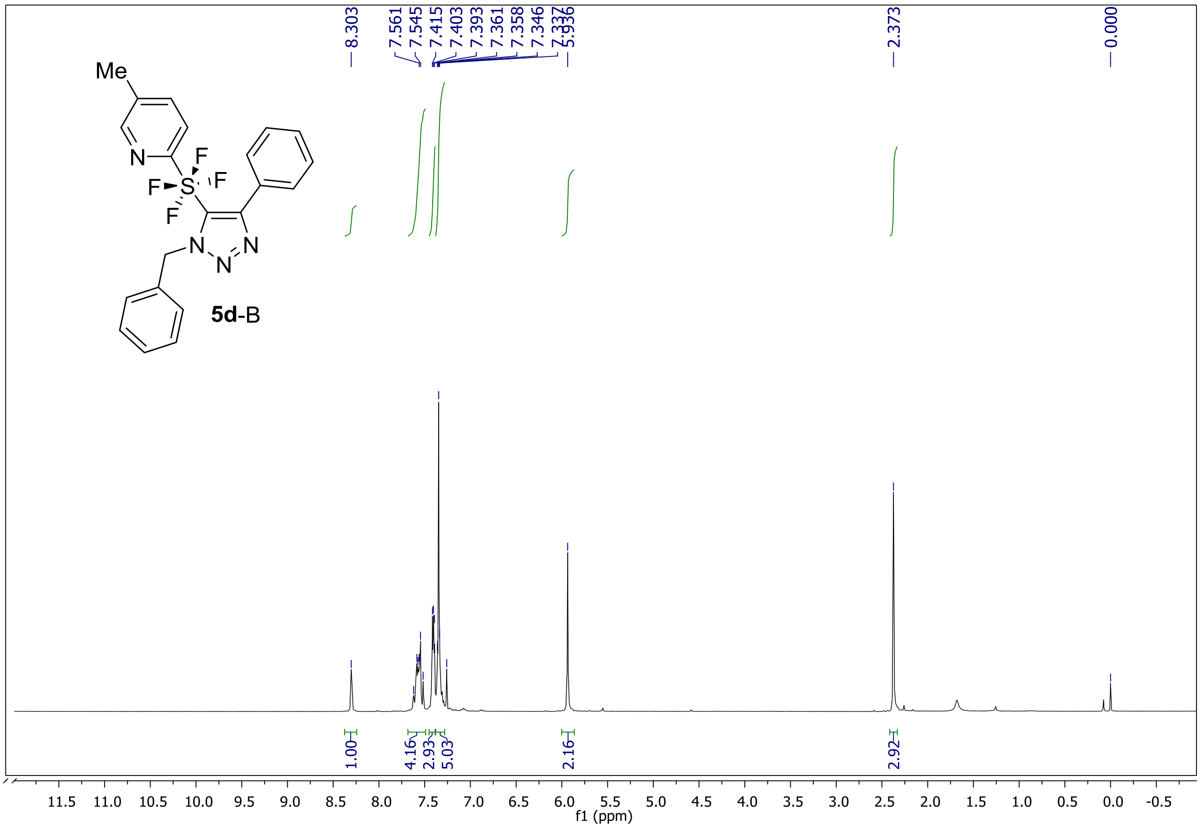
8.303
7.561
7.545
7.415
7.403
7.393
7.361
7.358
7.346
7.337
5.936
2.373
0.000
Me
N
F
F
S
F
F
N
N
N
5d-B
1.00
4.16
2.93
5.03
2.16
2.92
f1 (ppm)

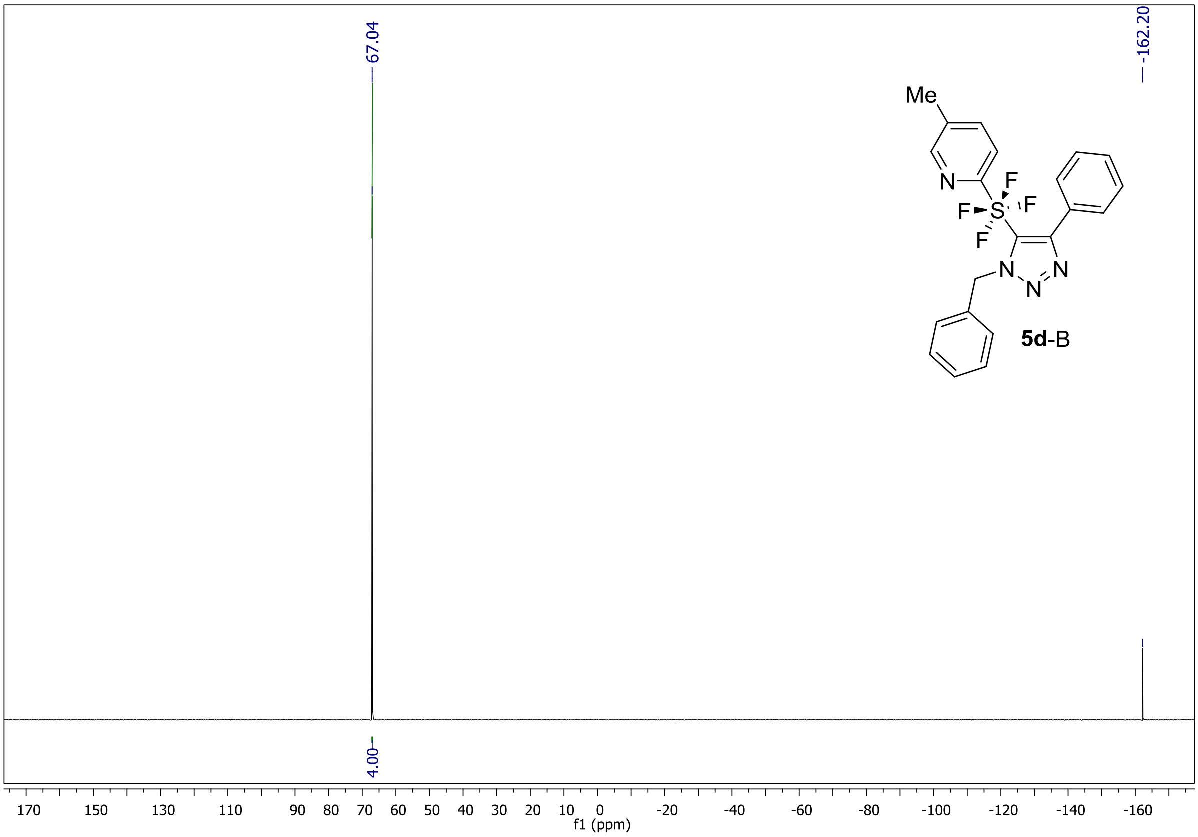

67.04
-162.20
Me
N
F
F
F
F
S
N
N
N
5d-B
4.00
170
150
130
110
90
80
70
60
50
40
30
20
10
0
-20
-40
-60
-80
-100
-120
-140
-160
f1 (ppm)

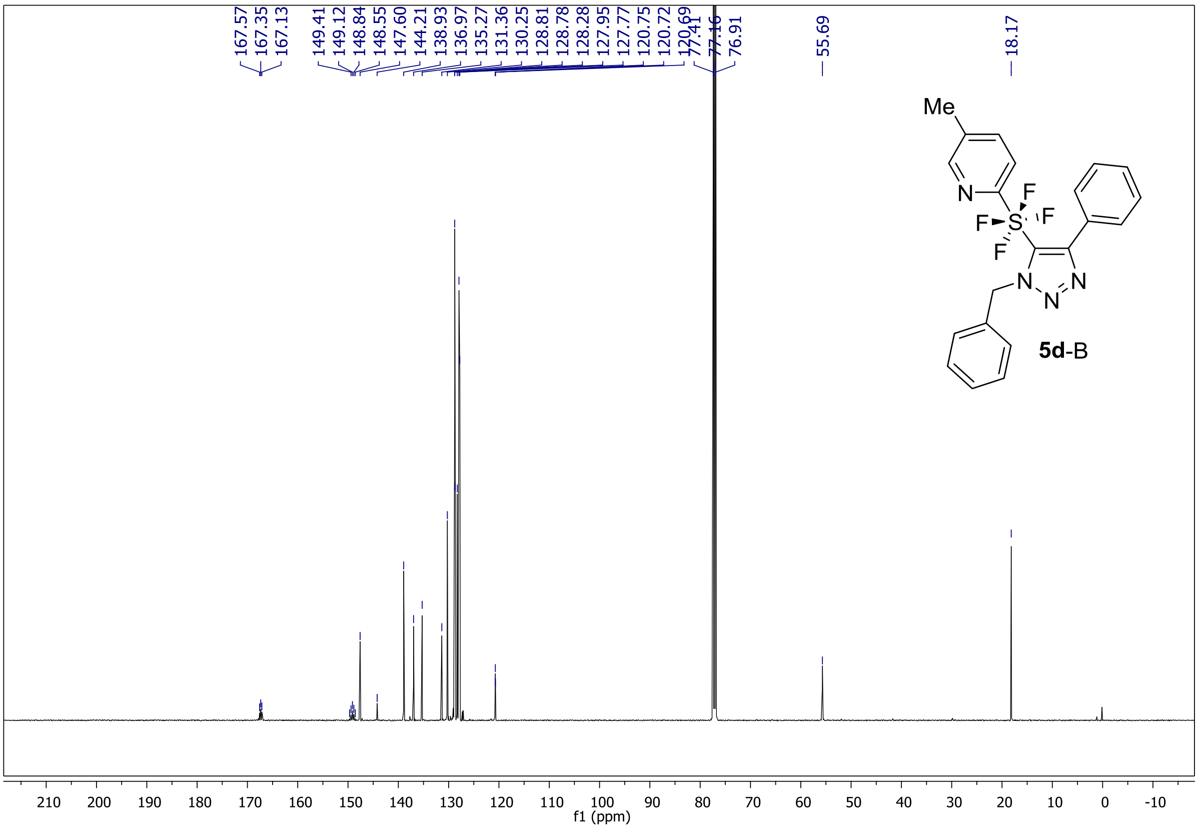

167.57
167.35
167.13
149.41
149.12
148.84
148.55
147.60
144.21
138.93
136.97
135.27
131.36
130.25
128.81
128.78
128.28
127.95
127.77
120.75
120.72
120.69
77.41
77.16
76.91
55.69
18.17
Me
N
F
F
F
F
S
N
N
N
5d-B
210
200
190
180
170
160
150
140
130
120
110
100
90
80
70
60
50
40
30
20
10
0
-10
f1 (ppm)

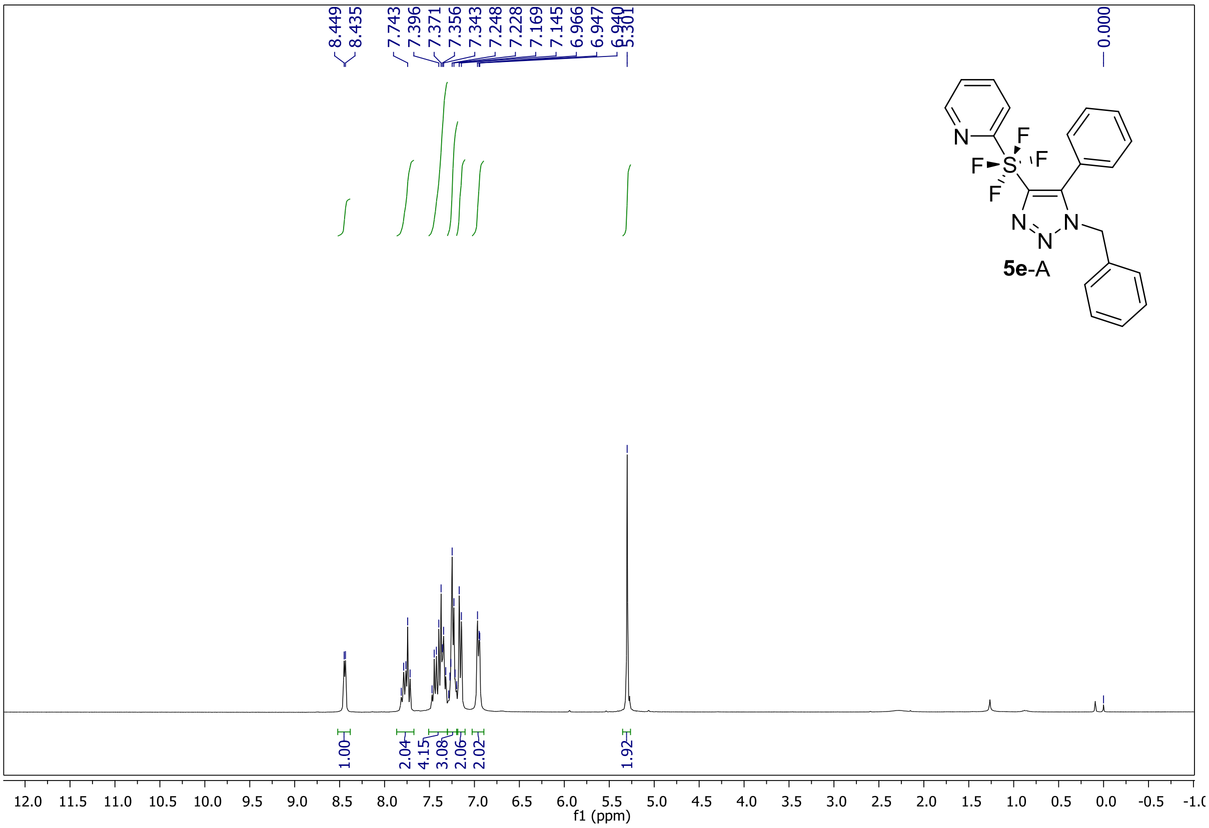
8.449
8.435
7.743
7.396
7.371
7.356
7.343
7.248
7.228
7.169
7.145
6.966
6.947
6.940
5.301
0.000
5e-A
1.00
2.04
4.15
3.08
2.06
2.02
1.92
f1 (ppm)

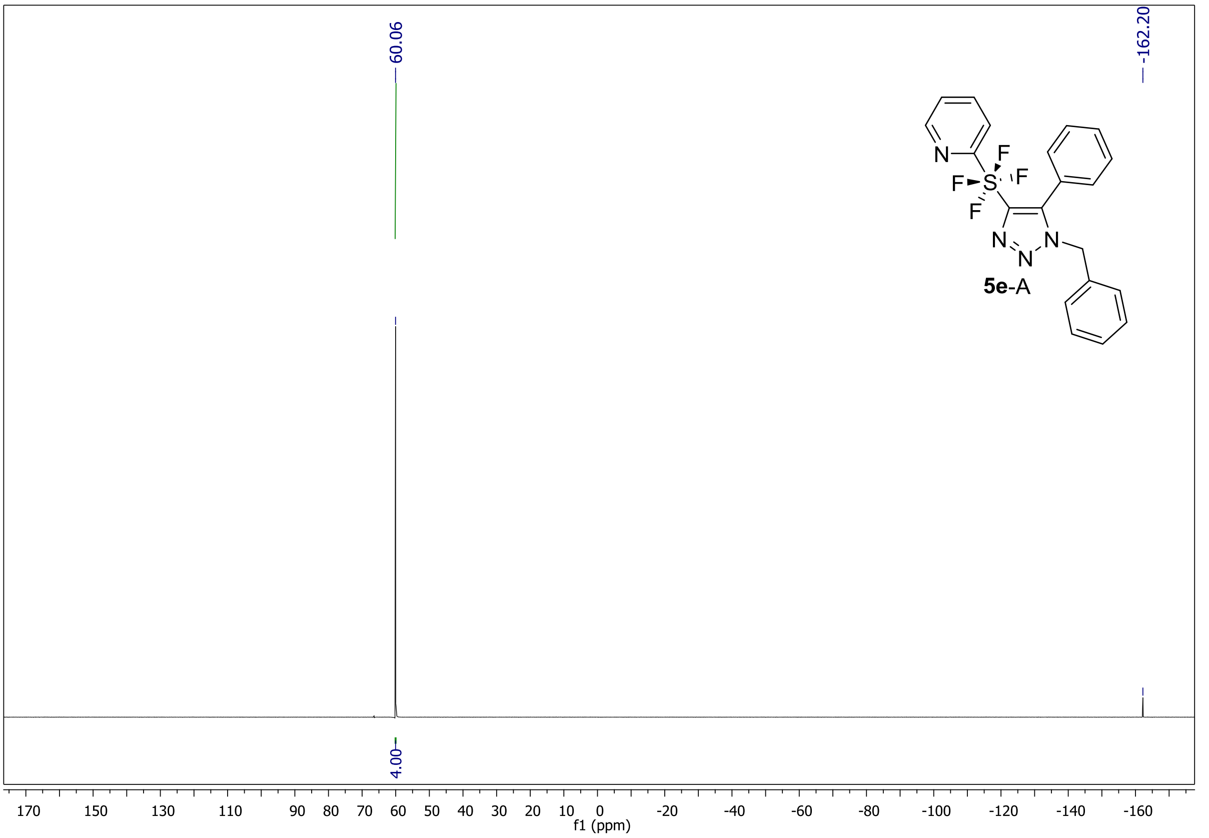
60.06
-162.20
5e-A
4.00
f1 (ppm)

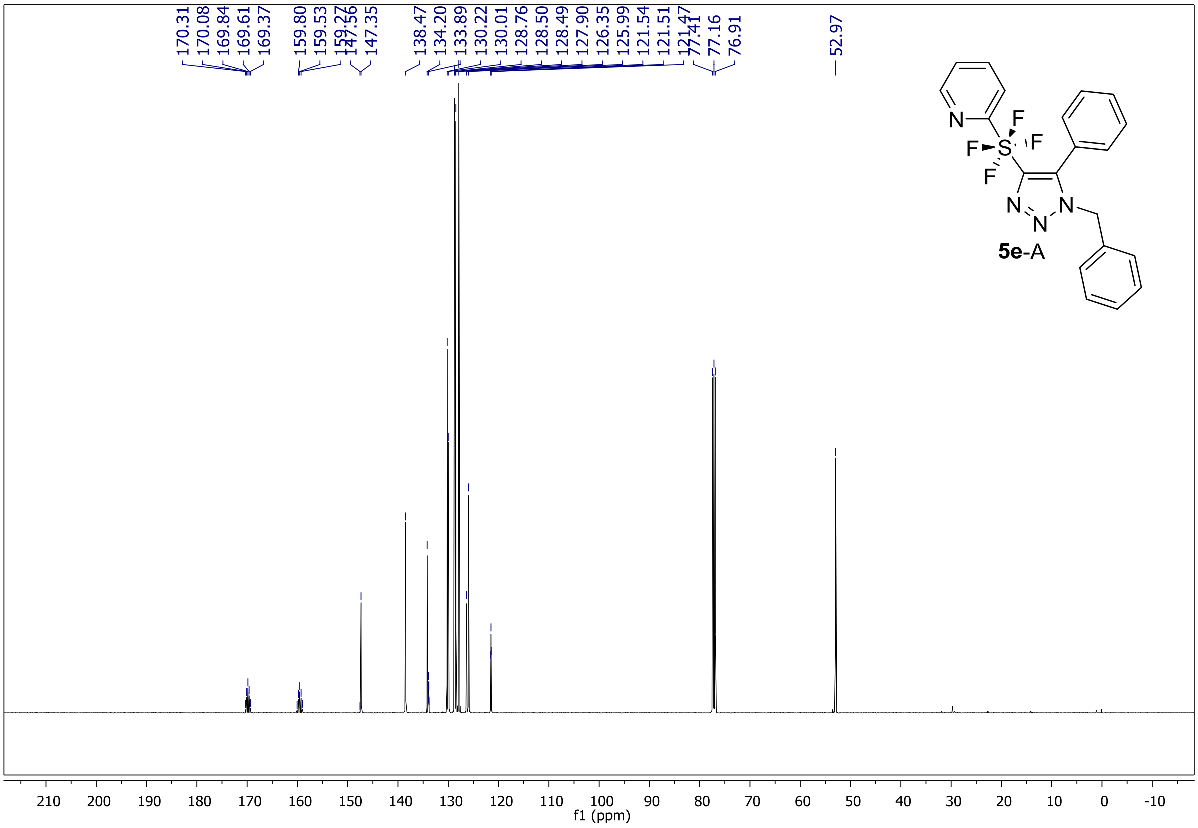
170.31
170.08
169.84
169.61
169.37
159.80
159.53
159.27
147.56
147.35
138.47
134.20
133.89
130.22
130.01
128.76
128.50
128.49
127.90
126.35
125.99
121.54
121.51
121.47
77.41
77.16
76.91
52.97
5e-A
210 200 190 180 170 160 150 140 130 120 110 100 90 80 70 60 50 40 30 20 10 0 -10
f1 (ppm)

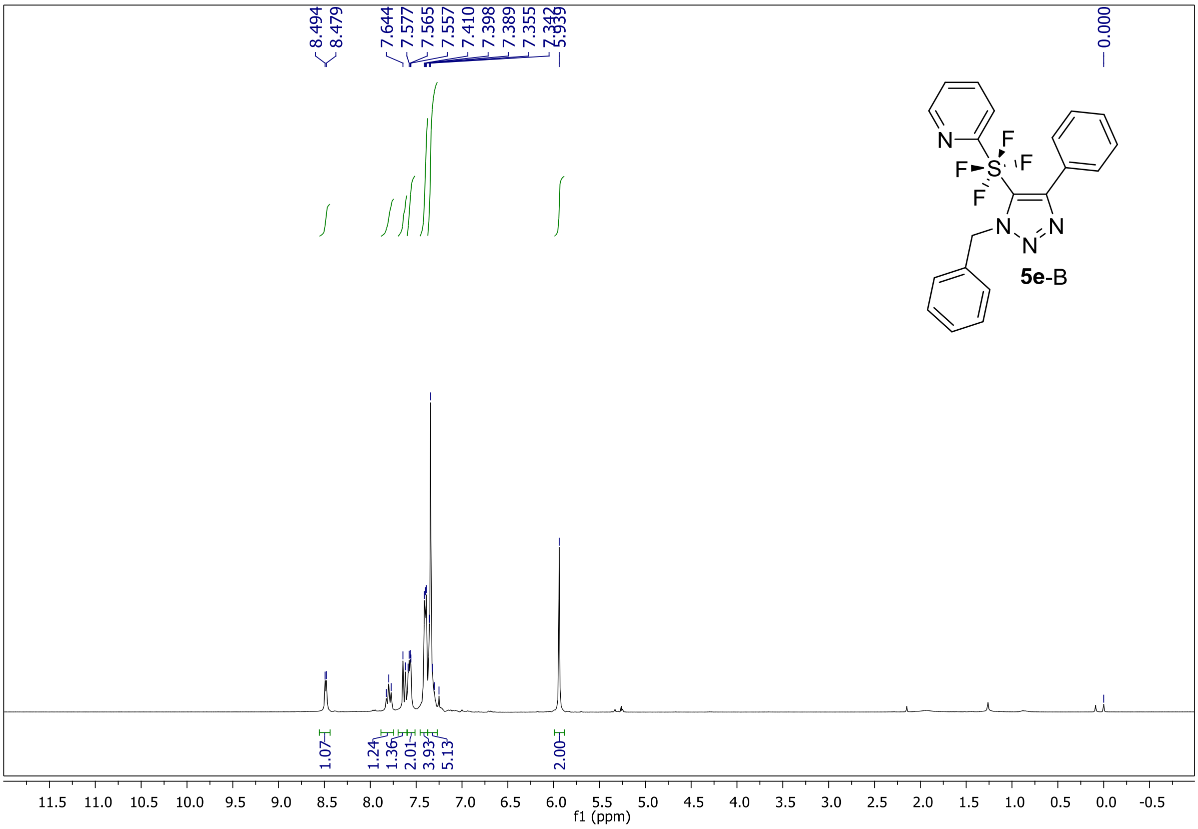
8.494
8.479
7.644
7.577
7.565
7.557
7.410
7.398
7.389
7.355
7.342
5.939
0.000
5e-B
1.07
1.24
1.36
2.01
3.93
5.13
2.00
11.5 11.0 10.5 10.0 9.5 9.0 8.5 8.0 7.5 7.0 6.5 6.0 5.5 5.0 4.5 4.0 3.5 3.0 2.5 2.0 1.5 1.0 0.5 0.0 -0.5
f1 (ppm)

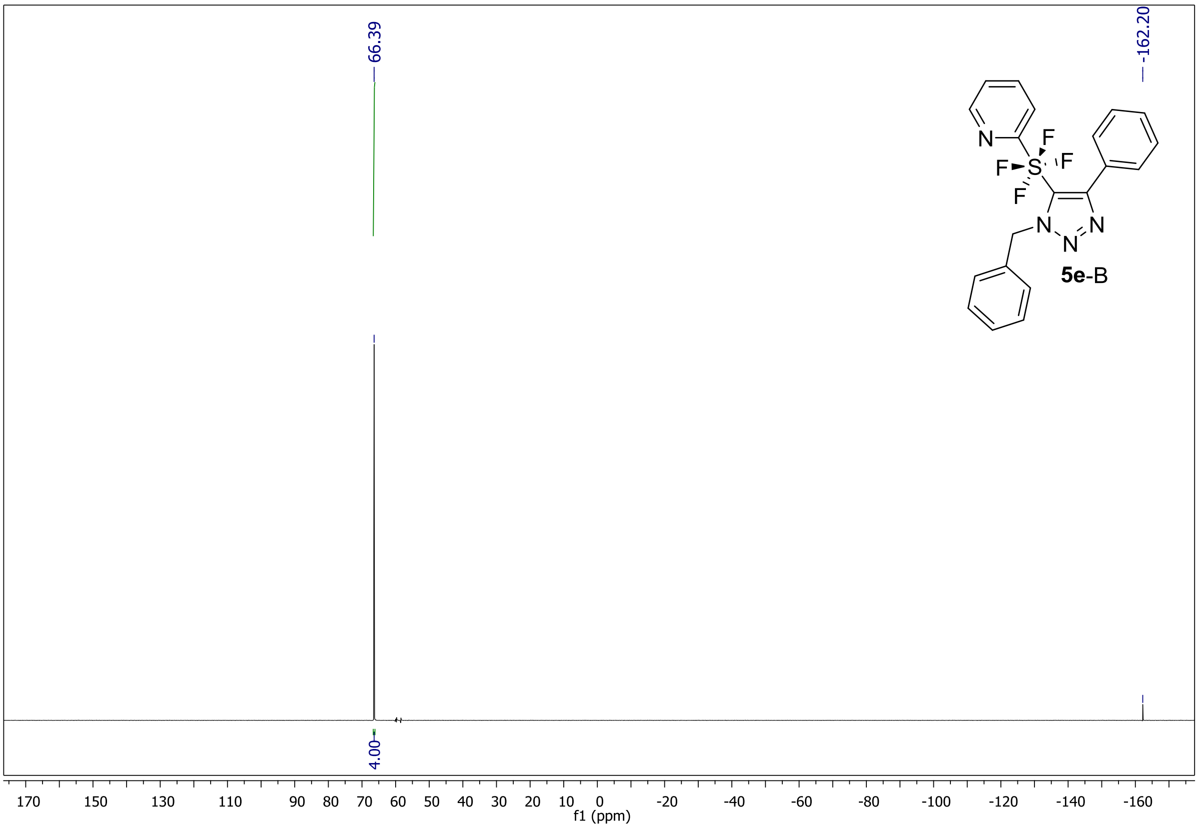

66.39
-162.20
4.00
5e-B
f1 (ppm)

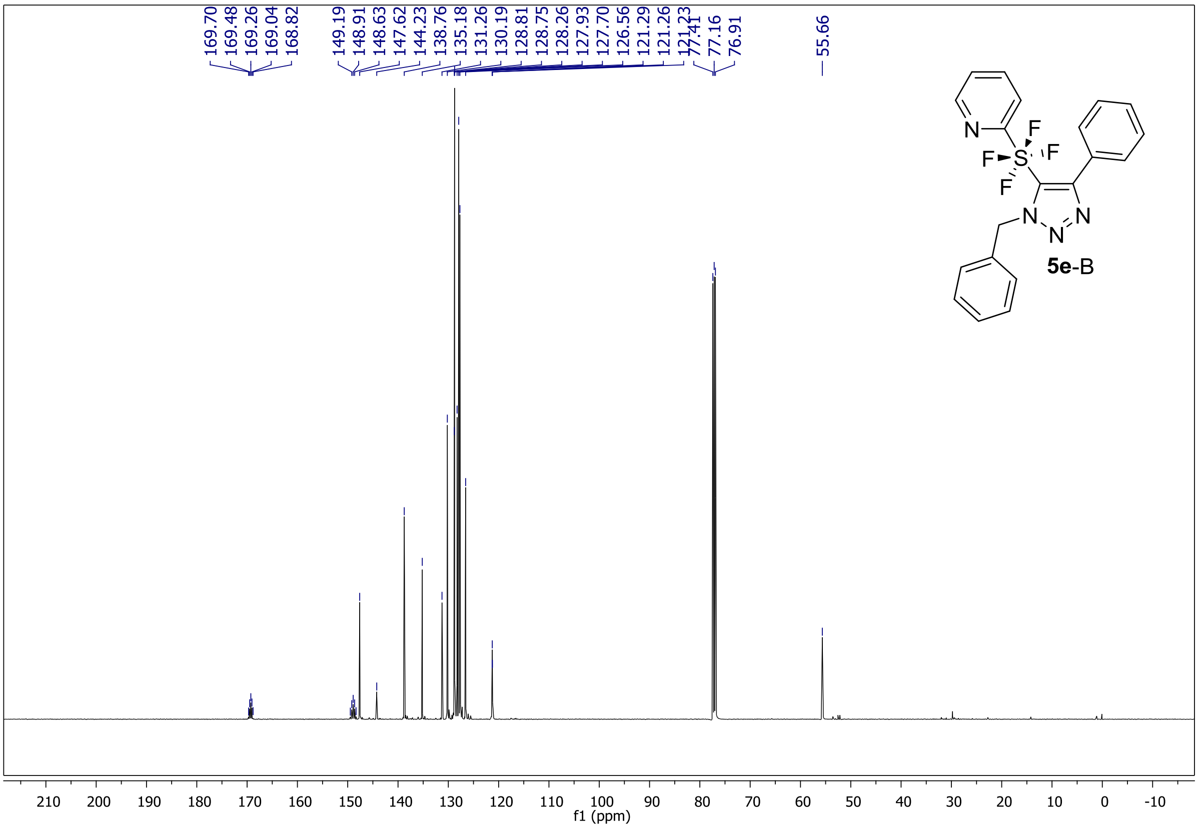

169.70
169.48
169.26
169.04
168.82
149.19
148.91
148.63
147.62
144.23
138.76
135.18
131.26
130.19
128.81
128.75
128.26
127.93
127.70
126.56
121.29
121.26
121.23
77.41
77.16
76.91
55.66
5e-B
f1 (ppm)

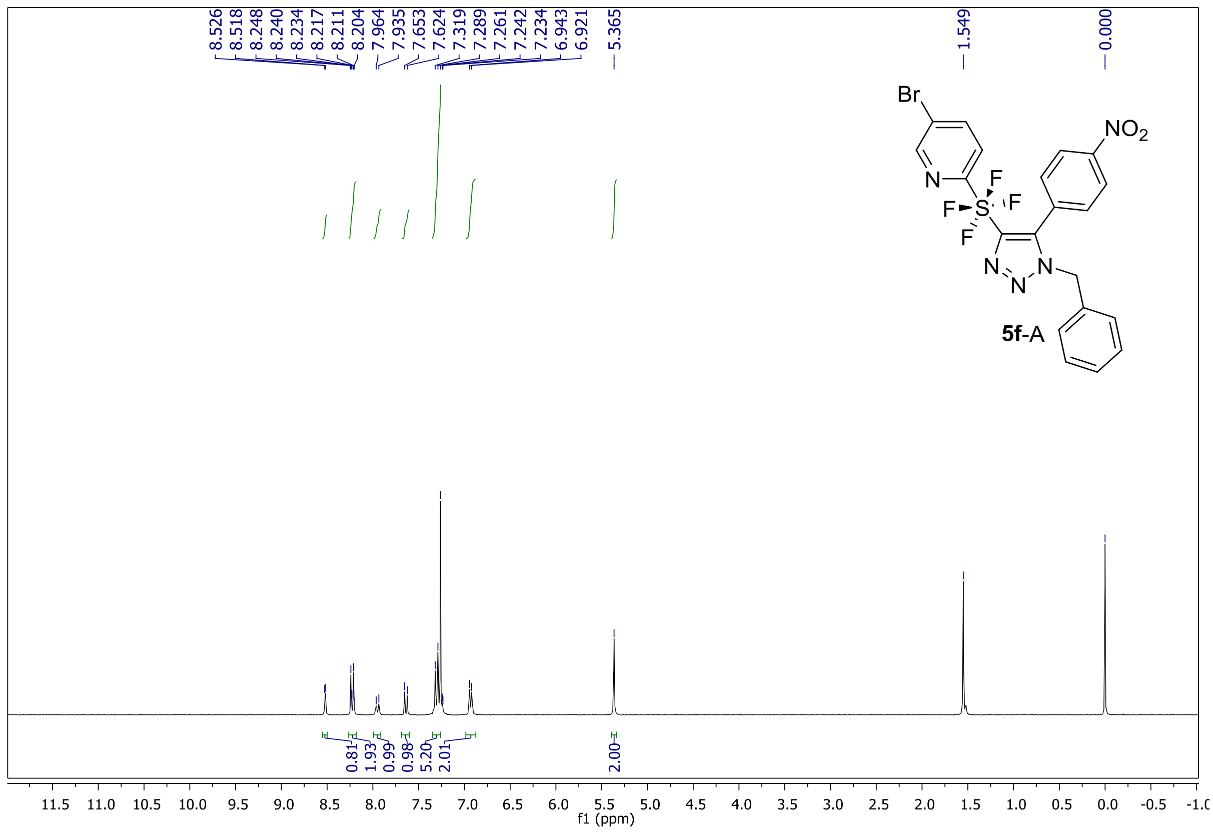
8.526
8.518
8.248
8.240
8.234
8.217
8.211
8.204
7.964
7.935
7.653
7.624
7.319
7.289
7.261
7.242
7.234
6.943
6.921
5.365
1.549
0.000
Br
NO2
N
F
F
F
S
F
N
N
N
5f-A
0.81
1.93
0.99
0.98
5.20
2.01
2.00
f1 (ppm)

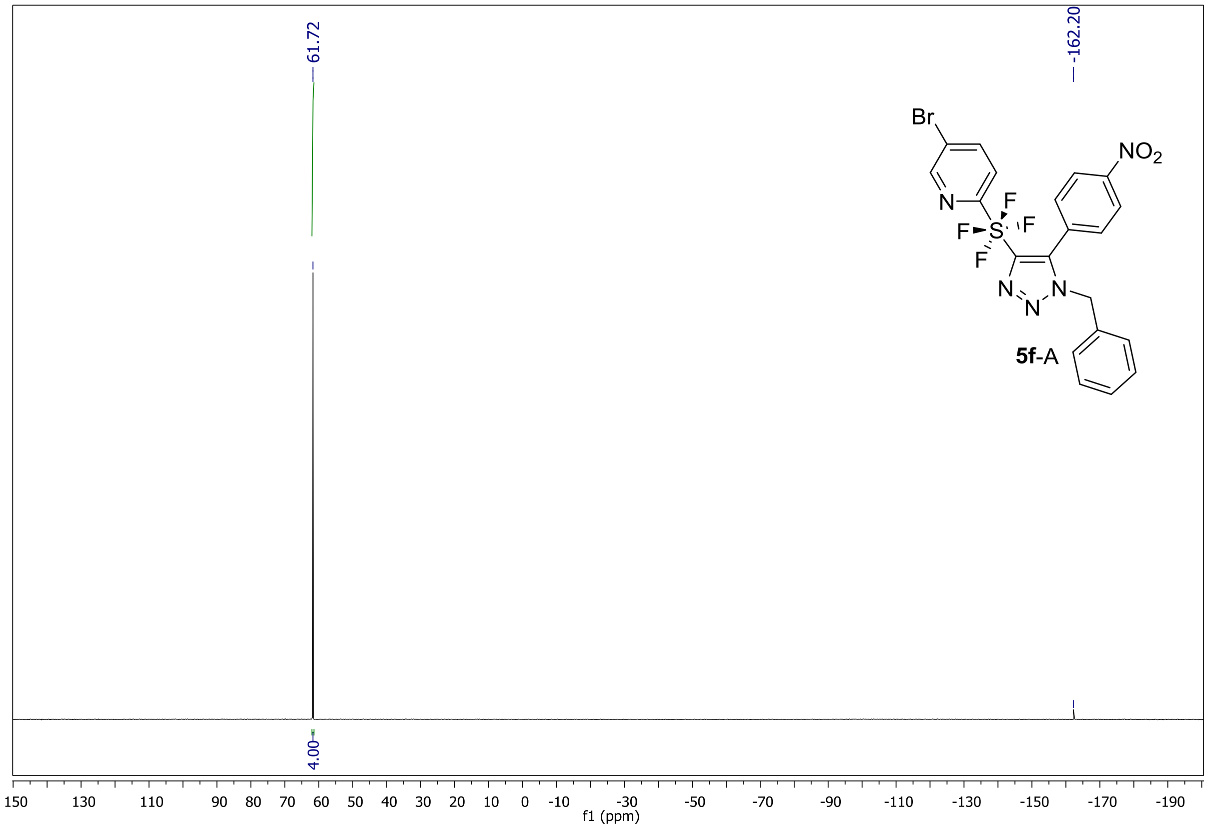
61.72
-162.20
Br
NO2
N
F
F
F
S
F
N
N
N
5f-A
4.00
f1 (ppm)

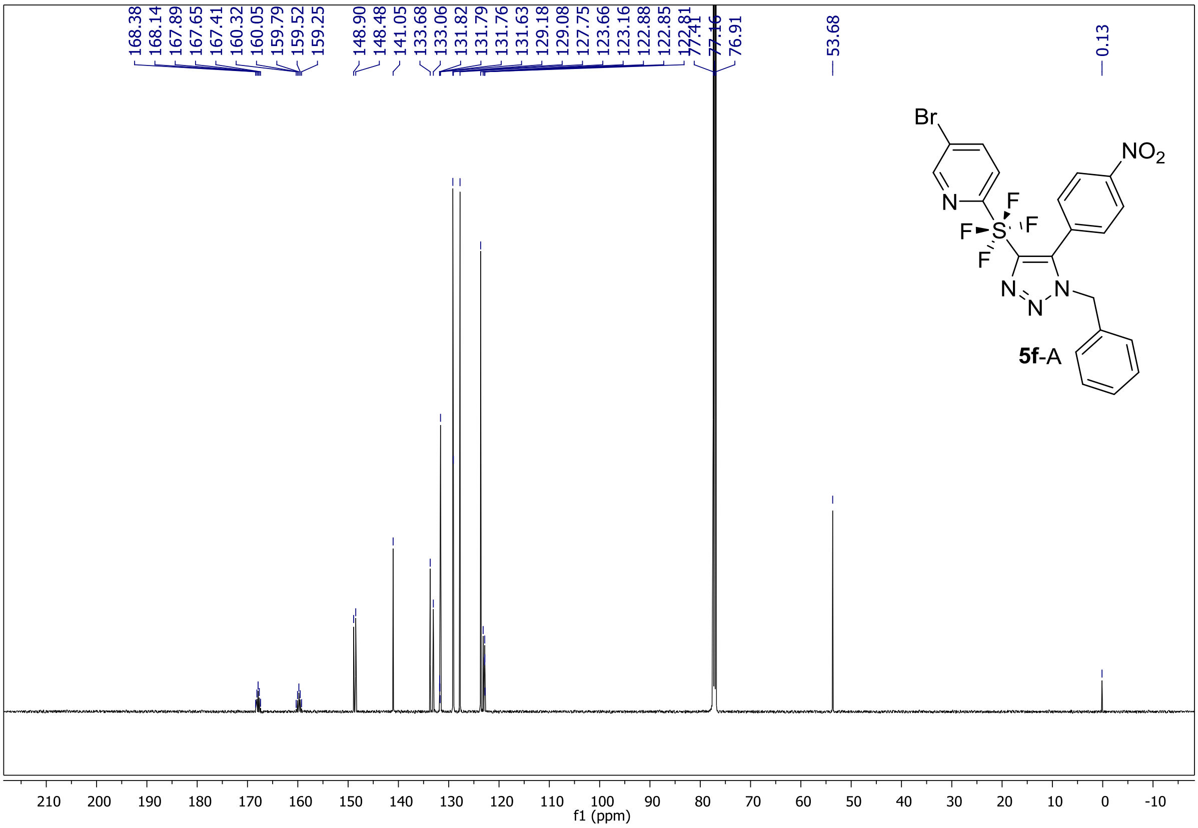
168.38
168.14
167.89
167.65
167.41
160.32
160.05
159.79
159.52
159.25
148.90
148.48
141.05
133.68
133.06
131.82
131.79
131.76
131.63
129.18
129.08
127.75
123.66
123.16
122.88
122.85
122.81
77.41
77.16
76.91
53.68
0.13
Br
NO2
N
F
F
F
S
F
N
N
N
5f-A
f1 (ppm)

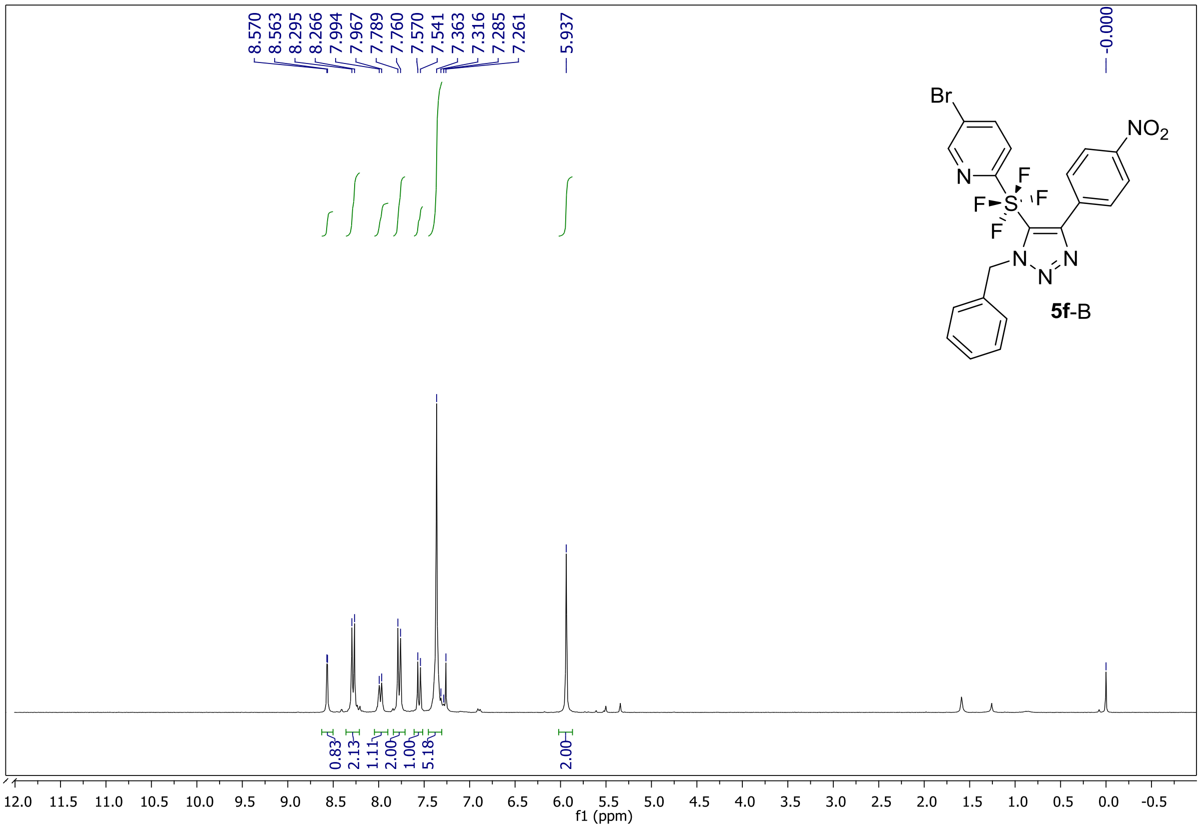
8.570
8.563
8.295
8.266
7.994
7.967
7.789
7.760
7.570
7.541
7.363
7.316
7.285
7.261
5.937
-0.000
Br
NO2
N
F
F
F
S
F
N
N
N
5f-B
0.83
2.13
1.11
2.00
1.00
5.18
2.00
f1 (ppm)

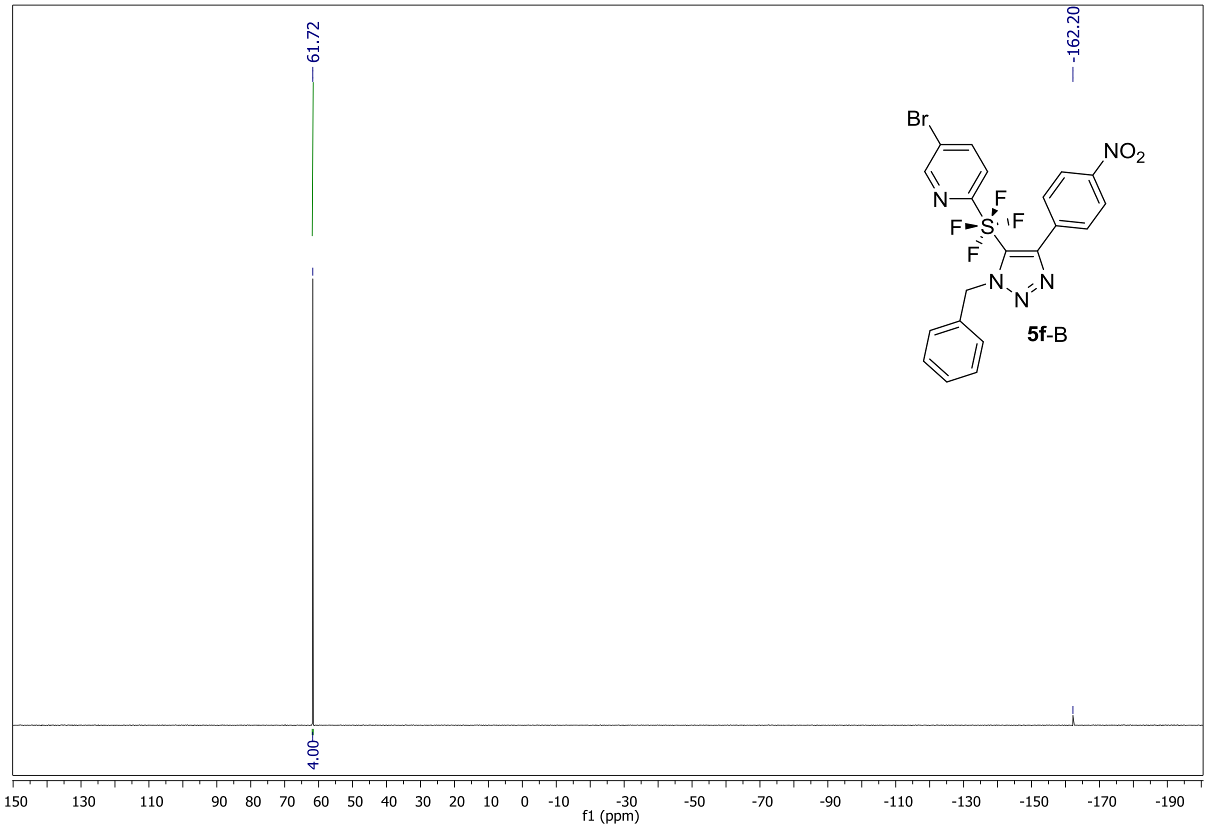
61.72
-162.20
Br
NO2
N
F
F
F
F
S
N
N
N
5f-B
4.00
150 130 110 90 80 70 60 50 40 30 20 10 0 -10 -30 -50 -70 -90 -110 -130 -150 -170 -190
f1 (ppm)

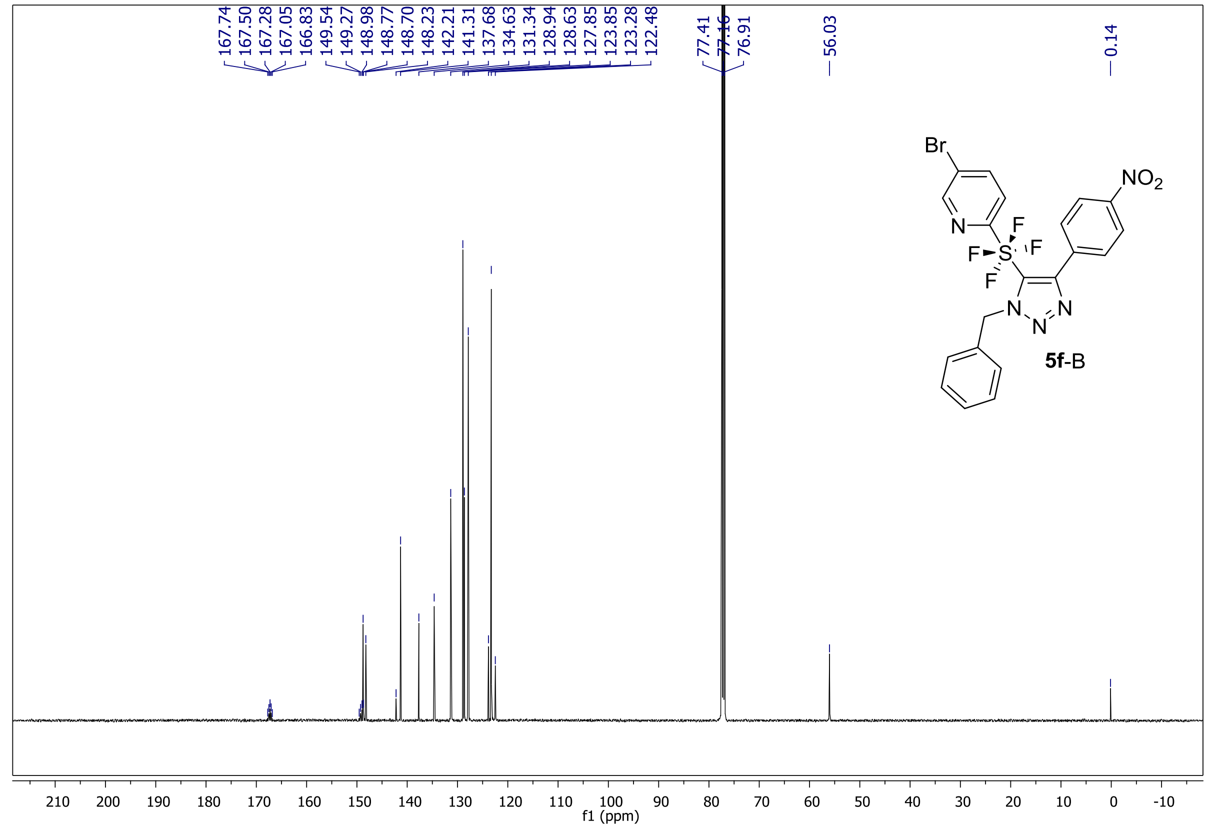
167.74
167.50
167.28
167.05
166.83
149.54
149.27
148.98
148.77
148.70
148.23
142.21
141.31
137.68
134.63
131.34
128.94
128.63
127.85
123.85
123.28
122.48
77.41
77.16
76.91
56.03
0.14
Br
NO2
N
F
F
F
F
S
N
N
N
5f-B
210 200 190 180 170 160 150 140 130 120 110 100 90 80 70 60 50 40 30 20 10 0 -10
f1 (ppm)

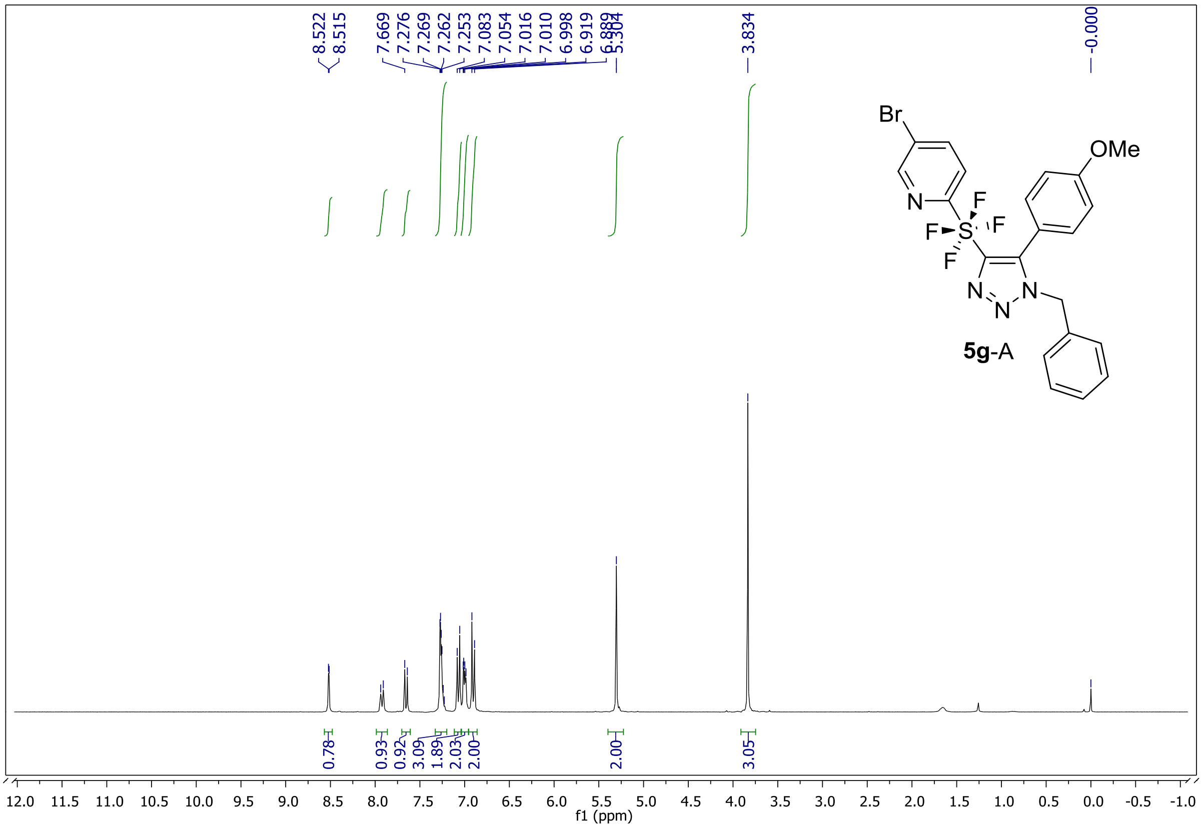
8.522
8.515
7.669
7.276
7.269
7.262
7.253
7.083
7.054
7.016
7.010
6.998
6.919
6.889
5.304
3.834
-0.000
Br
OMe
N
F
F
F
S
F
N
N
N
5g-A
0.78
0.93
0.92
3.09
1.89
2.03
2.00
2.00
3.05
12.0 11.5 11.0 10.5 10.0 9.5 9.0 8.5 8.0 7.5 7.0 6.5 6.0 5.5 5.0 4.5 4.0 3.5 3.0 2.5 2.0 1.5 1.0 0.5 0.0 -0.5 -1.0
f1 (ppm)

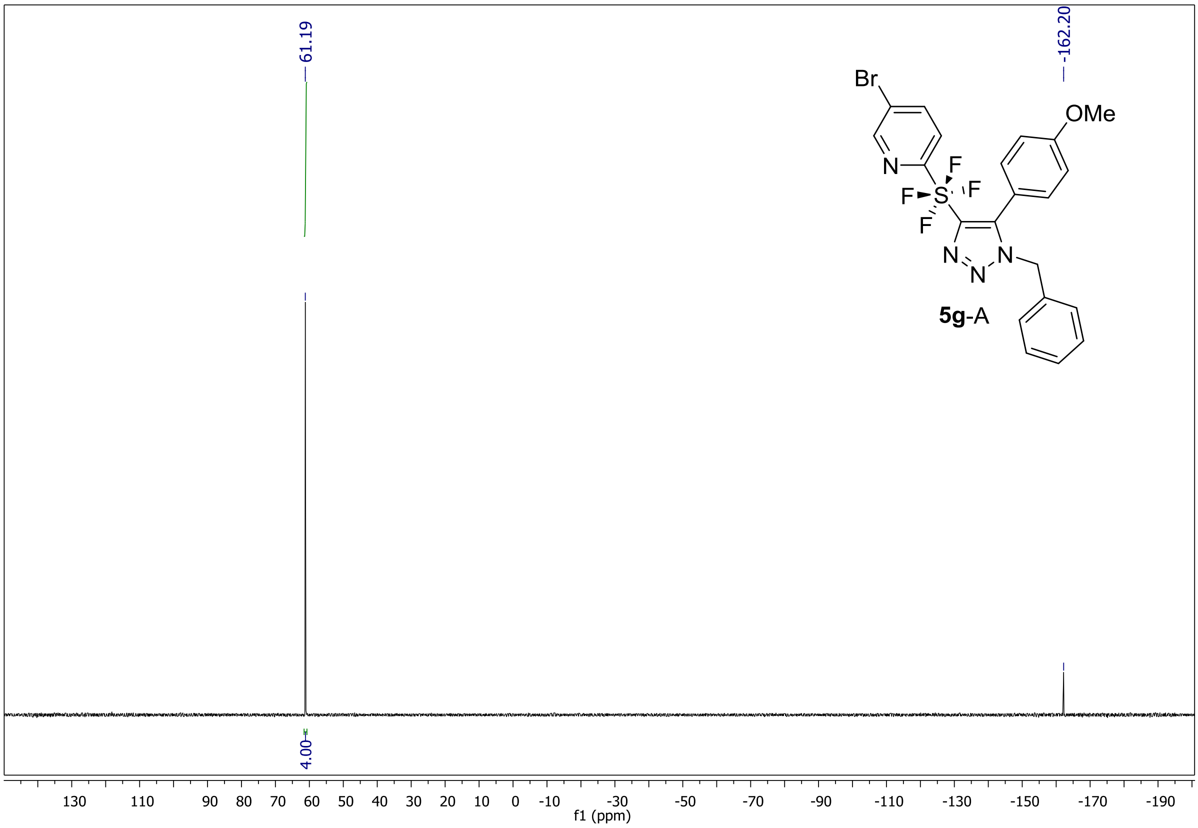
61.19
-162.20
Br
OMe
N
F
F
F
S
F
N
N
N
5g-A
4.00
130 110 90 80 70 60 50 40 30 20 10 0 -10 -30 -50 -70 -90 -110 -130 -150 -170 -190
f1 (ppm)

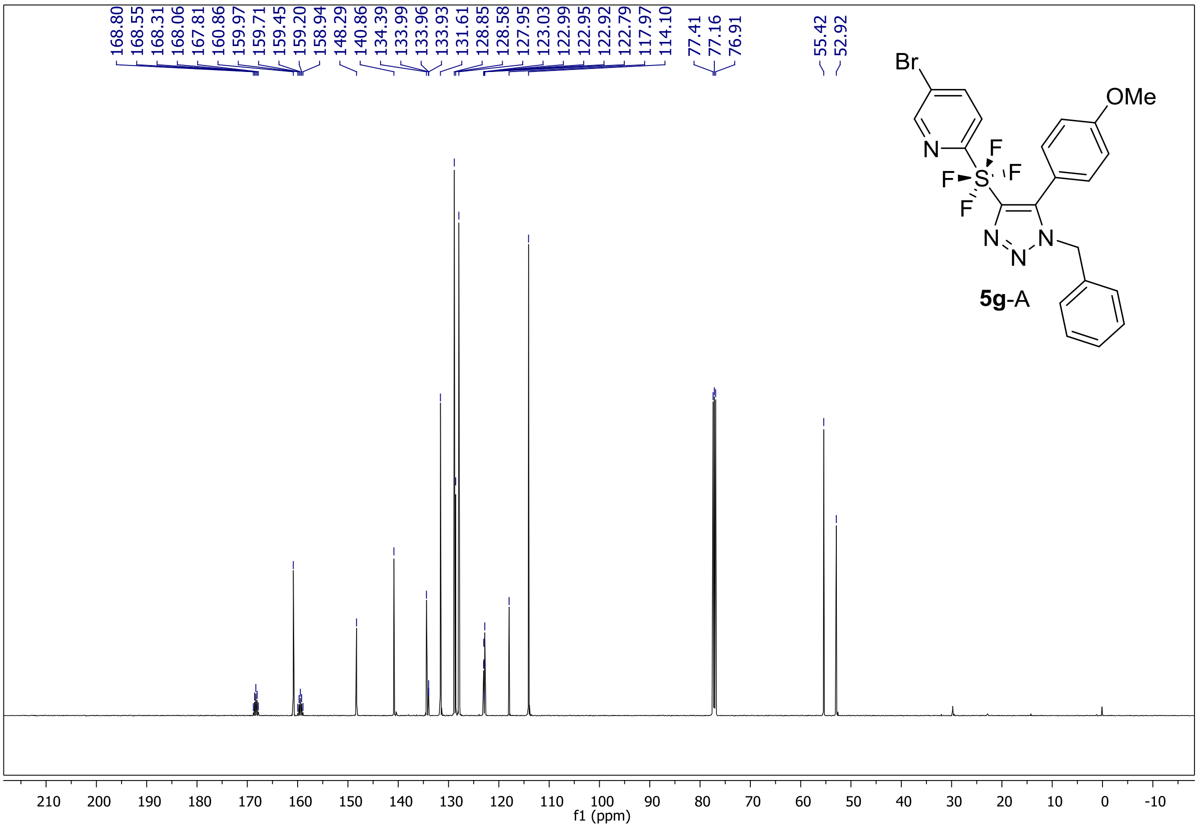
168.80
168.55
168.31
168.06
167.81
160.86
159.97
159.71
159.45
159.20
158.94
148.29
140.86
134.39
133.99
133.96
133.93
131.61
128.85
128.58
127.95
123.03
122.99
122.95
122.92
122.79
117.97
114.10
77.41
77.16
76.91
55.42
52.92
Br
OMe
N
F
F
S
F
F
N
N
N
5g-A
210 200 190 180 170 160 150 140 130 120 110 100 90 80 70 60 50 40 30 20 10 0 -10
f1 (ppm)

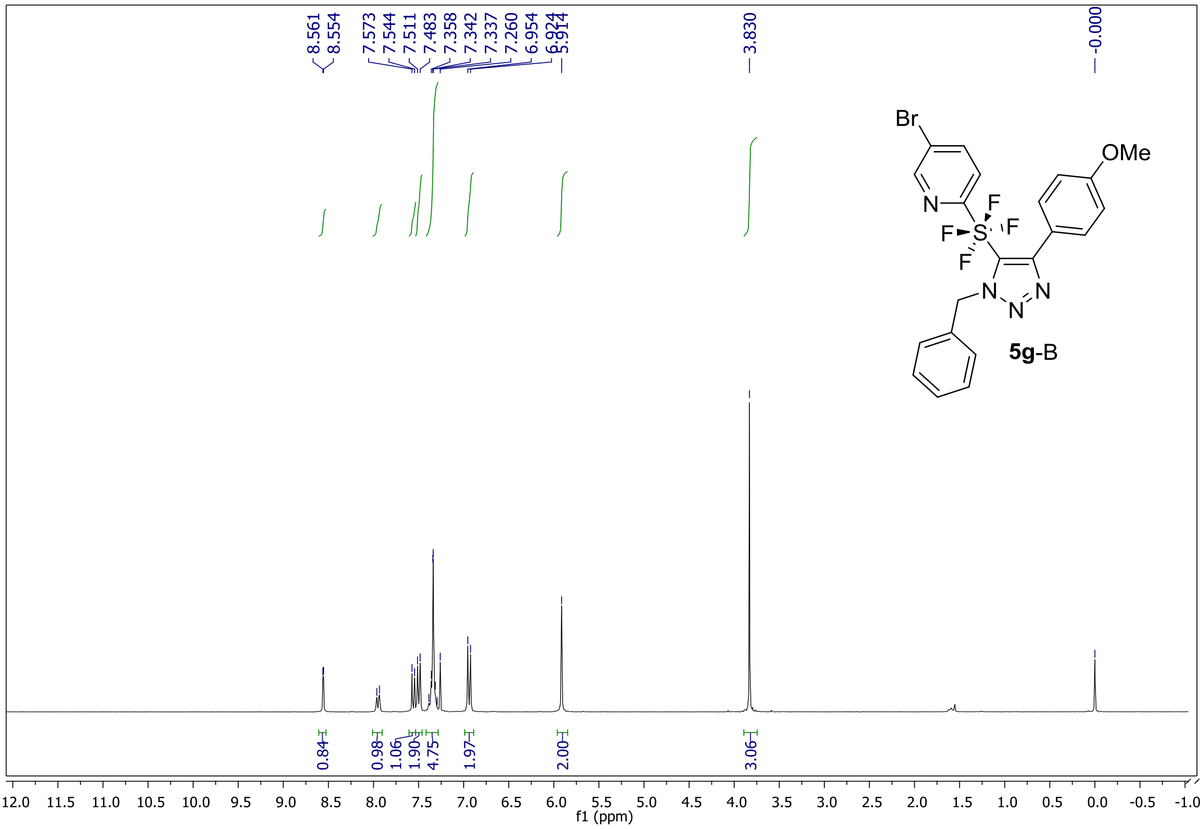
8.561
8.554
7.573
7.544
7.511
7.483
7.358
7.342
7.337
7.260
6.954
6.924
5.914
3.830
-0.000
Br
OMe
N
F
F
S
F
F
N
N
N
5g-B
0.84
0.98
1.06
1.90
4.75
1.97
2.00
3.06
12.0 11.5 11.0 10.5 10.0 9.5 9.0 8.5 8.0 7.5 7.0 6.5 6.0 5.5 5.0 4.5 4.0 3.5 3.0 2.5 2.0 1.5 1.0 0.5 0.0 -0.5 -1.0
f1 (ppm)

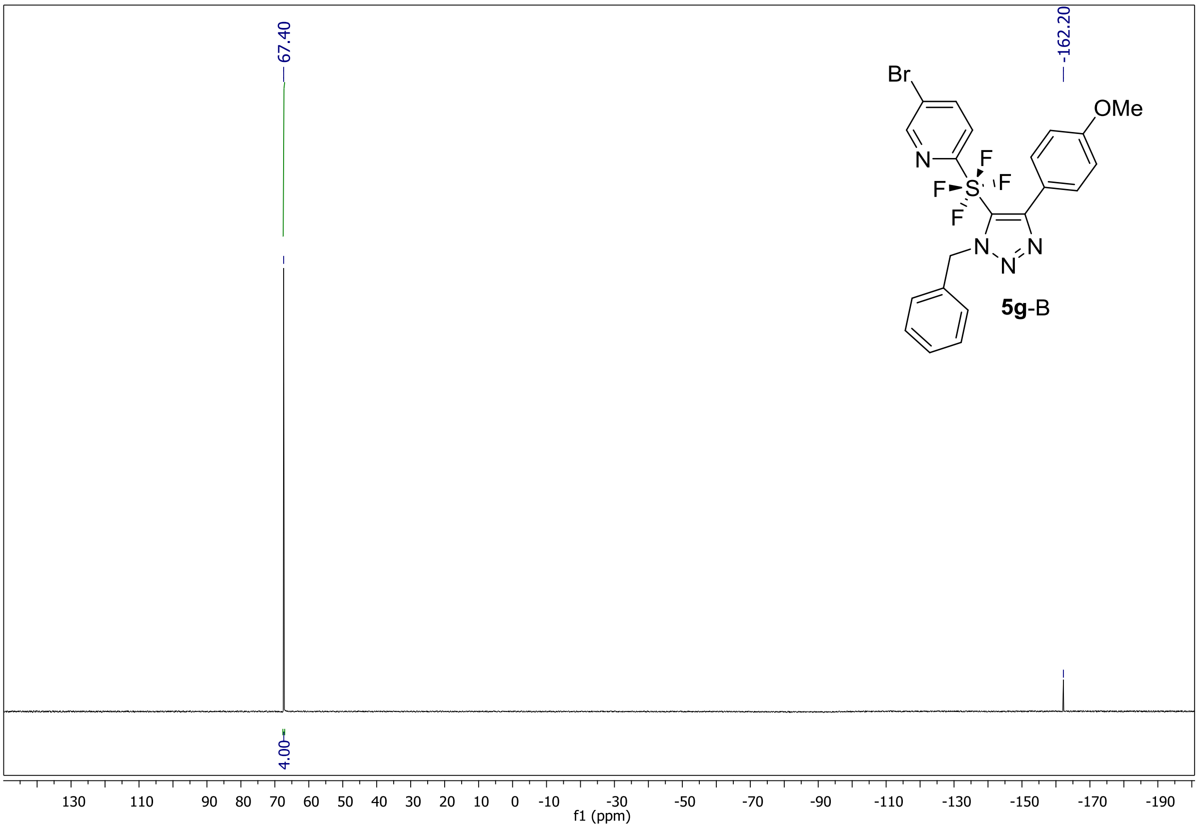
67.40
-162.20
4.00
Br
N
F
F
F
F
S
OMe
N
N
N
5g-B
130 110 90 80 70 60 50 40 30 20 10 0 -10 -30 -50 -70 -90 -110 -130 -150 -170 -190
f1 (ppm)

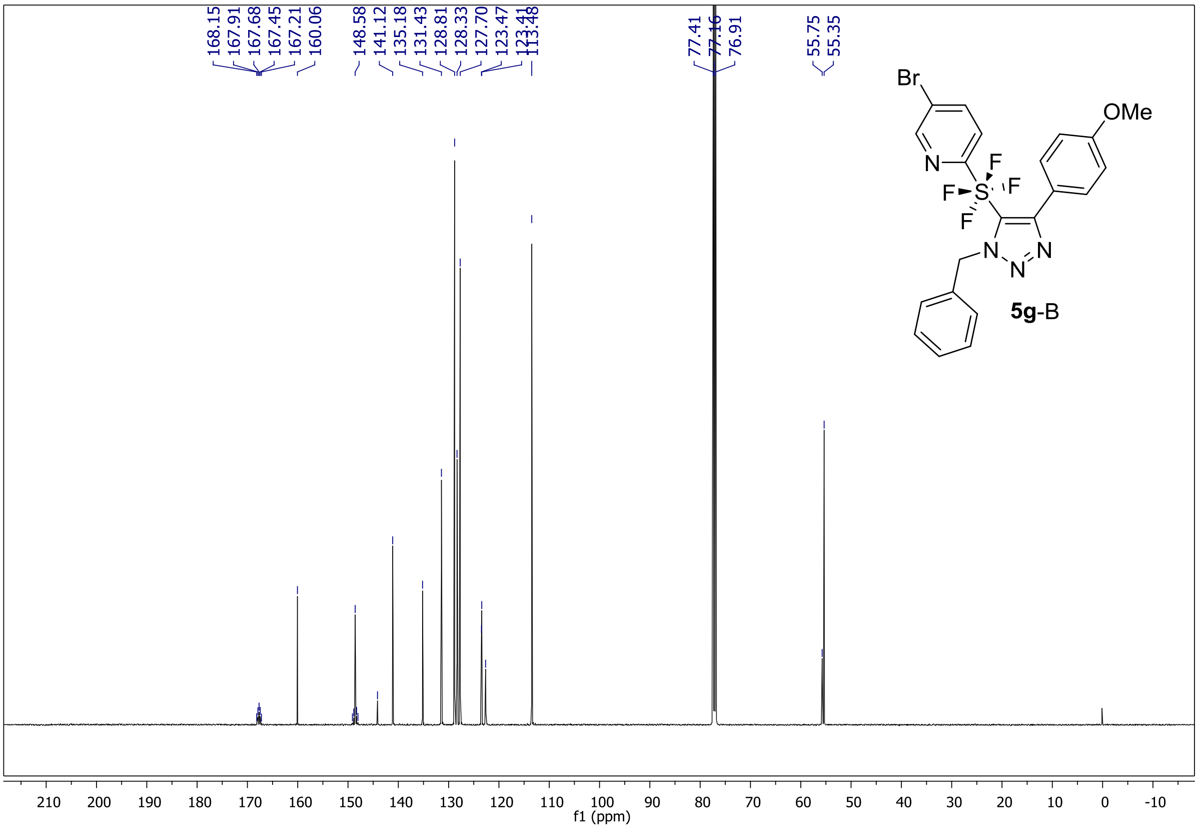
168.15
167.91
167.68
167.45
167.21
160.06
148.58
141.12
135.18
131.43
128.81
128.33
127.70
123.47
123.41
113.48
77.41
77.16
76.91
55.75
55.35
Br
N
F
F
F
F
S
OMe
N
N
N
5g-B
210 200 190 180 170 160 150 140 130 120 110 100 90 80 70 60 50 40 30 20 10 0 -10
f1 (ppm)

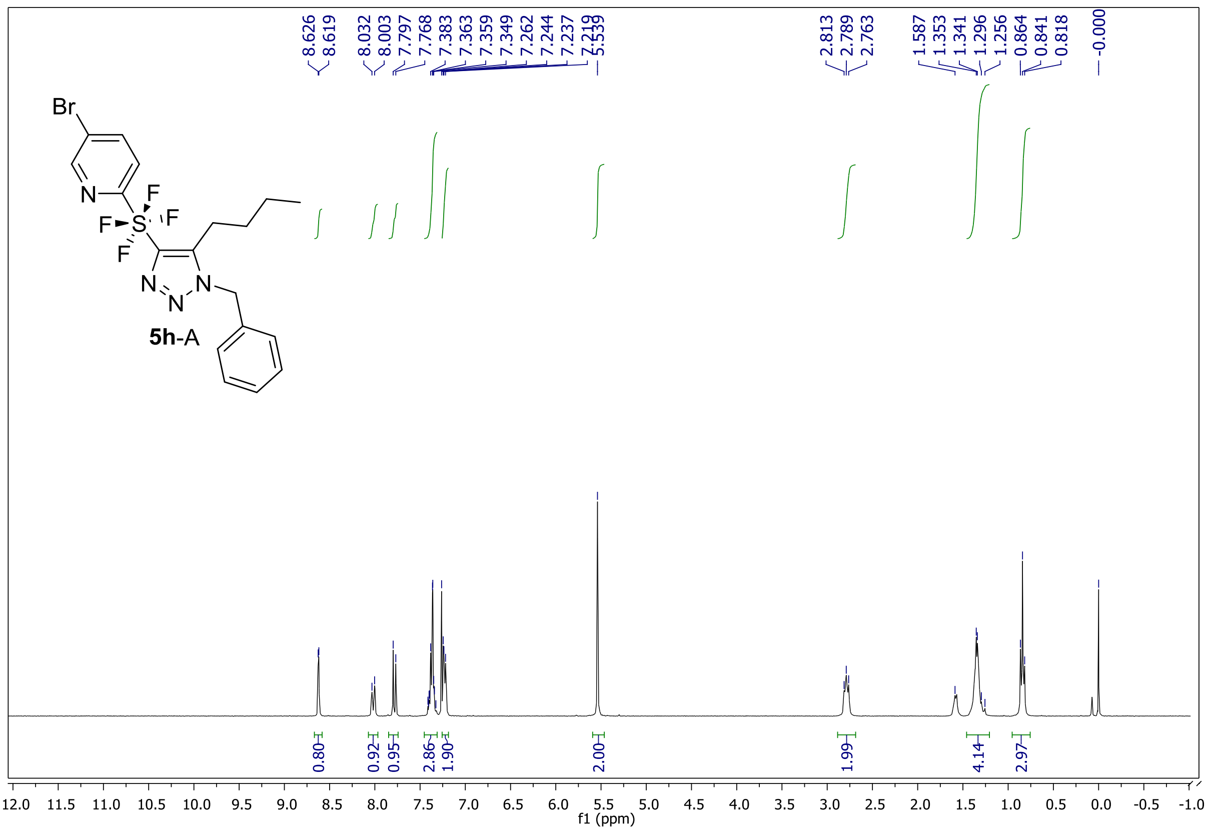

8.626
8.619
8.032
8.003
7.797
7.768
7.383
7.363
7.359
7.349
7.262
7.244
7.237
7.219
5.539
2.813
2.789
2.763
1.587
1.353
1.341
1.296
1.256
0.864
0.841
0.818
-0.000
Br
N
F
F
F
F
S
N
N
N
5h-A
0.80
0.92
0.95
2.86
1.90
2.00
1.99
4.14
2.97
12.0 11.5 11.0 10.5 10.0 9.5 9.0 8.5 8.0 7.5 7.0 6.5 6.0 5.5 5.0 4.5 4.0 3.5 3.0 2.5 2.0 1.5 1.0 0.5 0.0 -0.5 -1.0
f1 (ppm)

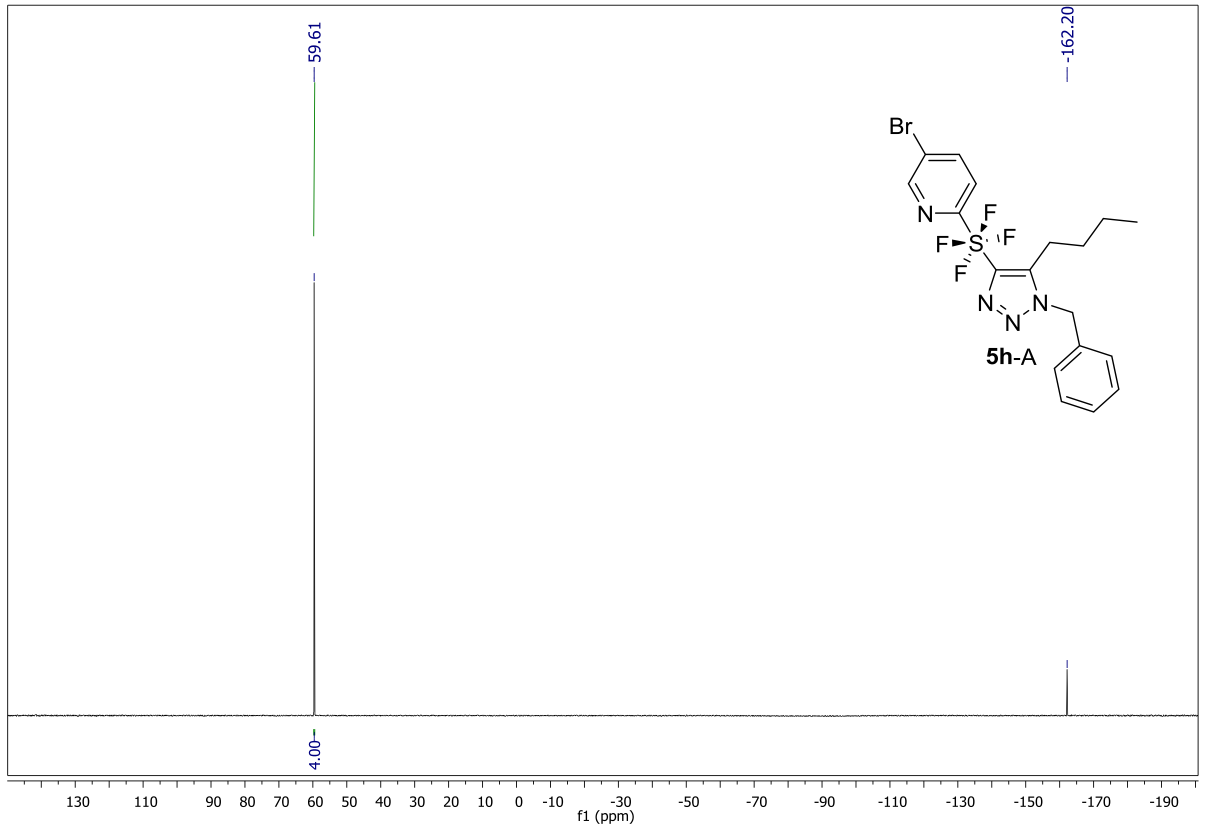

59.61
-162.20
Br
N
F
F
F
F
S
N
N
N
5h-A
4.00
130 110 90 80 70 60 50 40 30 20 10 0 -10 -30 -50 -70 -90 -110 -130 -150 -170 -190
f1 (ppm)

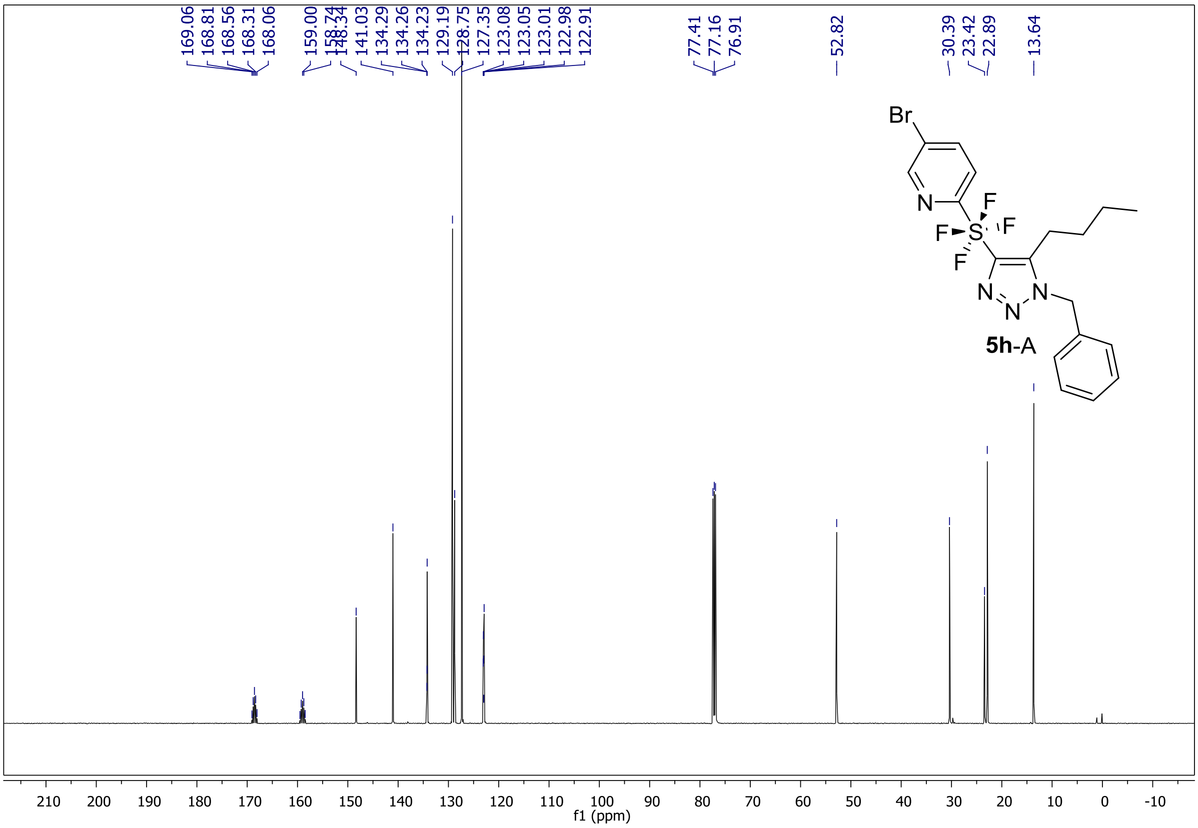

169.06
168.81
168.56
168.31
168.06
159.00
158.74
158.34
141.03
134.29
134.26
134.23
129.19
128.75
127.35
123.08
123.05
123.01
122.98
122.91
77.41
77.16
76.91
52.82
30.39
23.42
22.89
13.64
Br
N
F
F
F
S
F
N
N
N
5h-A
f1 (ppm)

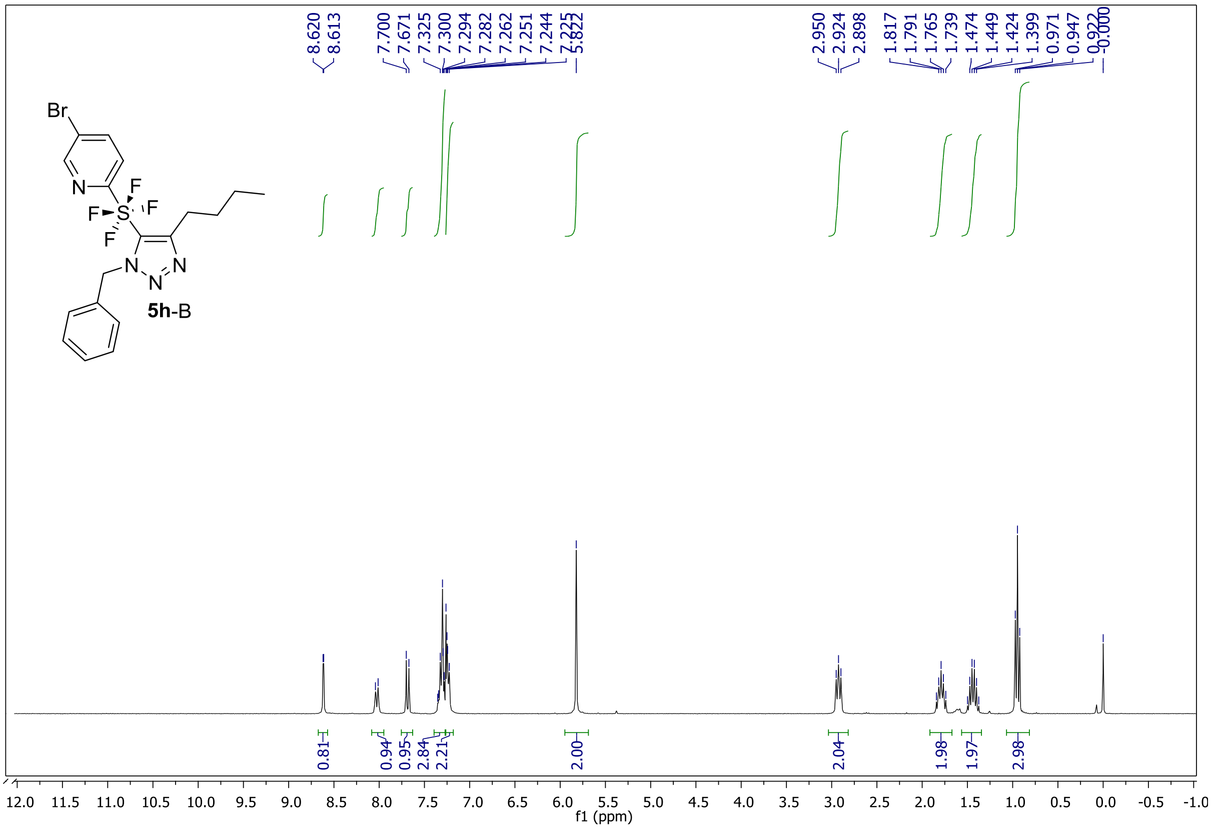

8.620
8.613
7.700
7.671
7.325
7.300
7.294
7.282
7.262
7.251
7.244
7.225
5.822
2.950
2.924
2.898
1.817
1.791
1.765
1.739
1.474
1.449
1.424
1.399
0.971
0.947
0.922
-0.000
Br
N
F
F
F
S
F
N
N
N
5h-B
0.81
0.94
0.95
2.84
2.21
2.00
2.04
1.98
1.97
2.98
f1 (ppm)

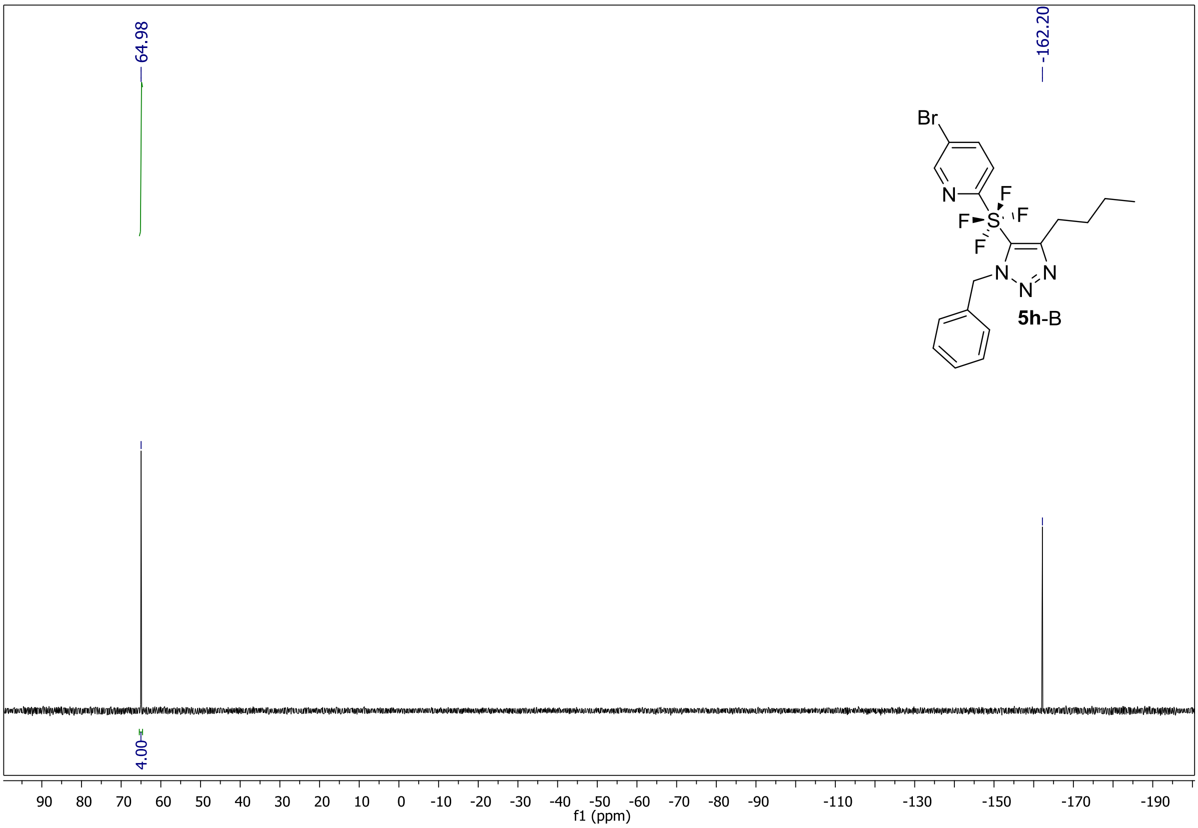
64.98
-162.20
4.00
Br
N
F
F
S
F
F
N
N
N
5h-B
f1 (ppm)

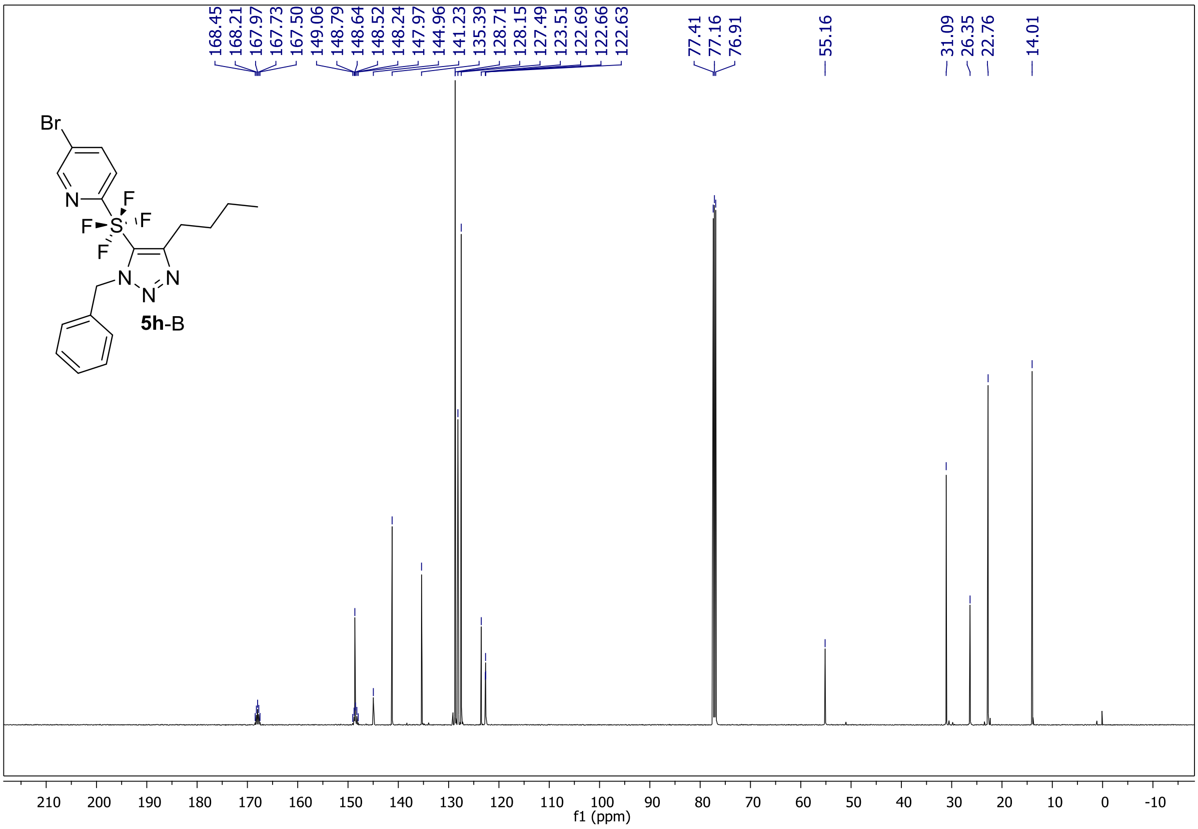
168.45
168.21
167.97
167.73
167.50
149.06
148.79
148.64
148.52
148.24
147.97
144.96
141.23
135.39
128.71
128.15
127.49
123.51
122.69
122.66
122.63
77.41
77.16
76.91
55.16
31.09
26.35
22.76
14.01
Br
N
F
F
S
F
F
N
N
N
5h-B
f1 (ppm)

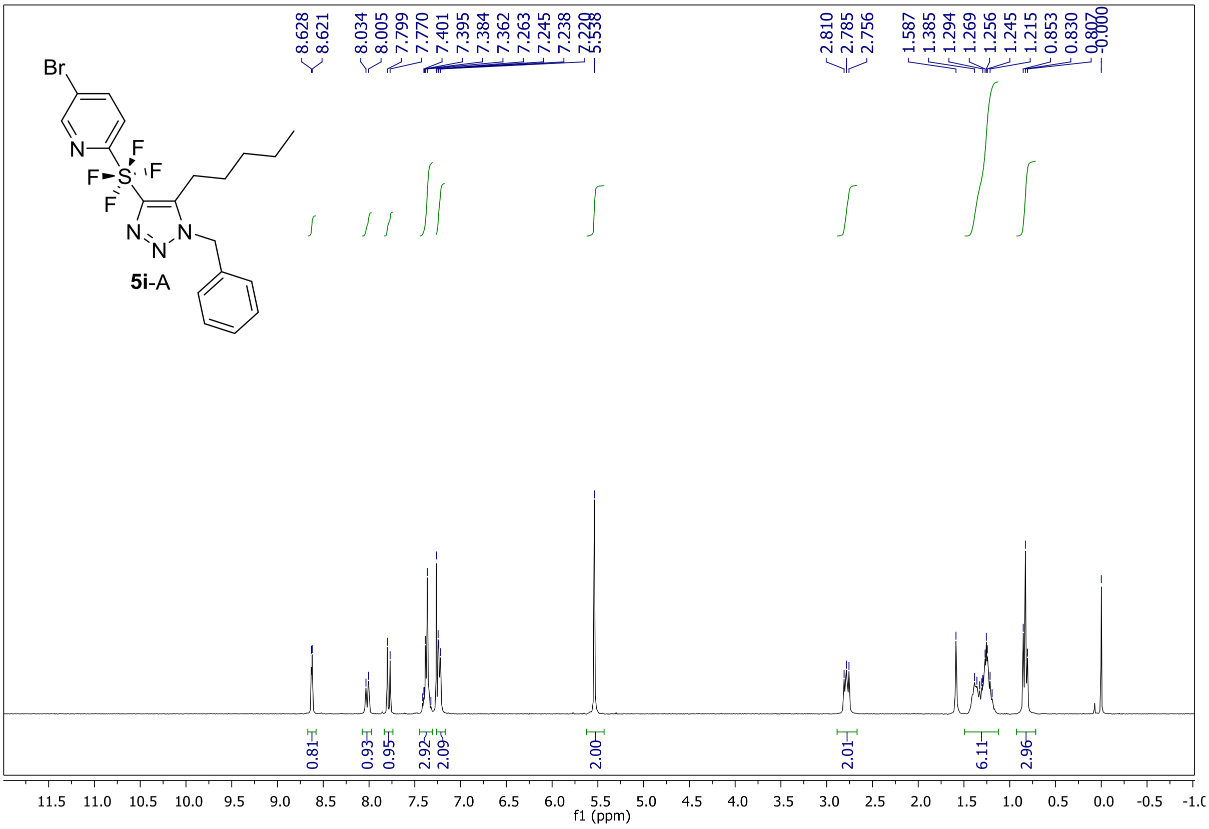
8.628
8.621
8.034
8.005
7.799
7.770
7.401
7.395
7.384
7.362
7.263
7.245
7.238
7.220
5.538
2.810
2.785
2.756
1.587
1.385
1.294
1.269
1.256
1.245
1.215
0.853
0.830
0.807
-0.000
Br
N
F
F
F
F
S
N
N
N
5i-A
0.81
0.93
0.95
2.92
2.09
2.00
2.01
6.11
2.96
f1 (ppm)

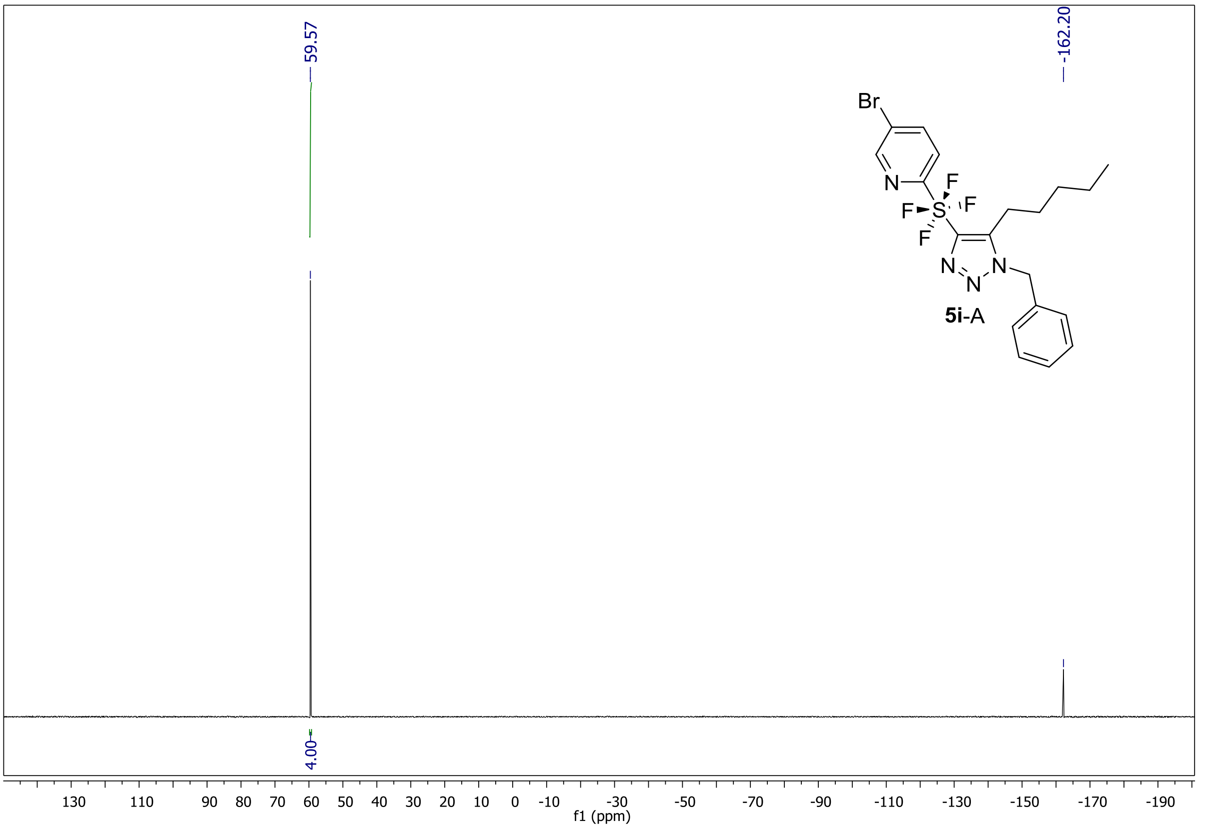
59.57
-162.20
Br
N
F
F
F
F
S
N
N
N
5i-A
4.00
f1 (ppm)

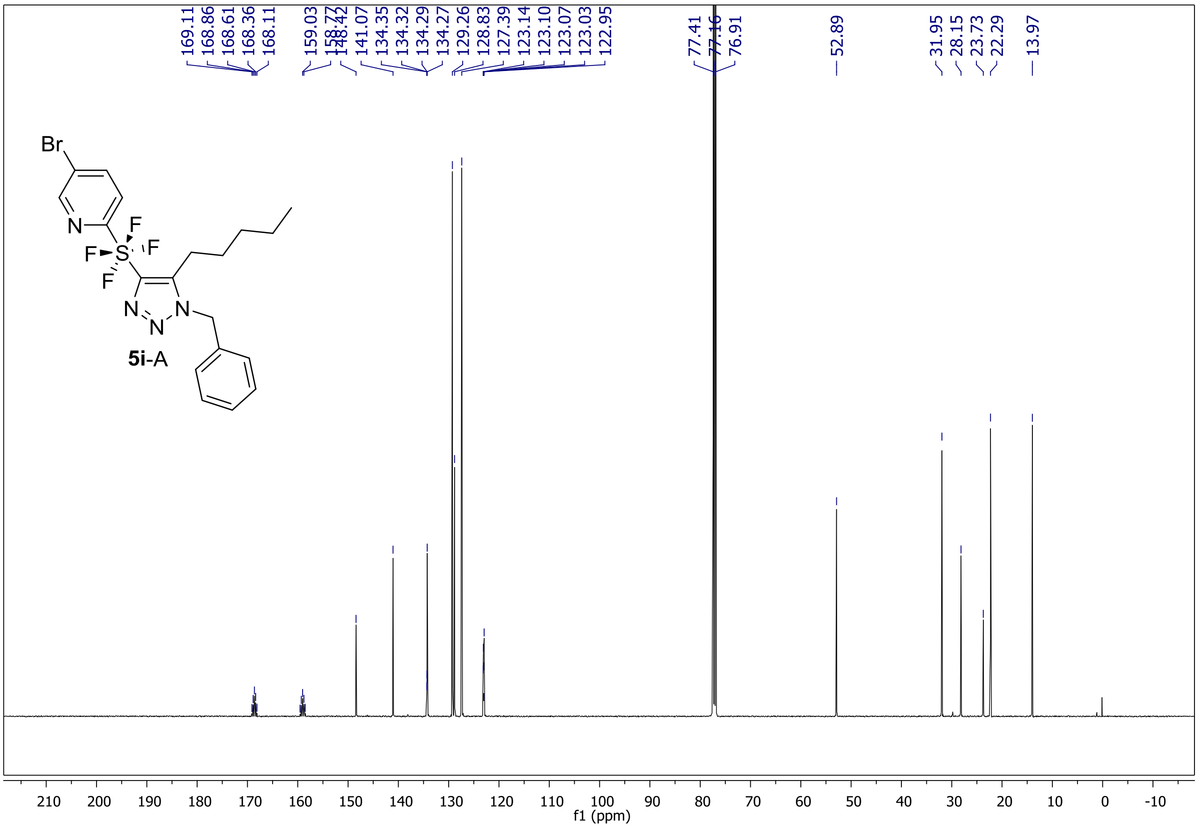
169.11
168.86
168.61
168.36
168.11
159.03
158.77
148.42
141.07
134.35
134.32
134.29
134.27
129.26
128.83
127.39
123.14
123.10
123.07
123.03
122.95
77.41
77.16
76.91
52.89
31.95
28.15
23.73
22.29
13.97
Br
N
F
F
F
F
S
N
N
N
5i-A
f1 (ppm)

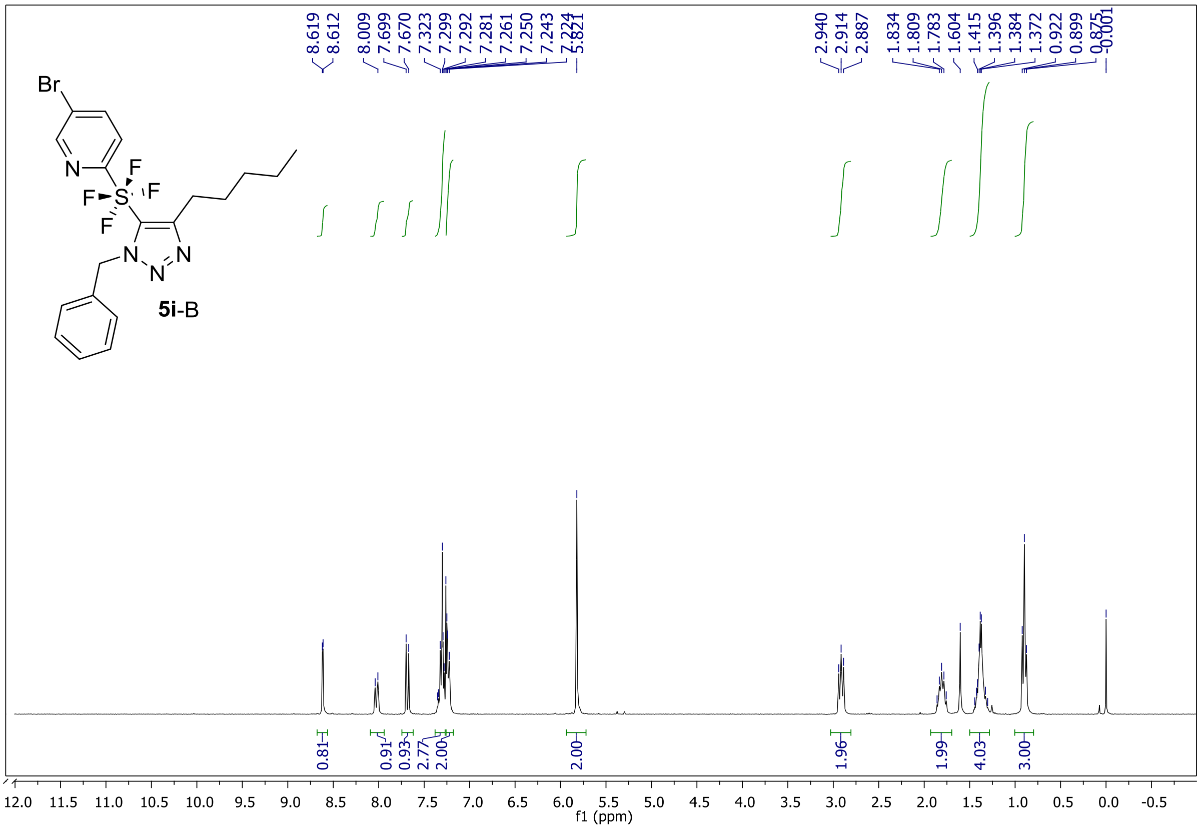
8.619
8.612
8.009
7.699
7.670
7.323
7.299
7.292
7.281
7.261
7.250
7.243
7.224
5.821
2.940
2.914
2.887
1.834
1.809
1.783
1.604
1.415
1.396
1.384
1.372
0.922
0.899
0.875
-0.001
Br
N
F
F
F
F
S
N
N
N
5i-B
0.81
0.91
0.93
2.77
2.00
2.00
1.96
1.99
4.03
3.00
f1 (ppm)

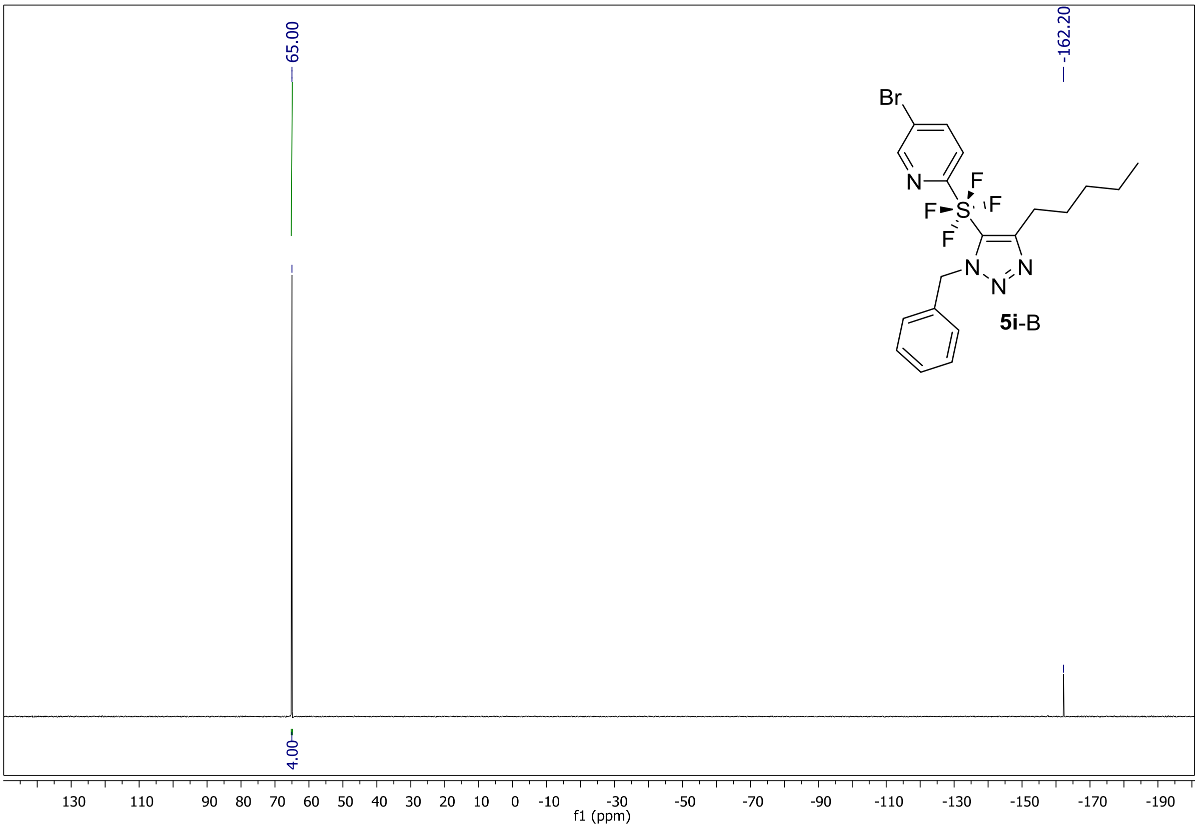
65.00
-162.20
Br
N
F
F
F
S
F
N
N
N
5i-B
4.00
130
110
90
80
70
60
50
40
30
20
10
0
-10
-30
-50
-70
-90
-110
-130
-150
-170
-190
f1 (ppm)

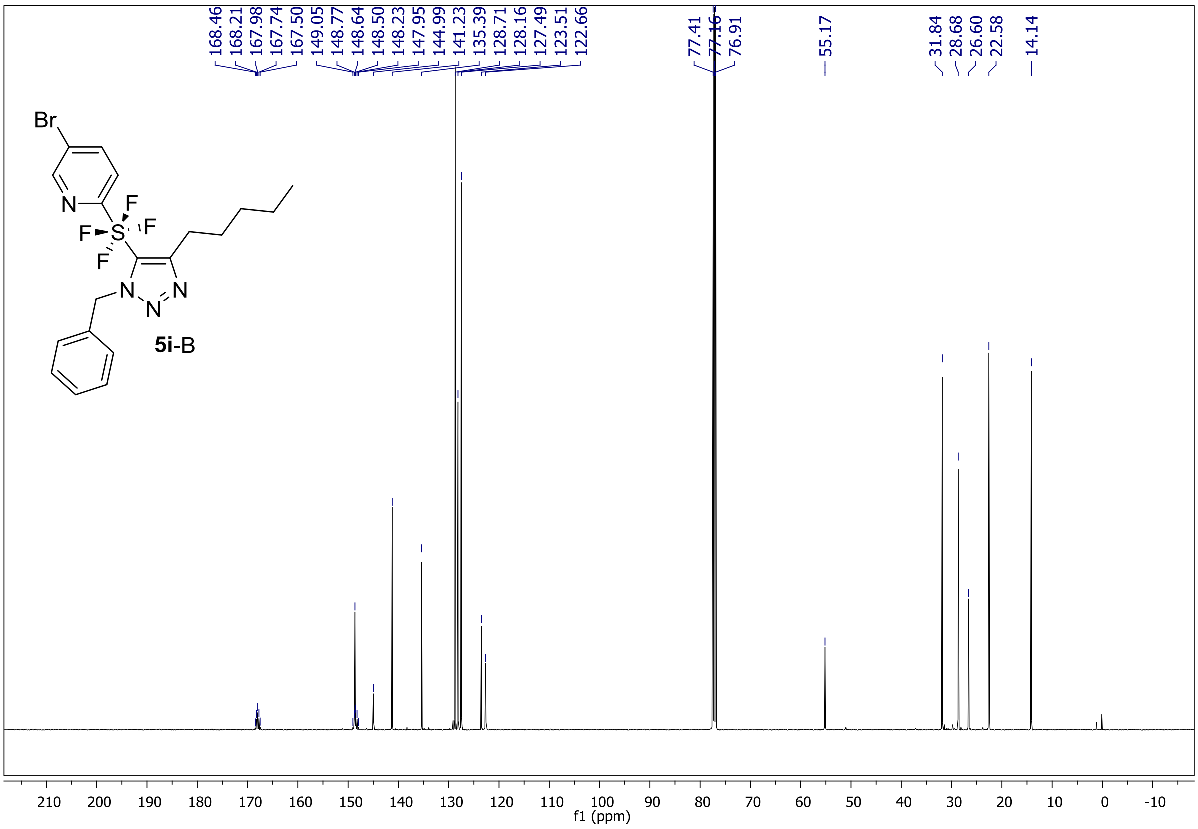
168.46
168.21
167.98
167.74
167.50
149.05
148.77
148.64
148.50
148.23
147.95
144.99
141.23
135.39
128.71
128.16
127.49
123.51
122.66
77.41
77.16
76.91
55.17
31.84
28.68
26.60
22.58
14.14
Br
N
F
F
F
S
F
N
N
N
5i-B
210
200
190
180
170
160
150
140
130
120
110
100
90
80
70
60
50
40
30
20
10
0
-10
f1 (ppm)

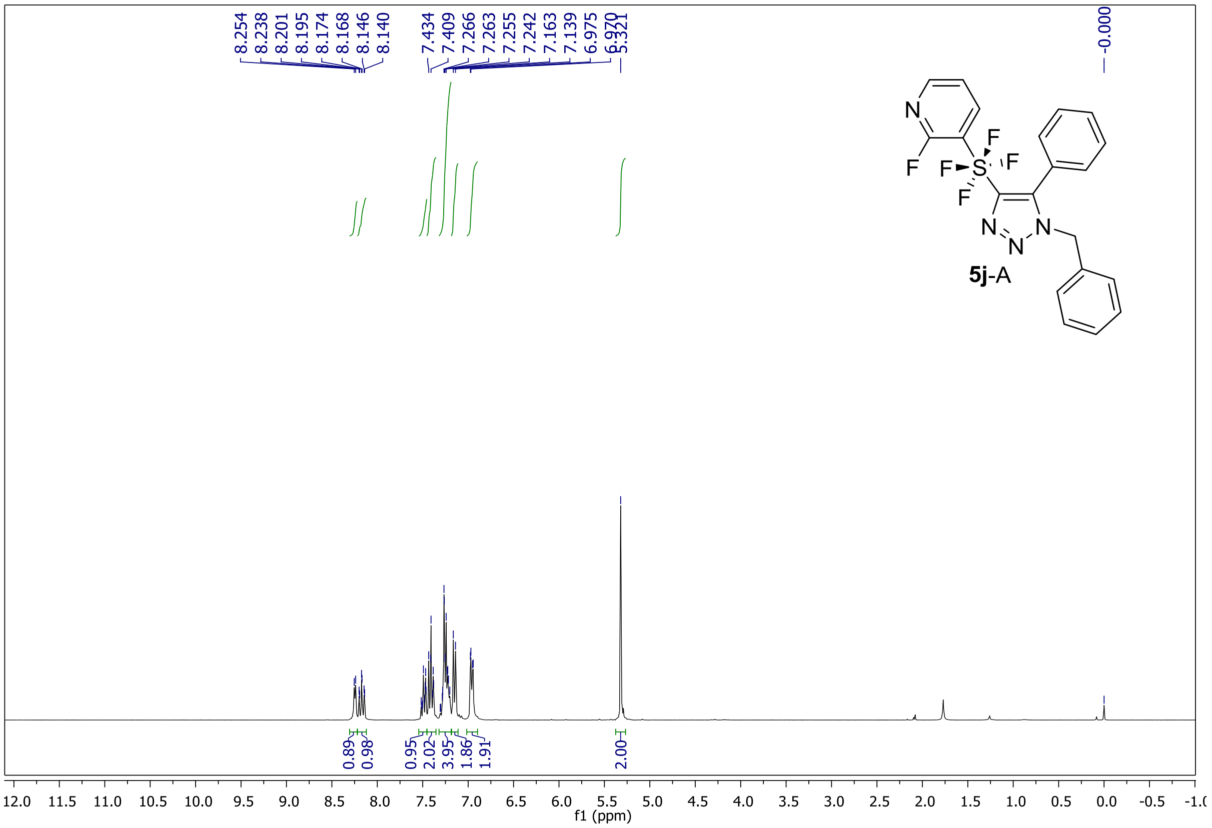
8.254
8.238
8.201
8.195
8.174
8.168
8.146
8.140
7.434
7.409
7.266
7.263
7.255
7.242
7.163
7.139
6.975
6.970
5.321
-0.000
5j-A
0.89
0.98
0.95
2.02
3.95
1.86
1.91
2.00
12.0 11.5 11.0 10.5 10.0 9.5 9.0 8.5 8.0 7.5 7.0 6.5 6.0 5.5 5.0 4.5 4.0 3.5 3.0 2.5 2.0 1.5 1.0 0.5 0.0 -0.5
f1 (ppm)

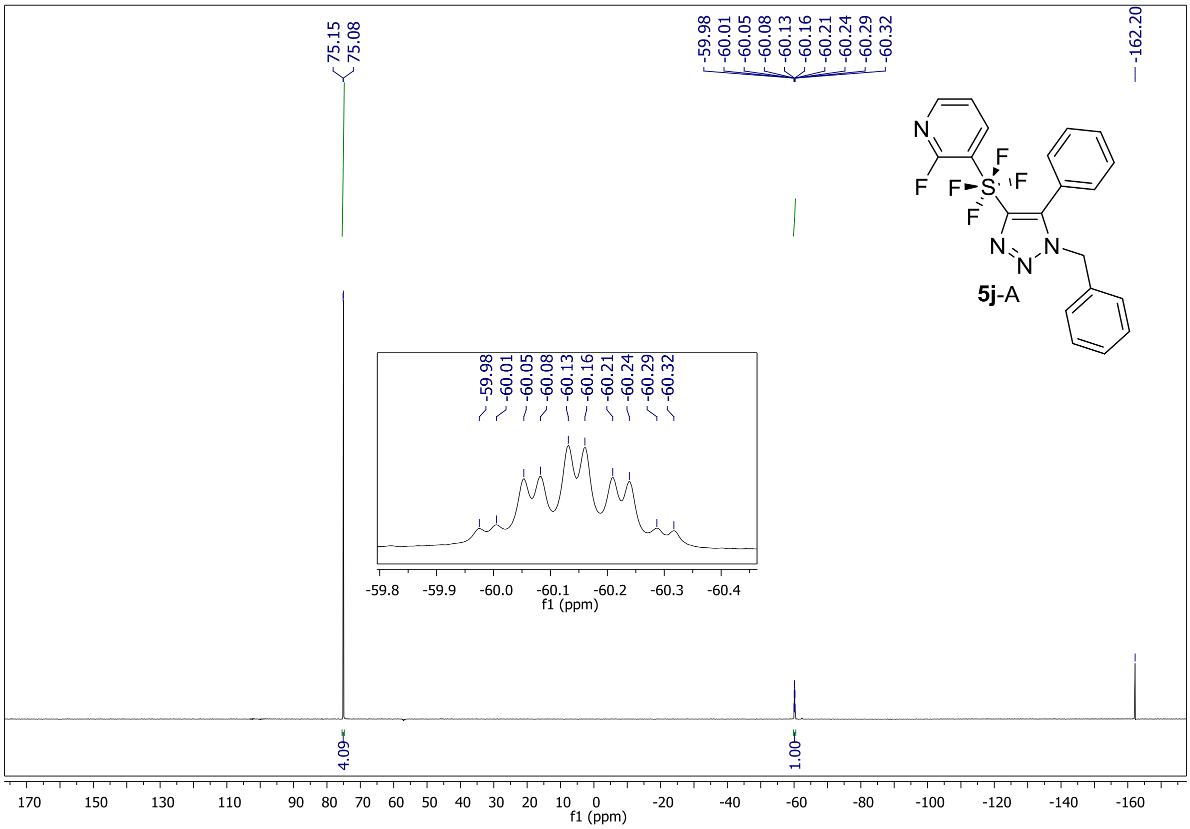
75.15
75.08
-59.98
-60.01
-60.05
-60.08
-60.13
-60.16
-60.21
-60.24
-60.29
-60.32
-162.20
5j-A
-59.8 -59.9 -60.0 -60.1 -60.2 -60.3 -60.4
f1 (ppm)
4.09
1.00
170 150 130 110 90 80 70 60 50 40 30 20 10 0 -20 -40 -60 -80 -100 -120 -140 -160
f1 (ppm)

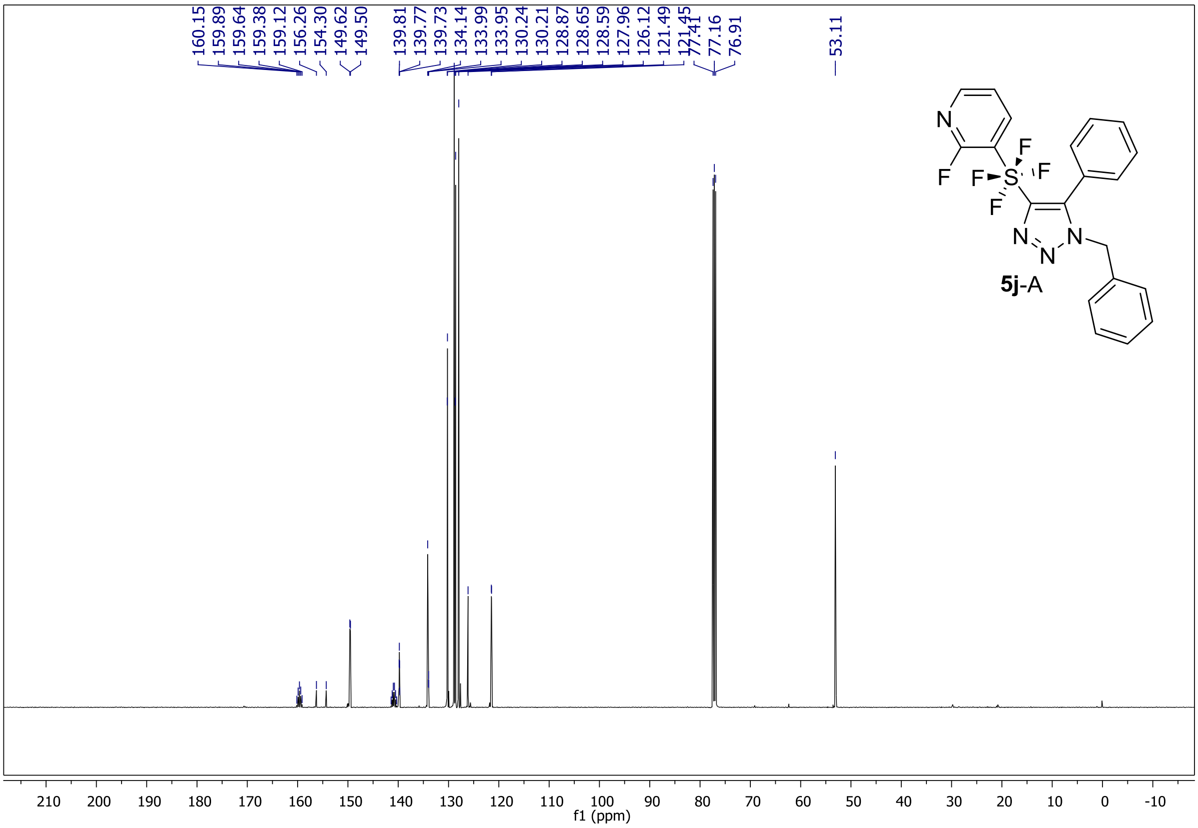
160.15
159.89
159.64
159.38
159.12
156.26
154.30
149.62
149.50
139.81
139.77
139.73
134.14
133.99
133.95
130.24
130.21
128.87
128.65
128.59
127.96
126.12
121.49
121.45
77.41
77.16
76.91
53.11
5j-A
f1 (ppm)

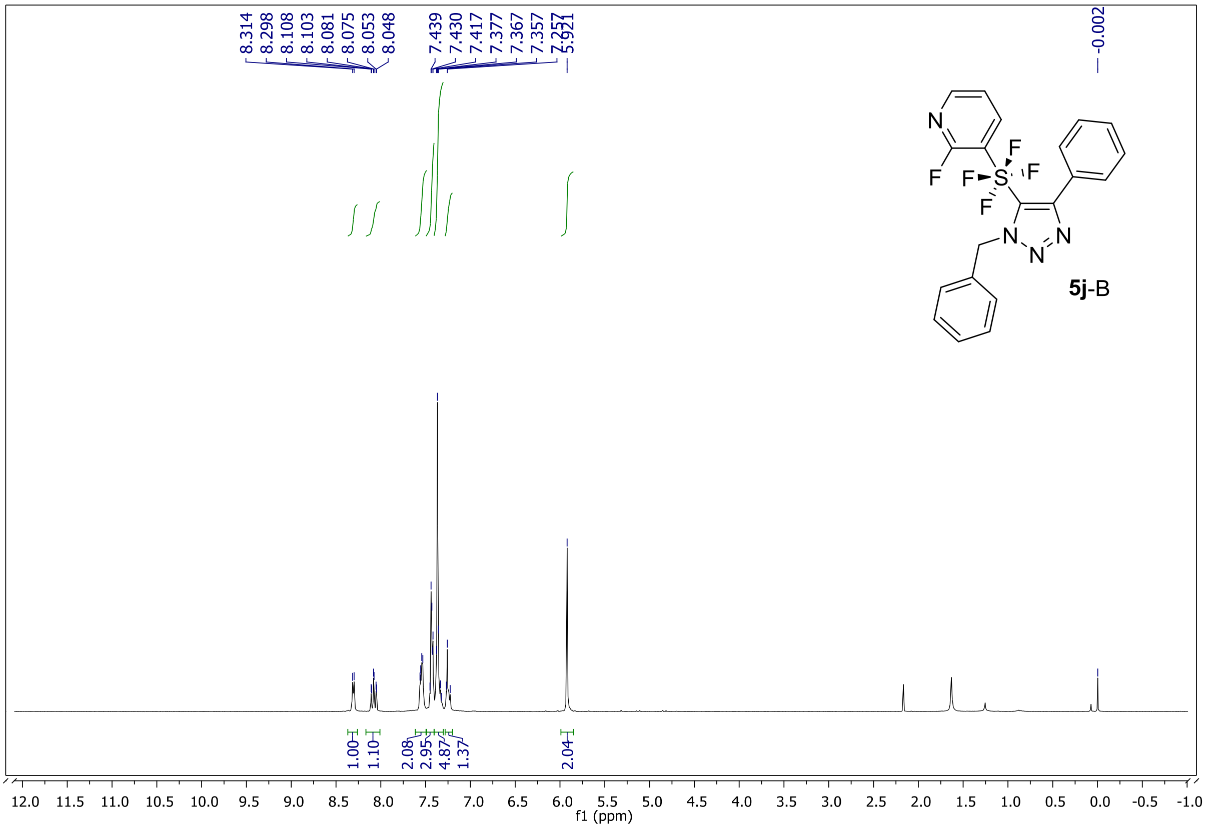
8.314
8.298
8.108
8.103
8.081
8.075
8.053
8.048
7.439
7.430
7.417
7.377
7.367
7.357
7.257
5.921
-0.002
5j-B
1.00
1.10
2.08
2.95
4.87
1.37
2.04
f1 (ppm)

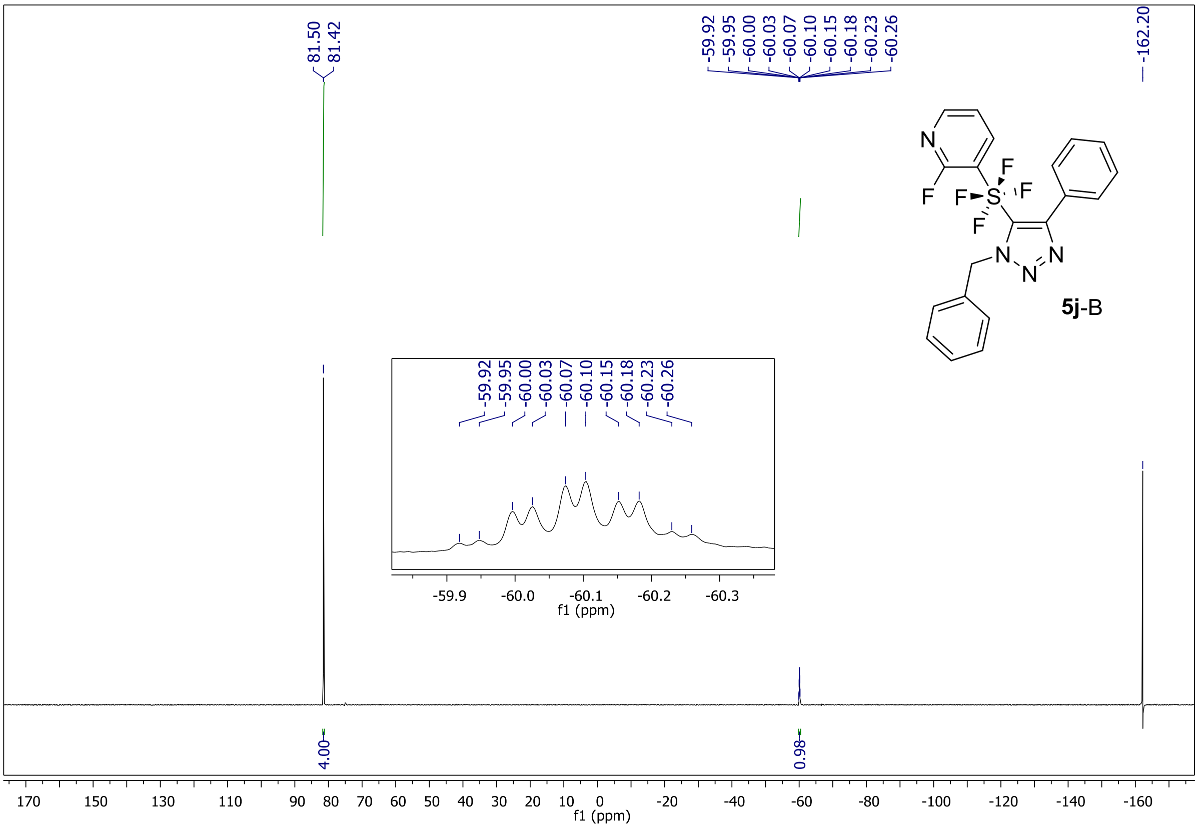
81.50
81.42
-59.92
-59.95
-60.00
-60.03
-60.07
-60.10
-60.15
-60.18
-60.23
-60.26
-162.20
N
F
F
F
F
F
S
N
N
N
5j-B
-59.9
-60.0
-60.1
-60.2
-60.3
f1 (ppm)
4.00
0.98
170 150 130 110 90 80 70 60 50 40 30 20 10 0 -20 -40 -60 -80 -100 -120 -140 -160
f1 (ppm)

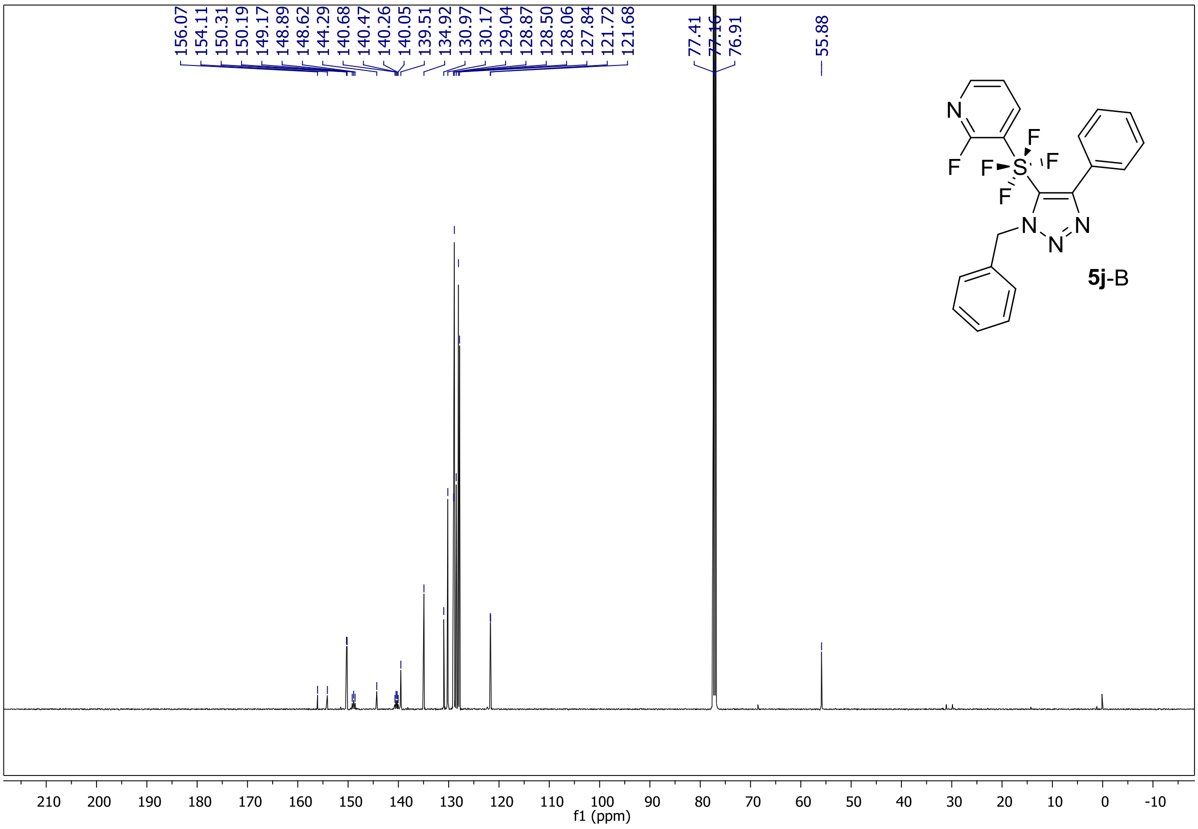
156.07
154.11
150.31
150.19
149.17
148.89
148.62
144.29
140.68
140.47
140.26
140.05
139.51
134.92
130.97
130.17
129.04
128.87
128.50
128.06
127.84
121.72
121.68
77.41
77.16
76.91
55.88
N
F
F
F
F
F
S
N
N
N
5j-B
210 200 190 180 170 160 150 140 130 120 110 100 90 80 70 60 50 40 30 20 10 0 -10
f1 (ppm)

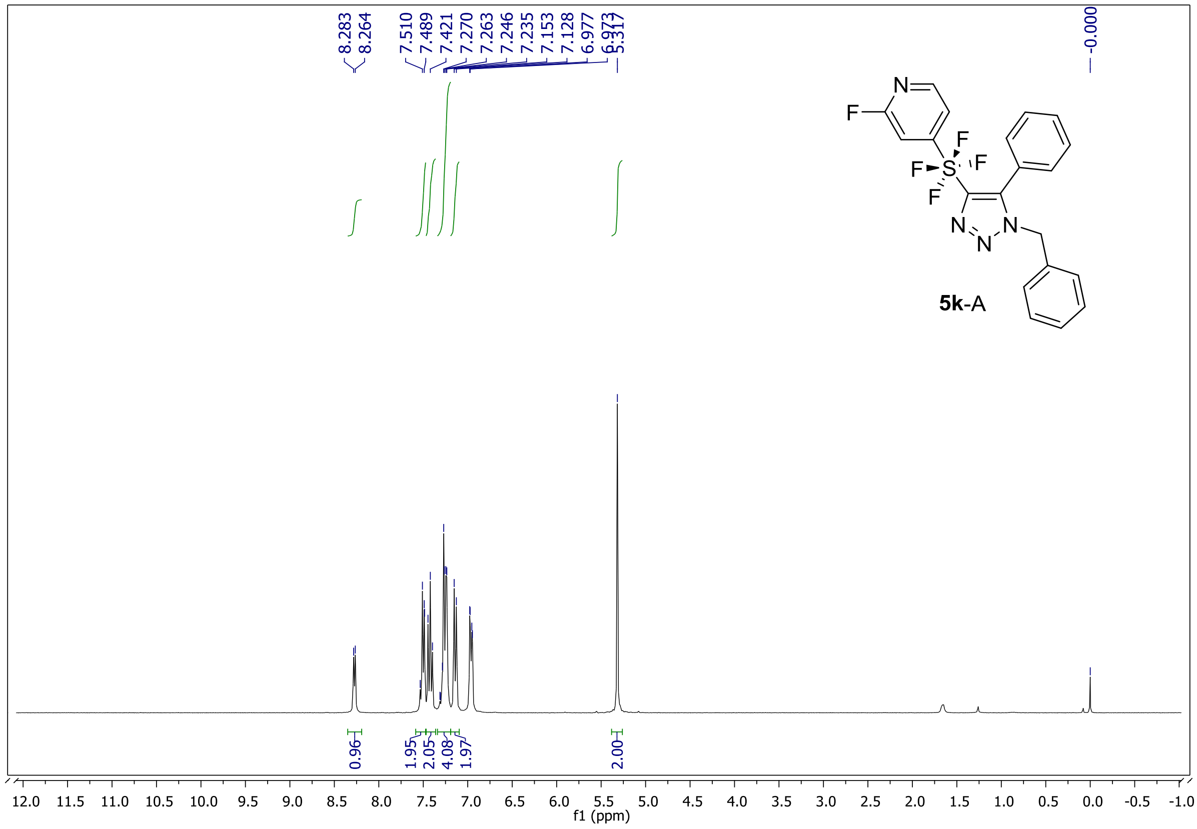
8.283
8.264
7.510
7.489
7.421
7.270
7.263
7.246
7.235
7.153
7.128
6.977
6.973
5.317
-0.000
5k-A
0.96
1.95
2.05
4.08
1.97
2.00
12.0 11.5 11.0 10.5 10.0 9.5 9.0 8.5 8.0 7.5 7.0 6.5 6.0 5.5 5.0 4.5 4.0 3.5 3.0 2.5 2.0 1.5 1.0 0.5 0.0 -0.5 -1.0
f1 (ppm)

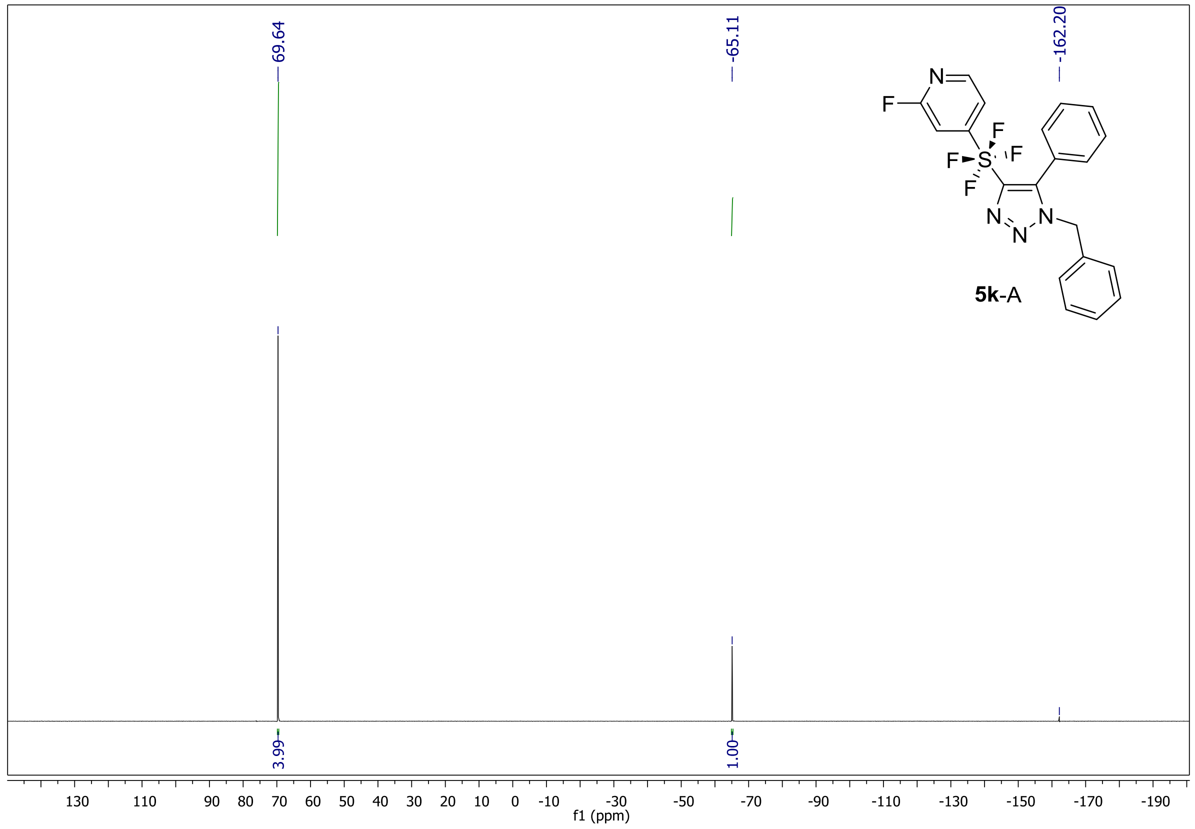
69.64
-65.11
-162.20
5k-A
3.99
1.00
130 110 90 80 70 60 50 40 30 20 10 0 -10 -30 -50 -70 -90 -110 -130 -150 -170 -190
f1 (ppm)

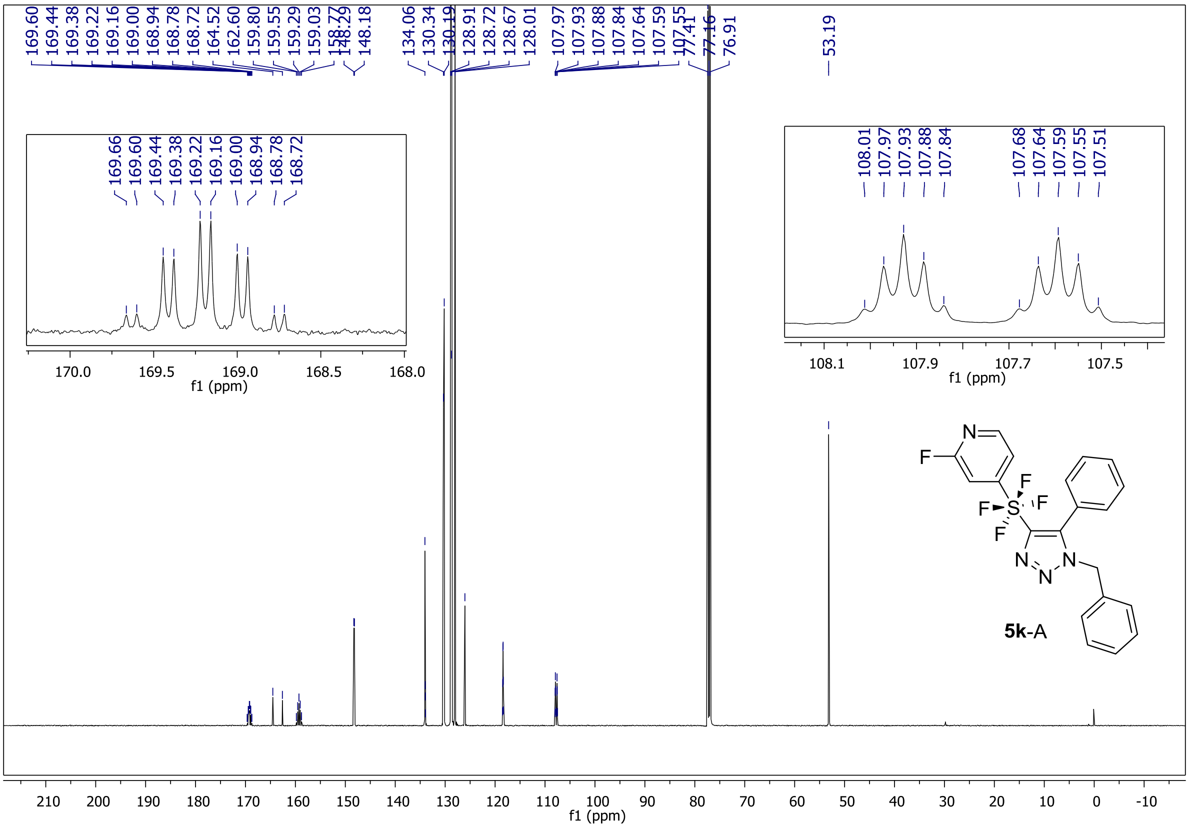

169.60
169.44
169.38
169.22
169.16
169.00
168.94
168.78
168.72
164.52
162.60
159.80
159.55
159.29
159.03
148.18
134.06
130.34
128.91
128.72
128.67
128.01
107.97
107.93
107.88
107.84
107.64
107.59
107.55
76.91
53.19
169.66
169.60
169.44
169.38
169.22
169.16
169.00
168.94
168.78
168.72
f1 (ppm)
108.01
107.97
107.93
107.88
107.84
107.68
107.64
107.59
107.55
107.51
f1 (ppm)
5k-A
f1 (ppm)

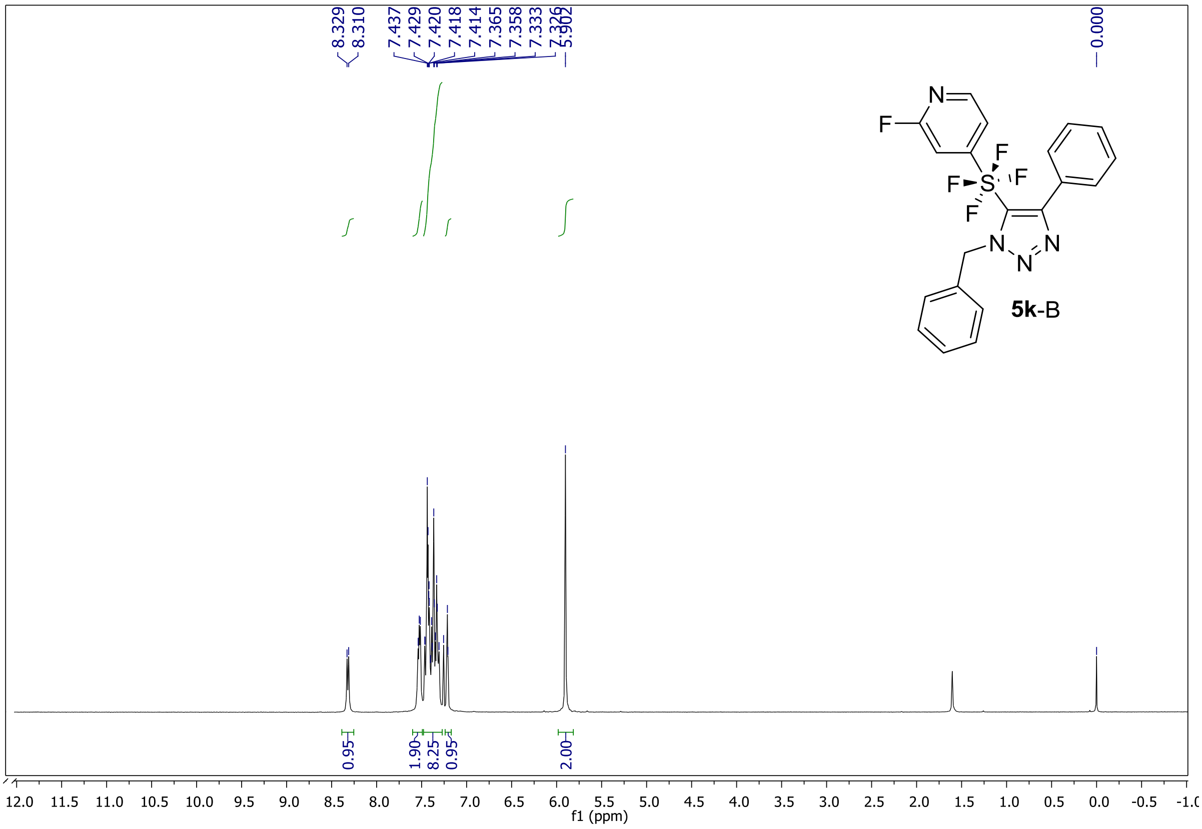

8.329
8.310
7.437
7.429
7.420
7.418
7.414
7.365
7.358
7.333
0.000
5k-B
0.95
1.90
8.25
0.95
2.00
f1 (ppm)

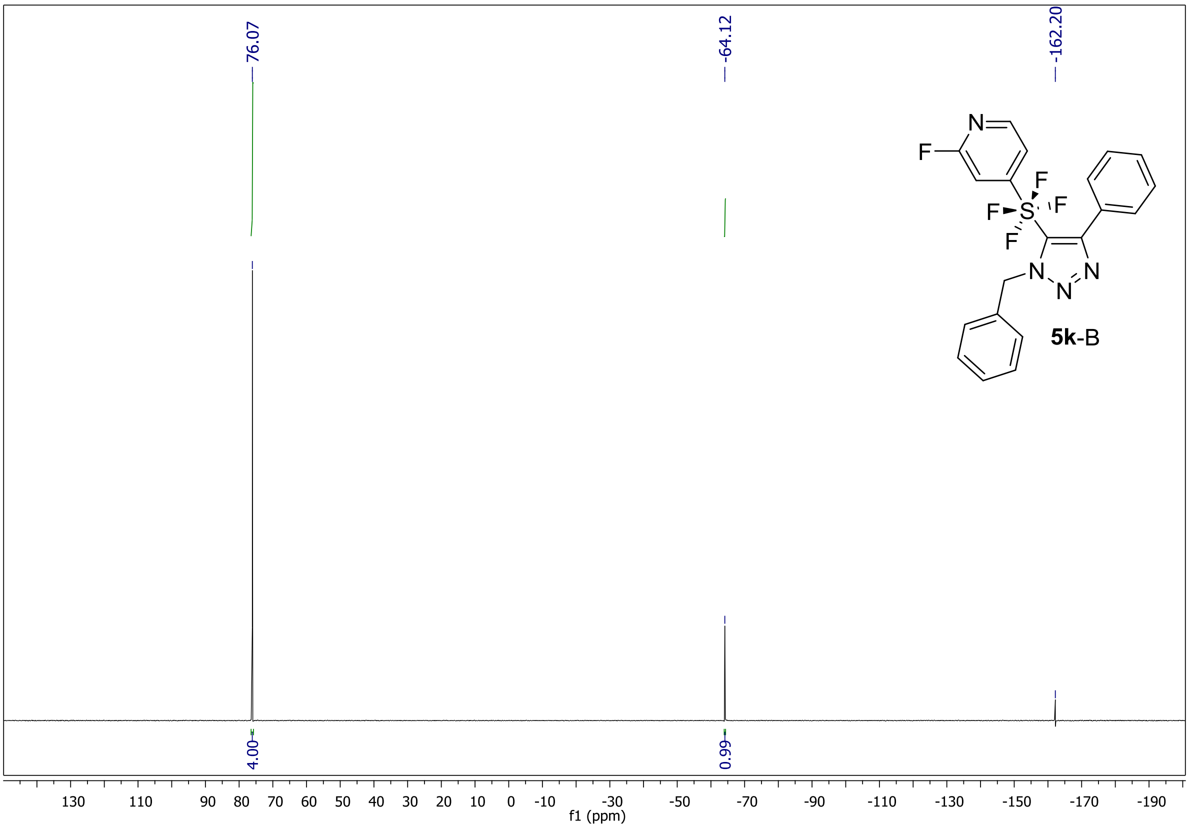

76.07
-64.12
-162.20
4.00
0.99
5k-B
130 110 90 80 70 60 50 40 30 20 10 0 -10 -30 -50 -70 -90 -110 -130 -150 -170 -190
f1 (ppm)

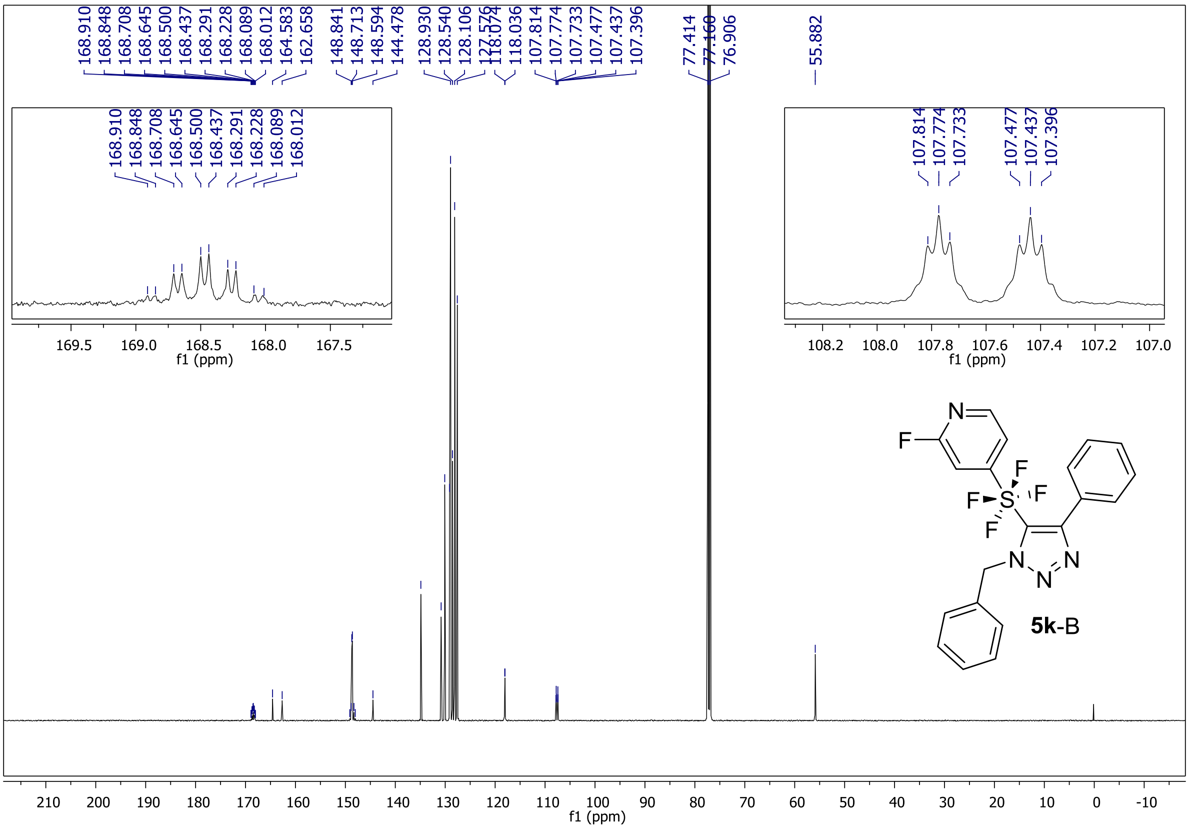

168.910
168.848
168.708
168.645
168.500
168.437
168.291
168.228
168.089
168.012
164.583
162.658
148.841
148.713
148.594
144.478
128.930
128.540
128.106
127.576
118.074
118.036
107.814
107.774
107.733
107.477
107.437
107.396
77.414
77.160
76.906
55.882
169.5 169.0 168.5 168.0 167.5
f1 (ppm)
108.2 108.0 107.8 107.6 107.4 107.2 107.0
f1 (ppm)
5k-B
210 200 190 180 170 160 150 140 130 120 110 100 90 80 70 60 50 40 30 20 10 0 -10
f1 (ppm)

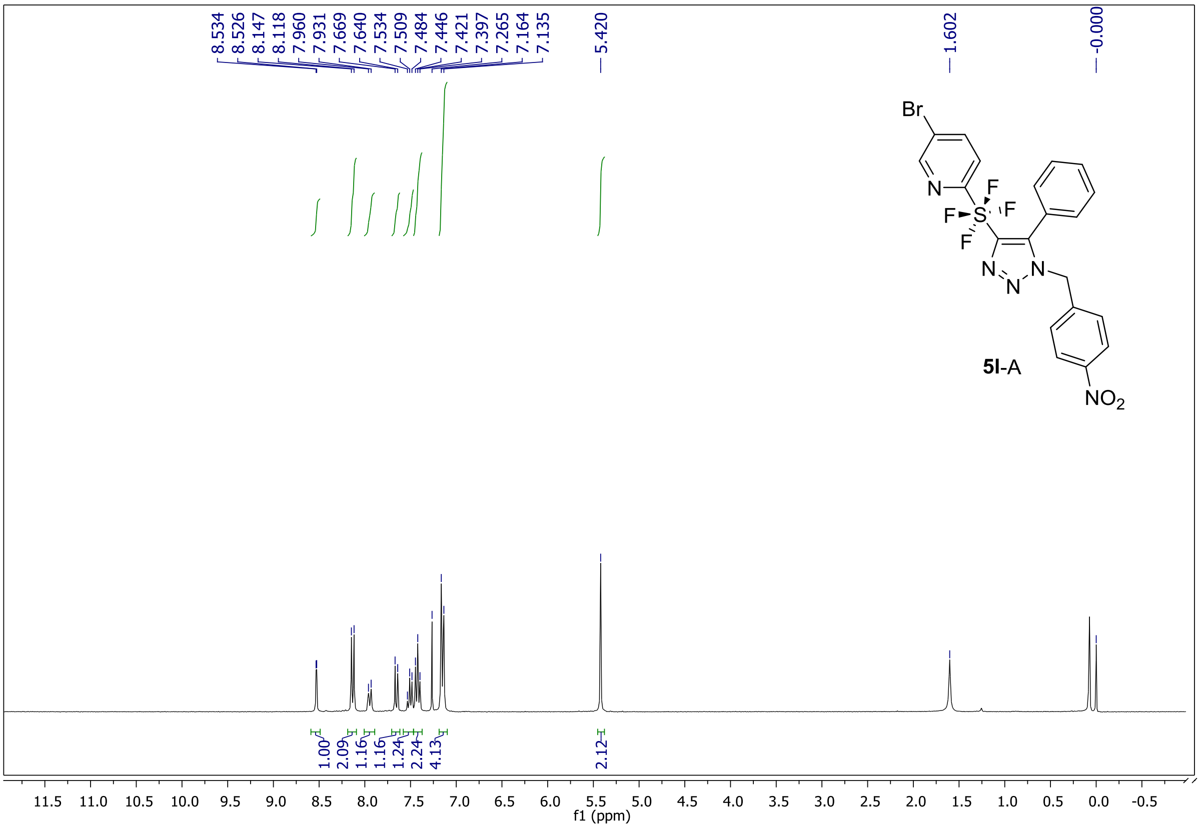
8.534
8.526
8.147
8.118
7.960
7.931
7.669
7.640
7.534
7.509
7.484
7.446
7.421
7.397
7.265
7.164
7.135
5.420
1.602
-0.000
Br
N
F
F
F
S
F
N
N
N
5l-A
NO2
1.00
2.09
1.16
1.16
1.24
2.24
4.13
2.12
f1 (ppm)

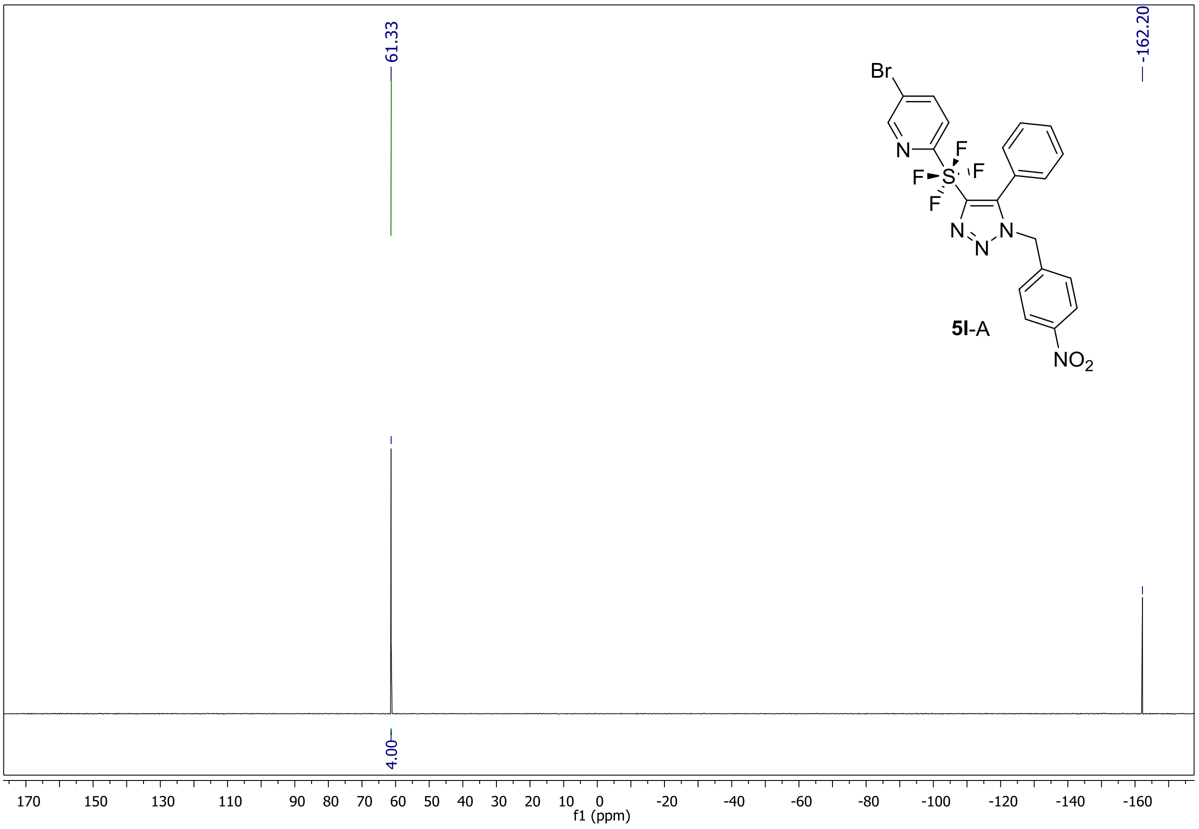
61.33
-162.20
Br
N
F
F
F
S
F
N
N
N
5l-A
NO2
4.00
f1 (ppm)

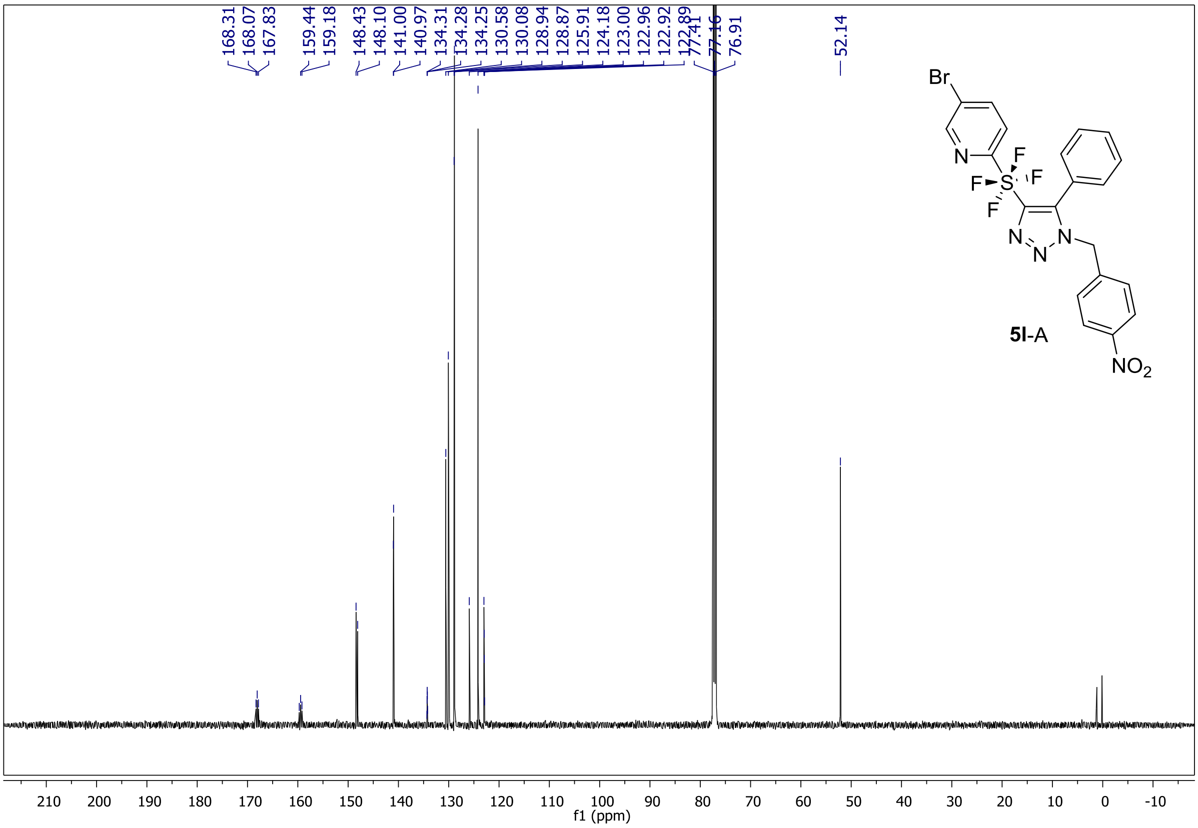
168.31
168.07
167.83
159.44
159.18
148.43
148.10
141.00
140.97
134.31
134.28
134.25
130.58
130.08
128.94
128.87
125.91
124.18
123.00
122.96
122.92
122.89
77.41
77.16
76.91
52.14
Br
N
F
F
F
S
F
N
N
N
NO2
5l-A
210 200 190 180 170 160 150 140 130 120 110 100 90 80 70 60 50 40 30 20 10 0 -10
f1 (ppm)

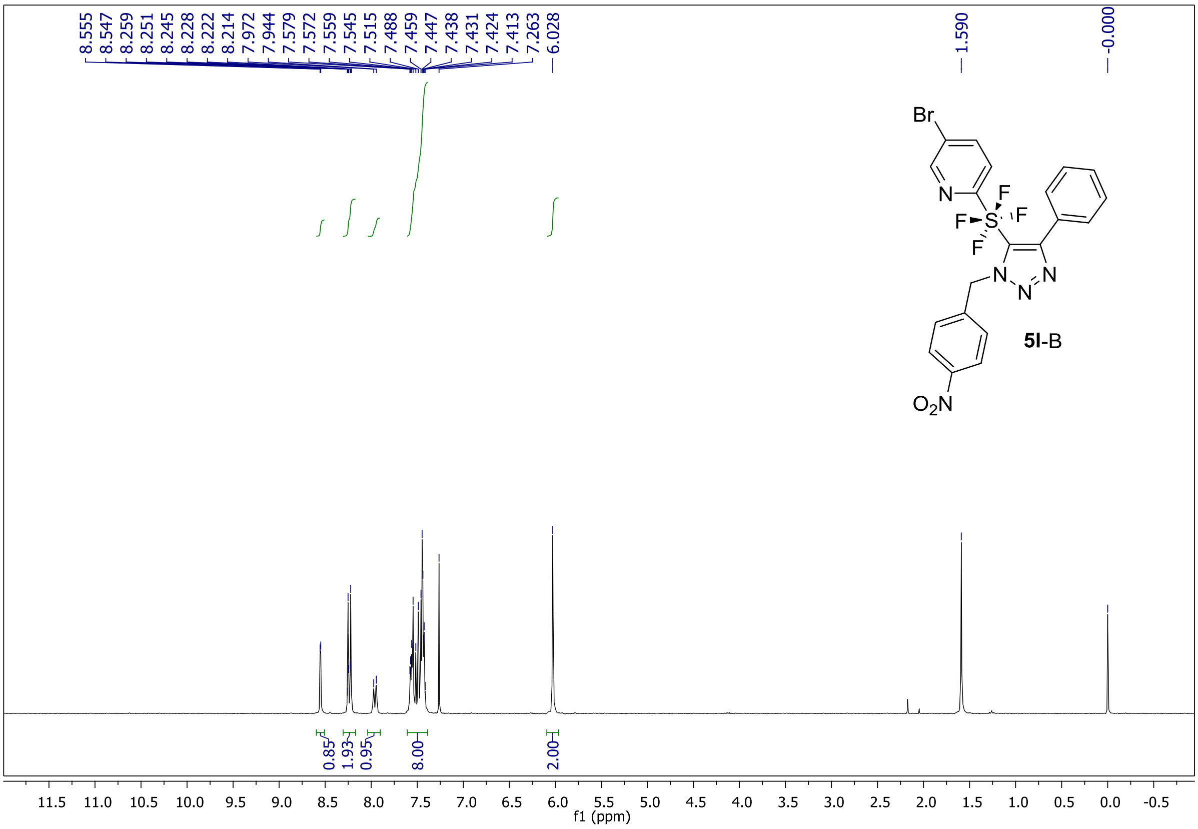
8.555
8.547
8.259
8.251
8.245
8.228
8.222
8.214
7.972
7.944
7.579
7.572
7.559
7.545
7.515
7.488
7.459
7.447
7.438
7.431
7.424
7.413
7.263
6.028
1.590
-0.000
Br
N
F
F
F
S
F
N
N
N
5l-B
O2N
0.85
1.93
0.95
8.00
2.00
11.5 11.0 10.5 10.0 9.5 9.0 8.5 8.0 7.5 7.0 6.5 6.0 5.5 5.0 4.5 4.0 3.5 3.0 2.5 2.0 1.5 1.0 0.5 0.0 -0.5
f1 (ppm)

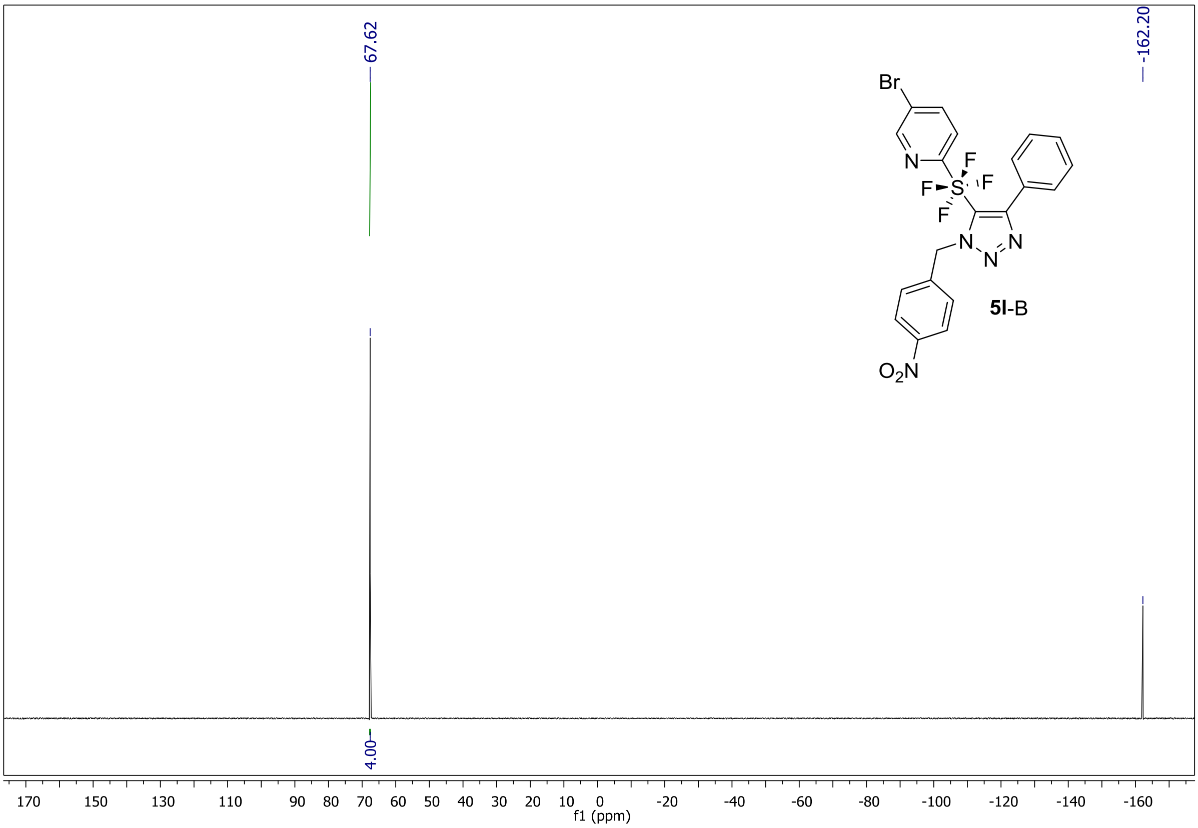
67.62
-162.20
Br
N
F
S
5l-B
O2N
4.00
170 150 130 110 90 80 70 60 50 40 30 20 10 0 -20 -40 -60 -80 -100 -120 -140 -160
f1 (ppm)

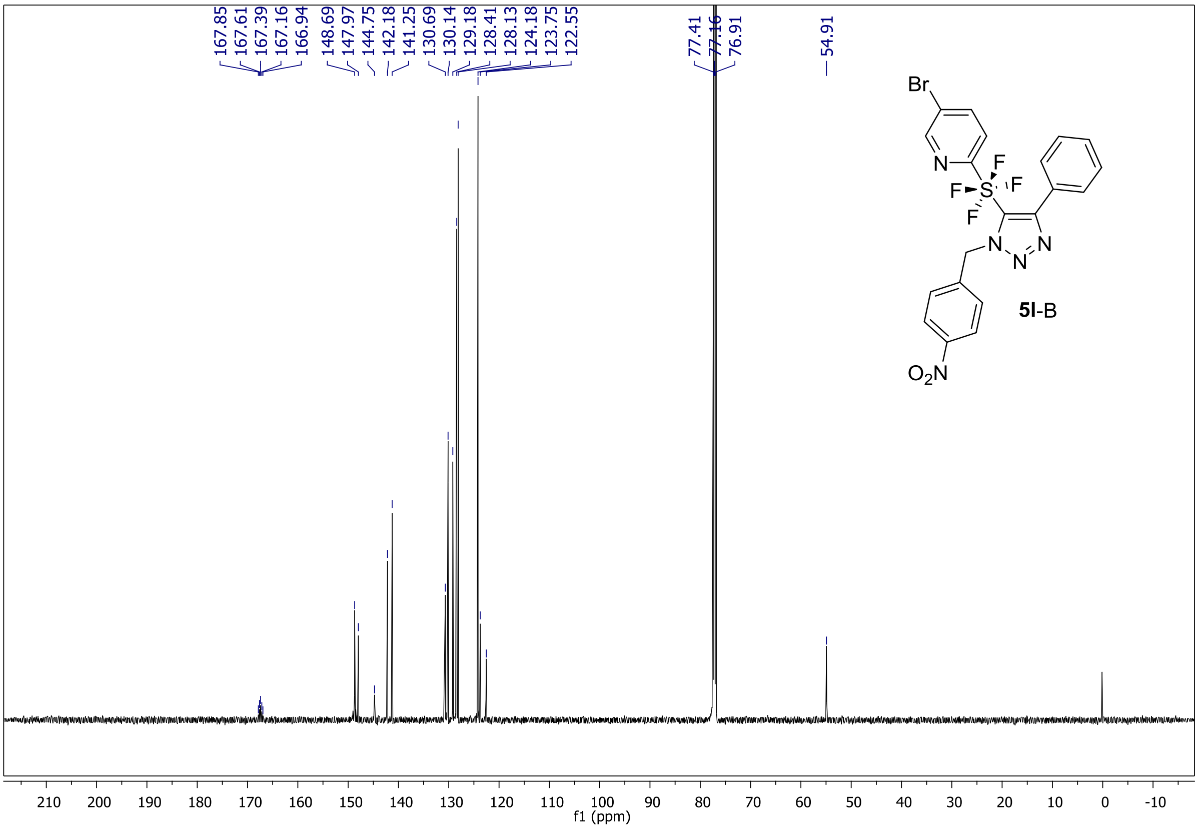
167.85
167.61
167.39
167.16
166.94
148.69
147.97
144.75
142.18
141.25
130.69
130.14
129.18
128.41
128.13
124.18
123.75
122.55
77.41
77.16
76.91
54.91
Br
N
F
S
5l-B
O2N
210 200 190 180 170 160 150 140 130 120 110 100 90 80 70 60 50 40 30 20 10 0 -10
f1 (ppm)

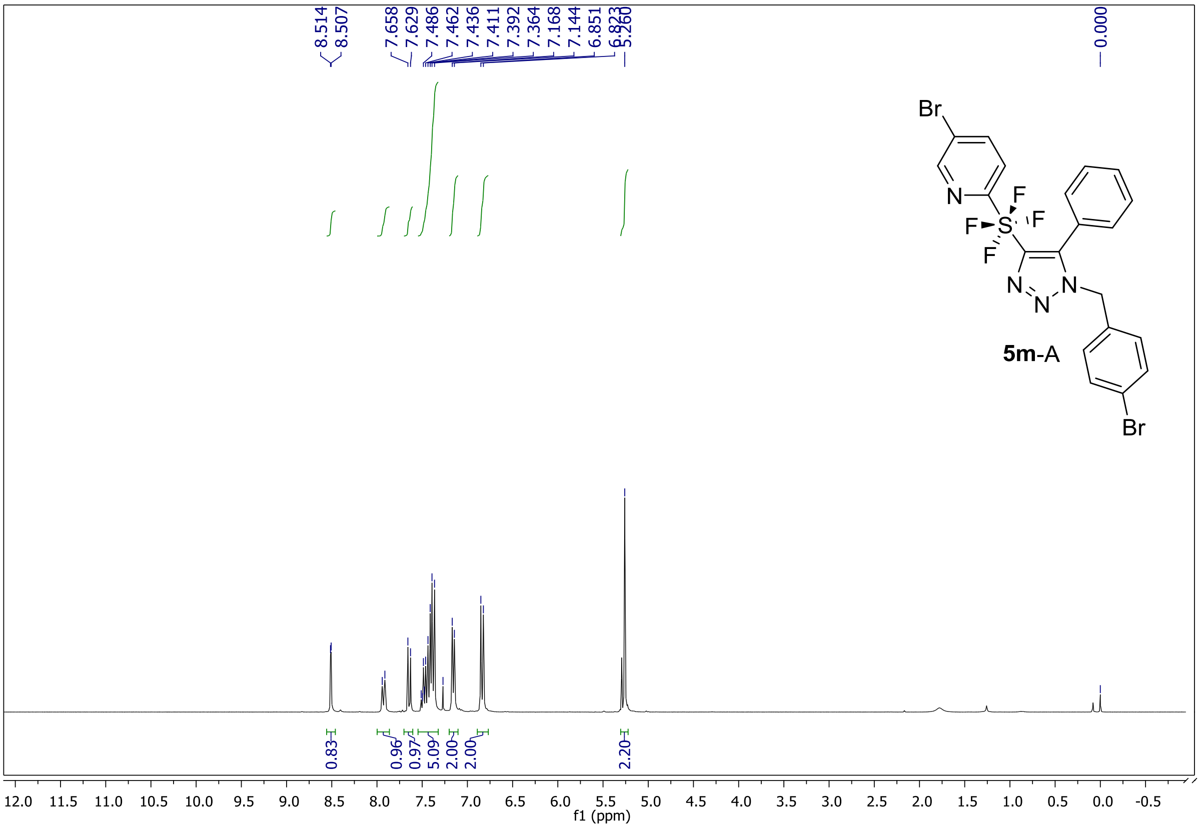
8.514
8.507
7.658
7.629
7.486
7.462
7.436
7.411
7.392
7.364
7.168
7.144
6.851
6.823
5.260
0.000
Br
N
F
F
F
S
F
N
N
N
5m-A
Br
0.83
0.96
0.97
5.09
2.00
2.00
2.20
12.0 11.5 11.0 10.5 10.0 9.5 9.0 8.5 8.0 7.5 7.0 6.5 6.0 5.5 5.0 4.5 4.0 3.5 3.0 2.5 2.0 1.5 1.0 0.5 0.0 -0.5
f1 (ppm)

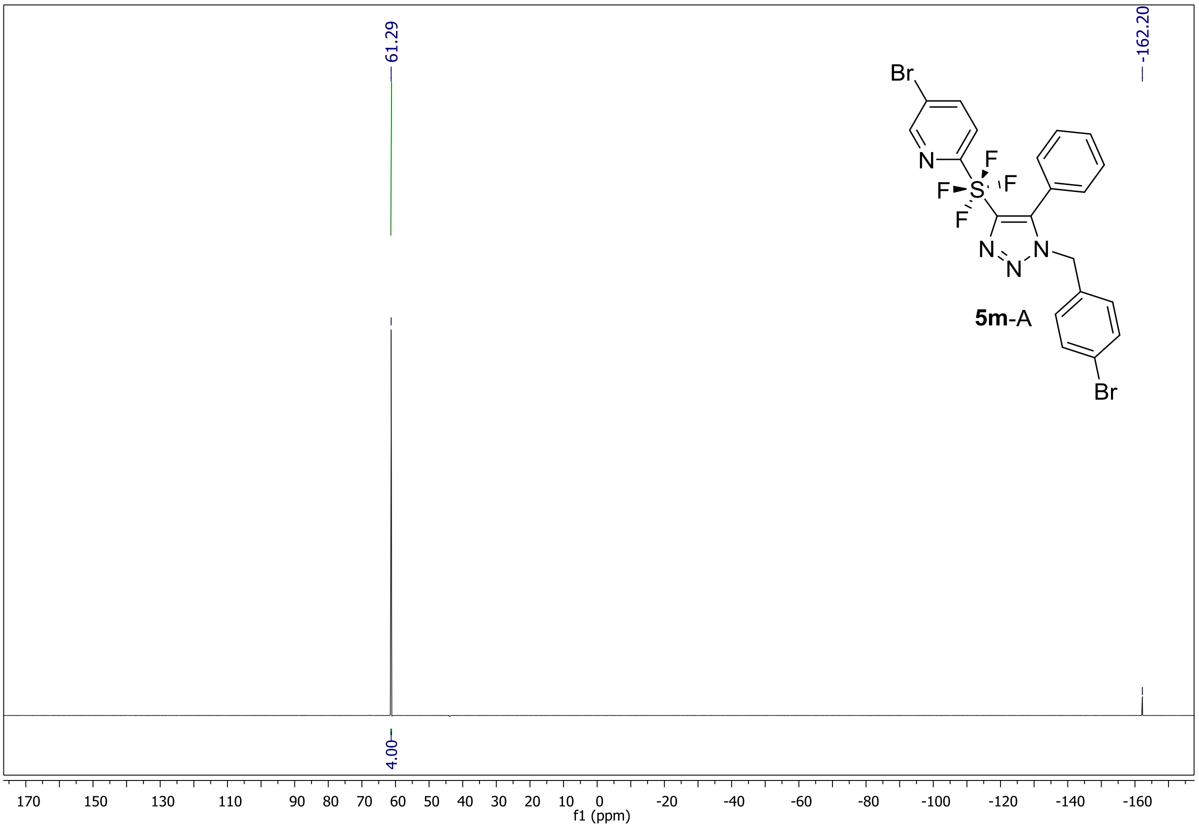
61.29
-162.20
Br
N
F
F
F
S
F
N
N
N
5m-A
Br
4.00
170 150 130 110 90 80 70 60 50 40 30 20 10 0 -20 -40 -60 -80 -100 -120 -140 -160
f1 (ppm)

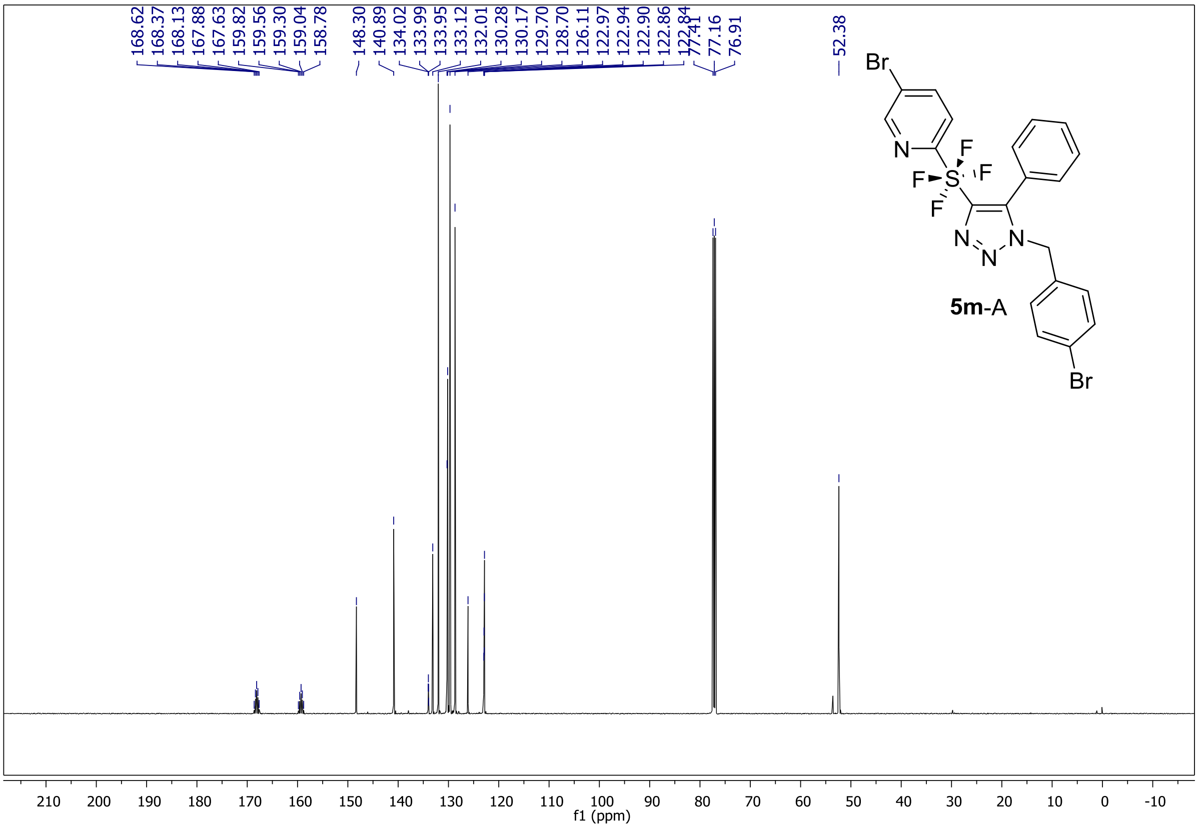
168.62
168.37
168.13
167.88
167.63
159.82
159.56
159.30
159.04
158.78
148.30
140.89
134.02
133.99
133.95
133.12
132.01
130.28
130.17
129.70
128.70
126.11
122.97
122.94
122.90
122.86
122.84
77.41
77.16
76.91
52.38
Br
N
F
F
S
F
F
N
N
N
Br
5m-A
210 200 190 180 170 160 150 140 130 120 110 100 90 80 70 60 50 40 30 20 10 0 -10
f1 (ppm)

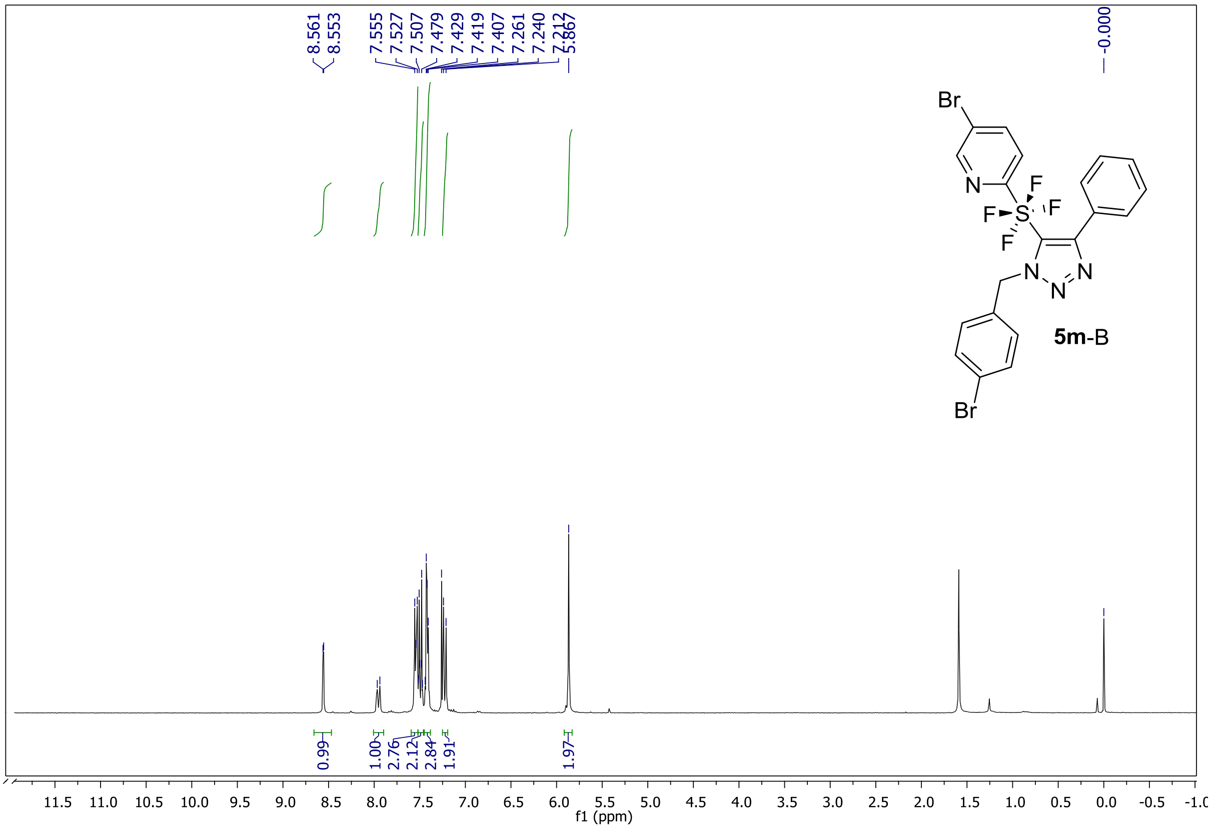
8.561
8.553
7.555
7.527
7.507
7.479
7.429
7.419
7.407
7.261
7.240
7.212
5.867
-0.000
Br
N
F
F
S
F
F
N
N
N
Br
5m-B
0.99
1.00
2.76
2.12
2.84
1.91
1.97
11.5 11.0 10.5 10.0 9.5 9.0 8.5 8.0 7.5 7.0 6.5 6.0 5.5 5.0 4.5 4.0 3.5 3.0 2.5 2.0 1.5 1.0 0.5 0.0 -0.5 -1.0
f1 (ppm)

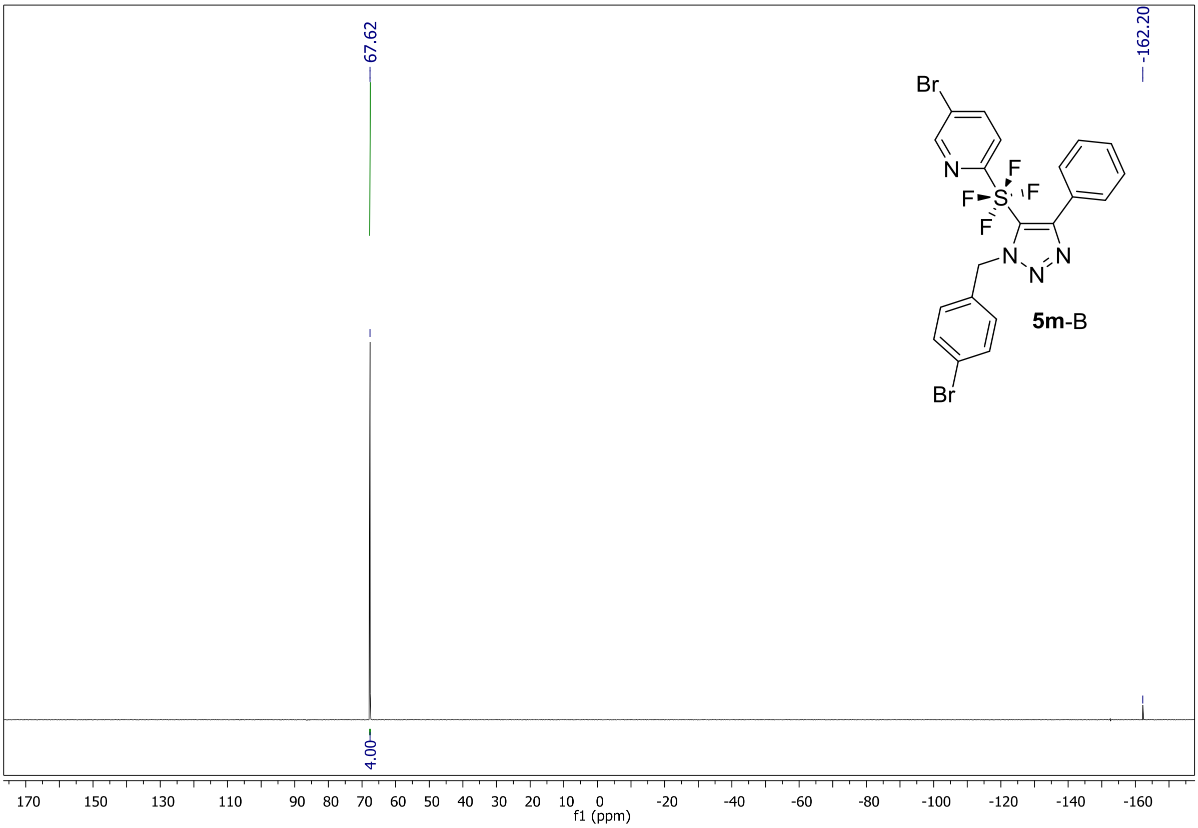
67.62
-162.20
Br
N
F
F
F
F
S
N
N
N
Br
5m-B
4.00
170
150
130
110
90
80
70
60
50
40
30
20
10
0
-20
-40
-60
-80
-100
-120
-140
-160
f1 (ppm)

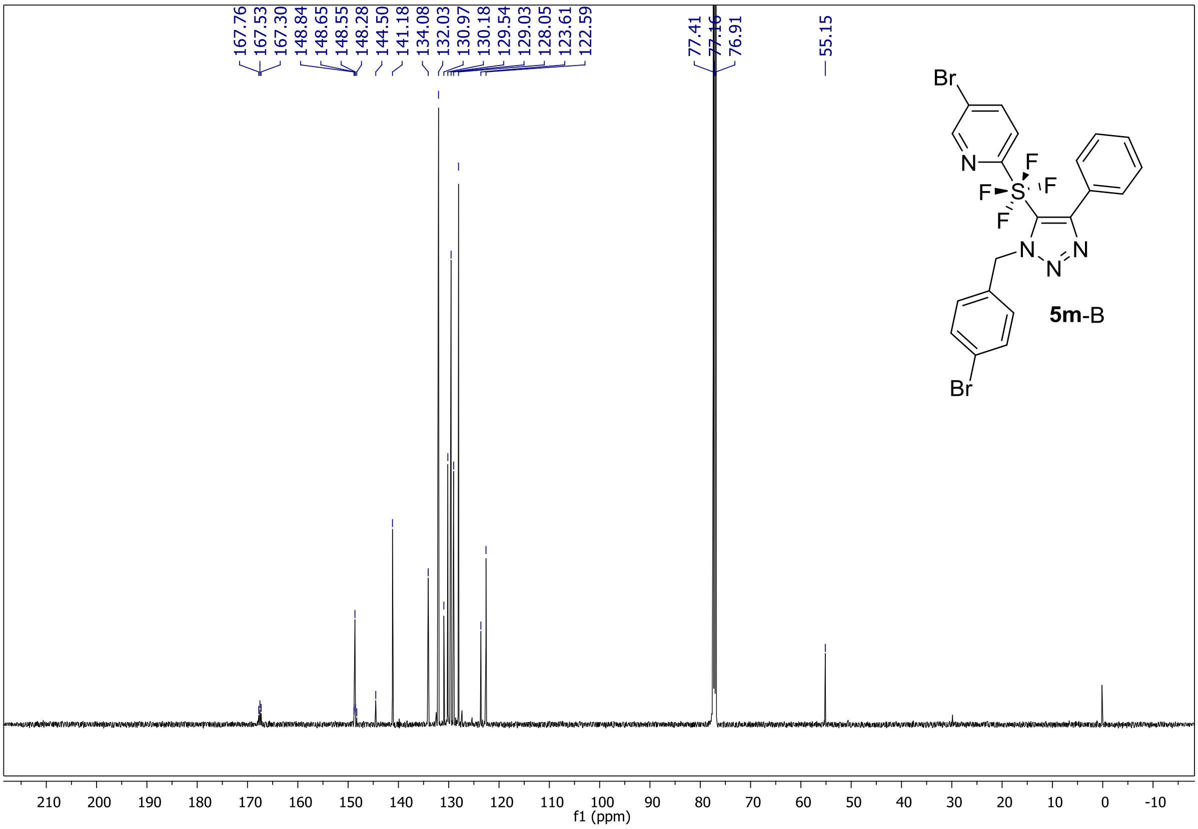
167.76
167.53
167.30
148.84
148.65
148.55
148.28
144.50
141.18
134.08
132.03
130.97
130.18
129.54
129.03
128.05
123.61
122.59
77.41
77.16
76.91
55.15
Br
N
F
F
F
F
S
N
N
N
Br
5m-B
210
200
190
180
170
160
150
140
130
120
110
100
90
80
70
60
50
40
30
20
10
0
-10
f1 (ppm)

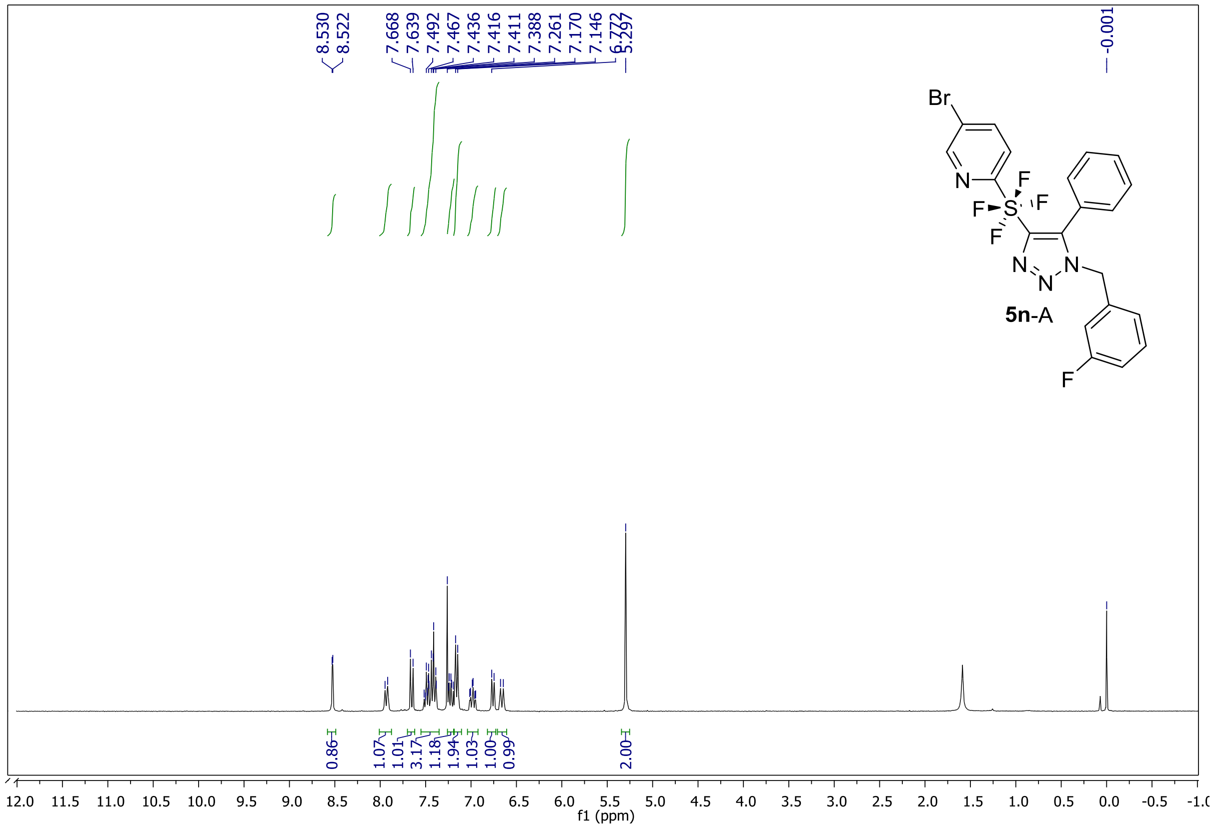
8.530
8.522
7.668
7.639
7.492
7.467
7.436
7.416
7.411
7.388
7.261
7.170
7.146
6.772
5.297
-0.001
Br
N
F
F
F
F
S
N
N
N
5n-A
F
0.86
1.07
1.01
3.17
1.18
1.94
1.03
1.00
0.99
2.00
f1 (ppm)

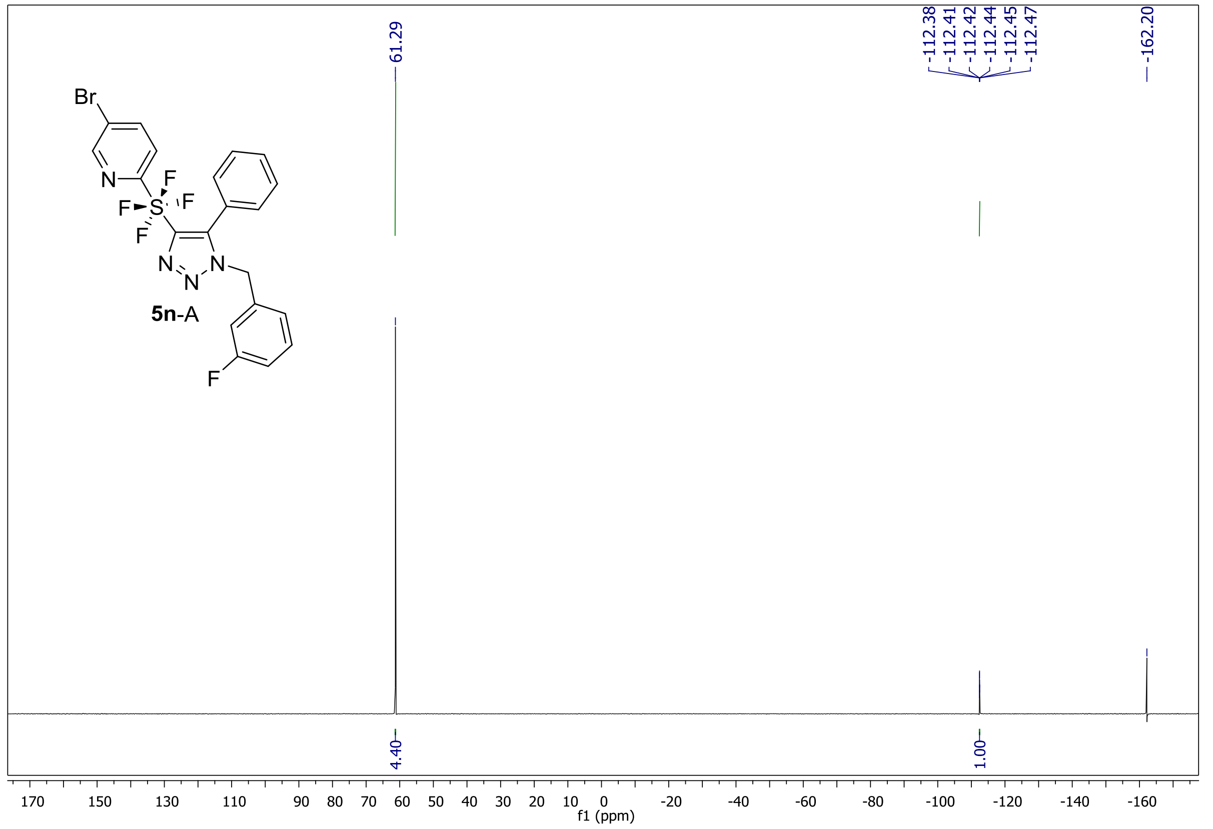
61.29
-112.38
-112.41
-112.42
-112.44
-112.45
-112.47
-162.20
Br
N
F
F
F
F
S
N
N
N
5n-A
F
4.40
1.00
f1 (ppm)

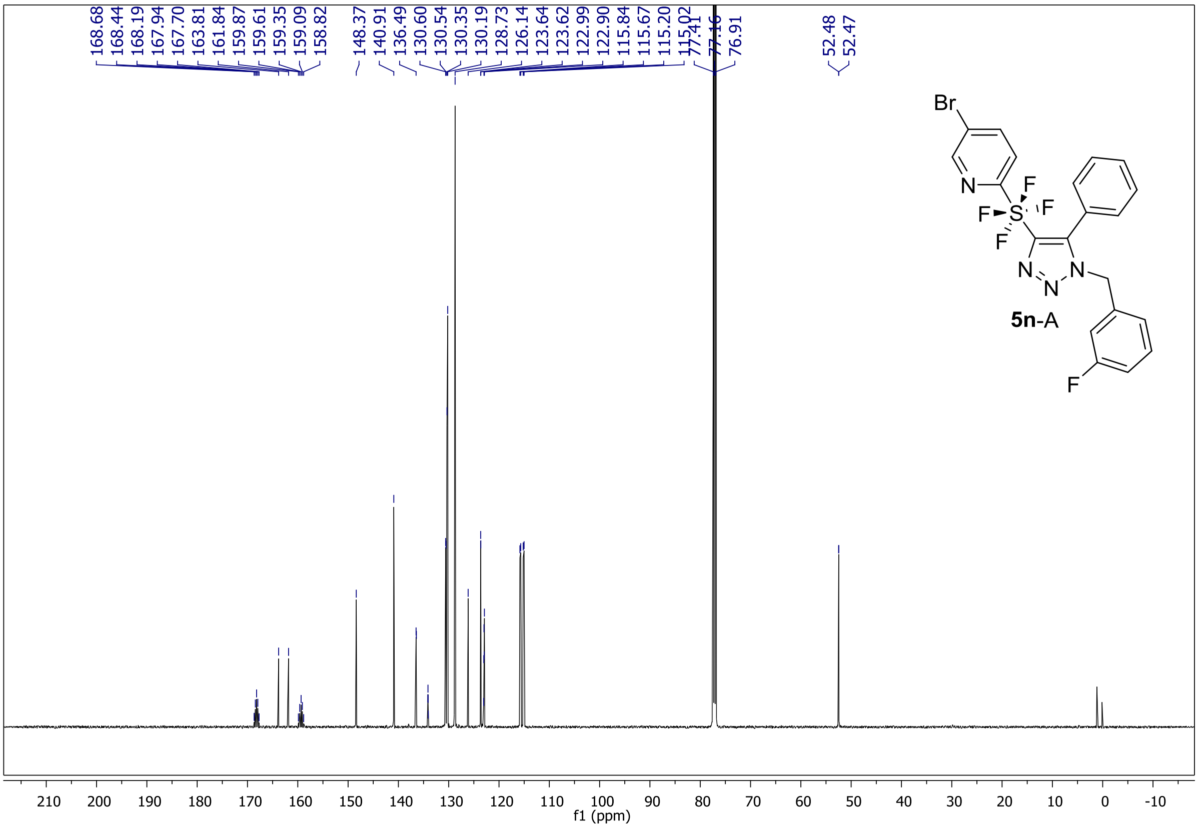
168.68
168.44
168.19
167.94
167.70
163.81
161.84
159.87
159.61
159.35
159.09
158.82
148.37
140.91
136.49
130.60
130.54
130.35
130.19
128.73
126.14
123.64
123.62
122.99
122.90
115.84
115.67
115.20
115.02
77.41
77.16
76.91
52.48
52.47
Br
N
F
F
F
F
N
N
N
5n-A
F
210 200 190 180 170 160 150 140 130 120 110 100 90 80 70 60 50 40 30 20 10 0 -10
f1 (ppm)

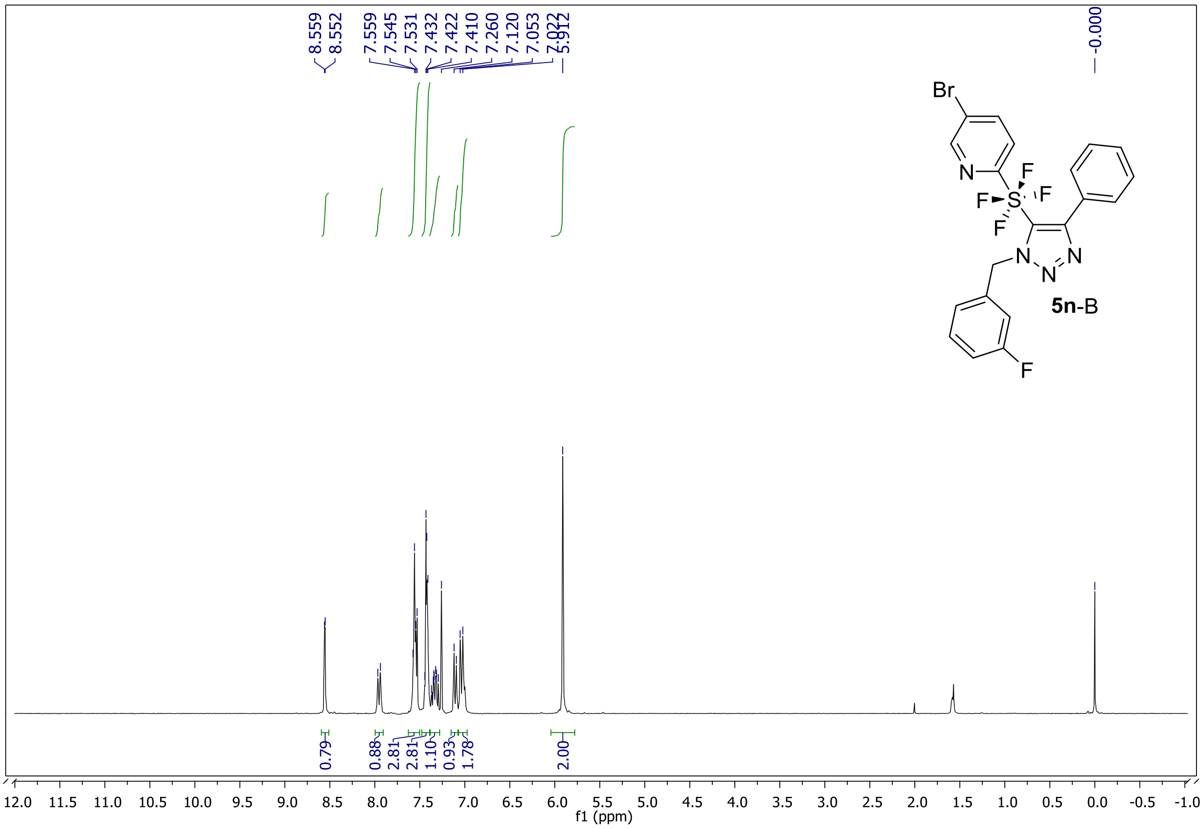
8.559
8.552
7.559
7.545
7.531
7.432
7.422
7.410
7.260
7.120
7.053
7.022
5.912
-0.000
Br
N
F
F
F
F
N
N
N
5n-B
F
0.79
0.88
2.81
2.81
1.10
0.93
1.78
2.00
12.0 11.5 11.0 10.5 10.0 9.5 9.0 8.5 8.0 7.5 7.0 6.5 6.0 5.5 5.0 4.5 4.0 3.5 3.0 2.5 2.0 1.5 1.0 0.5 0.0 -0.5 -1.0
f1 (ppm)

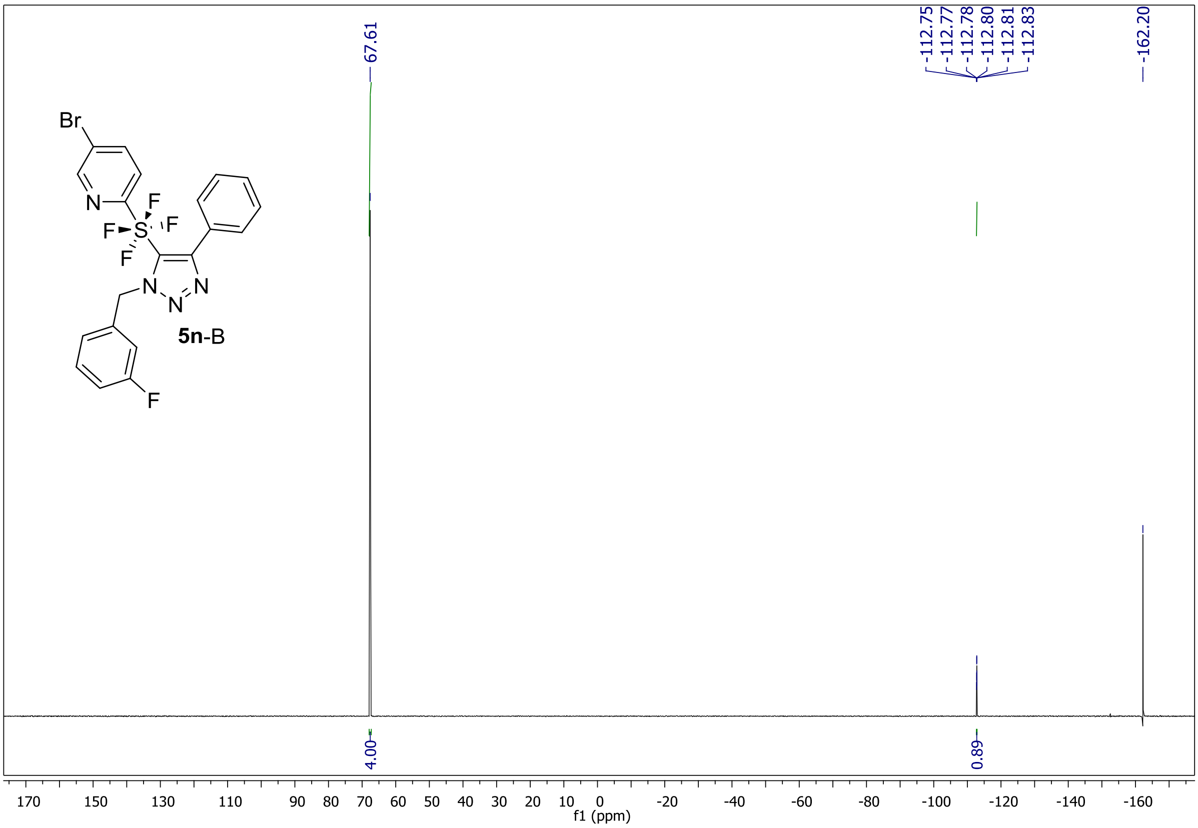

67.61
-112.75
-112.77
-112.78
-112.80
-112.81
-112.83
-162.20
Br
N
F
F
S
F
F
N
N
N
5n-B
F
4.00
0.89
170 150 130 110 90 80 70 60 50 40 30 20 10 0 -20 -40 -60 -80 -100 -120 -140 -160
f1 (ppm)

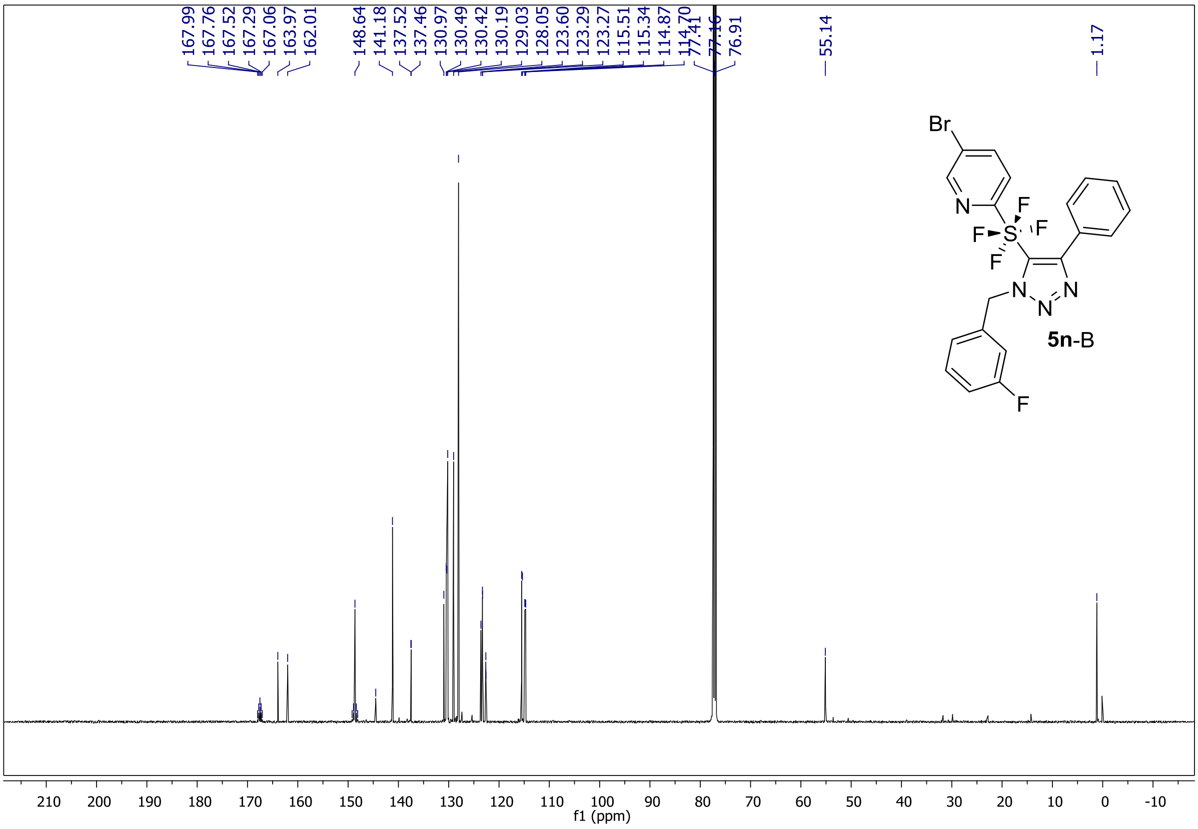

167.99
167.76
167.52
167.29
167.06
163.97
162.01
148.64
141.18
137.52
137.46
130.97
130.49
130.42
130.19
129.03
128.05
123.60
123.29
123.27
115.51
115.34
114.87
114.70
77.41
77.16
76.91
55.14
1.17
Br
N
F
F
S
F
F
N
N
N
5n-B
F
210 200 190 180 170 160 150 140 130 120 110 100 90 80 70 60 50 40 30 20 10 0 -10
f1 (ppm)

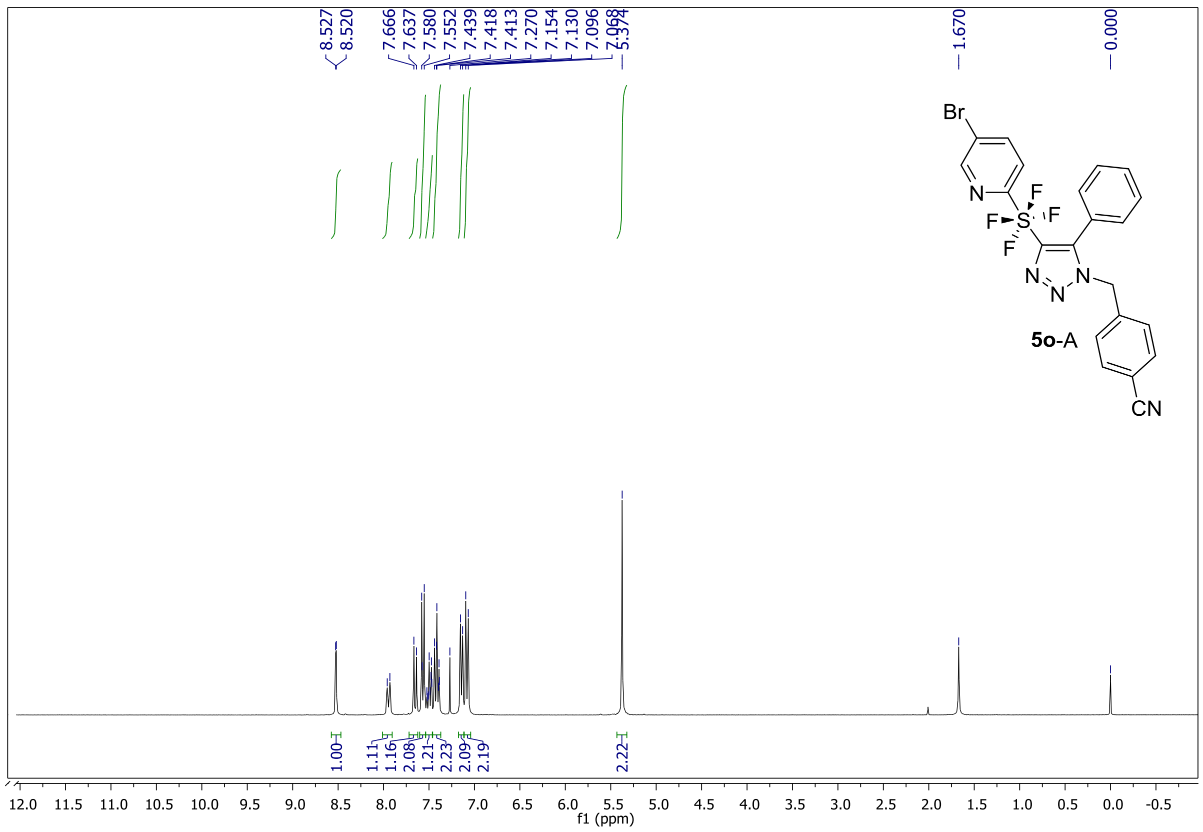
8.527
8.520
7.666
7.637
7.580
7.552
7.439
7.418
7.413
7.270
7.154
7.130
7.096
7.068
5.374
1.670
0.000
Br
N
F
S
N
N
N
5o-A
CN
1.00
1.11
1.16
2.08
1.21
2.23
2.09
2.19
2.22
12.0 11.5 11.0 10.5 10.0 9.5 9.0 8.5 8.0 7.5 7.0 6.5 6.0 5.5 5.0 4.5 4.0 3.5 3.0 2.5 2.0 1.5 1.0 0.5 0.0 -0.5
f1 (ppm)

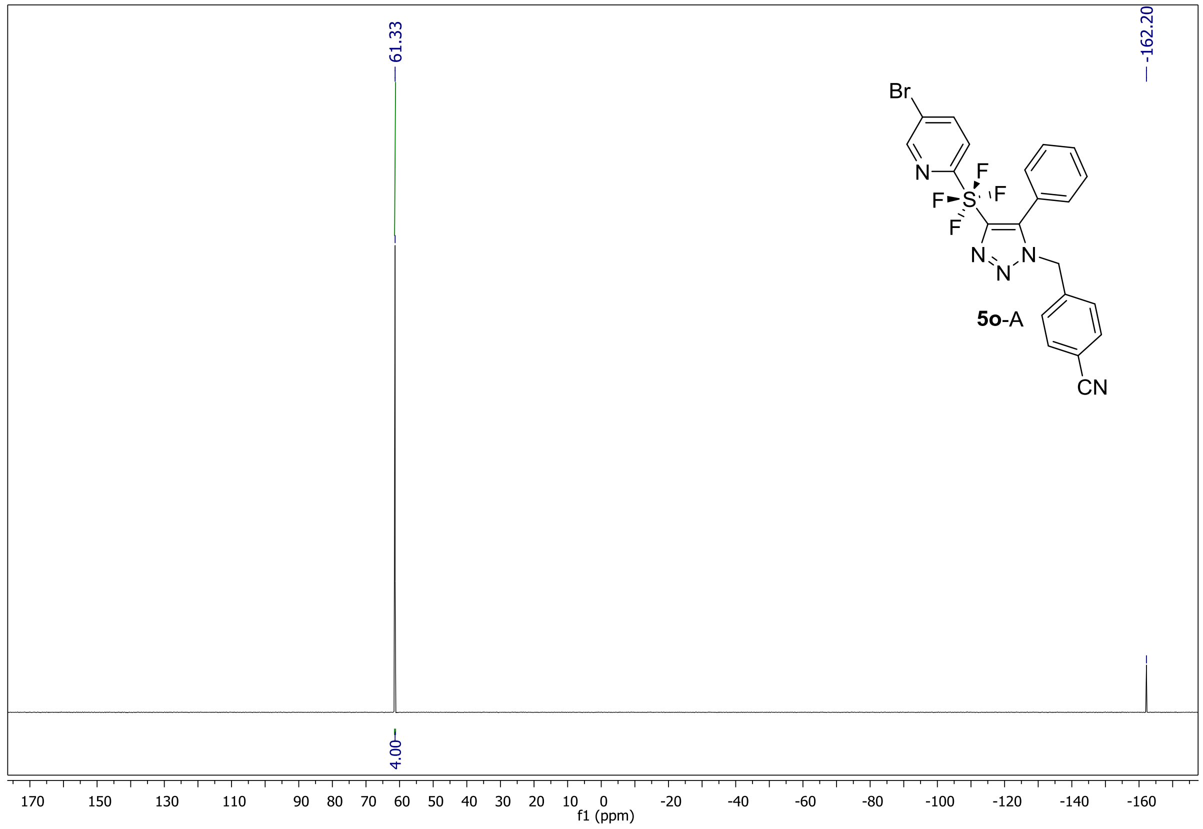
61.33
-162.20
Br
N
F
S
N
N
N
5o-A
CN
4.00
170 150 130 110 90 80 70 60 50 40 30 20 10 0 -20 -40 -60 -80 -100 -120 -140 -160
f1 (ppm)

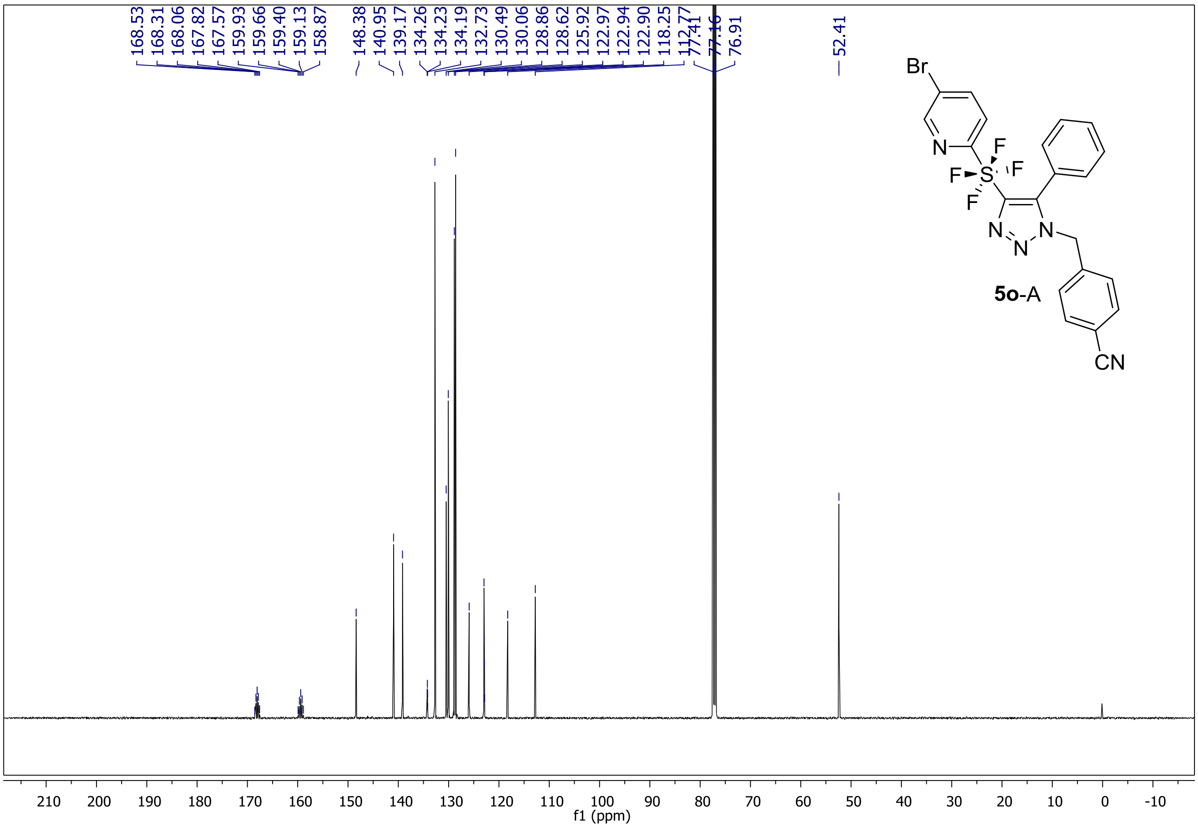
168.53
168.31
168.06
167.82
167.57
159.93
159.66
159.40
159.13
158.87
148.38
140.95
139.17
134.26
134.23
134.19
132.73
130.49
130.06
128.86
128.62
125.92
122.97
122.94
122.90
118.25
112.77
77.41
77.16
76.91
52.41
Br
N
F
F
F
F
S
N
N
N
5o-A
CN
210 200 190 180 170 160 150 140 130 120 110 100 90 80 70 60 50 40 30 20 10 0 -10
f1 (ppm)

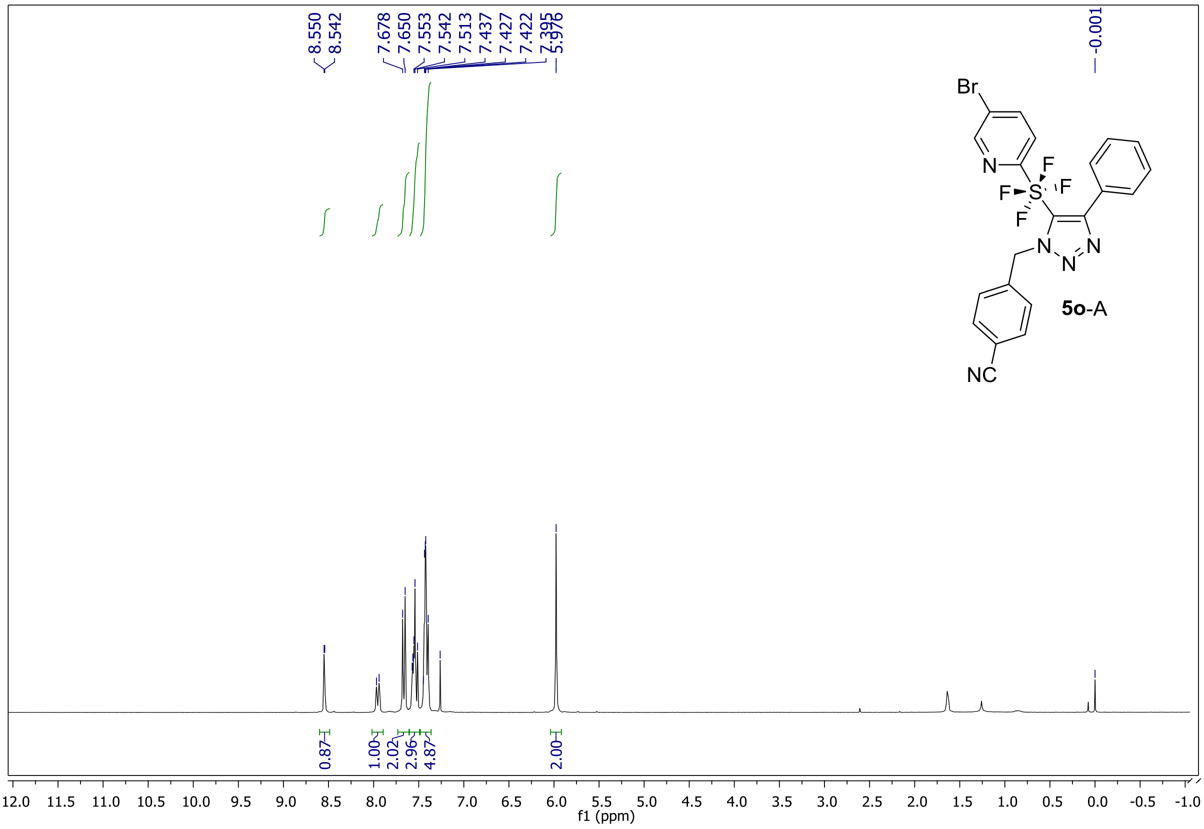
8.550
8.542
7.678
7.650
7.553
7.542
7.513
7.437
7.427
7.422
7.395
5.976
-0.001
Br
N
F
F
F
F
S
N
N
N
5o-A
NC
0.87
1.00
2.02
2.96
4.87
2.00
12.0 11.5 11.0 10.5 10.0 9.5 9.0 8.5 8.0 7.5 7.0 6.5 6.0 5.5 5.0 4.5 4.0 3.5 3.0 2.5 2.0 1.5 1.0 0.5 0.0 -0.5 -1.0
f1 (ppm)

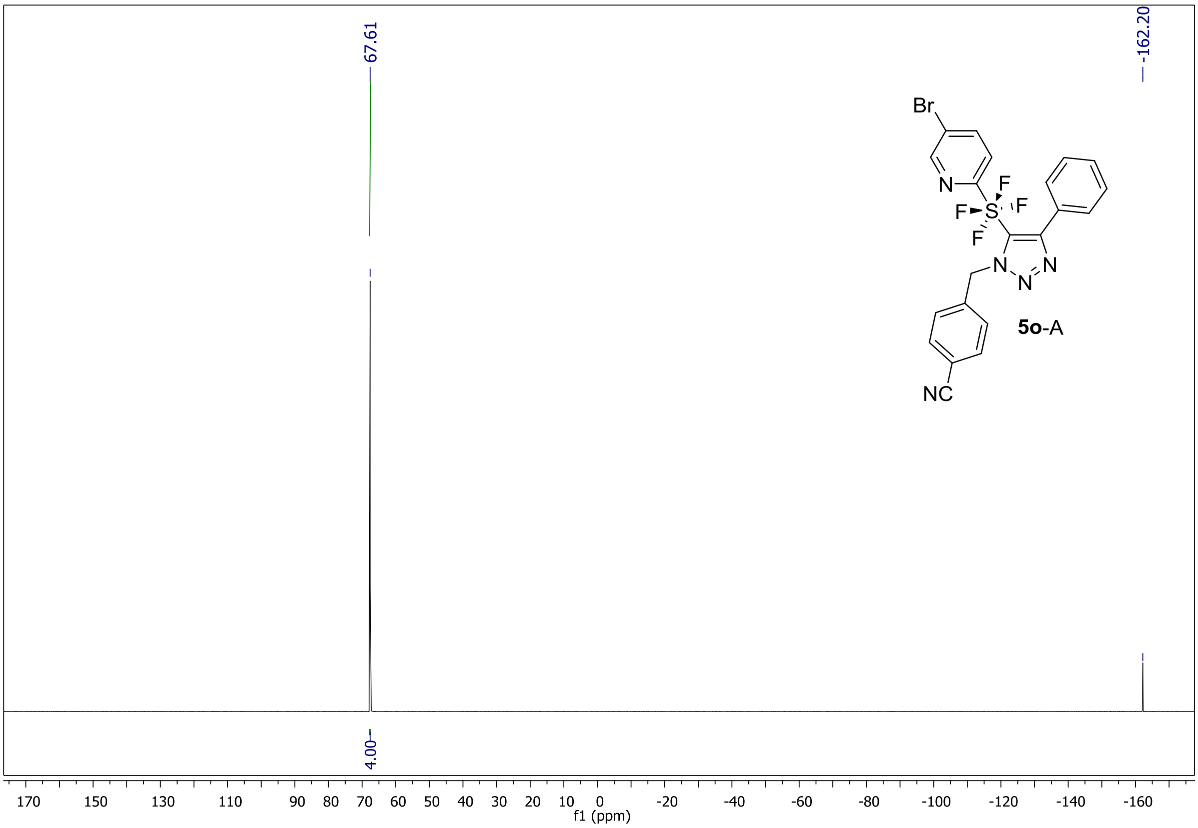

67.61
-162.20
Br
N
F
S
N
N
N
NC
5o-A
4.00
170 150 130 110 90 80 70 60 50 40 30 20 10 0 -20 -40 -60 -80 -100 -120 -140 -160
f1 (ppm)

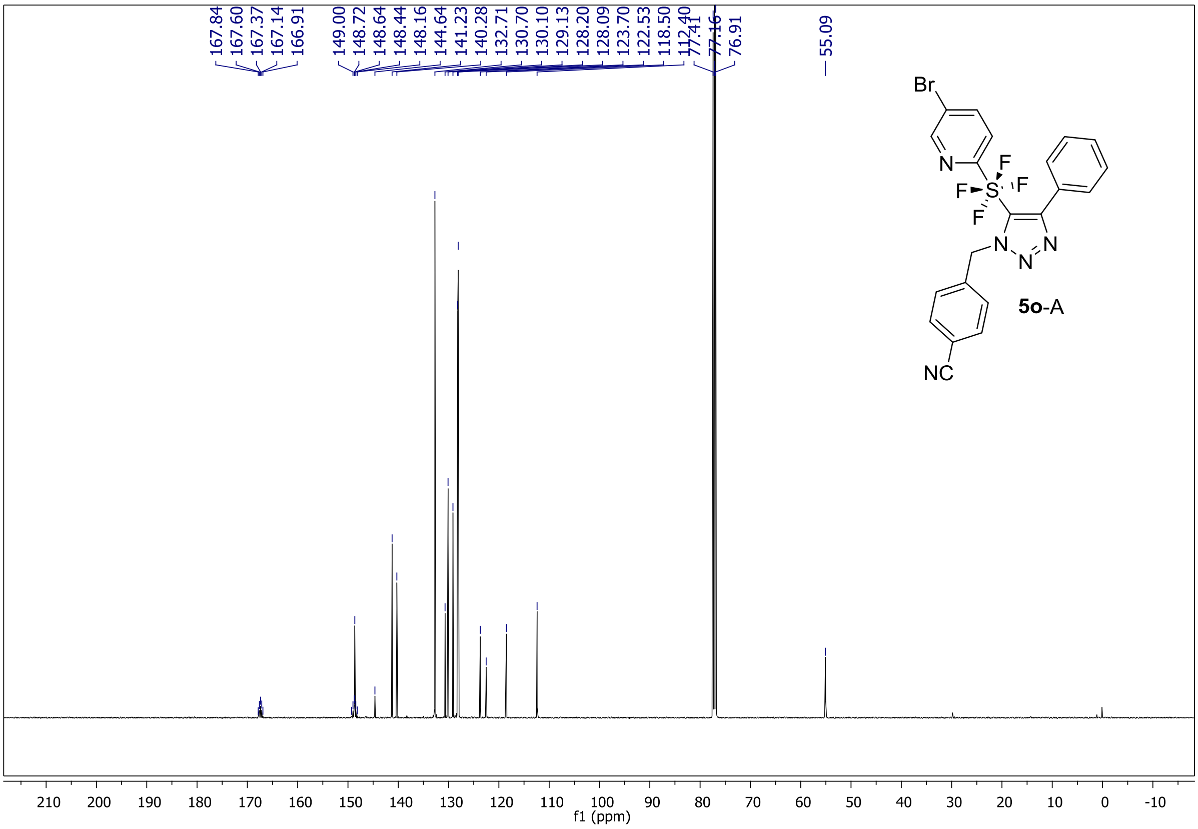

167.84
167.60
167.37
167.14
166.91
149.00
148.72
148.64
148.44
148.16
144.64
141.23
140.28
132.71
130.70
130.10
129.13
128.20
128.09
123.70
122.53
118.50
112.40
77.41
77.16
76.91
55.09
Br
N
F
S
N
N
N
NC
5o-A
210 200 190 180 170 160 150 140 130 120 110 100 90 80 70 60 50 40 30 20 10 0 -10
f1 (ppm)

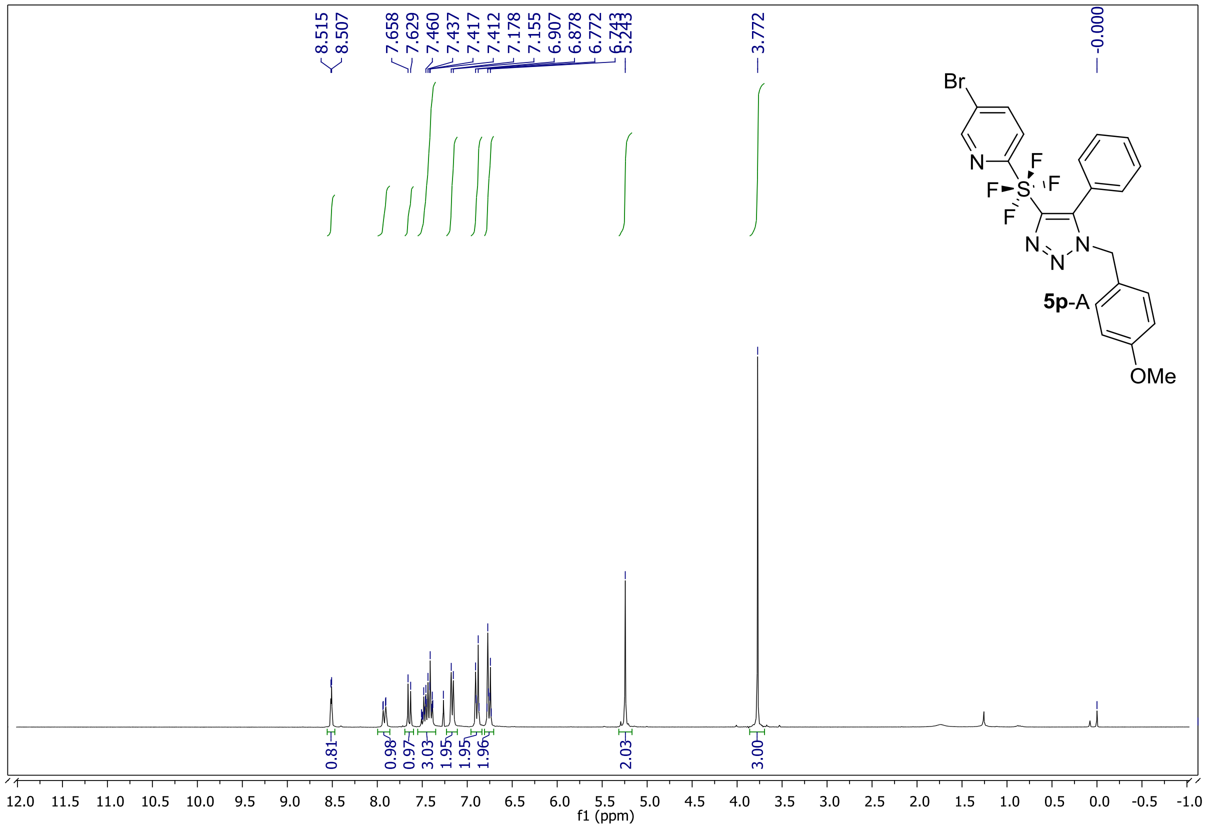

8.515
8.507
7.658
7.629
7.460
7.437
7.417
7.412
7.178
7.155
6.907
6.878
6.772
6.743
5.243
3.772
-0.000
0.81
0.98
0.97
3.03
1.95
1.95
1.96
2.03
3.00
12.0 11.5 11.0 10.5 10.0 9.5 9.0 8.5 8.0 7.5 7.0 6.5 6.0 5.5 5.0 4.5 4.0 3.5 3.0 2.5 2.0 1.5 1.0 0.5 0.0 -0.5 -1.0
f1 (ppm)
Br
N
F
F
F
F
S
N
N
N
5p-A
OMe

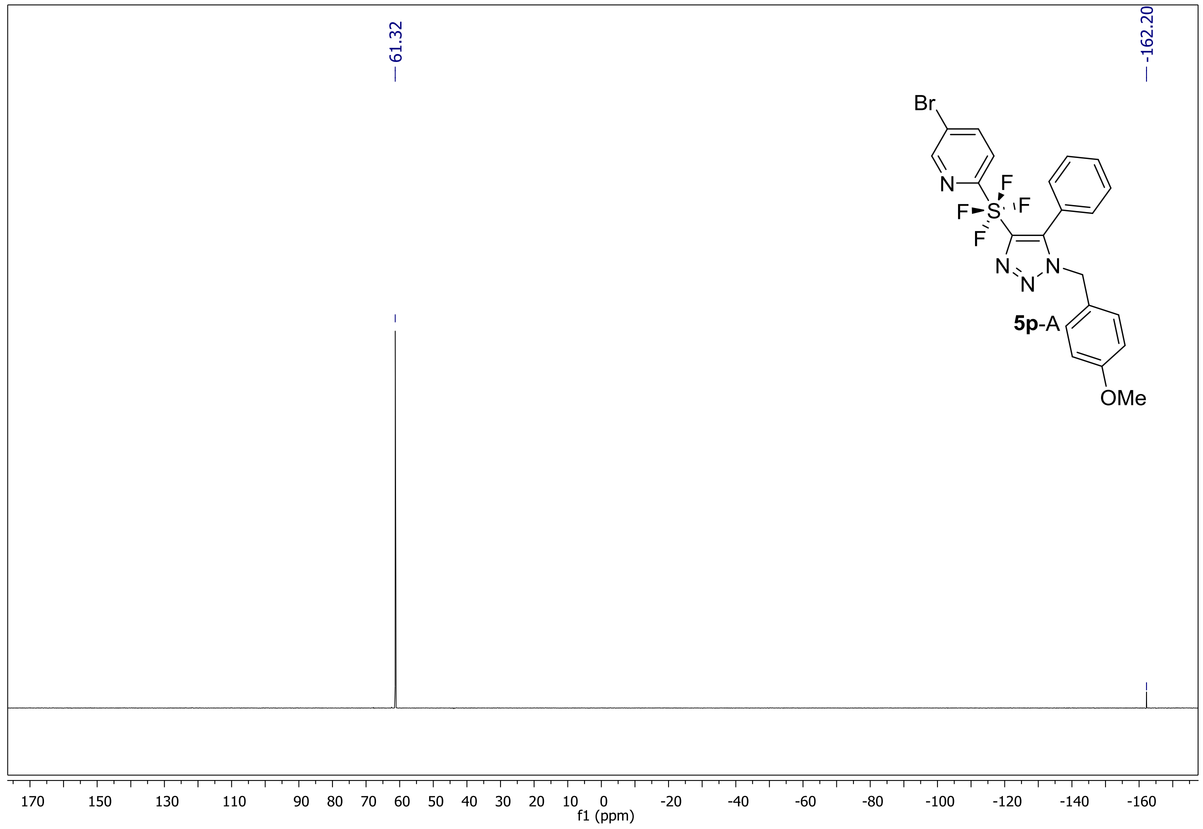

61.32
-162.20
170 150 130 110 90 80 70 60 50 40 30 20 10 0 -20 -40 -60 -80 -100 -120 -140 -160
f1 (ppm)
Br
N
F
F
F
F
S
N
N
N
5p-A
OMe

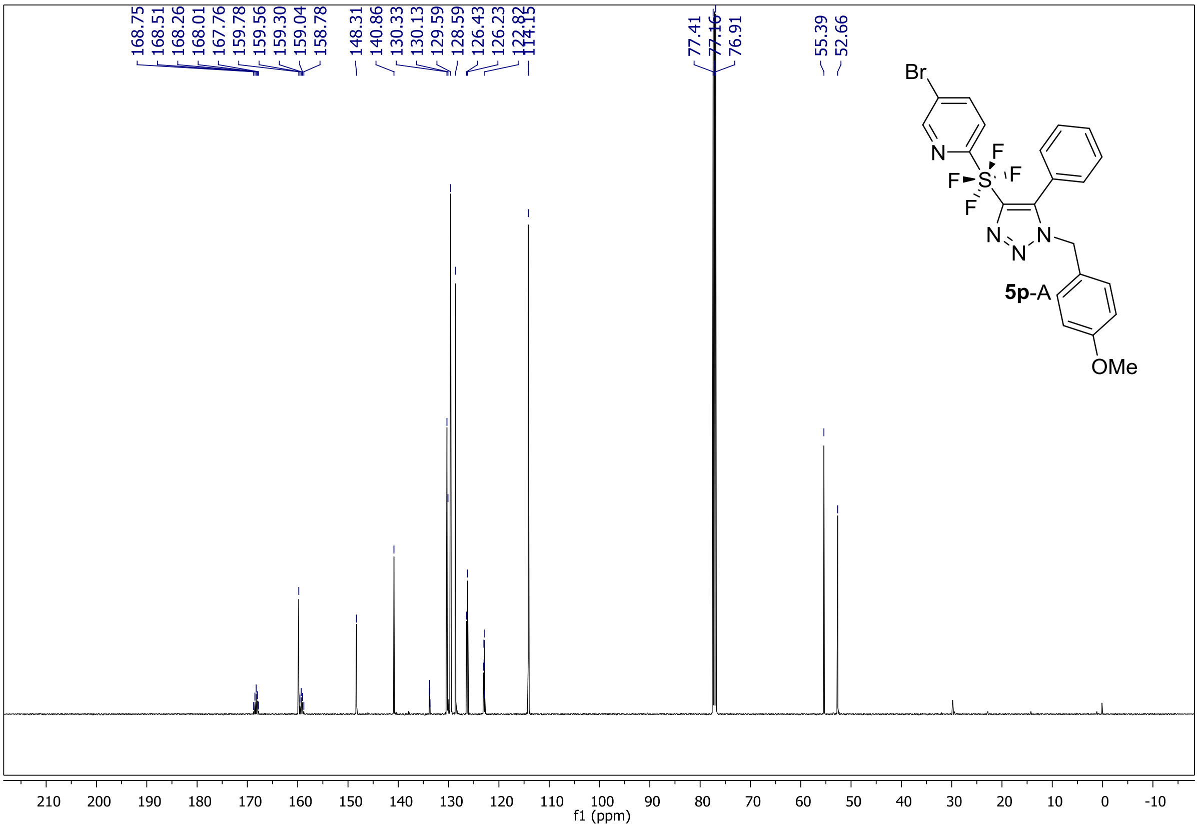
168.75
168.51
168.26
168.01
167.76
159.78
159.56
159.30
159.04
158.78
148.31
140.86
130.33
130.13
129.59
128.59
126.43
126.23
122.82
114.15
77.41
77.16
76.91
55.39
52.66
Br
N
F
F
F
S
F
N
N
N
5p-A
OMe
f1 (ppm)

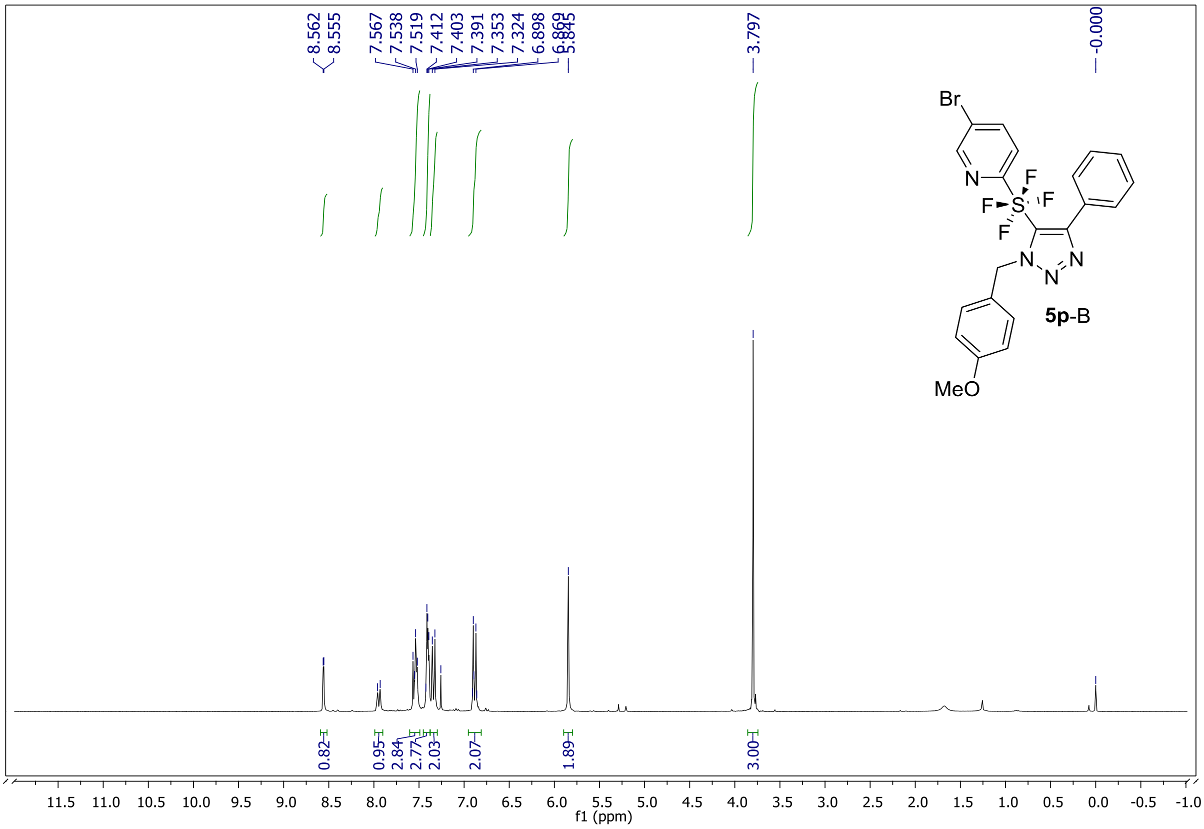
8.562
8.555
7.567
7.538
7.519
7.412
7.403
7.391
7.353
7.324
6.898
6.869
5.845
3.797
-0.000
Br
N
F
F
F
S
F
N
N
N
5p-B
MeO
0.82
0.95
2.84
2.77
2.03
2.07
1.89
3.00
f1 (ppm)

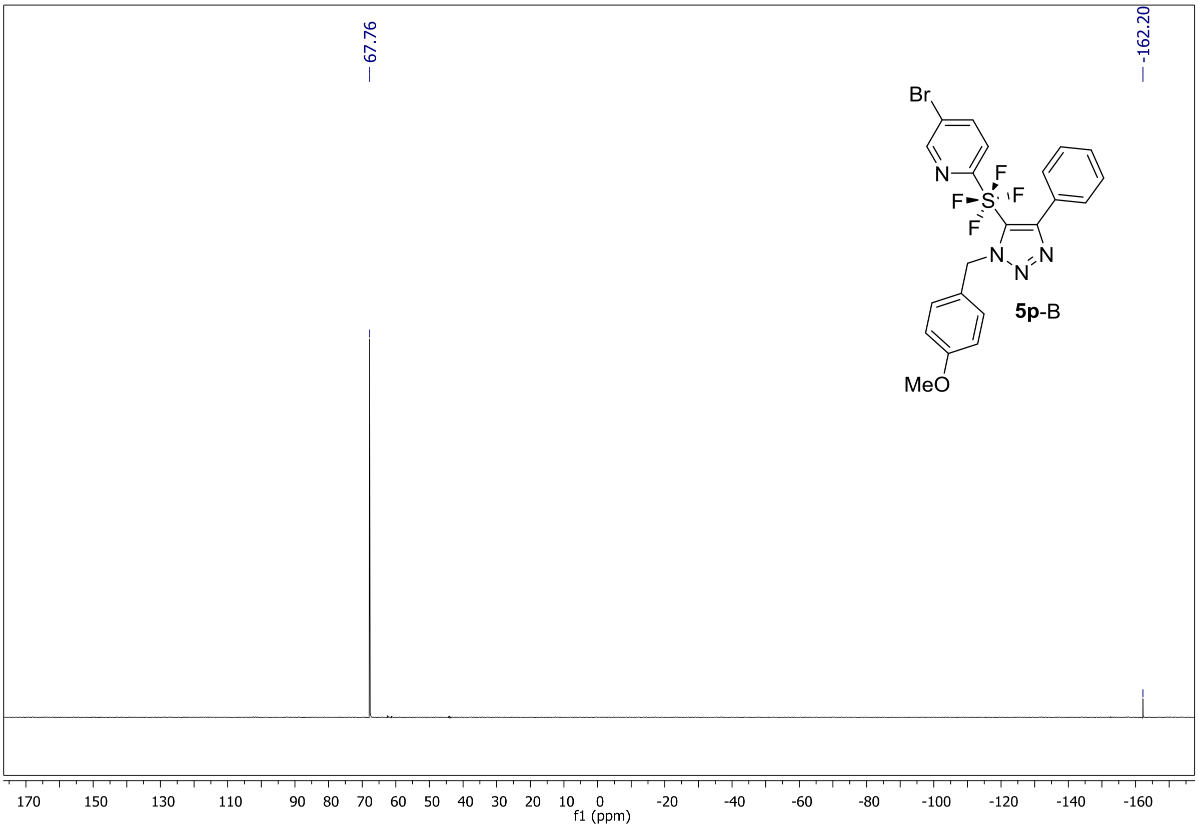
67.76
-162.20
Br
N
F
F
S
F
F
N
N
N
MeO
5p-B
170 150 130 110 90 80 70 60 50 40 30 20 10 0 -20 -40 -60 -80 -100 -120 -140 -160
f1 (ppm)

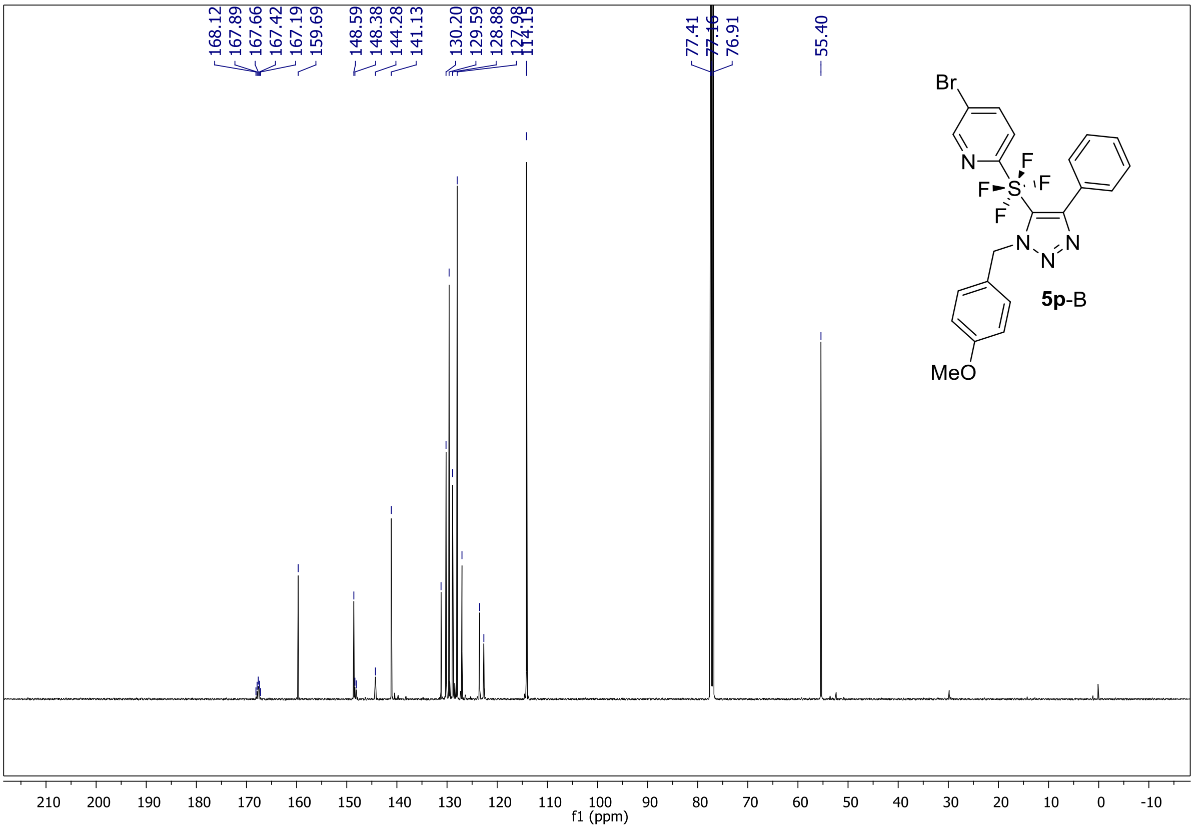
168.12
167.89
167.66
167.42
167.19
159.69
148.59
148.38
144.28
141.13
130.20
129.59
128.88
127.98
114.15
77.41
77.16
76.91
55.40
Br
N
F
F
S
F
F
N
N
N
MeO
5p-B
210 200 190 180 170 160 150 140 130 120 110 100 90 80 70 60 50 40 30 20 10 0 -10
f1 (ppm)

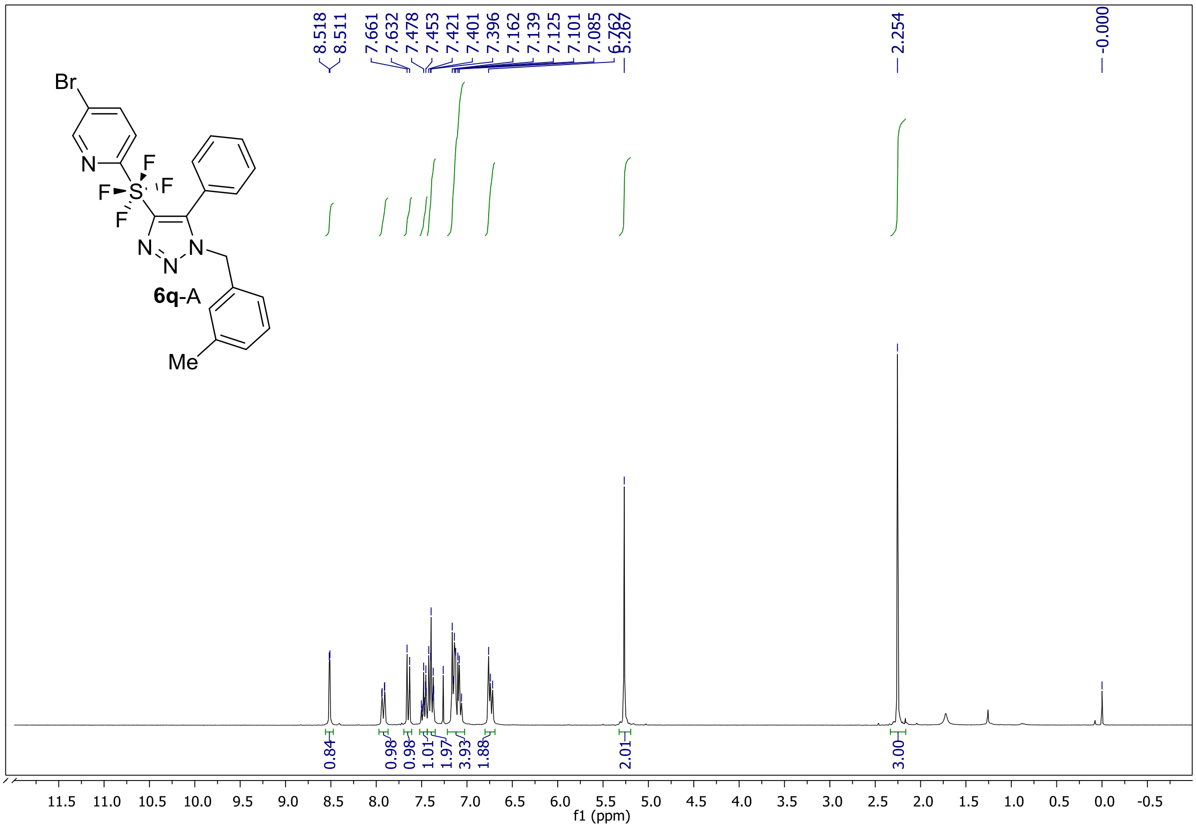
8.518
8.511
7.661
7.632
7.478
7.453
7.421
7.401
7.396
7.162
7.139
7.125
7.101
7.085
6.762
5.267
2.254
-0.000
Br
N
F
F
F
F
S
N
N
N
6q-A
Me
0.84
0.98
0.98
1.01
1.97
3.93
1.88
2.01
3.00
11.5 11.0 10.5 10.0 9.5 9.0 8.5 8.0 7.5 7.0 6.5 6.0 5.5 5.0 4.5 4.0 3.5 3.0 2.5 2.0 1.5 1.0 0.5 0.0 -0.5
f1 (ppm)

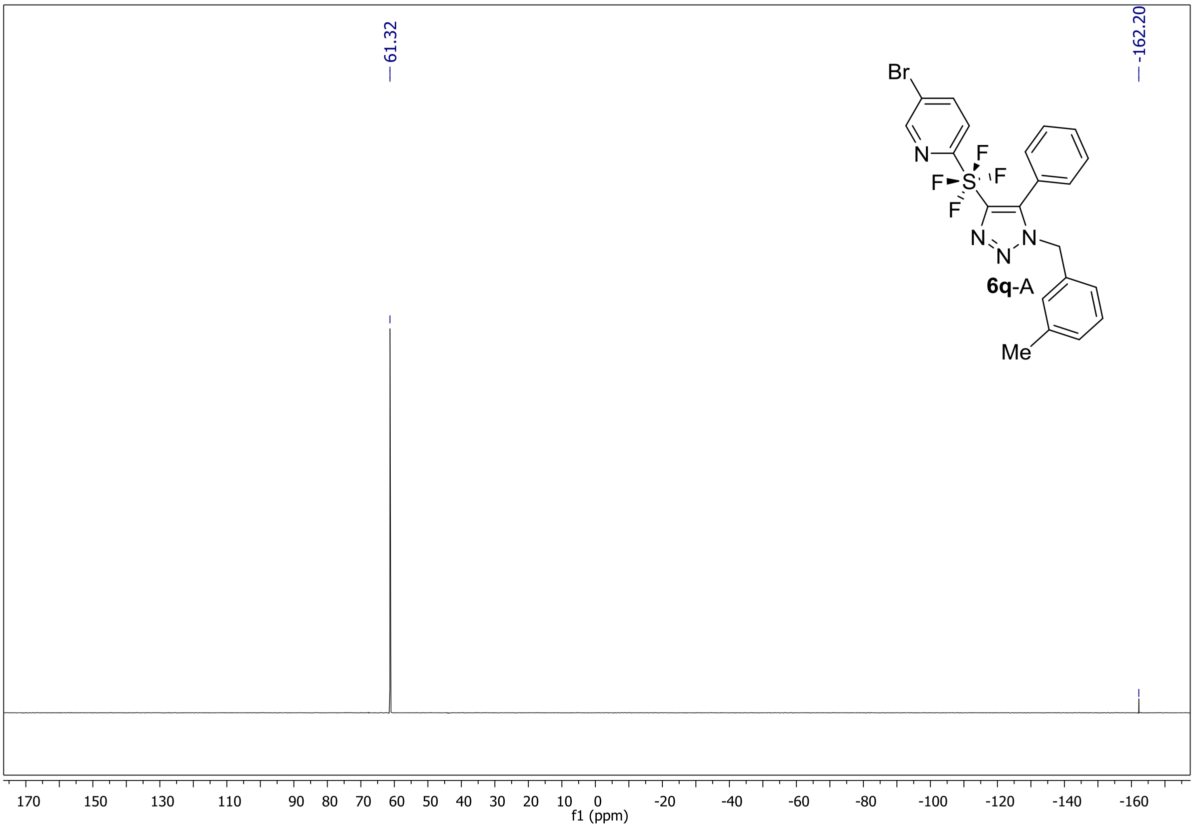
61.32
-162.20
Br
N
F
F
F
F
S
N
N
N
6q-A
Me
170 150 130 110 90 80 70 60 50 40 30 20 10 0 -20 -40 -60 -80 -100 -120 -140 -160
f1 (ppm)

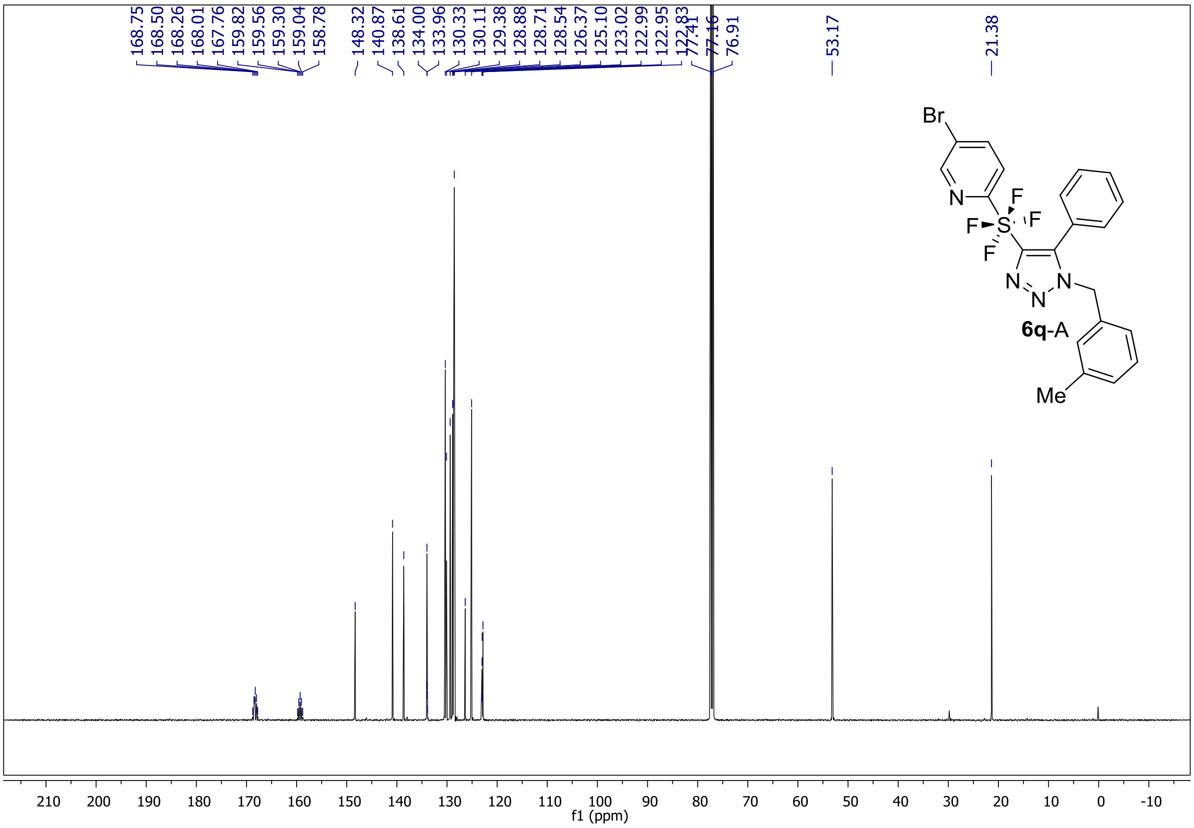
168.75
168.50
168.26
168.01
167.76
159.82
159.56
159.30
159.04
158.78
148.32
140.87
138.61
134.00
133.96
130.33
130.11
129.38
128.88
128.71
128.54
126.37
125.10
123.02
122.99
122.95
122.83
77.41
77.16
76.91
53.17
21.38
Br
N
F
F
F
F
S
N
N
N
6q-A
Me
f1 (ppm)

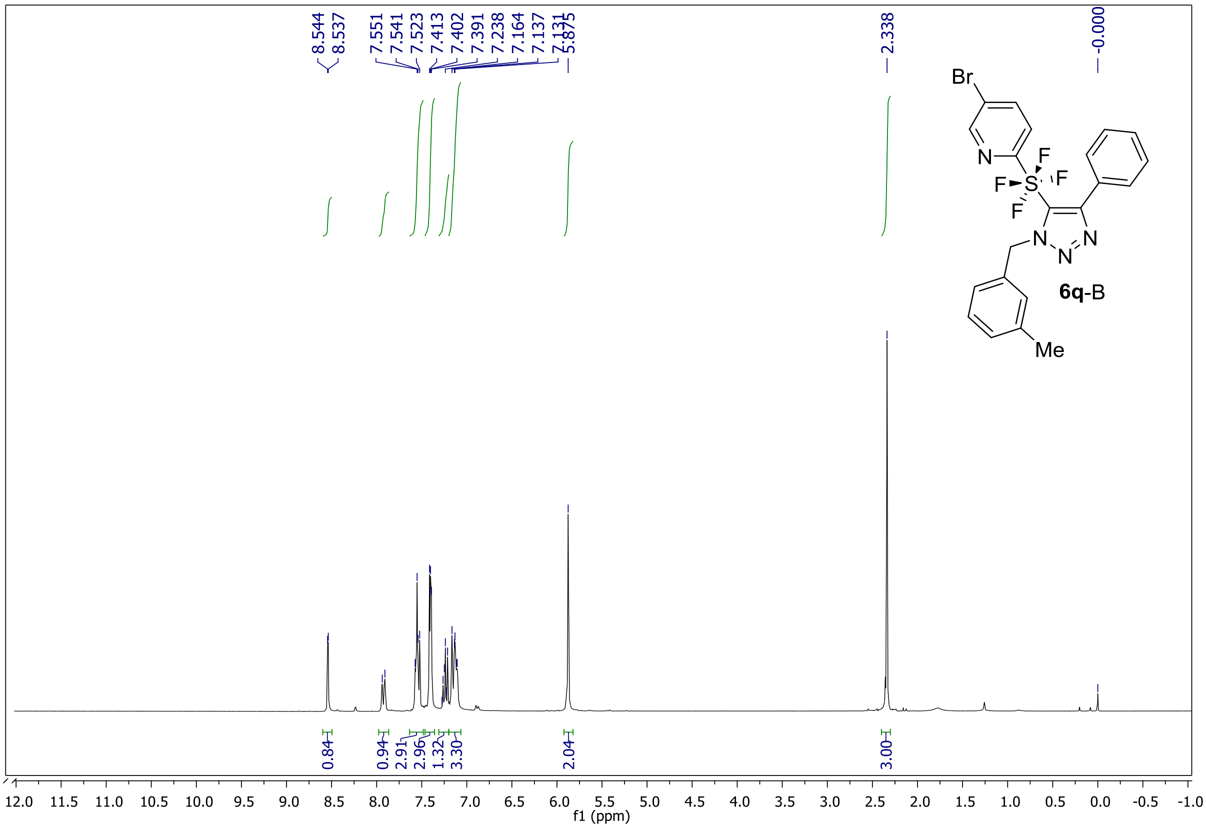
8.544
8.537
7.551
7.541
7.523
7.413
7.402
7.391
7.238
7.164
7.137
7.131
5.875
2.338
-0.000
Br
N
F
F
F
F
S
N
N
N
6q-B
Me
0.84
0.94
2.91
2.96
1.32
3.30
2.04
3.00
f1 (ppm)

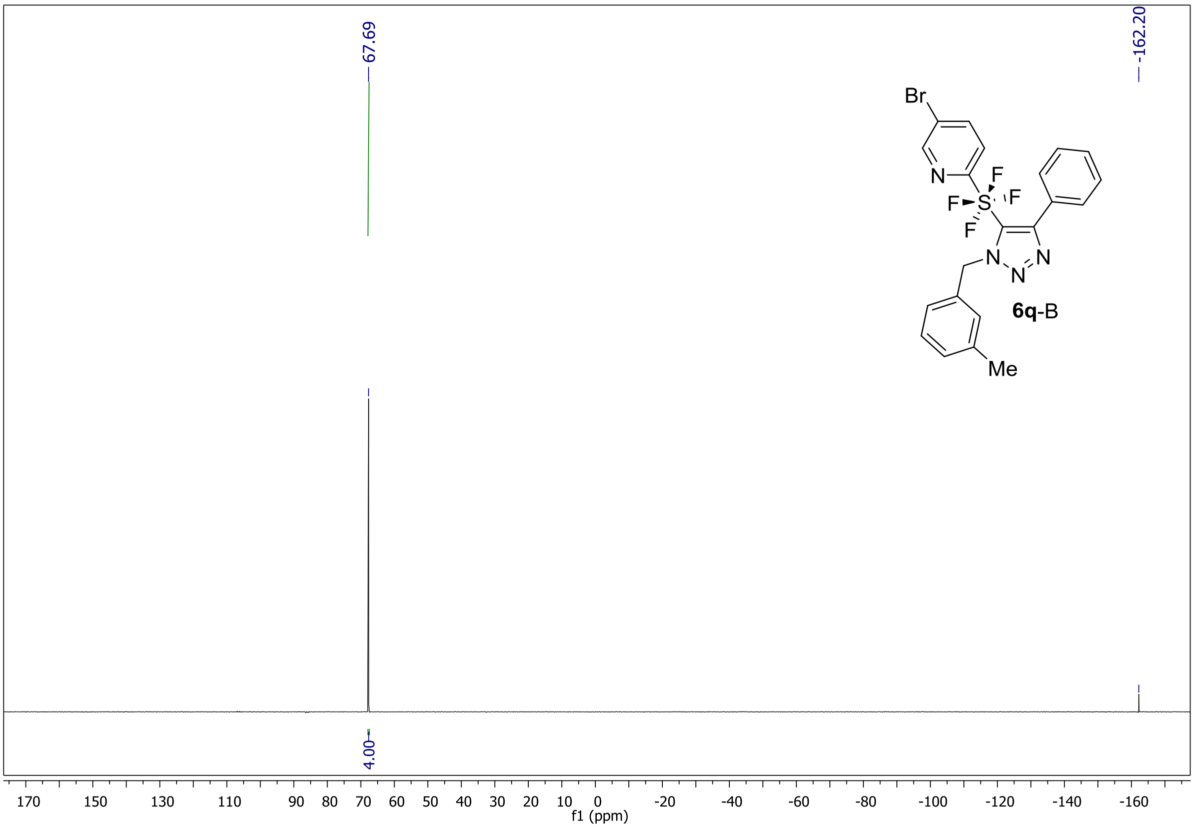
67.69
-162.20
Br
N
F
F
F
F
S
N
N
N
6q-B
Me
4.00
170 150 130 110 90 80 70 60 50 40 30 20 10 0 -20 -40 -60 -80 -100 -120 -140 -160
f1 (ppm)

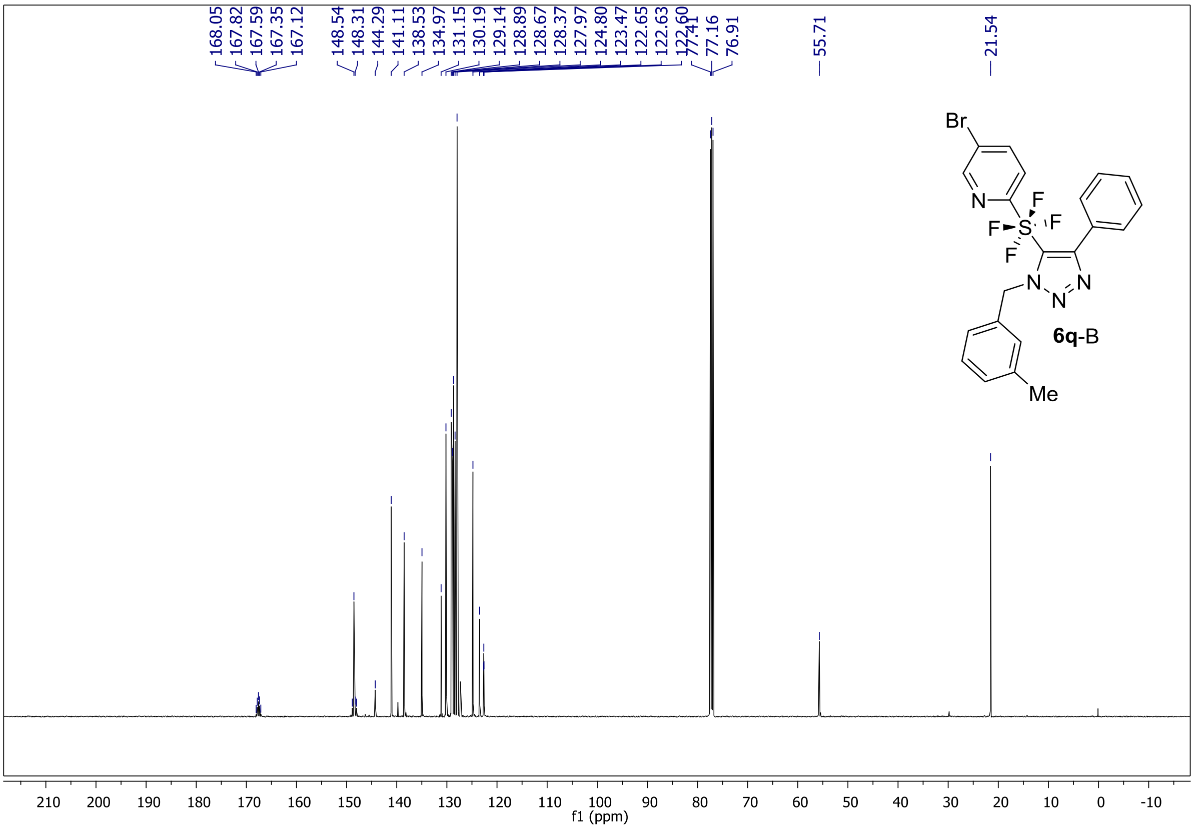
168.05
167.82
167.59
167.35
167.12
148.54
148.31
144.29
141.11
138.53
134.97
131.15
130.19
129.14
128.89
128.67
128.37
127.97
124.80
123.47
122.65
122.63
122.60
77.41
77.16
76.91
55.71
21.54
Br
N
F
F
F
F
S
N
N
N
6q-B
Me
210 200 190 180 170 160 150 140 130 120 110 100 90 80 70 60 50 40 30 20 10 0 -10
f1 (ppm)

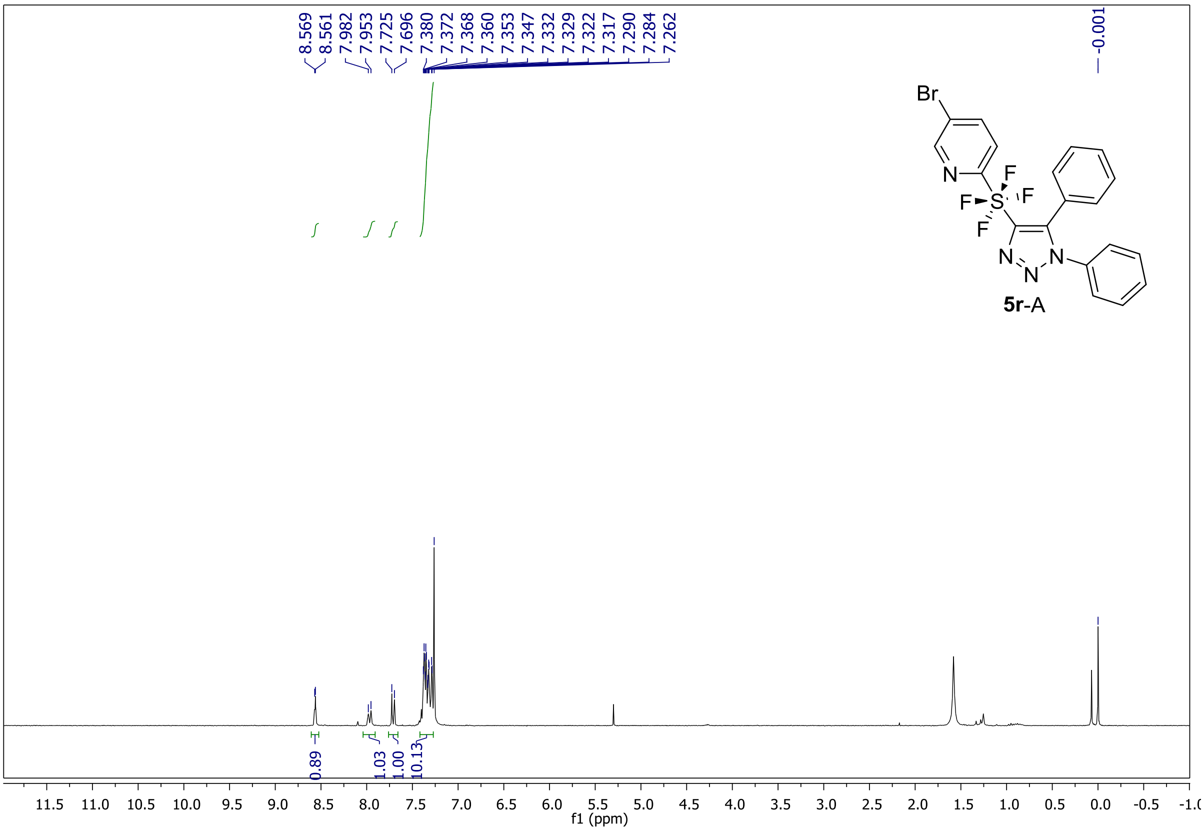
8.569
8.561
7.982
7.953
7.725
7.696
7.380
7.372
7.368
7.360
7.353
7.347
7.332
7.329
7.322
7.317
7.290
7.284
7.262
-0.001
Br
N
F
F
S
F
F
N
N
N
5r-A
0.89
1.03
1.00
10.13
11.5 11.0 10.5 10.0 9.5 9.0 8.5 8.0 7.5 7.0 6.5 6.0 5.5 5.0 4.5 4.0 3.5 3.0 2.5 2.0 1.5 1.0 0.5 0.0 -0.5
f1 (ppm)

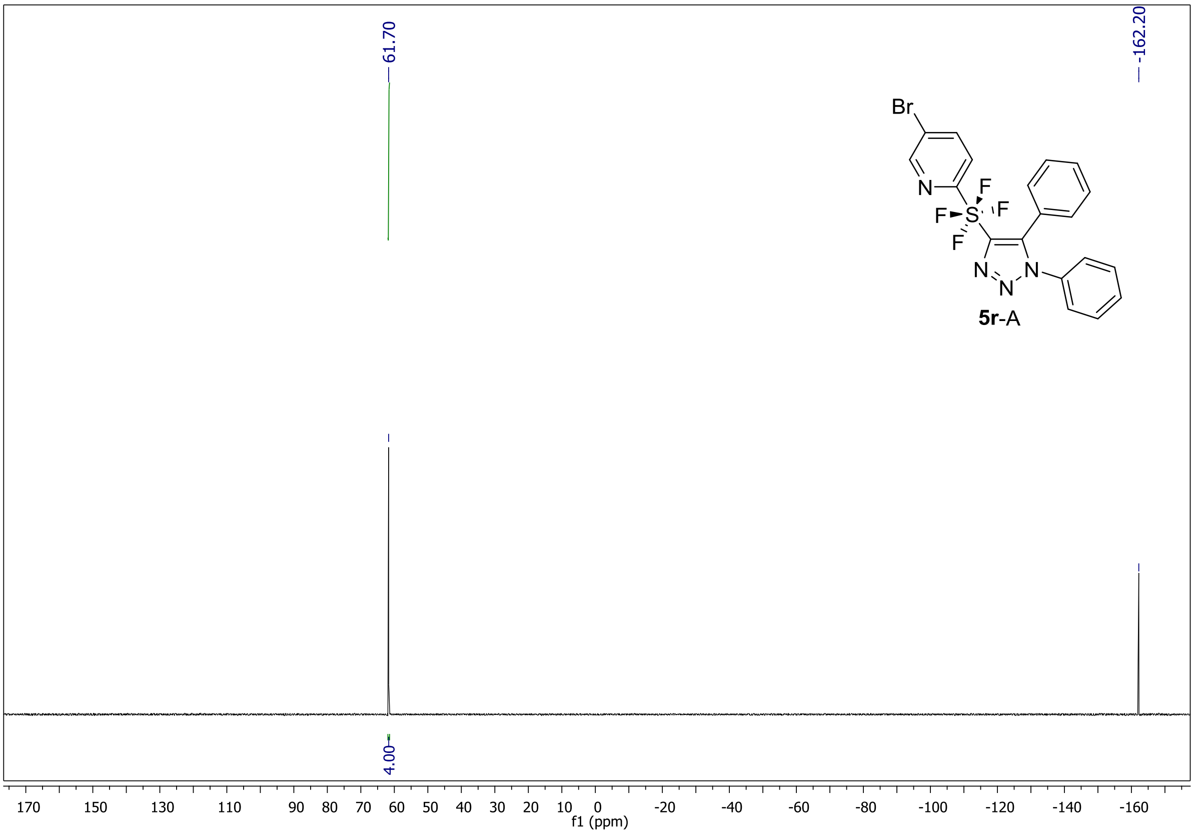
61.70
-162.20
Br
N
F
F
S
F
F
N
N
N
5r-A
4.00
170 150 130 110 90 80 70 60 50 40 30 20 10 0 -20 -40 -60 -80 -100 -120 -140 -160
f1 (ppm)

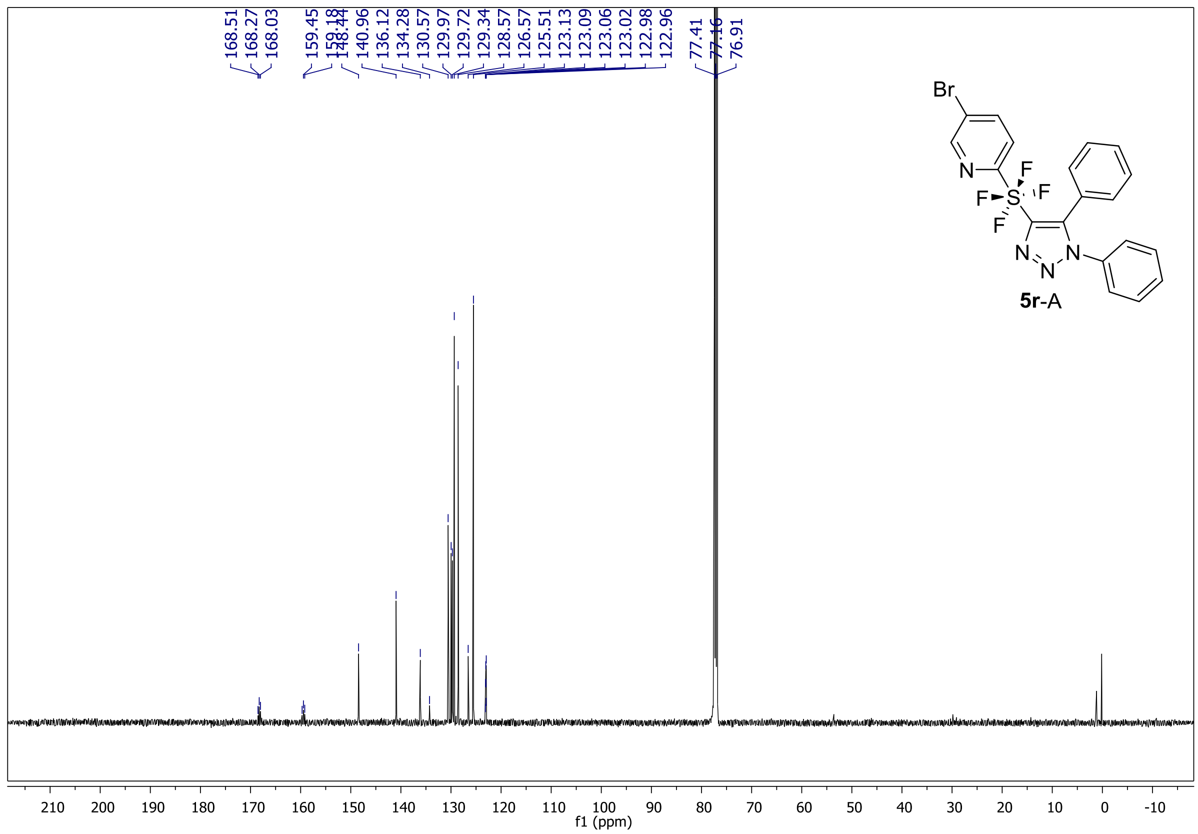
168.51
168.27
168.03
159.45
159.18
148.44
140.96
136.12
134.28
130.57
129.97
129.72
129.34
128.57
126.57
125.51
123.13
123.09
123.06
123.02
122.98
122.96
77.41
77.16
76.91
Br
N
F
F
F
S
F
N
N
N
5r-A
210 200 190 180 170 160 150 140 130 120 110 100 90 80 70 60 50 40 30 20 10 0 -10
f1 (ppm)

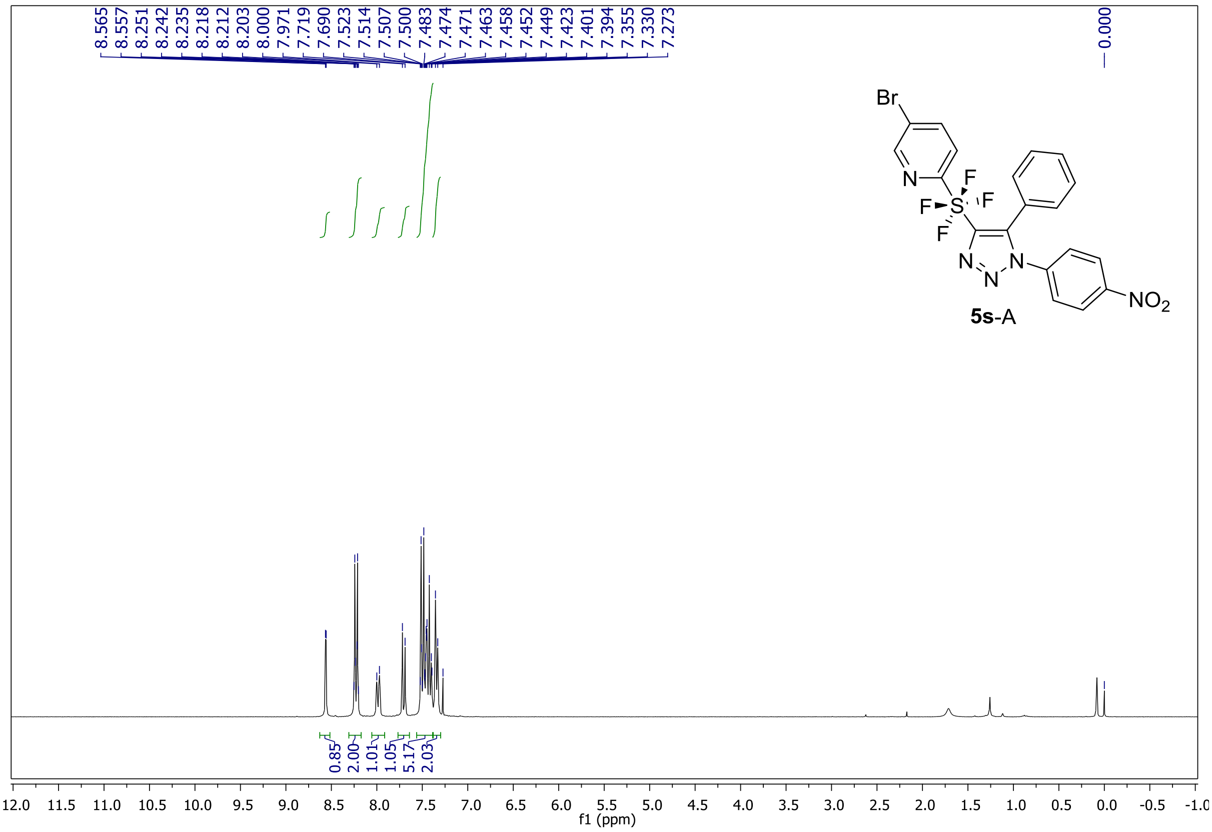
8.565
8.557
8.251
8.242
8.235
8.218
8.212
8.203
8.000
7.971
7.719
7.690
7.523
7.514
7.507
7.500
7.483
7.474
7.471
7.463
7.458
7.452
7.449
7.423
7.401
7.394
7.355
7.330
7.273
0.000
Br
N
F
F
F
S
F
N
N
N
NO2
5s-A
0.85
2.00
1.01
1.05
5.17
2.03
12.0 11.5 11.0 10.5 10.0 9.5 9.0 8.5 8.0 7.5 7.0 6.5 6.0 5.5 5.0 4.5 4.0 3.5 3.0 2.5 2.0 1.5 1.0 0.5 0.0 -0.5 -1.0
f1 (ppm)

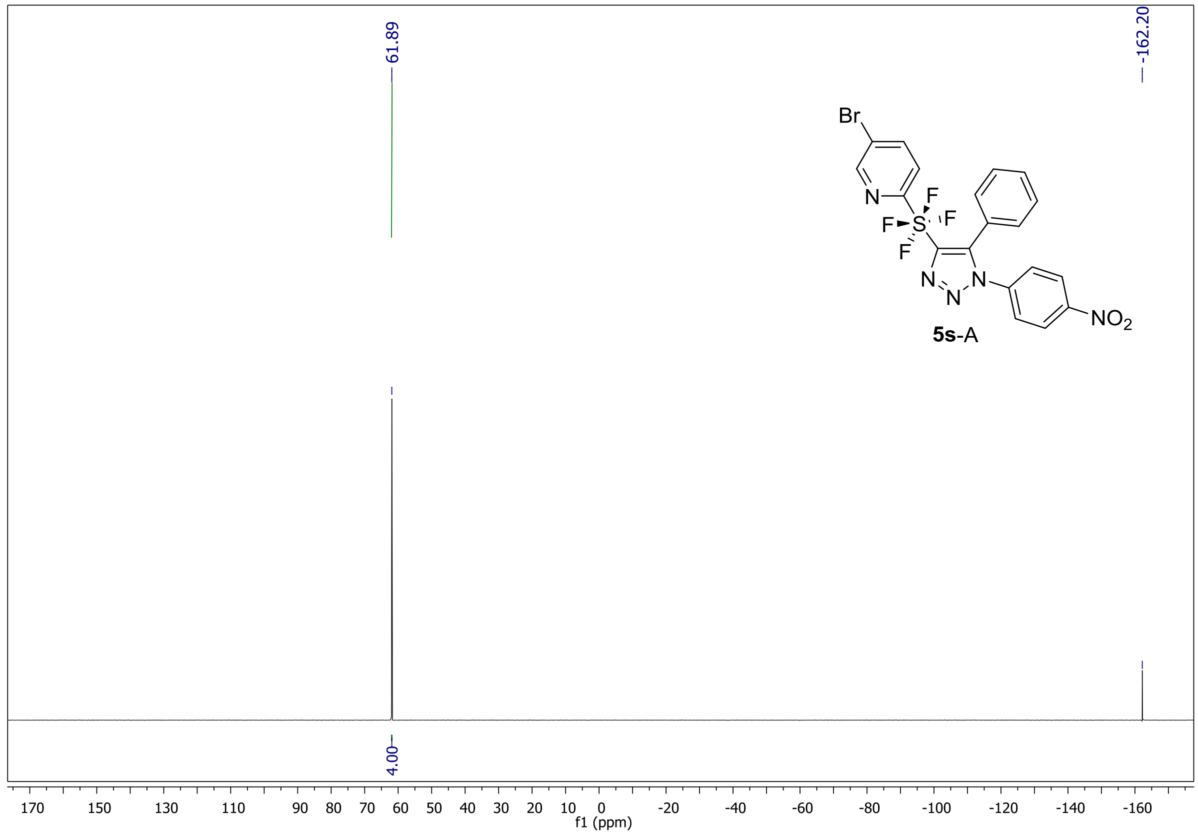
61.89
-162.20
Br
N
F
F
F
F
S
N
N
N
NO2
5s-A
4.00
170 150 130 110 90 80 70 60 50 40 30 20 10 0 -20 -40 -60 -80 -100 -120 -140 -160
f1 (ppm)

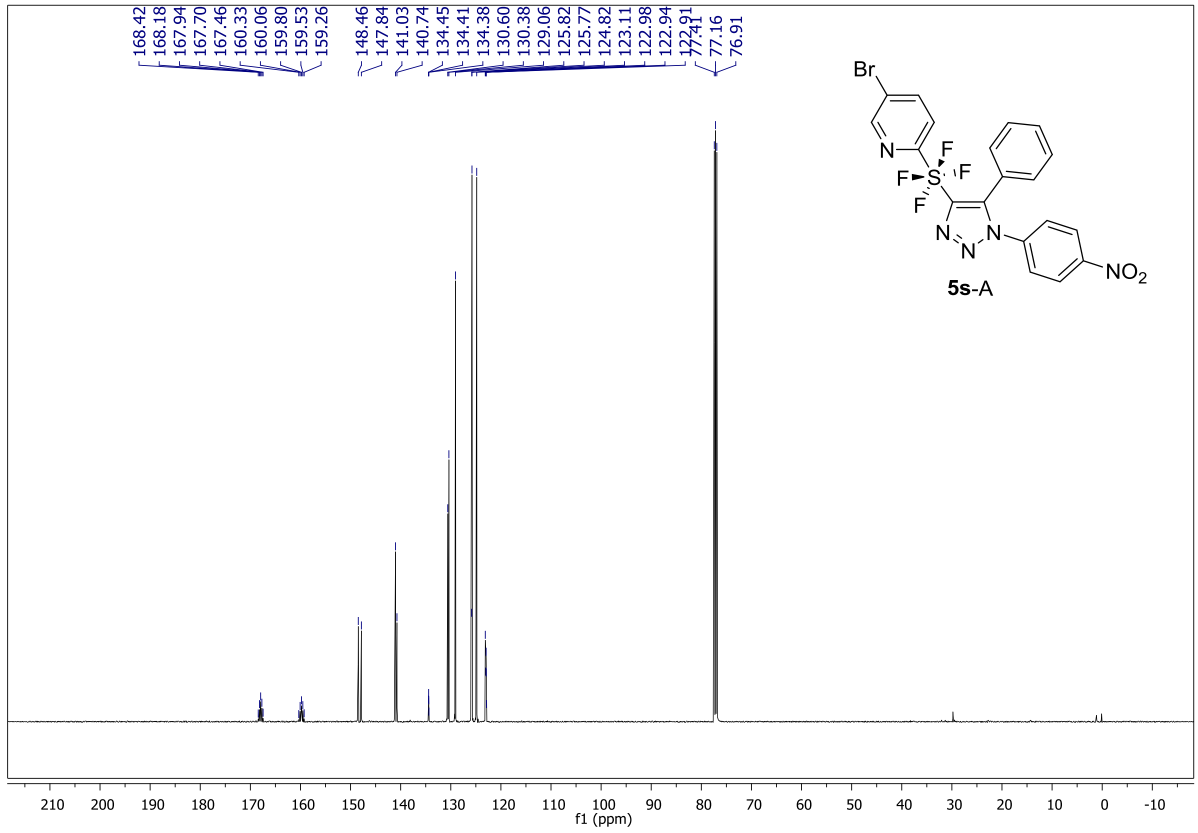
168.42
168.18
167.94
167.70
167.46
160.33
160.06
159.80
159.53
159.26
148.46
147.84
141.03
140.74
134.45
134.41
134.38
130.60
130.38
129.06
125.82
125.77
124.82
123.11
122.98
122.94
122.91
77.41
77.16
76.91
Br
N
F
F
F
F
S
N
N
N
NO2
5s-A
210 200 190 180 170 160 150 140 130 120 110 100 90 80 70 60 50 40 30 20 10 0 -10
f1 (ppm)

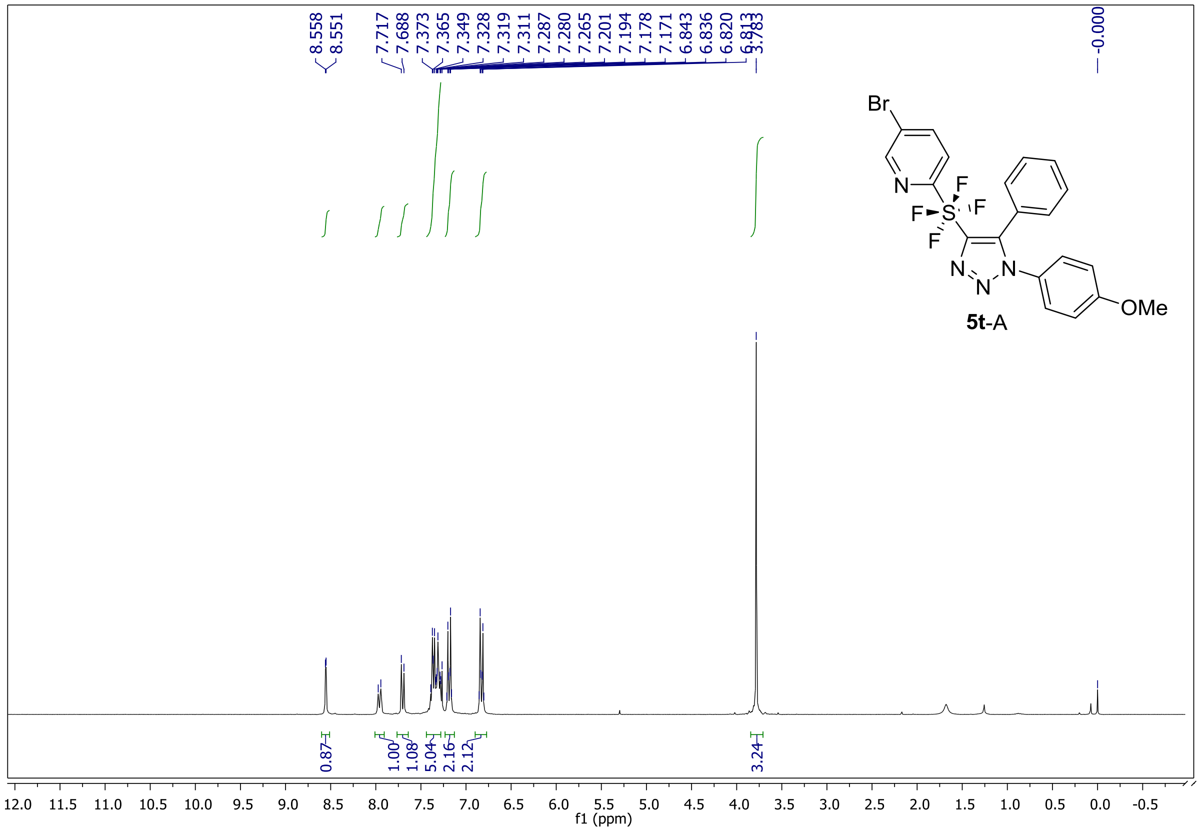
8.558
8.551
7.717
7.688
7.373
7.365
7.349
7.328
7.319
7.311
7.287
7.280
7.265
7.201
7.194
7.178
7.171
6.843
6.836
6.820
6.813
3.783
-0.000
Br
N
F
S
F
F
F
N
N
N
OMe
5t-A
0.87
1.00
1.08
5.04
2.16
2.12
3.24
f1 (ppm)

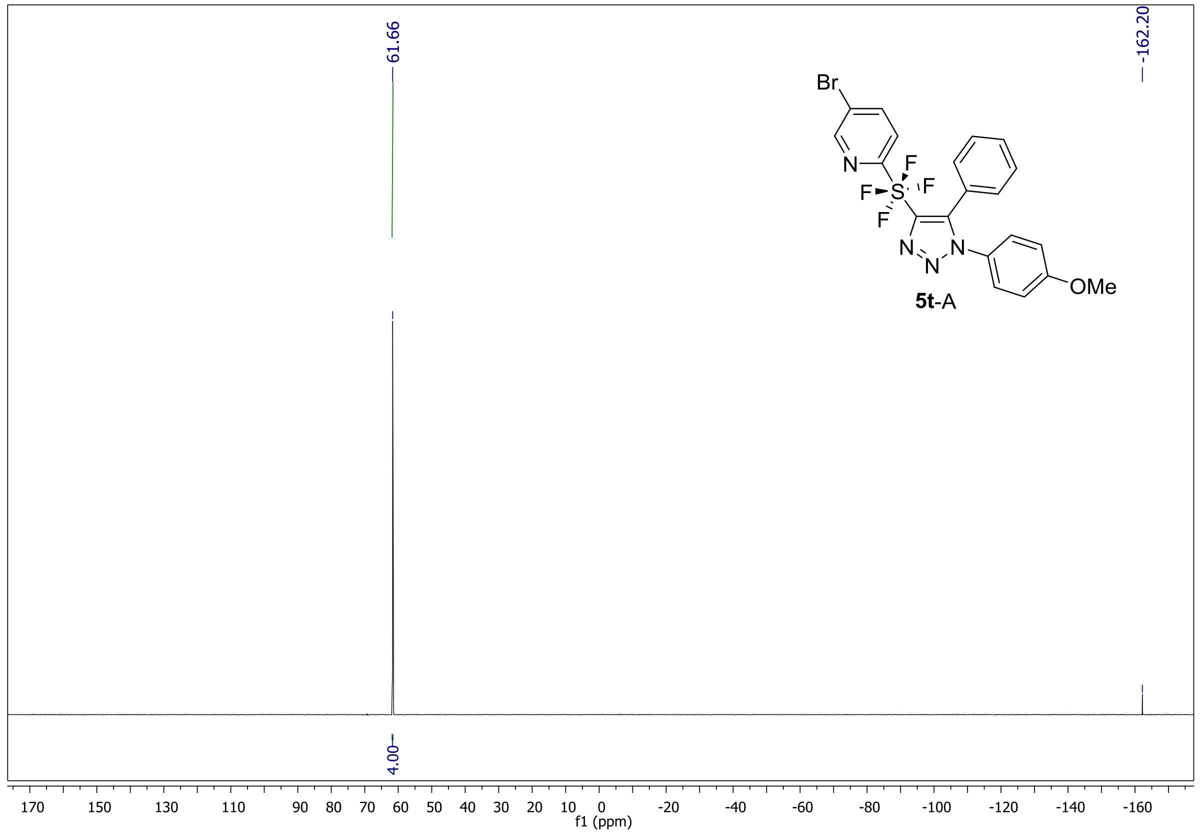
61.66
-162.20
Br
N
F
S
F
F
F
N
N
N
OMe
5t-A
4.00
f1 (ppm)

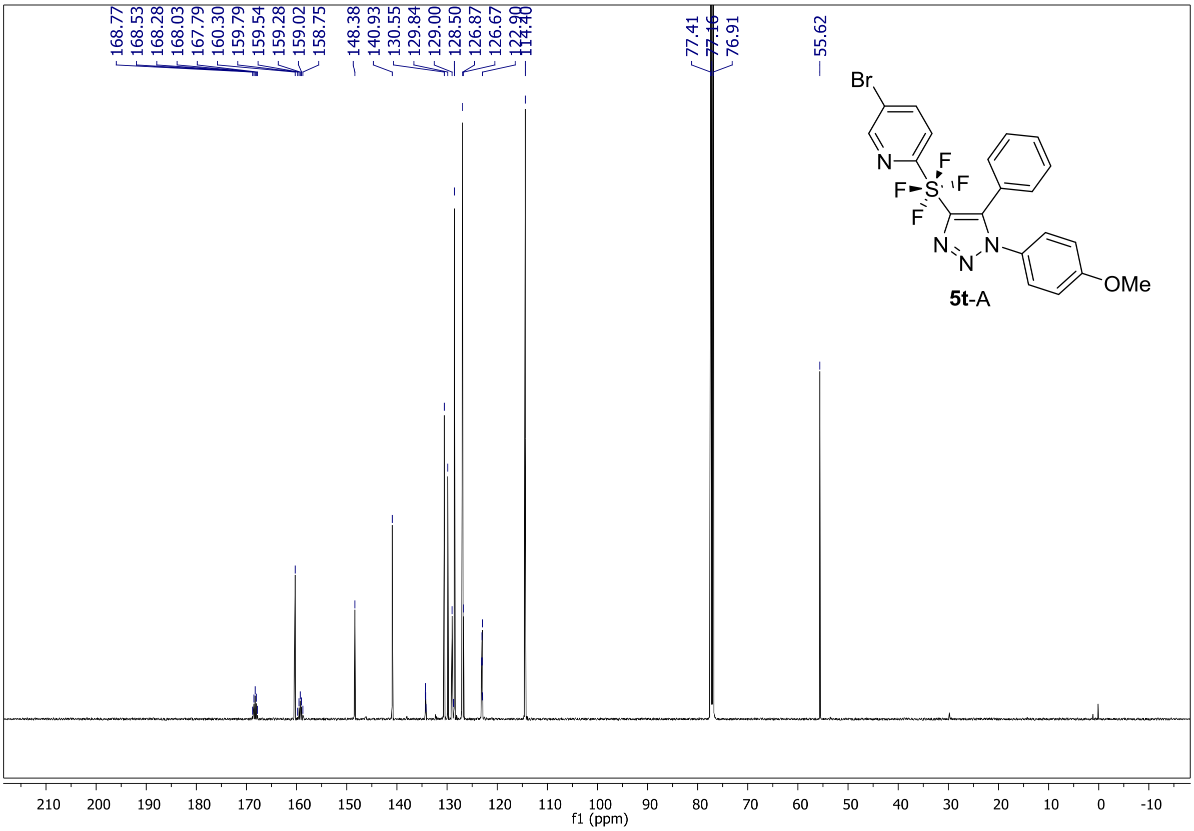

168.77
168.53
168.28
168.03
167.79
160.30
159.79
159.54
159.28
159.02
158.75
148.38
140.93
130.55
129.84
129.00
128.50
126.87
126.67
122.90
114.40
77.41
77.16
76.91
55.62
Br
N
F
F
F
F
S
N
N
N
OMe
5t-A
210
200
190
180
170
160
150
140
130
120
110
100
90
80
70
60
50
40
30
20
10
0
-10
f1 (ppm)

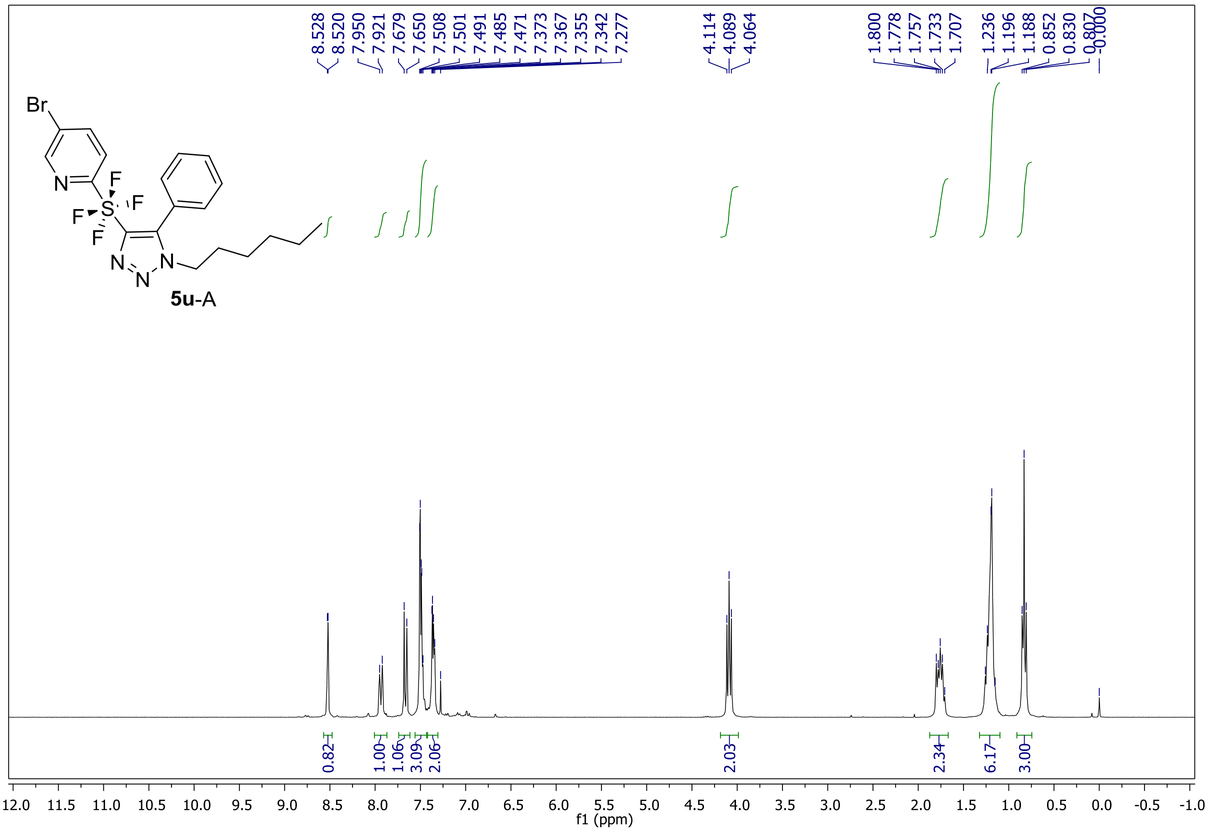

8.528
8.520
7.950
7.921
7.679
7.650
7.508
7.501
7.491
7.485
7.471
7.373
7.367
7.355
7.342
7.277
4.114
4.089
4.064
1.800
1.778
1.757
1.733
1.707
1.236
1.196
1.188
0.852
0.830
0.807
-0.000
Br
N
F
F
F
F
S
N
N
N
5u-A
0.82
1.00
1.06
3.09
2.06
2.03
2.34
6.17
3.00
12.0
11.5
11.0
10.5
10.0
9.5
9.0
8.5
8.0
7.5
7.0
6.5
6.0
5.5
5.0
4.5
4.0
3.5
3.0
2.5
2.0
1.5
1.0
0.5
0.0
-0.5
-1.0
f1 (ppm)

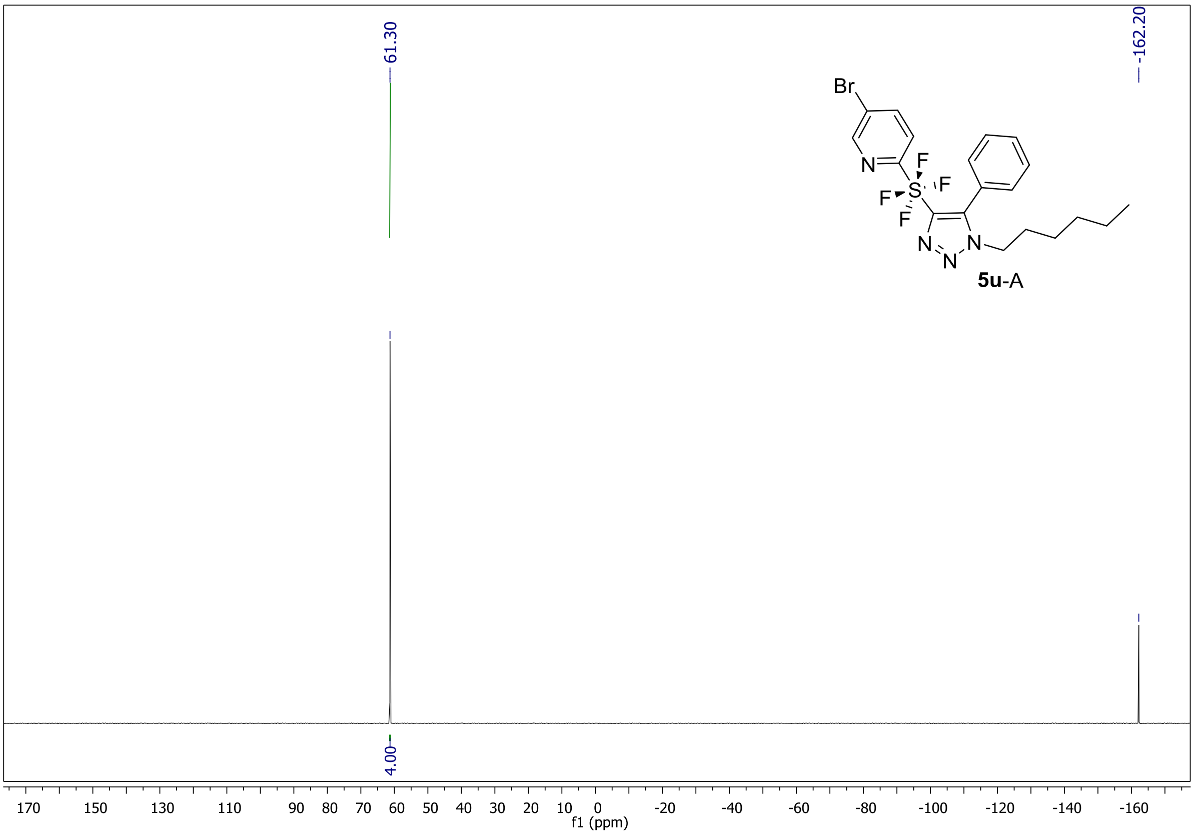
61.30
-162.20
Br
N
F
F
F
F
S
N
N
N
5u-A
4.00
170
150
130
110
90
80
70
60
50
40
30
20
10
0
-20
-40
-60
-80
-100
-120
-140
-160
f1 (ppm)

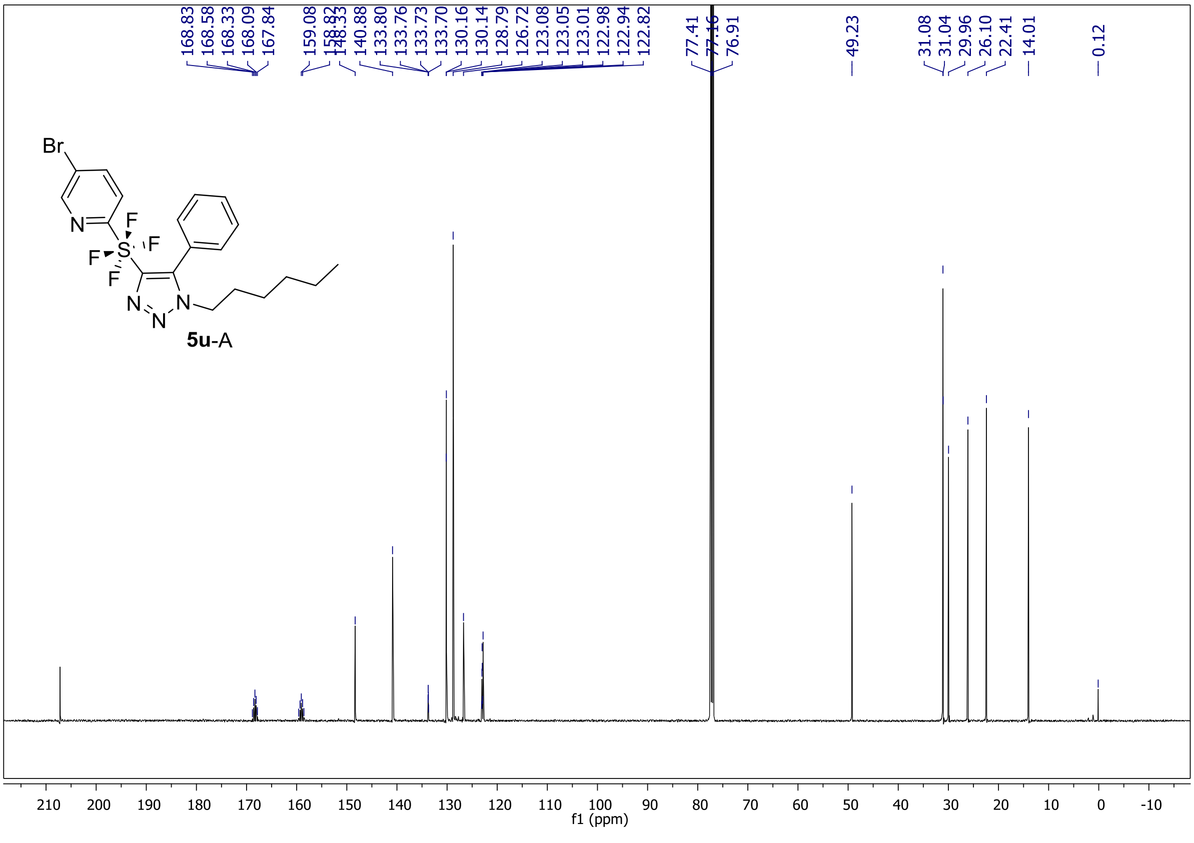
168.83
168.58
168.33
168.09
167.84
159.08
158.82
148.33
140.88
133.80
133.76
133.73
133.70
130.16
130.14
128.79
126.72
123.08
123.05
123.01
122.98
122.94
122.82
77.41
77.16
76.91
49.23
31.08
31.04
29.96
26.10
22.41
14.01
0.12
Br
N
F
F
F
F
S
N
N
N
5u-A
210
200
190
180
170
160
150
140
130
120
110
100
90
80
70
60
50
40
30
20
10
0
-10
f1 (ppm)

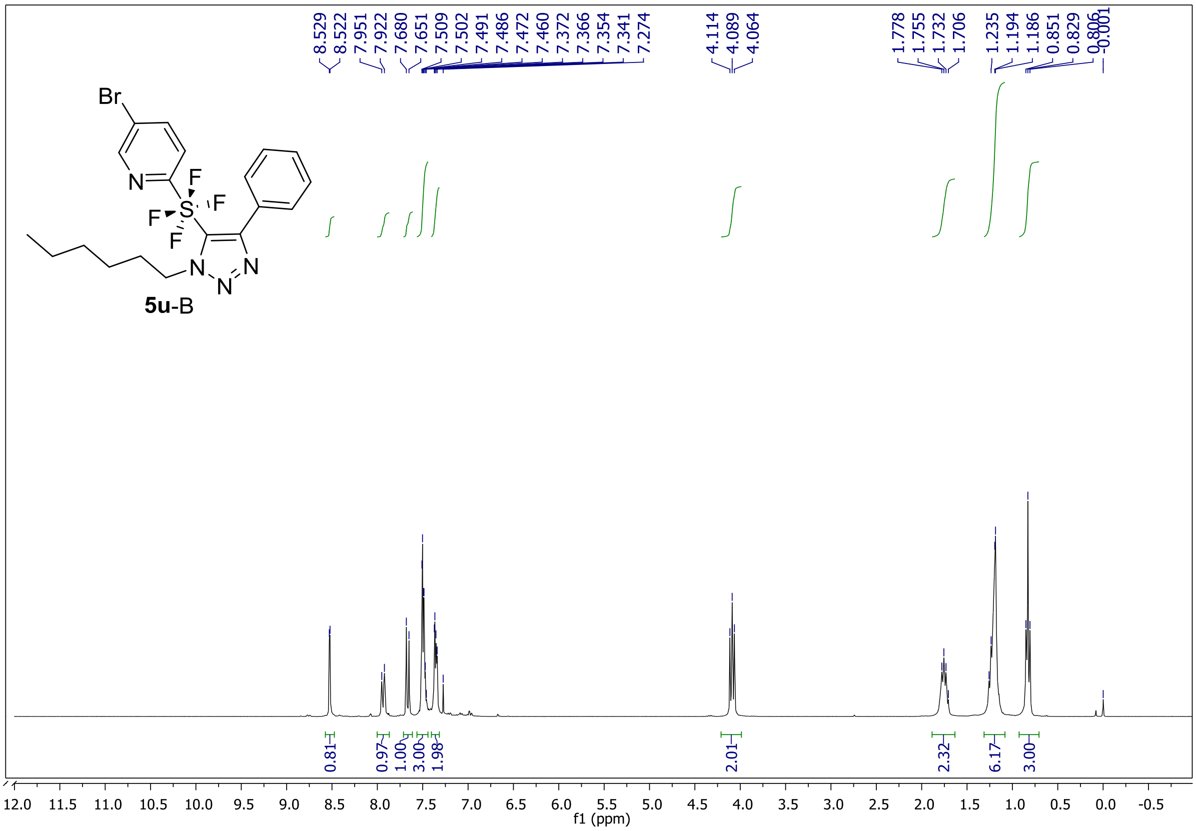

Br
N
F
S
F
F
F
N
N
N
5u-B
8.529
8.522
7.951
7.922
7.680
7.651
7.509
7.502
7.491
7.486
7.472
7.460
7.372
7.366
7.354
7.341
7.274
4.114
4.089
4.064
1.778
1.755
1.732
1.706
1.235
1.194
1.186
0.851
0.829
0.806
-0.001
0.81
0.97
1.00
3.00
1.98
2.01
2.32
6.17
3.00
12.0 11.5 11.0 10.5 10.0 9.5 9.0 8.5 8.0 7.5 7.0 6.5 6.0 5.5 5.0 4.5 4.0 3.5 3.0 2.5 2.0 1.5 1.0 0.5 0.0 -0.5
f1 (ppm)

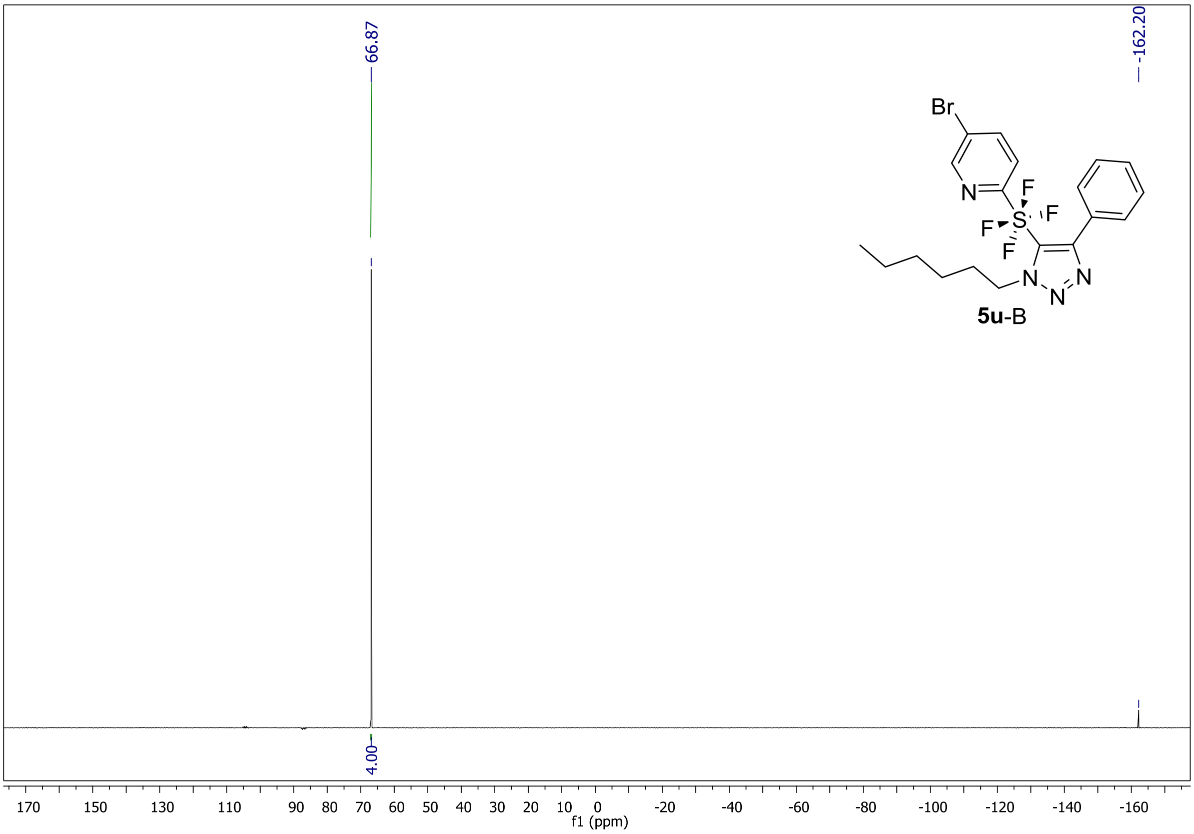

66.87
-162.20
Br
N
F
S
F
F
F
N
N
N
5u-B
4.00
170 150 130 110 90 80 70 60 50 40 30 20 10 0 -20 -40 -60 -80 -100 -120 -140 -160
f1 (ppm)

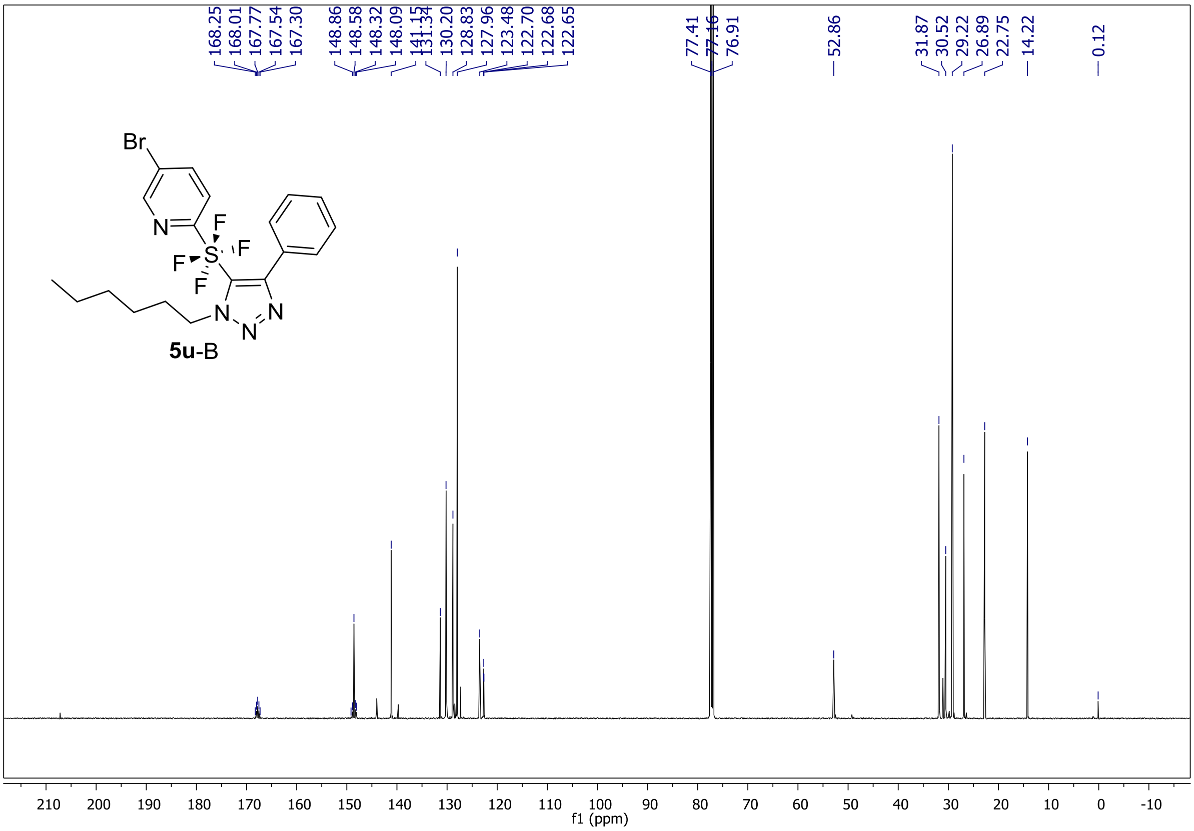
168.25
168.01
167.77
167.54
167.30
148.86
148.58
148.32
148.09
141.15
131.34
130.20
128.83
127.96
123.48
122.70
122.68
122.65
77.41
77.16
76.91
52.86
31.87
30.52
29.22
26.89
22.75
14.22
0.12
Br
N
F
F
F
S
F
N
N
N
5u-B
210 200 190 180 170 160 150 140 130 120 110 100 90 80 70 60 50 40 30 20 10 0 -10
f1 (ppm)

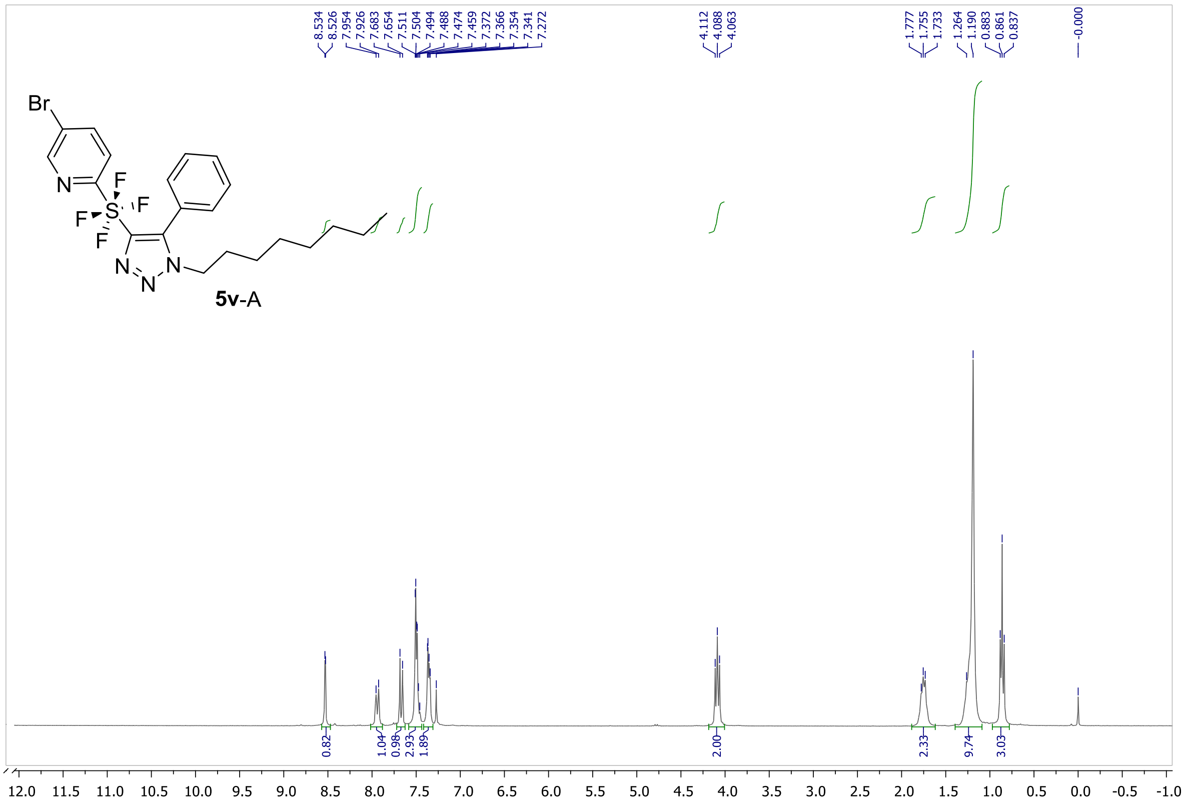
8.534
8.526
7.954
7.926
7.683
7.654
7.511
7.504
7.494
7.488
7.474
7.459
7.372
7.366
7.354
7.341
7.272
4.112
4.088
4.063
1.777
1.755
1.733
1.264
1.190
0.883
0.861
0.837
-0.000
Br
N
F
F
F
S
F
N
N
N
5v-A
0.82
1.04
0.98
2.93
1.89
2.00
2.33
9.74
3.03
12.0 11.5 11.0 10.5 10.0 9.5 9.0 8.5 8.0 7.5 7.0 6.5 6.0 5.5 5.0 4.5 4.0 3.5 3.0 2.5 2.0 1.5 1.0 0.5 0.0 -0.5 -1.0

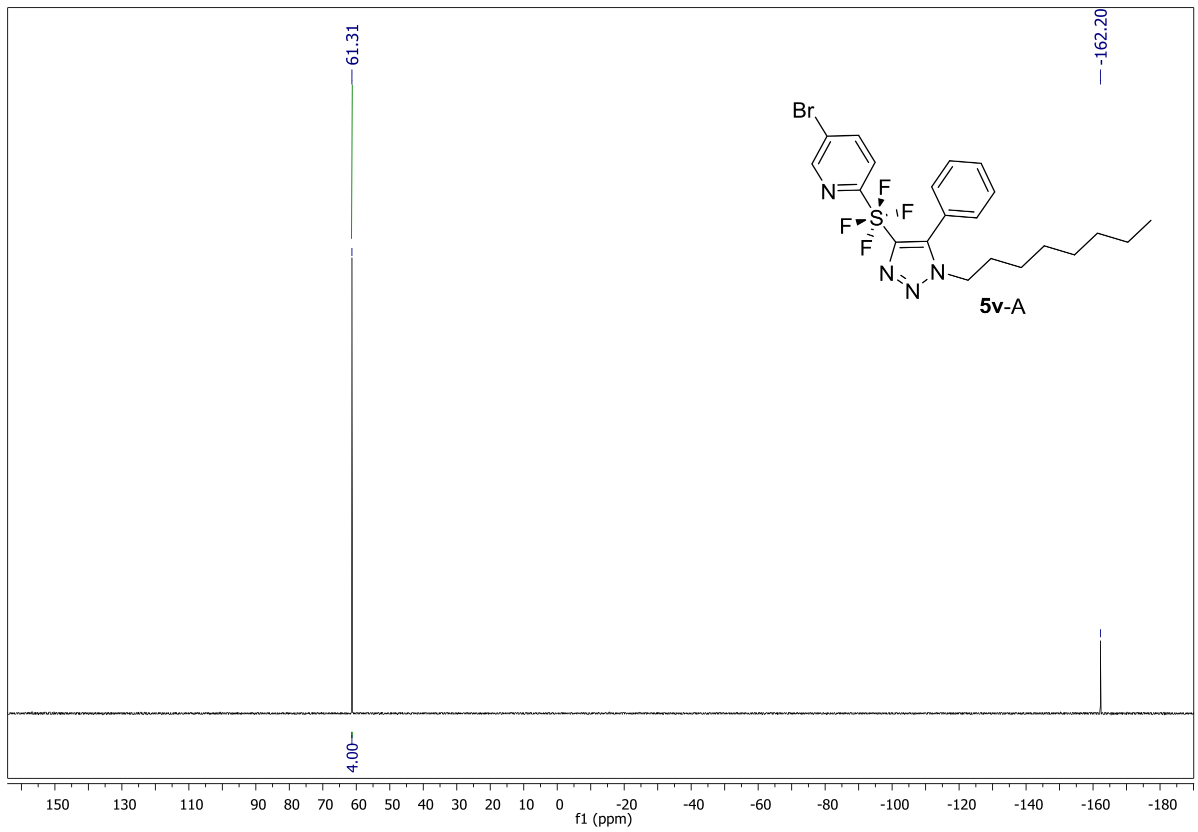
61.31
-162.20
Br
N
F
F
F
S
F
N
N
N
5v-A
4.00
150
130
110
90
80
70
60
50
40
30
20
10
0
-20
-40
-60
-80
-100
-120
-140
-160
-180
f1 (ppm)

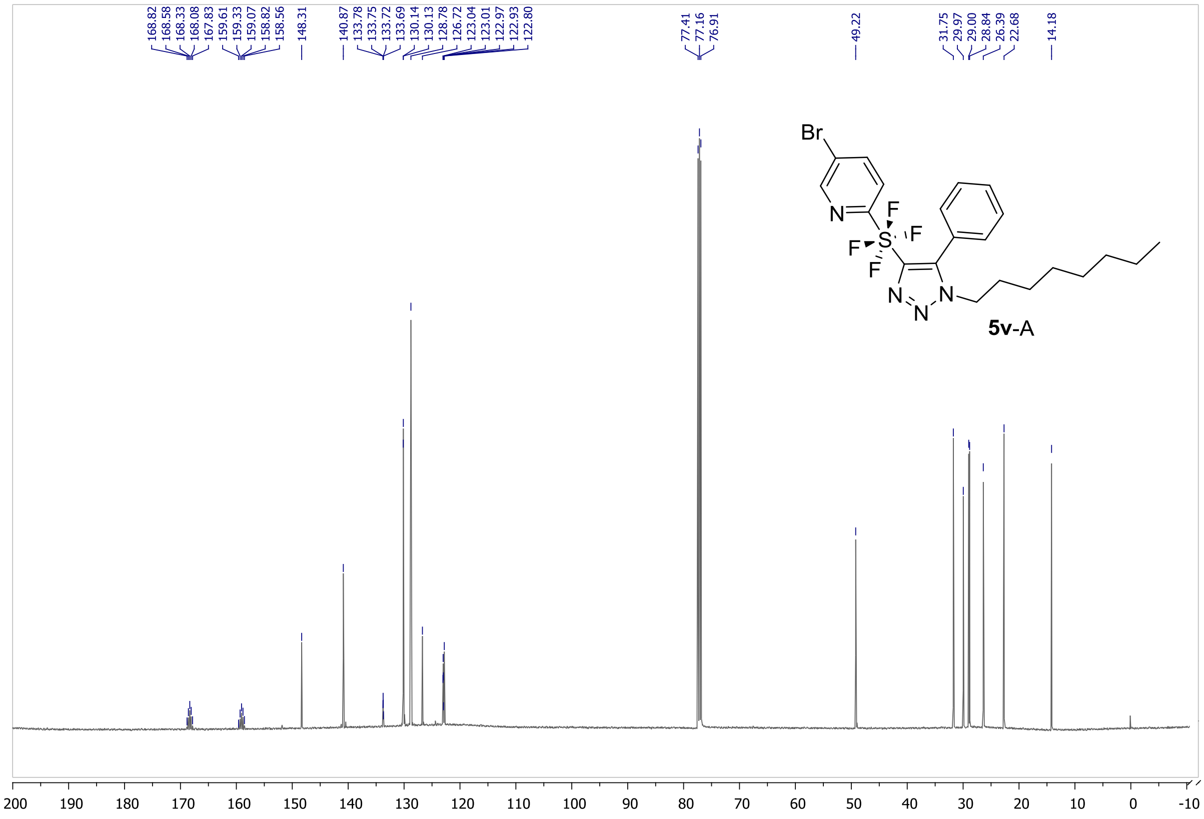
168.82
168.58
168.33
168.08
167.83
159.61
159.33
159.07
158.82
158.56
148.31
140.87
133.78
133.75
133.72
133.69
130.14
130.13
128.78
126.72
123.04
123.01
122.97
122.93
122.80
77.41
77.16
76.91
49.22
31.75
29.97
29.00
28.84
26.39
22.68
14.18
Br
N
F
F
F
S
F
N
N
N
5v-A
200
190
180
170
160
150
140
130
120
110
100
90
80
70
60
50
40
30
20
10
0
-10

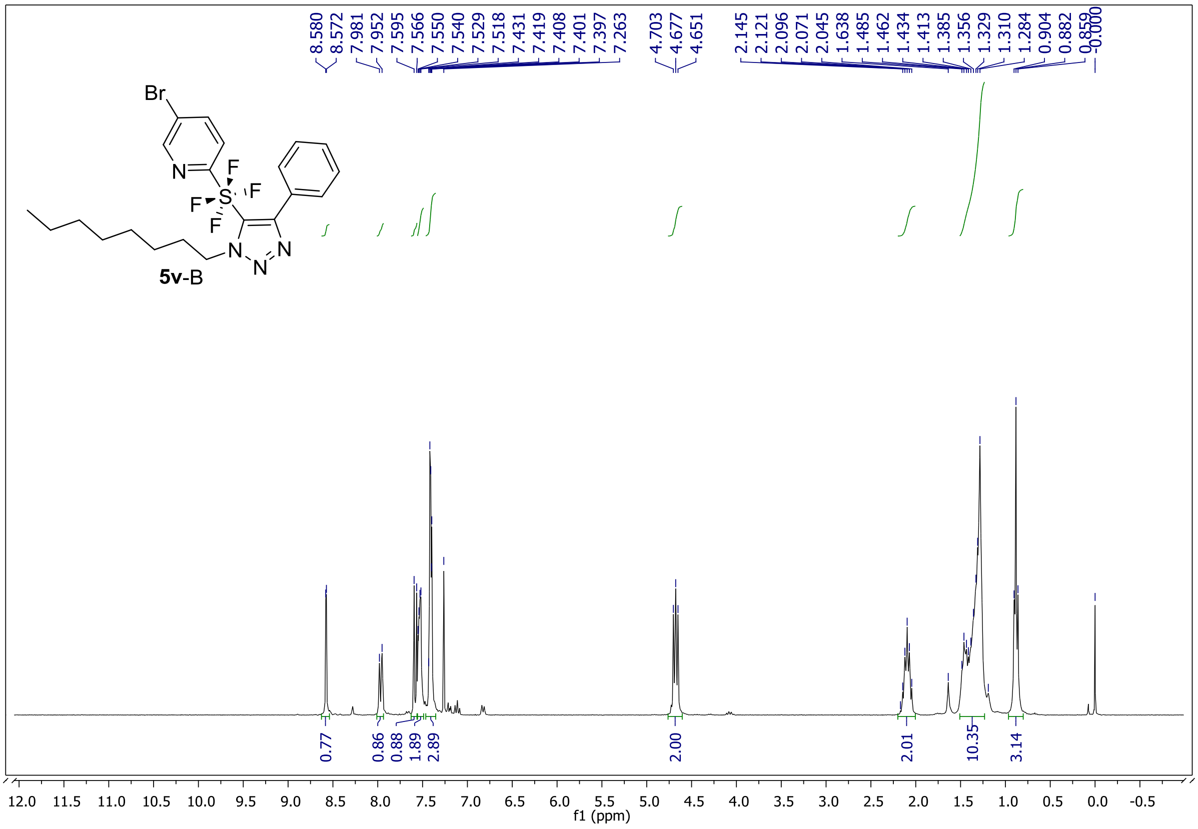
8.580
8.572
7.981
7.952
7.595
7.566
7.550
7.540
7.529
7.518
7.431
7.419
7.408
7.401
7.397
7.263
4.703
4.677
4.651
2.145
2.121
2.096
2.071
2.045
1.638
1.485
1.462
1.434
1.413
1.385
1.356
1.329
1.310
1.284
0.904
0.882
0.859
0.000
Br
F
F
F
F
S
N
N
N
N
5v-B
0.77
0.86
0.88
1.89
2.89
2.00
2.01
10.35
3.14
12.0 11.5 11.0 10.5 10.0 9.5 9.0 8.5 8.0 7.5 7.0 6.5 6.0 5.5 5.0 4.5 4.0 3.5 3.0 2.5 2.0 1.5 1.0 0.5 0.0 -0.5
f1 (ppm)

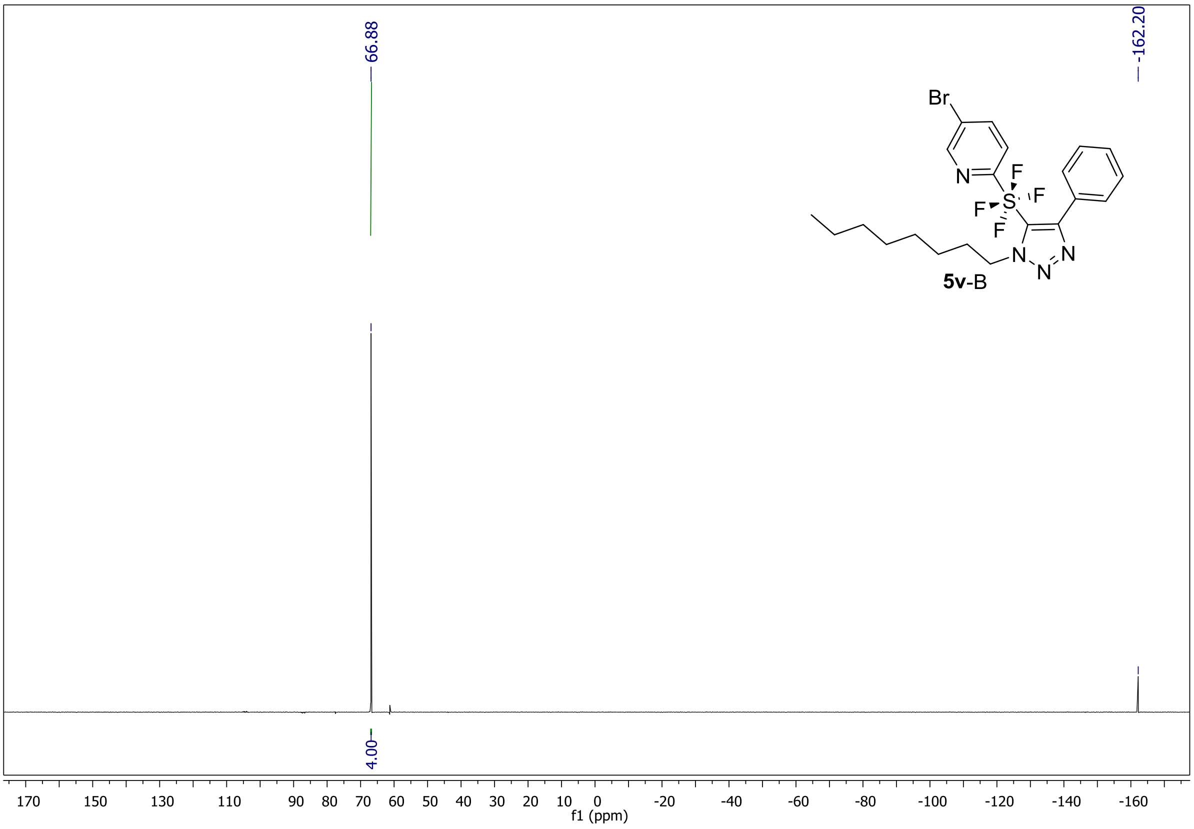
66.88
-162.20
Br
N
F
S
F
F
F
N
N
N
5v-B
4.00
170 150 130 110 90 80 70 60 50 40 30 20 10 0 -20 -40 -60 -80 -100 -120 -140 -160
f1 (ppm)

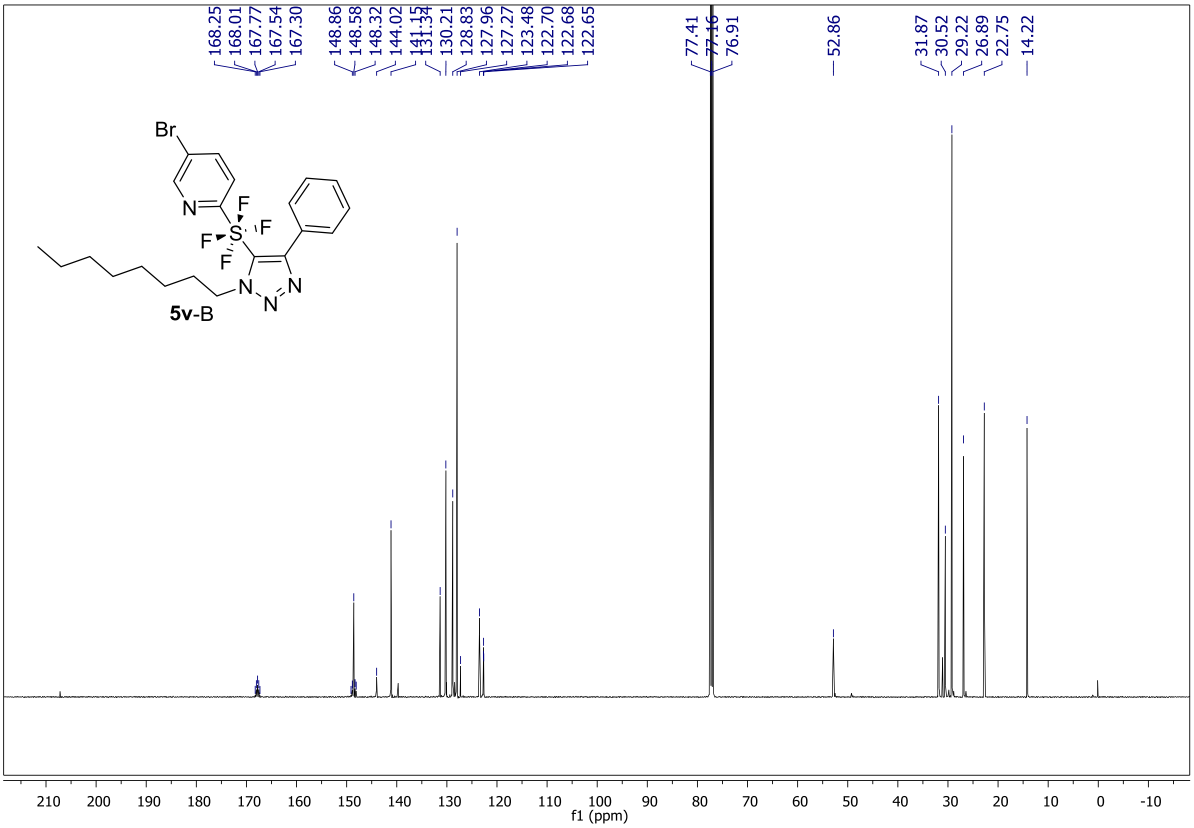
5v-B
168.25
168.01
167.77
167.54
167.30
148.86
148.58
148.32
144.02
130.21
128.83
127.96
127.27
123.48
122.70
122.68
122.65
77.41
76.91
52.86
31.87
30.52
29.22
26.89
22.75
14.22
f1 (ppm)

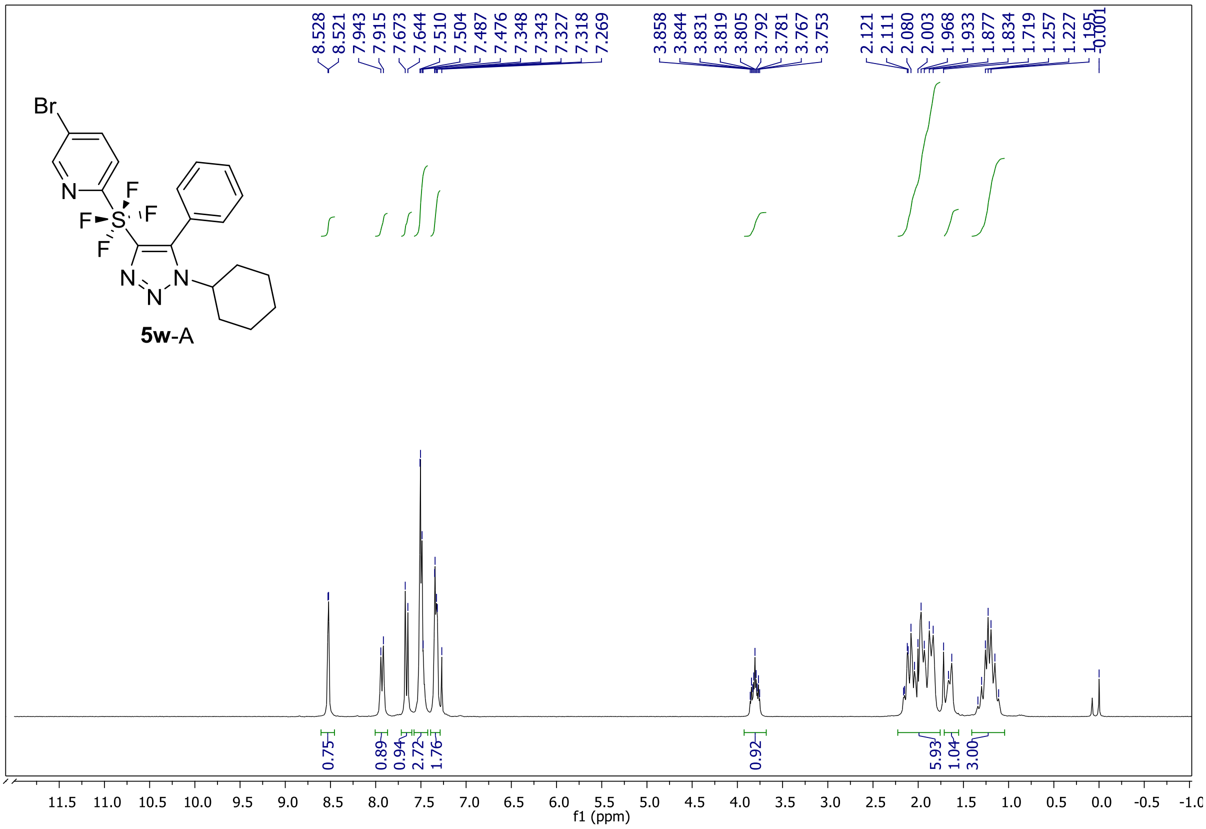
5w-A
8.528
8.521
7.943
7.915
7.673
7.644
7.510
7.504
7.487
7.476
7.348
7.343
7.327
7.318
7.269
3.858
3.844
3.831
3.819
3.805
3.792
3.781
3.767
3.753
2.121
2.111
2.080
2.003
1.968
1.933
1.877
1.834
1.719
1.257
1.227
0.75
0.89
0.94
2.72
1.76
0.92
5.93
1.04
3.00
f1 (ppm)

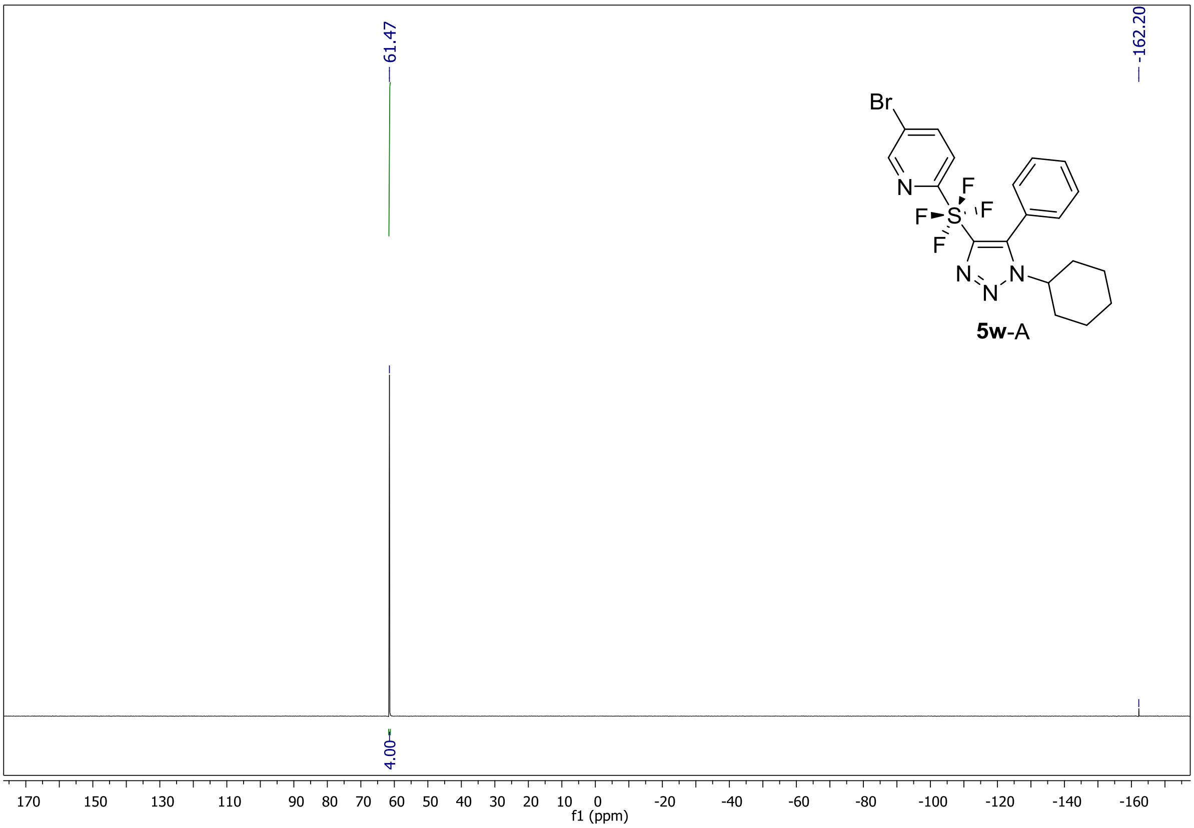

-61.47
-162.20
Br
N
F
F
F
S
F
N
N
N
5w-A
4.00
170 150 130 110 90 80 70 60 50 40 30 20 10 0 -20 -40 -60 -80 -100 -120 -140 -160
f1 (ppm)

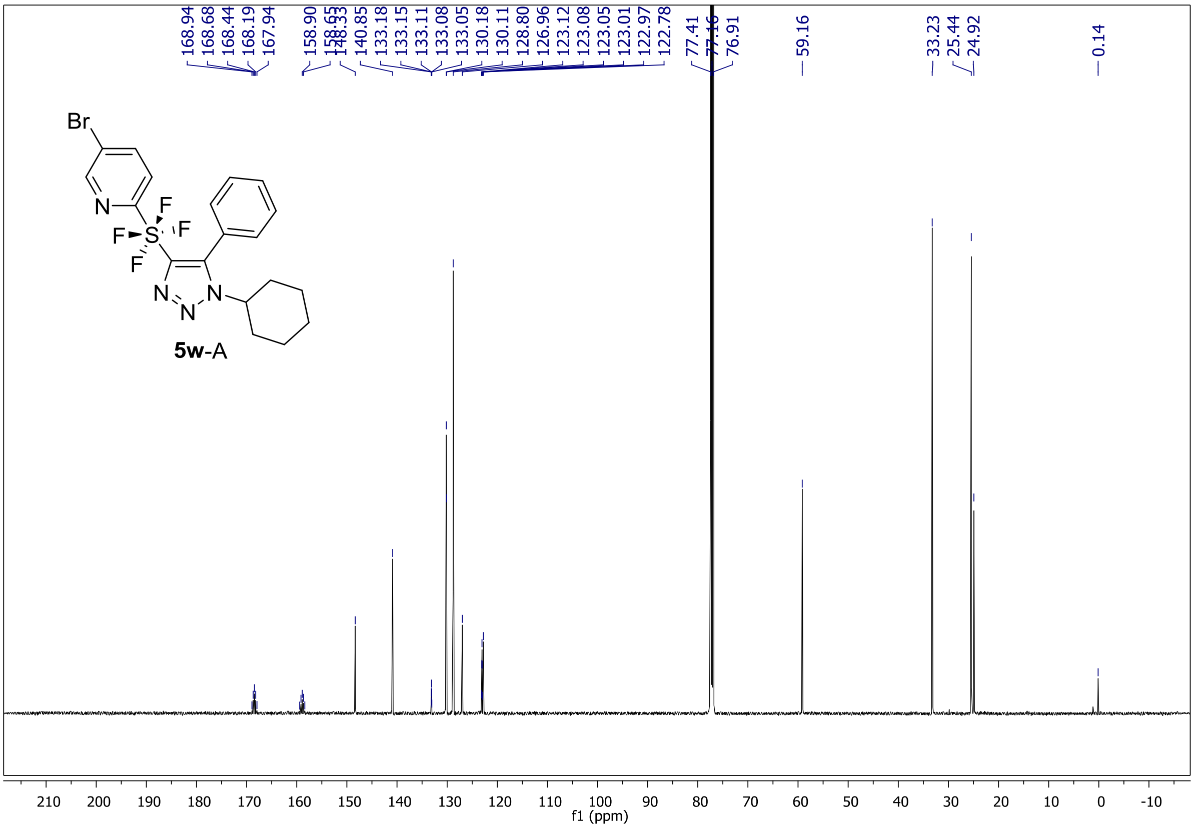

168.94
168.68
168.44
168.19
167.94
158.90
158.65
148.33
140.85
133.18
133.15
133.11
133.08
133.05
130.18
130.11
128.80
126.96
123.12
123.08
123.05
123.01
122.97
122.78
77.41
77.16
76.91
59.16
33.23
25.44
24.92
0.14
Br
N
F
F
F
S
F
N
N
N
5w-A
210 200 190 180 170 160 150 140 130 120 110 100 90 80 70 60 50 40 30 20 10 0 -10
f1 (ppm)

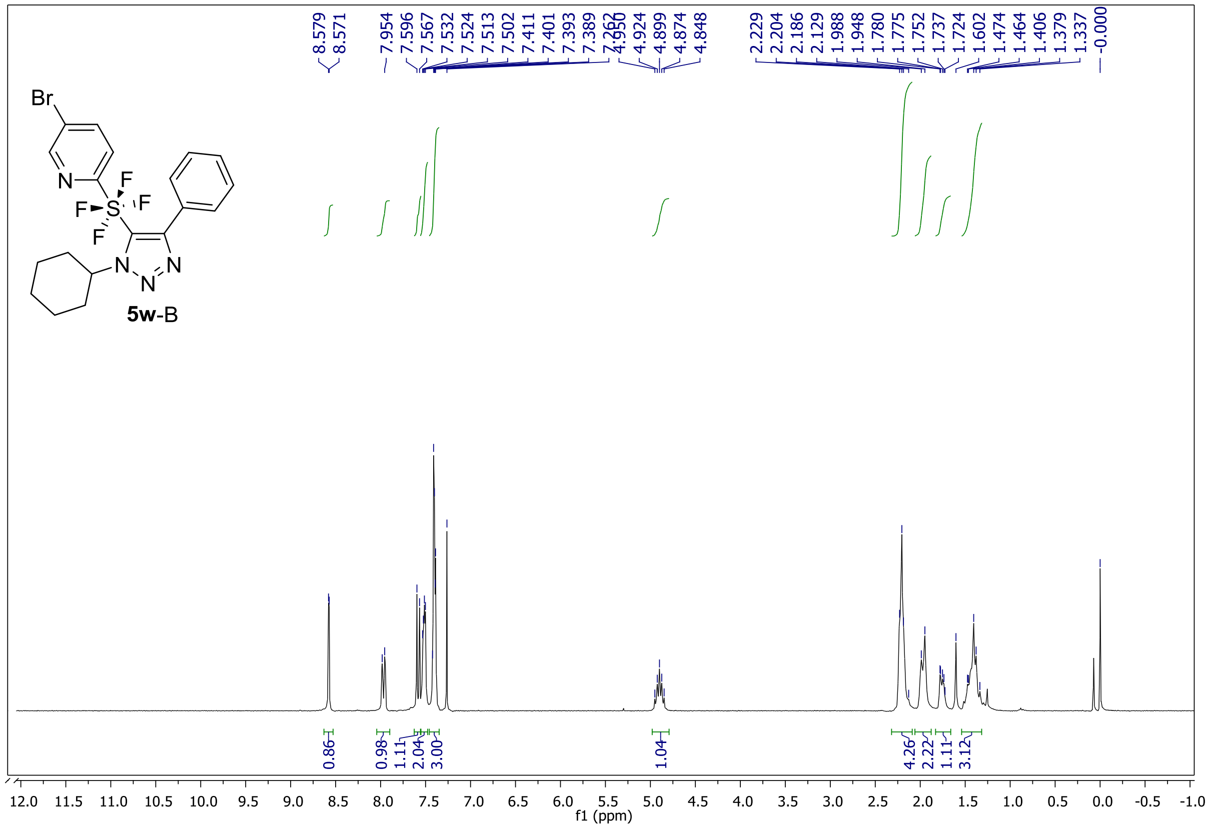

8.579
8.571
7.954
7.596
7.567
7.532
7.524
7.513
7.502
7.411
7.401
7.393
7.389
7.262
4.950
4.924
4.899
4.874
4.848
2.229
2.204
2.186
2.129
1.988
1.948
1.780
1.775
1.752
1.737
1.724
1.602
1.474
1.464
1.406
1.379
1.337
-0.000
Br
N
F
F
F
F
S
N
N
N
5w-B
0.86
0.98
1.11
2.04
3.00
1.04
4.26
2.22
1.11
3.12
f1 (ppm)

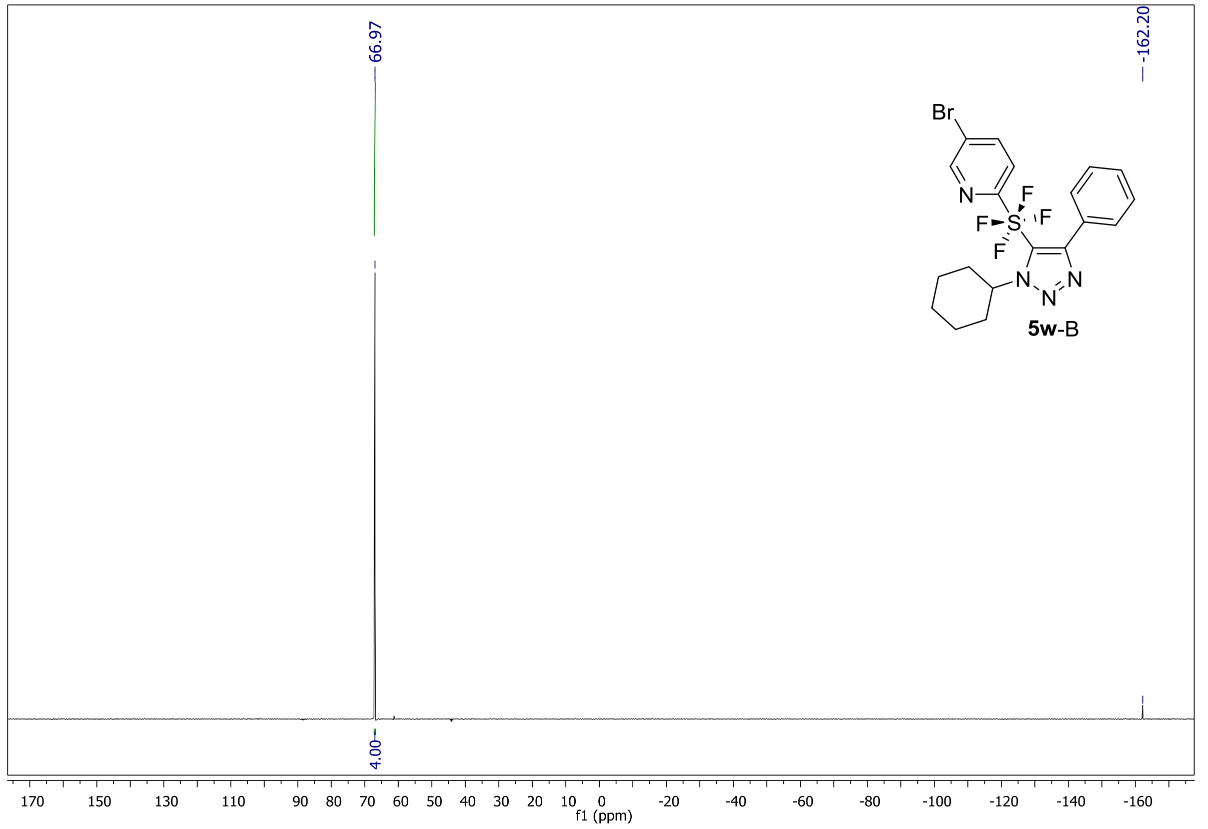

66.97
-162.20
Br
N
F
F
F
F
S
N
N
N
5w-B
4.00
f1 (ppm)

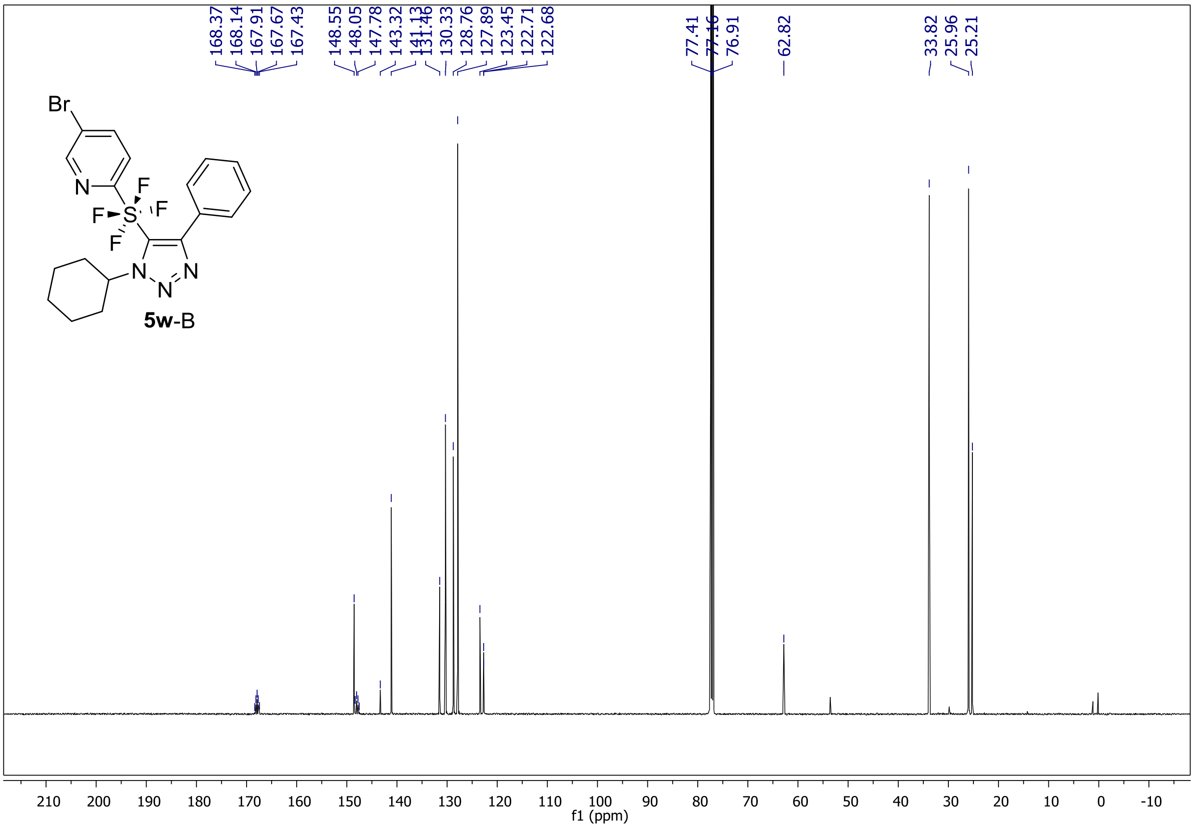
168.37
168.14
167.91
167.67
167.43
148.55
148.05
147.78
143.32
131.13
130.33
128.76
127.89
123.45
122.71
122.68
77.41
77.16
76.91
62.82
33.82
25.96
25.21
Br
N
F
F
F
F
S
N
N
N
5w-B
f1 (ppm)

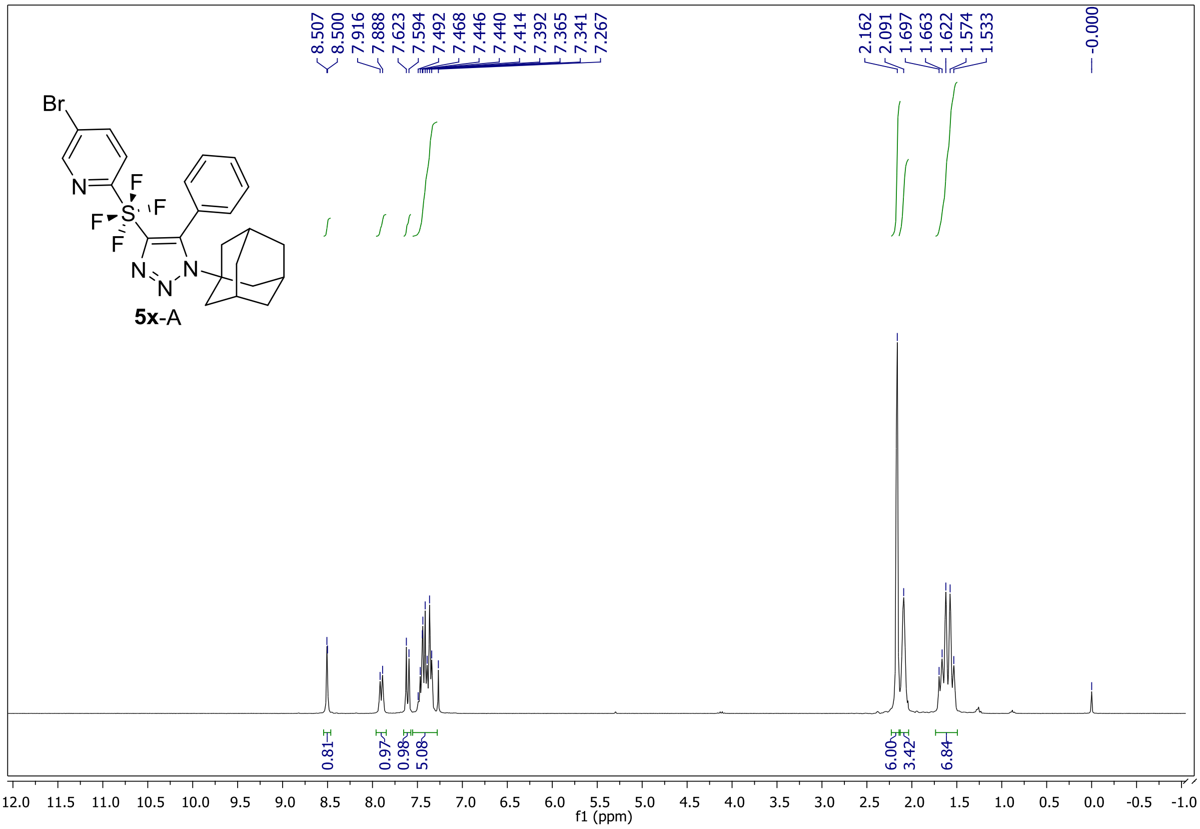
8.507
8.500
7.916
7.888
7.623
7.594
7.492
7.468
7.446
7.440
7.414
7.392
7.365
7.341
7.267
2.162
2.091
1.697
1.663
1.622
1.574
1.533
-0.000
Br
N
F
F
F
F
S
N
N
N
5x-A
0.81
0.97
0.98
5.08
6.00
3.42
6.84
f1 (ppm)

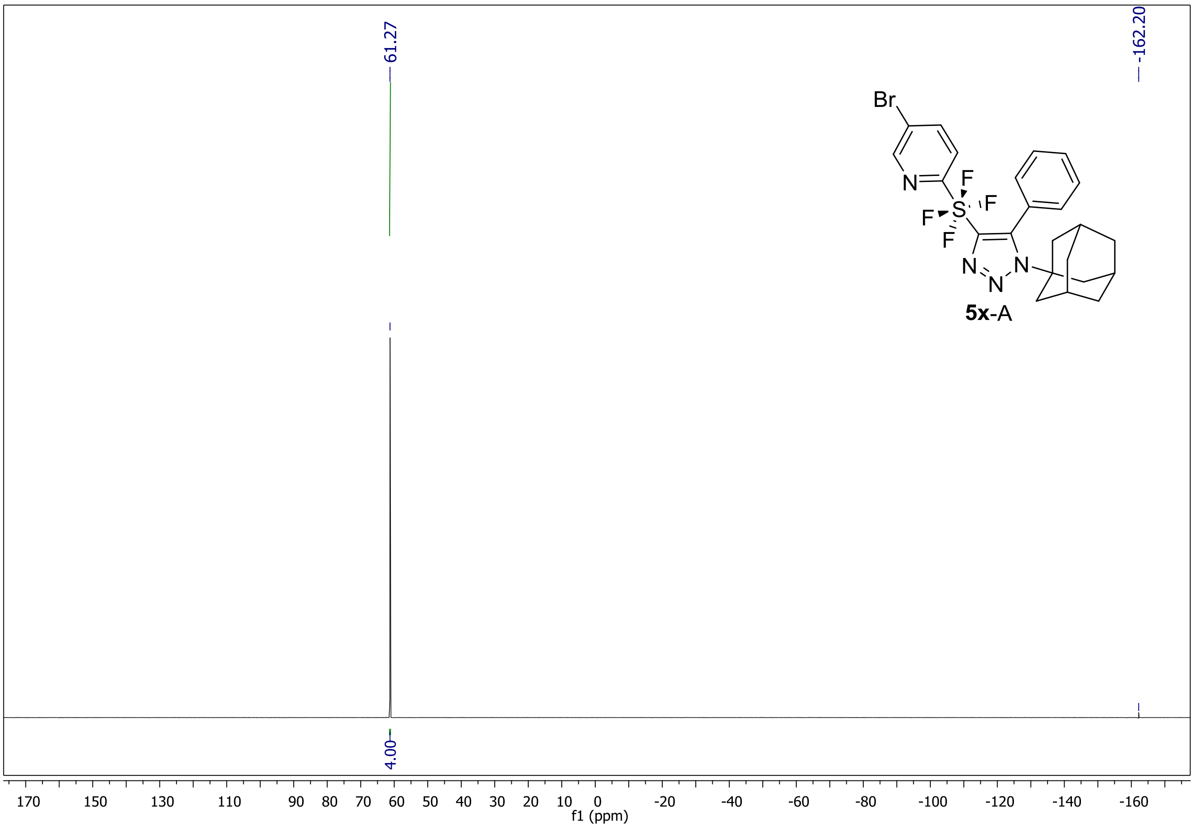
-61.27
-162.20
Br
N
F
S
F
F
F
N
N
N
5x-A
4.00
170 150 130 110 90 80 70 60 50 40 30 20 10 0 -20 -40 -60 -80 -100 -120 -140 -160
f1 (ppm)

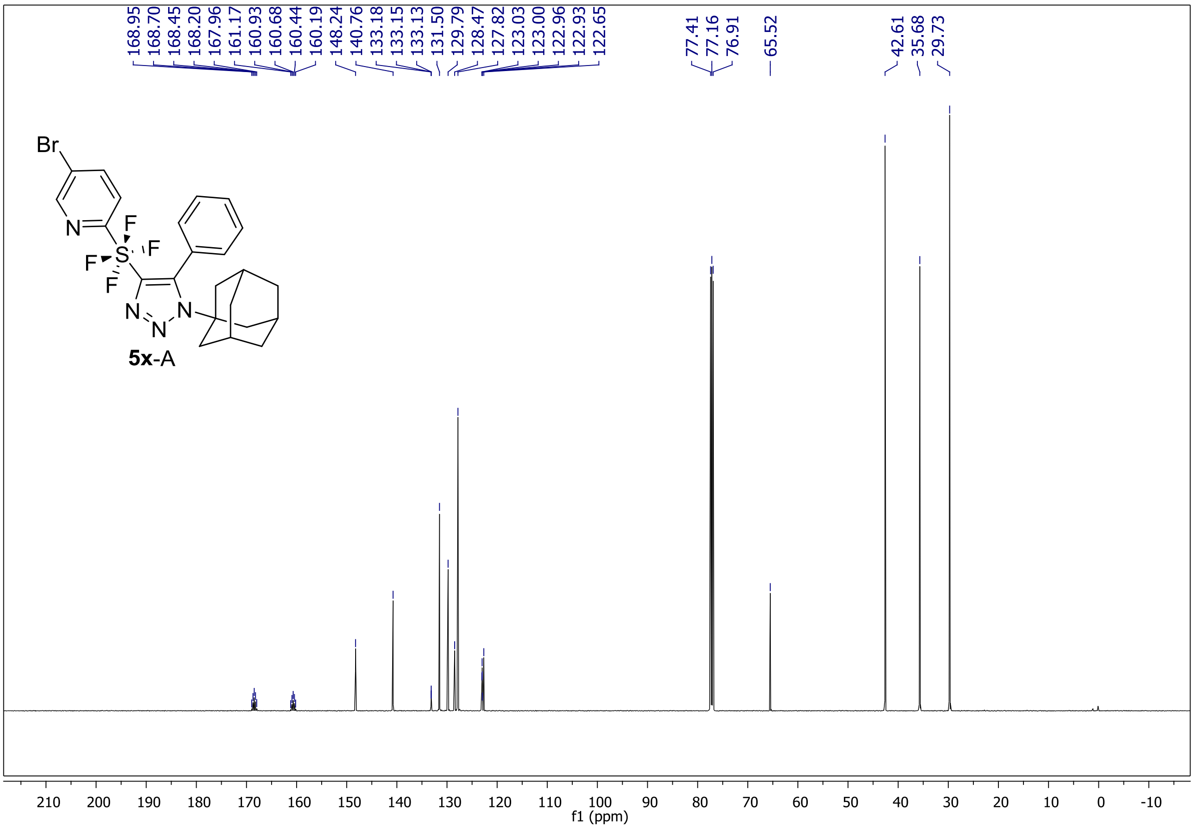
168.95
168.70
168.45
168.20
167.96
161.17
160.93
160.68
160.44
160.19
148.24
140.76
133.18
133.15
133.13
131.50
129.79
128.47
127.82
123.03
123.00
122.96
122.93
122.65
77.41
77.16
76.91
65.52
42.61
35.68
29.73
Br
N
F
S
F
F
F
N
N
N
5x-A
210 200 190 180 170 160 150 140 130 120 110 100 90 80 70 60 50 40 30 20 10 0 -10
f1 (ppm)

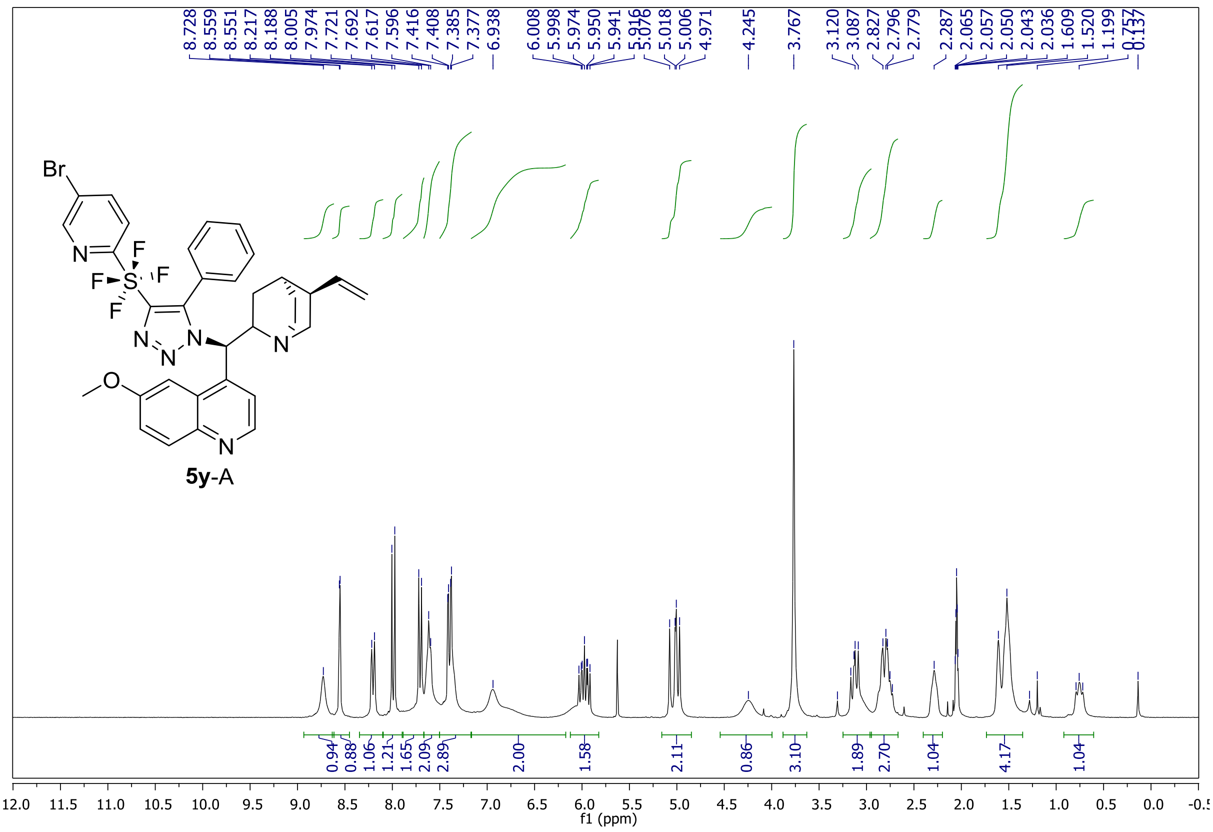

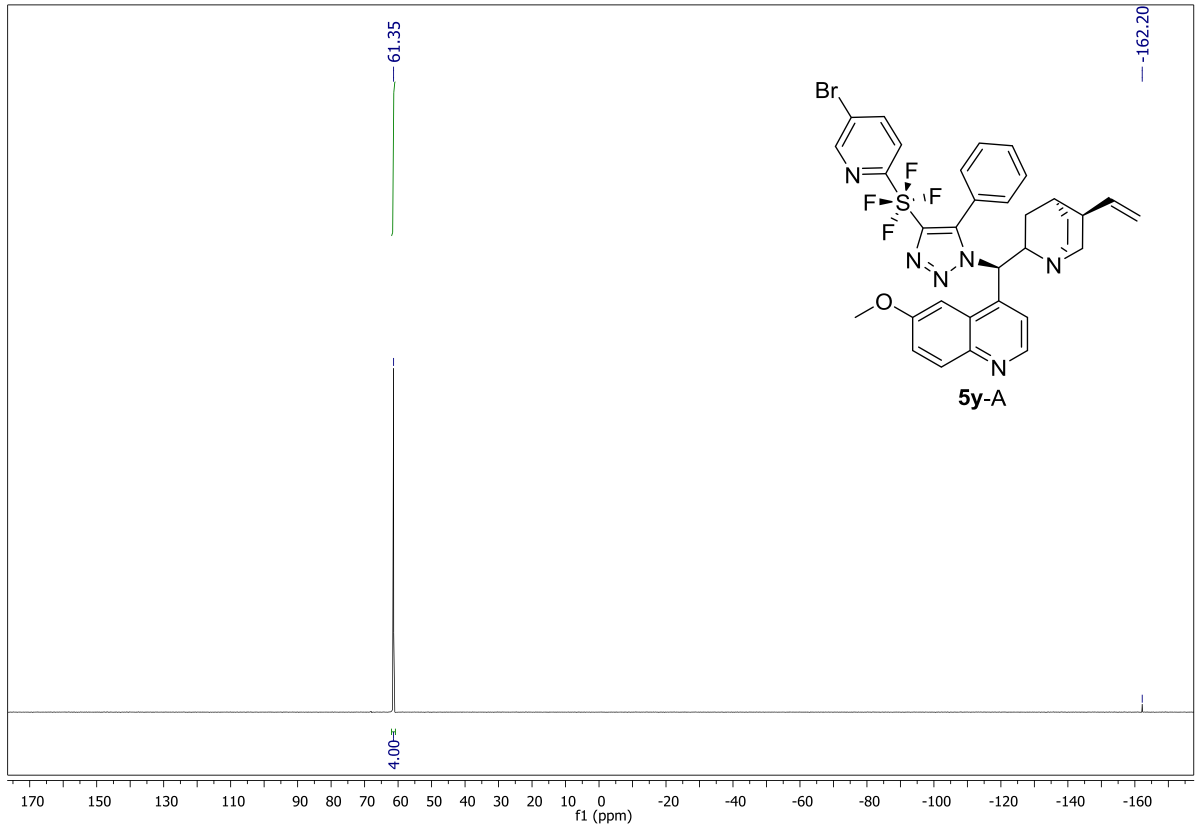

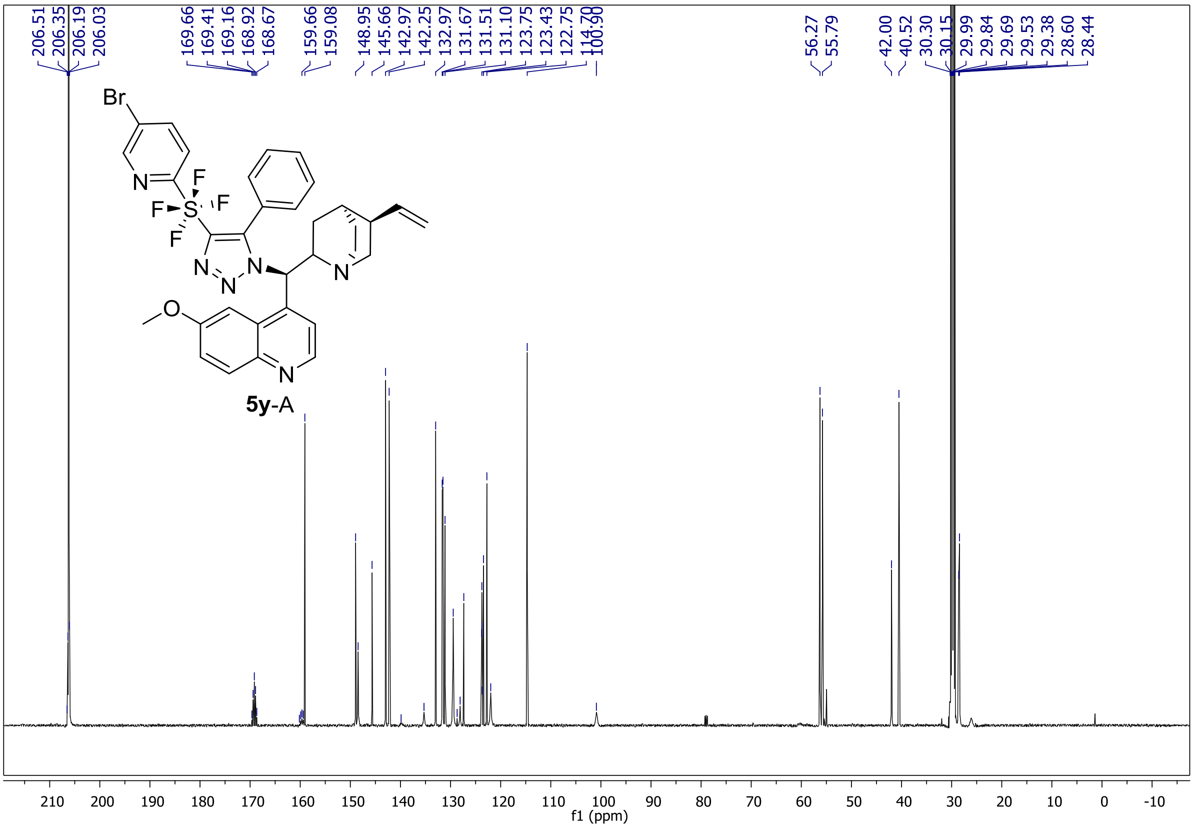

5y-A
f1 (ppm)

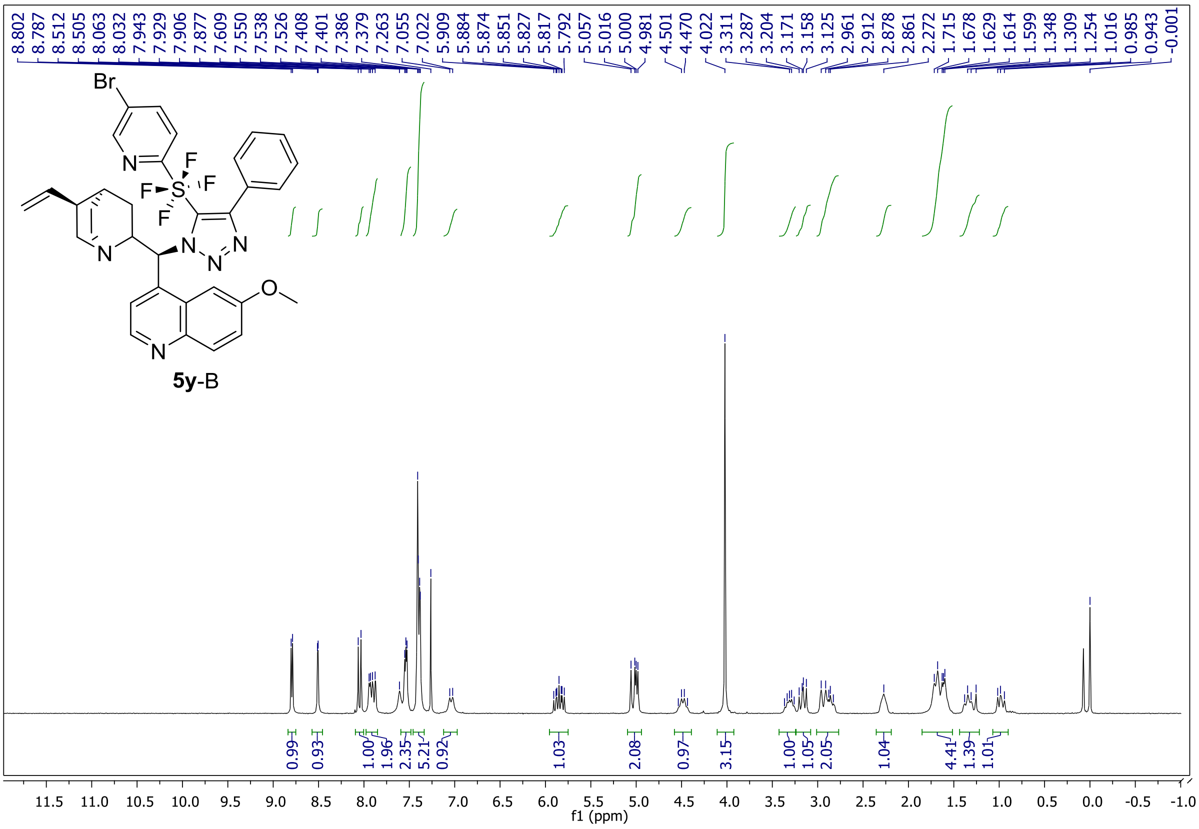

5y-B
f1 (ppm)

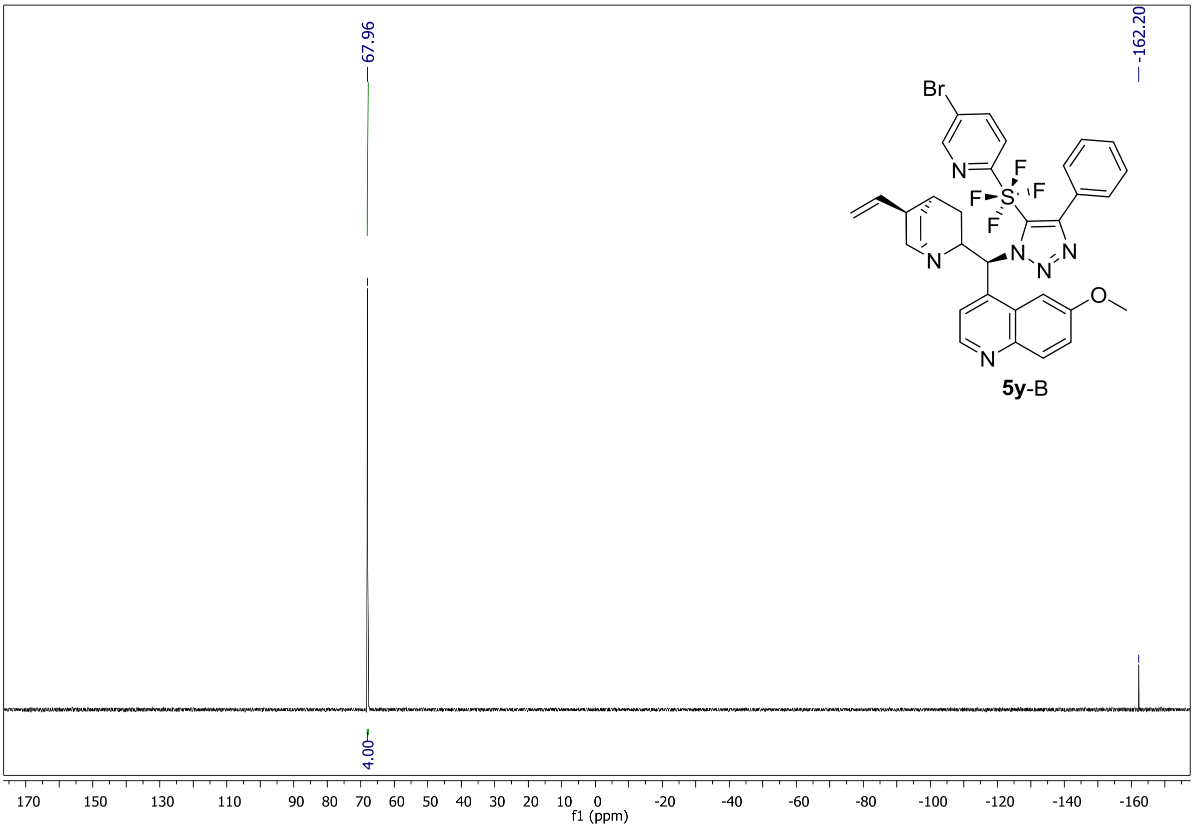
67.96
-162.20
4.00
5y-B
Br
N
F
S
N
O
170 150 130 110 90 80 70 60 50 40 30 20 10 0 -20 -40 -60 -80 -100 -120 -140 -160
f1 (ppm)

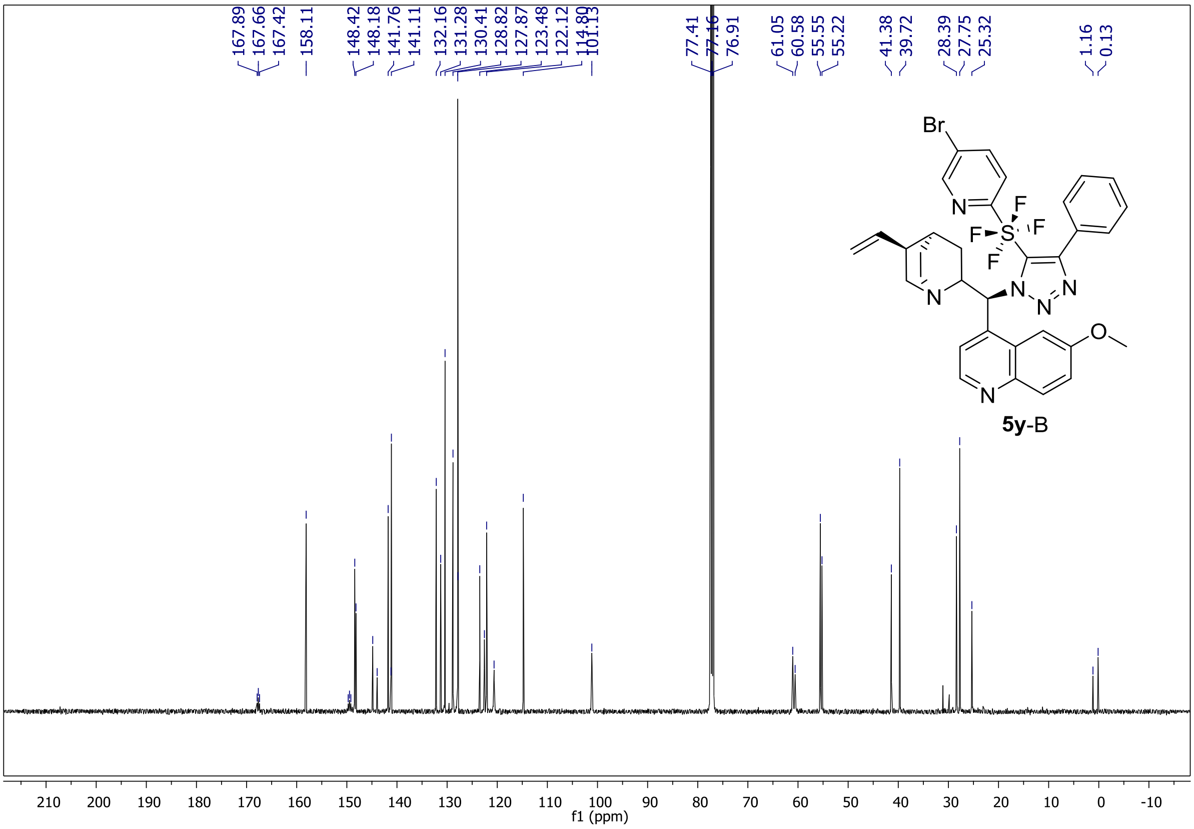
167.89
167.66
167.42
158.11
148.42
148.18
141.76
141.11
132.16
131.28
130.41
128.82
127.87
123.48
122.12
114.80
101.13
77.41
77.16
76.91
61.05
60.58
55.55
55.22
41.38
39.72
28.39
27.75
25.32
1.16
0.13
5y-B
210 200 190 180 170 160 150 140 130 120 110 100 90 80 70 60 50 40 30 20 10 0 -10
f1 (ppm)

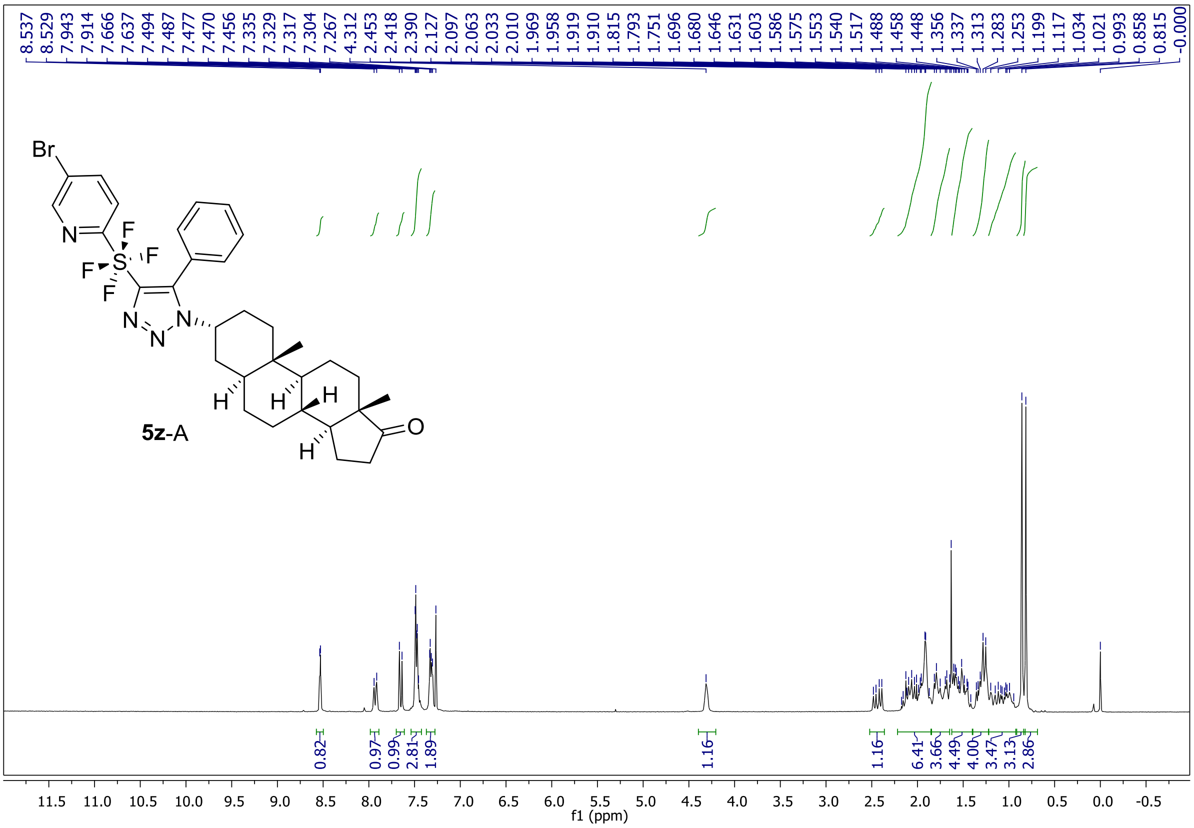
8.537
8.529
7.943
7.914
7.666
7.637
7.494
7.487
7.477
7.470
7.456
7.335
7.329
7.317
7.304
7.267
4.312
2.453
2.418
2.390
2.127
2.097
2.063
2.033
2.010
1.969
1.958
1.919
1.910
1.815
1.793
1.751
1.696
1.680
1.646
1.631
1.603
1.586
1.575
1.553
1.540
1.517
1.488
1.458
1.448
1.356
1.337
1.313
1.283
1.253
1.199
1.117
1.034
1.021
0.993
0.858
0.815
-0.000
Br
N
F
S
F
F
F
N
N
N
H
H
H
H
O
5z-A
0.82
0.97
0.99
2.81
1.89
1.16
1.16
6.41
3.66
4.49
4.00
3.47
3.13
2.86
f1 (ppm)

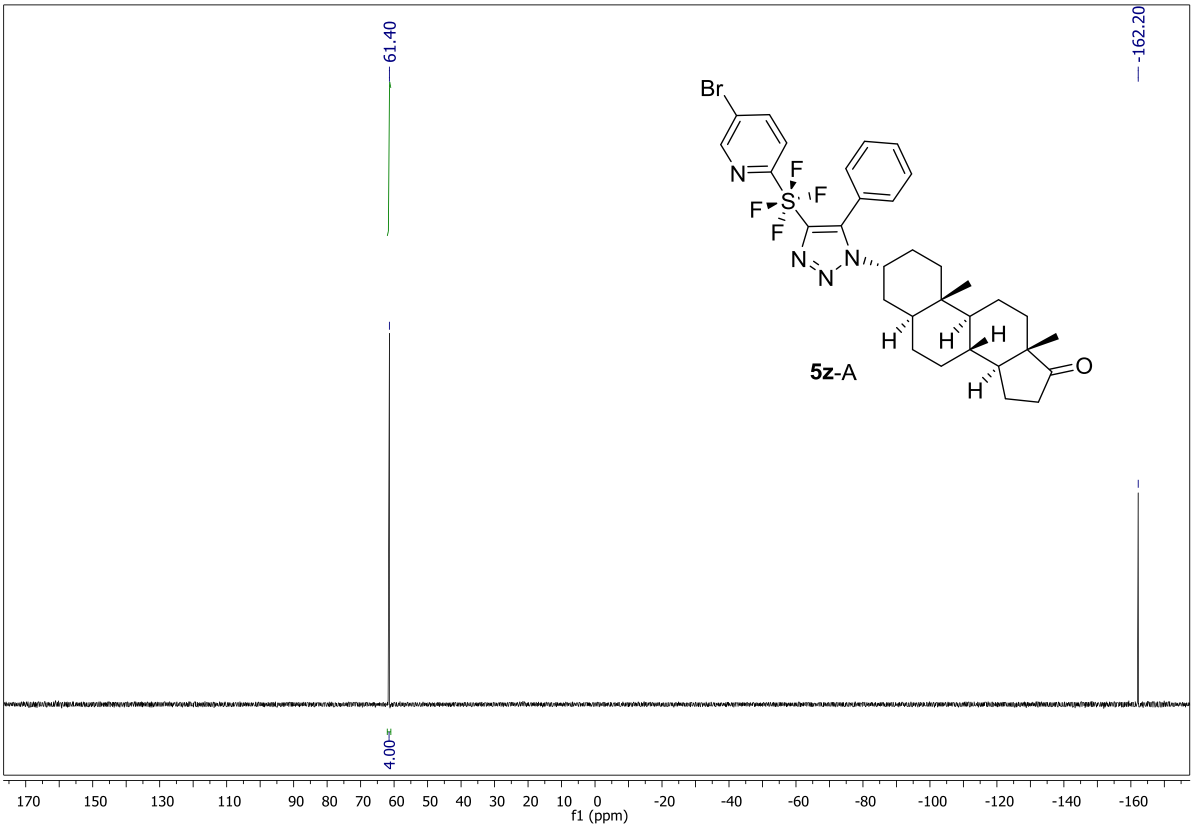
61.40
-162.20
Br
N
F
S
F
F
F
N
N
N
H
H
H
H
O
5z-A
4.00
f1 (ppm)

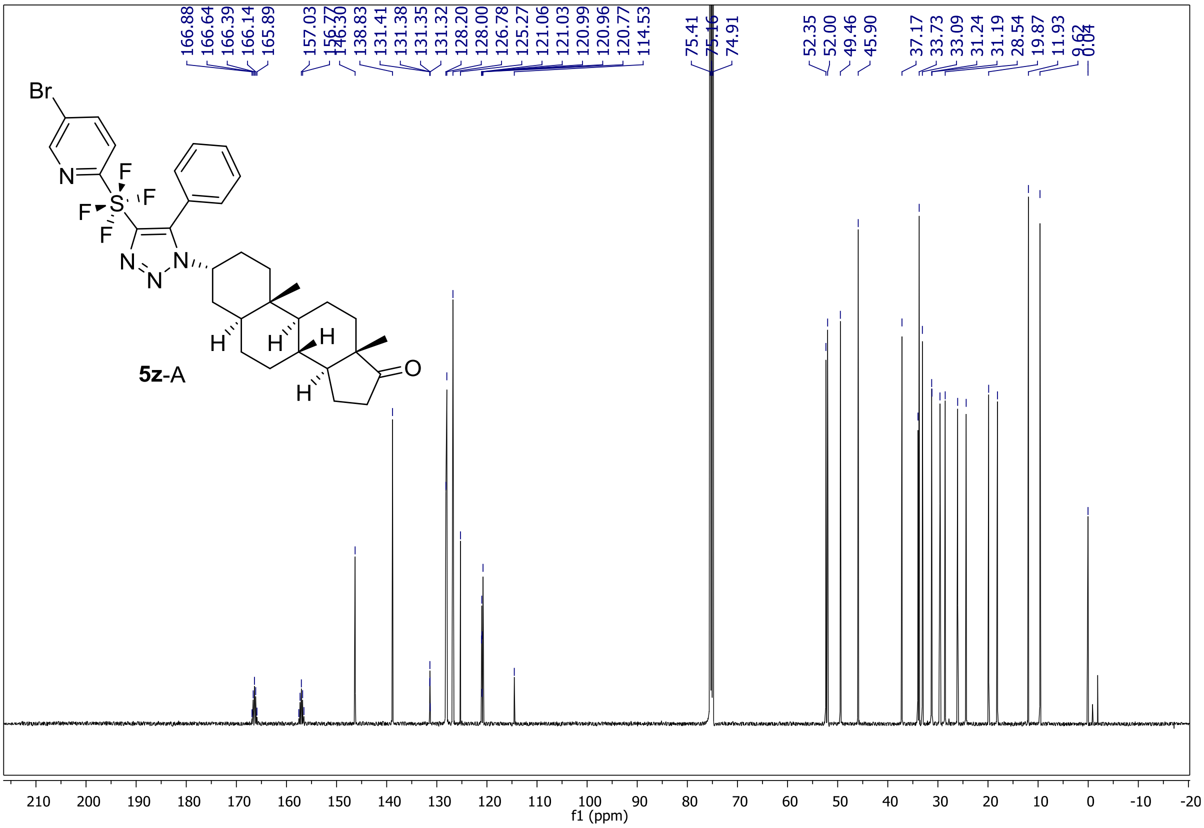
166.88
166.64
166.39
166.14
165.89
157.03
156.77
146.30
138.83
131.41
131.38
131.35
131.32
128.20
128.00
126.78
125.27
121.06
121.03
120.99
120.96
120.77
114.53
75.41
75.16
74.91
52.35
52.00
49.46
45.90
37.17
33.73
33.09
31.24
31.19
28.54
19.87
11.93
9.62
0.04
Br
N
F
S
F
F
F
N
N
N
H
H
H
H
O
5z-A
210 200 190 180 170 160 150 140 130 120 110 100 90 80 70 60 50 40 30 20 10 0 -10 -20
f1 (ppm)

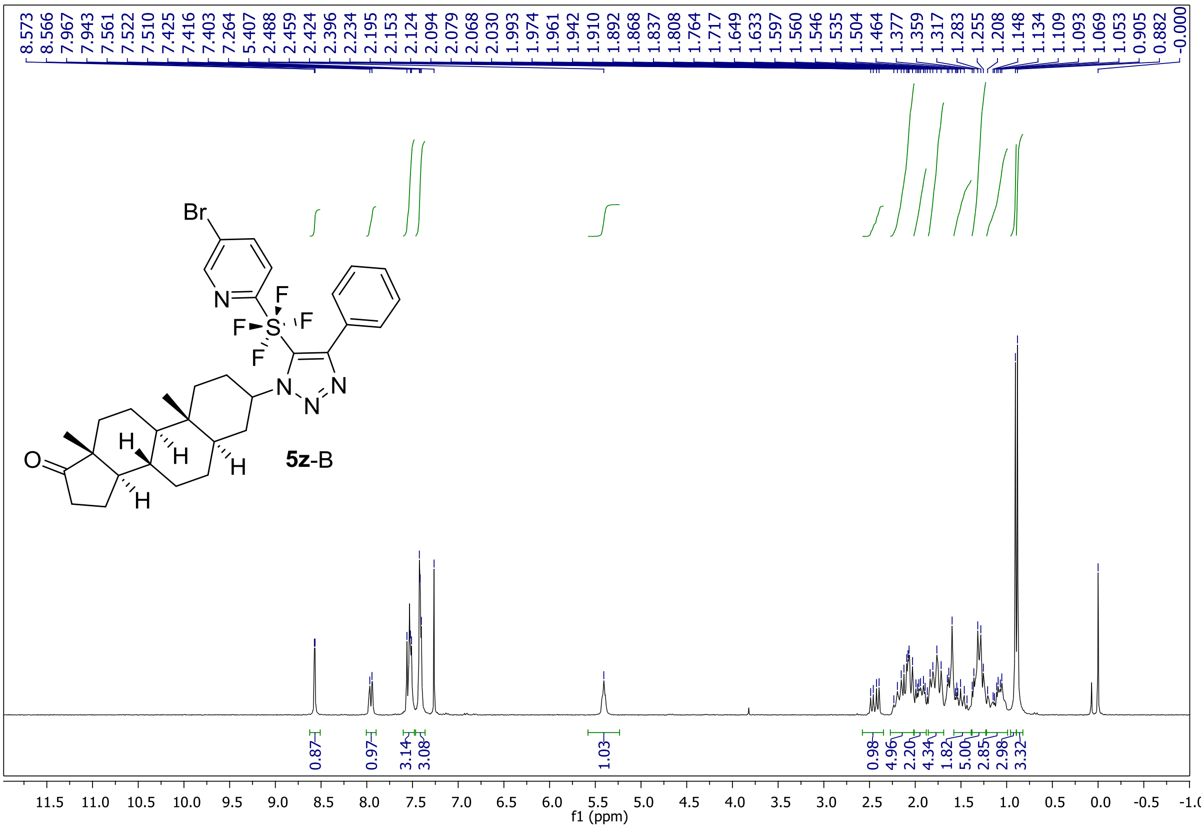
8.573
8.566
7.967
7.943
7.561
7.522
7.510
7.425
7.416
7.403
7.264
5.407
2.488
2.459
2.424
2.396
2.234
2.195
2.153
2.124
2.094
2.079
2.068
2.030
1.993
1.974
1.961
1.942
1.910
1.892
1.868
1.837
1.808
1.764
1.717
1.649
1.633
1.597
1.560
1.546
1.535
1.504
1.464
1.377
1.359
1.317
1.283
1.255
1.208
1.148
1.134
1.109
1.093
1.069
1.053
0.905
0.882
-0.000
Br
N
F
S
F
F
F
N
N
N
O
H
H
H
H
5z-B
0.87
0.97
3.14
3.08
1.03
0.98
4.96
2.20
4.34
1.82
5.00
2.85
2.98
3.32
11.5 11.0 10.5 10.0 9.5 9.0 8.5 8.0 7.5 7.0 6.5 6.0 5.5 5.0 4.5 4.0 3.5 3.0 2.5 2.0 1.5 1.0 0.5 0.0 -0.5 -1.0
f1 (ppm)

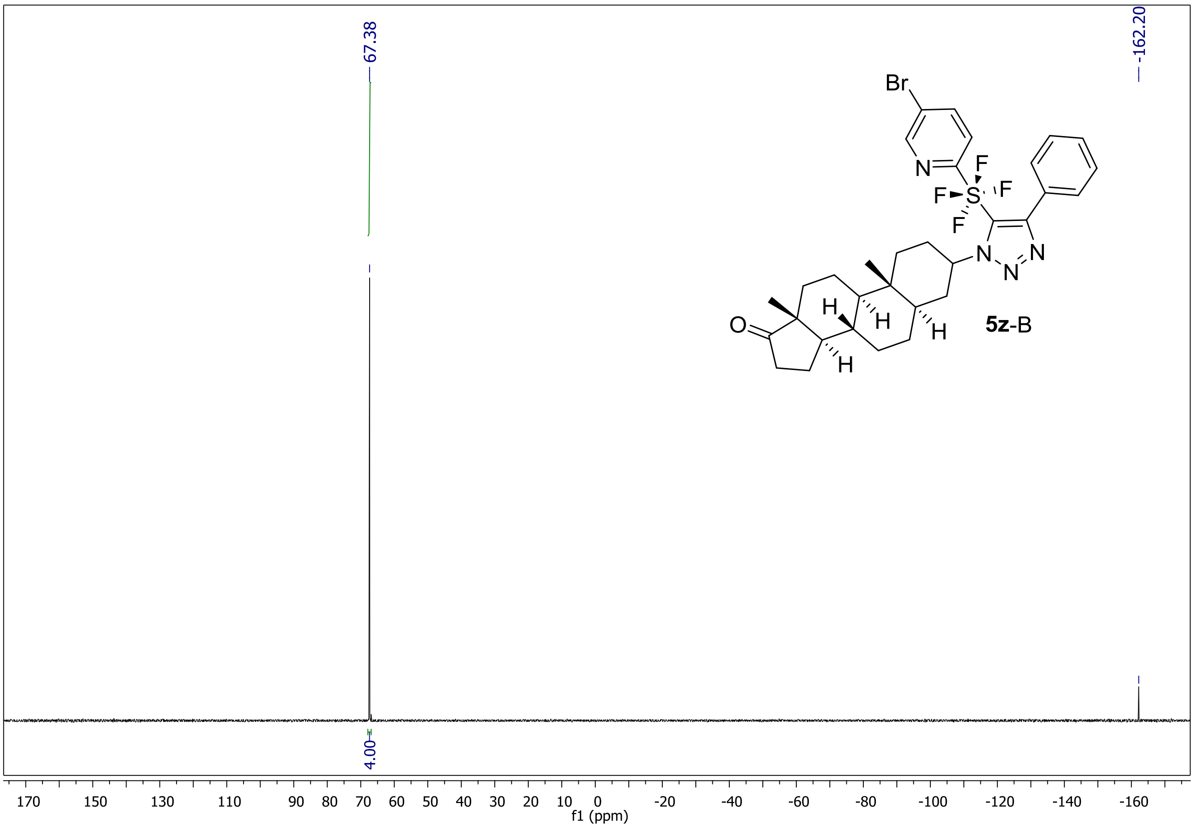

67.38
-162.20
Br
N
F
S
F
F
F
N
N
N
O
H
H
H
H
5z-B
4.00
170
150
130
110
90
80
70
60
50
40
30
20
10
0
-20
-40
-60
-80
-100
-120
-140
-160
f1 (ppm)

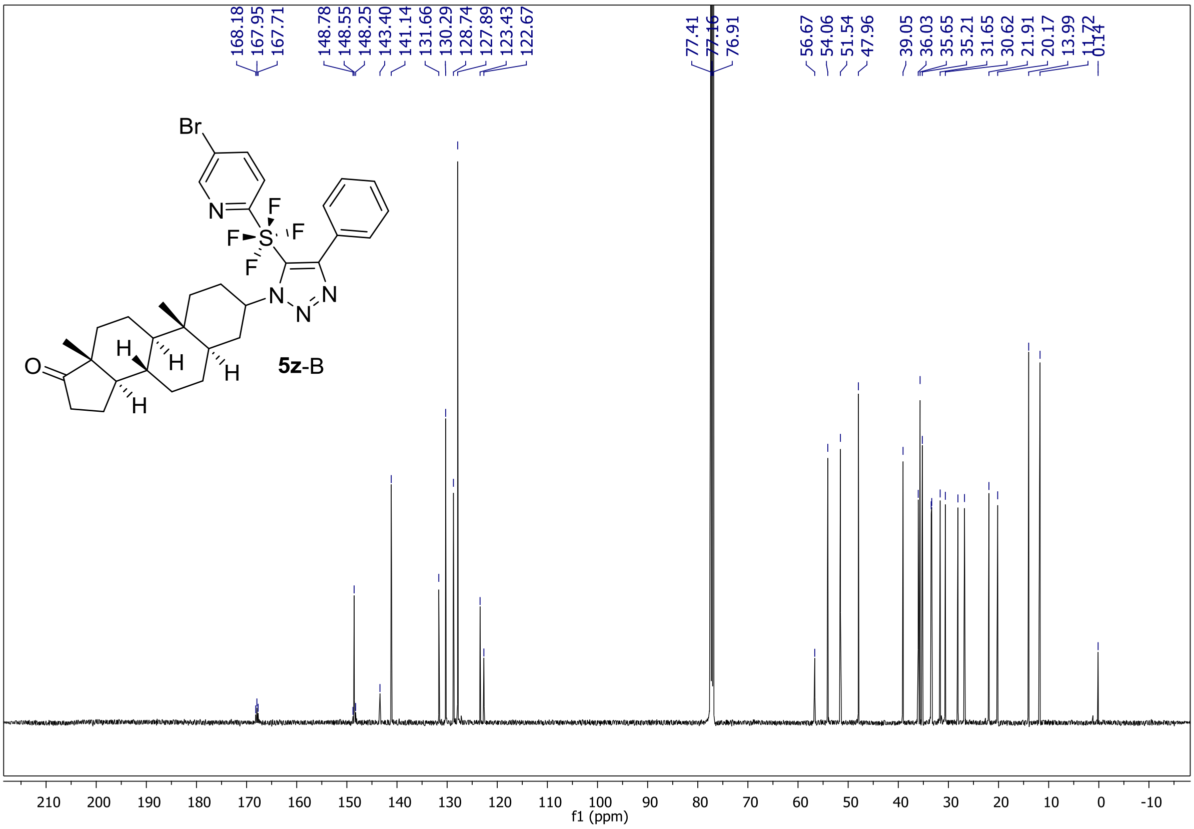

168.18
167.95
167.71
148.78
148.55
148.25
143.40
141.14
131.66
130.29
128.74
127.89
123.43
122.67
77.41
77.16
76.91
56.67
54.06
51.54
47.96
39.05
36.03
35.65
35.21
31.65
30.62
21.91
20.17
13.99
11.72
0.14
Br
N
F
S
F
F
F
N
N
N
O
H
H
H
H
5z-B
210
200
190
180
170
160
150
140
130
120
110
100
90
80
70
60
50
40
30
20
10
0
-10
f1 (ppm)

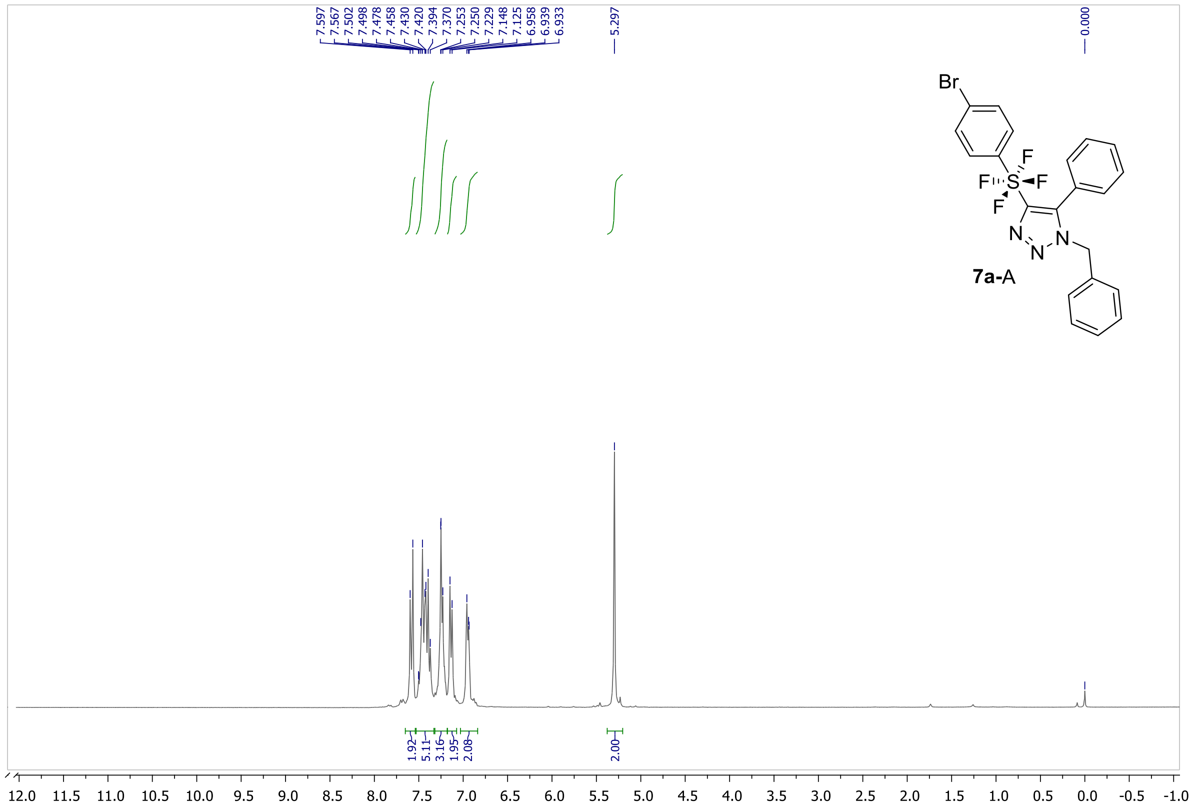
7.597
7.567
7.502
7.498
7.478
7.458
7.430
7.420
7.394
7.370
7.253
7.250
7.229
7.148
7.125
6.958
6.939
6.933
5.297
0.000
Br
F
F
F
F
S
N
N
N
7a-A
1.92
5.11
3.16
1.95
2.08
2.00
12.0 11.5 11.0 10.5 10.0 9.5 9.0 8.5 8.0 7.5 7.0 6.5 6.0 5.5 5.0 4.5 4.0 3.5 3.0 2.5 2.0 1.5 1.0 0.5 0.0 -0.5 -1.0

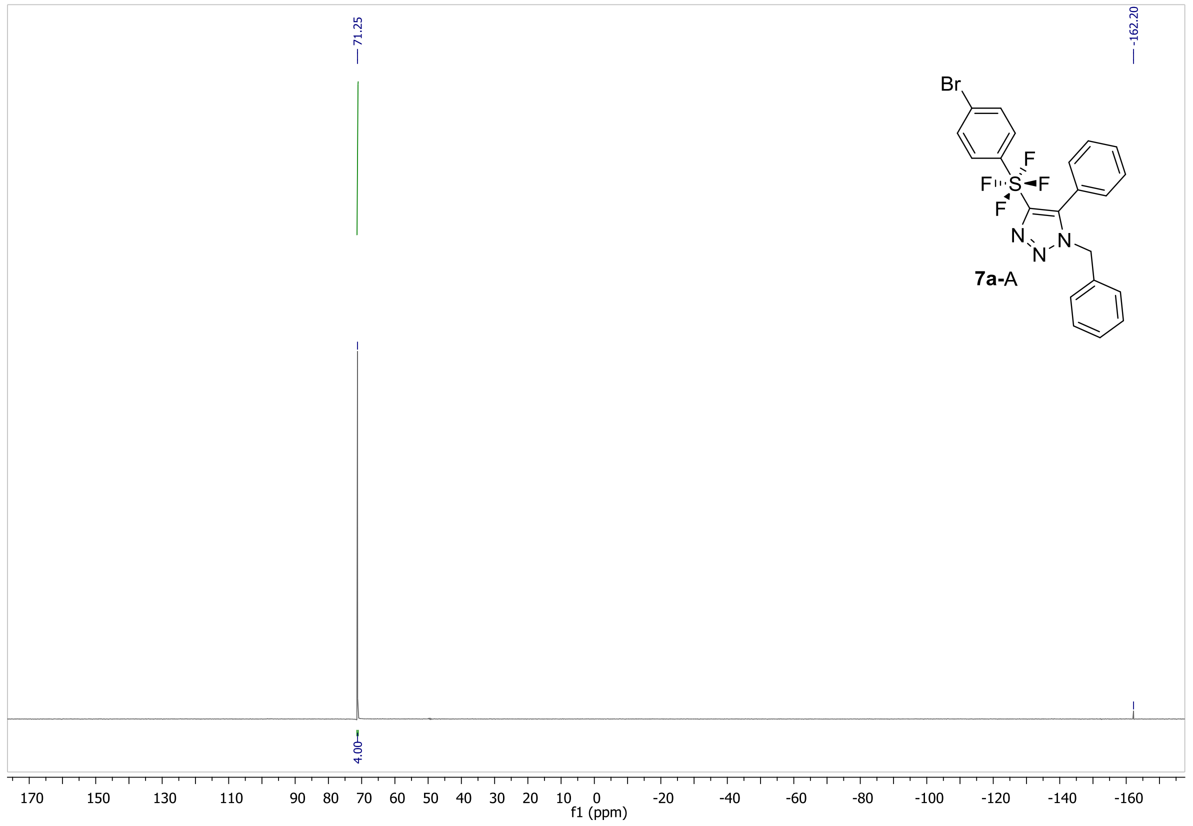
71.25
-162.20
Br
F
F
F
F
S
N
N
N
7a-A
4.00
170 150 130 110 90 80 70 60 50 40 30 20 10 0 -20 -40 -60 -80 -100 -120 -140 -160
f1 (ppm)

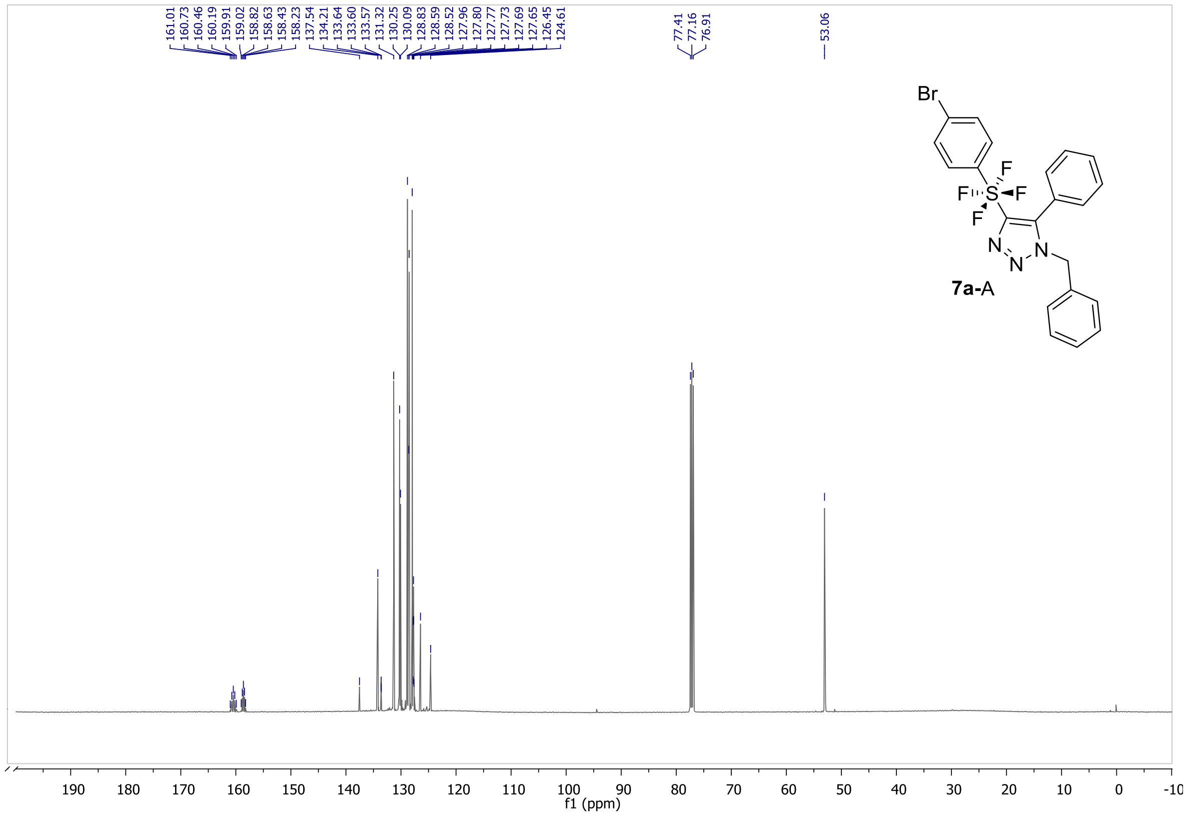
161.01
160.73
160.46
160.19
159.91
159.02
158.82
158.63
158.43
158.23
137.54
134.21
133.64
133.60
133.57
131.32
130.25
130.09
128.83
128.59
128.52
127.96
127.80
127.77
127.73
127.69
127.65
126.45
124.61
77.41
77.16
76.91
53.06
7a-A
f1 (ppm)

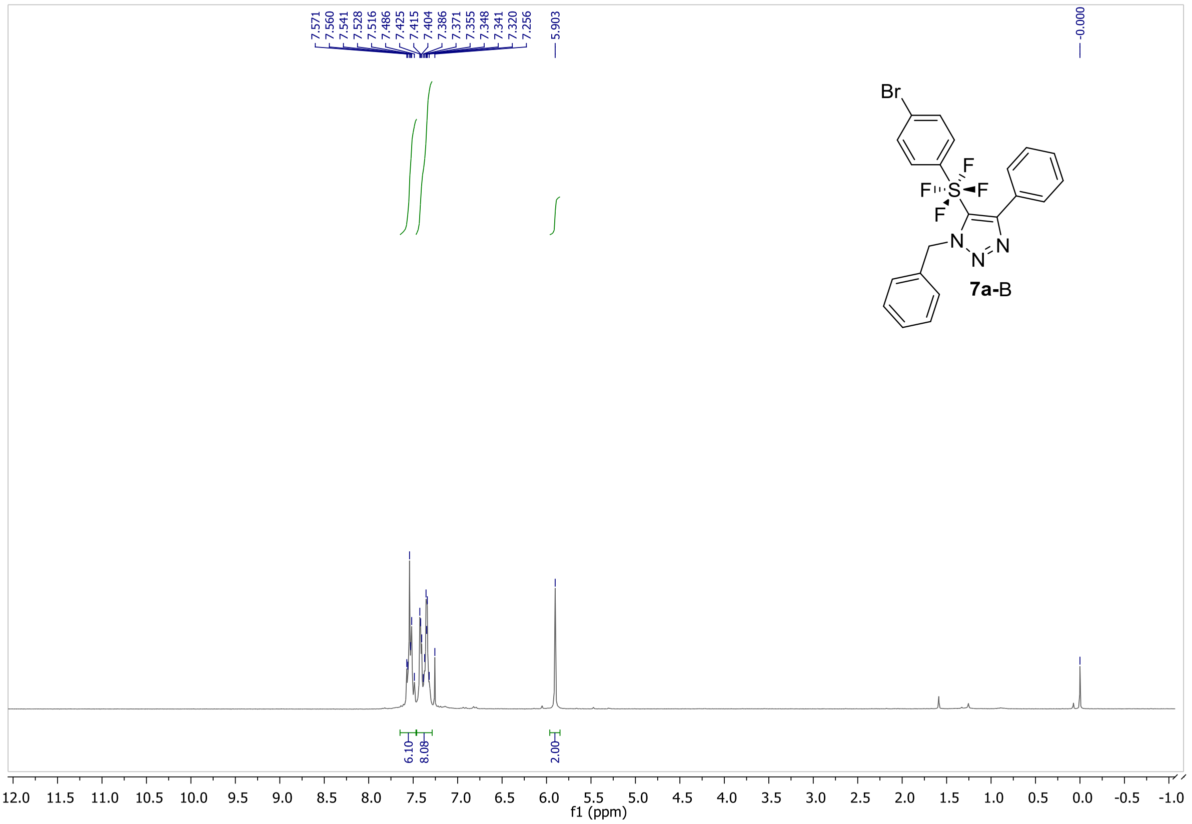
7.571
7.560
7.541
7.528
7.516
7.486
7.425
7.415
7.404
7.386
7.371
7.355
7.348
7.341
7.320
7.256
5.903
-0.000
7a-B
6.10
8.08
2.00
f1 (ppm)

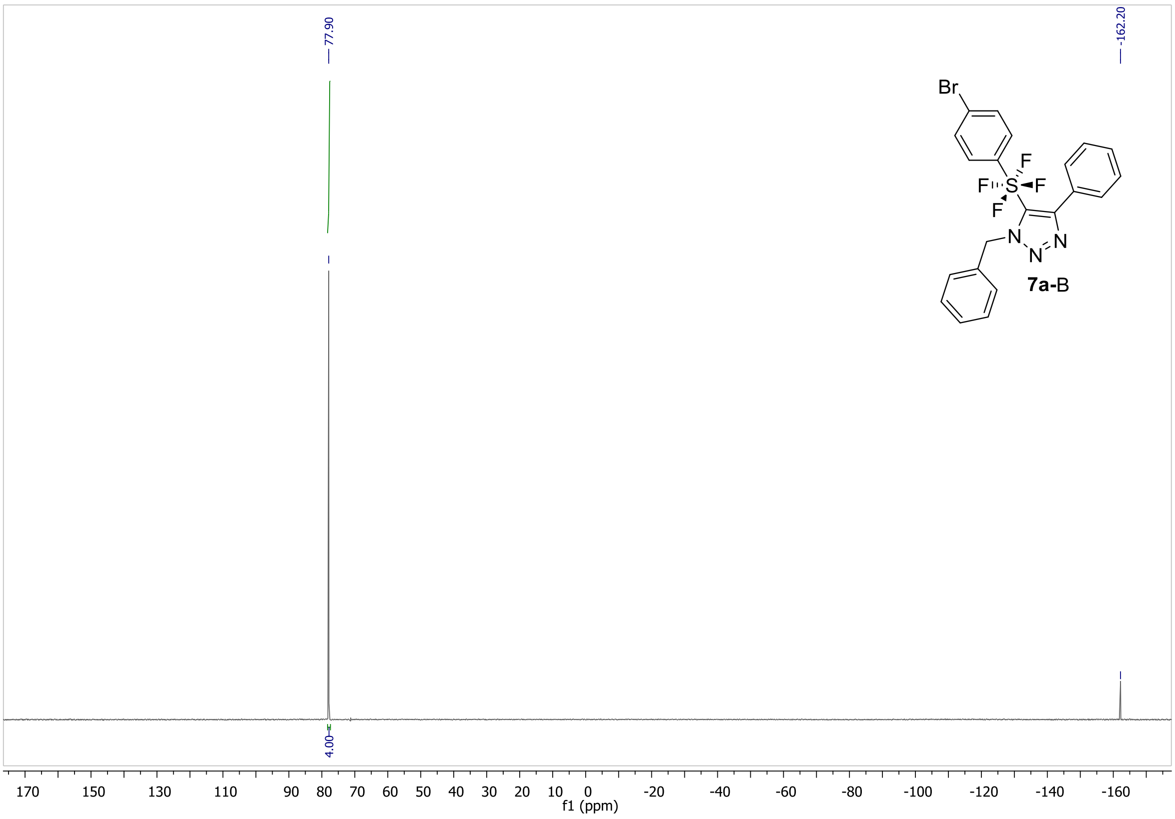

77.90
-162.20
Br
F
F
F
F
S
N
N
N
7a-B
4.00
170 150 130 110 90 80 70 60 50 40 30 20 10 0 -20 -40 -60 -80 -100 -120 -140 -160
f1 (ppm)

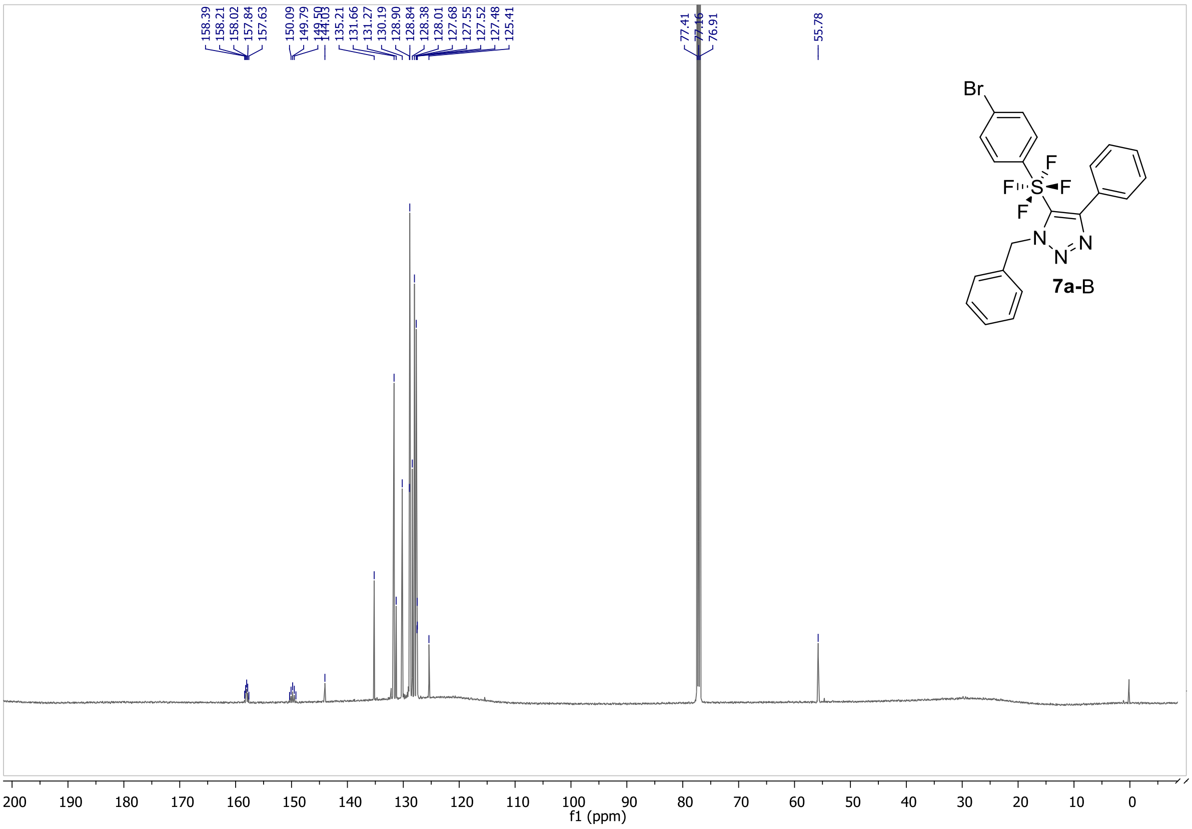

158.39
158.21
158.02
157.84
157.63
150.09
149.79
149.50
144.03
135.21
131.66
131.27
130.19
128.90
128.84
128.38
128.01
127.68
127.55
127.52
127.48
125.41
77.41
77.16
76.91
55.78
Br
F
F
F
F
S
N
N
N
7a-B
200 190 180 170 160 150 140 130 120 110 100 90 80 70 60 50 40 30 20 10 0
f1 (ppm)

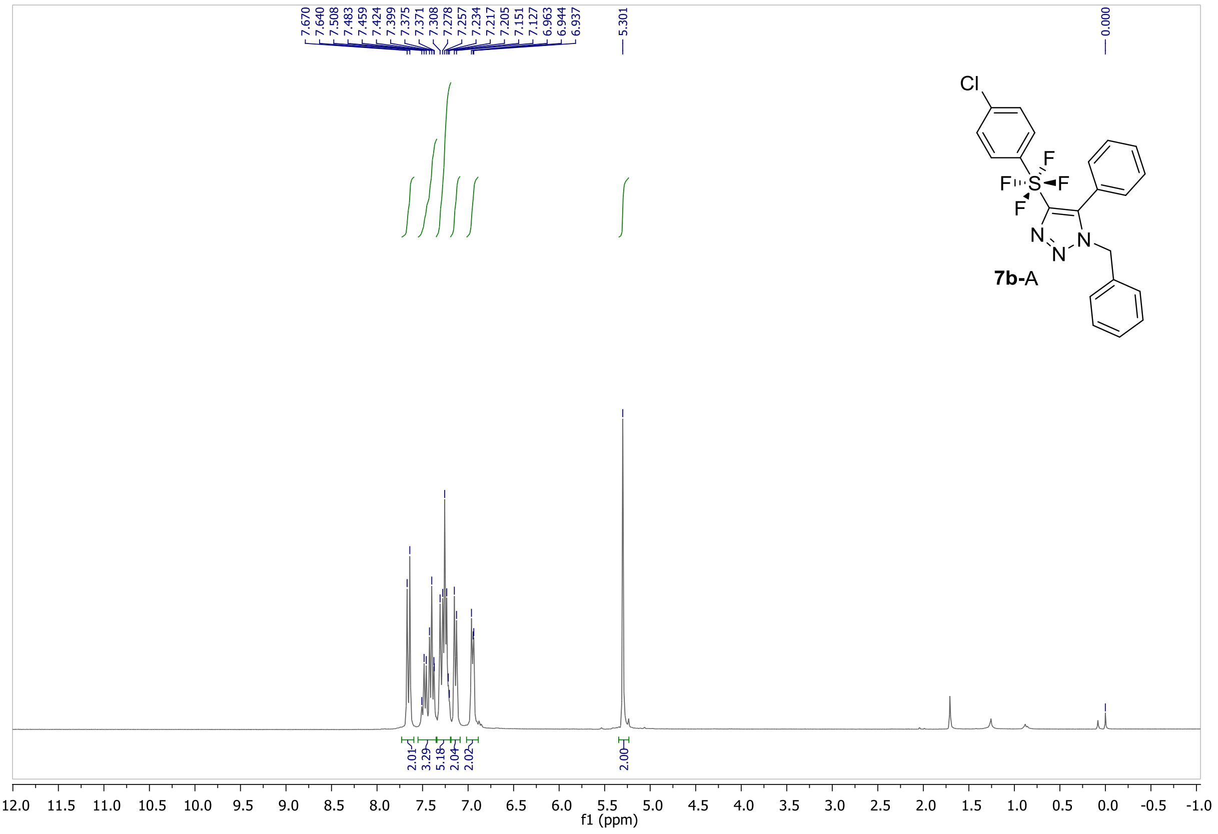
7.670
7.640
7.508
7.483
7.459
7.424
7.399
7.375
7.371
7.308
7.278
7.257
7.234
7.217
7.205
7.151
7.127
6.963
6.944
6.937
5.301
0.000
7b-A
2.01
3.29
5.18
2.04
2.02
2.00
f1 (ppm)

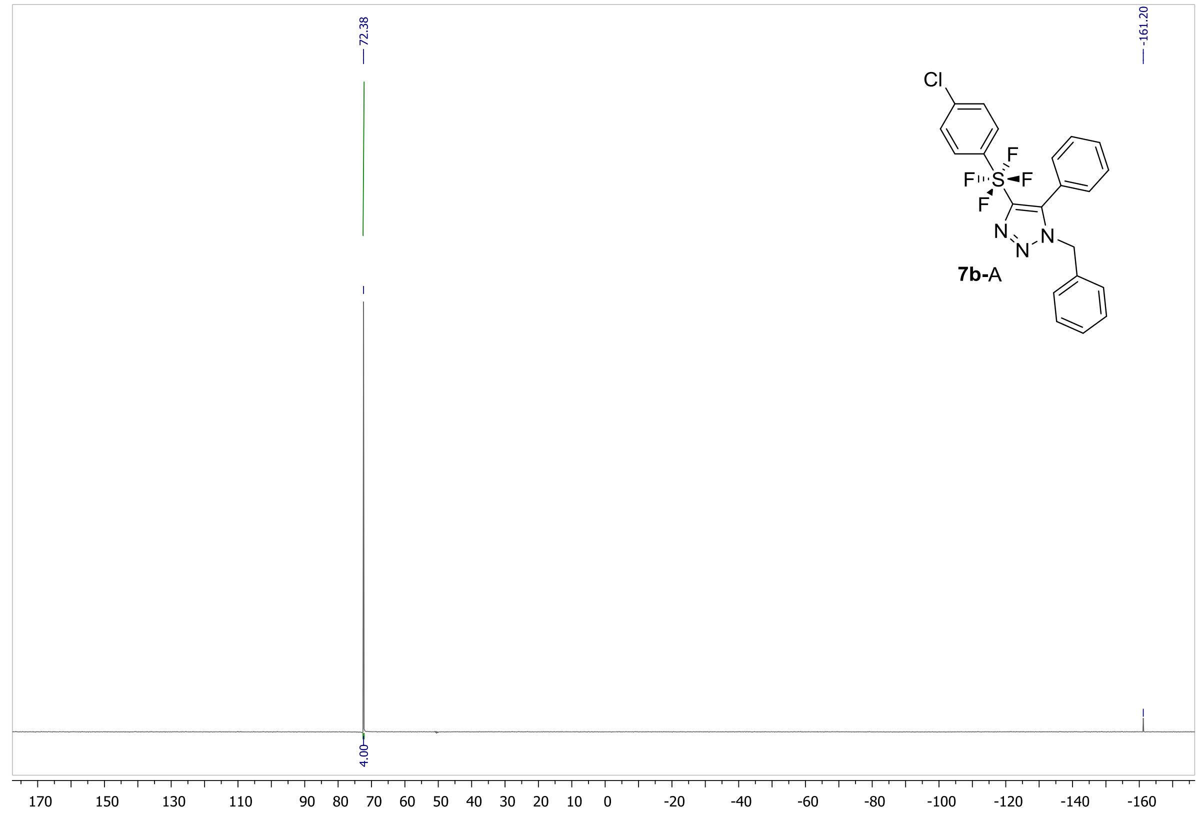
72.38
-161.20
7b-A
4.00

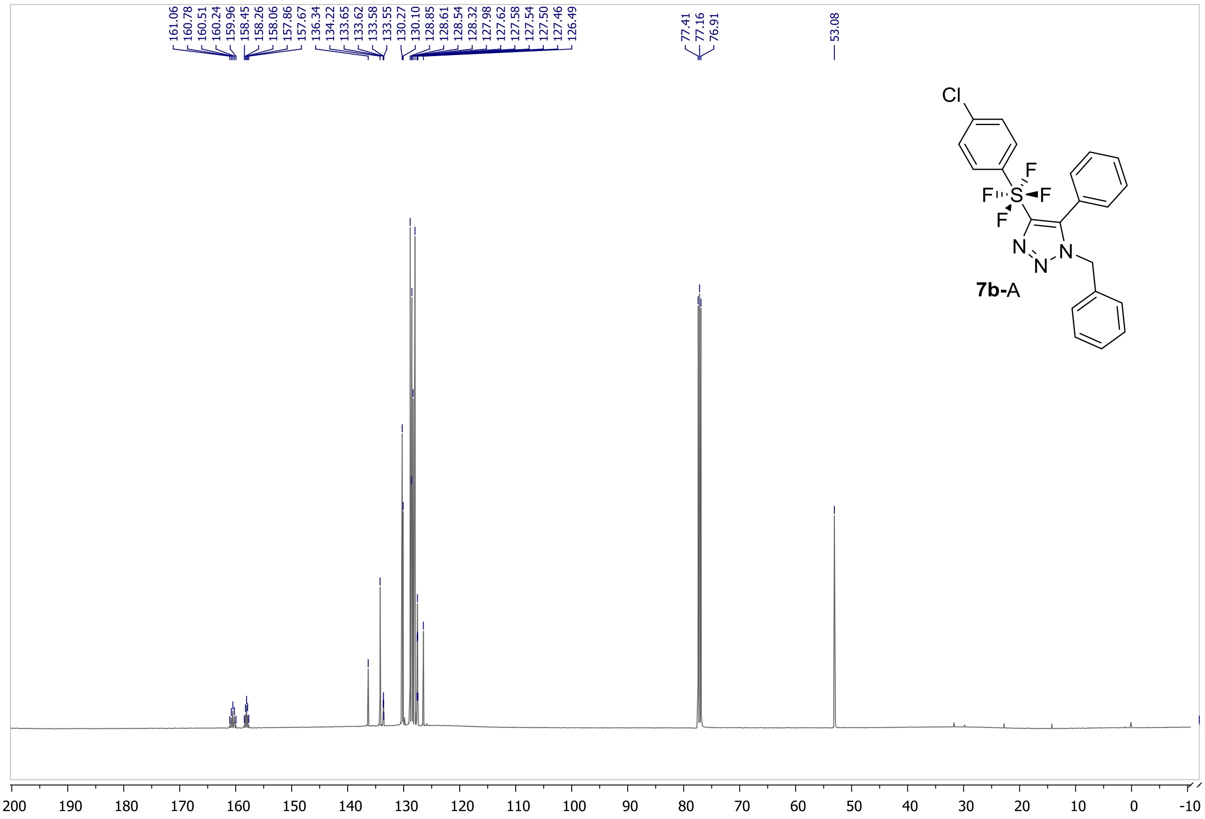
161.06
160.78
160.51
160.24
159.96
158.45
158.26
158.06
157.86
157.67
136.34
134.22
133.65
133.62
133.58
133.55
130.27
130.10
128.85
128.61
128.54
128.32
127.98
127.62
127.58
127.54
127.50
127.46
126.49
77.41
77.16
76.91
53.08
7b-A
200 190 180 170 160 150 140 130 120 110 100 90 80 70 60 50 40 30 20 10 0 -10

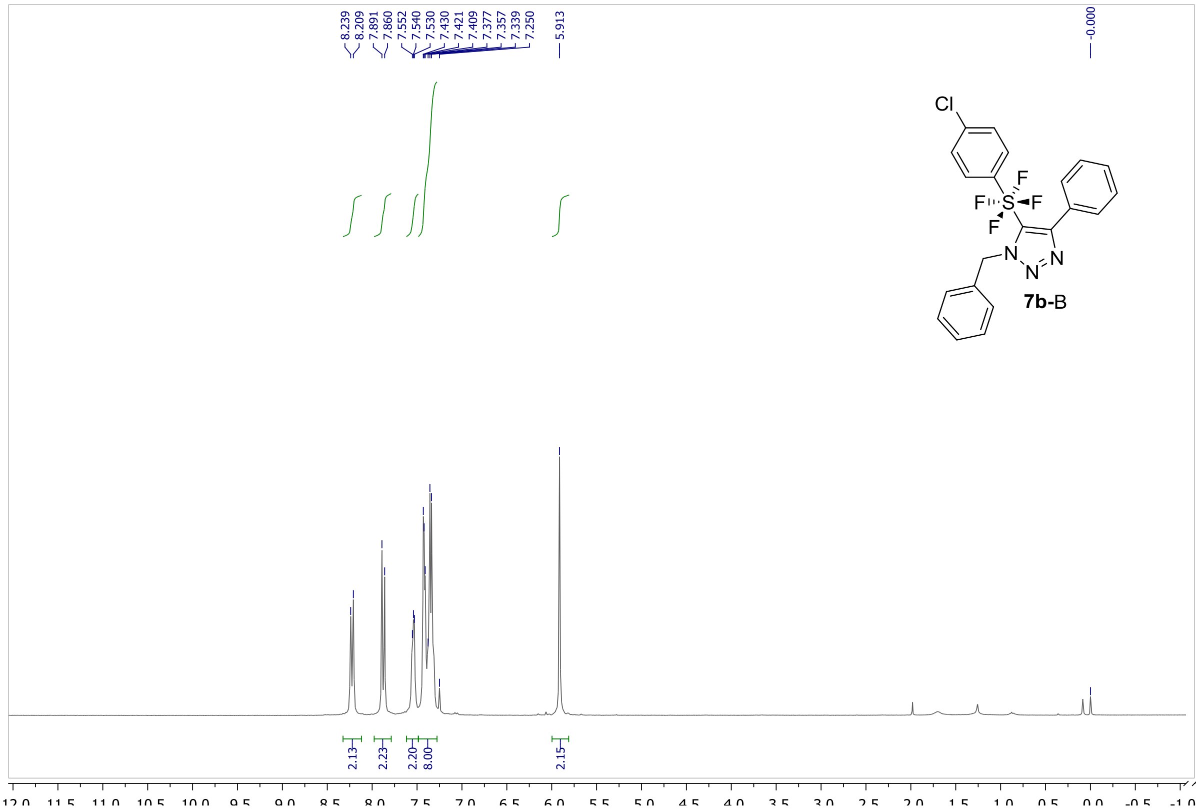
8.239
8.209
7.891
7.860
7.552
7.540
7.530
7.430
7.421
7.409
7.377
7.357
7.339
7.250
5.913
-0.000
7b-B
2.13
2.23
2.20
8.00
2.15

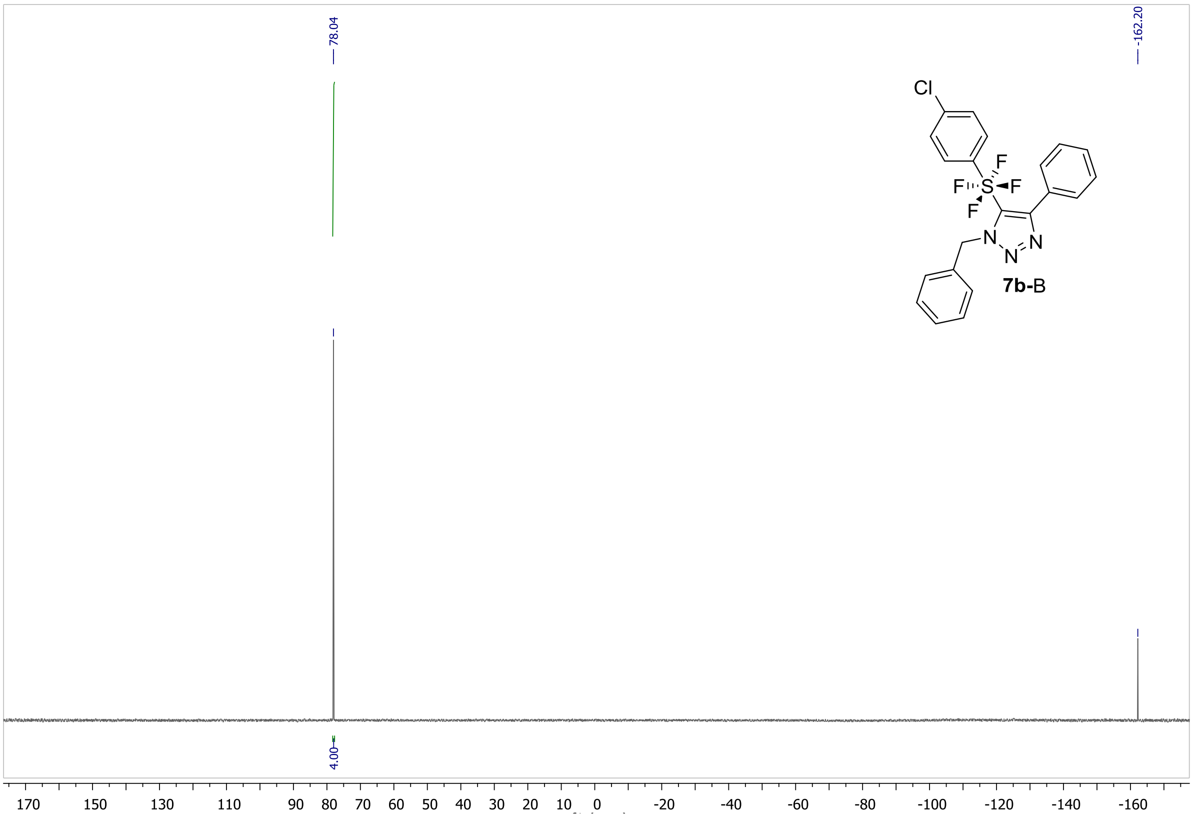
78.04
-162.20
Cl
F
F
F
F
S
N
N
N
7b-B
4.00
170
150
130
110
90
80
70
60
50
40
30
20
10
0
-20
-40
-60
-80
-100
-120
-140
-160

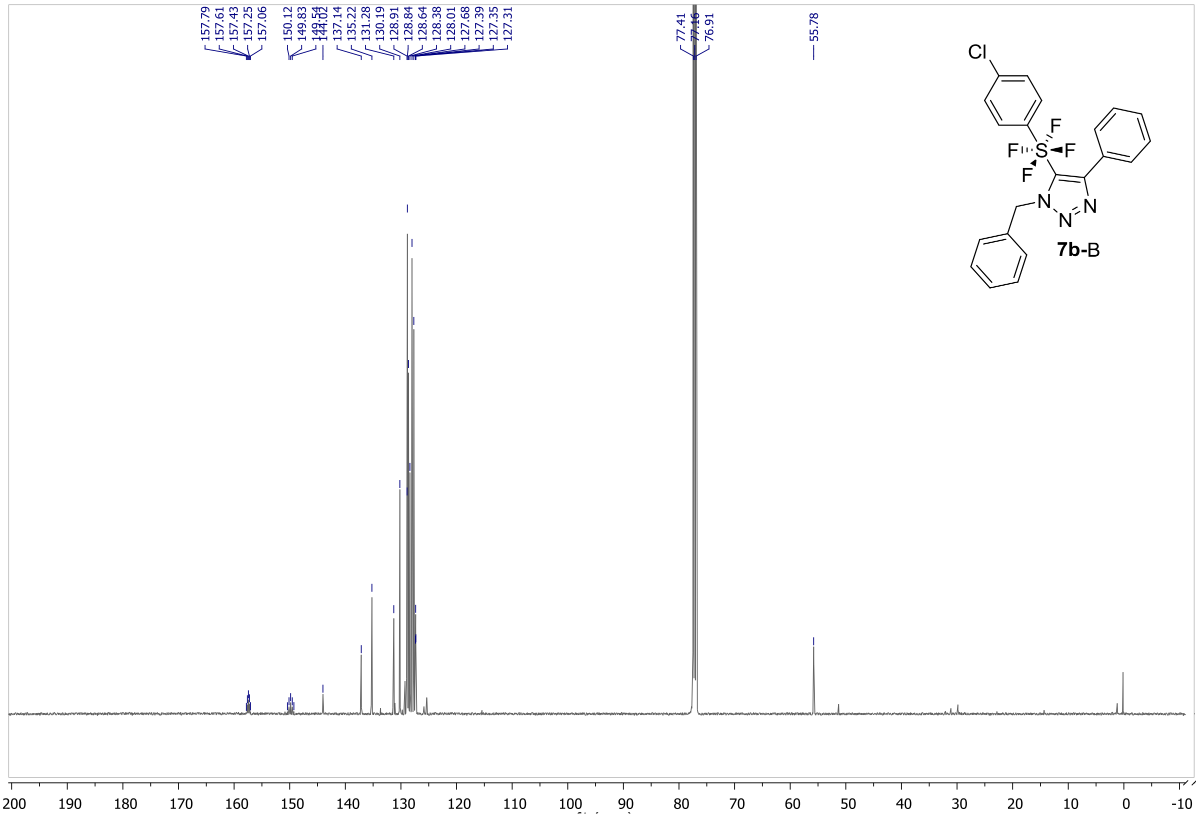
157.79
157.61
157.43
157.25
157.06
150.12
149.83
149.54
144.02
137.14
135.22
131.28
130.19
128.91
128.84
128.64
128.38
128.01
127.68
127.39
127.35
127.31
77.41
77.16
76.91
55.78
Cl
F
F
F
F
S
N
N
N
7b-B
200
190
180
170
160
150
140
130
120
110
100
90
80
70
60
50
40
30
20
10
0
-10

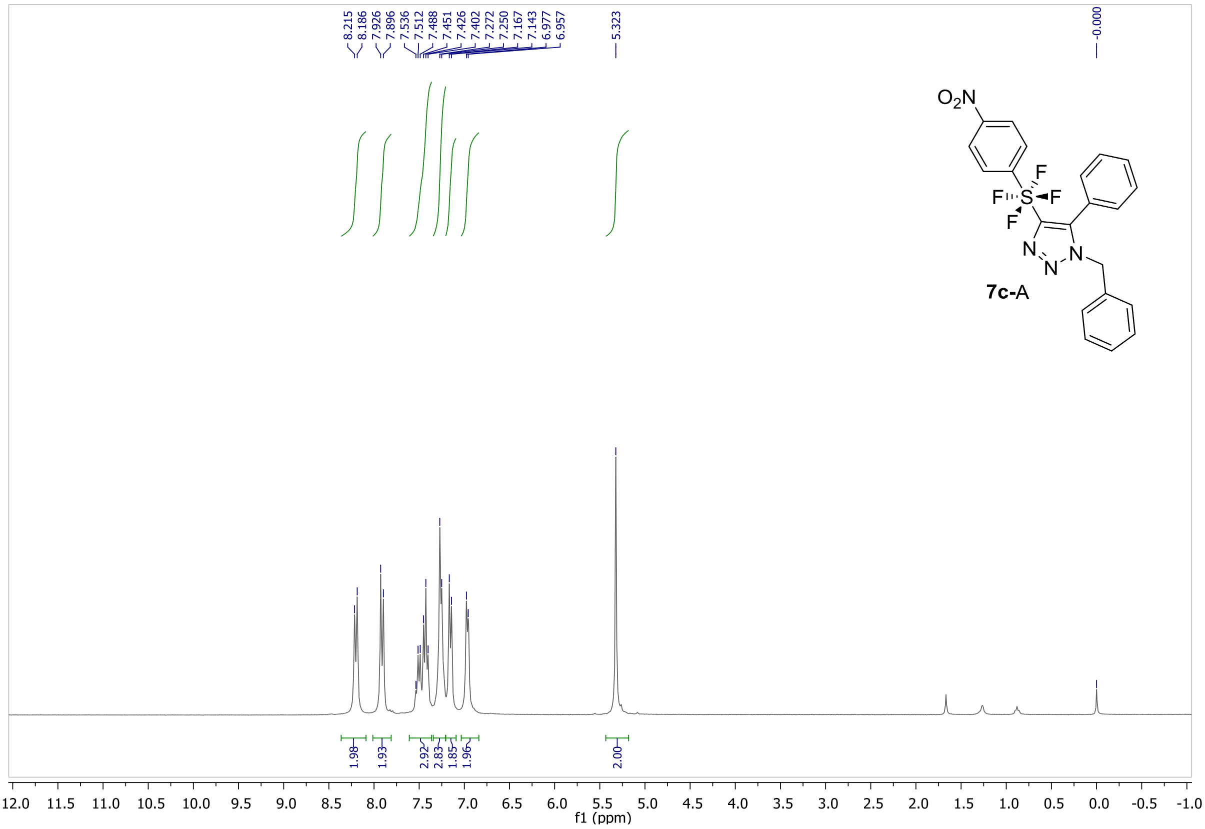
8.215
8.186
7.926
7.896
7.536
7.512
7.488
7.451
7.426
7.402
7.272
7.250
7.167
7.143
6.977
6.957
5.323
-0.000
O_2N
F
S
N
7c-A
1.98
1.93
2.92
2.83
1.85
1.96
2.00
f1 (ppm)

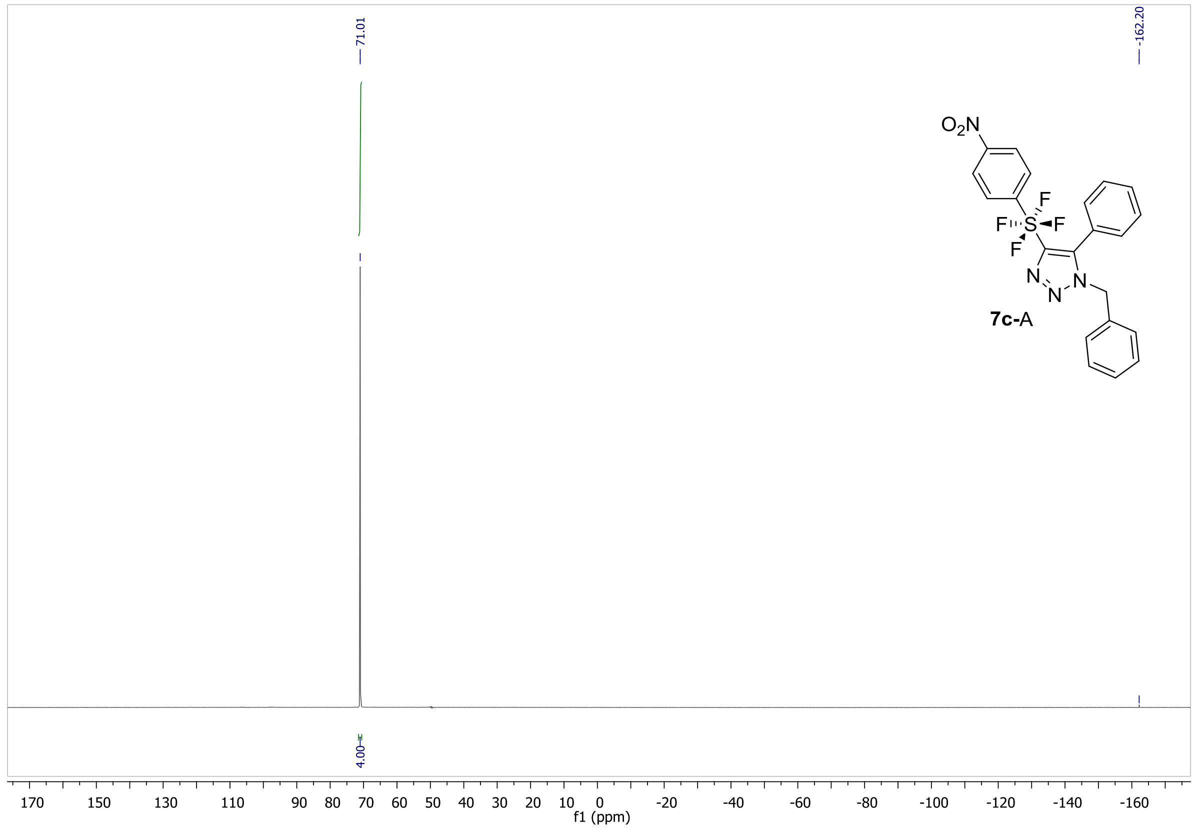
71.01
-162.20
O_2N
F
S
N
7c-A
4.00
f1 (ppm)

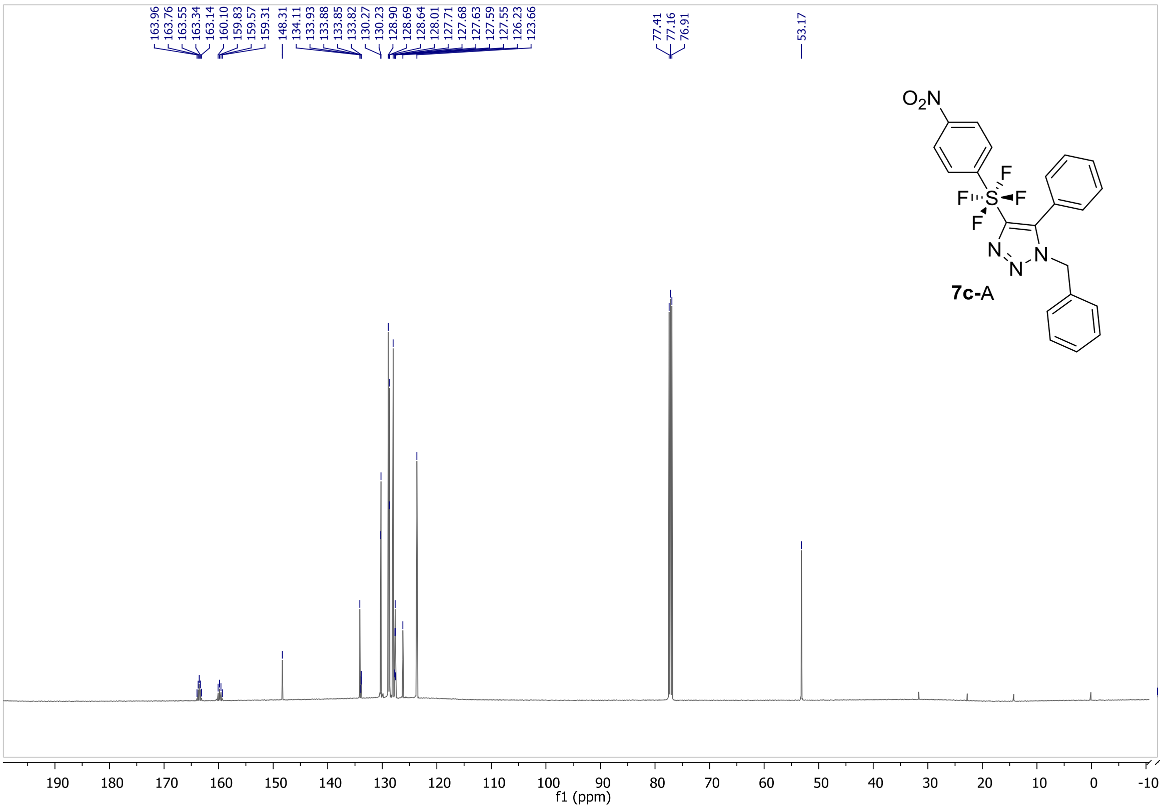

163.96
163.76
163.55
163.34
163.14
160.10
159.83
159.57
159.31
148.31
134.11
133.93
133.88
133.85
133.82
130.27
130.23
128.90
128.69
128.64
128.01
127.71
127.68
127.63
127.59
127.55
126.23
123.66
77.41
77.16
76.91
53.17
7c-A
f1 (ppm)

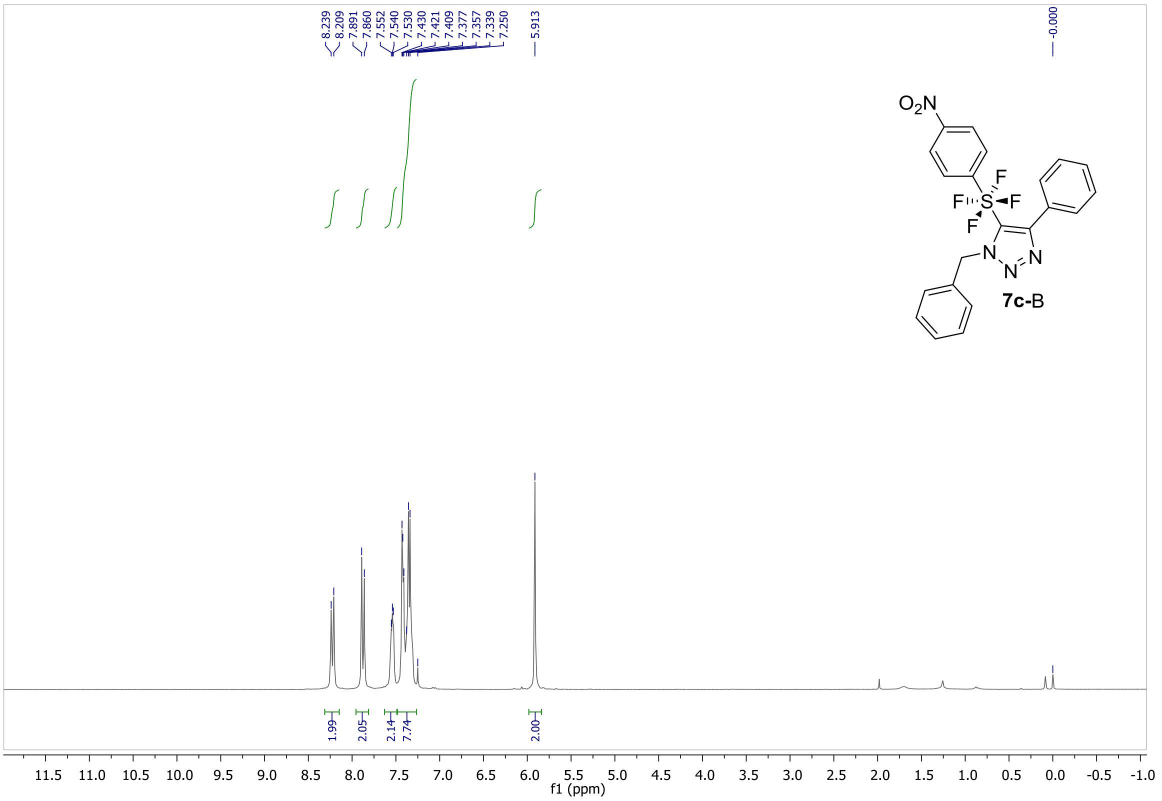

8.239
8.209
7.891
7.860
7.552
7.540
7.530
7.430
7.421
7.409
7.377
7.357
7.339
7.250
5.913
-0.000
7c-B
1.99
2.05
2.14
7.74
2.00
f1 (ppm)

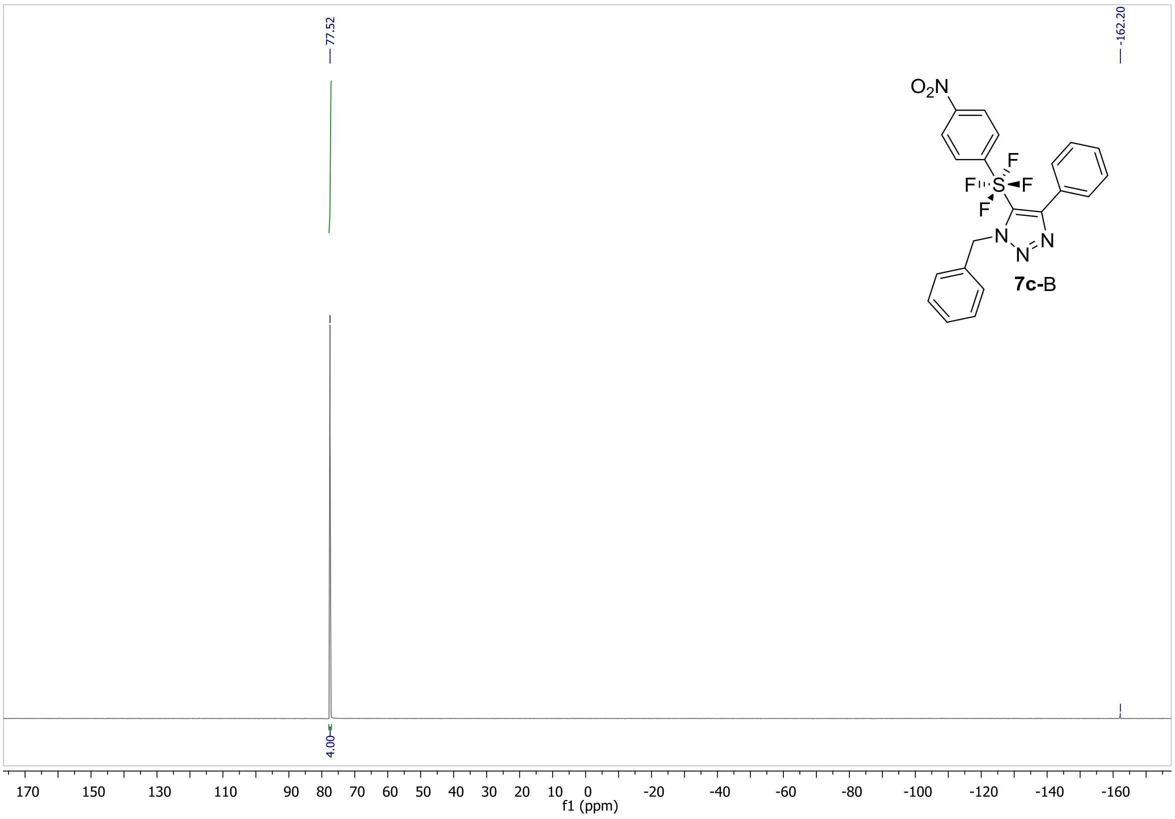
77.52
-162.20
O_2N
F
F
S
F
F
N
N
N
7c-B
4.00
170
150
130
110
90
80
70
60
50
40
30
20
10
0
-20
-40
-60
-80
-100
-120
-140
-160
f1 (ppm)

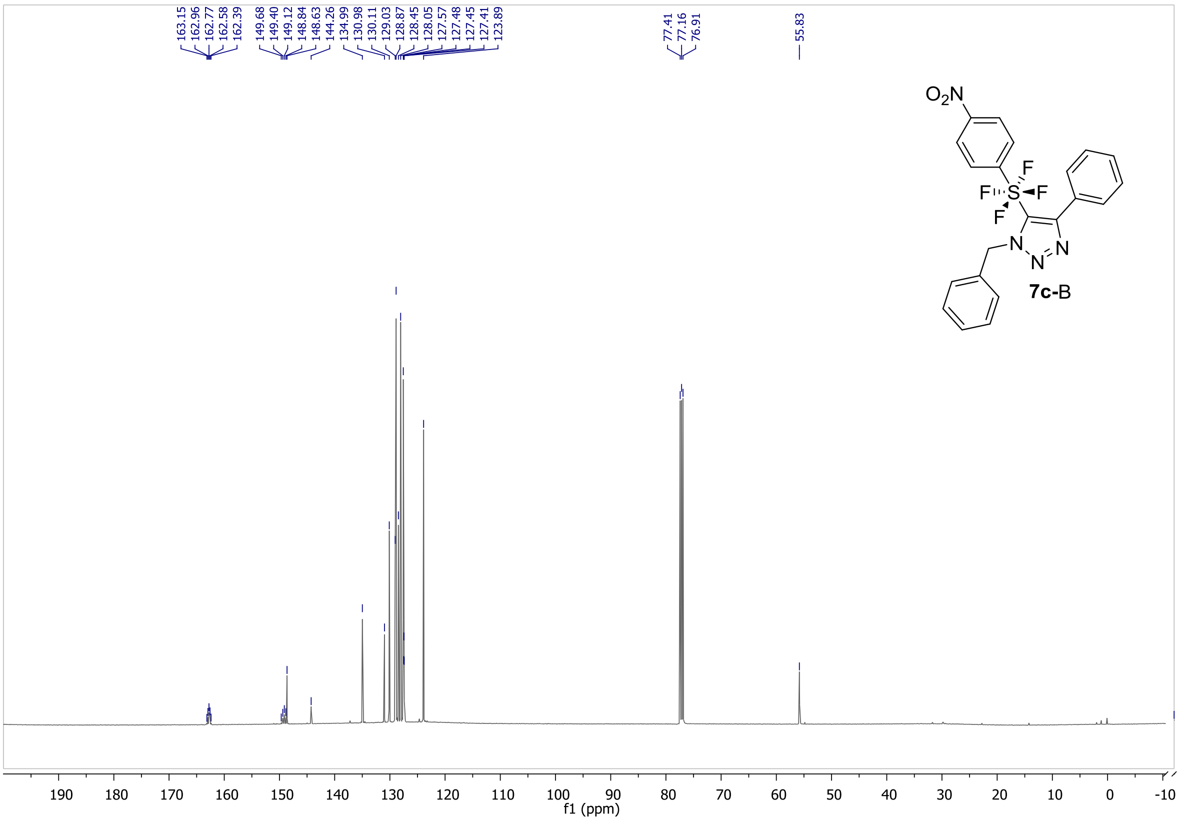
163.15
162.96
162.77
162.58
162.39
149.68
149.40
149.12
148.84
148.63
144.26
134.99
130.98
130.11
129.03
128.87
128.45
128.05
127.57
127.48
127.45
127.41
123.89
77.41
77.16
76.91
55.83
O_2N
7c-B
190
180
170
160
150
140
130
120
110
100
90
80
70
60
50
40
30
20
10
0
-10
f1 (ppm)

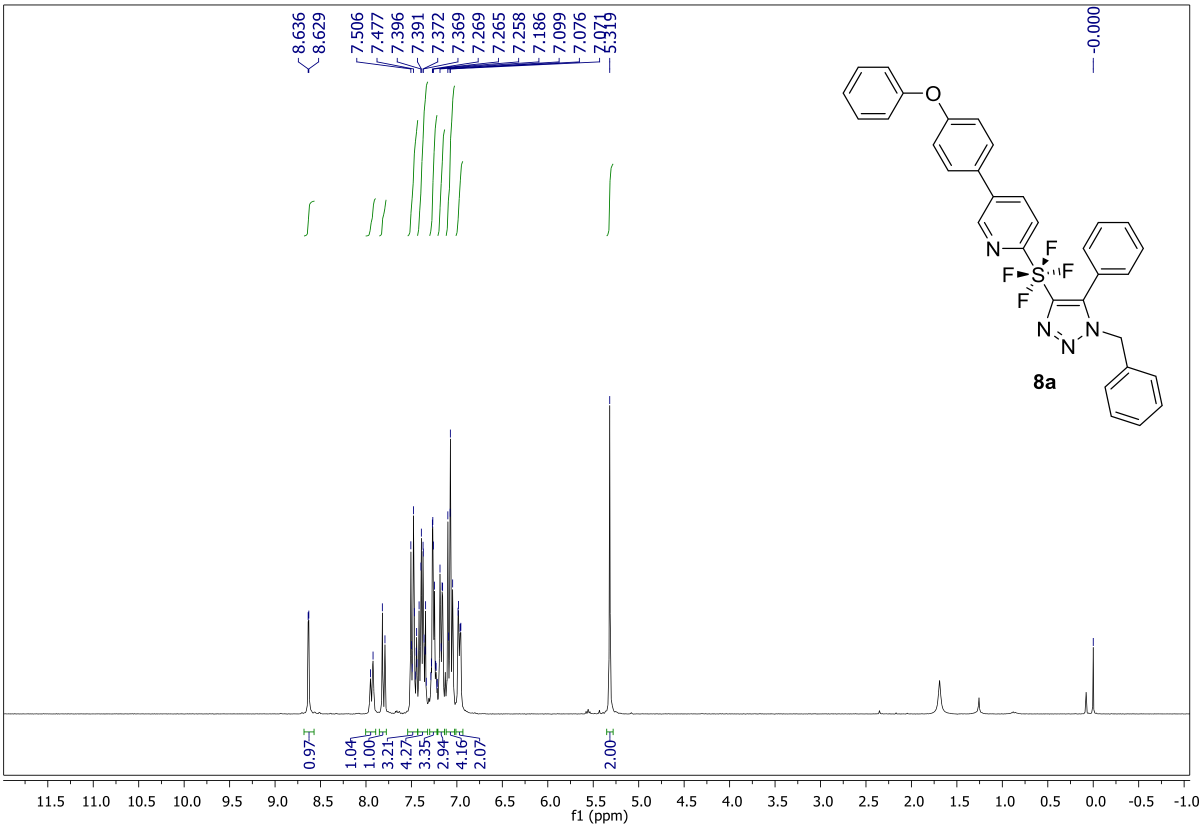

8.636
8.629
7.506
7.477
7.396
7.391
7.372
7.369
7.269
7.265
7.258
7.186
7.099
7.076
7.071
5.319
-0.000
0.97
1.04
1.00
3.21
4.27
3.35
2.94
4.16
2.07
2.00
8a
f1 (ppm)

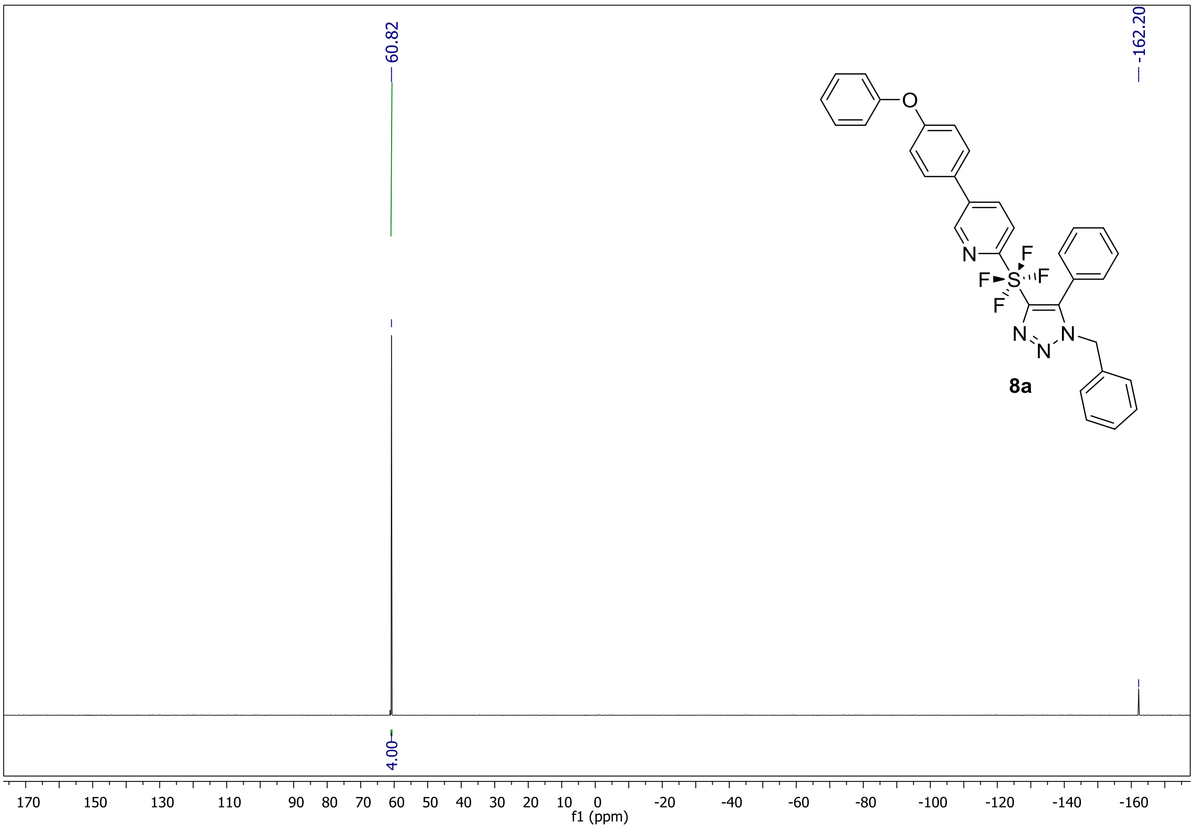

60.82
-162.20
4.00
8a
f1 (ppm)

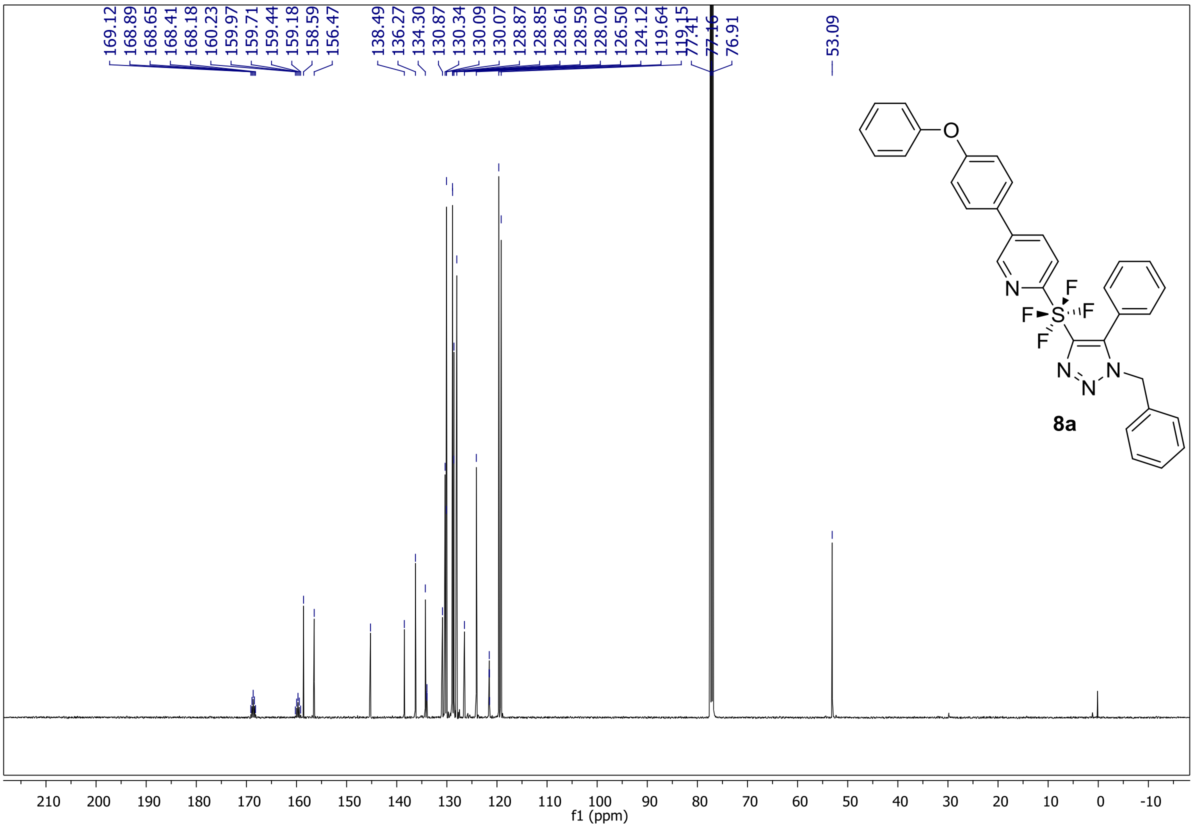
169.12
168.89
168.65
168.41
168.18
160.23
159.97
159.71
159.44
159.18
158.59
156.47
138.49
136.27
134.30
130.87
130.34
130.09
130.07
128.87
128.85
128.61
128.59
128.02
126.50
124.12
119.64
119.15
77.41
77.16
76.91
53.09
8a
210 200 190 180 170 160 150 140 130 120 110 100 90 80 70 60 50 40 30 20 10 0 -10
f1 (ppm)

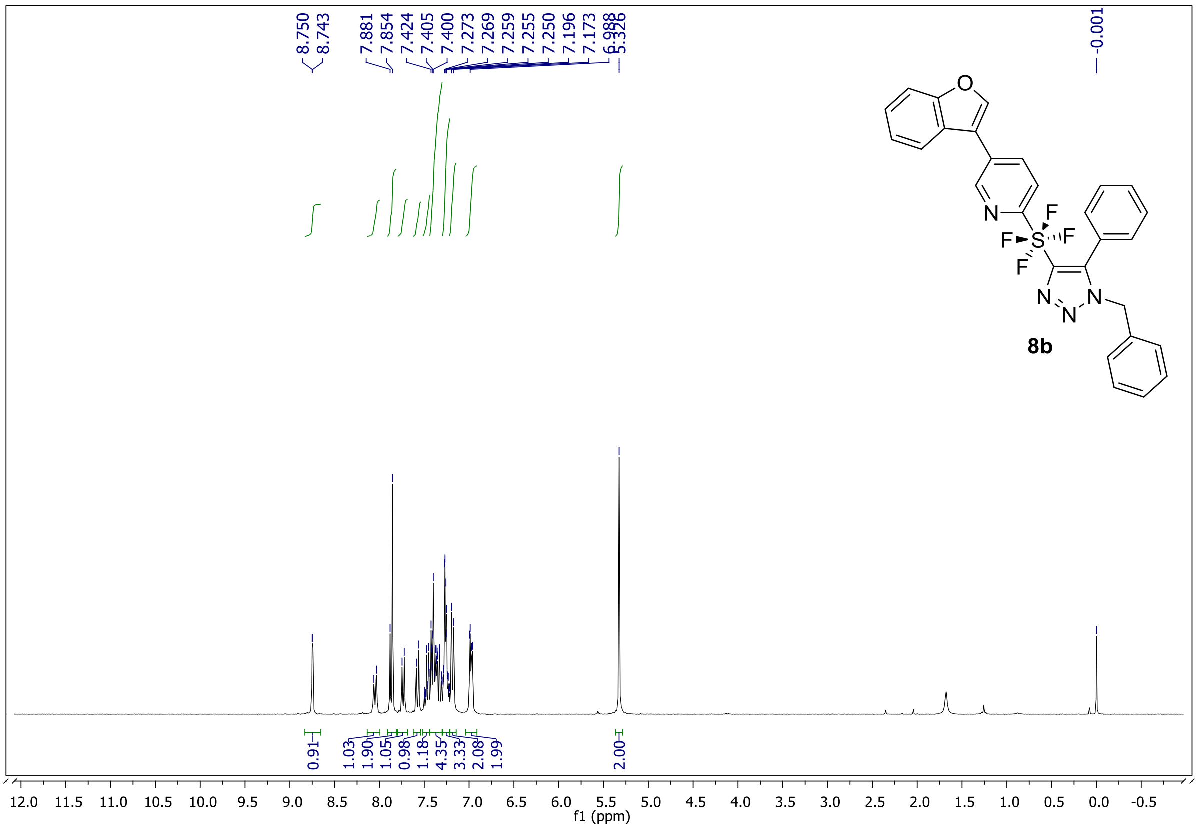
8.750
8.743
7.881
7.854
7.424
7.405
7.400
7.273
7.269
7.259
7.255
7.250
7.196
7.173
6.988
5.326
-0.001
8b
0.91
1.03
1.90
1.05
0.98
1.18
4.35
3.33
2.08
1.99
2.00
12.0 11.5 11.0 10.5 10.0 9.5 9.0 8.5 8.0 7.5 7.0 6.5 6.0 5.5 5.0 4.5 4.0 3.5 3.0 2.5 2.0 1.5 1.0 0.5 0.0 -0.5
f1 (ppm)

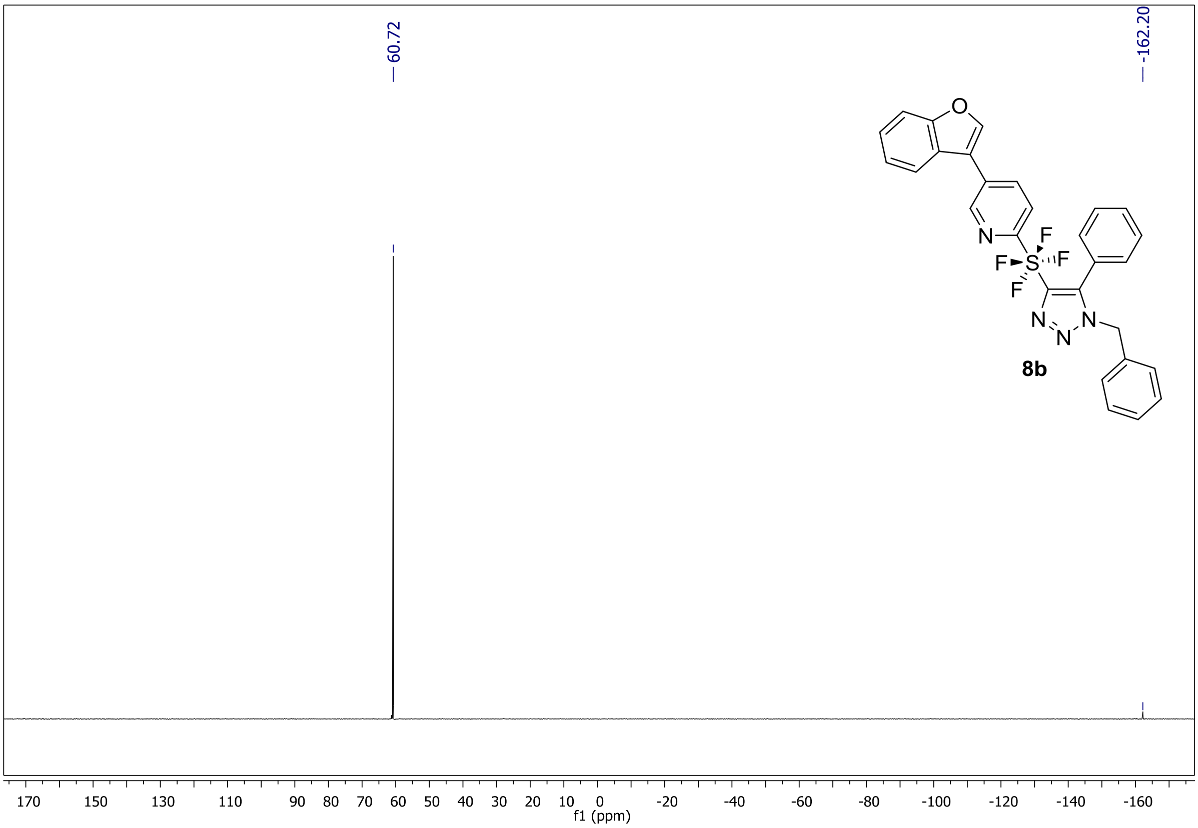
60.72
-162.20
8b
170 150 130 110 90 80 70 60 50 40 30 20 10 0 -20 -40 -60 -80 -100 -120 -140 -160
f1 (ppm)

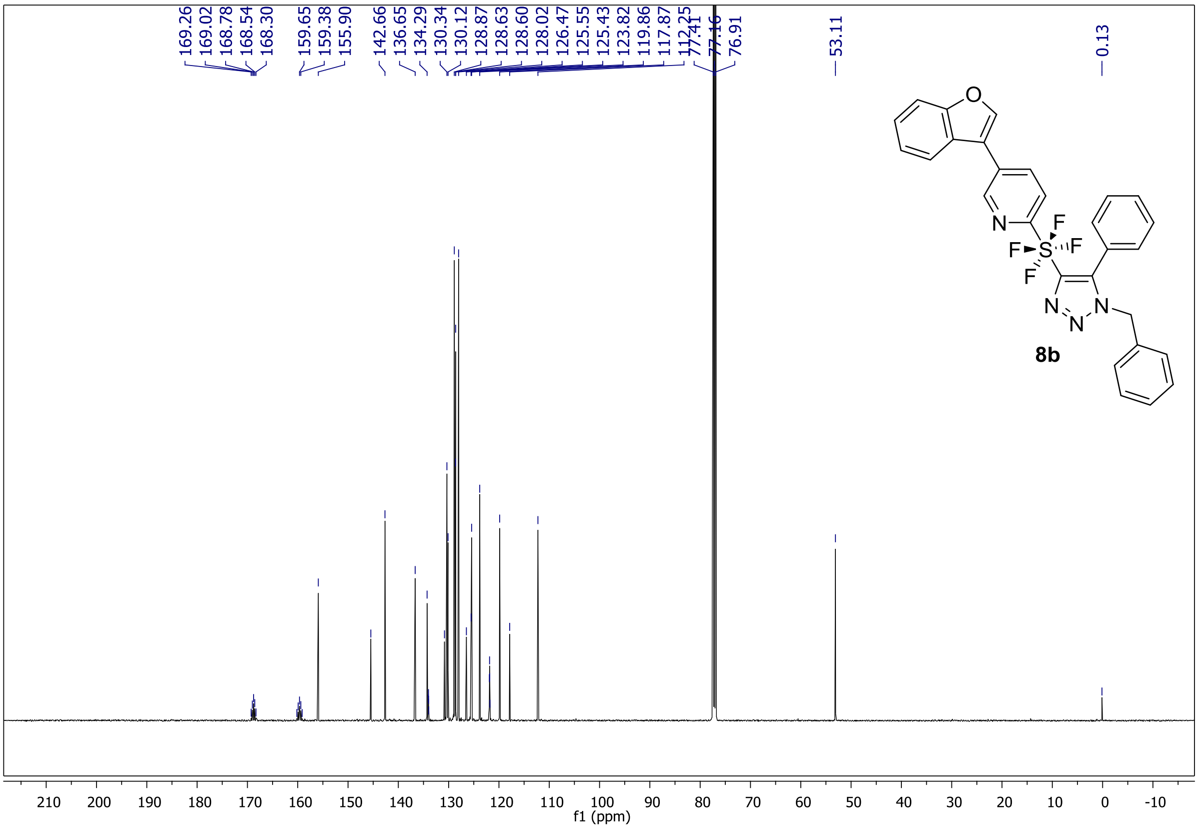
169.26
169.02
168.78
168.54
168.30
159.65
159.38
155.90
142.66
136.65
134.29
130.34
130.12
128.87
128.63
128.60
128.02
126.47
125.55
125.43
123.82
119.86
117.87
112.25
77.41
77.16
76.91
53.11
0.13
8b
210 200 190 180 170 160 150 140 130 120 110 100 90 80 70 60 50 40 30 20 10 0 -10
f1 (ppm)
